# A General Group Testing Strategy for Discovering Chemical Cooperativity

**DOI:** 10.1002/anie.202525278

**Published:** 2026-02-03

**Authors:** Philipp M. Pflüger, Felix Katzenburg, Frederik Sandfort, Michael Teders, Adrián Gómez‐Suárez, Eric A. Standley, Matthew N. Hopkinson, Constantin G. Daniliuc, Andreas Heuer, Frank Glorius

**Affiliations:** ^1^ Organisch‐Chemisches Institut Universität Münster Münster Germany; ^2^ Institut für Physikalische Chemie Universität Münster Münster Germany

**Keywords:** combinatorial designs, cooperativity, group testing, screening, trifluoromethylthiolation

## Abstract

The combinatorial explosion inherent to multi‐component systems limits their experimental exploration and ultimately chemical discovery. Here, we introduce a statistics‐based group‐testing strategy, which we couple with luminescence quenching assays to efficiently identify cooperative molecular interactions. Utilizing the quenching of a photosensitizer as a quick readout for chemical activity, 4,950 substrate pairs were screened in only 504 experiments, enabled through a combinatorial design theory‐based pooling approach and iterative deconvolution. Therefore, two algorithms—a greedy algorithm for group design and an iterative sectioning deconvolution method to resolve active pairs—were implemented. Fifteen cooperative pairs were identified, and the nature of their interactions and the resulting electronic perturbations were investigated. In a systematic follow‐up screen, it was found that the identified active pairs exhibit high reactivity towards a broad group of reaction partners. One such pair led to the discovery of a bench‐stable reagent, enabling efficient and regioselective trifluoromethylthiolation reactions. This work establishes a broadly applicable framework for accelerating the discovery of cooperative reactivity through optimized experimental designs.

## Graphical abstract

A combinatorial design theory‐based group testing strategy coupled with luminescence screening to uncover cooperative interactions in a large combinatorial space. Iterative deconvolution identifies reactive molecular pairs and enables the discovery of trifluoromethylthiolation (SCF_3_) reagents.

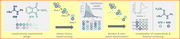

## 1 Introduction

**FIGURE 1:**
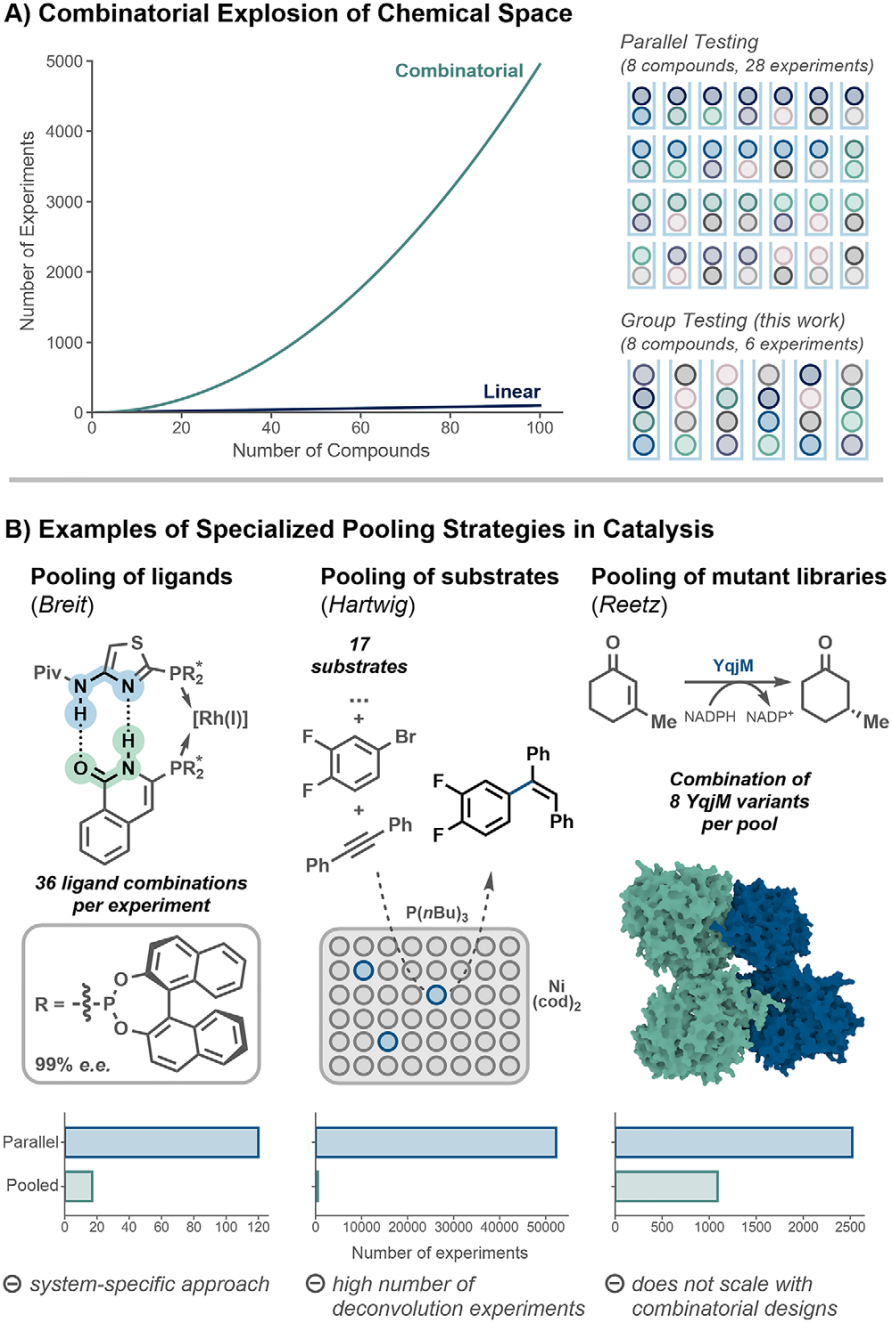
(A) The investigation of pairwise interactions across large molecular libraries becomes experimentally intractable for combinatorial search spaces. (B) Examples of specialized pooling strategies in catalysis [37, 38].

The exploration of chemical space is a central theme in drug development, catalyst design, functional molecule synthesis, and, fundamentally, all fields seeking compounds with a set of desired properties or activities. Although the concept of chemical space—cited to contain between 10^60^ and 10^200^ substances [1, 2] —was developed in the 1990s, it has been experiencing a renaissance. The combination of machine learning [3, 4], virtual screening strategies [5, 6, 7, 8], and ultra‐large compound spaces [9, 10, 11] has enabled scientists to evaluate previously elusive numbers of molecules. Experimental screening, however, has not kept pace with the number of candidates that can be generated through computational exploration [12]. While focused libraries built around a specific scaffold or variant can typically be assessed with parallel experimental and classical analytical techniques, highly diverse libraries can quickly render these approaches insufficient. Combinatorial designs exploring interactions between two or more compounds, in particular, can result in an intractably large number of potential experiments even for modest numbers of candidates (Figure 1A). Albeit progressive integration of high‐throughput screening [13] and automation has expanded the scale of chemical screening campaigns [14, 15, 16] to tens of thousands of experiments [17, 18, 19, 20, 21, 22], this still only corresponds to a fraction of available combinatorial ligand [23, 24], catalyst [25, 26] or make‐on‐demand compound libraries [9, 10].

In synthetic chemistry, this increase in possible combinations and complexity renders the discovery of multi‐component reactions, synergistic catalysis or cooperative interactions challenging [27, 28, 29, 30]. The combinatorial explosion arising from combining multiple substrates, ligands, or catalysts has previously been addressed through pooling methods (Figure 1B) [31, 32, 33, 34]. Instead of testing each possible combination of candidates individually, pools of multiple combinations are tested together, reducing experimental effort and analysis time. Only in case of positive results, hits are investigated further by deconvolving pools to identify the active combination. Breit and coworkers have applied this approach to a combinatorial ligand library for a rhodium‐catalyzed asymmetric hydrogenation [35]. From 120 self‐assembling ligands, the optimal, highly selective catalytic system was identified in 17 experiments by pooling up to 36 ligand combinations per experiment. In another example, Hartwig and coworkers screened an array of 17 substrates, 15 metal precursors and 23 ligands (i.e., 50,000 possible combinations) in under 500 experiments, resulting in the discovery of a hydroarylation and hydroamination reaction. [36].

Beyond organic chemistry, pooling has also found application in biocatalysis, where screening remains a bottleneck for directed evolution [39, 40, 41]. To identify more active enolate‐reductase YqjM variants for the asymmetric reduction of 3‐methylcyclohexenone, Reetz and coworkers tested pools of eight cell cultures for conversion [42]. By deconvolving only pools that exceed a conversion threshold, the number of experiments could be reduced by more than 50% without missing true positives.

These examples highlight the great potential of pooling strategies, especially when screening for pairwise interactions. However, the design of pools is typically carried out in a naïve way, combining all candidates into one pool [36], selecting pool sizes based on labware dimensions [42] or defining arbitrary pool sizes [35, 36], This increases the experimental effort required to screen pools as well as the number of tests needed to identify the reactive entities. This deconvolution frequently results in experimental burdens exceeding those of the initial screen [35]. In this work, we are moving beyond simple pooling approaches to statistics‐based group testing, not only reducing but minimizing the number of experiments needed to identify hits. We introduce algorithms for pool design and deconvolution applicable beyond chemistry. The numerical design of groups can be based either on precompiled covering tables or on an adaptive greedy algorithm conceptualized by MacDonald and coworkers [43]. We advance these methods by implementing the greedy group design for pairs and triples and developing an adaptive, multi‐stage deconvolution procedure for efficient scaling to very large candidate spaces and higher‐order combinations. We showcase the utility of our approach by identifying cooperative interactions across 4,950 molecular pairs, performing as few as 393 experiments, and identifying an unprecedented reagent for trifluoromethylations. During the finalization of this manuscript, Jacobsen and coworkers have published a pooling‐deconvolution workflow for the discovery of cooperative catalysts [44].

## 2 Results and Discussion

**FIGURE 2:**
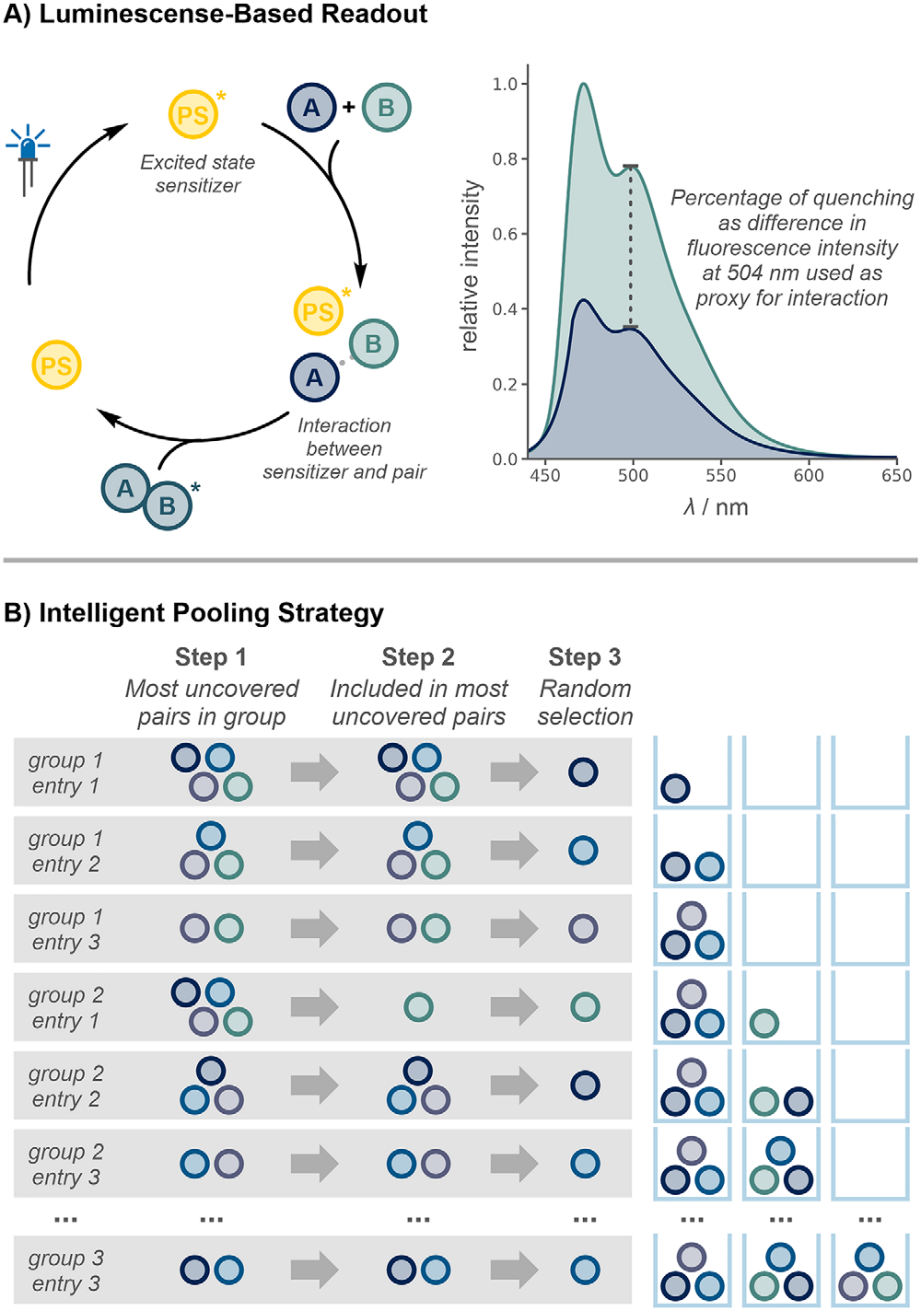
(A) Mechanism‐based luminescence quenching screen. The excited‐state photosensitizer (PS*) is quenched upon interaction with a cooperative substrate pair, leading to decreased fluorescence intensity. The quenching percentage, determined from the emission spectrum, serves as a readout to identify active groups. (B) Example of the greedy group design algorithm for four compounds and a group size of three. Substrates are combined into pools based on algorithmic selection to maximize coverage of unique pairs while minimizing redundancy. Each position is assigned by 1) identifying the compound(s) that, if added to the current group, would result in the coverage of the most uncovered pairs, 2) choosing the compound(s) from the finalists identified in step 1, that is included in the most uncovered pairs, 3) selecting a compound from the finalists from step 2 at random. This process is repeated until all pairs are covered and iterated for 1000 times to find the best solution.

Our group has previously developed a mechanism‐based screening approach for the discovery of photochemical reactions [45, 46, 47]. In contrast to most screening methods, which try to discover a chemical reaction by its product, this approach instead focuses on a substrate's activity in the key mechanistic step of a reaction. In photochemistry, this step is the quenching of an excited‐state photosensitizer by the substrate [48, 49, 50]. Luminescence spectroscopy can be used to detect the decrease in emission from the photosensitizer, providing a rapid and scalable readout to identify active substrates (Figure 2A). We envisioned that this strategy could not only be applied to identify individual quenchers, but also be applied to identify molecular pairs that cooperatively quench a photosensitizer, meaning that the quenching observed for a mixture of both substrates exceeds the sum of quenching observed for the individual substrates [30]. As cooperative interactions (i.e., quenching) are expected to be rare for a given set of molecules, a relatively extensive search space has to be considered to maintain high chances of success. However, the number of experiments required to test all possible pairwise combinations grows binomially with the number of molecules. We therefore reasoned that testing pools of molecules would increase the hit rate while reducing the number of required spectroscopic measurements by an order of magnitude. To test this concept of a luminescence‐based cooperativity screen, [Ir(dF(CF_3_)ppy)_2_(dtbpy)][PF_6_] (**PS**) was selected as photosensitizer. Its high excited‐state redox potentials (*E* ^1/2^(PS*/**PS** ^−^)  =  +1.21 V vs SCE and *E* ^1/2^(**PS** ^+^/PS*)  =  −0.89 V vs SCE) and high triplet excited‐state energy (*E*_T_  =  62 kcal/ mol) provide a large window for possible quenching processes [51].

To simplify the identification of chemical cooperativity in subsequent experiments, 100 substrates that do not interact with the excited‐state photosensitizer individually were identified and are referred to as non‐quenchers (Table S1). The non‐quenchers were randomly selected from stock compounds in our library, preselected solely on the basis of solubility in acetonitrile, while ensuring that diverse functional groups and their potential underlying activities were represented. When searching for cooperative interactions in a set of 100 substrates, 4,950 dual combinations (i.e., pairs) have to be considered—permitting manual testing of all pairs by luminescence spectroscopy. However, the number of required experiments can be significantly reduced by employing a group‐testing strategy, akin to medical screening methods for genetic disorders [52] or diseases at low prevalence [53]. Ideally, groups would be designed so that each possible pairing for the set of *n* = 100 molecules is present in exactly one group of size *k* [54]. However, there is no general analytical method to construct these optimal designs, called *Steiner Systems*, and for many (*n*,*k*)‐combinations an optimal design does not even exist (see Supporting Information section 2.2.1. for an example) [55, 56]. Furthermore, the number of required groups is highly sensitive to the chosen group size *k*. Although larger groups may appear advantageous from an experimental‐efficiency perspective, the mathematically optimal value of *k* is governed by the expected hit rate, defined as the probability of detecting a cooperative pair. At low hit rates (< 1%), increasing *k* reduces the number of initial screening experiments. At higher hit rates, it leads to a greater prevalence of multi‐hit pools, thereby increasing deconvolution complexity and the risk of false‐negative identification. This trade‐off is illustrated in Figure S5 for an assumed hit probability of 0.5%. In practice, the optimal group size is additionally constrained by experimental factors, most notably the background signal arising from additive effects of inactive candidates leading to cumulative luminescence quenching. Based on statistical modeling of the background quenching and the expected false‐positive rates for different *k*, we selected a group size of ten compounds, which provided a robust balance between experimental efficiency and reliable discrimination of cooperative quenching events (see Supporting Information Section 2.2.2 for details). With our set of compounds and optimal group size in hand, we designed and tested a swap algorithm for group design. After identifying a solution with 159 groups covering all possible pairs using the swap algorithm, we later found that a greedy algorithm [43] (Figure 2B) can find slightly better solutions with even lower runtimes (18.9 min vs. 0.6 min), rendering it superior in most cases(see Supporting Information section 2.3 for details).

**FIGURE 3:**
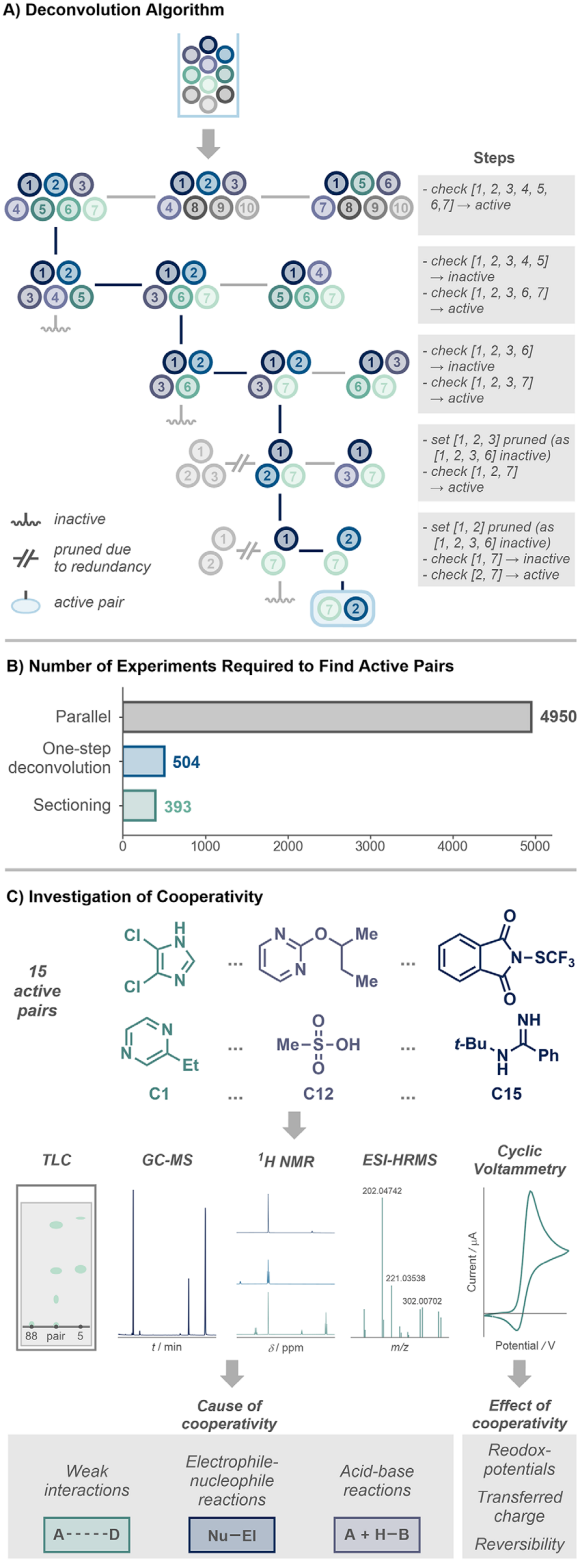
(A) Iterative deconvolution algorithm. Positive pools are recursively subdivided into smaller subsets until active pairs are uniquely identified. Redundant subsets are pruned to minimize experimental effort. (B) Comparison of total experiments required to identify all active pairs using parallel testing, one‐step deconvolution, or iterative sectioning. (C) Investigation of cooperative interactions. Active pairs were characterized using TLC, GC‐MS, ^1^H NMR, and ESI‐HRMS to determine the origin of cooperativity. Cyclic voltammetry was used to determine the effect of the cooperative interaction.

For the initial 159 convoluted groups, the luminescence quenching of each group was determined for the excited state photosensitizer **PS**. A quenching percentage of 60% or higher was selected as the threshold for defining a cooperative hit. This value was chosen based on the statistically modeled background quenching of individual substrates and expected random errors to minimize the probability of false positives (see Supporting Information section 2.2.2 for details). For 25 (16%) of the tested combinations, a strong increase in quenching, up to total fluorescence extinction, was observed. This proves that cooperative interactions between the tested substrates exist and strongly influence the properties of the tested pairs. While this result enables rapid screening for cooperativity, a hit only indicates that at least one of 45 possible binary combinations shows a cooperative interaction. Consequently, a deconvolution strategy is required to identify the active pairs and investigate both the nature of the cooperativity and their potential reactivity. We first applied a one‐step deconvolution strategy in which each quenching group of ten compounds was divided into eight subsets of seven compounds each, so that every pairwise combination of compounds displaying quenching would result in a unique pattern of subsets observed to show quenching (see Supporting Information section 2.5 for details). While this strategy enables parallel deconvolution of a set with a single round of experiments, its success is guaranteed only if the group contains only one active pair. During the investigation of the 25 active groups, we, however, observed both single quenchers and groups containing more than one active pair. Consequently, additional experiments were required to deconvolute the groups into 15 active pairs. To address these limitations and provide a reliable deconvolution strategy for scenarios where higher hit rates are expected, we developed an interactive, iterative approach capable of detecting multiple pairs, single quenchers, and even higher‐order interactions among three compounds (Figure 3A).

This algorithm is a generalization of the *interval bisectioning method*, which iteratively splits a group into equal‐sized subsets until an active element is found. Our approach is building a tree of sets with decreasing set sizes *k*_level_ on each tree level. Starting with the initial group size *k*_0_, the set size on the following levels is chosen as $k_{i}=\lceil \;\frac{2}{3}k_{i-1}\rceil \;$, stopping when a pair (i.e., *k*_i_ =  2) is reached. For our experimental design with *k*_0_ =  10, one thus obtains a tree with five levels featuring three sets of seven, five, four, three and two, which are constructed so that each possible pair from the parent set is contained at least once. After experimental evaluation of the first set's quenching, positive or negative feedback is given to the program. If the outcome is positive, the algorithm generates three sets for the next level and continues. This is repeated until the lowest level is reached, at which point a positive result indicates the identification of an active pair. If the user demands checking the possible presence of more than one active pair, the interval sectioning algorithm continues. In analogy to interval bisectioning, the number of attempts scales with the logarithm of the initial set size *k*_0_, rendering the algorithm very efficient for large set sizes (see Supporting Information section 2.7 for details). This approach allows identification of all 15 cooperative pairs in only 393 experiments, compared with 504 for one‐step deconvolution and 4,950 for full parallel screening (Figure 3B) (see Supporting Information section 2.7.7 for details).

**FIGURE 4:**
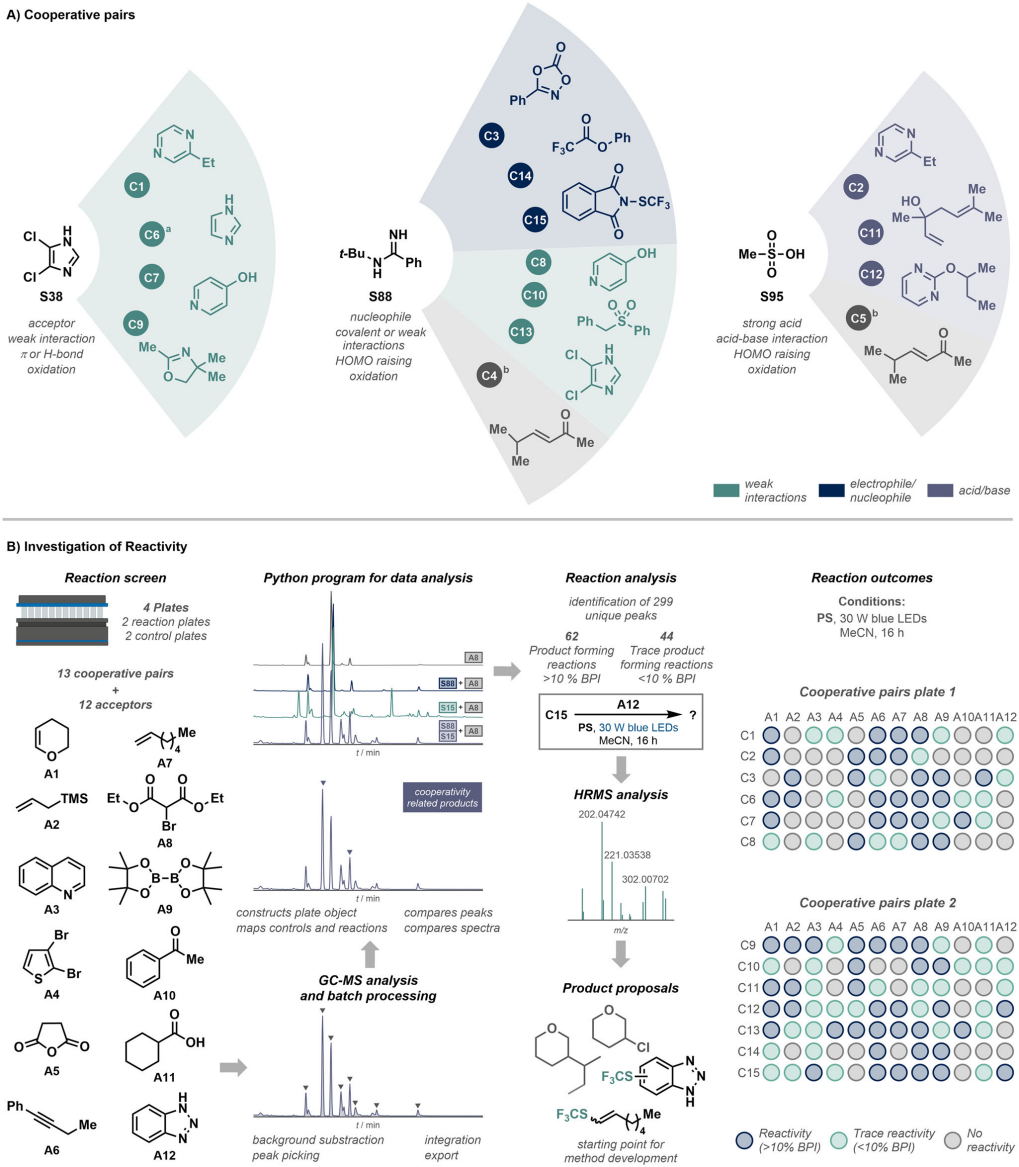
(A) Identified cooperative substrate pairs. Fifteen active pairs were discovered through algorithmic pooling and luminescence screening, grouped by mechanistic origin: weak non‐covalent donor–acceptor interactions, nucleophile–electrophile reactions, and acid–base equilibria. (B) Investigation of reactivity and reaction outcome analysis. Cooperative pairs were screened against a panel of twelve electrophilic acceptors using an automated GC‐MS workflow and a Python‐based data analysis pipeline. Automated background subtraction, peak matching, and batch processing enabled efficient identification of reactivities. ^a^The cooperative effect occurs between **S38** and imidazole (hydrolysis product of 1,1’‐carbonyldiimidazol) based on the conducted NMR studies. ^b^Additional quenching and NMR studies with different batches of the Michael acceptor suggest that an impurity is responsible for the cooperative effect. *Abbreviations*: BPI, base peak intensity; LED, light‐emitting diode.

Having successfully established our convolution/deconvolution and screening strategy to identify cooperative interactions, we aimed to further investigate the underlying cause (i.e., the type of interaction) and effect of cooperativity to enable potential applications in organic synthesis (Figure 3C). The individual non‐quenchers and interacting pairs were examined carefully using ^1^H nuclear magnetic resonance spectroscopy (NMR), high‐resolution mass spectrometry (HRMS), GC‐MS and thin‐layer chromatography (TLC). By this means, we found strong evidence that each of the observed interactions between the 15 cooperative pairs (see Supporting Information section 3 for details) follows one of three mechanisms: non‐covalent donor–acceptor interactions, acid–base equilibria, and nucleophile–electrophile reactions. Weak non‐covalent interactions and acid–base reactions were mainly studied by means of ^1^H NMR spectroscopy, with strong signal shifts near acidic or basic sites indicating protonation or deprotonation, while moderate shifts indicate weak interactions such as hydrogen bonding or π–π interactions [57]. In cases where new signals in the ^1^H‐NMR spectrum, a new peak in the GC‐MS chromatogram, and a new spot on the TLC plate were observed, we propose that a reaction product caused the quenching. The effect of the interaction, i.e., the change in the electronic structure and therefore the chemical properties, was determined by cyclic voltammetry (CV), quantitatively studying potentials, transferred charge, and the reversibility of the electron transfer (see Supporting Information section 4 for details). For ten pairs, we were able to elucidate the electronic changes induced by cooperativity, e.g., the potential and direction of electron transfer exhibited by the pair. We were excited to find that even supposedly weak non‐covalent interactions can lead to significant changes in redox potentials, thereby opening up potential electron‐transfer reaction pathways for otherwise inactive compounds. Interestingly, three substrates appear in each pair and seem to be privileged for cooperativity (Figure 4A). Unsurprisingly, methane sulfonic acid (**S95**) was found to participate in multiple acid–base interactions, thereby providing access to reducible species. By contrast, *N*‐(*tert*‐butyl)benzimidamide (**S88**) can act ambivalently as a nucleophile activating electrophilic species, while also being a suspected electron donor in weak non‐covalent interactions. 4,5‐dichloroimidazole (**S38**) was found to be a privileged structural motif involved in various non‐covalent interactions with different reagents. In conclusion, all studies conducted demonstrate a strong, cooperativity‐induced increase in activity for almost all pairs identified.

We thus sought to systematically investigate the reactivity of the pairs, focusing on evaluating the limits of the new cooperativity‐induced reactivity rather than on developing synthetically useful reactions *per se*. Since previously performed CV studies demonstrated that all observed electron‐transfer processes are irreversible, we hypothesized that pairs might undergo fast subsequent reactions after electron transfer. To confirm this, decomposition studies were conducted using stoichiometric amounts of the photosensitizer. It was shown that eight of twelve pairs studied undergo significant decomposition reactions, although the single substrates show no or significantly less decomposition under the same conditions (see section 5.1 in the Supporting Information). In order to evaluate the chemical utility of these processes, a subsequent reaction screen was designed (Figure 4B). For this purpose, 13 pairs were tested with twelve acceptors, while performing controls for all individual substances, acceptors and substance‐acceptor combinations. The acceptors were chosen to be diverse but comparatively non‐polar and small, to allow analysis of possible products by GC‐MS. Because manual inspection of the 324 chromatograms and over 2,000 peaks was impractical, a custom Python program was developed to process the GC‐MS data and automatically compare each reaction to its corresponding controls (Figure 4B). This automated analysis facilitated the identification of prominent new peaks that likely arise from reactions between the cooperative pairs and the tested acceptors.

**FIGURE 5:**
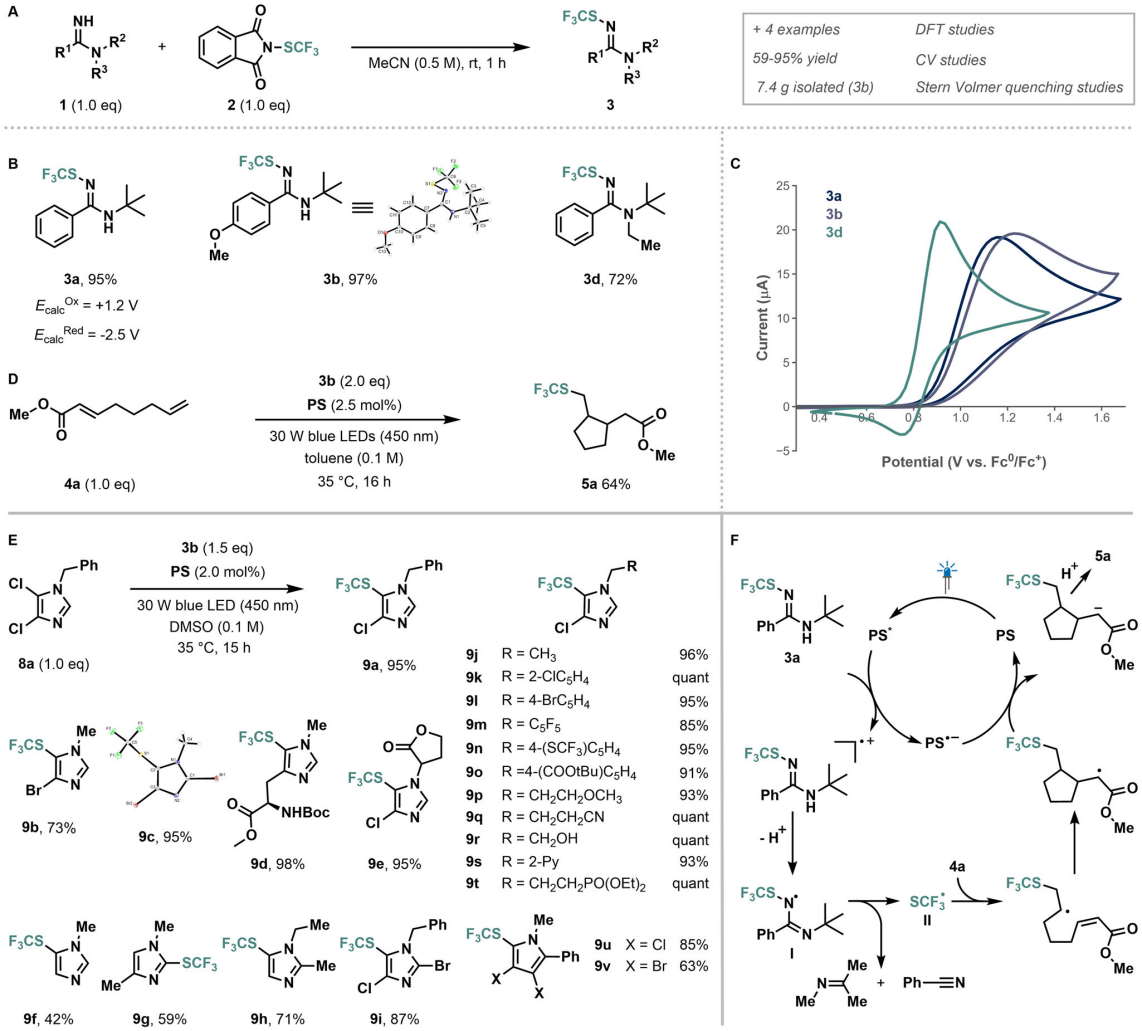
Synthesis and application of *N*‐trifluoromethylthiolated amidines as redox‐active SCF_3_‐radical precursors. (A) Synthesis of *N*‐trifluoromethylthiolated amidine reagents. (B) Synthesized *N*‐trifluoromethylthiolated amidine derivatives. (C) Voltammograms of SCF_3_‐amidines 3a, 3b and 3d at scan rates of 10 V/s. (D) Application of **3b** in the redox‐neutral, photocatalytic cyclization of methyl octa‐2,7‐dienoate (**4a**). Conditions: **4a** (0.3 mmol, 1.0 eq), **3b** (2.0 eq), [Ir(dF(CF_3_)ppy)_2_(dtbpy)][PF_6_] (PS) (2.5 mol%), toluene (0.1 M), 450 nm, 35°C, 16 h. (E) Scope of the trifluoromethylthiolation of halogenated heteroarenes. Conditions: heteroarene (0.3 mmol, 1.0 eq), **3b** (1.5 eq), **PS** (2.0 mol%), DMSO (0.1 M), 450 nm, 35°C, 15 h. (F) Proposed mechanism for the trifluoromethylthiolative cyclization of **4a**.

Considering the potential synthetic utility of the reactivities identified in the reaction screen, we found the combination of amidine **S88/1a** with *N‐*(trifluoromethylthio)phthalimide **S75/2** to be of particular interest since the strong electron‐withdrawing ability [58], lipophilicity [59], and contribution to metabolic stability and bioavailability of the trifluoromethylthiolated (SCF_3_) group makes it a valuable handle in drug design [60, 61, 62]. As our investigations regarding the cause of cooperativity for the pair suggest a nucleophile–electrophile reaction, we started our investigation by simply mixing amidine **S88** and *N*‐(trifluoromethylthio)phthalimide in acetonitrile at room temperature (Figure 5A). Pleasingly, we were able to isolate product **3a** carrying the SCF_3_‐group on the *sp* ^2^‐hybridized nitrogen atom in very high yields after one hour. Based on the reactivities observed for the cooperative pair in our reaction screen, we hypothesized that upon activation of the SCF_3_‐amidine, the SCF_3_‐radical might be formed, which can subsequently add into heterocycles or alkenes [63]. CV‐experiments (Figure 5C) revealed that reagent **3a** can indeed be oxidized by the photocatalyst [Ir(dF(CF_3_)ppy)_2_(dtbpy)][PF_6_], qualifying it as a novel, reducing SCF_3_‐reagent that might enable access to new reactivity manifolds [64, 65, 66]. To improve the applicability and reactivity of the reagent, derivatives of **3a** were synthesized and investigated by CV experiments and DFT (Figure 5B). Based on these experiments, the 4‐methoxy substituted SCF_3_‐amidine **3b**, a crystalline solid that can be synthesized on a seven‐gram scale from commercially available starting materials without the need for column chromatography, was selected to study potential trifluoromethylthiolation reactions.

Due to the ability of SCF_3_‐radicals to add into unactivated C─C‐double and triple bonds [67], we synthesized methylocta‐2,7‐dienoate **4a** as a model substrate, as it allows for a redox‐neutral addition‐cyclization sequence. Gratifyingly, the desired product was obtained in 64% yield following optimization (Figure 5D). Detailed mechanistic studies support a process initiated by the oxidation of the SCF_3_‐amidine, followed by the release of an SCF_3_‐radical **II** upon decomposition of the *N*‐amidine radical **I** (Figure 5F). The radical then adds into the terminal double bond of **4a**, followed by a cyclization, oxidation and protonation sequence leading to **5a** (see Supporting Information section 7 for details).

With the activation mode of the trifluoromethyl‐thiolation reagent established, we turned to using its redox properties for functionalizing valuable building blocks. While developing the SCF_3_‐amidines, we also examined another cooperative pair (**S38**/**S88**) and, serendipitously, observed that the addition of thiols to this system with **PS** resulted in redox‐neutral thiolation of halogenated heteroarenes via thiyl radicals [68]. However, since the thiol is used as a radical precursor, a direct trifluoromethylthiolation would require a suitable SCF_3_‐radical source. Our SCF_3_‐amidine platform enabled the trifluoromethyl‐thiolation of 1‐benzyl‐4,5‐dichloro‐imidazole **8a** with **3b** in the presence of **PS** in excellent yields (Figure 5E). Notably, there is no requirement to pre‐form the reagent with in situ‐generation of **3b** directly from **1b** and **2,** giving product **9a** in comparable yields (Table S64). The reaction shows perfect regioselectivity (**9a**‐**c**, **i**) and is broadly applicable to imidazoles bearing various functional groups (**9d**‐**t**, while also tolerating pyrroles as substrates (**9u, v**).

## 3 Conclusion

This work presents a powerful, generalizable strategy for exploring combinatorial chemical space by integrating statistical group testing with mechanism‐based luminescence screening. Our approach reduces the number of potential experiments from thousands to a few hundred while reliably identifying rare cooperative molecular interactions. Applied to luminescence quenching, the method uncovered multiple cooperative pairs among otherwise inactive substrates, arising from non‐covalent donor–acceptor, acid–base, and nucleophile–electrophile interactions. These interactions were shown to significantly alter redox properties, enabling previously inaccessible reactivity. From these findings, we developed *N*‐trifluoromethylthiolated amidines as novel, redox‐active SCF_3_‐radical precursors, achieving efficient and regioselective trifluoromethylthiolation under mild photoredox conditions. Although the present study focuses on pairwise interactions, both the greedy group‐design and the iterative deconvolution algorithm support designs that cover triplets and require multiple coverages per combination. We therefore anticipate that this framework will enable efficient experimental exploration of cooperative phenomena beyond pairwise testing. More generally, the framework is compatible with physicochemical prioritization strategies, which could be used in future work to bias the candidate space toward likely cooperative interactions while retaining the robustness of experimental group testing. Overall, this study demonstrates how optimized pooling and iterative deconvolution can transform the search for new reactivity. By linking algorithmic experiment design with mechanistically informed screening insight, it provides a broadly applicable framework for accelerating discovery in catalysis, synthesis, and beyond.

## Conflicts of Interest

The authors declare no conflict of interest.

## Supporting information




**Supporting File 1**: The authors have cited additional references within the Supporting Information [69, 70, 71, 72, 73, 74, 75, 76, 77, 78, 79, 80, 81, 82, 83, 84, 85, 86, 87, 88, 89, 90, 91, 92, 93, 94, 95, 96, 97, 98, 99, 100, 101, 102].

## Data Availability

Detailed descriptions of all performed experiments, as well as computational details, are given in the Supplementary Information. Crystallographic data for the structures reported in this article have been deposited at the Cambridge Crystallographic Data Centre under deposition numbers CCDC 2049022 (3b) and 2049023 (9c). Copies of the data can be obtained free of charge at https://www.ccdc.cam.ac.uk/structures/. All other data supporting the findings of this study are available within the article and its Supplementary Information, or from the corresponding author upon request. The source code of the algorithms developed and used in this work is available at https://zivgitlab.uni‐muenster.de/ag‐glorius/published‐paper/coop_screen. A Python implementation extending the greedy group design algorithm to triples and higher coverages is available at https://github.com/FelixKatz77/GroupDesign.
