# Supplementary material for: A General Group Testing Strategy for Discovering Chemical Cooperativity
Source: Angew Chem Int Ed Engl. 2026 Feb 3;65(11):e25278. doi: 10.1002/anie.202525278 (PMC12970518; doi:10.1002/anie.202525278)
Supplement: Supplementary file 1 — Supporting File 1: The authors have cited additional references within the Supporting Information [69, 70, 71, 72, 73, 74, 75, 76, 77, 78, 79, 80, 81, 82, 83, 84, 85, 86, 87, 88, 89, 90, 91, 92, 93, 94, 95, 96, 97, 98, 99, 100, 101, 102]. [file ANIE-65-e25278-s001.pdf]

Supplementary Information for

A General Group Testing Strategy for Discovering Chemical Cooperativity

Philipp M. Pflüger[1], Felix Katzenburg1, Frederik Sandfort[1], Michael Teders[1], Adrián Gómez-Suárez[1], Eric A. Standley[1], Matthew N. Hopkinson[1], Constantin G. Daniliuc[1], Andreas Heuer[2*] Frank Glorius[1*]

[1] Organisch-Chemisches Institut, Universität Münster, Corrensstraße 40, 48149 Münster (Germany).

[2] Institut für Physikalische Chemie, Universität Münster, Münster, Corrensstraße 28/30, 48149 Münster (Germany).

*Correspondence to: glorius@uni-muenster.de or andheuer@uni-muenster.de

Table of Contents

1. Materials and Methods 4
1.1 Experimental Methods 4
1.2 Computational Methods 7
2. Cooperativity Screening 8
2.1 Identification of Non-Quenching Compounds 8
2.2 Background for the Generation of Screening-Sets 10
2.3 Software for the Construction of Screening Sets 17
2.4 Screening of 159 Sets of 10 Compounds each 24
2.5 Initial One-Step Deconvolution of Screening sets 30
2.6 Statistical Evaluation of the Convoluted/Deconvolution Approach 38
2.7 Interval Sectioning Algorithm for Pair Finding 41
2.8 Calculation and Comparison of Hit Rates 66
3. Origin of the Cooperative Luminescence Quenching 69
3.1 Theoretical Background to Investigate the Origin of Cooperativity 69
3.2 Gas Chromatography–Mass Spectrometry Studies 71
3.3 Thin Layer Chromatography Studies 88
3.4 Nuclear Magnetic Resonance Studies 93
3.5 Electrospray Ionization High Resolution Mass Spectrometry 105
3.6 Conclusion of Origin Studies 115
4. Investigation of the Change in Activity and Reactivity 118
4.1 Theoretical Procedure to Investigate the Fluorescence Quenching 118
4.2 Results of Multi-Catalyst Quenching Studies 119
4.3 Background for the usage of Cyclic Voltammetry Studies 121
4.4 General Procedure for Cyclic Voltammetry Studies 121
4.5 Investigation on Redox Potentials and Transferred Charges 122
4.6 Investigation on Redox Reversibility and Possible Reactivity 147
4.7 Conclusion of Studies Investigating the Change in Activity and Reactivity 148
5.1 Decomposition Screen 151
5.2 Reaction Screening 156
6. SCF_3-Amidines 189
6.1 Preparation of Starting Materials 189
6.2 Preparation of SCF_3-Amidines 196
6.3 Mechanistic Studies on SCF_3-Amidines 202
7. Redox-Neutral Radical Cyclization as Model Reaction 207

7.1 Preparation of Starting Materials 207
7.2 Optimization of the Model Reaction 209
7.3 Scope of the Redox-neutral Radical Cyclization 214
7.4 Mechanistic Investigations on the Redox-neutral Radical Cyclization 216
8. Trifluoromethylthiolation of Heteroarenes 220
8.1 Preparation of Starting Materials 220
8.2. Reaction Optimization 227
8.3 Scope of the Trifluoromethylthiolation of Heteroarenes 231
8.4 Mechanistic Studies on SCF_3-Amidines 243
9. Geometries Optimized Structures 248
10. X-ray Crystal Structure Analysis 281
11. NMR Spectra 284
12. References 337

1. Materials and Methods

1.1 Experimental Methods

Unless otherwise noted, all reactions were carried out under an atmosphere of argon in oven-dried glassware. Anhydrous solvents were either purchased and stored over molecular sieves under argon or purified by distillation over standard drying agents. Reaction temperatures are reported as the temperature of the medium surrounding the vessel. Commercially available chemicals were used as received if not stated otherwise.

Photochemical reactions were performed in a *Hepatochem* EvoluChem^{TM} PhotoRedOx Box Duo device (Figure S1) and irradiated with two EvoluChem^{TM} P303-30-1 LEDs (30 W, λ_{max} = 450 nm, Figure S2). The reaction temperature was measured to be between 30 °C and 35 °C using this setup.

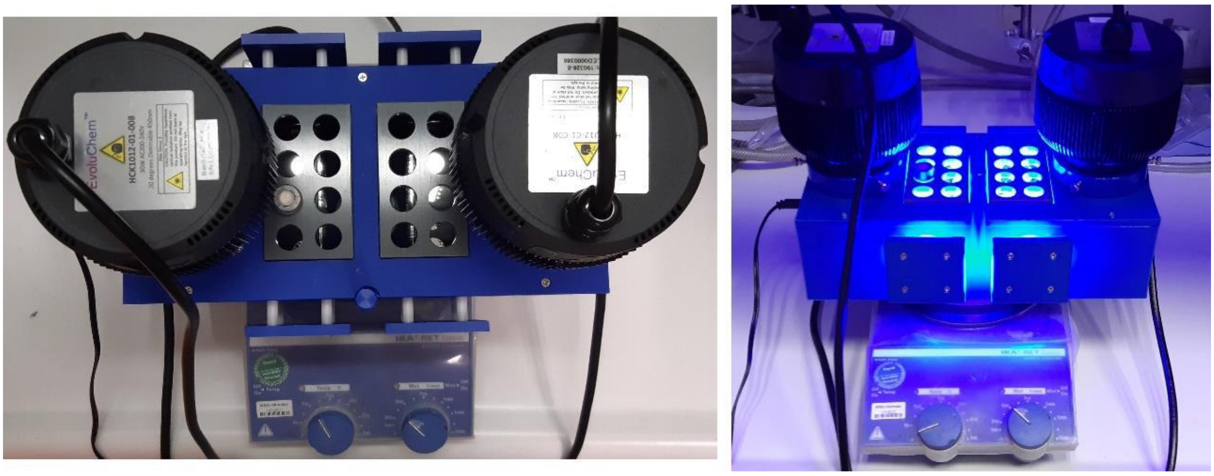

Figure S1: *Hepatochem* EvoluChem^{TM} PhotoRedOx Box Duo device.

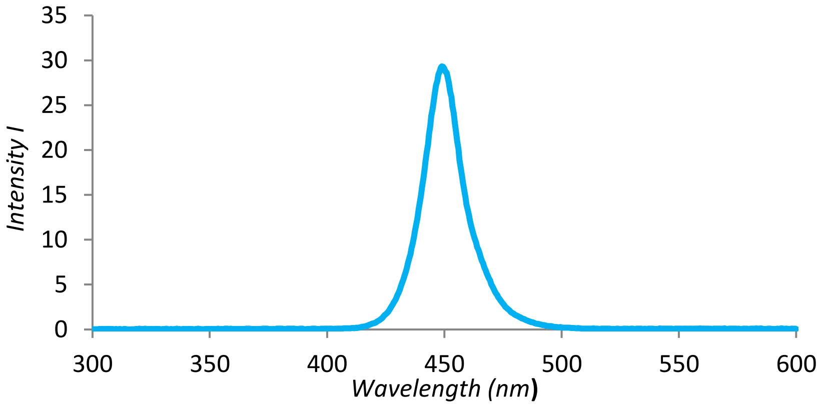

Figure S2: Emission spectra of the EvoluChem^{TM} P303-30-1 LEDs (30 W, λ_{max} = 450 nm) used for reaction irradiation.

Analytical thin-layer chromatography (TLC) was performed on silica gel 60 F254 aluminium plates (*Merck*). TLC plates were visualized by exposure to ultraviolet light (254 nm, 366 nm) and were dipped into a solution of $KMnO_4$. Flash column chromatography was performed on *Acros Organics* silica gel (35-70 mesh) under positive pressure of argon elution with the specified solvent system.

NMR spectra were recorded on a *Bruker* Avance II 300, *Bruker* Avance II 400, *Agilent* DD2 500 or on an *Agilent* DD2 600 spectrometer. Chemical shifts (δ) are quoted in ppm downfield of tetramethylsilane. The residual solvent signals were used as references for ^{1}H and ^{13}C NMR spectra ($CDCl_3$: δH = 7.26 ppm, δC = 77.16 ppm). ^{19}F NMR spectra are not calibrated by an internal reference. The multiplicity of all signals was described with standard abbreviations. Coupling constants (*J*) are quoted in Hz.

Besides reactions conducted in decomposition (5.1) and reaction screening experiments (5.2), GC-MS spectra were recorded on an *Agilent* Technologies 7890A GC-system with an *Agilent* 5975C VL MSD or an *Agilent* 5975 inert Mass Selective Detector (EI) and a HP-5MS column (0.25 mm x 30 m, film: 0.25 µm). The major signals are quoted in *m*/*z* with the relative intensity in parentheses. The method indicated as '50_40' starts with the injection temperature T_0 (50 °C); after holding this temperature for 3 min, the column is heated by 40 °C/min to temperature T_1 (290 °C or 320 °C) and this temperature is held for an additional time *t*. GC-FID analysis was undertaken on an *Agilent* Technologies 6890A equipped with an HP-5 quartz column (0.32 mm x 30 m, film: 0.25 µm) using flame ionization detection. Method: Initial temperature 50 °C, hold 3 min, increment 40 °C/min, final temperature 280 °C, hold 3 min. High-resolution mass spectra (HRMS) were obtained by the MS service of the Organisch-Chemisches Institut, Universität Münster, using electrospray ionisation (ESI) on a *Bruker* Daltonics MicroTof spectrometer and on a *Thermo Fischer Scientific* Orbitrap LTQ XL.

The luminescence quenching was carried out using a *Jasco* FP-8300 spectrofluorometer equipped with a Xenon light source. The following parameters were employed: excitation wavelength = 420 nm, emission wavelength recorded = 440 nm to 700 nm, excitation bandwidth = 5 nm, emission bandwidth = 5 nm, data interval = 0.1 nm, scan speed = 1000 nm/min, response time = 0.2 sec. The following parameters were employed throughout Stern-Volmer luminescence quenching studies: Excitation bandwidth = 5 nm, data interval = 0.2 nm, scan speed = 500 nm/min, response time = 0.2 sec. The samples were measured in Hellma fluorescence QS quartz cuvettes (chamber volume = 1.1 mL, H × W × D = 46 mm × 12.5 mm, 12.5 mm) fitted with a PTFE stopper. All samples used in the luminescence

quenching-based screening studies were prepared under oxygen-free conditions. The photocatalysts and potential quenchers were weighed into vials and placed inside an argon box under a positive pressure of argon. Acetonitrile was degassed by argon sparging for at least one hour and also placed inside along with micropipettes and their tips, cuvettes, empty vials and waste containers. The argon box was then left to purge for one hour under a positive argon pressure. Each photocatalyst and substrate sample was then dissolved in acetonitrile. For each measurement, the appropriate amounts of the photocatalyst and substrate were added to a cuvette and diluted to 1 mL with acetonitrile using micropipettes. For the screening studies, the cuvette was then capped with a PTFE stopper before being removed from the argon box and transferred to the fluorescence spectrometer. After the measurements, the sealed cuvette was brought back into the argon box, emptied, cleaned with acetonitrile before preparation of the next sample.

Cyclic voltammetry experiments in 6.3.2 were conducted in a *TSC 1600* closed measuring cell that was connected to a *Metrohm Autolab PGSTAT204* potentiostat. The cell consists of the front-end of a platinum wire as the working electrode, a platinum crucible counter electrode and a silver wire pseudo-reference electrode. The examined substances (0.02 mmol) were measured in a solution of tetrabutylammonium hexafluorophosphate in acetonitrile (2.0 mL, 0.1 M). The applied voltage was referenced to the redox potential of the ferrocene/ferrocenium ion pair. A scan rate of 0.1 to 10.0 V/s was applied.

Cyclic voltammetry experiments in 4.1 were conducted using a *CH Instruments CHI600E* potentiostat. The cell consists of the front-end of a platinum wire as the working electrode, a platinum crucible counter electrode and a silver/silver chloride reference electrode (Ag/AgCl, NaCl 3M). The examined substances (0.01 mmol) or substance pairs (0.01 mmol, each) were measured in a solution of tetrabutylammonium hexafluorophosphate in acetonitrile (2.0 mL, 0.1 M).

X-Ray diffraction: Data sets for compound **9c** were collected with a *Bruker* D8 Venture CMOS diffractometer. For compound **3b,** data sets were collected with a *Bruker* APEX II CCD diffractometer. The following programs were used: data collection: APEX3 V2016.1-0 (*Bruker* AXS Inc., **2016**)[1]; cell refinement: SAINT V8.37A (*Bruker* AXS Inc., **2015**)[2]; data reduction: SAINT V8.37A (*Bruker* AXS Inc., **2015**)[2]; absorption correction, SADABS V2014/7 (*Bruker* AXS Inc., **2014**); structure solution *SHELXT-2015*[42], structure refinement *SHELXL-2015*[4,5] and graphics, *XP*[6] (Version 5.1, *Bruker* AXS Inc., Madison, Wisconsin, USA, **1998**). *R*-values are given for observed reflections, and wR^2 values are given for all reflections.

1.2 Computational Methods

All computations were carried out using the ORCA 4.1.1 software package[7]. The geometries were optimized with the CAM-B3LYP range-separated hybrid functional[8] on a recontracted Ahlrichs double-ζ-basis (def2-SVP)[9], applying the CPCM continuum solvation model for acetonitrile or DMSO[10]. For calculations regarding the mechanistic studies in chapter seven, a Grimme D3 atom-pairwise dispersion correction[11] was applied. Optimized geometries were characterized to be minima or transition states on the respective potential energy surface by harmonic frequency analysis on the same level of theory. For further energy evaluation single point calculations were performed on the optimized geometries using the CAM-B3LYP range-separated hybrid functional on a recontracted triple-ζ-basis set (def2-TZVPP) with the CPCM for acetonitrile or DMSO. No symmetry or internal coordinate constraints were applied. Optimized structures are given in the .xyz format and were visualized using Jmol[12]. Redox potentials were determined by adapting a protocol reported by Nicewicz and coworkers[13] using the CAM-B3LYP range-separated hybrid functional on a triple-ζ-basis set (def2-TZVPP) with the CPCM model for acetonitrile.

The energy of different conformers and rotamers was evaluated and the energetically most stabilized conformer was used in following calculations. Gibbs free energies at 298 K (G_{298}) were obtained as the sum of the electronic energies (SCF energies) and the corresponding free energy corrections (ZPVE, thermal corrections, enthalpy correction, entropic corrections), as obtained from the frequency calculation.

Redox potentials were then calculated from the Gibbs free energies of the respective oxidized and reduced species; the values were referenced to saturated calomel electrode (SCE) by subtraction of its absolute potential in acetonitrile (E_{ref} = 4.42 V)[14].

$$E^{calc} = \frac{G_{298}(red) - G_{298}(ox)}{F} - E_{ref}$$

2. Cooperativity Screening

2.1 Identification of Non-Quenching Compounds

Table S1: 100 Identified, non-quenching compounds showing no significant quenching (<10%) of the excited state photocatalyst [Ir(dF(CF_3)ppy)$_2$(dtbpy)][PF_6] (**PC1**).

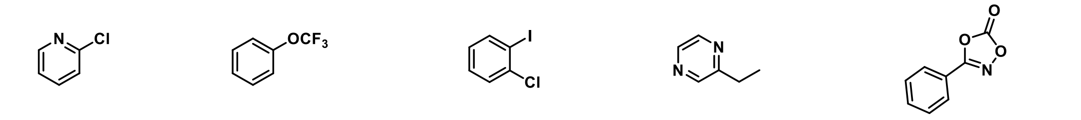

S1 S2 S3 S4 S5

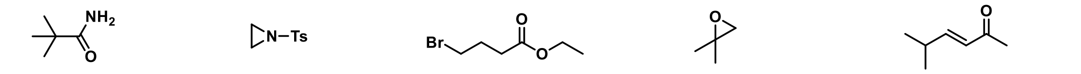

S6 S7 S8 S9 S10

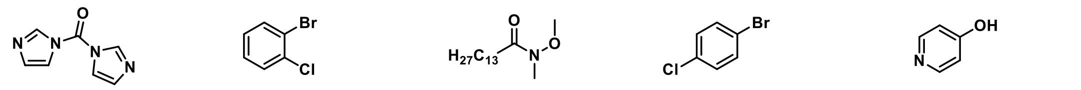

S11 S12 S13 S14 S15

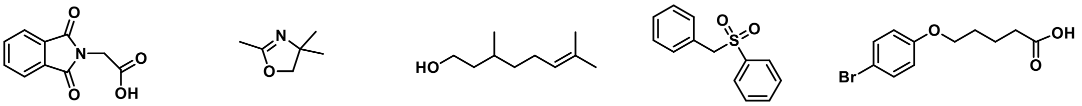

S16 S17 S18* S19* S20

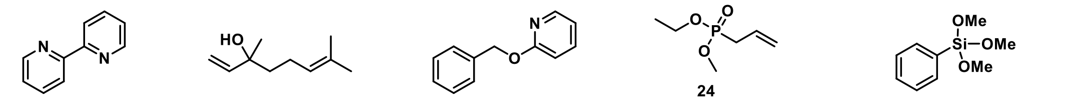

S21 S22 S23 S24 S25

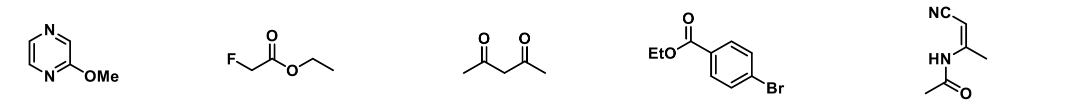

S26 S27 S28 S29 S30*

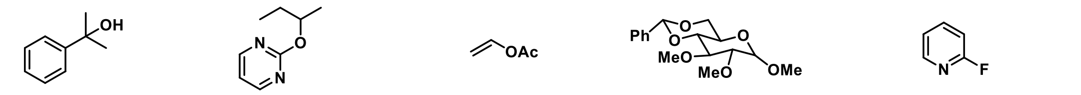

S31 S32* S33 S34* S35

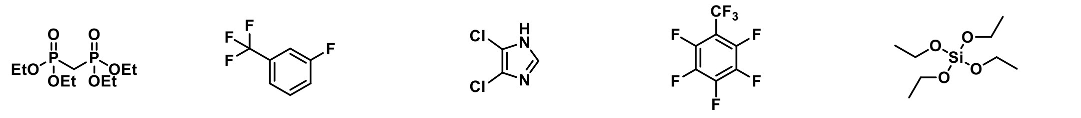

S36 S37 S38 S39 S40

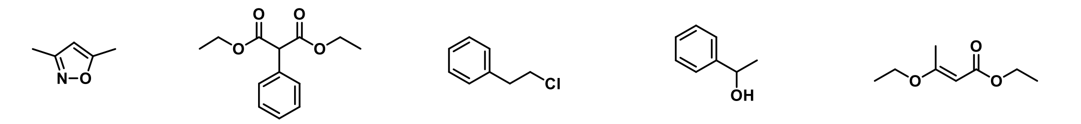

S41 S42 S43 S44 S45

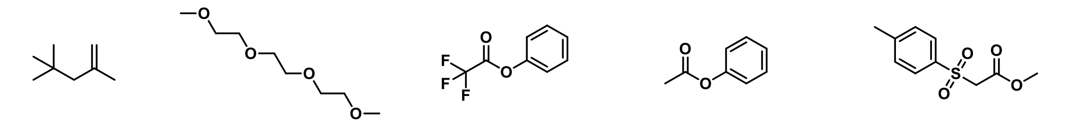

S46 S47 S48 S49 S50*

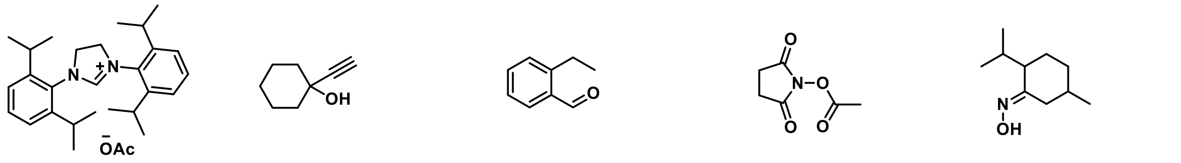

S51* S52 S53 S54 S55

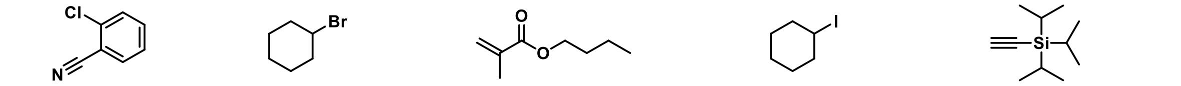

S56 S57 S58 S59 S60

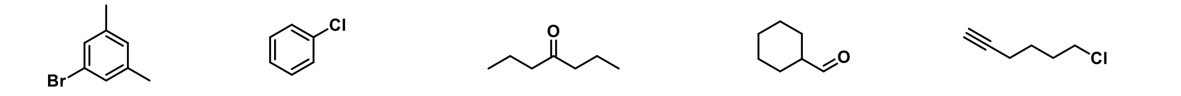

S61 S62 S63 S64 S65

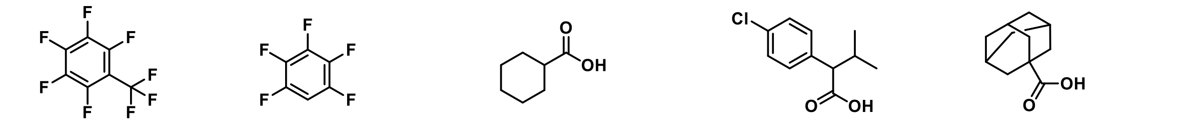

S66 S67 S68* S69 S70

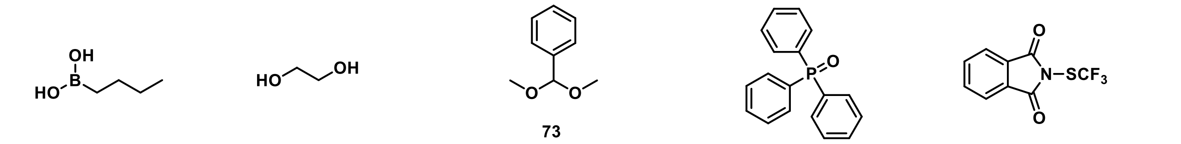

S71 S72 S73 S74 S75

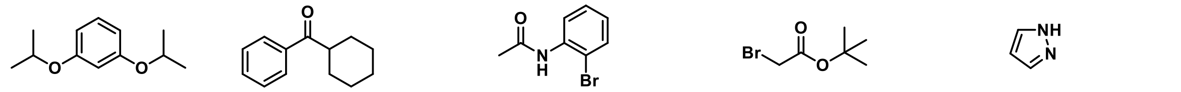

S76 S77 S78 S79 S80

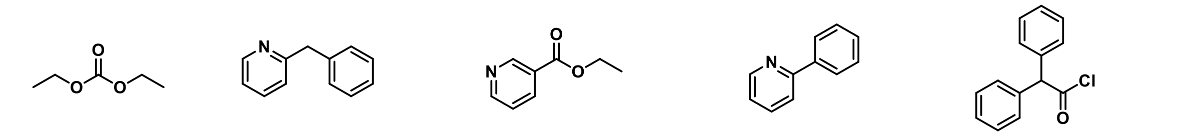

S81* S82 S83 S84 S85

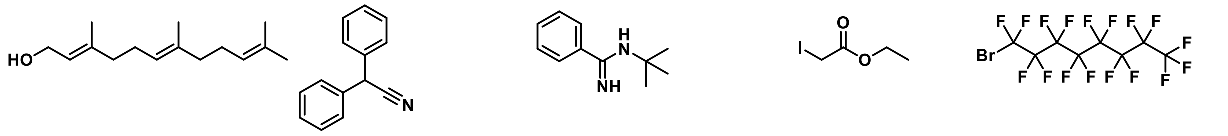

S86* S87 S88* S89 S90

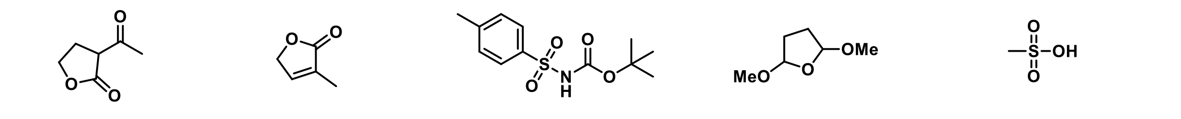

S91* S92 S93 S94 S95

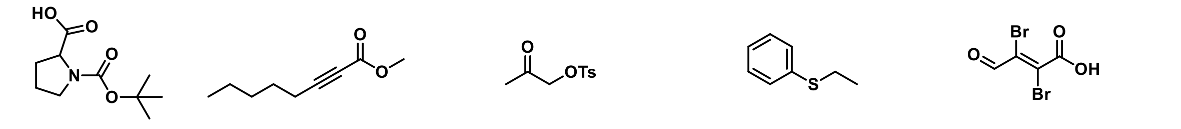

S96* S97* S98* S99* S100

*Quenching between 5% and 10%

For the selection of 100 non-quenching compounds, substrates were tested individually using [Ir(dF(CF$_3$)ppy)$_2$(dtbpy)][PF$_6$] (**PC1**) as photocatalyst. Stock solutions of the photocatalyst (0.1 mM) and the substrates (250 mM) were prepared in degassed MeCN. To ensure good reproducibility and low errors during the quenching studies, the fluorescence of [Ir(dF(CF$_3$)ppy)$_2$(dtbpy)][PF$_6$] (**PC1**) was probed multiple times until reproducible intensities could be achieved. In doing so, 100 μL of the catalyst stock solution was pipetted into a cuvette and diluted with 1000 μL of MeCN. To identify potential non-quenchers, 100 μL of the catalyst stock solution and 100 μL of the substrate stock solution were pipetted into the cuvette and diluted with 900 μL of MeCN, resulting in a photocatalyst:potential quencher ratio of 1:2500. The fluorescence of the catalyst-substrate mixture was measured, and a substrate was identified as "non-quencher" if the fluorescence-intensity at 504 nm was not reduced by more than 10%. For later identification and deconvolution, the non-quenchers were further divided into two classes: substances with no quenching (up to 5%) and substances exhibiting minor quenching (5% to 10%) (Table S1).

2.2 Background for the Generation of Screening-Sets

2.2.1 Theoretical Background for the Generation of Screening-Sets

This screening uses a convolution approach to search for dual combinations of individually non-quenching substances (number of non-quenching substrates = n), which together quench the photocatalyst through different effects. If these dual pairs were tested individually, $\binom{n}{2}$ experiments would have to be performed. For 100 substrates, as used in this screen, 4950 experiments would be required. Such a screening approach is not only time-consuming but requires higher quantities of both photocatalyst and substrates while the number of required fluorescence quenching experiments would increase approximately by half of the square for higher numbers of substrates, our approach is based on a convolution, where more than 2 substrates are combined to sets. In each set k compounds are combined, resulting in $\binom{k}{2}$ dual combinations for each set, and each dual pair must in total occur at least once.

In literature, this problem is known as (n,k,t)-block designs (please note that blocks are denoted as sets in the following) and is well-known from different subjects. Starting from n numbers (compounds in our case), one aims to identify a number of blocks (i.e. set or experiments), each containing k numbers, such that any t-element subset (t = 2: pairs, t = 3; triplets) occurs at least once. We mainly concentrate on t = 2 but also t = 1 and t = 3 will become relevant in the

discussion. The number of blocks for a chosen design is denoted b. Here are three examples: (1) A (7,3,2) block design is given by (1,2,3), (1,4,5), (1,6,7), (2,4,6), (2,5,7), (3,4,7), (3,5,6), yielding $b = 7$. (2) A (4,3,2) block design is given by (1,2,3), (1,2,4), (2,3,4), corresponding to $b = 3$. (3) A (4,2,2) block design is given by (1,2), (1,3), (1,4), (2,3), (2,4), (3,4), corresponding to $b = 6$.

Here, we briefly discuss two types of optimality. First, one may ask about the minimum value of b for a (n,k,t)-block design. For all examples given above, there is no solution with a smaller number of blocks. However, for general triplets a (n,k,t) with larger values of n and k, optimum solutions for the block design are generally not known. Rather, one may use specialized, partly heuristic, algorithms to find solutions with a possibly small number of blocks. For $n < 100$, several solutions (up to $t = 7$) can be found on the LA JOLLA COVERING REPOSITORY TABLES.[17] There is also a second type of optimality. Just from counting the number of, e.g., pairs per block, it is easy to see that the theoretical minimum of b is given by $b_{min} = \frac{\binom{n}{t}}{\binom{k}{t}}$ which for the case $t = 2$ can be rewritten as $\frac{(n-1)n}{(k-1)k}$. If b_{min} is an integer value and if there exists a block design with $b = b_{min}$, this block design is denoted a Steiner system. Naturally, for a Steiner system, each pair is found exactly once. For the three examples given above, (1) and (3) are Steiner systems, whereas (2) is not a Steiner system.[15] Indeed, for (2) one obtains an integer value $b_{min} = 2$. However, it is easy to check that no block design with $b = 2$ is possible.

2.2.2 Estimation of Applicable Thresholds

Although the reduction of the fluorescence of a photocatalyst is suitable for determining whether individual substances interact with it, the quantitative observation can be subject to errors. Due to the chosen setup (argon box with fluctuating oxygen content, slight quenching of some individual substrates, absorption of the substrates at the excitation wavelength, ...), several errors must be considered. A key issue that makes a statistical analysis of the error necessary is the slightly fluctuating oxygen content in the used argon box, which results from the opening and closing of the box. Since the fluorescence of the used photocatalyst (**PC1**) strongly depends on the given oxygen concentration, this pseudo-random error has to be taken into account. To minimize systematic errors, the baseline fluorescence of **PC1** was measured until the observed emissions fluctuated around a constant mean value. In general, to determine whether cooperative interactions are present, the quenching of the individual substrates could be subtracted from the measured quenching of the substrate combinations. However, since the

magnitude of this error appears to be only slightly smaller than the true quenching of the individual substrates, such a quantitative determination of the cooperative interaction cannot be carried out. Since only strong cooperative interactions are of interest in the context of this work, as they induce strong changes in physicochemical properties and significant changes in reactivity (4.1 to 5.2), a qualitative approach seems to be sufficient to find chemically relevant interactions. To achieve this, thresholds, above which quenching can only be explained by cooperative interactions with an excellent degree of certainty, must be defined depending on the set size.

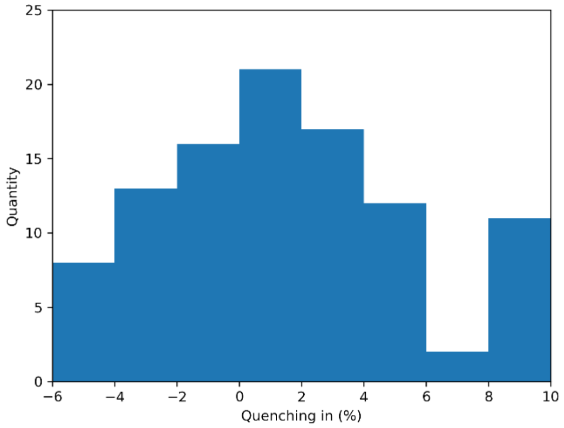

Figure S3: Quantity of measured quenching percentages among the screened individual compounds in given ranges.

To determine these thresholds, the relative quenching of all substrates was measured as specified in chapter 2.1. The resulting distribution (Figure S3) clearly shows the effect of a significant random error, since rises in the measured fluorescence, thereby negative quenching percentages are observed in about one third of all cases. Nonetheless, the distribution clearly tends towards values > 0% meaning that statistically, slight quenching can be observed in the majority of cases. Since the influences of those errors are overlapping, a clear distinction and separate calculation can not be made. In order to make a meaningful assessment of the quenching to be expected from sets with multiple substrates (k), the mean (μ = 1.46%) and the standard deviation (σ = 4.00%) of the found distribution were determined (Figure S4).

Since, for higher numbers of k, the quenching percentage is expected to be normally distributed, the determined statistical characteristics were used to model this distribution for different set sizes k (Figure S4, left). This approximation can be considered particularly valid (Figure S4, right) for the ensemble of all tested 159 sets, since within this experiment, individual compounds will appear similarly often. However, it has to be mentioned that the part of the

standard deviation caused by randomly fluctuating oxygen is not additive for $k > 1$, while in this setting also other systematic errors can occur due to non-quenching and thus irrelevant cooperative effects (e.g. scattering due to reduced solubilities as well as absorption due to colour changes).

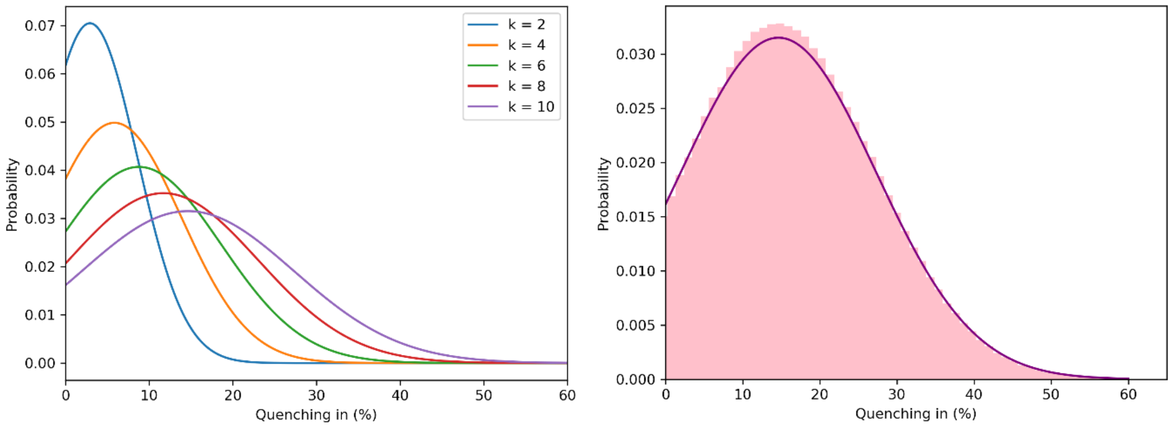

Figure S4. Modelled normal distributions for different set sizes *k* based on the statistical characteristics of the determined substrate quenching distribution (left). Normal distribution of $k = 10$ (purple line) and a distribution as generated numerically from randomly sampling values out of the measured quenching of individual compounds (right).

Table S2: Borders of the 90.0, 95.0, 99.0, 99.5 and 99.9%-quantiles for different set sizes *k*.

Number of Sub-strates per Set	q-Quantile Borders				
k	**90.0%**	**95.0%**	**99.0%**	**99.5%**	**99.9%**
2	10.2%	12.2%	16.1%	17.5%	20.5%
3	13.3%	15.8%	20.5%	22.2%	25.9%
4	16.1%	19.0%	24.5%	26.5%	30.7%
5	18.8%	22.0%	28.1%	30.4%	35.1%
6	21.3%	24.9%	31.6%	34.0%	39.2%
7	23.8%	27.7%	34.9%	37.5%	43.1%
8	26.2%	30.3%	38.0%	40.9%	46.8%
9	28.6%	32.9%	41.1%	44.1%	50.4%
10	30.9%	35.5%	44.1%	47.2%	53.9%
12	35.3%	40.4%	49.8%	53.3%	60.5%
14	39.7%	45.1%	55.3%	59.1%	66.9%
16	43.9%	49.8%	60.7%	64.7%	73.0%
18	48.1%	54.3%	65.8%	70.1%	79.0%
20	52.2%	58.7%	70.9%	75.4%	84.8%

For the distribution functions determined in this way, the probability of quenching above a certain threshold can be estimated using the corresponding quantiles. To ensure that no sets showing solely additive effects are categorized as cooperative quenching, the values of the 99.9% quantiles (Table S2) are used to determine the quenching thresholds in all conducted studies. For the sake of convenience and to ensure that unexpected positive systematic errors are taken into account, sets need to exhibit significantly higher quenching (~5%) to be considered as clear hits. In case that any set exhibits quenching in the range of ~±5% to the 99.9% quantile it is considered as borderline hit and in the case of deconvolution it needs further investigation. For the sake of convenience, all figures are rounded to the nearest integer fully divisible by five. The values estimated in this way reflect the empirical values applied within the scope of this work and have been proven to be practically applicable and robust.

Table S3: Applied thresholds (upper and lower limit) depending on set size n based on 99.9% quantiles and empirical data

n	1	2	3	4	5	6	7	8	9	10
Threshold (%) (upper and lower limit)	10<br>20	15<br>25	20<br>30	25<br>35	30<br>40	35<br>45	40<br>50	40<br>50	45<br>55	50<br>60

Based on these thresholds, reasonable limits can be defined for a reasonable maximum set size. Thereby and limited by the underlaying luminescence reduction of all individual compounds even set sizes of $n > 10$, up to $n = 20$ seem to be reasonable. But since for these large set sizes, multiple weak and therefore irrelevant cooperative effects could reduce the efficiency of a convoluted approach and would lead to multiple false positive results. In addition, multiple dual or even triplet cooperative effects could occur in one set. Although the developed interval sectioning algorithm is able to clarify these hits, it would significantly reduce its efficiency. For $n = 10$, the occurrence of multiple pairs or triple cooperative effects is comparably low, thereby allowing the deconvolution or interval sectioning to be kept efficient. In addition, a threshold between 50% and 60% quenching (2.5) can be set, providing leeway (60% to 100% quenching) for cooperative effects and allowing for the distinction between strong and weak cooperative interactions. Other practical reasons for the decision on $k = 10$, which become relevant in the used setup, are the solubility of compounds in regard to the pipette volume (needed concentration could not be reached in any case), limited space for samples in the used box and a higher probability for occurring errors with higher set sizes. For this set size, quenching above

60% means that a strong indication for a cooperative interaction has been found, while a quenching between 50 and 60% means that a weak indication for cooperativity or indication for weak cooperativity has been found (2.5). In those cases, the probability for a later unsuccessful deconvolution is considered to be comparably high. For the block-wise one-step deconvolution, the upper threshold (50%) was used again, enforcing the finding of strong interactions. For the iterative deconvolution, the upper limit was used as threshold (Table S3).

To enable lower and more accurate thresholds as well as larger set sizes in similar future studies, some modifications can be made to the screening. To have lower standard deviations, photocatalysts or other quenchable luminescence exhibitors should be chosen less oxygen sensitive, or alternatively, a measuring system, including a spectrometer, should be handled under a static inert atmosphere, as in a classical glovebox. In addition, and for being able to also detect less intensive cooperative effects, individual substrates should be chosen to reduce the luminescence of the quenchable complex as little as possible, in an ideal case, not at all. It should also be avoided to use overall poorly soluble or visible light-absorbing substrates to avoid multiple systematic errors.

2.2.3 Practical Decisions for the Generation of Screening-Sets

A major goal in the design of a convolutional screening approach is to reduce the number of experiments while maintaining a high accuracy. Regarding this accuracy, it is of practical importance to minimize the false positive rate to zero by adequate controls and experimental design, while a high false negative rate represents overlooking of hits and thus a reduction of efficiency. Taken together, this means that high hit rates (i.e., minimizing experimental effort while maximizing detection) must be achieved.

In the case of the convolutional/deconvolutional screening for cooperativity presented here, this means that the total number of experiments for convolution/deconvolution, as well as controls, must be minimized. This number of experiments to be conducted statistically is thereby determined by the total number of substrates to be tested (n), by the set size (k), by the expected hit probability (h), and by the algorithms used to generate the corresponding sets (detailed mathematical considerations of this problem can be found in Section 2.7.2). Since the expected hit probability for a screen searching for strong cooperative interactions is particularly low (<1.0%), comparatively large set sizes are reasonable. For a screen like the one performed in this work, two limits apply. The first one is given by the expected hit probability. If the hit probability is too high, the number of experiments needed to find the active pair (deconvolution)

exceeds the number of experiments needed for the screen, even in cases of small set sizes, by far, and thus renders the approach inefficient. In other words, this means that a convolutional approach becomes particularly efficient whenever the hit probability is particularly low. The second limit derives from the experimental error and possible background quenching, i.e., activity (in cases of potential other convolutional screens). For cases in which the error is not cumulative (random errors), because no background quenching (activity) can be observed, no limitation is given here. Since slight background quenching was observed for many substrates in the screening performed in this work, making the statistical experiment not negligible, for values k > 10, the problem arises that cooperative quenching can no longer be detected unequivocally (2.2.2.). Taken together, these two characteristics or limits allow the assumption that a set size of $k = 10$ is particularly efficient (Figure S5). Even though larger set sizes would theoretically allow the approach to be slightly more efficient, not statistically distributed cooperative pairs or other experimental issues, as encountered within this screen, make smaller set sizes preferable (see below)

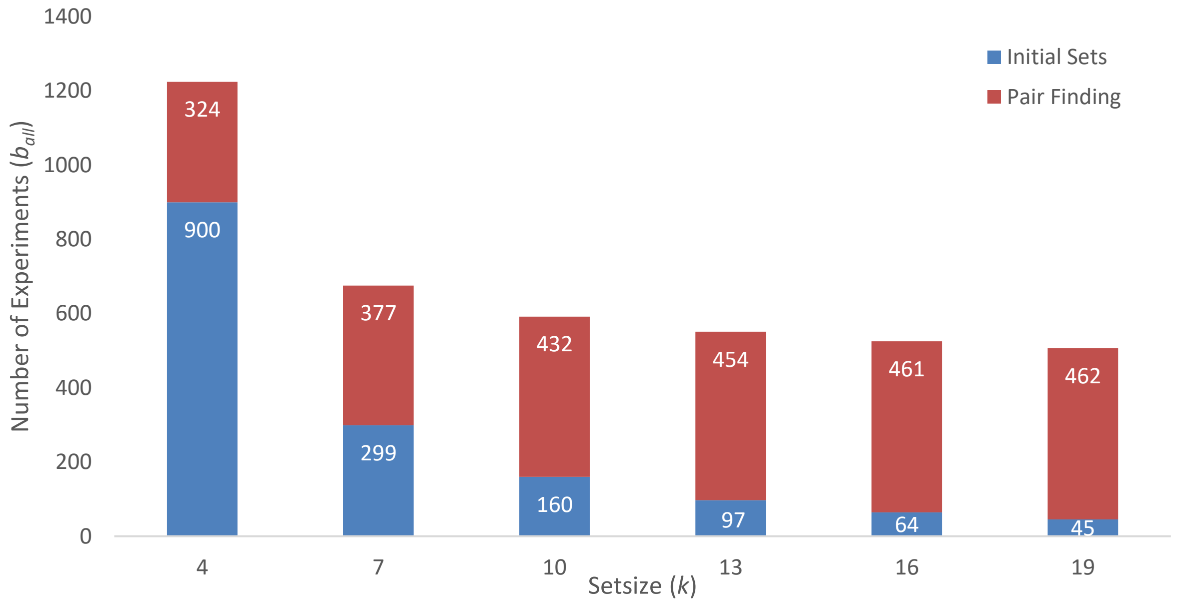

Figure S5: Approximation for the number of experiments needed to find all cooperative pairs in dependence on the chosen set size (Expected hit chance h= 0.5%). The greedy algorithm (2.3.2) was used for set construction and the number of experiments needed for the pair finding by one-step deconvolution the was approximated (8+4 experiments per deconvolution). The approximation was made that only one pair exists in every set while no deconvolution errors occur.

In case of utilizing the convolutional strategy for pairwise design issues presented here for related screens, the following procedure should be followed (for details on the improved procedure and full description, please consult section 2.7.5):

1. Estimation of the expected hit probability.
2. Determination of the maximum experimentally possible set size (2.2.2).
3. Determination of the theoretically optimal set size (2.7.5).
4. In case of uncertainties with respect to the above parameters, prefer smaller set sizes.

2.3 Software for the Construction of Screening Sets

2.3.1 Initial Software Approach (Swap algorithm)

Description of the algorithm

The swap algorithm aims for given values of n, k and a predetermined number of sets b to find a good allocation of the $k \cdot b$ numbers on the different sets. For this purpose, one defines a cost value $C \geq 0$, by considering all possible pairs of numbers and counting how many pairs are not present in the b sets. A successful block design implies $C = 0$. Thus, one has to deal with a minimization problem.

The swap algorithm, developed for this purpose, works as follows:

1) Initialization: We start with an initial distribution of the k numbers on the b sets so that, first, all numbers appear with similar frequency (the difference being at most unity) and, second, a set does not contain two identical numbers. One simple option is to randomly sort the n numbers in different order and to allocate the different arrangements of the n numbers successively to the b sets, each with k<n numbers. By construction each set then only contains different numbers. In general, the initial choice implies a large value of C.
2) Random selection of two elements from two randomly chosen sets.
3) Swapping of these elements if the following conditions are fulfilled: (a) C does not increase, (b) there are still no identical numbers in one set.
4) Stop of the routine after a predetermined number of iterations (denoted *iter*).
5) In case that the final value of C is positive, iteration of steps (2)-(4) with different random choices for a predetermined maximum number r_{swap} of times.

6) If no block design with $C = 0$ has been found, repetition of steps (1)-(5) for a larger value of b. This is continued until $C = 0$ is fulfilled.

In practice, for the swap algorithm the quality of the block design as expressed by the final value of b is very similar than for the greedy algorithm. However, it requires by far more CPU time in the relevant range of parameters (see below). One notable exception of only academic interest is the fact that in contrast to the greedy algorithm the swap algorithm succeeded to find the Steiner system with $n = 49$ and $k = 7$.

Practical Usage

Before using the presented software for the construction of screening sets or blocks the number of compounds which need to be tested for cooperative behavior (n) should be given. Subsequentially to determine the block size (k) the guide, as given in 2.2.3., shall be followed. After having the finished the experimental part of this project, a more efficient algorithm was found for the construction of sets. It generates excellent solutions for sets/blocks in a robust and time-efficient fashion (2.3.2). In case that the overall number of compounds n is below 100 the LA JOLLA COMBINATORICS REPOSITORY can be used to find differently optimized block designs.[17] **In cases $n \geq 100$ the utilization of the presented greedy algorithm is highly recommended.**[16] If the initial software approach shall be utilized the input file (input.dat) has to be configured by changing the integers in line one, two, three, five, seven and eight (Table S3).

Table S3: Explanation of the input file (input.dat) for the applied set construction software

Line	Variable	Explanation	Example (this screening)
1	k	Size of individual sets/blocks	10
2	n	Number of compounds	100
3	b	Number of sets	159
4		Empty line	
5	Δb	Increase of b if no block design was found	1
6		Empty line	
7	$iter$	Number of iterations per run	20000
8	r_{swap}	Number of repetitions for every value of S	20

Since the final number of sets (Line 3, Table S3) is usually unknown a starting point needs to be given for this value. In practice the theoretical minimum b_{min} (2.3.1 Mathematical Background) can serve as a starting point:

$$b_{min} = \left\lceil \frac{\binom{n}{2}}{\binom{k}{2}} \right\rceil$$

By choosing b_{min} initially the program will search for a block wise design for b_{min} while increasing b every r_{swap} steps by Δb until a solution has been found. In cases in which large numbers of n are required Δb can be increased while *iter* and r_{swap} can be decreased to get a result for b_{found} which is close to the obtainable minimum. If the number of experiments (b) needs to be decreased subsequential a second search can be conducted with decreased Δb and increased *iter* and r_{swap}. Thereby a value lower but close to b_{found} should serve as starting point. If needed this procedure can be repeated. The final sets can be found in the "results.dat" file, whereby each set is printed in a individual line. The optimization process can be following by observation of the "control.dat" file which displays the number of sets ("vectors") and not included pairs ("error") for every tested number of sets b and repetitions r.

Installation and Execution

The software is written in C/C++ and can be compiled using any C/C++ compiler. We highly recommend using the "GNU Compiler Collection" (GCC) with the command (gcc -O3 convolution_maker.c -o convolution_maker.exe) After compilation (on a Windows System) the executable (.exe) needs to be started within the same folder as the input.dat file.

2.3.2 Improved Software Approach (Greedy Algorithm)

Background

In the literature, one can find algorithms that can be applied for a heuristic search of a block design. Here, we resort to the recently published greedy algorithm, which has been proven superior to previous approaches.[16] This algorithm guarantees that each pair appears at least once and that the number of blocks is comparably small. Furthermore, it can be applied to any value of n and k, if the computer's memory and the user's time permit. Naturally, with this (or other) heuristic approaches, it is not possible to see whether block designs with significantly smaller

values of *b* would be available. Later we report a test of the algorithm in the parameter range of $n = 10$ to 1000 and $k = 5$ to 500. For details on the background and alternative solutions please consult MACDONALD et al.[16]

Theoretical Function

The key idea of the greedy algorithm to is generate blocks one after the other according to criteria which reduce the number of unseen pairs in an optimum manner. In case that this criterion does not clearly distinguish between different numbers, a random number generator is used for the decision. Therefore, the greedy algorithm is not deterministic and it is advisable to perform independent runs with different seeds. The algorithm was implemented according to MACDONALD et al..[16] In that reference a detailed description of the algorithm can be found.

Practical Usage

Since the software designs sets in a greedy fashion until all pairs are covered, only three input parameters are needed for the set construction. These are the number of compounds in the screen *n*, which can be set in the first line, the chosen size of every set *k* and the number of repetitions r_{greedy} which defines how often the greedy set construction should be repeated with different seeds. Thereby in every run a new seed will be used giving different sets and potentially better solutions for the number of sets *b*. The best solution will be kept and can be found in the "data_greedy.dat" file. This file shows *n*, *k* and *b* in the first line followed by the constructed sets given as one set per line (Table S4).

Table S4: Explanation of the input file (input_greedy.dat) for the applied set construction software.

Line	Variable	Explanation	Example (this screening)
1	n	Number of compounds	100
2	k	Size of individual sets/blocks	10
3	r_{greedy}	Number of repetitions	100

Installation and Execution

The software is written in C/C++ and can be compiled using any C/C++ compiler. We highly recommend using the "GNU Compiler Collection" (GCC) with the command (gcc -O3 greedy.c

-o greedy.exe). After compilation (on a Windows System), the executable (.exe) needs to be started within the same folder as the input.dat file. Depending on the used operating system variables have to the reduced in size. Especially, on windows systems stack overflow errors can occur if “NMAX” is chosen too largely. We recommend using this variable, which corresponds to number of compounds *n*+1, as needed.

2.3.3 Comparison of different approaches

The runtime of the swap algorithm strongly depends on the size *n*, as well as *k*, and takes extremely long if *b* is chosen unrealistically. In the case of high n (>250), the runtime can exceed the domain of a few hours towards multiple days or the timeframe of weeks, even for efficient settings. Therefore, we recommend following the instructions above to get a rough information about the size of the practical minimum of *b* before iteratively lowering *Δb,* while raising the number of repetitions and iterations.

By testing possible settings for *iter*, r_{swap} in case of the swap algorithm related and r_{greedy} for the adapted greedy algorithm it can be found, that the results of the former strongly depend on these parameters, while they only influence the later slightly. Thereby, it was found that the number of iterations per run (*r*), i.e., changes that are made within the designed sets, strongly influences the finally found set, while the number of repetitions only influences the outcome slightly and strongly in case of smaller values or r_{swap}. For the given settings (n = 100, k = 10), the best solution was found after a calculation time of 11.5 days (Table S5). From the perspective of calculation time, the algorithm scales linearly with respect to the parameters r_{swap} and *iter*. The greedy algorithm delivers good to excellent results for all tested settings even though a higher number of repetitions (r_{greedy}) yields in slightly better results. Thereby, the algorithm also scales linearly with respect to r_{greedy} while it has to be mentioned that the computational costs for the same results can be reduced by approximately two orders of magnitude compared to the swap algorithm. Therefore, and because of the high robustness/independence of the chosen input parameters, the usage of the greedy algorithm is recommended for the design of future screenings (Table S6).

Table S5: Number of sets (*b*) found using the swap set design algorithm in dependence on *iter* and r_{swap}. Timings (min) are given in brackets.

r_{swap}\ *iter*	10	100	1000	10000	100000
**1**	202 (0.1)	182 (0.4)	165 (2.4)	159 (18.9)	158 (180.7)
**5**	199 (0.4)	181 (1.7)	165 (10.9)	159 (91.3)	157 (849.2)
**10**	199 (0.6)	177 (3.0)	165 (21.8)	159 (181.4)	157 (1715.0)
**50**	196 (2.5)	177 (14.8)	164 (106.0)	157 (864.7)	156 (8278.8)
**100**	196 (5.0)	175 (28.4)	164 (210.9)	157 (1708.9)	156 (16535.0)

Table S6: Number of sets (b) found using the adapted greedy set design algorithm in dependence on r_{greedy}. Timings (min) are given in brackets.

r_{greedy}	1	5	10	50	100	500	1000	5000	10000
	160 (0.0)	158 (0.0)	158 (0.0)	158 (0.0)	158 (0.1)	157 (0.3)	157 (0.6)	157 (3.3)	**157 (7.4)**

In addition to tests in dependence on the input parameters r_{swap} and *iter*, both algorithms were compared with regard to the number of compounds (n) and the set size (k). To achieve comparable results in a reasonable time period (approximately one week), comparably simple settings were used for the swap (*iter* = 100, r_{swap} = 1) and greedy (r_{greedy} = 10) algorithm. Overall k was varied between 5 and 500 (5, 10, 50, 100, 500) while n was varied between 10 and 1000 (10, 50, 100, 500, 1000).

Table S7: Number of sets (b) found using the swap set design algorithm in dependence on the set size (k) and number of substrates (n). Timings (s) given in brackets. Jobs were cancelled after one week of time (*iter* = 100, r_{swap} = 1).

k\n	10	50	100	500	1000
**5**	7 (0.0)	153 (10.7)	596 (588.6)	Not finished	Not finished
**10**		45 (0.8)	182 (104.2)	4175 (246309.2)	Not finished
**50**			9 (0.0)	Not finished	Not finished
**100**				Not finished	Not finished
**500**					Not finished

Table S8: Number of sets (b) found using the adapted greedy set design algorithm in dependence on the set size (k) and number of substrates (n). Timings (s) given in brackets. Jobs were cancelled after one week of calculation time (r_{greedy} = 10).

k\n	10	50	100	500	1000
**5**	7 (0.0)	147 (0.1)	572 (0.8)	Not finished	Not finished
**10**		37 (0.0)	159 (0.4)	3523 (618.3)	Not finished
**50**			6 (0.0)	169 (131.5)	789 (3516.8)
**100**				6 (17.2)	168 (2252.5)
**500**					167 (257.7)

In case of the swap set design algorithm, solutions for $n = 10, 50, 100$ have been found for every k, while for n = 500, only the result for $k = 10$ has been found. In case of lower k, a solution has not been found in the time period of one week (Table S7). For $k > 50$ (100, 500) internal errors occurred related to the shortage of RAM and the size of internal variables. For the greedy algorithms, set designs have been found even in the case of high n (1000, i.e., 499500 pairs), while even higher values are possible. Thereby time was a limiting factor for the determination of sets with high n and small k wherefore some solutions have not been found in the scope of one week. Overall, it has been found, that the greedy algorithm poses superior with respect to the calculation time, found solutions and stability (Table S8).

It has to be mentioned, that the algorithm is not optimized on runtime, since this optimization would go far beyond the scope of this work. It is reasonable to that an implementation which is optimized on hardware requirements and on runtime of individual functions might enable the use of larger values for n. It has to be clarified that different operating systems and hardware was used for the benchmarks regarding r_{swap}, *iter* and n, k. Therefore, absolute timings are not comparable while results and general trends should not be influenced by this. For the direct comparison of both algorithms the same operating system and hardware was used.

2.3.4 Availability

The software can be found using the following link:

https://zivgitlab.uni-muenster.de/ag-glorius/published-paper/coop_screen

A Python implementation extending the greedy set design algorithm to t = 3 and λ >= 1 can be found using the following link:

https://github.com/FelixKatz77/GroupDesign

2.4 Screening of 159 Sets of 10 Compounds each

2.4.1 Background

The aim of this convolutional screening was to efficiently detect cooperative interactions between substrates, i.e. the change in the substrates' electronics by the copresence of the corresponding partner. Since generally utilizable changes in the electronic structure (e.g. raise

of the HOMO- or reduction of the LUMO-energy) of substrates are searched for, a probe is needed. Another prerequisite for such a screen is that these changes can be probed quickly, while also convolutive set design could be applied. One efficient possibility to detect such changes in the electronic energy rapidly is to measure the quenching of a versatile photocatalyst. In this context, quenching of the fluorescence of a photocatalyst, by a substrate pair that individual compounds have not quenched individually, would mean that cooperativity causes a change in the compound(s) electronic properties. Therefore $[Ir(dF(CF_3)ppy)_2(dtbpy)][PF_6]$ (**PC1**) was chosen as photocatalyst as it is able to interact (i.e. getting quenched) with the substrate in multiple ways: It can serve as an oxidizing ($E^{1/2}$ (M^-/M^*) = 1.21 V), reducing ($E^{1/2}$ (M^*/M^+) = -0.89 V), as well as an energy transfer agent ($E(T_1)$ = 63 kcal/mol) in its excited state[18].

Since it is assumed that cooperative interactions are particularly useful when the properties of a substance/combination are altered strongly, the goal of such a screening is the detection of these significant changes (i.e. impacting cooperative interactions). Even though cooperative interactions could be found applying the lower threshold of 50% intensity reduction (99.9%-quantile borders, 2.2.2.), the upper threshold of 60% was chosen to find strong and thereby usable cooperative interactions (2.2.2., Table S3). Following this, sets that showed a quenching fraction of more than or equal to 60% were classified as "Hit-Sets" and deconvolved as described in the following. For all of these sets, the origin of the quenching could be clarified by using the analysis outlined below. Sets with a quenching fraction between the upper and lower threshold (50 and 59%) form an in-between group ("Borderline Hit-Sets") in which cooperative interaction exhibited are comparably weak.

2.4.2 Experimental Details

Stock solutions of the photocatalyst (0.1 mM) and the 100 non-quenching compounds (250 mM) were prepared in degassed MeCN. To ensure good reproducibility and low errors during the quenching study, the fluorescence of the photocatalyst $[Ir(dF(CF_3)ppy)_2(dtbpy)][PF_6]$ (**PC1**) was probed multiple times until reproducible intensities could be achieved. Further, the photocatalyst fluorescence was probed repeatedly every 8 to 10 sets, and no significant changes in fluorescence intensity (e.g. by introducing oxygen) were observed. To evaluate the screening sets, 100 µL of the catalysts and 100 µL of each of the stock solutions of the respective 10 substrates were pipetted into the cuvette. To determine the

percentage of quenching, the measured fluorescence intensity at 504 nm was directly compared with the previously measured fluorescence of the pure catalyst at the same wavelength.

2.4.3 Results

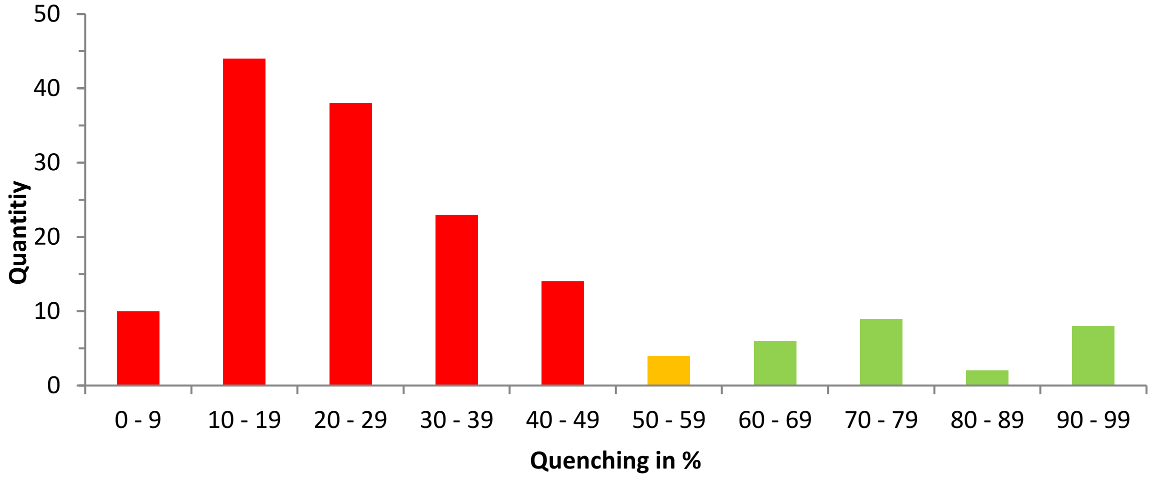

Figure S6: Quantity of screened sets quenching the photocatalyst in given ranges.

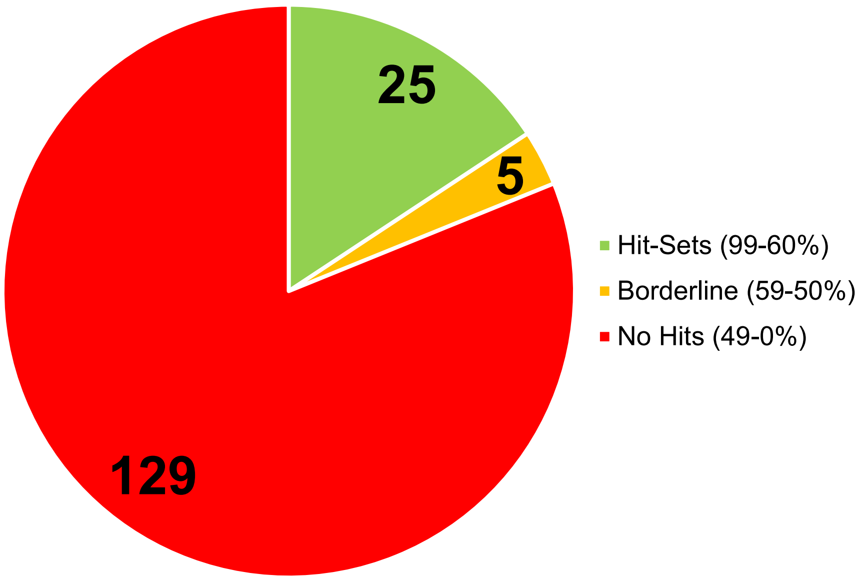

Figure S7: Chart representing ratio of hits, borderline hits and no hits acquired using our convolution screening approach in order to demonstrate the suitability to identify cooperative interactions.

Table S9: Results of the convolution screening of 159 investigated sets and the determined quenching of the photocatalyst [Ir(dF(CF_3)ppy)$_2$(dtbpy)][PF_6] (**PC1**) in the respective set.

Set	Compound N° S.										Q %
**1**	1	2	3	4	5	6	7	8	9	10	24%
**2**	11	12	13	14	15	16	17	18	19	20	20%
**3**	21	22	23	24	25	26	27	28	29	30	20%
**4**	31	32	33	34	35	36	37	38	39	40	54%
**5**	41	42	43	44	45	46	47	48	49	50	15%
**6**	51	52	53	54	55	56	57	58	59	60	93%
**7**	61	62	63	64	65	66	67	68	69	70	1%
**8**	71	72	73	74	75	76	77	78	79	80	19%
**9**	81	82	83	84	85	86	87	88	89	90	36%
**10**	91	92	93	94	95	96	97	98	99	100	22%
**11**	1	11	21	31	41	51	61	71	81	91	68%
**12**	2	12	22	32	42	52	62	72	82	92	16%
**13**	3	13	23	33	43	53	63	73	83	93	11%
**14**	4	14	24	34	44	54	64	74	84	94	16%
**15**	5	15	25	35	45	55	65	75	85	95	26%
**16**	6	16	26	36	46	56	66	76	86	96	30%
**17**	7	17	27	37	47	57	67	77	87	97	6%
**18**	8	18	28	38	48	58	68	78	88	98	97%
**19**	9	19	29	39	49	59	69	79	89	99	19%
**20**	10	20	30	40	50	60	70	80	90	100	28%
**21**	5	19	22	27	36	50	53	62	71	92	21%
**22**	10	15	18	32	35	41	70	73	79	83	40%
**23**	15	26	33	38	39	57	62	74	90	98	77%
**24**	1	6	18	27	39	51	52	74	83	96	46%
**25**	1	7	15	24	32	44	45	58	66	71	18%
**26**	7	10	12	18	54	62	66	77	80	93	22%
**27**	6	25	31	34	41	57	65	70	82	97	12%
**28**	10	18	25	37	46	47	53	82	94	95	60%
**29**	10	12	29	39	41	63	68	73	76	87	18%
**30**	9	16	31	45	48	51	62	83	89	100	94%
**31**	17	25	26	43	49	59	65	70	78	92	20%
**32**	3	8	21	23	46	61	66	82	90	95	14%
**33**	2	13	20	46	47	59	71	74	87	88	12%
**34**	9	15	20	31	37	55	56	72	80	92	14%
**35**	2	8	27	28	31	42	45	67	85	93	20%
**36**	16	21	27	30	32	43	54	67	68	76	35%
**37**	3	9	28	39	41	52	65	77	86	94	16%
**38**	17	23	36	38	41	55	76	85	89	100	65%
**39**	2	5	15	29	36	43	58	80	90	97	14%
**40**	5	11	44	46	52	57	67	79	81	98	41%

**80**	13	22	30	40	48	49	52	59	66	97	26%
**81**	6	15	22	30	37	51	64	67	75	91	76%
**82**	6	20	28	32	35	41	54	69	83	95	78%
**83**	1	15	23	47	49	68	71	80	82	86	12%
**84**	2	23	39	40	50	55	67	68	74	97	15%
**85**	13	26	34	35	41	54	62	73	81	92	17%
**86**	8	19	21	35	53	61	74	86	97	100	41%
**87**	1	3	5	12	18	20	26	38	93	99	30%
**88**	4	41	43	57	69	71	75	80	86	98	19%
**89**	1	17	23	34	35	48	49	56	69	74	17%
**90**	8	12	24	47	61	65	76	81	89	93	18%
**91**	26	34	42	48	63	67	71	80	89	95	48%
**92**	9	19	34	37	44	68	72	76	90	93	11%
**93**	5	7	9	15	19	21	33	40	50	81	10%
**94**	1	11	22	32	48	55	78	79	93	94	51%
**95**	4	6	17	22	68	80	85	88	94	99	21%
**96**	3	12	14	44	55	71	75	81	83	97	18%
**97**	13	14	21	22	39	44	46	56	65	100	25%
**98**	2	16	25	50	56	63	66	69	74	81	15%
**99**	20	21	22	36	45	47	64	74	78	83	6%
**100**	3	24	42	45	60	62	76	79	88	97	53%
**101**	12	21	34	42	50	57	59	77	83	90	25%
**102**	1	16	24	31	36	50	65	73	87	88	52%
**103**	1	14	15	28	57	59	63	76	93	95	18%
**104**	13	20	24	27	39	59	75	80	82	98	10%
**105**	20	24	34	43	51	53	65	66	79	85	63%
**106**	2	8	11	15	40	53	76	77	83	98	23%
**107**	5	7	14	31	43	74	78	82	86	99	34%
**108**	3	16	32	40	57	59	68	84	85	91	33%
**109**	31	35	43	44	68	77	81	88	91	95	26%
**110**	10	14	16	23	37	45	58	65	98	99	20%
**111**	6	8	19	29	55	68	75	82	96	100	40%
**112**	4	29	39	45	53	54	60	82	91	95	74%
**113**	7	22	33	35	50	58	64	76	82	93	31%
**114**	6	26	27	33	44	60	61	77	79	99	18%
**115**	5	6	17	37	45	46	59	62	69	73	23%
**116**	10	14	19	41	51	56	67	78	88	90	92%
**117**	6	13	14	40	42	43	58	77	89	96	11%
**118**	5	9	15	34	48	60	66	84	88	96	77%
**119**	4	16	28	32	46	64	77	80	81	97	26%
**120**	9	18	22	30	43	45	57	70	81	86	31%

**41**	17	32	33	40	48	54	65	75	86	90	**31%**
**42**	4	12	18	28	33	40	45	56	87	94	**23%**
**43**	4	15	28	30	38	46	56	70	89	94	**85%**
**44**	10	11	18	28	36	74	75	89	91	92	**31%**
**45**	8	9	14	26	44	53	70	75	80	87	**12%**
**46**	6	12	28	30	53	71	78	84	90	96	**24%**
**47**	4	11	19	25	26	38	65	80	83	91	**70%**
**48**	1	22	25	38	40	64	73	90	96	99	**34%**
**49**	5	11	32	42	51	53	68	88	89	100	**93%**
**50**	2	3	8	19	37	48	49	54	64	87	**17%**
**51**	3	4	12	24	25	36	58	59	67	100	**38%**
**52**	6	20	23	36	42	63	70	81	94	96	**11%**
**53**	3	17	18	31	45	50	52	61	80	96	**31%**
**54**	9	10	15	42	46	54	61	71	85	99	**22%**
**55**	1	22	38	39	43	45	61	77	84	92	**33%**
**56**	5	7	28	49	59	60	61	72	83	94	**32%**
**57**	4	31	38	42	50	54	63	66	75	78	**55%**
**58**	11	29	39	47	58	66	70	84	85	98	**13%**
**59**	7	14	20	25	29	36	48	61	68	79	**5%**
**60**	5	24	41	46	56	57	64	68	83	92	**19%**
**61**	6	11	14	24	35	47	48	49	90	91	**22%**
**62**	9	14	19	30	32	58	62	73	74	95	**78%**
**63**	3	4	12	35	48	51	70	76	92	99	**87%**
**64**	8	13	25	26	29	32	50	51	86	94	**64%**
**65**	8	10	16	22	29	31	33	34	41	59	**21%**
**66**	5	8	22	30	39	51	63	69	77	92	**68%**
**67**	3	22	25	50	54	72	87	89	95	98	**77%**
**68**	16	38	40	47	48	51	53	69	72	82	**91%**
**69**	4	5	23	32	47	54	58	79	87	96	**37%**
**70**	14	18	23	29	35	38	52	59	60	64	**47%**
**71**	3	4	15	27	34	52	69	78	87	100	**43%**
**72**	7	16	23	31	46	49	60	75	84	93	**18%**
**73**	8	10	13	36	43	52	57	72	84	99	**29%**
**74**	9	10	17	27	38	39	48	64	72	81	**41%**
**75**	9	11	12	13	23	24	36	60	69	91	**7%**
**76**	7	11	18	21	34	45	52	63	72	90	**19%**
**77**	17	19	28	32	34	49	55	60	63	98	**28%**
**78**	6	17	21	36	38	51	87	93	97	98	**96%**
**79**	7	16	30	35	39	42	55	61	87	98	**30%**

**121**	17	37	41	49	53	58	66	81	83	99	**16%**
**122**	8	29	43	56	60	64	71	81	94	100	**7%**
**123**	2	10	17	24	38	40	71	79	86	95	**28%**
**124**	2	16	18	25	33	42	44	65	69	71	**14%**
**125**	18	20	25	49	67	76	77	84	97	100	**36%**
**126**	10	26	44	55	63	64	69	85	88	97	**41%**
**127**	1	47	60	62	67	75	86	87	92	94	**19%**
**128**	16	21	31	58	69	76	79	90	92	94	**15%**
**129**	13	19	20	26	31	33	45	47	52	68	**1%**
**130**	18	19	24	27	55	56	70	77	84	85	**31%**
**131**	21	25	37	39	52	70	71	75	88	93	**74%**
**132**	11	28	43	50	55	62	66	87	91	99	**30%**
**133**	9	24	25	33	35	63	67	72	96	99	**30%**
**134**	6	7	14	33	50	66	72	85	88	92	**30%**
**135**	3	9	11	47	56	73	78	82	97	98	**20%**
**136**	4	10	20	21	48	49	57	62	85	96	**14%**
**137**	8	17	20	42	56	62	73	79	84	91	**25%**
**138**	2	21	24	37	52	60	73	78	85	89	**8%**
**139**	11	20	37	42	44	59	64	86	89	96	**29%**
**140**	1	7	33	54	70	72	89	91	97	100	**10%**
**141**	3	29	30	40	41	60	65	72	74	93	**20%**
**142**	11	12	27	30	33	36	49	54	88	95	**16%**
**143**	2	7	14	26	27	41	65	69	84	96	**26%**
**144**	26	28	37	47	50	63	79	82	84	100	**46%**
**145**	21	33	35	46	51	55	78	80	84	95	**47%**
**146**	4	13	27	37	55	61	73	86	90	93	**25%**
**147**	2	5	13	16	52	70	76	78	91	95	**11%**
**148**	7	13	28	38	44	49	51	53	67	73	**99%**
**149**	26	27	34	40	46	58	63	72	86	91	**14%**
**150**	2	19	27	35	57	66	73	89	94	100	**28%**
**151**	23	29	40	44	57	61	62	78	88	92	**25%**
**152**	1	12	19	23	29	37	42	43	46	74	**9%**
**153**	17	23	29	30	36	44	67	77	82	83	**25%**
**154**	2	30	32	34	47	56	58	61	75	99	**48%**
**155**	1	12	13	30	31	53	64	79	85	98	**24%**
**156**	2	4	22	23	25	51	60	62	68	72	**43%**
**157**	7	39	56	59	64	69	78	81	93	95	**4%**
**158**	12	20	48	58	70	73	74	77	85	86	**27%**
**159**	21	35	47	55	71	78	81	99			**33%**

2.5 Initial One-Step Deconvolution of Screening sets

2.5.1 Theoretical Background for the One-Step Deconvolution of Screening-Sets

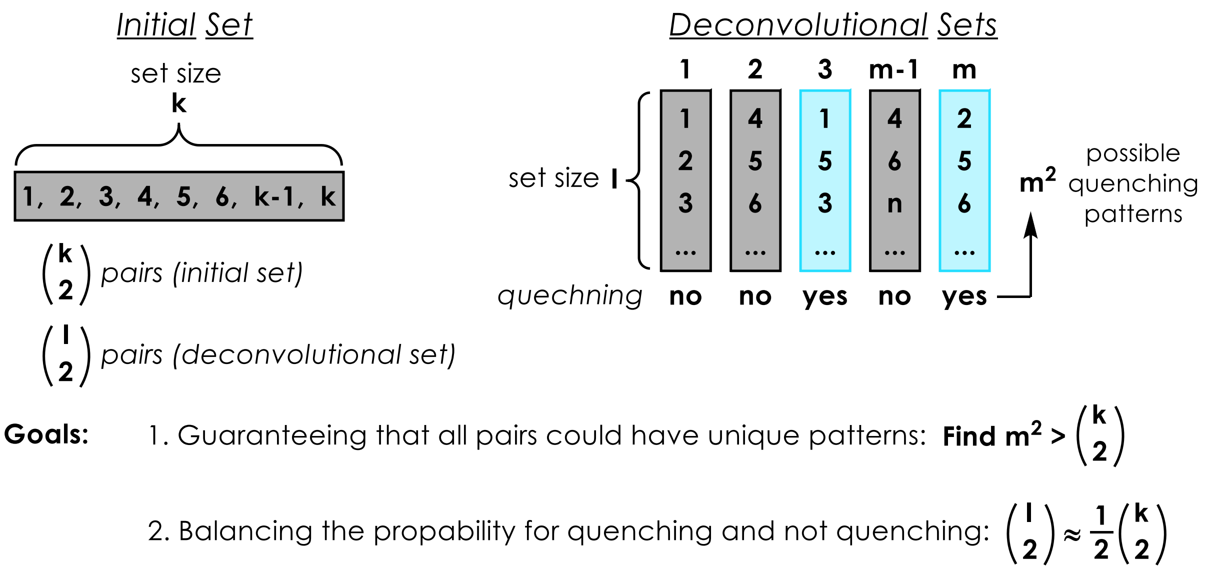

Figure S8: Visualization of the simplified theoretical prerequisites for the construction of deconvolutional sets for pair finding.

Since each set contains $\binom{k}{2}$ dual combinations, a pairwise experimental testing of them would be highly time-consuming even in the case k = 10 (45 possible pairs). To simplify this, it is possible to create m small sets with l substrates each in which the corresponding pairs occur several times. A necessary condition for the ability of identification of the active pair is given by the condition that the number of possible binary combinations (2^m) is larger than the number of possible pairs (Figure S8). This would yield m = 6 as the combinational minimum. To obtain an actual realization, we have written a simple program that generates m sets of length l with the condition that all numbers per set (drawn from the integers up to 10) are different. Then we checked whether for each combination of pairs, there exists at least one set where exactly one of these two pairs is present. This condition is equivalent to the requirement that each pair has its own signature (quenching pattern) when it is active, so that unique deconvolution is possible. In total 10^6 independent examples are analyzed along this line. In this way no realization has been identified for m = 6. Systematic exploration of all combinations (m, l) for $m \leq 9$ show that for $(m, l) = (7,7); (8,7); (8,8), (9,6); (9,7); (9,8); (9,9)$ realization can be found which allow a unique deconvolution. It is not surprising that the number of l = 7 substrates per set is favorable. There exist $\binom{7}{2}$ = 21 possible pairs per subset (about half of 45 possible pairs in total), so the expectation for "quenching" or "non-quenching" is about equal (Figure S8). For our actual screening, we choose not the minimum value of m = 7 sets but rather m = 8 sets (together

with *l*=7). In this way we obtain additional information for cases of additive quenching, three-component combinations or multiple pairs per set, etc., which can simplify manually designed deconvolution in those cases. The chosen realization is listed in Table S11. For the sake of clarity, it has to be mentioned that a one-step deconvolution, as presented in this work, can only guarantee finding the active pair if no other active pairs are present. As the probability for only one pair being able strongly depends on the true hit rate and the distribution of the hits (e.g. if one substrate is involved in multiple pairs the chances for multiple pairs being present in one set dramatically rises) and the set size an iterative strategy for pair finding was developed during the revision of this publication which has been proven to be able of finding all active pairs even if multiple pairs or single quenchers are present in parallel (2.7).

2.5.2 Practical Deconvolution and Final Hits

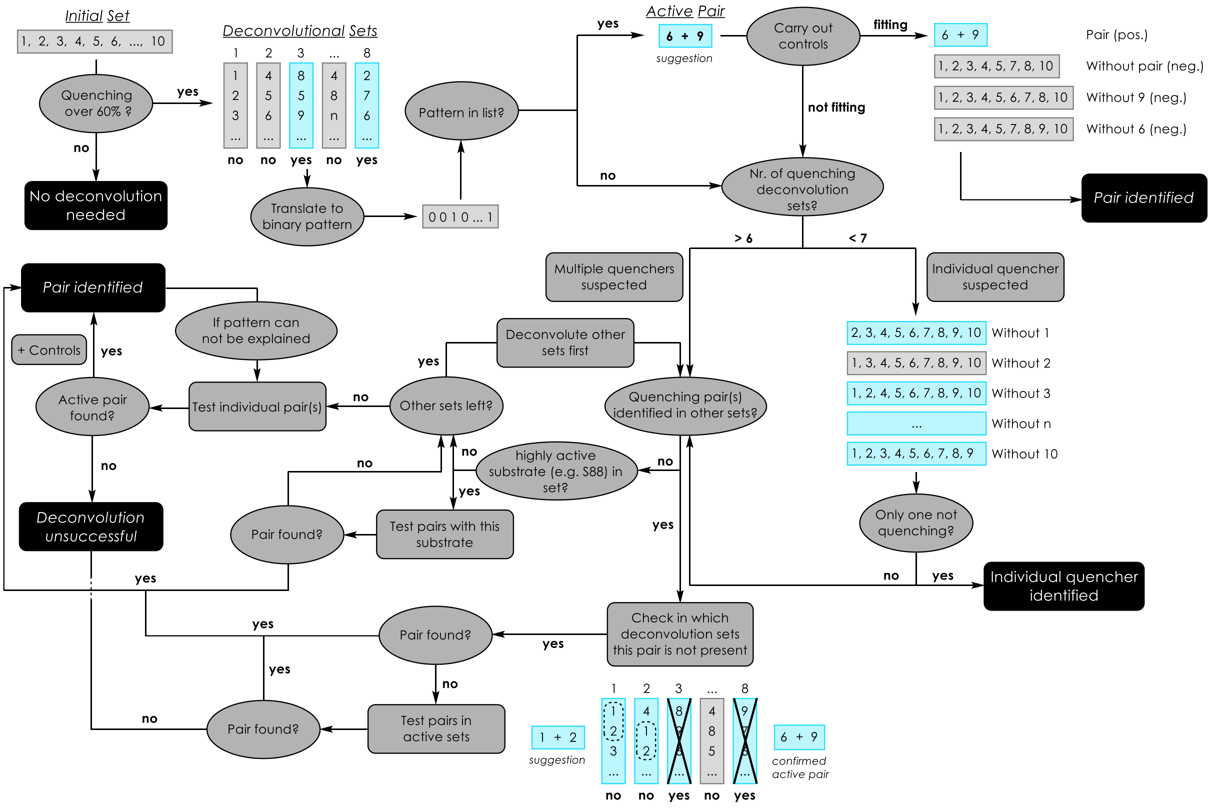

Figure S9: Flowchart representing the process of deconvolution using the initial one-step deconvolution approach.

In order to deconvolve the obtained hits (Table S9), the substrates of the respective sets were assigned to numbers from 1 to 10 (Table S11). These were then reassembled in 8 subsets of 7 substrates each and the quenching of the photocatalyst’s luminescence was measured. Stock

solutions of the photocatalyst (0.1 mM) and the 100 non-quenching compounds (250 mM) were prepared in MeCN. To ensure good reproducibility and low errors during the quenching study, the fluorescence of [Ir(dF(CF_3)ppy)$_2$(dtbpy)][PF_6] (**PC1**) was probed multiple times until reproducible intensities could be achieved. This procedure was repeated roughly every 20 measurements. To evaluate the convolution-sets, 100 µL of the catalyst stock solution, as well as 100 µL of each of the substrate stock solutions, were pipetted into the cuvette and diluted with 300 µL MeCN. To determine the quenching fraction, the measured fluorescence intensity at 504 nm was directly compared with the previously measured fluorescence of the pure catalyst at the same wavelength. To distinguish between quenching and non-quenching and to rule out additive effects of slightly quenching compounds, a threshold of 50% (2.2.2) was chosen, which means that a reduction of the fluorescence intensity by more than 50% can be considered as cooperative quenching. In this case, the lower threshold limit was chosen to make sure no pair can be overseen, thereby making it necessary to carry out a control experiment (see below). To be able to clearly deduce a cooperative quencher pair from the results of the 8 sets, it must be determined which pair is present in all quenching sets. For this purpose, quenching is interpreted as 1 and non-quenching as 0 (binary quenching pattern) and the corresponding pair can be assigned via Table S11. To verify the result, each determined cooperative quenching pair was investigated individually after the deconvolution using luminescence quenching. In addition, three negative controls were carried out: For the first and second control the set is tested excluding one compound of the deconvoluted pair while the other compound stays within the set. For the third control, eight compounds of the set are tested, excluding the identified pair. In some of the quenching sets, a single (decomposed) molecule, additive effects of non-quenching compounds, or interactions of more than two compounds were identified to be the reason for the observed quenching. In the case of a single (decomposed) molecule being the origin of quenching, the binary quenching pattern, given by the deconvolution, is not solvable with the given table (Table S11), but instead the pattern corresponds to a single number. This usually corresponds to a compound that decomposed throughout the screening studies. Leaving this compound out of the respective sets resulted in only a low quenching. Compound **S51** was identified as a monomolecular quencher in various sets, possibly due to decomposition (**Sets 63, 64, 66, 68, 81, 105**). In the case of additive-quenching effects of multiple compounds and poly molecular interactions, the binary code, given by the deconvolution, is not solvable, as the number of zeros is significantly higher than expected (6 or higher). Another possibility is that two or more dual interactions take place in the same set. In this case, the deconvolution using the binary code gives less zeros and a convolution can be performed by applying knowledge

about already known combinations and by testing a few possible binary combinations (**Sets 43, 47, 116, 118**). Following this procedure and verifying possible quenching pairs by testing them individually, 25 hit sets and 1 borderline hit set could be successfully deconvoluted (Table S10, Figure S6).

Table S10: Deconvoluted hits and the corresponding quenchers or cooperative quenching combinations (pairs).

Set	Compound N° S.										Q %	Origin of Quenching
**148**	7	13	28	38	44	49	51	53	67	73	99%	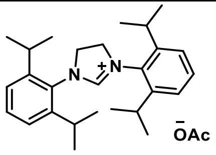 **S51**
**18**	8	18	28	38	48	58	68	78	88	98	97%	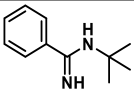 **S88** 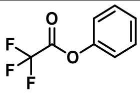 **S48** 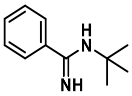 **S88** 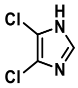 **S48**
**78**	6	17	21	36	38	51	87	93	97	98	96%	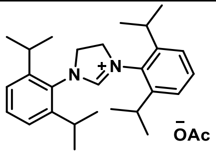 **S51** 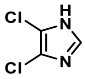 **S38** 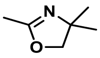 **S17**
**30**	9	16	31	45	48	51	62	83	89	100	94%	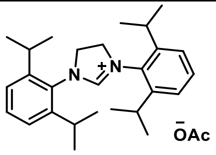 **S51**
**6**	51	52	53	54	55	56	57	58	59	60	93%	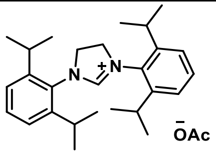 **S51**
**49**	5	11	32	42	51	53	68	88	89	100	93%	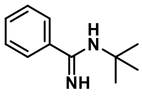 **S88** 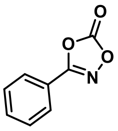 **S5**

**116**	10	14	19	41	51	56	67	78	88	90	**92%**	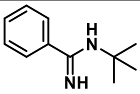 S88 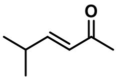 S10 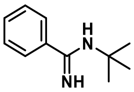 S88 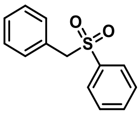 S19
**68**	16	38	40	47	48	51	53	69	72	82	**91%**	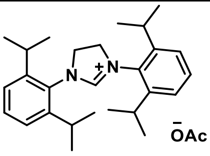 S51
**63**	3	4	12	35	48	51	70	76	92	99	**87%**	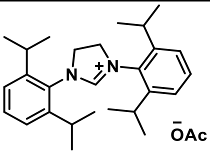 S51
**43**	4	15	28	30	38	46	56	70	89	94	**85%**	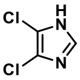 S38 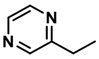 S4 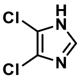 S38 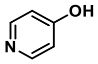 S15
**62**	9	14	19	30	32	58	62	73	74	95	**78%**	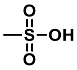 S95 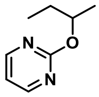 S32
**82**	6	20	28	32	35	41	54	69	83	95	**78%**	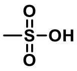 S95 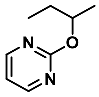 S32
**23**	15	26	33	38	39	57	62	74	90	98	**77%**	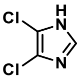 S38 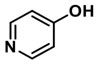 S15
**67**	3	22	25	50	54	72	87	89	95	98	**77%**	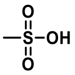 S95 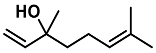 S22

**118**	5	9	15	34	48	60	66	84	88	96	**77%**	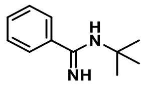 S88 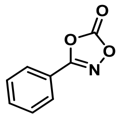 S5<br>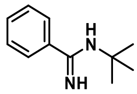 S88 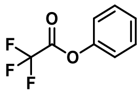 S48<br>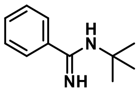 S88 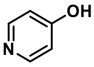 S15
**81**	6	15	22	30	37	51	64	67	75	91	**76%**	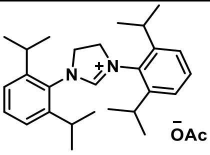 S51
**112**	4	29	39	45	53	54	60	82	91	95	**74%**	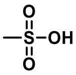 S95 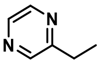 S4
**131**	21	25	37	39	52	70	71	75	88	93	**74%**	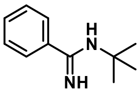 S88 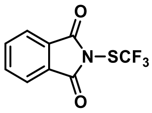 S75
**47**	4	11	19	25	26	38	65	80	83	91	**70%**	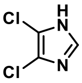 S38 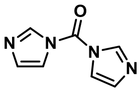 S11<br>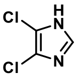 S38 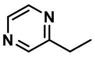 S4
**11**	1	11	21	31	41	51	61	71	81	91	**68%**	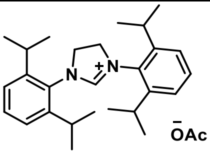 S51
**66**	5	8	22	30	39	51	63	69	77	92	**68%**	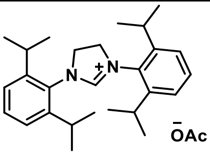 S51
**38**	17	23	36	38	41	55	76	85	89	100	**65%**	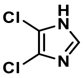 S38 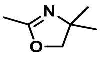 S17

**64**	8	13	25	26	29	32	50	51	86	94	**64%**	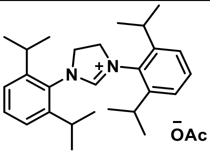 **S51**
**105**	20	24	34	43	51	53	65	66	79	85	**63%**	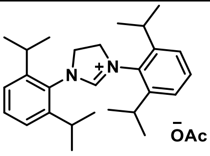 **S51**
**28**	10	18	25	37	46	47	53	82	94	95	**60%**	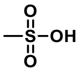 **S95** 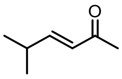 **10**
**57**	4	31	38	42	50	54	63	66	75	78	**55%**	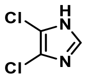 **S38** 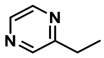 **S4**

Table S11: Sheet for the deconvolution and assignment of screening hits – Enumeration of the compounds from 1 to 10, testing 8 subsets with 7 compounds each. Comparing the result (quenching = 1, no quenching = 0) with the given binary table gives the active pair.

**Set**	**1**	**2**	**3**	**4**	**5**	**6**	**7**	**8**	
**Compound Nr.**	1	2	1	1	2	1	1	1	
	2	4	4	2	3	2	3	2	
	3	5	6	3	5	3	4	5	
	6	6	7	4	6	4	5	6	
	7	7	8	5	8	5	8	7	
	8	9	9	6	9	7	9	8	
	9	10	10	9	10	10	10	10	Pair
**Q?**	Yes	No	No	No	No	Yes	No	No	*3+7*

**Nr.**	**Nr.**	**Binary Result**							
6	*9*	1	1	1	1	1	0	0	0
6	*7*	1	1	1	0	0	0	0	1
7	*9*	1	1	1	0	0	0	0	0
2	*6*	1	1	0	1	1	0	0	1
2	*9*	1	1	0	1	1	0	0	0
2	*7*	1	1	0	0	0	1	0	1
1	*9*	1	0	1	1	0	0	1	0
1	*6*	1	0	1	1	0	0	0	1
8	*9*	1	0	1	0	1	0	1	0
6	*8*	1	0	1	0	1	0	0	1
1	*7*	1	0	1	0	0	1	0	1
1	*8*	1	0	1	0	0	0	1	1
7	*8*	1	0	1	0	0	0	0	1
2	*3*	1	0	0	1	1	1	0	0
3	*9*	1	0	0	1	1	0	1	0
3	*6*	1	0	0	1	1	0	0	0
1	*3*	1	0	0	1	0	1	1	0
1	*2*	1	0	0	1	0	1	0	1
3	*8*	1	0	0	0	1	0	1	0
2	*8*	1	0	0	0	1	0	0	1
3	*7*	1	0	0	0	0	1	0	0
4	*9*	0	1	1	1	0	0	1	0
4	*6*	0	1	1	1	0	0	0	0

**Nr.**	**Nr.**	**Binary Result**							
9	*10*	0	1	1	0	1	0	1	0
6	*10*	0	1	1	0	1	0	0	1
4	*10*	0	1	1	0	0	1	1	0
7	*10*	0	1	1	0	0	1	0	1
4	*7*	0	1	1	0	0	1	0	0
2	*5*	0	1	0	1	1	1	0	1
5	*9*	0	1	0	1	1	0	1	0
5	*6*	0	1	0	1	1	0	0	1
4	*5*	0	1	0	1	0	1	1	0
2	*4*	0	1	0	1	0	1	0	0
5	*10*	0	1	0	0	1	1	1	1
2	*10*	0	1	0	0	1	1	0	1
5	*7*	0	1	0	0	0	1	0	1
1	*4*	0	0	1	1	0	1	1	0
8	*10*	0	0	1	0	1	0	1	1
1	*10*	0	0	1	0	0	1	1	1
4	*8*	0	0	1	0	0	0	1	0
3	*5*	0	0	0	1	1	1	1	0
1	*5*	0	0	0	1	0	1	1	1
3	*4*	0	0	0	1	0	1	1	0
3	*10*	0	0	0	0	1	1	1	0
5	*8*	0	0	0	0	1	0	1	1

2.6 Statistical Evaluation of the Convoluted/Deconvolution Approach

To evaluate the developed screening process the false positive and false negative rate (i.e. the wrongly determined and the overseen hits) have to be determined. For this purpose, the ground truth would be needed which can be determined by testing all pairs individually for luminescence quenching while predicting the cooperativity as difference between the additive quenching and the pairs' quenching. Since a testing of all 4950 pairs is experimentally not possible within the scope of this project, a subspace of 20 individual compounds, thereby $\binom{20}{2}$=190 pairs and 3.8% of the full space was examined. Due to the high experimental effort the sampling of a significantly larger space appeared to be unreasonable. Statistically, this subspace also includes 0.58 cooperative pairs, wherefore one pair (**C10**, **S19**, **S88**) was randomly chosen. The other 18 compounds (**S2**, **S7**, **S11**, **S14**, **S28**, **S33**, **S35**, **S40**, **S41**, **S43**, **S52**, **S53**, **S59**, **S71**, **S74**, **S80**, **S84**, **S87**) were randomly drawn from among all available compounds.

2.6.1 Determination of the Precision

Since all 15 hit pairs have been tested individually within the process of deconvolution and thereby have exhibited cooperative luminescence quenching, it can be assumed that no false positives have been found applying the block-wise or iterative deconvolution procedure (2.7 to 2.8). By the utilization of these strategies, it was possible to exclude non-dual and thereby non cooperatively quenching hit sets. By this means, the precision can be determined to be 1.0. In subsequent approaches aimed at elucidating the cause of the cooperativity, it was found for three pairs (**C4**, **C5**, **C6**) that the cooperativity does not originate from the respective individual compounds themselves but from impurities and degradation products. Since a strong cooperative effect is present, also confirmed by dual testing, these hits cannot be considered as false positives.

$$\boldsymbol{precision = \frac{true\ positives}{true\ positives + false\ positives} = \frac{15}{15+0} = 1.0}$$

2.6.2 Determination of the Recall

For the determination of the false negative rate 20 non-quenching compounds were sampled and all possible dual combinations and substrates were tested individually on quenching using

$[Ir(dF(CF_3)ppy)_2(dtbpy)][PF_6]$ (**PC1**) as photocatalyst. Stock solutions of the photocatalyst (0.1 mM) and the substrates (250 mM) were prepared in degassed MeCN. To ensure a good reproducibility and low errors during the quenching studies, the fluorescence of $[Ir(dF(CF_3)ppy)_2(dtbpy)][PF_6]$ (**PC1**) was probed multiple times until intensities varied around a fixed value. In doing so, 100 μL of the catalyst stock solution was pipetted into a cuvette and diluted with 900 μL of MeCN. To determine the quenching of the individual substrates again, 100 μL of the catalyst stock solution and 100 μL of the substrate stock solution were pipetted into the cuvette and diluted with 900 μL of MeCN, resulting in a photocatalyst:substrate ratio of 1:2500. For testing the dual combination 100 μL of the catalyst stock solution and 100 μL of each substrates stock solution were pipetted into the cuvette and diluted with 800 μL of MeCN. The fluorescence was measured for every individual compound and for all dual pairs. Since a determination of thresholds based on a normal distribution of additive errors, as presented in 2.2.2, could enable biases by the selected subset of compounds, and due to the testing of only two compounds, a background subtraction was performed. Therefore, the luminescence quenching percentage of a pair's individual compounds is subtracted from the pair's quenching, giving the cooperative fraction of the determined quenching percentage. To keep errors as low as possible, the catalyst's luminescence was measured three times after the completion of 20 measurements. The reference value to determine the quenching percentage was given by a linear function between the mean of the last and the newly the measured catalyst luminescence. Even though this method reduces the influence of systematic errors (e.g. changing oxygen levels) a significant random error still remains in every measurement and therefore even in the cooperative fraction of the determined quenching percentage. To avoid the identification of false positives a relatively low threshold was set on 10% for identifying relevant cooperative effects. For clarification, it has to be mentioned that cooperativity is an in theory quantifiable phenomenon, meaning that a binary classification can only be made by means of a delimiting threshold. This threshold is set to clearly detect significant cooperative interactions (i.e., which are strong enough to cause significant changes in the substrate's electronic structure), but since the chosen photocatalyst is able to detect different types of cooperativity, no quantitative statement can be made on the absolute strength of the detected cooperative effects.

Three pairs (**C10**, **C16**, **C17**) have been identified to exhibit non-additive queening above the posed threshold, thereby showing a clear cooperative interaction by this dual testing (Table S12). Two previously unknown cooperative pairs have been detected.

Within the convoluted screening, both pairs have been present in sets (**Set 18**, **Set 49**) exhibiting strong luminescence quenching. In both cases, the block-wise deconvolution and manual testing revealed one, respectively two cooperative pairs incorporating amidine **S88**. Thus, it is possible that in the process of deconvolution, unclear binary patterns arose, which made an unambiguous assignment impossible due to the coexistence of several pairs with **S88**. In the subsequent manual deconvolution, the interaction between **S28/S53** and **S88** may have been overlooked. This can be prevented by using the later developed iterative deconvolution, which is capable of testing and excluding each pair by adaptive set design.

Table S12: Cooperatively interacting pairs as identified by dual testing. **C10** has been identified by the convoluted screening. **C16** and **C17** have not been found in this approach.

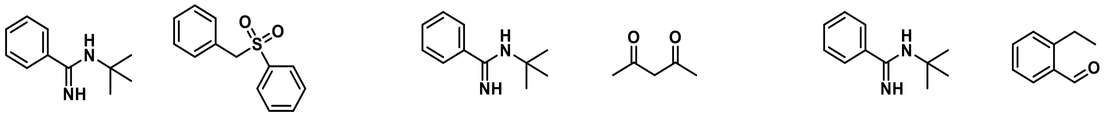

S88 S19 S88 S28 S88 S53

C10 **C16** **C17**

Based on this subset a recall can be determined:

$$\boldsymbol{recall_{subset}} = \frac{\boldsymbol{true\ positives}}{\boldsymbol{true\ positives + false\ negatives}} = \frac{\mathbf{1}}{\mathbf{1+2}} = \mathbf{0.33}$$

Even though the recall is relatively low and only 1 of 3 hits could be found due to issues in the deconvolution procedure only 3.8% of the full combinatorial space was sampled whereby this value is subject to a high degree of uncertainty. Since both newly found pairs (**C16**, **C17**) include the amidine **S88**, which has also been found to exhibit covalent and not-covalent cooperativity with different other compounds (3.1 to 3.6) this subspace could be subjected to a strong bias originated by the incorporation of this privileged compound.

2.6.3 Conclusion

Based on the precision and estimated recall a F_1-score can be determined:

$$\boldsymbol{F_1} = \mathbf{2} * \frac{\boldsymbol{precision * recall}}{\boldsymbol{precision + recall}} = \mathbf{2} * \frac{\mathbf{1 * 0.33}}{\mathbf{1 + 0.33}} = \mathbf{0.50}$$

This score provides a balanced performance metric and expresses that false positives should be avoided, whereas the discovery of true cooperative pairs should be maximized. Overall, no false positive pairs have been found thanks to the controls conducted during the block-wise

deconvolution. Since also the iterative deconvolution algorithms tests for individual pairs at the end of each deconvolution tree a drop in recall is not expected. In contrast to this, the developed screening approach allows to oversee pairs exhibiting a cooperative effect. Within this dual control, testing 3.8% of the full combinatorial space two new hits have been found. It has to be mentioned that supposedly these hits have been overseen within the deconvolution process due to the presence of other cooperatively interacting substrates. This could be avoided by the utilization of the developed iterative deconvolution procedure (2.7). No new cooperative pair could be identified that had previously been present in a non-quenching set of 10 compounds, underlining the efficiency and reliability of the developed convolutional approach. Based on the tested space, deactivating effects seem to occur remarkably rarely within the tested sets of ten compounds or to do not have a significant influence on the measurable quenching. In addition, these results underline the effectiveness of the chosen thresholds for finding cooperativity.

Since the goal of screening and especially convolutional approaches is to find new, unforeseen hits efficiently, overlooking possible hits cannot be seen as a failure but at best as a reduction of effectiveness. Thus, the expected hit rate (2.8) could still be increased significantly, i.e. by an order of magnitude, by the convolutional screening developed in this work, compared to the single test. Even though the dual testing could prove that hits can be overseen in the process of deconvolution, the determined recall and F1-score can only be seen as an approximation and could include a bias due to the presence of **S88** while covering only a small fraction (3.8%) of the full combinatorial space.

2.7 Interval Sectioning Algorithm for Pair Finding

2.7.1 Issues of a One-Step Deconvolution Approach

A major drawback of the initially used one-step deconvolution approach is that it is designed to allow only one pair to be active in a certain set. Provided that the expected hit probability can be estimated beforehand and the hits are distributed statistically, the risk of multiple pairs in a set can be omitted, however, this is not the case in the conducted screen. Indeed, one of three different substrates (**S38**, **S88**, **S95**) occurs in all 15 identified pairs, thus these compounds occur significantly more frequently in hits, suggesting that they are either easy to activate through cooperative interactions or can activate a variety of different substrates. Moreover, the presence of substrates that show quenching in sets without clearly identifiable cooperative

interactions (**S51**) complicates deconvolution. For both of these cases, direct identification of a potentially active pair cannot be performed by the quenching pattern of the deconvolution sets. Thus, different pairs and sets must be manually designed and tested to identify active pairs in these cases. Another consequence is, that the search for active pairs is mostly stopped, if any pair could be found or the quenching pattern could be explained encouraging the overseeing of relevant pairs. Dual testing of 190 pairs showed that two active pairs were missed when using the one-step deconvolution approach (2.5). To avoid this problem of reliable pair finding if single quenchers and multiple pairs are present simultaneously, an improved iterative approach was developed and tested during the revision process. In summary, additional analysis is required when the binary deconvolution pattern is ambiguous, typically due to multiple active pairs or weak signals and, thus,, static block designs cannot provide unambiguous resolutions.

2.7.2 Mathematical Background

We aim for the identification of an algorithm which generates blocks. For each block it is checked whether the active pair is part of that block or not. We will start with the simplest combinational problem which subsequently will be generalized. We assume that there is a single active pair and *search for an optimum way to identify this pair*. A straightforward approach is to consider all pairs individually until the active pair is found. This corresponds to finding a (n,2,2)-block design. Naturally, via systematically taking all pairs for the experimentally relevant example of n = 100 one has a Steiner system with $b = \binom{100}{2} = 4950$. This approach, implying to perform this large number of experiments, would in principle do the job but is, of course, highly inefficient.

To approach this combinational problem, we were inspired by the analogous question to identify a specific single number if exactly one number is active. Here the appropriate method is interval bisectioning. Starting, e.g. from n = 16, the numbers are grouped in two equally sized blocks $A_1 = \{1, \dots, 8\}$ and $A_2 = \{9, \dots, 16\}$. In the terminology of the block design we have k_1 = 8 and b_1 = 2. If A_1 is active one would apply the bisectioning idea to that block, thus generating $A_{1,1} = \{1, \dots, 4\}$ and $A_{1,2} = \{5, \dots, 8\}$, corresponding to k_2 = 4 and b_2 = 2. If A_1 is not active, in the strict mathematical framework, it would be clear that A_2 has to be active. However, in the experimental context, also in order to identify possible experimental errors, we always explicitly require identifying an active block before reducing the size of a block further. Thus, for the present example, one needs on average 1.5 experiments until one can continue with a

block size of $k_2 = 4$. This approach has to be continued until the fourth *level* is reached where $k_4 = 1$ and $b_4 = 2$. Thus, on average $1.5 * 4 \approx 6$ experiments have to be performed, whereas a simple successive testing of all numbers requires, on average, 8.5 experiments. For that small value of n, the gain in efficiency is not huge, but evidently for larger n, a dramatic improvement is achieved by the bisectioning because of the scaling of the required number of experiments with the logarithm of n rather than directly with n.

Naturally, this algorithm can be applied for any value of n by choosing $k_i = \left\lceil \frac{k_{i-1}}{2} \right\rceil$. When identifying $k_0 = n$ this iterative formula determines the sizes of the subsequent blocks. For example, the bisectioning of $\{1, \ldots, 7\}$ with block size 7 would generate the blocks $\{1,2,3,4\}$ and $\{1,5,6,7\}$ with block size 4. Here we have formally added the number 1 also to the second block in order to have blocks with fixed size.

For the simple problem of identifying a single number, the optimization problem can now be formulated within the language of block design. Starting with a value of n one reduces the size of the blocks from level to level until on the final level r one has $k_r = 1$. In this way one generates the sequence $n > k_1 > k_2 > \cdots > k_r$. The size of the blocks reads $b_1, \ldots, b_r$. Note that this general scenario includes very different approaches. E.g., the direct testing of individual numbers implies $r = 1, k_1 = 1, b_1 = n$ whereas the values for the interval bisectioning for the example $n = 16$ are given as $r = 1, k_1 = 8, k_2 = 4, k_3 = 2, k_4 = 1, b_1 = b_2 = b_3 = b_4 = 1$. The expected number B of experiments is given by $\left(\frac{1}{2}\right)[(b_1 + 1) + (b_2 + 1) + \cdots + (b_r + 1)\,] \equiv \left(\frac{1}{2}\right) B$. The goal is finding a procedure which renders B small. This is achieved by the interval bisectioning.

The problem of identifying active pairs can be formulated in a fully analogous manner. On each level r, characterized by the block size k_r, one needs to identify a minimum number of blocks b_r such that all pairs of the numbers, which have been taken into account, are present. This is nothing else than finding an appropriate block design. In contrast to the identification of a single number, the identification of a single pair requires that on the final level one has $k_r = 2$. A specific realization of an optimum block design for $n = 6$, using $k_1 = 4, k_2 = 3, k_3 = 2$, is shown in Figure S10. The number of expected experiments, until an active pair is identified, can be easily determined. A naïve estimation for a given level reads $\left(\frac{1}{2}\right)(b_i + 1)$. For the present example this would correspond to an average of 2 attempts per level. This were true in case of a Steiner system. However, in general one has no Steiner system and thus due to the

finite number of multiple appearances of a pair the number of attempts can be slightly lower. For the specific example, on the first level its value is given by $\left(\frac{1}{15}\right)[6 \cdot 1 + 5 \cdot 2 + \ 4 \cdot 3] \approx$ 1.85. In analogy, one obtains average attempt values of 1.65 for the second level and exactly 2 for the final level. Neglecting these minor deviations an upper bound of the expected number B of experiments is given by $\left(\frac{1}{2}\right) B$Thus, we need again to search for a sequence of block designs such that B is a minimum.

This optimization problem has a well-defined solution, starting from the list of optimum block designs as given in the LA JOLLA COVERING REPOSITORY TABLES.[17] It can be solved recursively. Let us assume that for a given value of n one has identified the sequence $k_1, \ldots, k_r$ and the corresponding values $b_1, \ldots, b_r$, based on block design, such that B is a minimum. If this solution is available for all values of $n = 3, \ldots, n_{max}$, then it is possible to determine $B(n_{max} + 1)$ via $B(n_{max} + 1) = \min_{n_s \leq n_{max}} (B(n_s) + b(n = n_{max} + 1, k = n_s) + 1)$ where $b(n, k)$ denotes the minimum number of blocks of size k, obtained from an optimum block design for n numbers. These values are listed in the repository tables (under the reasonable assumption that for the small values of n and k indeed optimum block designs are listed).

Table S13: The optimum solution for minimizing B for different values of n (the k-values on the different levels, the corresponding b-values, and the resulting minimum value of B are listed). Furthermore, for the case of interval sectioning, the resulting number of the block sizes (k-values sectioning) and the resulting B values are listed.

n	***k*-values**	***b*-values**	**min. *B*/2**	***k*-values sectioning**	**$B_{sect}/2$**
**3**	2	3	2	2	2
**4**	3,2	3,3	4	3,2	4
**5**	3,2	4,3	4.5	4,3,2	6
**6**	4,3,2	3,3,3	6	4,3,2	6
**7**	5,3,2	3,4,3	6.5	5,4,3,2	8
**8**	5,3,2	4,4,3	7	6,4,3,2	8
**9**	6,4,3,2	3,3,3,3	7.5	6,4,3,2	8
**10**	7,5,3,2	3,3,4,3	8.5	7,5,4,3,2	10
**11**	8,5,3,2	3,4,4,3	9	8,7,5 ,4, 3	10
**12**	8,5,3,2	3,4,4,3	9	8,6,4,3,2	10

Note that in table S13 the number of blocks per level is always 3 or 4. On this basis we suggest the *interval sectioning algorithm* which is the equivalent to interval bisectioning for the case of identifying a single number. Starting from $k_0 = n$, we iteratively choose the block size of the next level as $k_i = \left\lceil \frac{2}{3} k_{i-1} \right\rceil$. The number of blocks is always 3. The specific form of the blocks is shown in table 14 for a few selected values of n. If n is a multiple of 3, we divide the interval into three parts and join always two of them to one block. For different values of n slight modifications are required, yielding a straightforward building principle.

Table S14: The specific form of the three blocks when performing interval sectioning for n values between 9 and 12.

n	***k***			
**9**	6	1,2,3,4,5,6	1,2,3,7,8,9	4,5,6,7,8,9
**10**	7	1,2,3,4,5,6,7	1,2,3,4,8,9,10	1,5,6,7,8,9,10
**11**	8	1,2,3,4,5,6,7,8	1,2,3,4,5,9,10,11	1,2,6,7,8,9,10
**12**	8	1,2,3,4,5,6,7,8	1,2,3,4,9,10,11,12	5,6,7,8,9,10,11,12

As shown in table 13, the values of B for the case of interval sectioning (here denoted B_{sect}) are very close to the optimum values. In practice, the difference is even smaller because, due to the slightly larger number of blocks, more pairs appear twice, which further increases the chance to find a pair already quite early (see discussion above).

We note that the general idea of interval sectioning can be directly translated to triplets (and even higher values of t). The general reduction formula reads $k_i = \left\lceil \frac{t}{t+1} k_{i-1} \right\rceil$ and the number of blocks per level is just t+1. The program, supplied on github, can deal with singlets, pairs, and triplets.

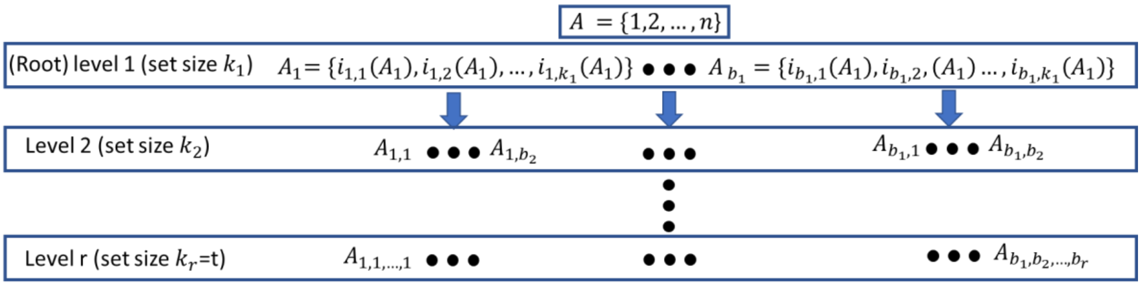

Figure S10: General scheme to successively generate sets of smaller sizes until level r is reached where the sets contain singlets (for $t = 1$), pairs (for $t = 2$), or triplets (for $t = 3$), respectively. The first level is denoted the root level. Key variables are the total number of compounds n, the set size k_r, the number of sets per level b_r and the value of t, specifying whether one searches for singlets, pairs, triplets.

The general procedure for the generation of blocks on different levels is visualized in Figure S10. A specific example for interval sectioning is shown in Figure S11, where the resulting tree-like structure is shown when identifying active pairs. For the example $n = 100$ one can easily check that there are 11 levels, i.e. $k_{11} = 2$. Thus, on average one would need slightly less than 22 experiments until one pair is identified. As already mentioned above, this is only an upper limit since the optimized algorithms takes into account that one does not have a perfect Steiner system, i.e. some pairs occur twice. The actual use of the interval sectioning algorithm reveals that on average less than 17 attempts are required. This has to be compared with the 4950/2 = 2375 experiments, when checking all pairs individually. Of course, this is equivalent to the acceleration via bisectioning when identifying a single number.

2.7.3 Theoretical Function

Working Principle

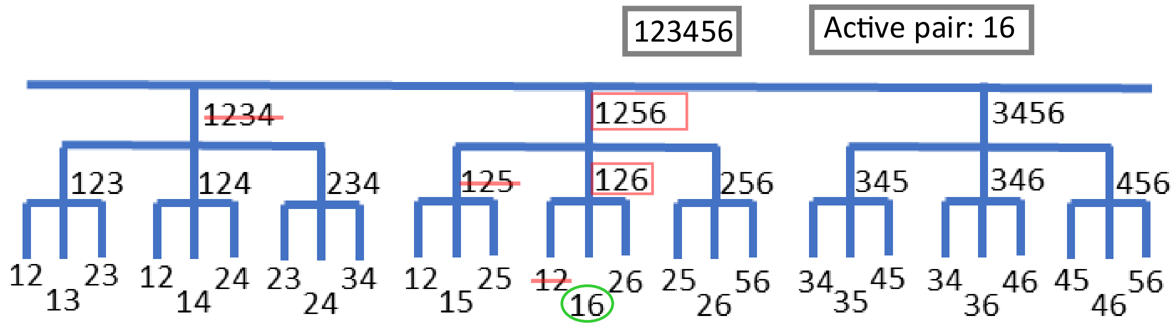

Figure S11: Example of a tree for $n = 6$, generated via the interval sectioning algorithm. This results in the presence of 3 levels. Furthermore, the search procedure is sketched for the case of a single active pair. One stops after its identification on level 3. One proceeds from left to right and only enters a lower levels if a set contains the active pair (for example in the case of (1,2,5,6)).

We start with the simple case that one wants to identify exactly one active pair. An example is shown in Figure S11. One starts on the first level and as soon as one finds a block with a positive response one continues one level further, thereby digging deeper into the sub-tree. At some stage one reaches the final level with block size two and can identify the active pair. For the example, shown in Figure S12 one would need 6 experiments to identify the pair (1,6). However, this simple example already shows that there is room for improvement. The fifth experiment, i.e. when checking the pair (1,2), was unnecessary because in the first as well as the third experiment it is was already clear that the pair (1,2) cannot be active.

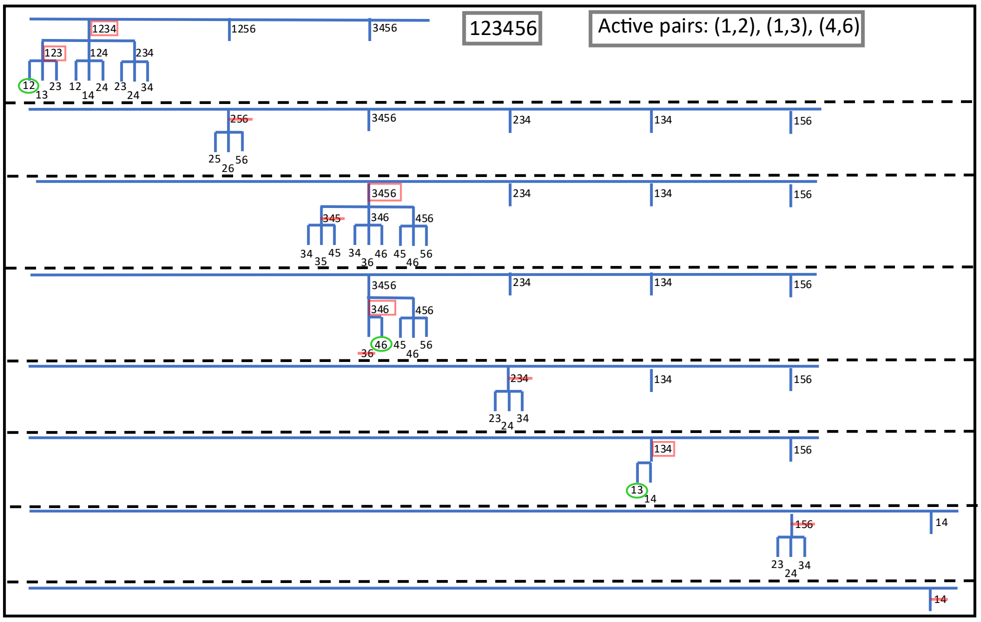

Figure S12. Analogous tree as in Figure S11. Here three active pairs are present, and one searches until all possibly active pairs have been checked. As outlined in the main text, this procedure is more involved. In our implementation this involves a growth process of the root level as well as several simplifying features in order to avoid experiments where the result is already known beforehand (negative or positive).

This motivates the final version of our interval sectioning algorithm. It has different highly desirable properties: (1) It works for an arbitrary number of active pairs. (2) It can find all active pairs. Of course, one can stop after having identified, e.g., two pairs (if present). (3) Via some straightforward features the efficiency is significantly increased. (4) It can be applied to a large range of n values.

The algorithm is described with the help of Figure 12. For this example, we have n=6 and there exist three active pairs, namely (1,2), (1,3), and (4,6). The level *root*=1 is denoted *root level* in the subsequent discussion. We have constructed this example such that all features of the algorithm come into play:

- Step 1: Check (1,2,3,4) => Positive result
- Feature 1: After a positive result continue on the next level
- Step 2: Check (1,2,3) => Positive result
- Step 3: Check (1,2) => Positive result => First pair found

- Feature 2: After finding a pair one always starts again with the next block on the root level rather than searching further in the lower regions of the tree.
- Feature 3: One must take into account that a block on the root level (here: (1,2,3,4)), which finally gave rise to the identification of an active pair (here: (1,2)), may also contain further active pairs. In order to be able to find all of them, the number of blocks in the root level is increased by two. They are generated from the block, just analyzed on the root level, by individually removing the individual elements of the active pair, yielding the new blocks (2,3,4) and (1,3,4). Furthermore, the procedure is applied to all other blocks on the root level which contain the active pair (1,2). As a consequence, the block (1,2,5,6) is substituted by the block (2,5,6) and additionally the block (1,5,6) is added on the root level.
- Step 4: Check (2,5,6) => Negative result
- Feature 4: When finding a negative result continue on the same level.
- Feature 5: By construction, not all three blocks can be negative when working on levels $r \geq 2$. If yes, an error message is given. Example: If (1,2,3,4) is positive it is not possible that (1,2,3), (1,2,4), and (2,3,4) are negative.
- Step 5: Check (3,4,5,6) => Positive results
- Step 6: Check (3,4,5) => Negative result
- Step 7: Check (3,4,6) => Positive result
- Feature 6: Before a block is evaluated, it is checked whether with the available information this block can be simplified (less elements) or even removed. For this purpose, all previous negative results are taken into account because they yield information about pairs which have to be negative. In the present example, the next block to be checked would be (3,4) However, since (3,4,5) was negative it is clear that (3,4) also has to be negative. Therefore, (3,4) is removed from the tree structure. More generally, if one has a block ($i_1,\ldots,i_k$), and from previous experiments it is known that, e.g., all pairs (i_1,i_2), (i_1,i_3), …,(i_1,i_k) are negative, then the element i_1 is removed from this block because its presence does not contain any new information.
- Step 8: Check (3,6) => Negative result
- Step 9: Check (4,6) => Positive result => Second pair found
- Step 10: Check (2,3,4) => Negative result
- Step 11: Check (1,3,4) => Positive result. Note that on the next level of the sub-tree below (1,3,4) the block (3,4) is removed because previously (2,3,4) turned out to be negative.

- Step 12: Check (1,3) => Positive result => Third pair found. As a consequence, the root level needs to be extended by the blocks (1,4) and (3,4), resulting from applying feature 3 to the block (1,3,4) on the root level. However, due to reasons, already mentioned above ((2,3,4) was negative), the block (3,4) does not need to be taken into account.
- Step 13: Check (1,5,6) => Negative result
- Step 14: Check (1,4) => Negative result
- Feature 7: When the last block of the root level is checked and no new blocks are added to the root level, the program is terminated.

In summary, the algorithm can be described as a smart tree search with the addition of the 7 features which render the exploration more efficient and, at the same time, guarantee that all active pairs can be found. For details on evaluation, comparison and practical application, please consult section 2.7.4. to 2.7.6.

We note that the same algorithm can be used for the identification of triplets. There is one natural modification. When, e.g., for the block (1,2,3,4,5) it is found that (1,2,4) is an active triplet, three additional blocks have to be studied on the root level, namely (1,2,3,5), (1,3,4,5), and (2,3,4,5).

Finally, we note that the presence of single quenchers does not hamper the possibility to find all active pairs. As is obvious from the workflow some additional experiments would have been performed (for example if 5 would be a single quencher the experiment would have, e.g., additionally suggested that (i,5) are active pairs for all *i*. These false-positive experiments would not exclude the identification of any other active pair.

Incorporation of the greedy algorithm

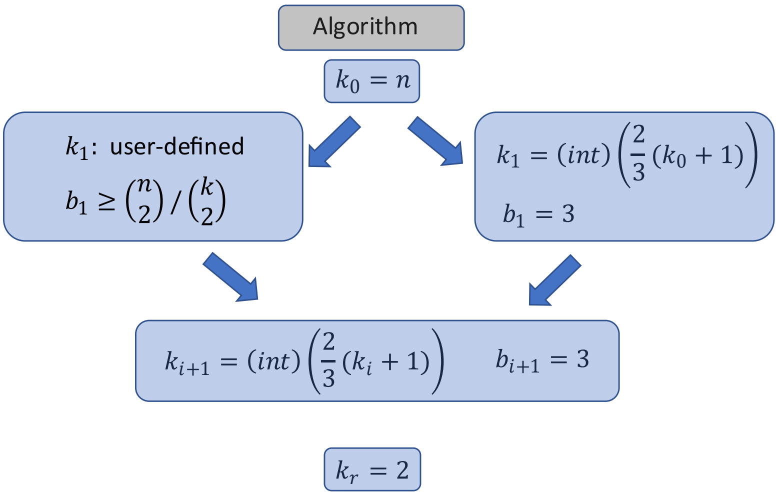

Figure S13: Sketch of the general algorithm, involving the possibility to use the greedy algorithm for the construction of the first level rather than the interval sectioning algorithm. The greedy algorithm is used if for experimental reasons the set size k_1 cannot be as large as the value, one would obtain from the interval sectioning algorithm.

The interval sectioning, described so far, continuously reduces the size of the blocks in relative small steps. However, for experimental reasons (e.g. background activity or high expected hit probabilities) one may need to request that any block size, studied experimentally, should not be larger than some value k_{max}. In our studies k_{max} was set to 10 to make sure additive effects are not superimposing cooperative effects. This can be easily achieved by the procedure as sketched in Fig. 13. Then the interval sectioning is only applied to all levels below the root level whereas the blocks of the root level are calculated via the greedy algorithm, using a block size of $k_1 = k_{max}$.

Setup Guide for different variables

As the developed program can become comparably memory intensive while large adjacent storage areas have to be allocated for corresponding matrices internal variables should be adjusted in case of large values for n or k. Please note that some variables need to be defined slightly larger (+1 or +2) than expected which is due to internal handing and termination rules. The following variables need to be set:

KMAX
Defines the maximal set size k the program has to deal with. If the greedy algorithm was used to determine the sets it shall be set to the set size $k_{greedy}+1$ from the "input_greedy.dat". In other cases it shall be set to $n+1$.

NMAX
Defines the maximal number of substrates the program has to store. Therefore it shall be set to $n+1$.

NMAX3
Defines the maximal number of substrates the program has to store in case of search for triplets If triplets are searched it should be set to $n+1$, otherwise it can be defined as 2.

PAIRMAX
Defines the maximal pairs the program has to expect. Therefore, it should be set to $P+2$.

LEVMAX
Defines the maximal levels the program will dig through. It can be derived from the maximal set size KMAX and should be set to:

$$LEVMAX = -\left\lceil \frac{ln(KMAX-1)}{ln\left(\frac{2}{3}\right)} \right\rceil + 1$$

For triplets the numerate has to be substituted by ln(3/4).

BMAX
BMAX is an internal parameter dependent of the maximal number of sets or blocks on the root level. As it cannot be calculated beforehand before initial runs it shall be set to the expected number of experiments b while iteratively raising it in cases of segmentation related errors (e.g. core dumped, no solution found, unrealistic numbers as solution, …). Please note that, in case of comparably small sizes for KMAX (k<20) the size of this variable can be chosen comparably large (e.g. 1000 to 10000). However, on most operating systems limitations are given by maximal stack sizes which are usually comparably small in case of Windows operating systems.

2.7.4 Statistical Evaluation

To evaluate the efficiency of the developed interval sectioning algorithm, we tested how many experiments are needed to identify all cooperative pairs in a space of n = 100 substrates. The

sets were generated using the greedy algorithm and fed into and searched using the interval sectioning approach as described above. For the generation of the sets, the number of repetitions of the greedy algorithm was set to r_{greedy} = 100. In order to test different scenarios (hit probabilities, set sizes, number of substrates) an evaluation mechanism was implemented (2.8.5), which can be used to specify how many pairs are present in the searched space. Unless otherwise described, each evaluation was performed 10 times and given values correspond to average results. The algorithm was set to find every pair within the given space i.e. follow the approach until every pair has been classified as cooperative or not. While the hit probability, i.e. the probability of a pair being active directly determines the absolute number of experiments needed to search the full combinatorial space the set size k_1 is mainly responsible for the searching efficiency. As an example, in the case of a relatively high hit probability of h = 2.0% a minimum for the overall number of experiments lies in between a set size of 5 and 15, meaning that relatively small sets would make the search for pairs more efficient. This minimum appears to be the global minimum and exists for every h (Figure S14).

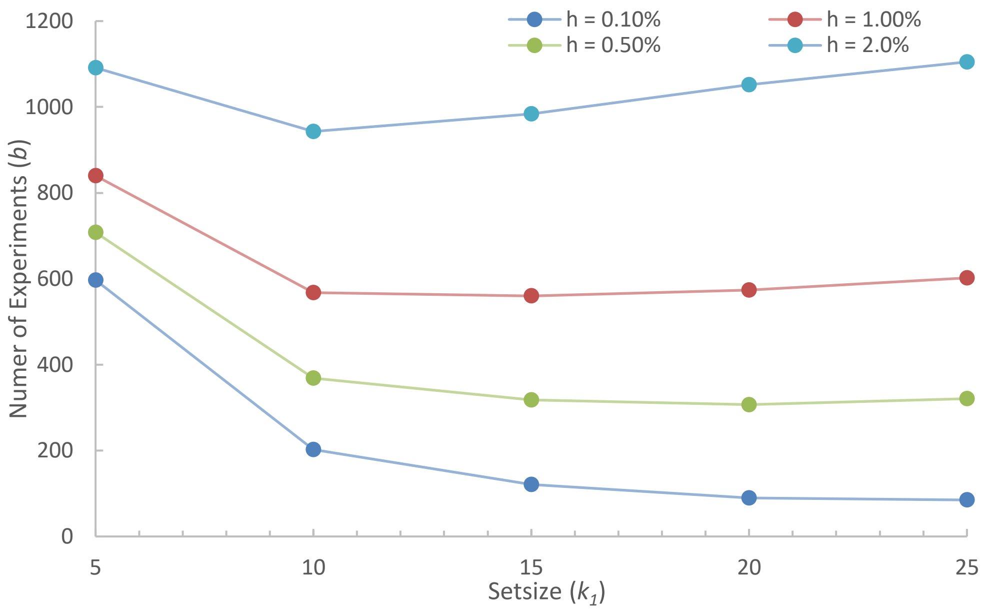

Figure S14: Number of experiments needed to identify all active pair in a space of n = 100 compounds in dependence of the hit probability h and the set size k_1.

In case of extremely high hit probabilities (~100%) the optimal set size is two since every non-pairwise test would be positive thereby increasing the overall number of experiments. In all other cases a distinct minimum exists for $k_1 < n$ as long as at least one active pair is within the search space. This can be explained by the fact, that a set with the size $k_1 = n$ would always be positive and therefore redundant. As expected, the optimal set size shifts towards larger set sizes

for a higher hit probabilities, since numerous pairs can be excluded in one experiment (Figure S14**)**. Even though the overall number of experiments raises in for higher hit probabilities, the efficiency as determined by the number of experiments needed per found pair is higher in these cases as it is lower for higher hit probabilities (Figure S15). In addition, it can be observed, that the relevance of the chosen set size decreases towards higher hit probabilities as variations in the number of experiments needed to identify one pair are lower in these cases within the tested space (k_1 = 5, 10, 15, 20, 25; h = 0.1%, 0.5%, 1.0%, 2.0%)

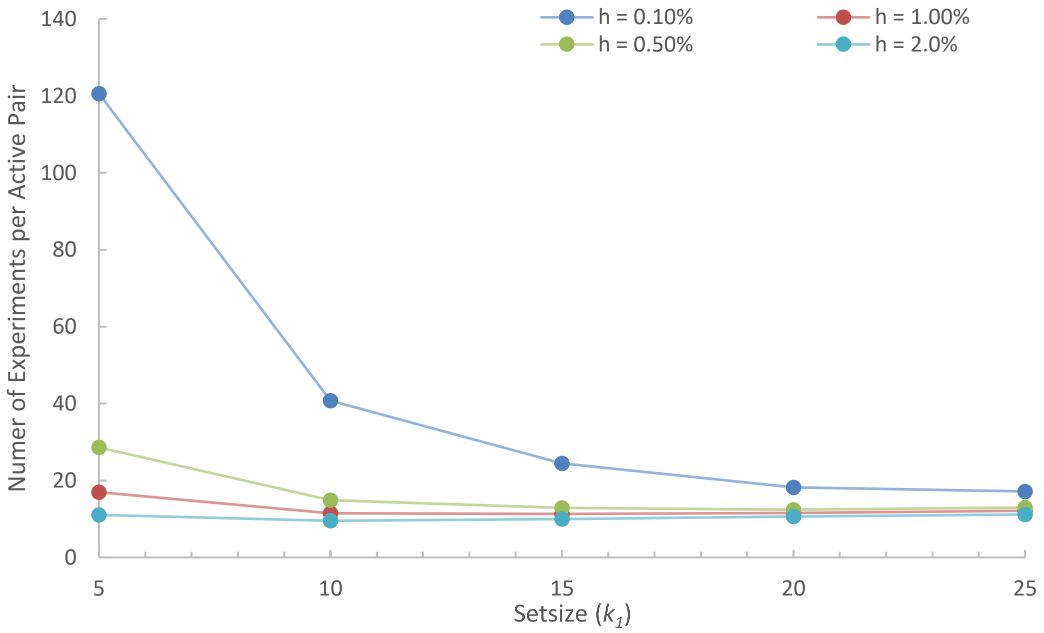

Figure S15: Number of experiments per identified/active pair in a space of n = 100 compounds in dependence of the hit probability h and the set size k_1.

As it is expected that the number of substrates tested n i.e. the size of the searched space does not influence the efficiency of the developed method different values were tested (n = 100, 200, 300, 400, 500) while keeping the hit probability identical (Table S15). Thereby it can be shown that the efficiency does indeed not decrease towards larger spaces while even increasing insignificantly. This means, that the overall efficiency of the developed approach is only limited by the underlaying hit probability and experimental restrains to set size. Using the developed interval sectioning tool a highly efficient set size and good block design can be found therefore minimizing the experimental efforts.

Table S15: Number of experiments and number of experiments per identified/active pair for $k_1 = 10$ and $h = 0.5\%$ in dependence of number of compounds n.

n	**100**	**200**	**300**	**400**	**500**
**Nr of experiments**	366.4	1434.9	3187.9	5640.0	8762.6
**Nr of experiments/ pair**	14.8	14.4	14.2	14.1	14.0

2.7.5 Practical Application

General Information

The developed interval sectioning software uses "input_iterations.dat" as configuration file (Table S16), while printing results in the "output_iterations.dat" text file. In addition, it is able to read block designs from the output of the greedy algorithm ("data_greedy.dat") if demanded by the users input. Thereby two mods are available for the initial set construction, determined by the flag as set in line three of the input file. If this flag is set on false (0) the algorithm will not use the "data_greedy.dat" file but constructs sets in a strict fashion as described above (2.8.3). If the flag is set on true "data_greedy.dat" will be used as input to construct initial sets as determined before. Three modes are available for the dimensionality of search, determined by the flag in line one. This variable dictated whether the program shall search for individual compounds by simple group testing and interval halving, pairs or triplets interval sectioning (2.8.3). The variable in line two is the number of substrates (n) for which singles, pairs or triplets are searched. In case of the conducted screening it can be set on 100 to read the "data_greedy.dat" file while subsequentially finding pairs in every of the constructed sets or could be set to 10 to find pairs in an individual set. Finally, the variable in line four determines the number of pairs which the user wants to find. In case of $P = 1$ the algorithm would stop proposing and constructing sets after one pairs has been identified. If the user is aiming for a full investigation of the search space it can be set to the theoretical upper limit $\binom{n}{2}$ to continue searching until all pairs have been classified as active or not active by the program. Please note that internal variables may have to be adjusted according to the chosen inputs (2.7.4).

Table S16: Explanation of the input file (input_iterations.dat) for the finding of pairs from the in prior generated set design.

Line	Variable	Explanation	Example (this screening)
1	d	Dimensionality of the search (single, pairs, triplets)	2
2	n	Number of Substrates (in the tested set)	100
3	*read_greedy*	Flag for reading initial sets from the "data_greedy.dat" file. 0 = False, 1 = True	1
4	P	Number of identified active pairs before the algorithm stops searching	4950

At the start of the program two possible modes can be selected. The first (Input: 0) is the interactive mode whereby the software proposes sets which need to be tested by the user. Subsequently the user inputs the outcome of the experiment as Boolean (0: no quenching or activity, 1: quenching or activity). Afterward, the procedure is repeated until *P* active pairs or all active pairs have been confirmed. The tested sets, inputted values and results including possible inconsistencies (e.g. as given by individual quenchers) can be found in the "output_iterations.dat" file.

To evaluate the expected number of experiments in case of a specific set design as given by the greedy algorithm for certain settings of n and k a benchmarking subroutine is included in the program. This routine had been used to obtain, e.g., the results about the efficiency of the algorithm, discussed above. This possibility can be accessed by entering True (1) after the start of the program. Subsequentially the number of pairs which should be simulated can be typed in. In this case two options are given. Either the user can specify the pairs which should be simulated (0) or the software can pick them randomly (1). In the first case the pair can be entered as tuple of two integers (e.g. 31 4). In the later the number of iterations can be determined meaning that the procedure of random pair selection and evaluation is repeated multiple times with different seeds (Figure S16).

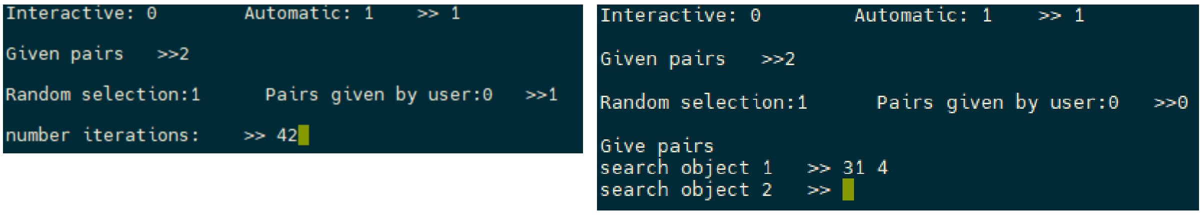

Figure S16: Screenshots of two the inputs and menus at the start of the pair finding software.

Practical Guide

To search for cooperative interactions or dual activities within a defined search space by the means of our software the following procedure is recommended (for details on specific steps please consult the corresponding sections individually). Please note that the instruction is written as general as possible to be valid for various screens for cooperativity.

1. Estimate the hit probability within the search space

If the probability for a hit is known or can be estimated reasonably the efficiency of the block design and following pair finding can be maximised. In case of an unknown but presumably

low hit probability, the initial set size should be designed rather small to efficiently deal with unforeseen effects.

2. Individual testing of compounds

Compounds need to be selected for the screening based on their individual activity. Thereby, it is recommended to keep background activity as low as possible, especially if the measuring methods comes with comparably high errors. If background activity is additive, like in case of luminescence quenching it is recommended to avoid any background activity at all.

3. Determine thresholds based on the background activity

Based on the previously determined background activity an analysis can be carried out to determine quantiles of the distribution function for the highest expectable values. If the method for the measurement of the demanded activity has a low error, comparably to the average absolute activity of the individual compounds it is also possible to sum up highest background activities to determine set sizes and thresholds. In this case even a quantification of the cooperative effects strength could be possible.

4. Determination of the highest experimentally possible pool/set size

The set size should be selected to make a distinction between additivity and cooperativity possible. In our case this meant to choose the set size to have a maximum of 60% background activity (compared to full quenching as 100%). Therefore, it is done to render the thresholds in dependence of the set size.

5. Optimization of the set-size

Dependent of the approximated hit probability the optimal set size can be determined as described above. As the efficiency is independent of n, automated analysis can be carried out for small $n > k_1$. Therefore, the greedy algorithm can be used for the determination of initial sets while subsequentially using the benchmarking subroutine of the interval sectioning software. By checking the number of needed experiments in dependency of k_1, for the previously determined hit probability the optimum can be found. Note that the hit probability corresponds to a number of pairs which can then be inputted into the benchmarking system and that non-statistically distributed cooperative effects can render the usage of smaller set sizes more efficient.

6. Decision for a full or partially search

As the constructed software allows for the search of a pre-determined number of pairs, it has to be decided if the full space needs to be searched or if a specific number of pairs is sufficient, e.g. to find hits for a subsequent second screen or a proof of principle or if the full combinatorial space needs to be searched. For the first case, the desired number or pairs can be specified in the "input_iterations.dat" file. To be on the safe side when finding all parameters, the value of P can be chosen as theoretical maximum value of active pairs which is given by $\binom{n}{2}$ after *n* has been selected.

7. Determination of n

In case of a partial search, the number of *n* is not relevant and can be chosen to include the most promising compounds within the search space. In case of a full search the number of *n* can be determined by the approximated number of experiments to conduct. Therefore, the number of experiments can simply be calculated by the expected numbers of active pairs, as determined by the hit probability and screening efficiency (number of experiments/pair found).

8. Conduction of the cooperative screen

Finally, the screening sets can be constructed by applying the adapted greedy algorithm with *k* and *n* as input settings.[16] If $n < 100$ the La Jolla Combinatorics Repository can be used to find differently optimized block designs.[17] If no limitation is given for the set size (low experimental error and hit rate) the interval sectioning algorithm can be applied directly. To reduce the number of experiments, it is recommended to use a comparably high number of repetitions r_{greedy} (i.e. 1000). After set construction, the interval sectioning algorithm can be applied inputting the determined block design (sets). Using the interactive program or implementing it into a robotic approach, if needed, pairs can be found even if triplets or individually active compounds are present within the mixtures. If this occurs, the program will give information about an inconsistency. If it is demanded to investigate this the software can used again on the initial sets with first with $d = 1$ to searching for individually active compounds followed by applying $d = 3$ to find active triplets.

2.7.6 Experimental Validation

Table S17: Structures of all compounds within set 118 with old and new numberings.

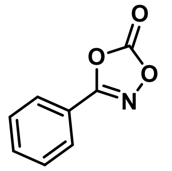	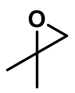	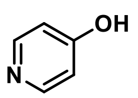	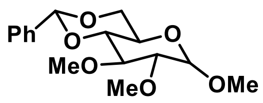	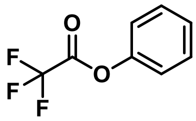
**S5**<br>**1**	**S9**<br>**2**	**S15**<br>**3**	**S34**<br>**4**	**S48**<br>**5**
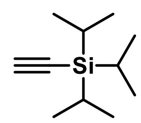	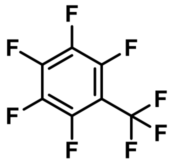	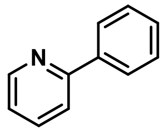	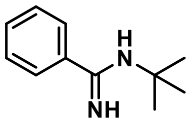	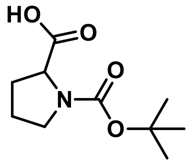
**S60**<br>**6**	**S66**<br>**7**	**S84**<br>**8**	**S88**<br>**9**	**S96**<br>**10**

As the initially developed and tested one-step deconvolution procedure posed several challenges and inefficiencies, especially in the case of the copresence of multiple active pairs in one mixture the interval sectioning algorithm was tested experimentally on one of these problematic sets. As especially challenging pair finding task, set **118** (**S5**, **S9**, **S15**, **S34**, **S48**, **S60**, **S66**, **S84**, **S88**, **S96**) was selected since three pairs have been found being present while exhibiting cooperative effects. In opposite to the full deconvolution of the whole screen (i.e. all sets) whereby all sets were giving by the greedy algorithm and solved by subsequential interval sectioning (2.8.5, 2.8.7) in this approach the set was solved individually as proof of concept. Therefore, all substrates were renumerated with numbers reaching from one to ten (e.g. **S5** ->**1**, **S9** -> **2**, **S15** -> **3**, …)(Table S17). Two experiments were carried out, one searing for only one active pair and another one searching for all active pairs within the set. The following input settings were used: $d = 2$, $n = 10$, *read_gready* = 0, $P = 1$ or 45. For the experimental setup stock solutions of [Ir(dF(CF$_3$)ppy)$_2$(dtbpy)][PF$_6$] (**PC1**) as photocatalyst (0.1 mM) and the substrates (250 mM) were prepared in degassed MeCN. To ensure a good reproducibility and low errors during the testing of the sets as given by the algorithm, the fluorescence of [Ir(dF(CF$_3$)ppy)$_2$(dtbpy)][PF$_6$] (**PC1**) was probed multiple times until intensities varied around a fixed value. In doing so, 100 µL of the catalyst stock solution was pipetted into a cuvette and diluted with 900 µL of MeCN. For testing the sets 100 µL of the catalyst stock solution and 100

µL of each substrates stock solution were pipetted into the cuvette and diluted with MeCN to a total volume of 1000 µL. The fluorescence was measured as before. Thresholds differentiating between quenching or not quenching were used adaptive in dependence of the sub set size k_{sub} for every experiments whereby the upper limit was used to reduce the chance for conflicts within the deconvolution process (2.2.2). As mentioned before conflicts or inconsistencies are tolerated by the program but raise the experimental effort (i.e. the number of overall experiments).

Table S18: Subsets as proposed by the interval sectioning algorithm for the finding of one pair (P = 1) within set 118. Results are given as quenching fraction. Thresholds are given in dependence of the set size k.

Experiment	Subset	Quenching(%)	Threshold	Input
**1**	**1 2 3 4 5 6 7**	13	50	0
**2**	**1 2 3 4 8 9 10**	71	50	1
**3**	**1 2 3 4 8**	25	40	0
**4**	**1 2 3 9 10**	67	40	1
**5**	**1 2 3 9**	80	35	1
**6**	**1 2 9**	77	30	1
**7**	**1 9**	79	25	1

In case of P = 1, i.e. the search for one pair within the set (**1**,**9**) (S5, S88) was found within 7 attempts. Overall, previously determined thresholds worked out well while no unclear or borderline case occurred (Table S18). In case of P = 45, i.e. the search for all pairs within the given set all previously deconvoluted pairs ((**1**,**9**) (S5, S88), (**3**,**9**) (S15, S88), (**5**,**9**) (S48, S88)) where found without any issues and with 21 experiments. Again, no measured quenching fraction was close to the threshold proofing the concept as robust. Negative quenching fractions for the final values can be explained by slightly lower oxygen values within the utilized wet-box. Nonetheless, pair (**3**,**9**) (S15, S88) has been identified clearly (Table S19). Output files show no sign for inconsistencies meaning that a clear assignment was made for every pair (Table S20). With this test it has been demonstrated, that the developed algorithm can easily be applied for experimental testing, while pairs can be found even in cases of the co-presence of multiple pairs. Since no human intervention and redesign was needed this would allow for the application of fully automated screening approaches.

Table S19: Subsets as proposed by the interval sectioning algorithm for the finding of all pairs ($P = 45$) within set 118. Results are given as quenching fraction. Thresholds are given in dependence of the set size k.

Experiment	Subset	Quenching(%)	Threshold	Input
**1**	**1 2 3 4 5 6 7**	4	50	0
**2**	**1 2 3 4 8 9 10**	71	50	1
**3**	**1 2 3 4 8**	17	40	0
**4**	**1 2 3 9 10**	68	40	1
**5**	**1 2 3 9**	70	35	1
**6**	**1 2 9**	81	30	1
**7**	**1 9**	81	25	1
**8**	**5 6 7 8 9 10**	66	45	1
**9**	**5 6 7 8**	−5	35	0
**10**	**5 6 9 10**	72	35	1
**11**	**5 6 9**	37	30	1
**12**	**5 9**	39	25	1
**13**	**2 3 4 8 9 10**	98	45	1
**14**	**2 3 9 10**	97	35	1
**15**	**2 3 9**	97	30	1
**16**	**2 9**	−10	25	0
**17**	**3 9**	92	25	1
**18**	**1 2 3 4 8 10**	−8	45	0
**19**	**5 6 7 10**	−11	35	0
**20**	**6 7 8 9 10**	−5	40	0
**21**	**4 9**	−6	25	0

Table S20: Screenshots of the Output_iteration.dat files for the finding of one pair (P = 1)(left) and all pairs (P = 45)(right).

```
0       1   2   3   4   5   6   7
1       1   2   3   4   8   9   10
0       1   2   3   4   8
1       1   2   3   9   10
1       1   2   3   9
1       1   2   9
1       1   9
attempts:
7
found solutions:
1   9
```

```
0       1   2   3   4   5   6   7
1       1   2   3   4   8   9   10
0       1   2   3   4   8
1       1   2   3   9   10
1       1   2   3   9
1       1   2   9
1       1   9
1       5   6   7   8   9   10
0       5   6   7   8
1       5   6   9   10
1       5   6   9
1       5   9
1       2   3   4   8   9   10
1       2   3   9   10
1       2   3   9
0       2   9
1       3   9
0       1   2   3   4   8   10
0       5   6   7   10
0       6   7   8   9   10
0       4   9
attempts:
21
found solutions:
1   9
5   9
3   9
```

2.7.7 Benchmark on the Conducted Screening

To test the reliability and efficiency of the interval sectioning algorithm, also in comparison to the previously utilized de-/convolutive strategy benchmark experiments were carried out testing and calculating the pair finding efficiency of both algorithms. In case of the interval sectioning algorithm initial sets were constructed applying the greedy algorithm (n = 100, k = 10, r_{greedy} = 100) whereby a design of 158 sets was found. This set design was used as input for the interval sectioning algorithm (d = 2, n = 100, *read_gready* = 1, P = 1, 5, 10, 15, 17, 20 or 25) whereby the automatic benchmark routine with manual pair input (2.7.5) was used. All 17 pairs which have been found within the initial screening (2.5) and the statistical evaluation (2.6) were used as input and goal to find. For the sake of clarity, it has to be mentioned, that more pairs are expected to be present within the explored space which might have been overseen by the previously utilized deconvolution. However, it can be estimated that these pairs will be found

in an interactive approach which would even raise its efficiency. Therefore, the given results for the number of experiments per found pair can be seen as lower limit approximation.

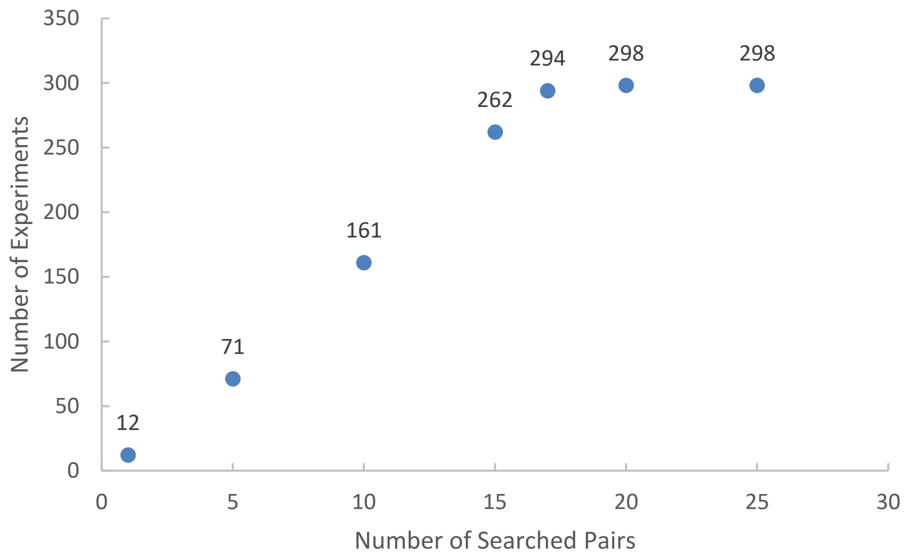

Figure S16: Theoretical number of experiments needed for the conducted screen (n = 100, k = 10, 17 active pairs) in dependence of the number of searched pairs (P). For P >17 the algorithm continued until all pairs were assigned.

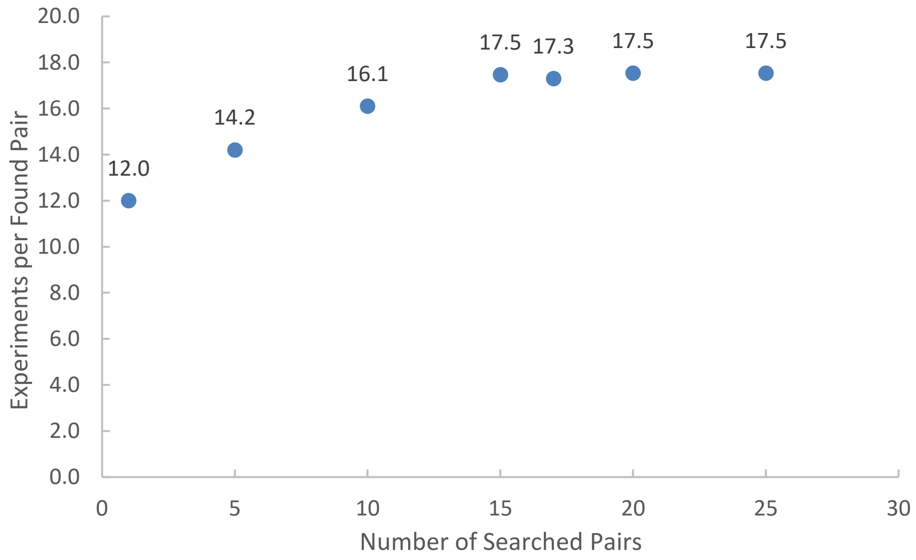

Figure S17: Theoretical number of experiments needed per found pair for the conducted screen (n = 100, k = 10, 17 active pairs) in dependence of the number of searched pairs (P). For P >17 the algorithm continued until all pairs were assigned.

It has been found that, while the number of experiments needed to find a certain number of pairs raises continuously if searched, there is only a slight difference between searching for the

exact number of pairs (P = 17, 294 experiments) and searching for all pairs (P > 17, 298 experiments)(Figure S16). Trivially it is irrelevant if searched for slightly more or even all (P = 4950) pairs. Thereby the efficiency, is slightly higher if only a few pairs are searched for which can be explained by the immediate interruption of the program, not following any additional branches on lower levels (Figure S17). Overall, 298 experiments are needed to find all present pairs by this strategy. However, as compound **S51** posed a major challenge in the conducted screen due to non-cooperative quenching a second benchmark was performed giving positive feedback to the program (1 as input) if this compound was present in a set with $k > 2$. Even though this is not a fair comparison since some sets were below the threshold even if **S51** was present, is renders the worst case scenario and thereby gives a upper limit approximation for the number of experiments needed. In this benchmark with P > 17, 393 experiments were needed for finding all pairs even though multiple inconsistencies occurred due to the positive response to **S51**. For the initially developed de-/convolutive screening approach 159 experiments were needed for the testing of the initial deconvoluted sets, 200 experiments were needed for the first deconvolution sets (25 hit sets x 8 deconvolution sets), 60 experiments were needed for controls (4 controls for every pair) while 85 experiments were taken into account for additional testing for the deconvolution. As this number is not fully known to the authors and strongly depends on the depth of analysis. It was approximated that ever set which was not deconvolute with the initial deconvolution set required 5 additional experimental trials (Figure S18) before the solution was found.

Overall, the de-/convolutional approach could be found having a hit rate of 2.9% (33.6 experiments per pair) while the interval sectioning approach was benchmarked to raise the efficiency giving a hit rate of 4.3 to 5.7% (23.1 to 17.5 experiments per pair). In comparison a naïve testing of pairs would lead to a hit rate of >0.3% (<291.2 experiments pair pair) (2.9). However, it has to be mentioned that efficiency for the later approaches will most likely be higher as more pairs are expected to be found (2.6).

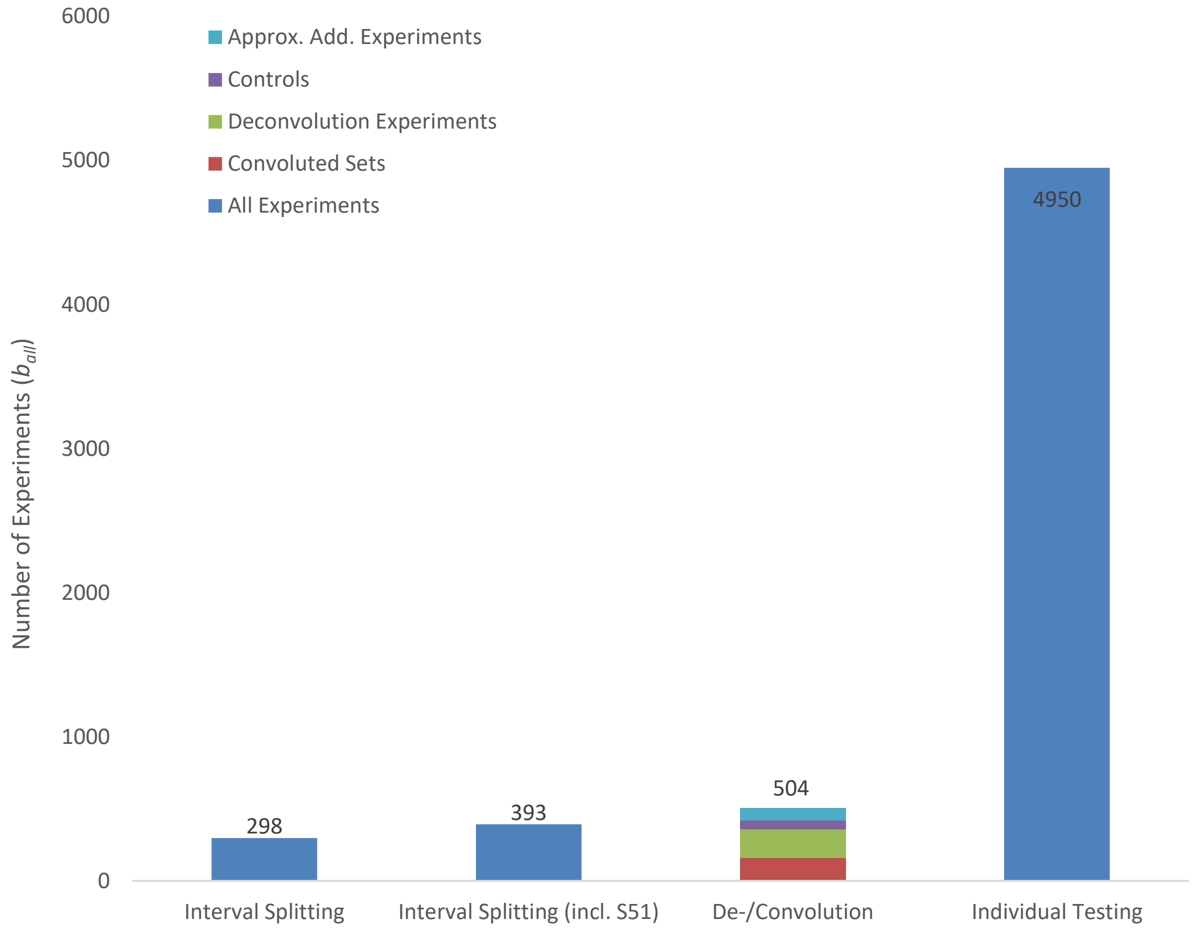

Figure S18. Approximated number of experiments needed to search the given dual space (n = 100, k = 10, 17 active pairs). Active pairs were chosen as found during the conducted screen. The interval sectioning algorithm was configured to continue until all pairs are assigned. In one case quenching substrate **S51** was included into the algorithm evaluation by marking all experiments with **S51** as "quenching" as long as more than two substrates were present in a set.

2.8 Calculation and Comparison of Hit Rates

2.8.1 General Formular

To calculate the hit rate ($ratio_{hits}$) for our screening approach, we divided the number of hits ($count_{hits}$) by the number of experiments performed throughout the respective screening strategies. All experiments needed for the convolution, deconvolution as well as controls and approximated additional experiments are taken into account.

$$ratio_{hits} = \frac{count_{hits}}{count_{experiments}}$$

In the following, a unique and in prior unknown finding, will be considered as a hit, if the designed screening approach aimed to find this target (cooperative interaction). The number of all of these unique hits is the number of hits ($count_{hit}$).

2.8.2 Hit rate of the Convoluted Cooperativity Screening (Initial approach)

Target:	Binary combinations of substrates showing cooperative interaction
$count_{experiments}$:	159 + 200 + 60 + approx. 85 = 504
$count_{hits}$:	15
$ratio_{hits}$ *(hit rate):*	**2.9%**

2.8.3 Hit rate of the Interval Halving Approach

The hit rate for an interval halving approach was approximated using the benchmarking subroutine of the developed software while taking all pairs into account which have been identified in the context of this project.

Target:	Binary combinations of substrates showing cooperative interaction
$count_{experiments}$:	298 to 393 (in case that only 17 pairs are present)
$count_{hits}$:	17
$ratio_{hits}$ *(hit rate):*	**> 4.3%**

2.9.4 Hit rate of a Theoretical Unconvoluted Cooperativity Screening

To calculate the hit rate of a theoretical unconvoluted cooperativity screen the approximation has to be made, that there are no other hits within the searched space other than the 17 pairs found. Since hits were overseen by the conducted convolutional screening approach (2.9), only a lower limit can be given for the hit rate of a unconvoluted screen. By using recall as determined by testing all pairs of 20 compounds (2.9) the hit rate can be approximated to be 0.90%.

Target: Binary combinations of substrates showing cooperative interaction

$count_{experiments}$: 4950

$count_{hits}$: 17

$ratio_{hits}$ *(hit rate):* **>0.34%** (0.90%)

3. Origin of the Cooperative Luminescence Quenching

3.1 Theoretical Background to Investigate the Origin of Cooperativity

In order to investigate the origin of cooperativity of all identified pairs (Table S22), qualitative analytical studies were carried out. Therefore, all individual substances and dual combinations of those were examined using different analytical techniques. Gas Chromatography–Mass Spectrometry (GC-MS, 3.2) as well as Thin Layer Chromatography (TLC, 3.3) were used to identify whether two substances underwent a chemical reaction, thereby forming new chemical bond(s) and molecules. To get detailed information about the new formed species, Nuclear Magnetic Resonance (NMR, 3.4) and Electrospray Ionization High Resolution Mass spectrometry (ESI, 3.5) investigations were performed. To identify other cooperative interactions (acid-base and non-covalent interactions), NMR results were mainly taken into account. Stock solutions of all substances were prepared in d_3-MeCN (0.20 M).

Individual Compounds

Table S21: Individual compounds identified to show cooperative effects.

N°	Molecule	N°	Molecule	N°	Molecule
**S4**	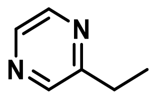	**S17**	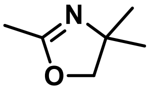	**S48**	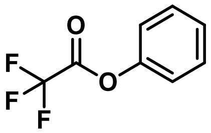
**S5**	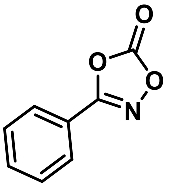	**S19**	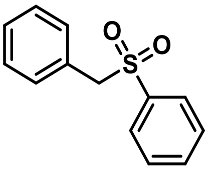	**S75**	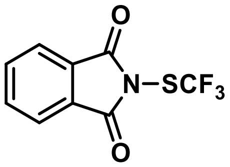
**S10**	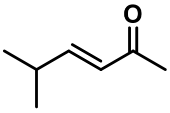	**S22**	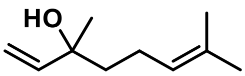	**S88**	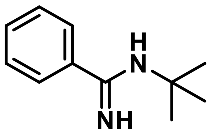
**S11**	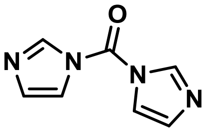	**S32**	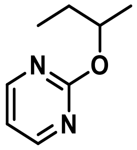	**S95**	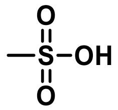
**S15**	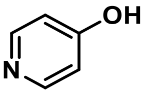	**S38**	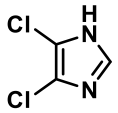		

Cooperative Pairs

Table S22: All cooperative pairs found in the convoluted screening.

N°	Pair	N°	Pair
**C1**	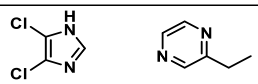 S38 S4	**C9**	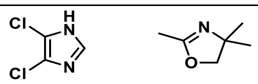 S38 S17
**C2**	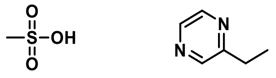 S95 S4	**C10**	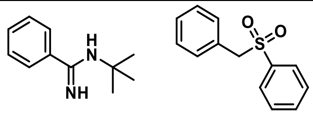 S88 S19
**C3**	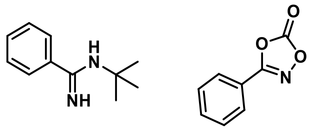 S88 S5	**C11**	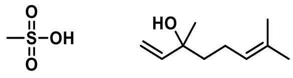 S95 S22
**C4**	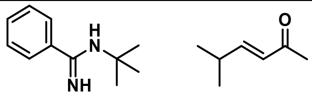 S88 S10	**C12**	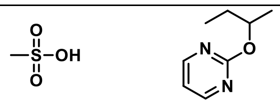 S95 S32
**C5**	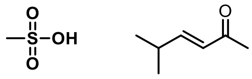 S95 S10	**C13**	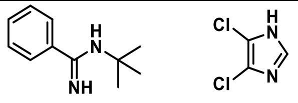 S88 S38
**C6**	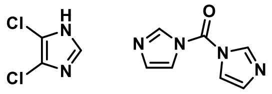 S38 S11	**C14**	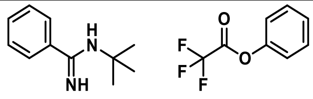 S88 S48
**C7**	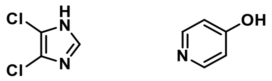 S38 S15	**C15**	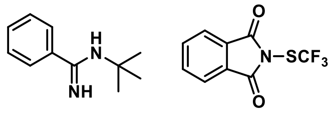 S88 S75
**C8**	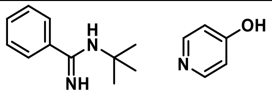 S88 S15		

3.2 Gas Chromatography–Mass Spectrometry Studies

3.2.1 Analytical Details

To prepare reference samples for every individual compound, 20 µL of the stock solution (0.2 M) were transferred to a glass vial (2.0 mL) and 1.0 mL of MeCN was added. To prepare samples of the cooperative pairs, 10 µL of both stock solutions were transferred to a glass-vial (2.0 mL) and 1.0 mL of MeCN was added. All samples were mixed and measured within 24 h after preparation. A solvent delay of 4.0 min was chosen cutting of low boiling compounds.

3.2.2 Results

Chromatograms and MS-spectra were analyzed using *Agilent* ChemStation 2.2.1431. To obtain less noisy spectra, a background subtraction was performed. Measured spectra of every peak are analyzed using the fragmentation pattern and the NIST07-database for comparison. As result of their low boiling points, compounds S17 and S95 are not visible in the recorded chromatogram, while compound S15 is not visible due to its high boiling point.

Chromatogramms and Mass-Spectra

S4 Chromatogram

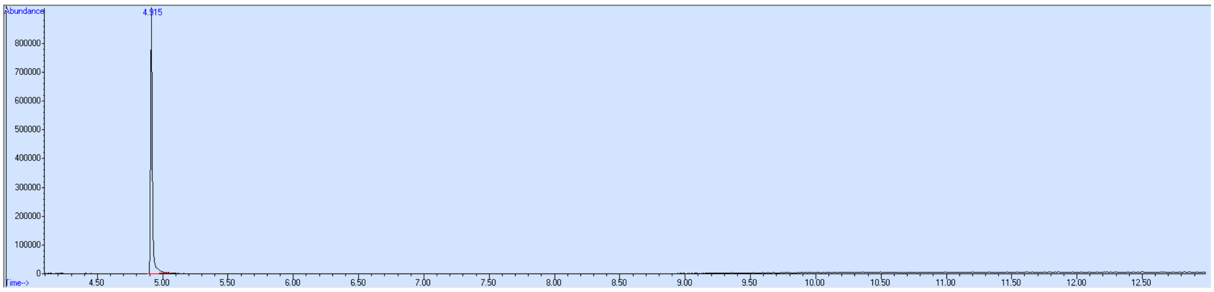

S4 Mass Spectrum @ 4.92 min

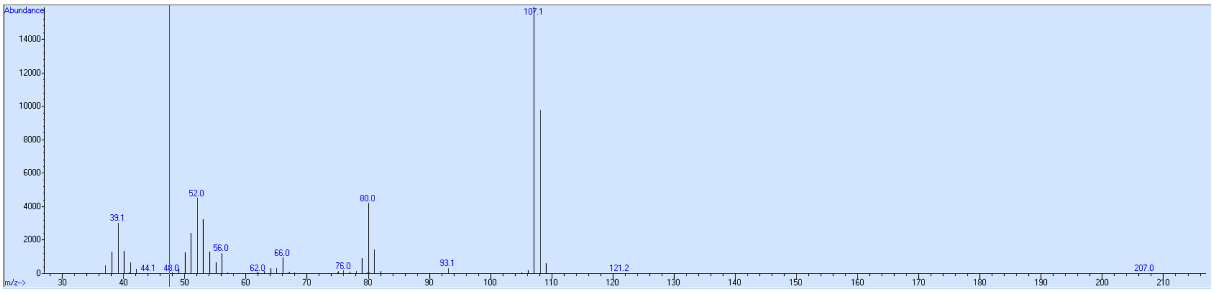

S5 Chromatogram

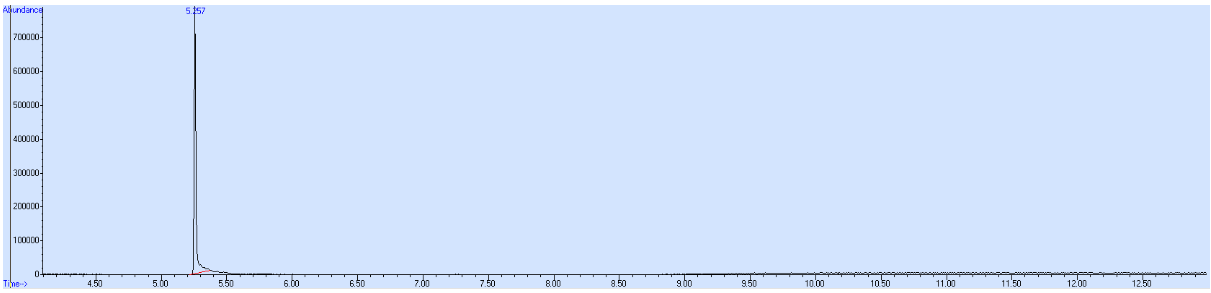

S5 Mass Spectrum @ 5.26 min

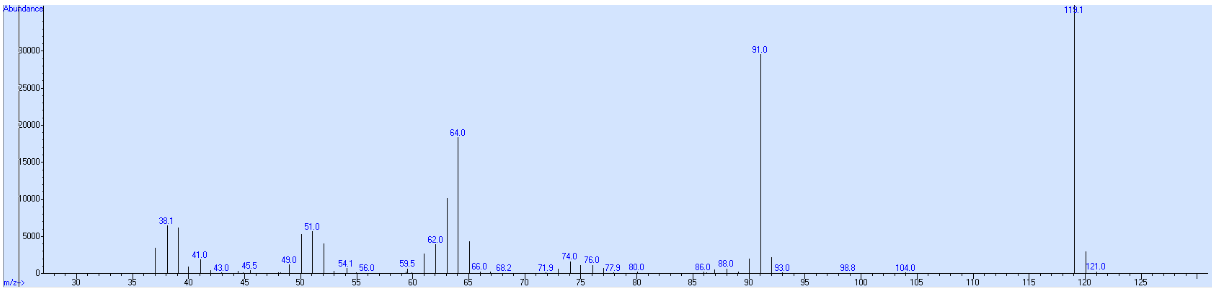

S10 Chromatogram

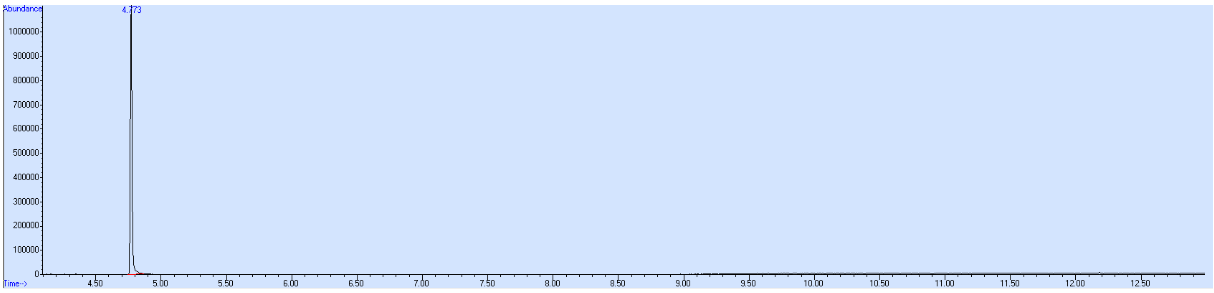

S10 Mass Spectrum @ 4.77 min

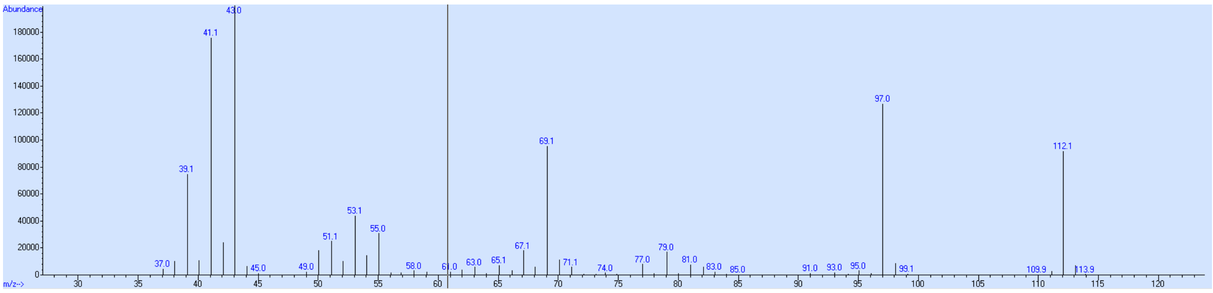

S11 Chromatogram

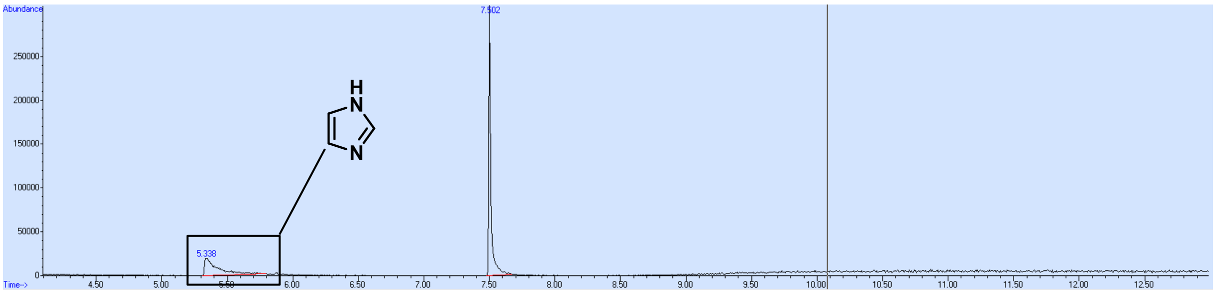

S11 Mass Spectrum @ 5.34 min

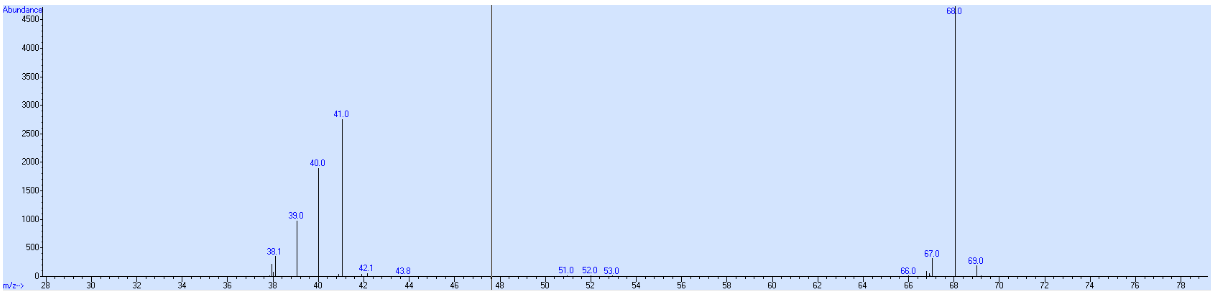

S11 Mass Spectrum @ 7.50 min

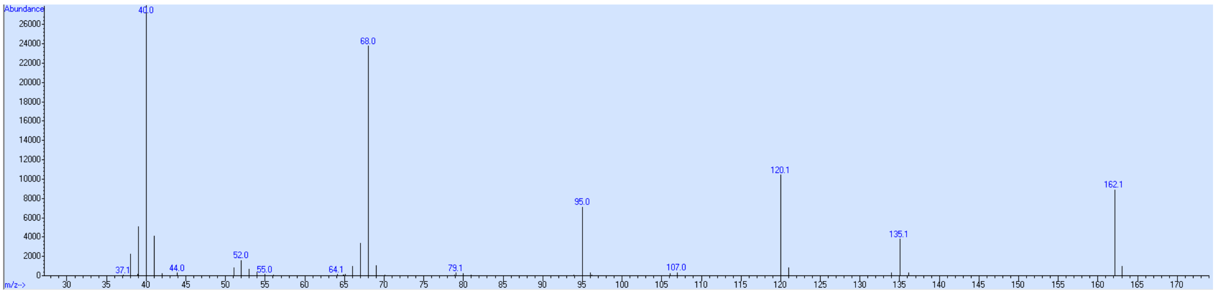

S15 Chromatogram

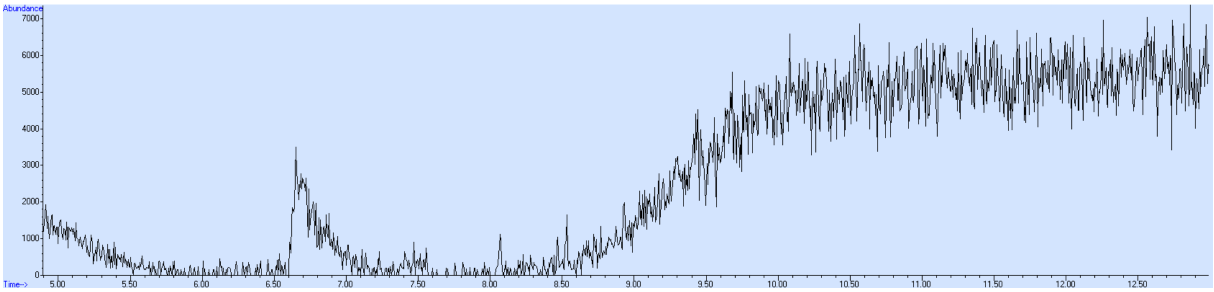

S17 Chromatogram

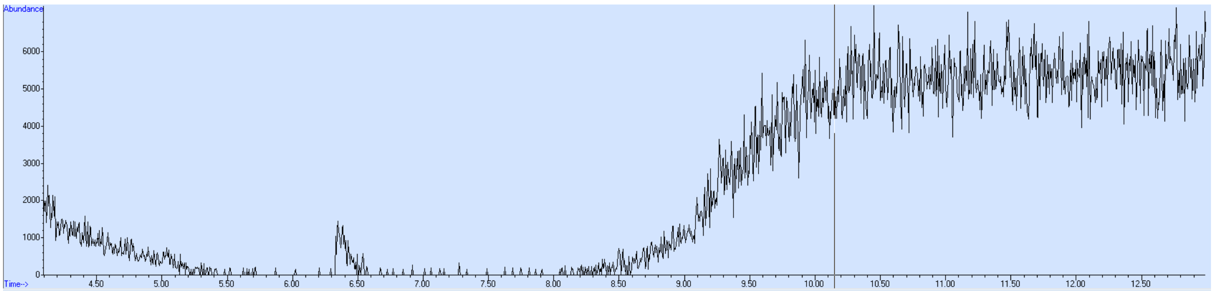

S19 Chromatogram

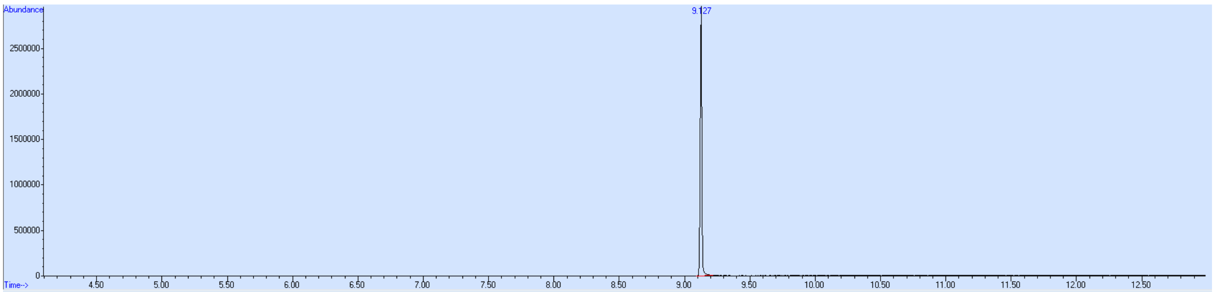

S19 Mass Spectrum @ 9.13 min

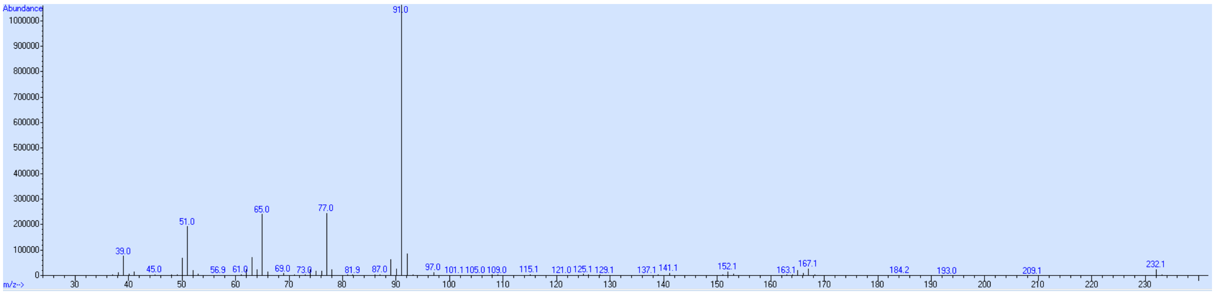

S22 Chromatogram

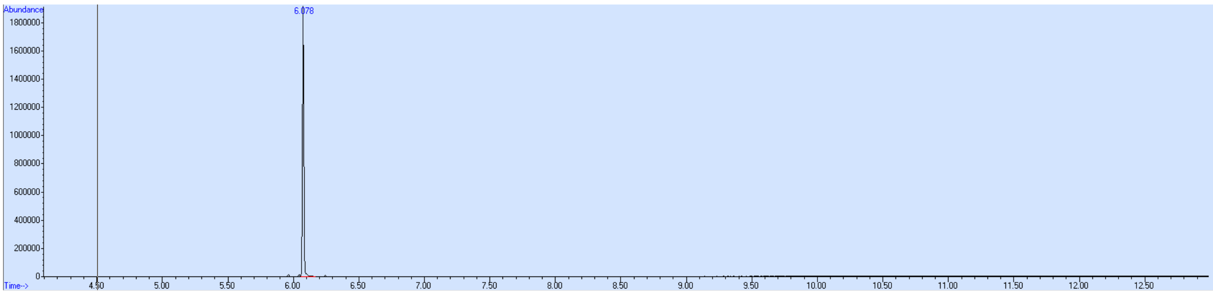

S22 Mass Spectrum @ 6.08 min

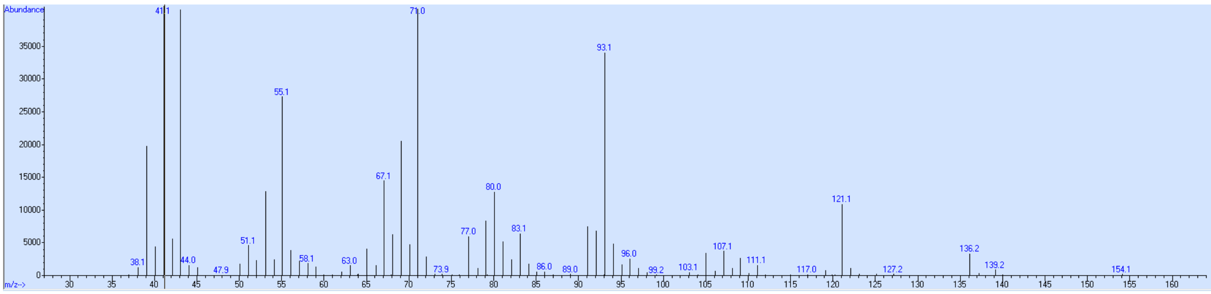

S32 Chromatogram

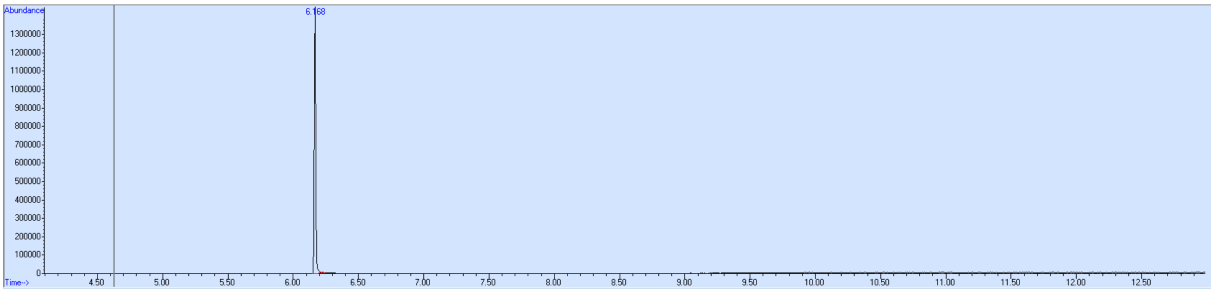

S32 Mass Spectrum @ 6.17 min

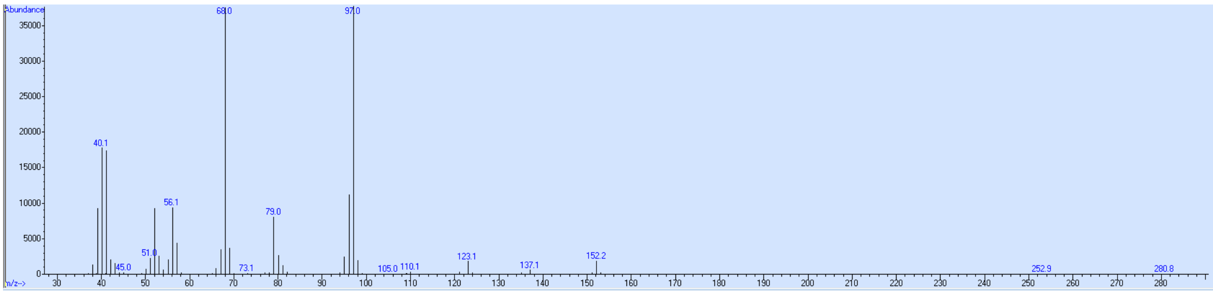

S38 Chromatogram

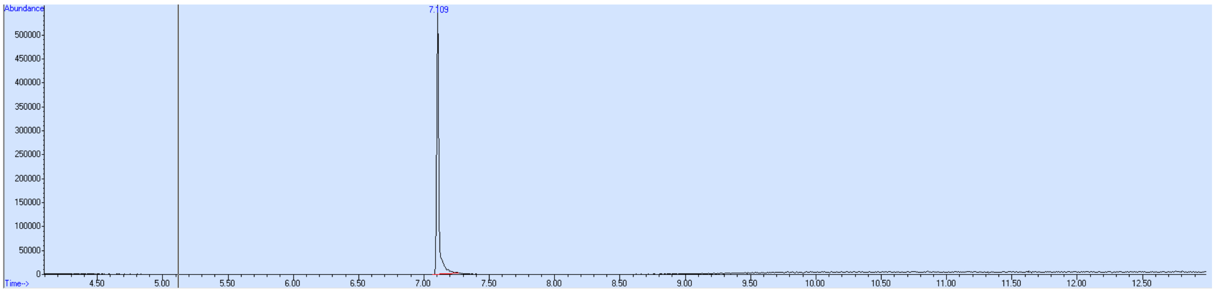

S38 Mass Spectrum @ 7.11 min

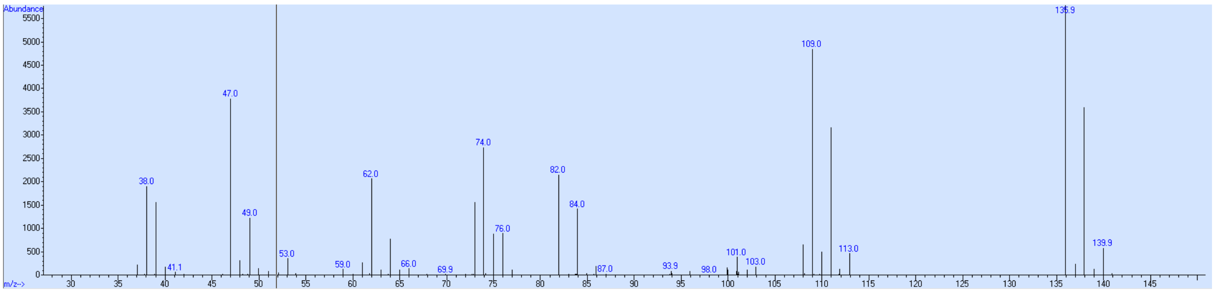

S48 Chromatogram

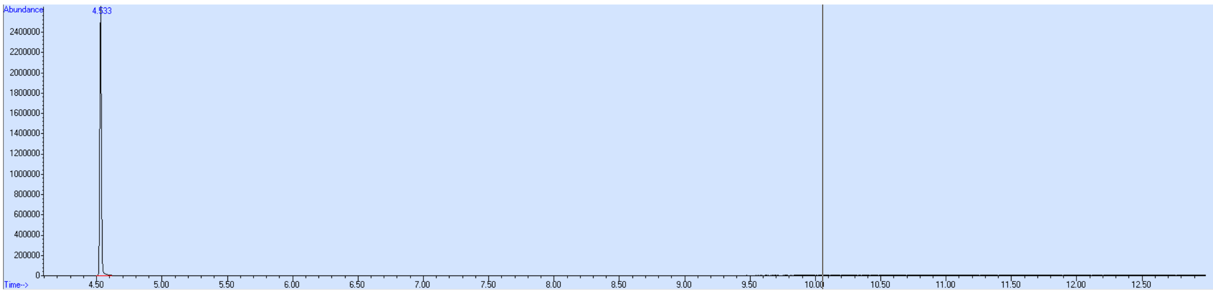

S48 Mass Spectrum @ 4.53 min

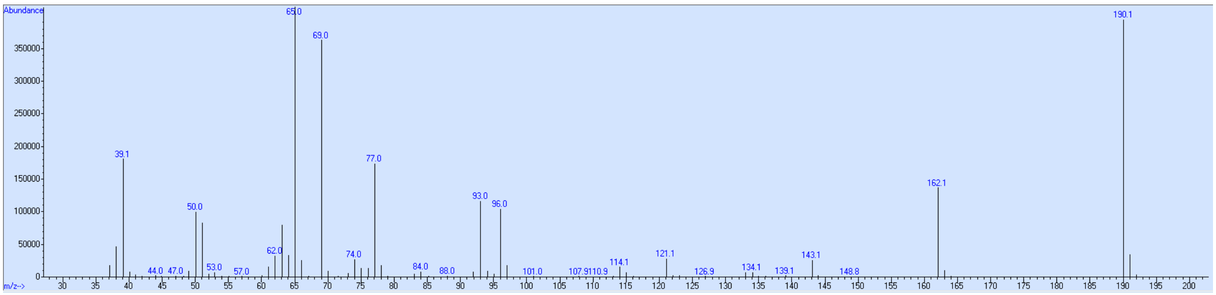

S75 Chromatogram

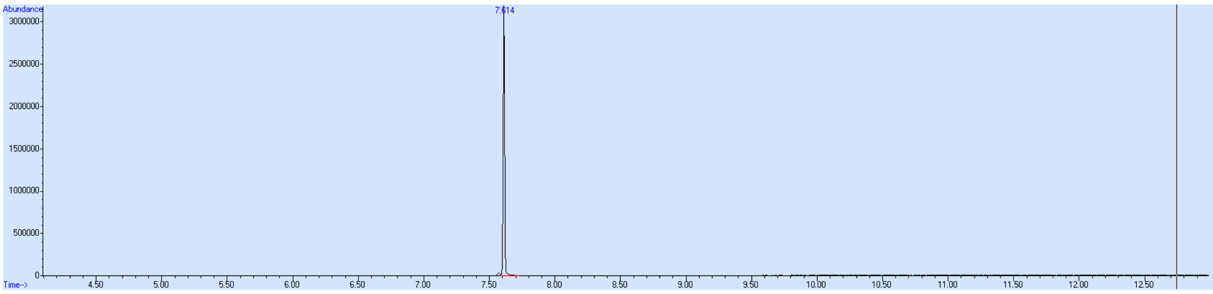

S75 Mass Spectrum @ 7.61 min

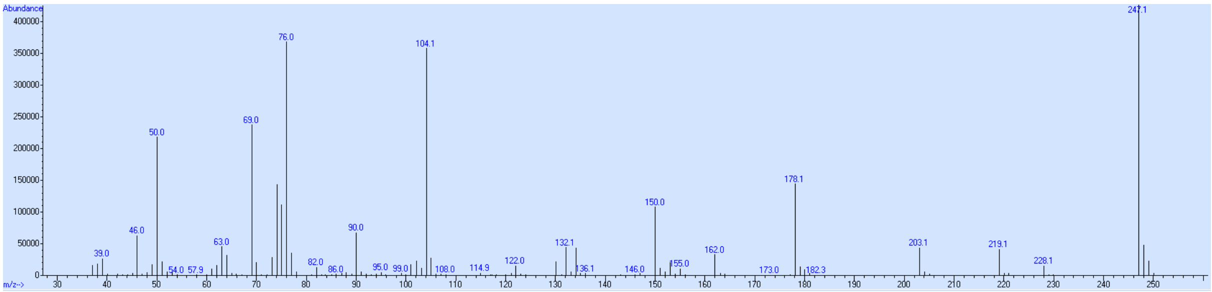

S88 Chromatogram

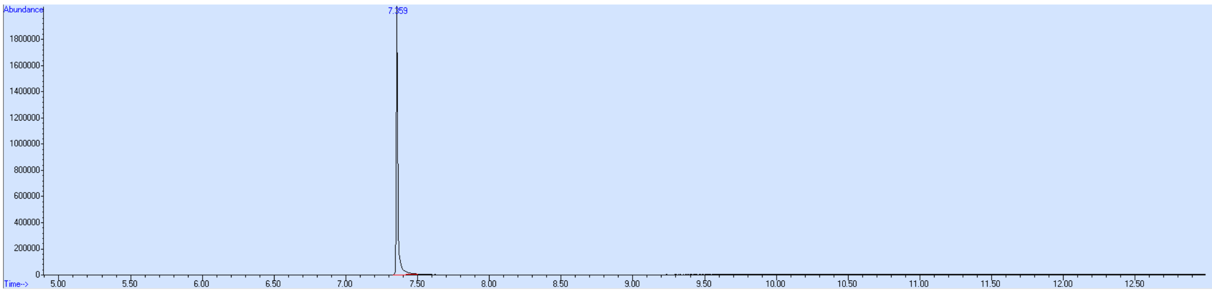

S88 Mass Spectrum @ 7.36 min

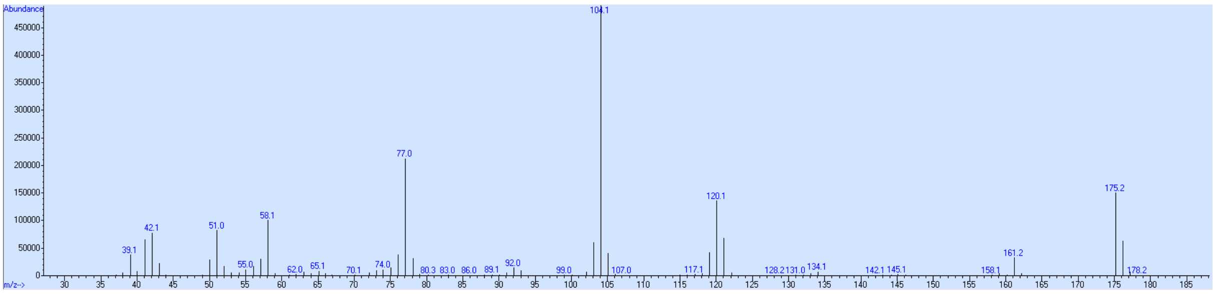

S95 Chromatogram

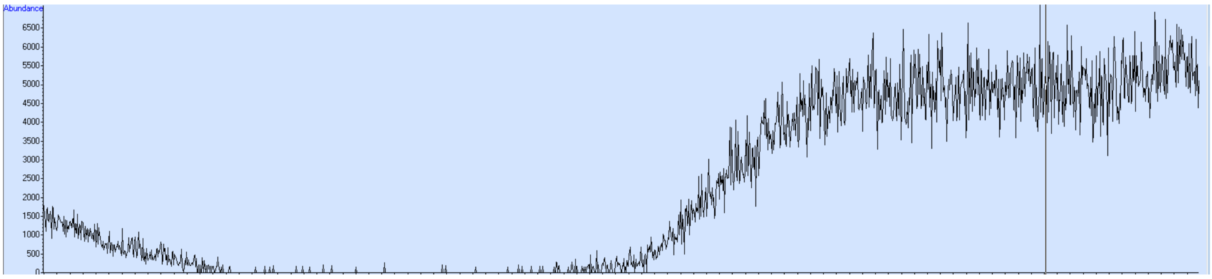

C1 Chromatogram

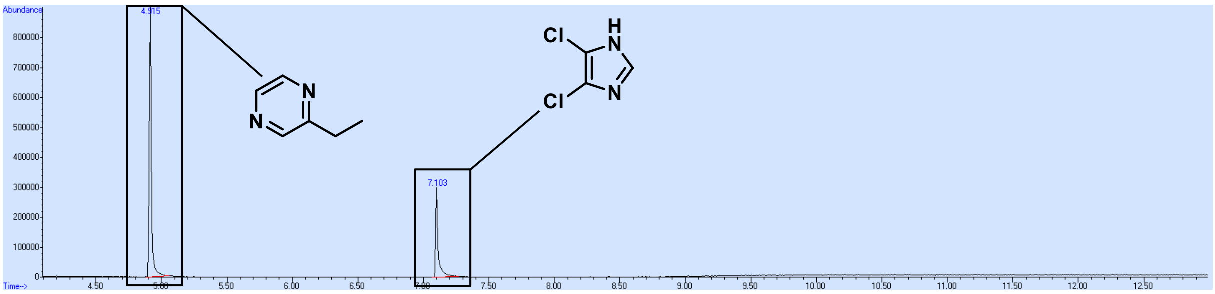

C2 Chromatogram

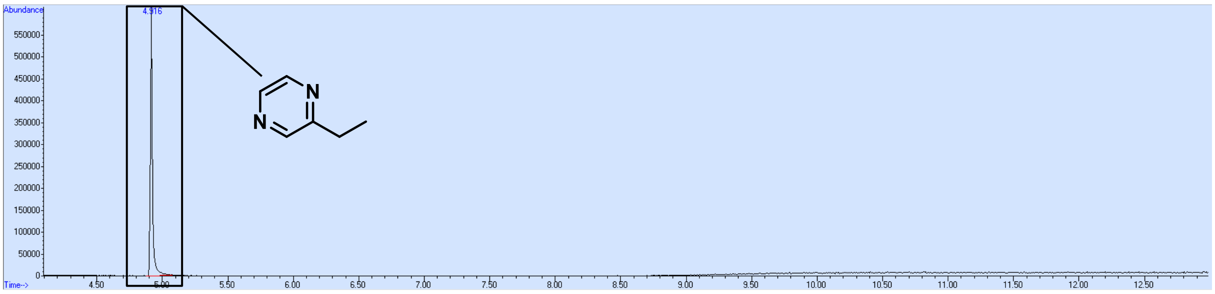

C3 Chromatogram

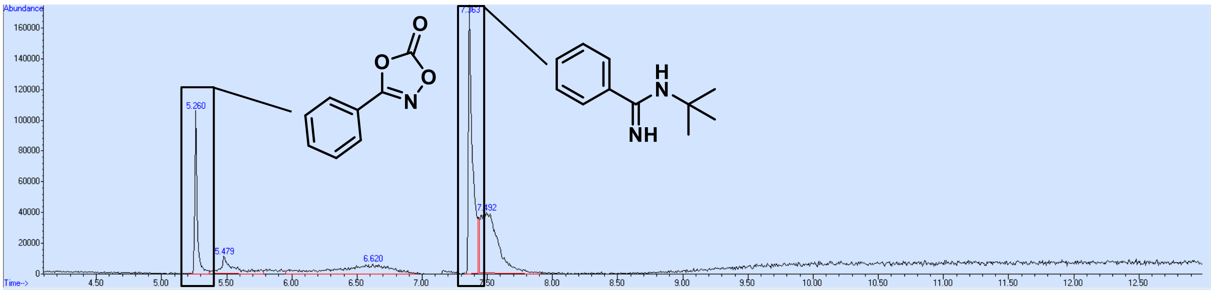

C3 Mass Spectrum @ 5.48 min

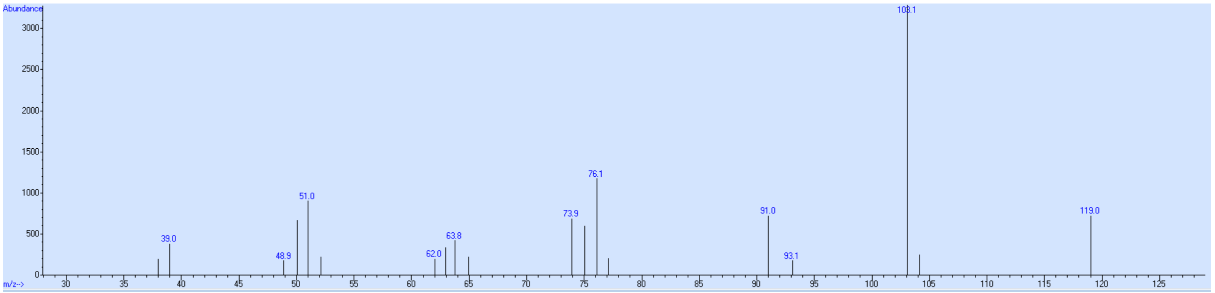

Possible Structure: 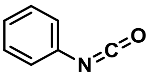

C3 Mass Spectrum @ 6.62 min

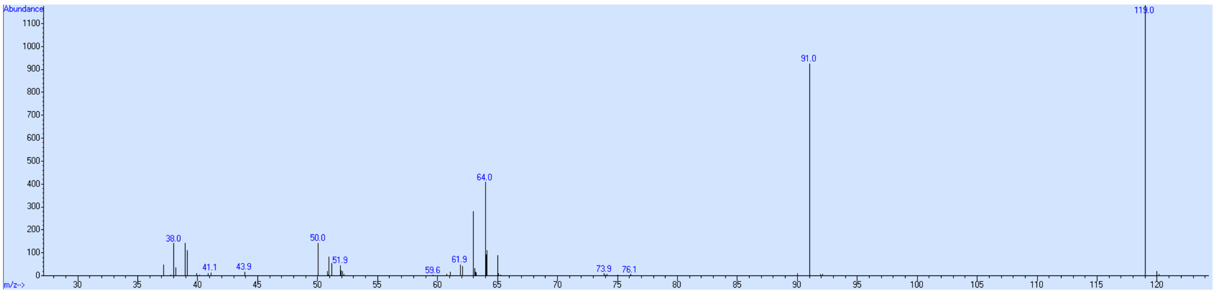

Possible Structure: 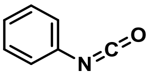

C3 Mass Spectrum @ 7.49 min

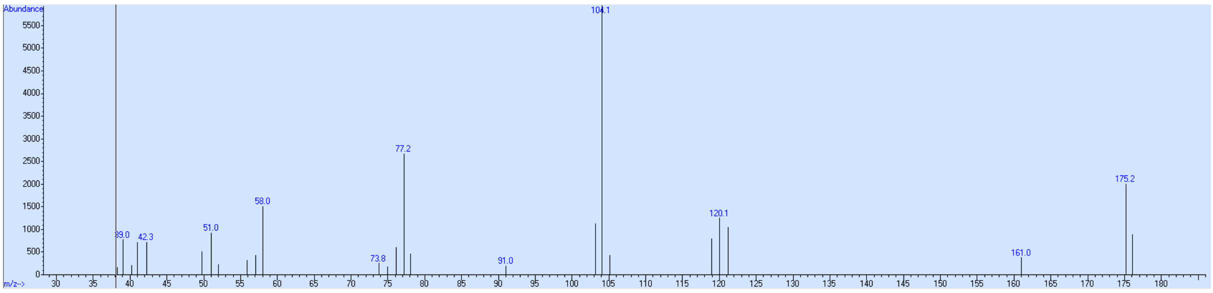

Possible Structure: 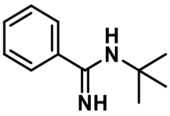

C4 Chromatogram

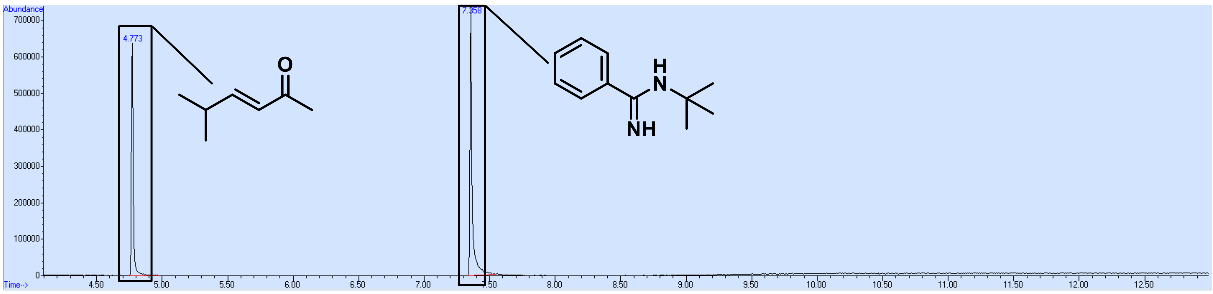

C5 Chromatogram

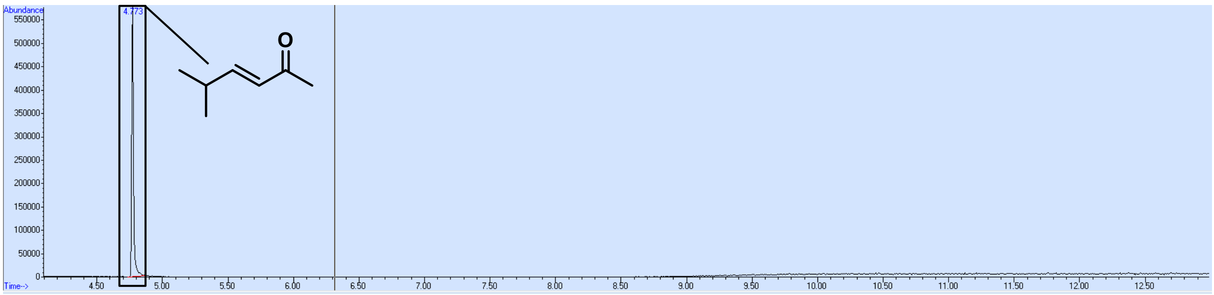

C6 Chromatogram

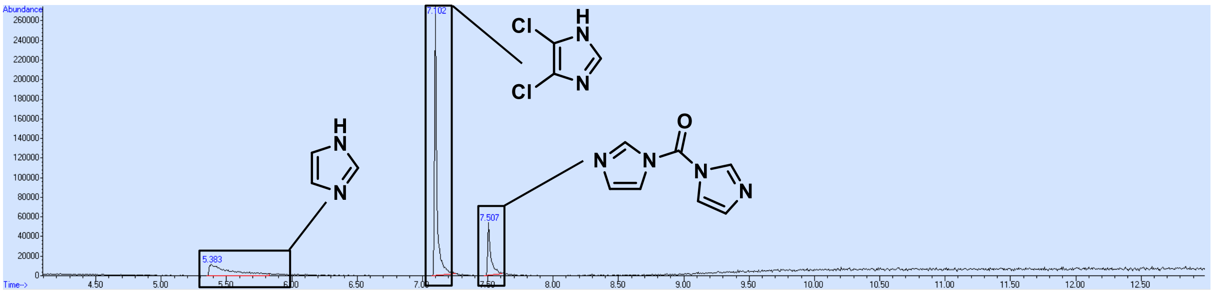

C7 Chromatogram

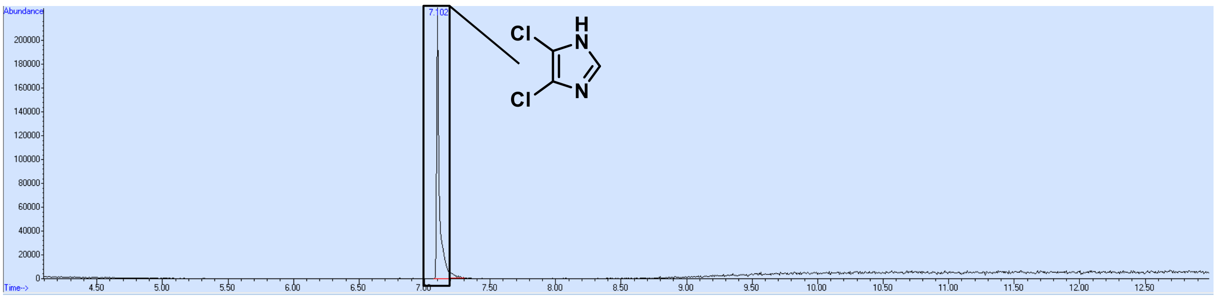

C8 Chromatogram

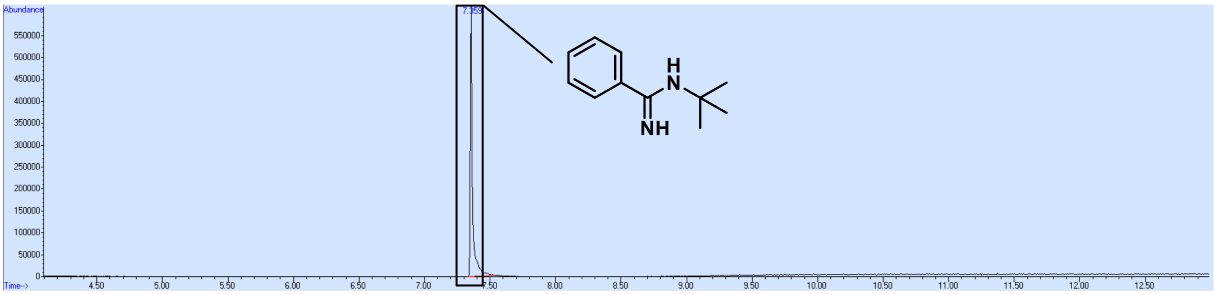

C9 Chromatogram

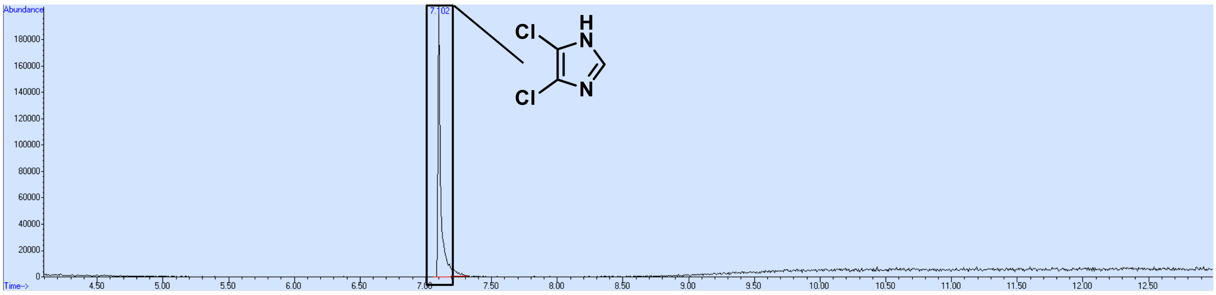

C10 Chromatogram

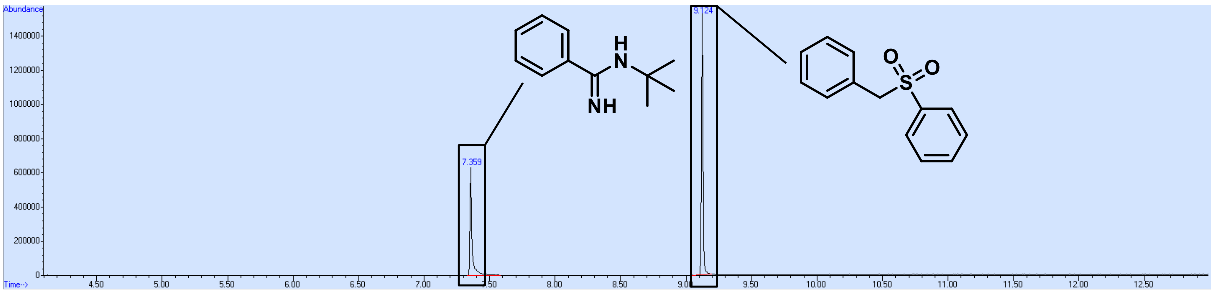

C11 Chromatogram

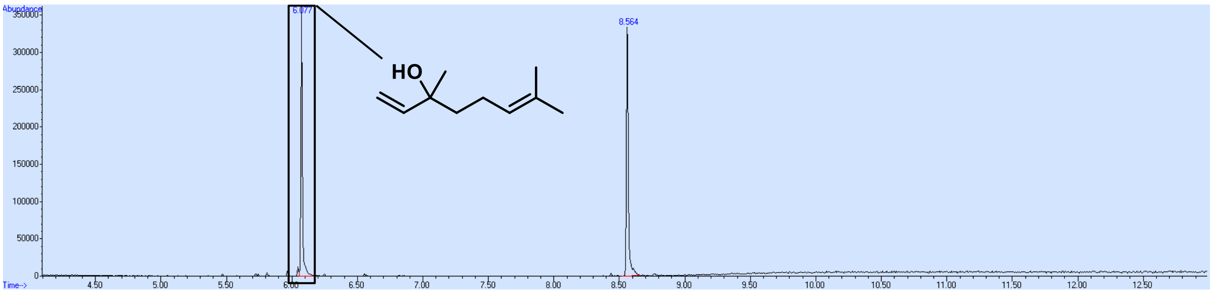

C11 Mass Spectrum @ 8.56 min

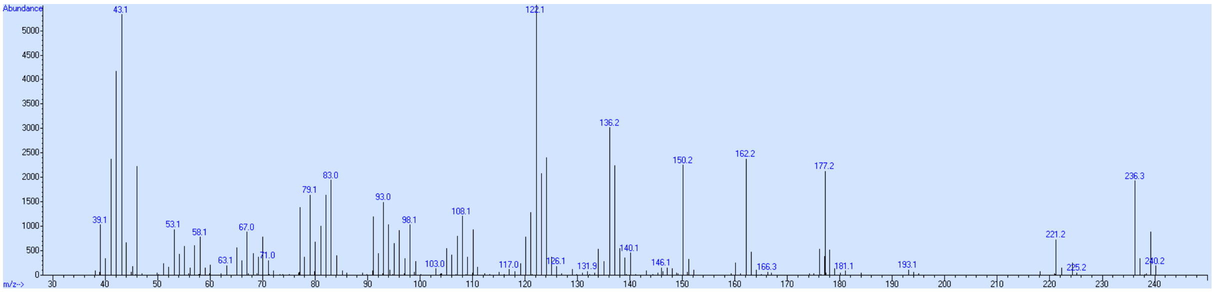

Possible Structure: unknown

C12 Chromatogram

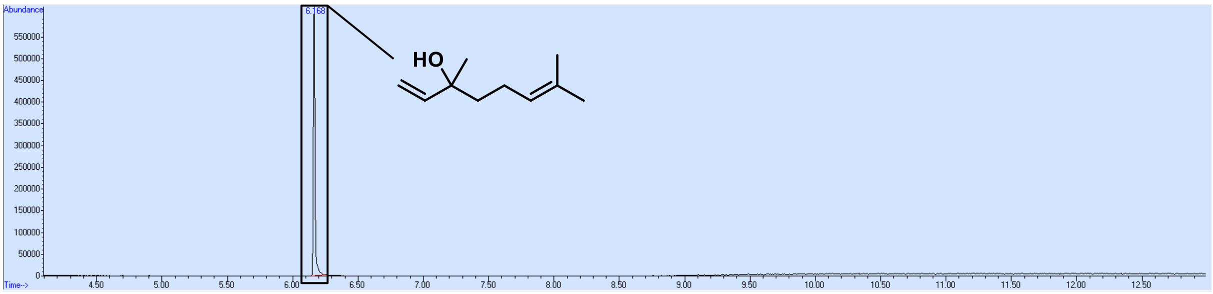

C13 Chromatogram

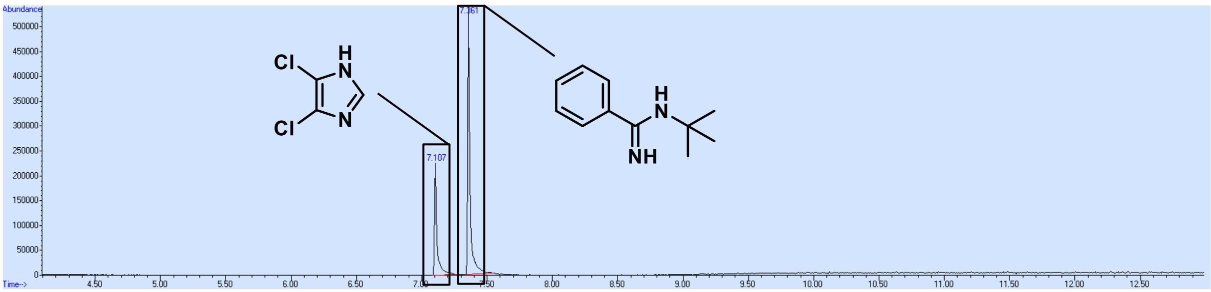

C14 Chromatogram

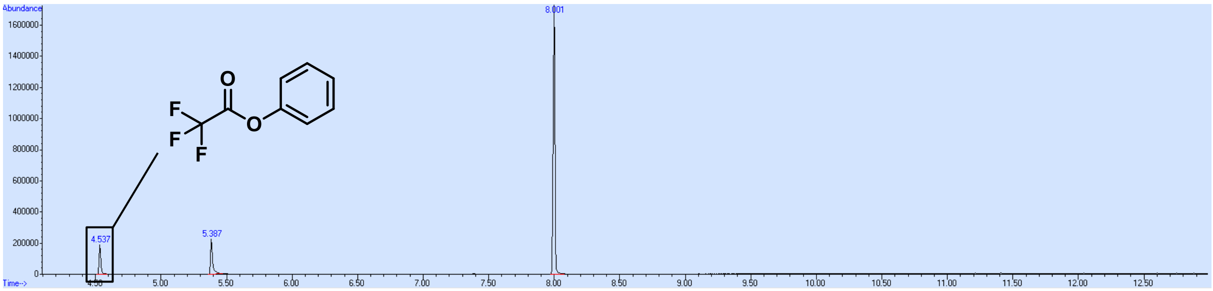

C14 Mass Spectrum @ 5.39 min

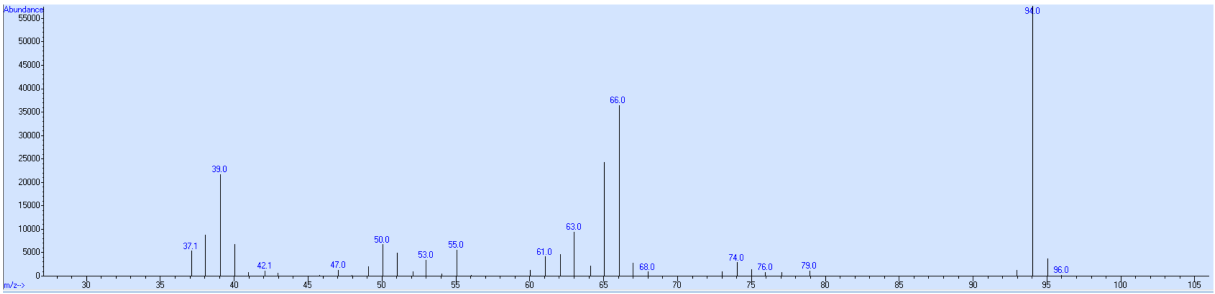

Possible Structure: 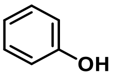

C14 Mass Spectrum @ 8.00 min

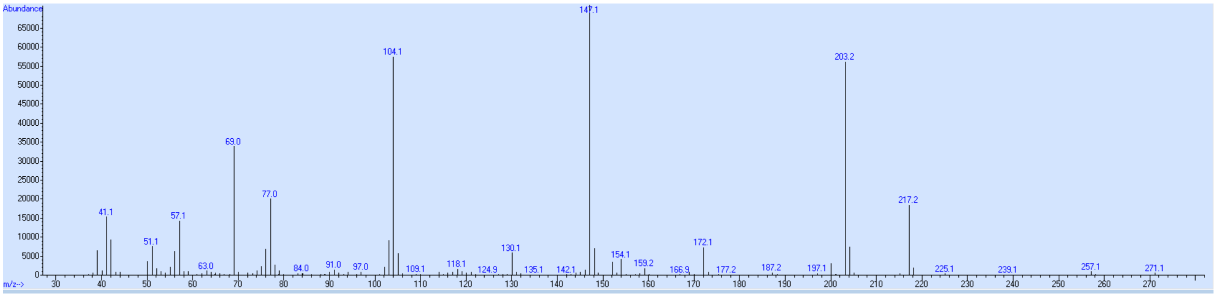

Possible Structure: 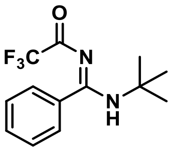

C15 Chromatogram

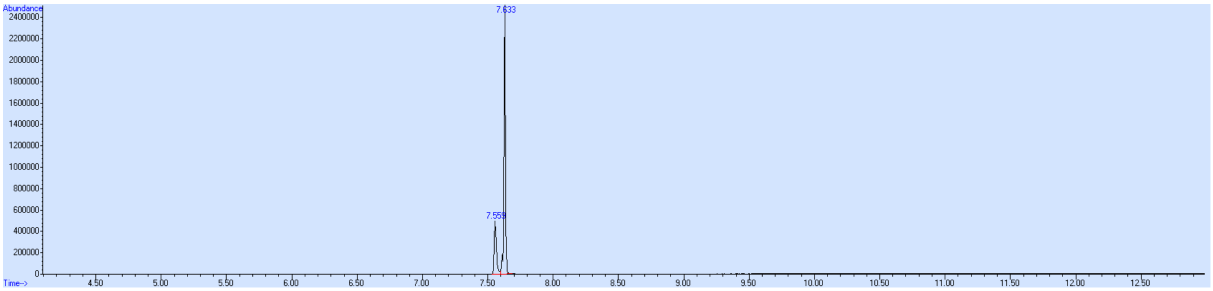

C15 Mass Spectrum @ 7.56 min

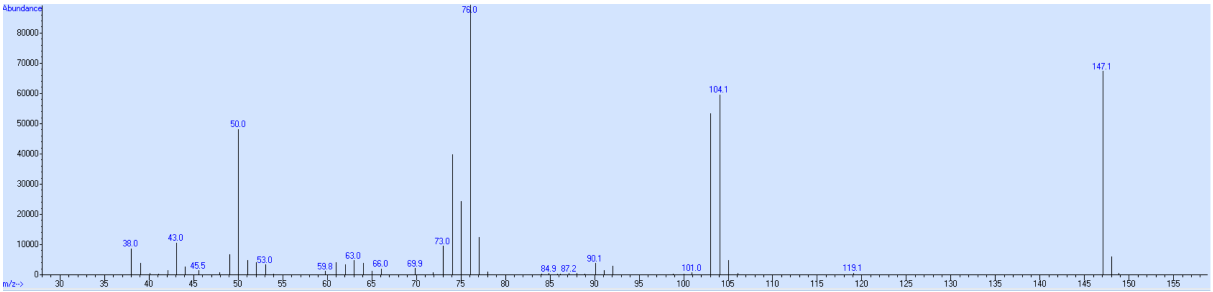

Possible Structure: 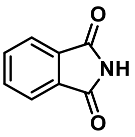

C15 Mass Spectrum @ 7.63 min

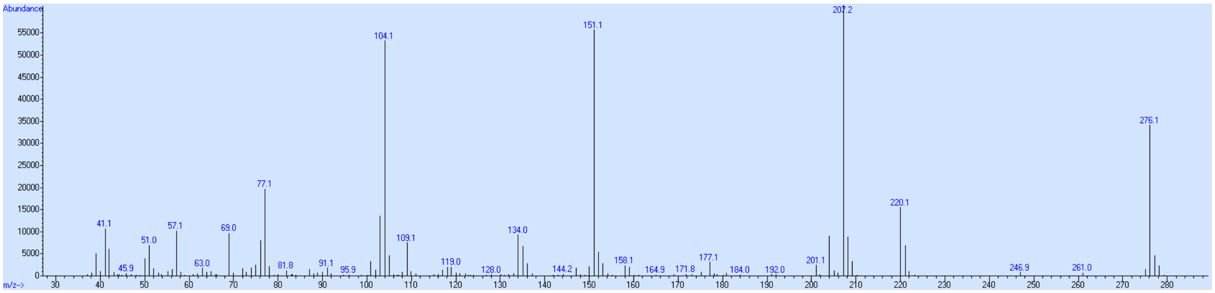

Possible Structure: 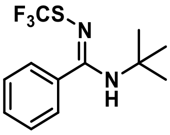

Table S23: Results of GC-MS studies of all cooperative pairs. The table states whether the individual compounds were found, if any impurity of the starting material was observed, the retention times of the new peaks and suggested structures corresponding to the new peak.

Pair N°	Expected SM found?	Impurities in SM?	New Peaks	Suggested Structures
C1	yes	no	no	
C2	yes	no	no	
C3	yes	no	t_R = 5.48 min 6.62 min, 7.49 min	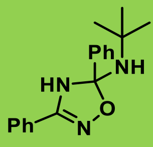 or 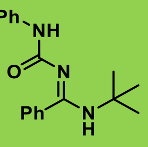
C4	yes	no	no	
C5	yes	no	no	
C6	yes	no		
C7	yes	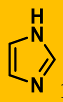 in **S11**	no	
C8	yes	no	no	
C9	yes	no	no	
C10	yes	no	no	
C11	yes	no	t_R = 8.56 min	unknown
C12	yes	no	no	
C13	yes	no	no	
C14	one of two	no	t_R = 5.39 min, 8.00 min	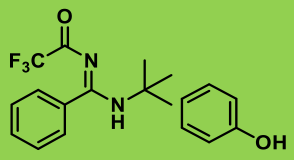
C15	no	no	t_R = 7.63 min, 7.56 min	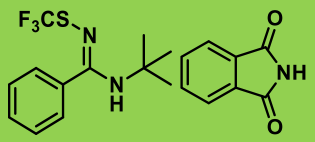

3.2.3 Interpretation

While most of the measured samples of the cooperative pairs show no additional peaks in their chromatogram (**C1**, **C2**, **C4**, **C5**, **C7**, **C8**, **C9**, **C10**, **C12**, **C13**), new peaks were observed in the chromatograms of **C3**, **C11**, **C14** and **C15**. In those cases, both substrates of the cooperative pair undergo a chemical reaction releasing a compound which is able to quench the excited state photocatalyst **PC1** (cooperativity via reactivity). An impurity of imidazole was found within the sample of **S11**, which could be caused by hydrolysis.

C3

The chromatogram of the cooperative pair **C3** shows three new and broad peaks. Based on their fragmentation pattern, the first two peaks (t_R = 5.48 min, 6.62 min) seem to correspond to isocyanatobenzene, while the third peak is likely caused by *N*-(*tert*-butyl)benzimidamide (**S88**) (t_R = 7.45 min). The peaks are exceptionally broad, and the retention times are higher than expected, leading to the conclusion that a thermally instable species forms within the reaction mixture which decomposes slowly during the high temperature chromatography process. This species contains parts of *N*-(*tert*-butyl)benzimidamide (**S88**) as well as 3-phenyl-1,4,2-dioxazol-5-one (**S5**). As the amidine **S88** is known to be a strong nucleophile and 3-phenyl-1,4,2-dioxazol-5-one (**S5**) could release CO_2 as part of an occurring reaction, we suggest the structure of this quencher as shown in Table S23 (entry C3).

C14

The chromatogram of the cooperative pair **C14** shows two additional peaks, while no peak corresponding to amidine **S88** could be found. The first peak (t_R = 5.39 min) is caused by phenol, while the second one (t_R = 8.00 min) is likely to correspond to *N*-((*tert*-butylamino)(phenyl)methylene)-2,2,2-trifluoroacetamide (Table S23, entry **C14**). A possible mechanism of this formation could be a nucleophilic attack of the amidine **S88** at the carbonyl carbon of phenyl trifluoroacetate (**S48**), leading to the release of phenol and the formation of *N*-((tert-butylamino)(phenyl)methylene)-2,2,2-trifluoroacetamide. The latter compound is suggested to cause the observed quenching.

C15

The chromatogram of the cooperative pair **C15** shows only two peaks, both of them not corresponding to *N*-(*tert*-butyl)benzimidamide (**S88**) and *N*-(trifluoromethylthio)phthalimide (**S75**). The first peak (t_R = 7.63 min) is caused by phthalimide, while the second one (t_R = 7.56

min) corresponds to *N*-(*tert*-butyl)-*N'*-((trifluoromethyl)thio)benzimidamide (Table S23, entry **C15**). This compound is suggested to cause the observed quenching (5.1 to 5.3).

3.3 Thin Layer Chromatography Studies

3.3.1 Analytical Details

10 µL of the stock solution of both substrates (0.2 M) were combined in a glass-vial (2.0 mL) and mixed. After roughly 24 h, the samples were spotted 3 times on a thin layer chromatography (TLC) plate using a capillary tube. For comparison, the samples containing the individual compound (0.2 M) were spotted (1 time each) on the same plate. This procedure was repeated, thereby obtaining two TLC plates which were developed (pentane:EtOAc, 9:1 v/v and pure EtOAc) in a glass chamber. After development, the plates were taken out of the glass chamber, dried and visualized by exposure to UV light (λ = 254 nm and 365 nm), while all visible spots were marked. The plates were stained by oxidation with a $KMnO_4$ solution, all new spots were marked, and the R_f-values were measured for comparison.

3.3.2 Results

C1 TLC

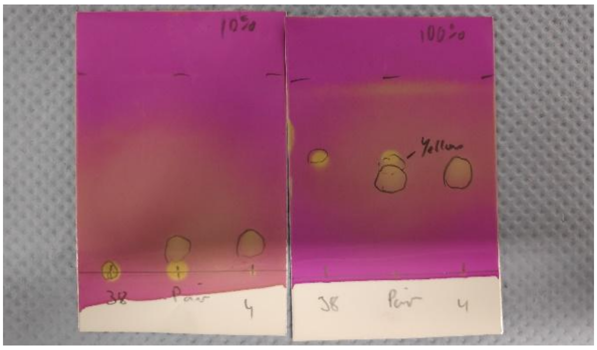

C2 TLC

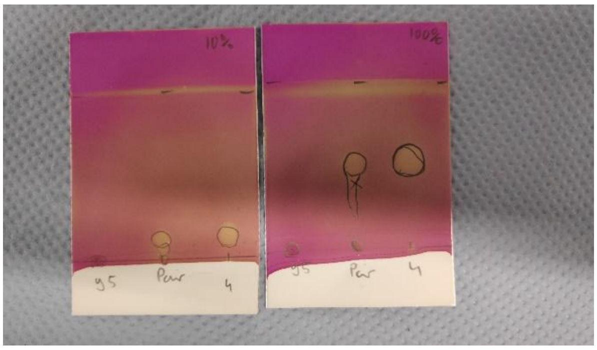

C3 TLC

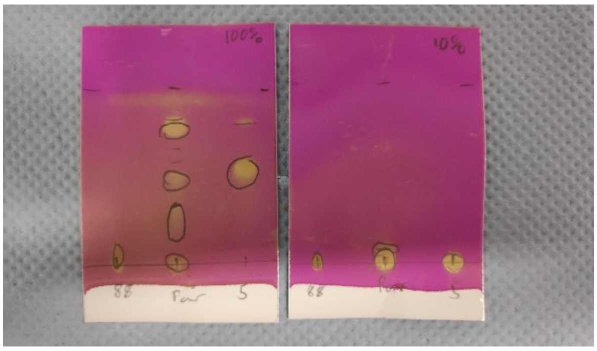

C4 TLC

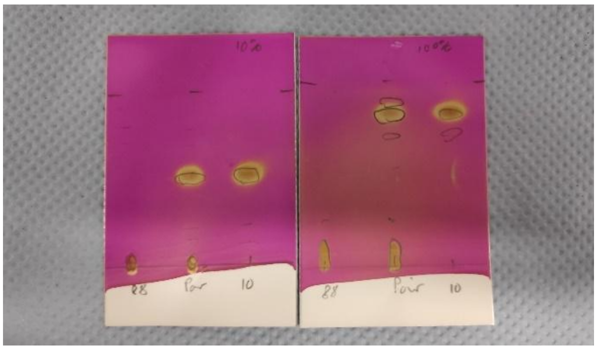

C5 TLC

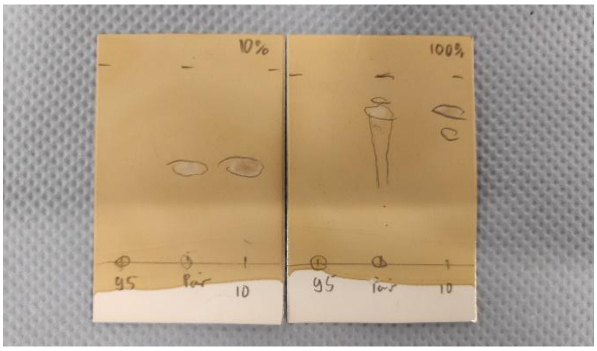

C6 TLC

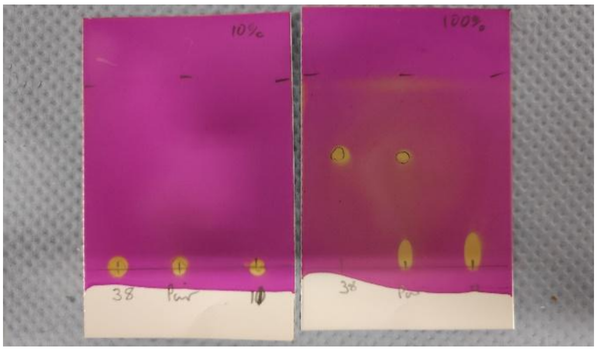

C7 TLC

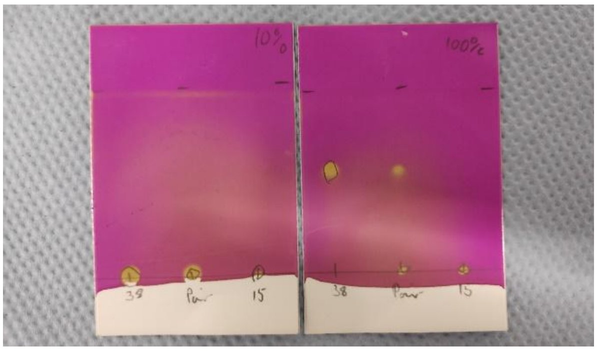

C8 TLC

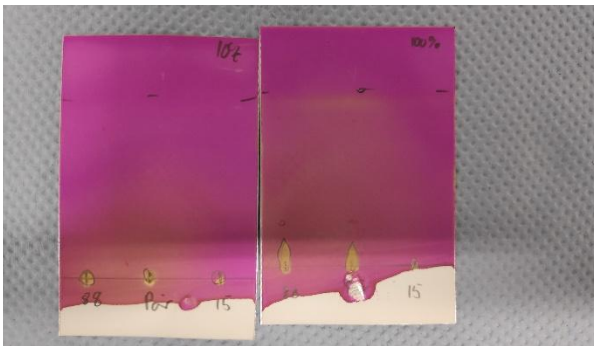

C9TLC

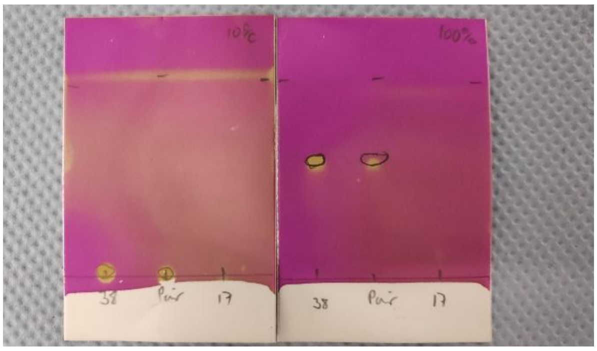

C10 TLC

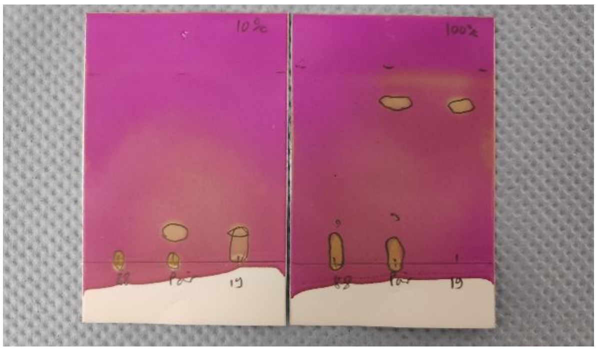

C11 TLC

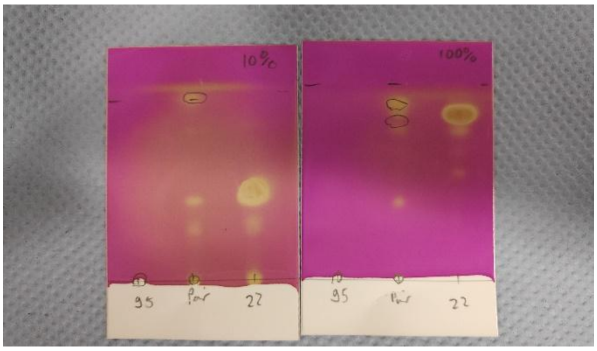

C12 TLC

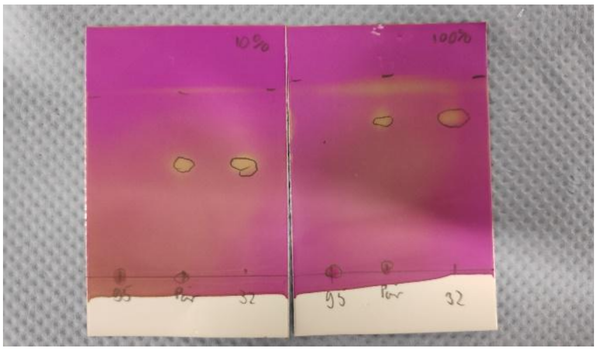

C13 TLC

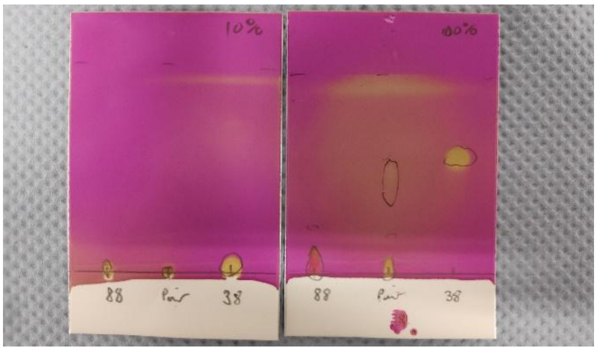

C14 TLC

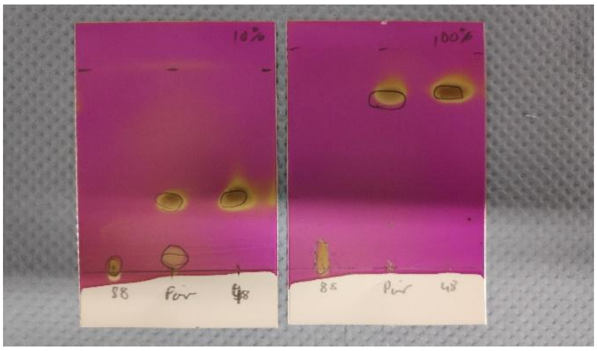

C15 TLC

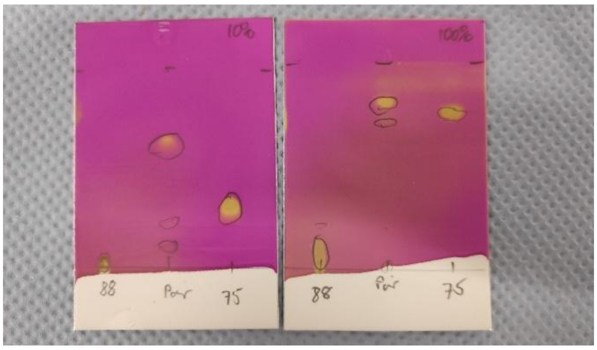

Table S24: Measured R_f-values of all individual compounds.

**Compound N°**	$\mathbf{R_f}$ (EtOAc)	$\mathbf{R_f}$ (pentane:EtOAc, 9:1 v/v)	**Visible impurities**
**S4**	0.50	0.11	-
**S5**	0.49	0.00	-
**S10**	0.83	0.50	Traces of 1 compound (lower R_f)
**S11**	0.90	0.00	-
**S15**	0.00	0.0	-
**S17**	0.11	0.00	-
**S19**	0.81	0.16	-
**S22**	0.84	0.48	traces of 3 compounds (lower R_f)
**S32**	0.79	0.60	-
**S38**	0.57	0.04	-
**S48**	0.78	0.37	-
**S75**	0.76	0.29	-
**S88**	0.08	0.00	-
**S95**	0.00	0.00	-

Table S25: Measured R_f-values of new spots of the cooperative pair.

Pair N°	New spot observed?	R_f (EtOAc)	R_f (pentane:EtOAc, 9:1 v/v)
**C1**	pale-yellow spot	0.56	not visible
**C2**	no	-	-
**C3**	UV/$KMnO_4$	0.75/0.23	0.07/0.00
**C4**	no	-	-
**C5**	no	-	-
**C6**	no	-	-
**C7**	no	-	-
**C8**	no	-	-
**C9**	no	-	-
**C10**	no	-	-
**C11**	UV/$KMnO_4$	0.92	0.91
**C12**	no	-	-
**C13**	UV/$KMnO_4$	0.52	not visible
**C14**	no	-	-
**C15**	UV/$KMnO_4$	0.82	0.62

3.3.3 Interpretation

While most of the measured samples of the cooperative pairs show no additional spots on TLC (**C2**, **C4**, **C5**, **C7**, **C8**, **C9**, **C10**, **C12**, **C14**), new spots were observed in the case of **C1**, **C3**, **C11**, **C13**, **C15**.

C1

A pale-yellow spot is visible in between the spots of the individual compounds, which could relate to a species composed of **S38** and **S4**. This species is not visible under UV light and using $KMnO_4$-stain.

C3

Two new spots are visible under UV light and using $KMnO_4$-stain, indicating the occurrence of a chemical reaction between species **S88** and **S5**.

C11

One new spot is visible under UV light and using $KMnO_4$-stain, suggesting the occurrence of a chemical reaction between species **S95** and **S22**. The low polarity of this species as well as the molecular structure of **S22** indicate a reaction in which the alcohol eliminates.

C13

One new spot is visible under UV light and using $KMnO_4$-stain, which is located in between the spots of the individual compounds which could relate to a species composed of **S88** and **S38**.

C15

One new spot is visible under UV light and using $KMnO_4$-stain, suggesting the occurrence of a chemical reaction between species **S88** and **S75**.

3.4 Nuclear Magnetic Resonance Studies

3.4.1 Analytical Details

To prepare reference samples for every individual compound, 700 µL of the stock solution (0.2 M) were transferred to an NMR tube. To prepare samples of the cooperative pairs, 350 µL of both stock solutions were transferred to a glass vial (2.0 mL) and mixed. This mixture was then transferred to an NMR tube and measured within 24 h.

3.4.2 Results

C1 NMR-Spectrum

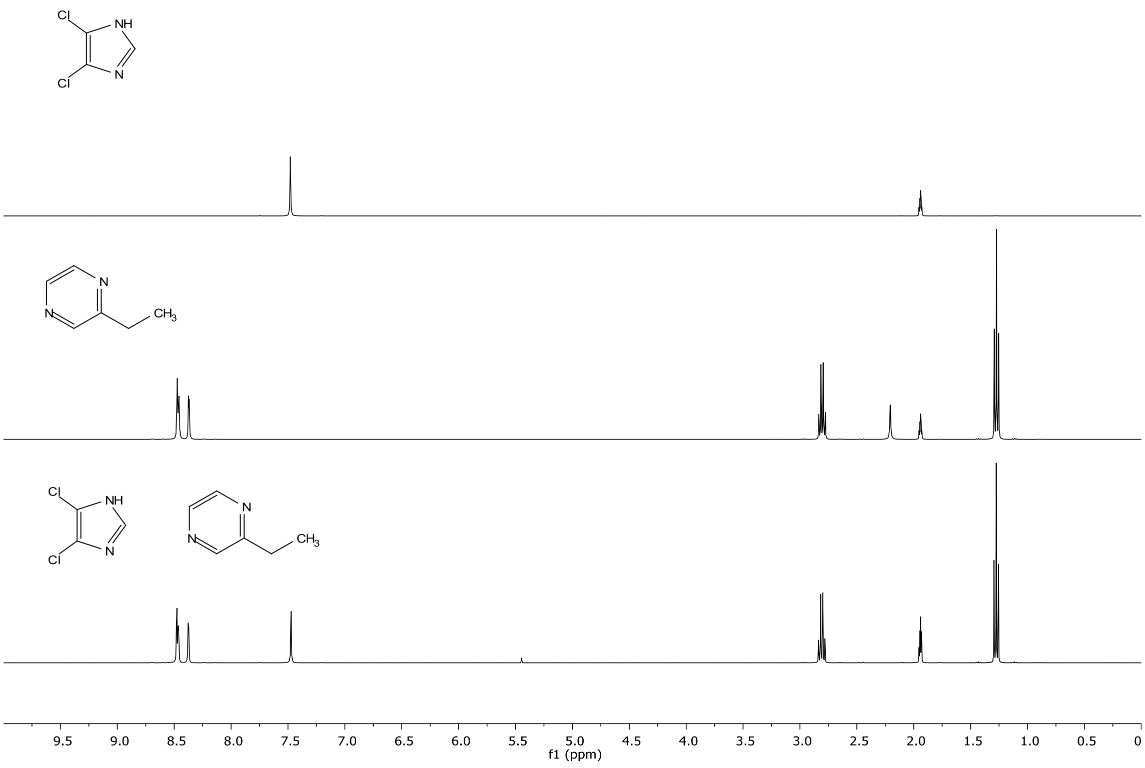

C2 NMR-Spectrum

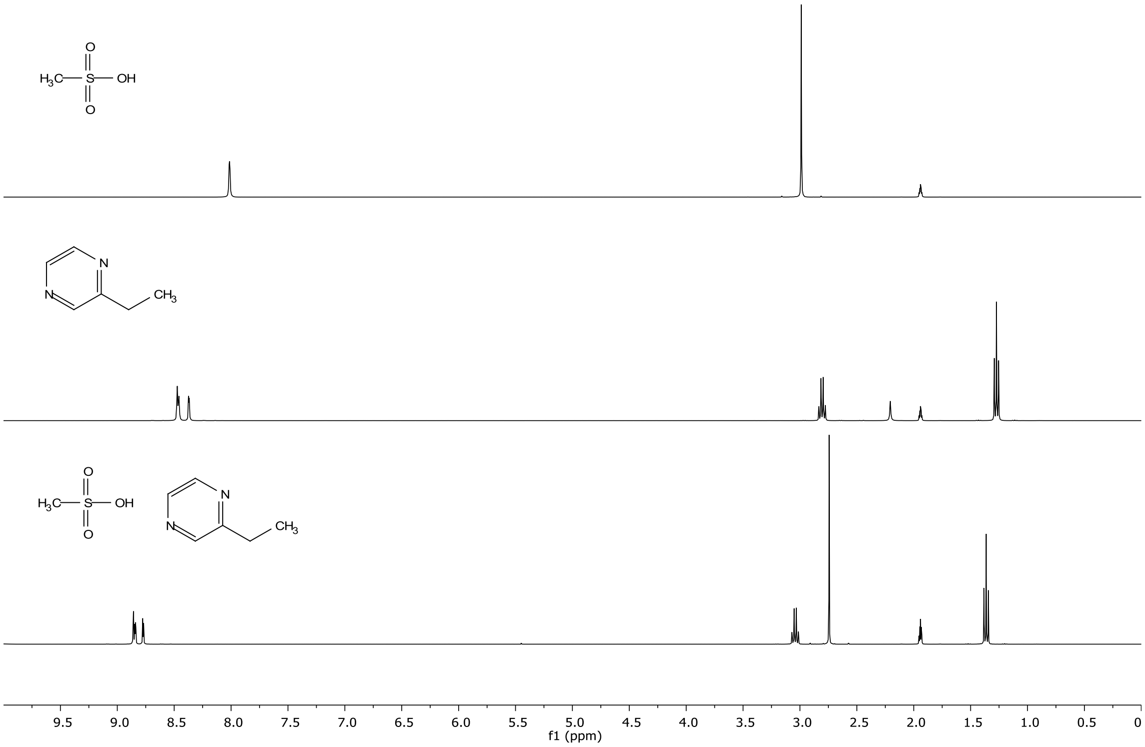

C3 NMR-Spectrum

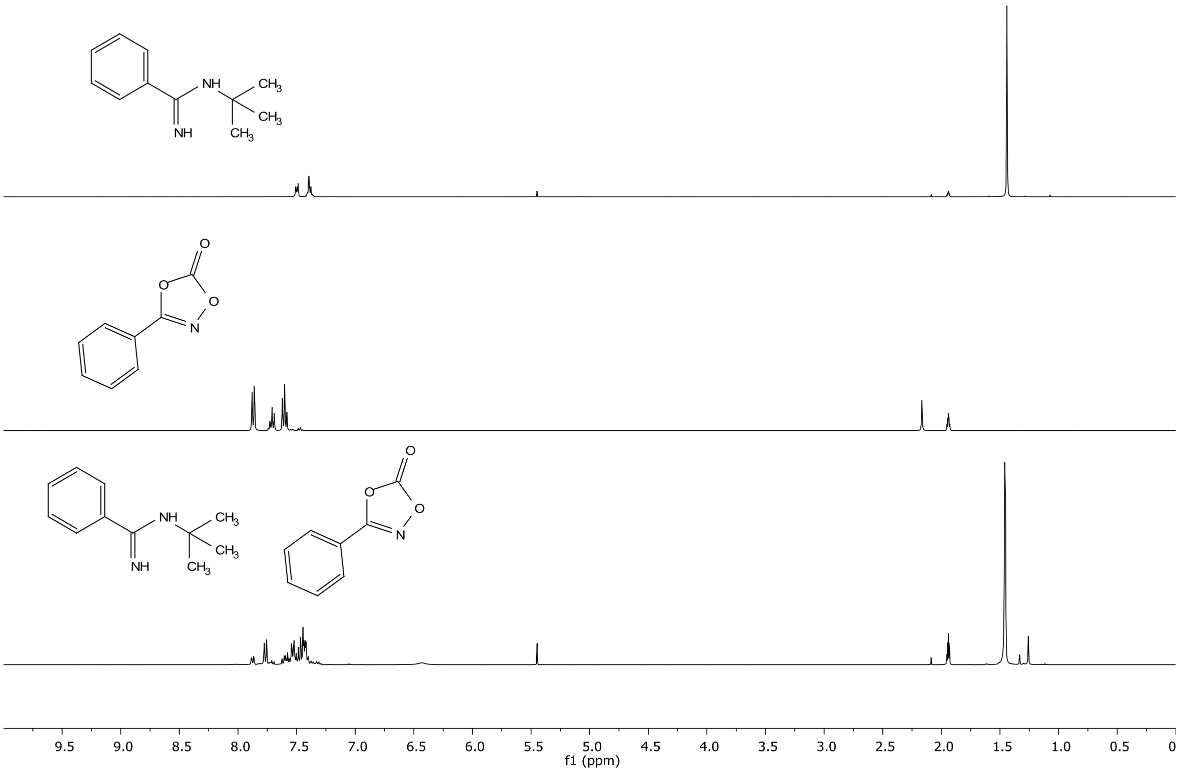

C4 NMR-Spectrum

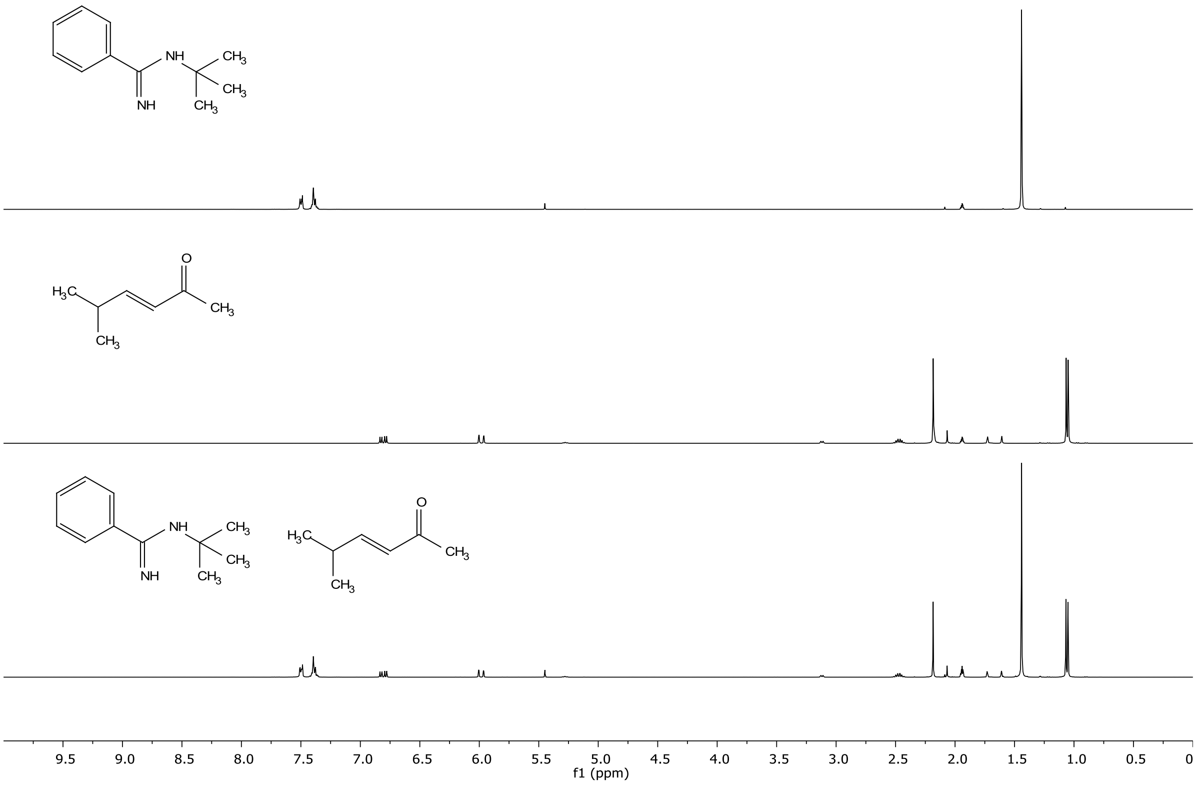

C5 NMR-Spectrum

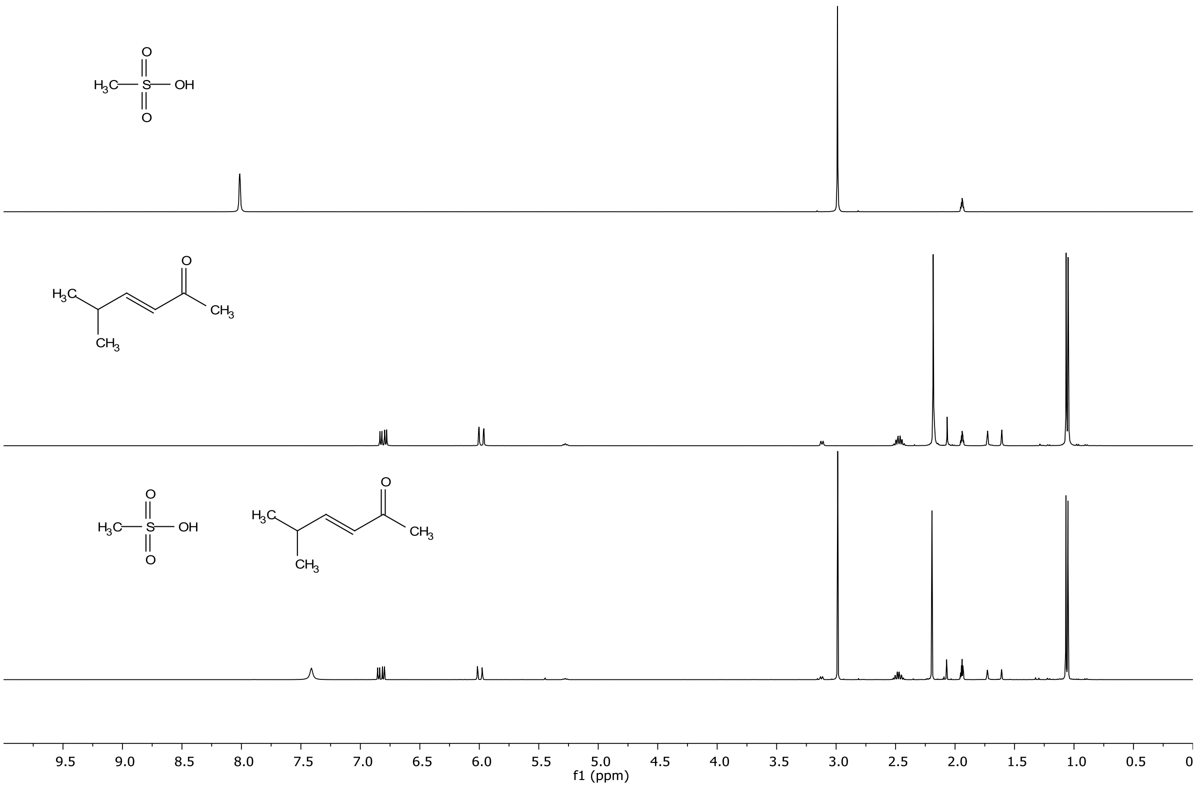

C6 NMR-Spectrum

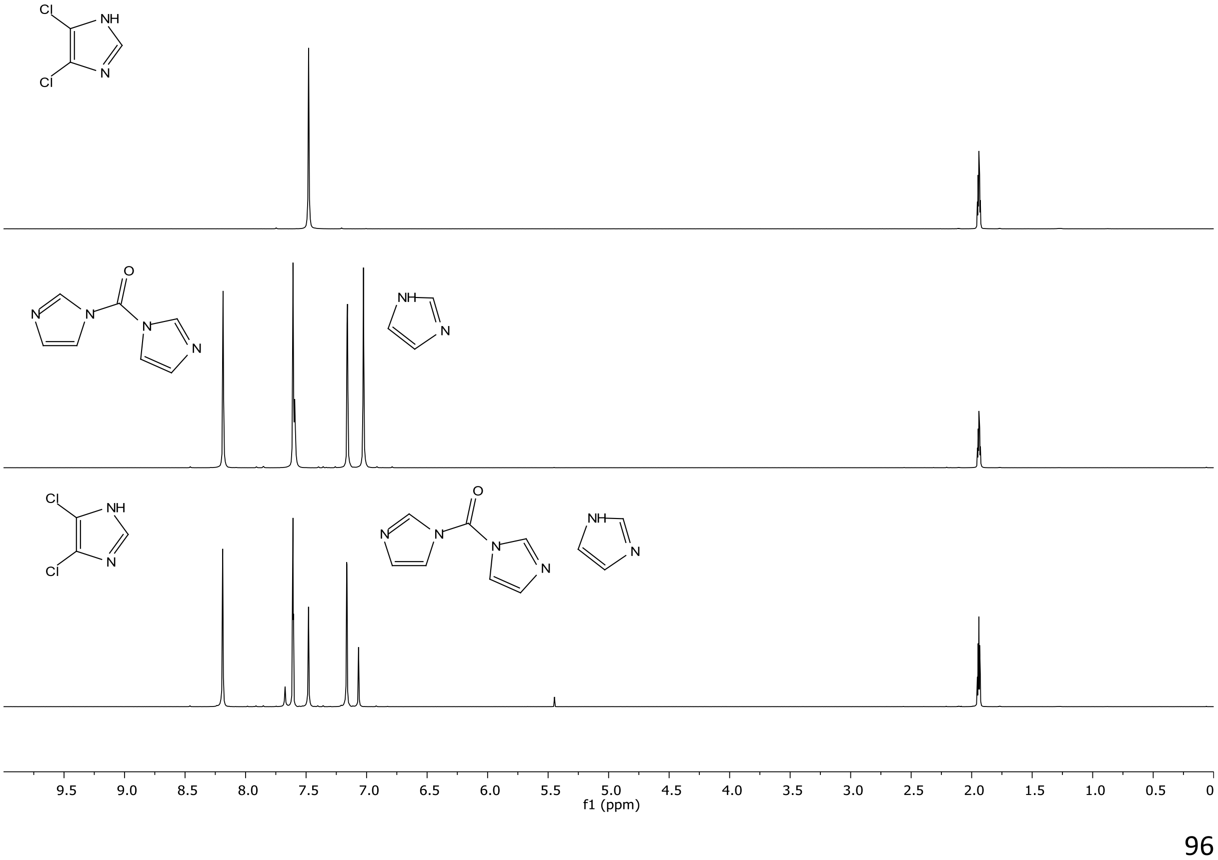

C7 NMR-Spectrum

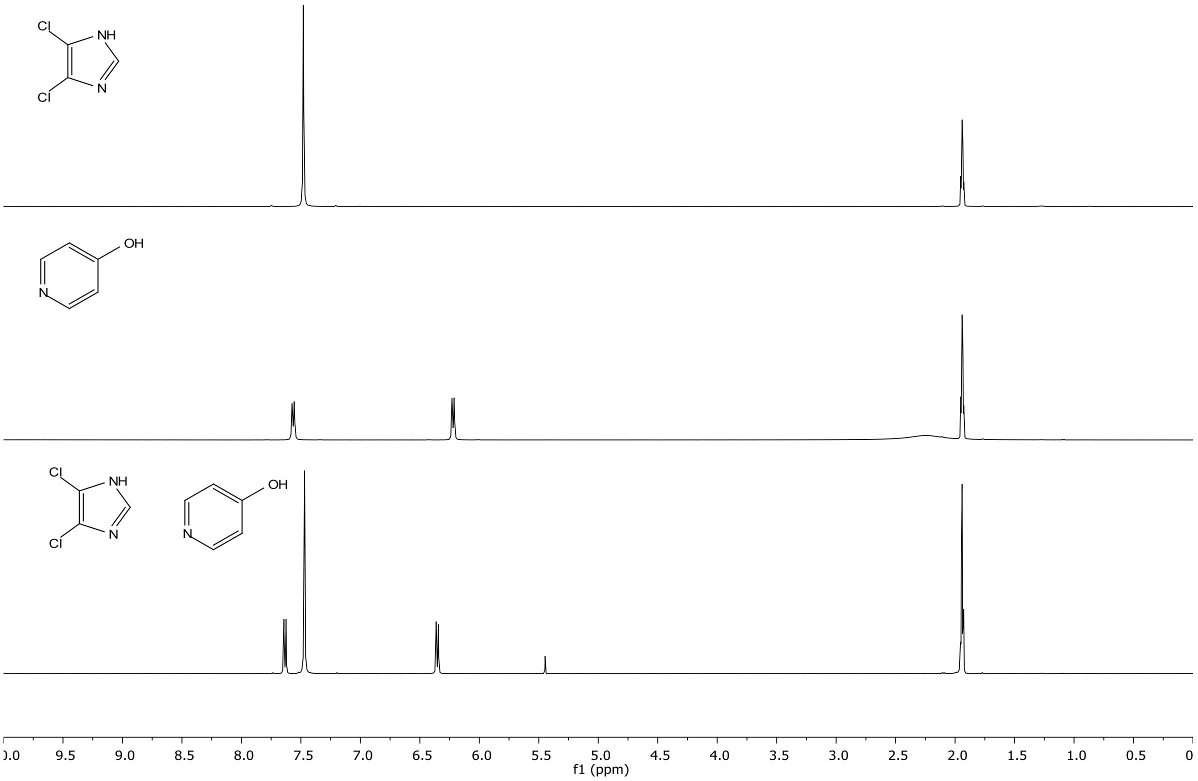

C8 NMR-Spectrum

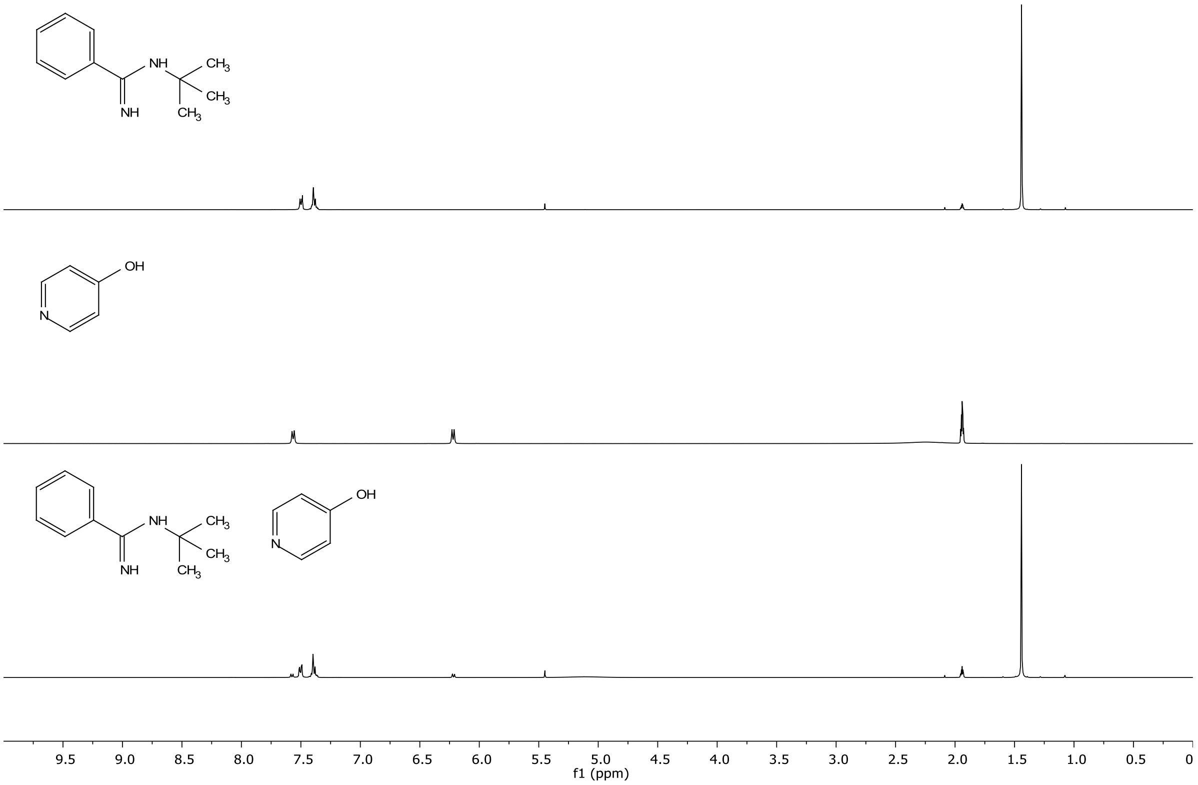

C9 NMR-Spectrum

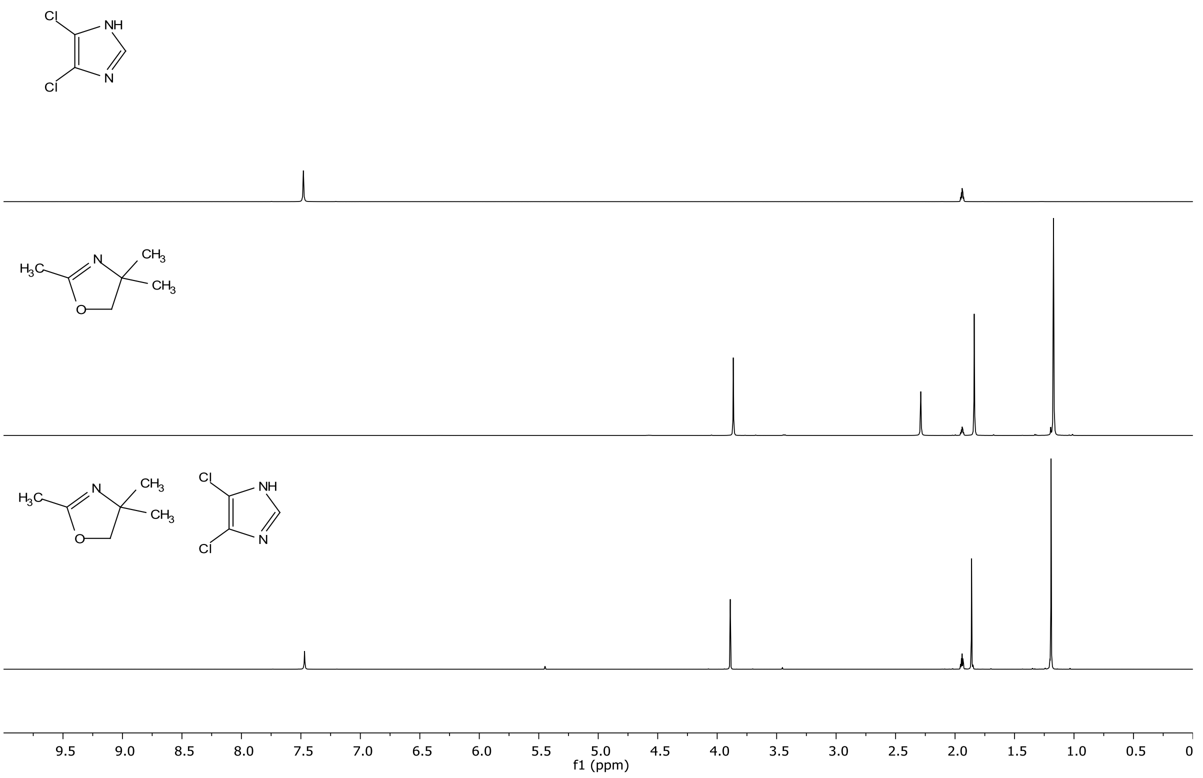

C10 NMR-Spectrum

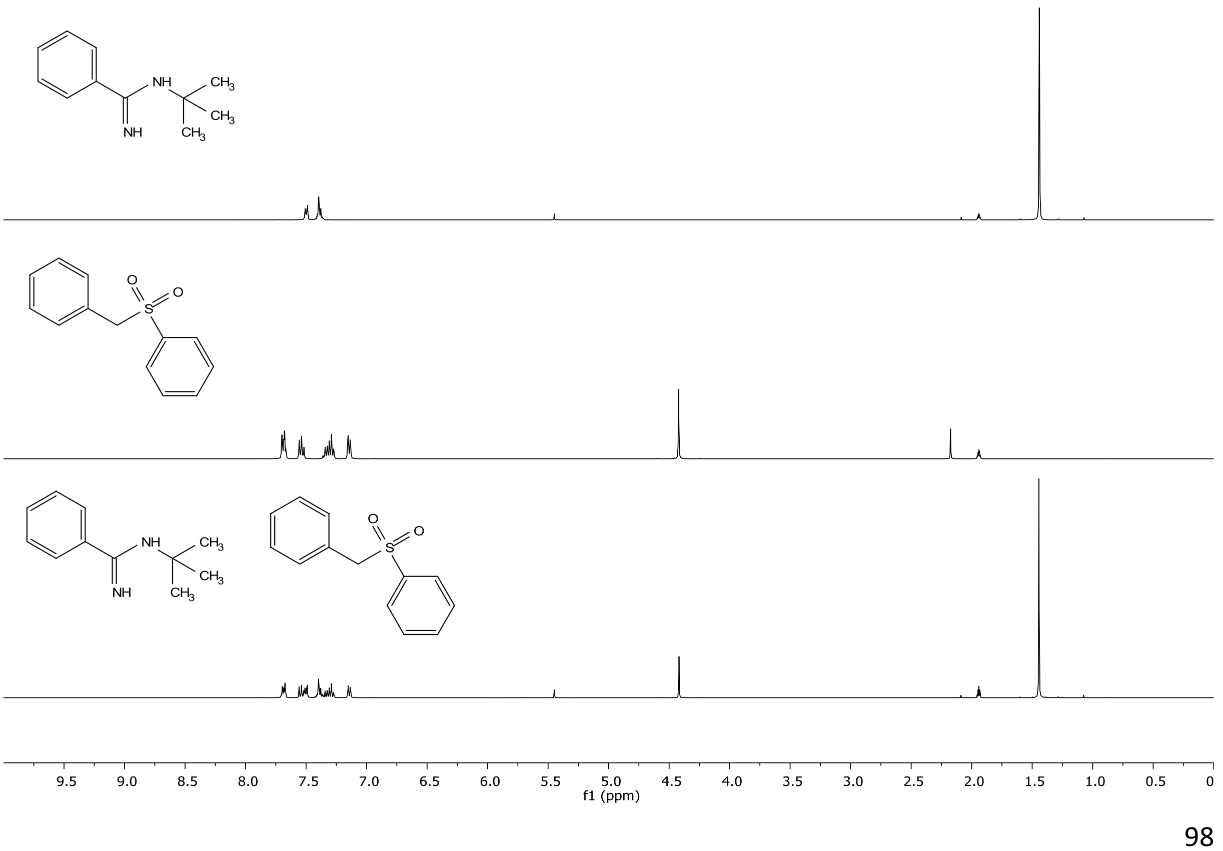

C11 NMR-Spectrum

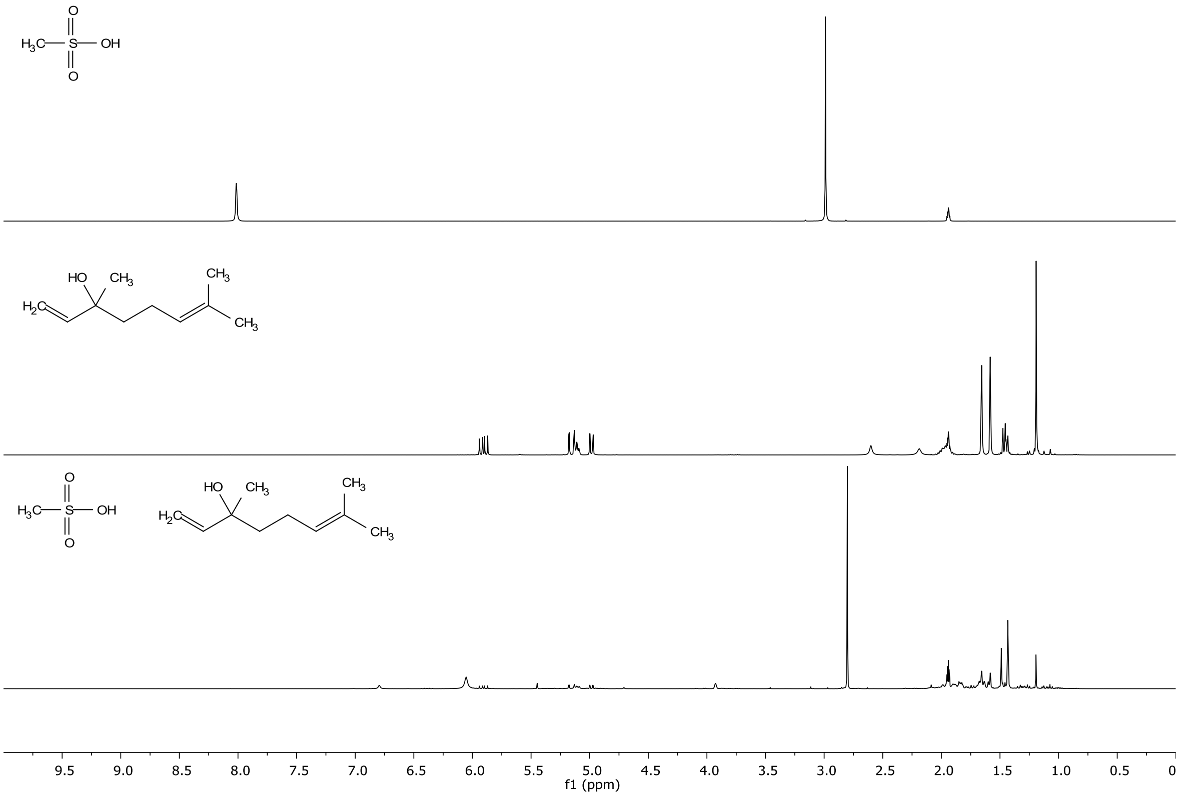

C12 NMR-Spectrum

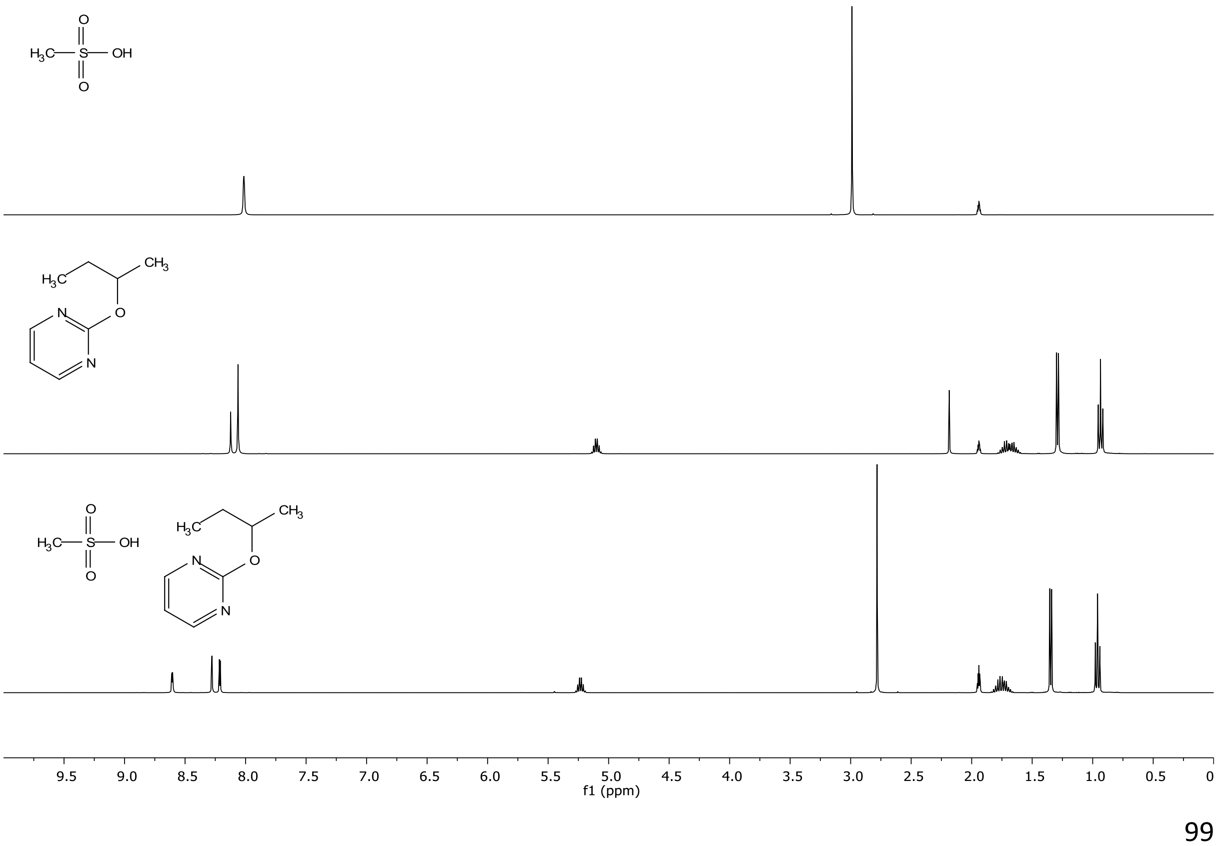

C13 NMR-Spectrum

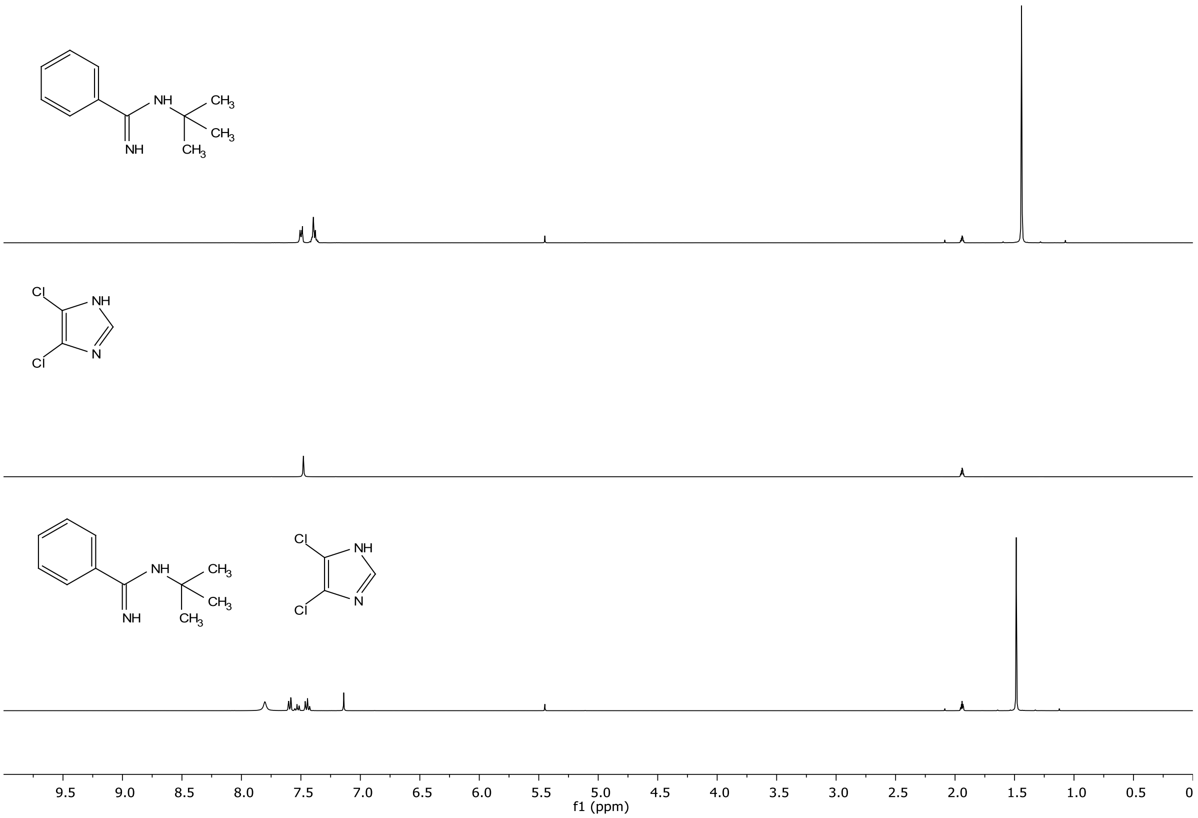

C14 NMR-Spectrum

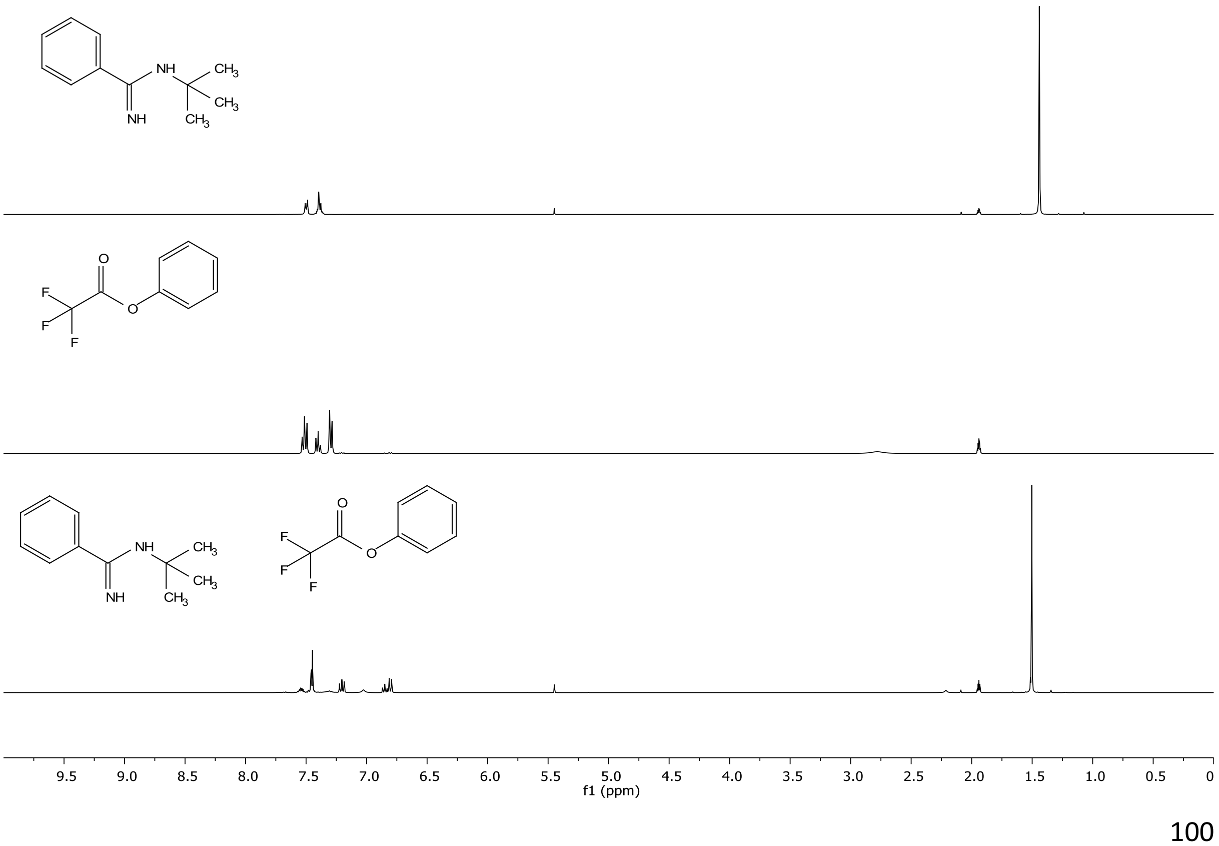

C15 NMR-Spectrum

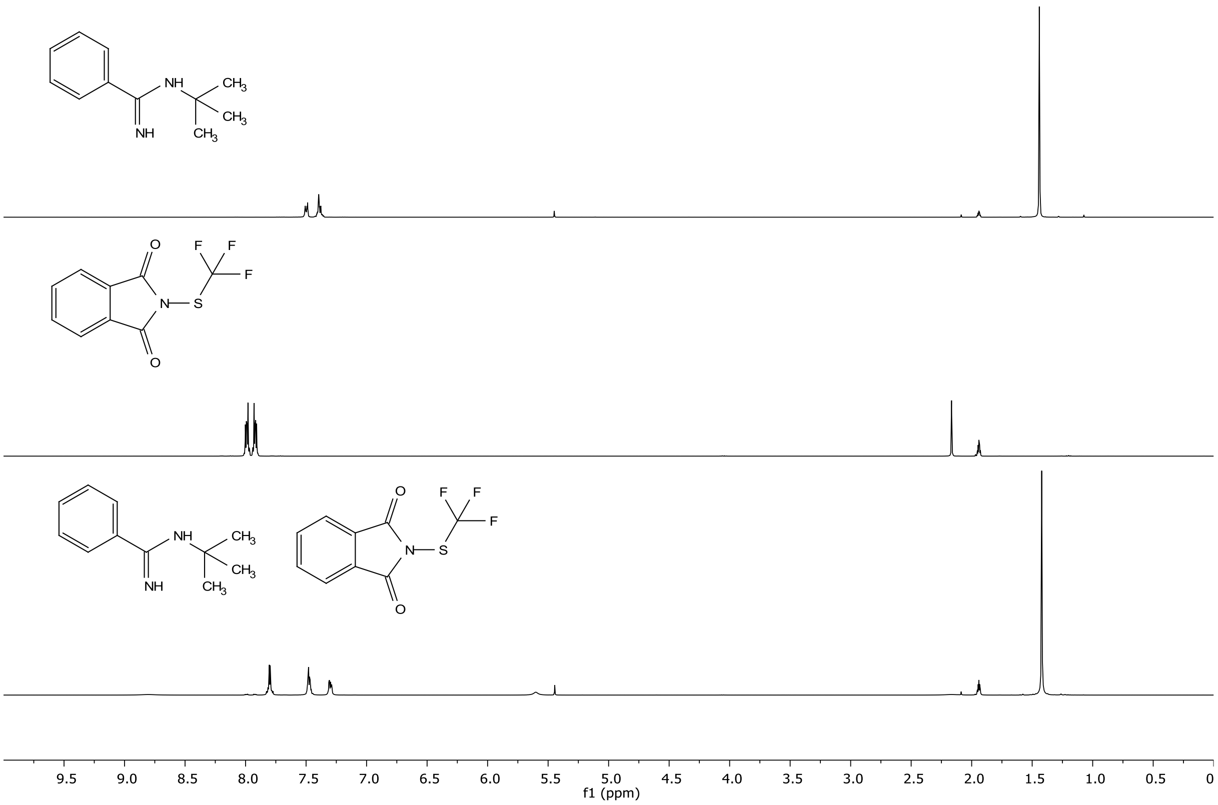

3.4.3 Interpretation

Table S26: Comparison of NMR-spectra of the cooperative pair with the NMR-spectra of the individual compounds. Summary of decreased, shifted (highest shift) and new peaks within the spectrum.

Pair N°	Signals of SMs decreased?	Signals shifted?	New Peaks?
**C1**	no	~0.01 ppm	no
**C2**	no	>0.40 ppm	no
**C3**	overlapping	~0.01 ppm	yes
**C4**	no	no	no
**C5**	no	~0.02 ppm	no
**C6**	no	>0.05 ppm	no
**C7**	(only signal of O*H*)	>0.10 ppm	no
**C8**	(only signal of O*H*)	~0.02 ppm	broad peak at 5.11 ppm
**C9**	no	~0.02 ppm	no
**C10**	no	no	no
**C11**	yes (**S22**)	no	yes
**C12**	no	>0.20 ppm	peak split (8.21 ppm to 8.21 and 8.61 ppm)
**C13**	no	>0.30 ppm	broad peak at 7.80 ppm
**C14**	yes (both)	no	yes
**C15**	yes (both)	no	yes

C1

Slight shift (low-field) of signals corresponding to aromatic hydrogen atoms of **S4** indicating a weak acceptor-donor interaction with **S38**.

C2

Strong shift (low-field) of signals corresponding to aromatic hydrogen atoms of **S4** as well as strong shift (high field) of signals corresponding to the methyl hydrogen atoms of **S95**, indicating a strong acid-base interaction of both substrates and a protonation of **S4** by **S95**.

C3

The occurrence of new signals in the NMR spectrum indicate the presence of an uncomplete chemical reaction between **S5** and **S88**, since signals of the starting materials are still visible .

C4

Since no change in the NMR spectrum is visible, the cooperative effect is probably induced by weak interactions between **S10** and **S88** (for details on this pair, 3.6).

C5

Slight shift (low-field) of signals corresponding to the alkene hydrogen atoms of **S10**, indicating a weak acceptor-donor interaction (reversible protonation) with **S95** (for details on this pair, 3.6).

C6

Moderate shift (low-field) of signals corresponding to the aromatic hydrogen atoms of the imidazole impurity, thereby indicating a weak acceptor-donor interaction with **S38**.

C7

Moderate shift (low-field) of signals corresponding to aromatic hydrogen atoms of **S15** as well as slight shift (high field) of signals corresponding to the aromatic hydrogen atom of **S38**, indicating a weak acceptor-donor interaction between the substrates.

C8

Slight shift (low-field) of signals corresponding to the aromatic hydrogen atoms of **S15**, indicating a weak acceptor-donor (H–bond) interaction with **S88**.

C9

Slight shift (low-field) of signals corresponding to the hydrogen atoms of the methyl groups of **S17** as well as slight shift (high field) of signals corresponding to the aromatic hydrogen atoms of **S38**, indicating a weak acceptor-donor interaction between the substrates.

C10

Since no change in the NMR spectrum is visible, the cooperative effect is probably induced by weak interactions between **S19** and **S88**.

C11

The occurrence of new signals in the NMR spectrum indicate the presence of an uncomplete chemical reaction between **S22** and **S95**, since signals of the starting materials are still visible. Because the signal corresponding to the methyl hydrogen atoms of **S95** is not reduced but highly shifted (high field), a protonation probably initiates the reaction.

C12

Strong shift (low-field) of signals corresponding to the aromatic hydrogen atoms of **S32** as well as strong shift (high field) of signals corresponding to the methyl hydrogen atoms of **S95** indicate a strong acid-base interaction between both substrates and a protonation of **S32** by **S95**. This assumption gets supported by the splitting of one signal corresponding to the two aromatic hydrogen atoms into two distinguished signals, thereby inducing a desymmetrization of the pyrimidine moiety of **S32**.

C13

Moderate shift (low-field) of signals corresponding to the hydrogen atoms of **S88** as well as strong shift (high field) of signals corresponding to the aromatic hydrogen atom of **S38** indicate an acid-base or H–bonding interaction between **S38** and **S88**.

C14

The occurrence of new signals in the NMR spectrum indicate the presence of a quantitative chemical reaction between **S48** and **S88** since no signals of the starting materials are still visible.

C15

The occurrence of new signals in the NMR spectrum indicate the presence of a quantitative chemical reaction between **S75** and **S88** since no signals of the starting materials are still visible.

3.5 Electrospray Ionization High Resolution Mass Spectrometry

3.5.1 Analytical Details

To prepare reference samples for every individual compound, 20 µL of the stock solution (0.2 M) were transferred to a glass vial (2.0 mL). To prepare samples of the cooperative pairs, 10 µL of both stock solutions were transferred to a glass vial (2.0 mL) and 1.0 mL of MeCN was added. All samples were mixed and measured within 24 h after preparation. Signals are quoted in *m*/*z* in a range between 100 and 1500. To clearly define the *m*/*z* of an expected product as "found" in the spectrum, the corresponding signal needs to have an intensity of >1.0% relative to the highest. In addition, the found mass should differ <0.0001 from the expected *m*/*z*.

3.5.2 Results

S4 High Resolution Mass Spectrum

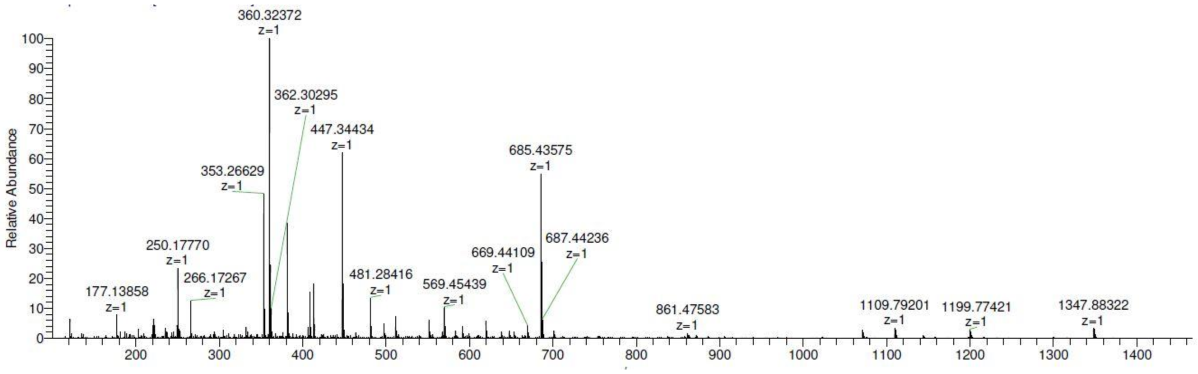

S5 High Resolution Mass Spectrum

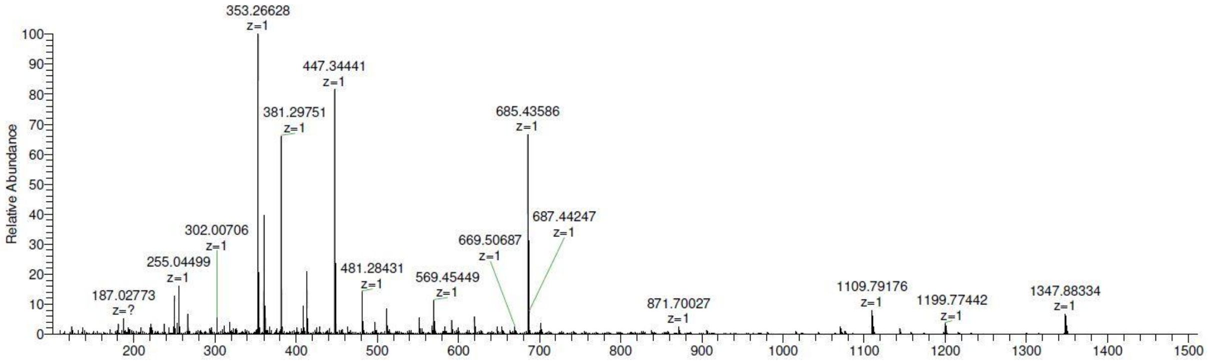

S10 High Resolution Mass Spectrum

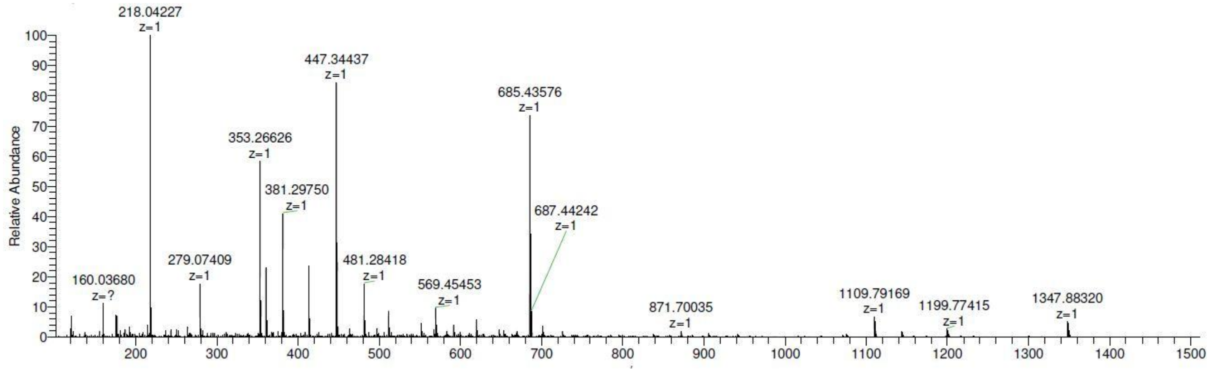

S11 High Resolution Mass Spectrum

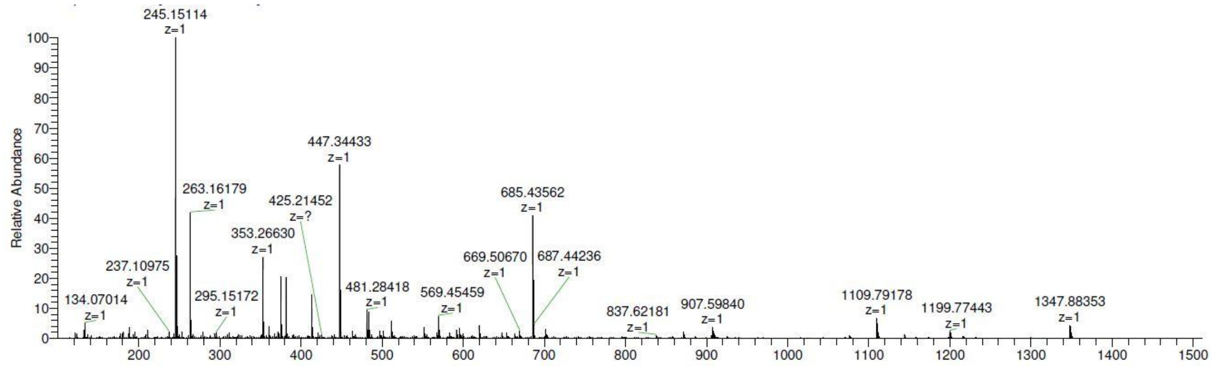

S15 High Resolution Mass Spectrum

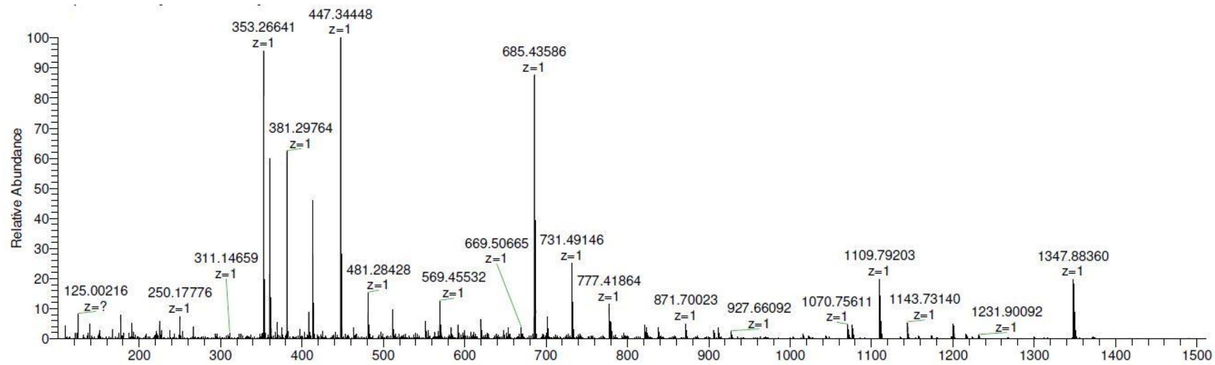

S17 High Resolution Mass Spectrum

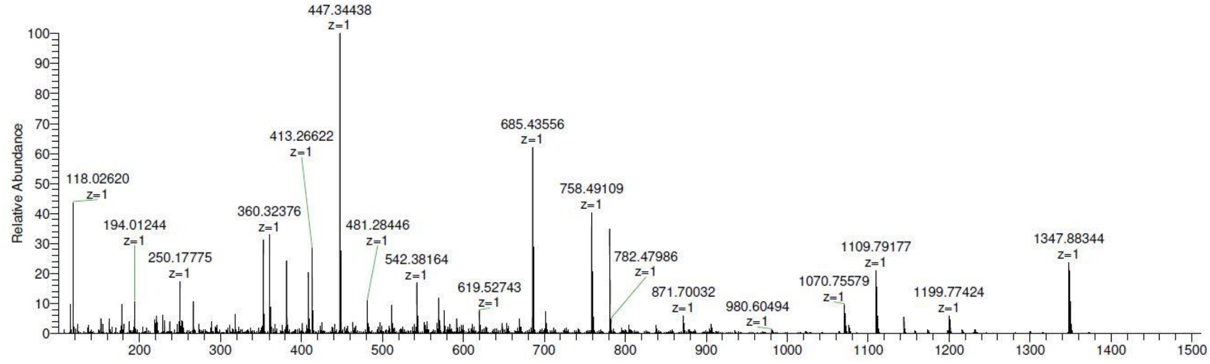

S19 High Resolution Mass Spectrum

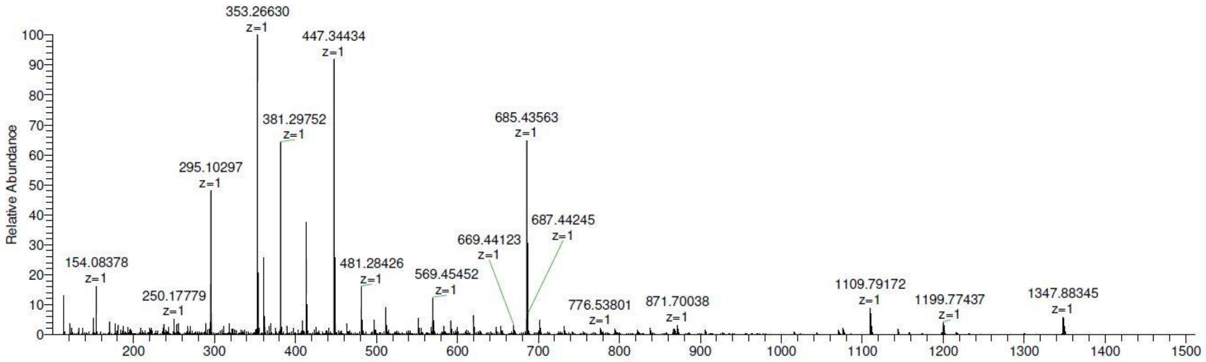

S22 High Resolution Mass Spectrum

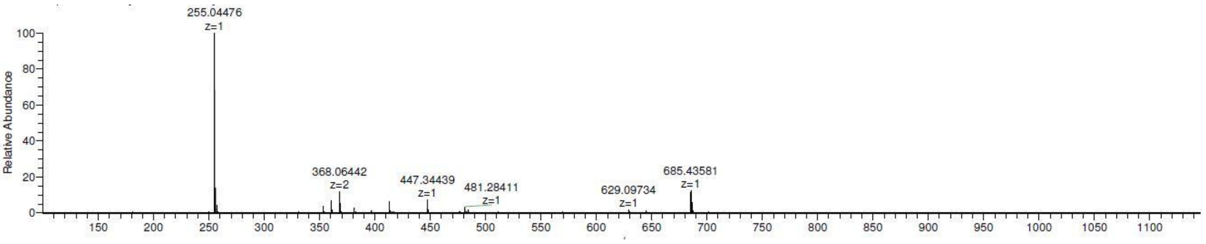

S32 High Resolution Mass Spectrum

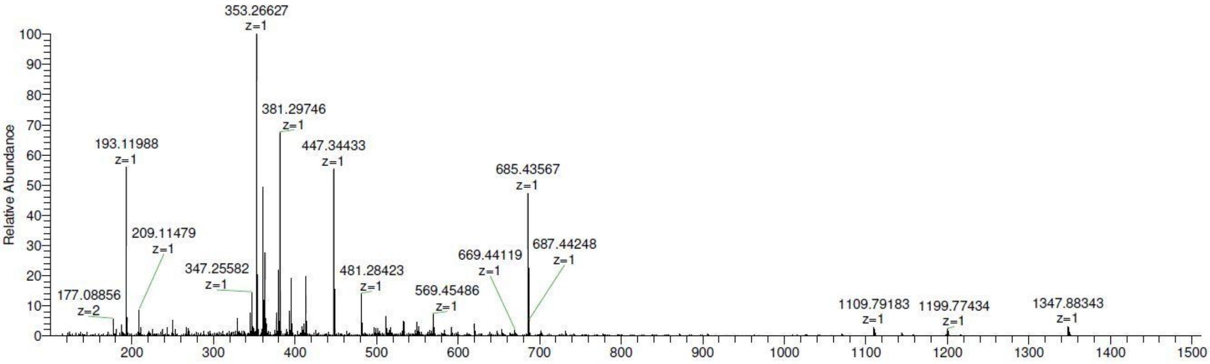

S38 High Resolution Mass Spectrum

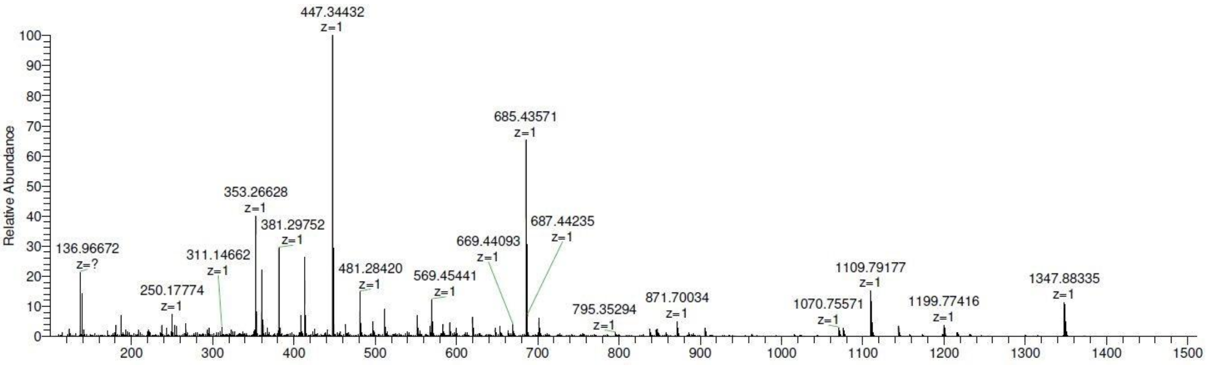

S48 High Resolution Mass Spectrum

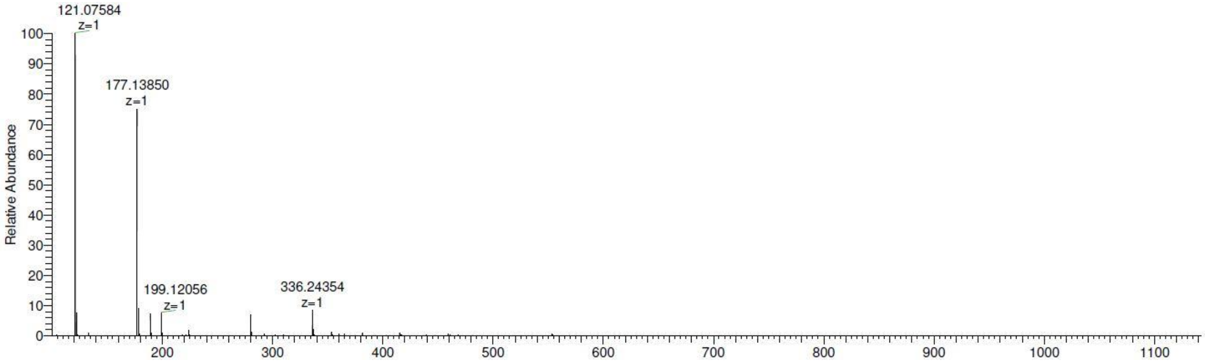

S75 High Resolution Mass Spectrum

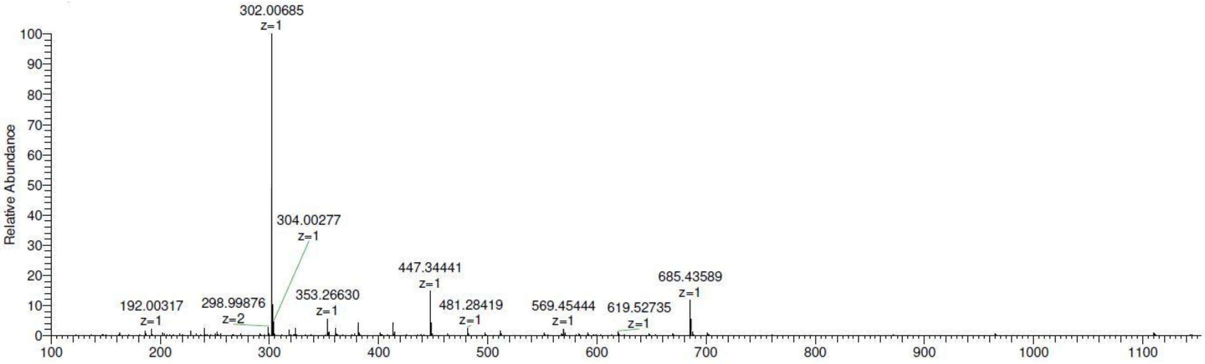

S88 High Resolution Mass Spectrum

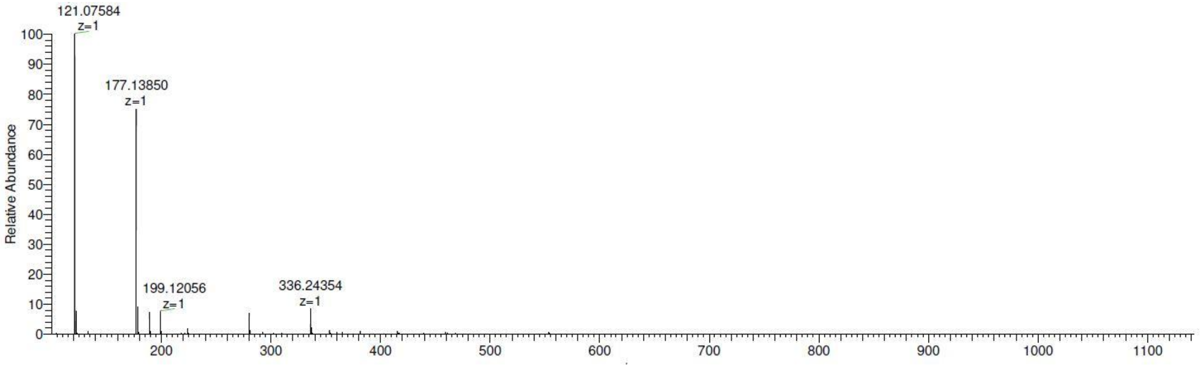

S95 High Resolution Mass Spectrum

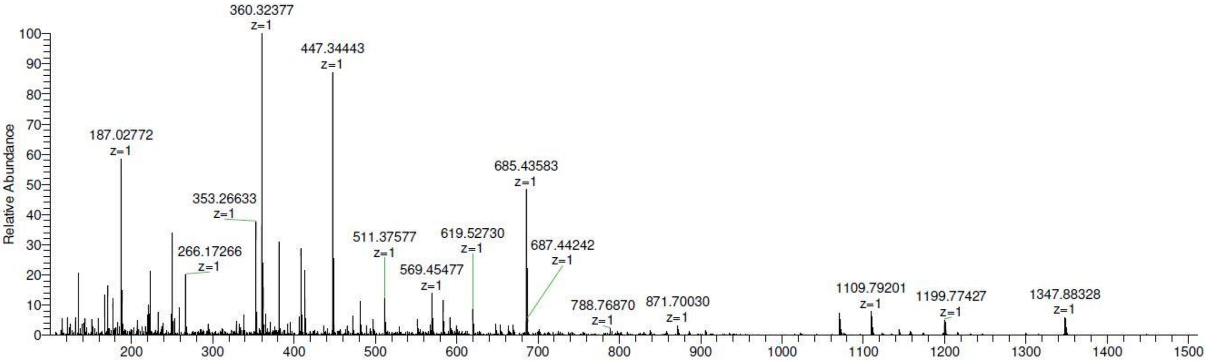

C1 High Resolution Mass Spectrum

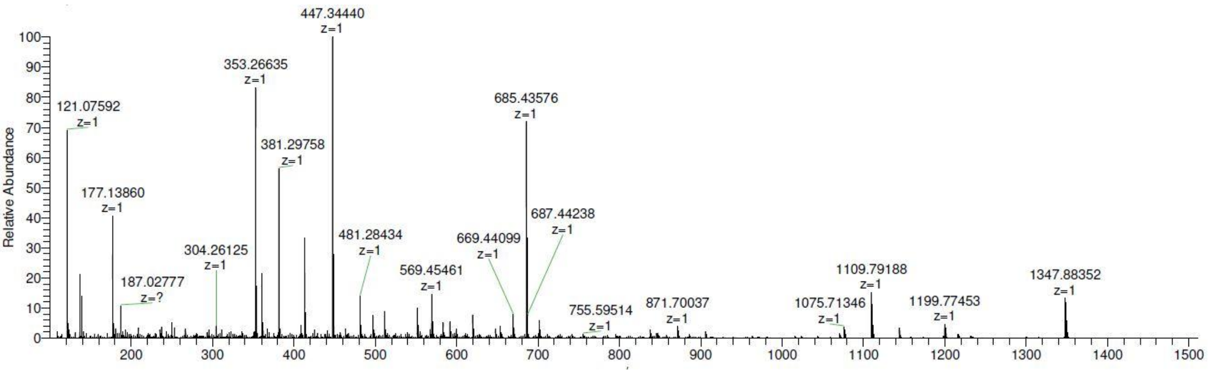

C2 High Resolution Mass Spectrum

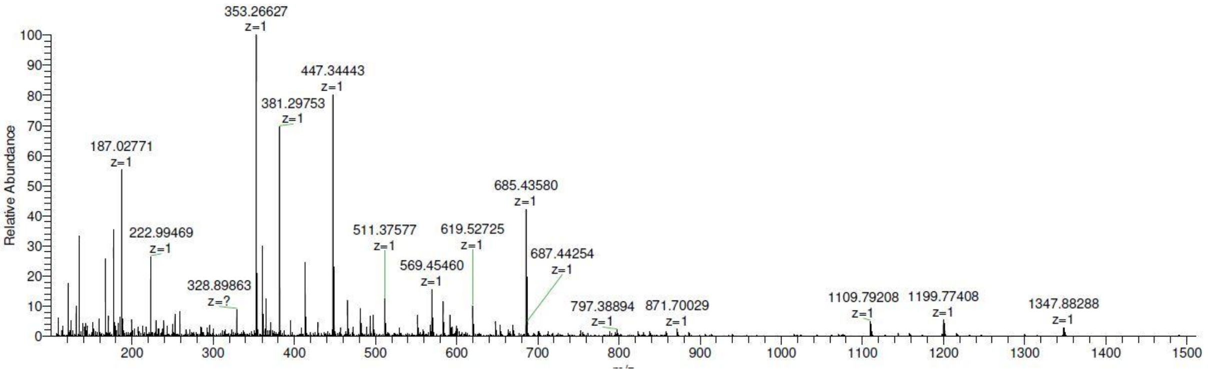

C3 High Resolution Mass Spectrum

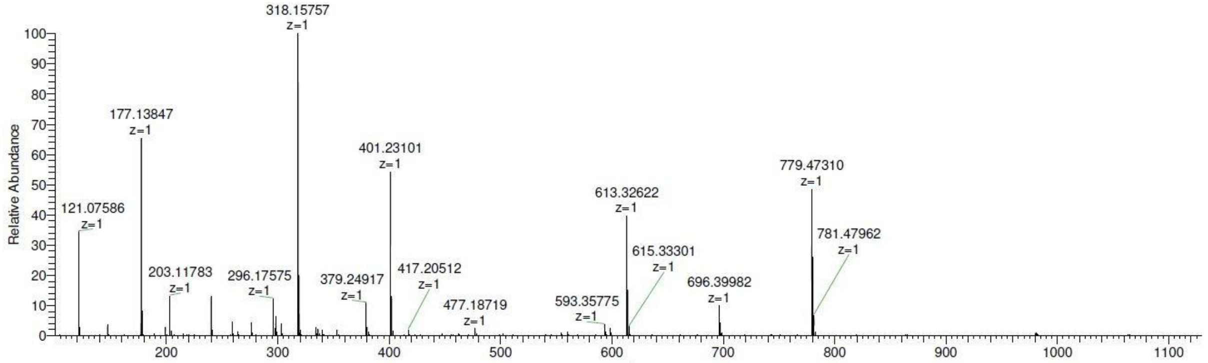

C4 High Resolution Mass Spectrum

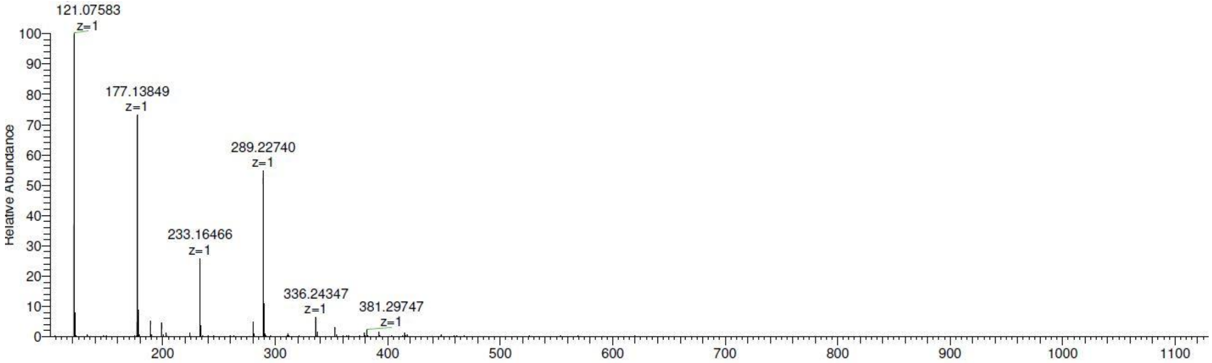

C5 High Resolution Mass Spectrum

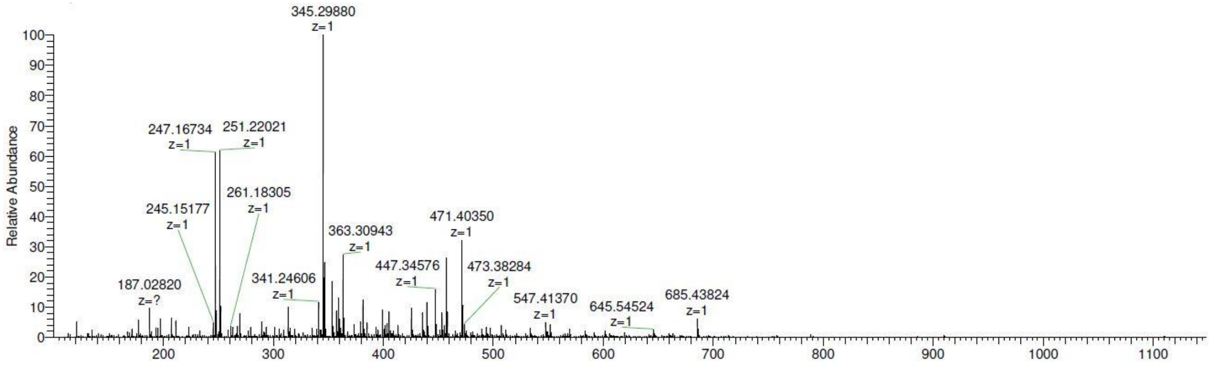

C6 High Resolution Mass Spectrum

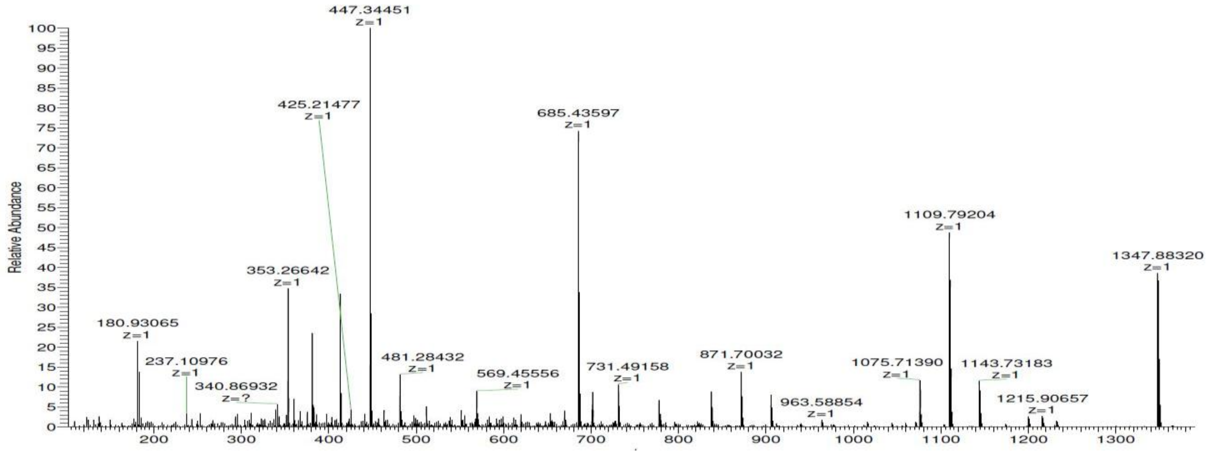

C7 High Resolution Mass Spectrum

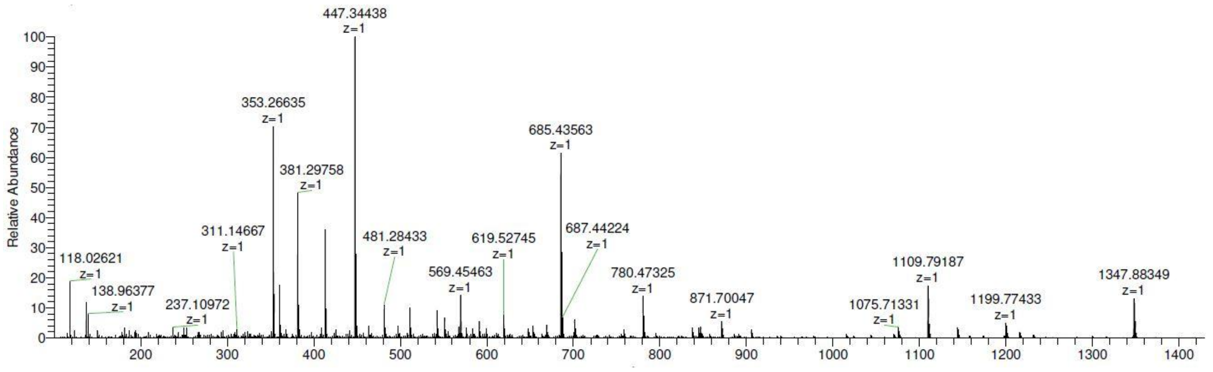

C8 High Resolution Mass Spectrum

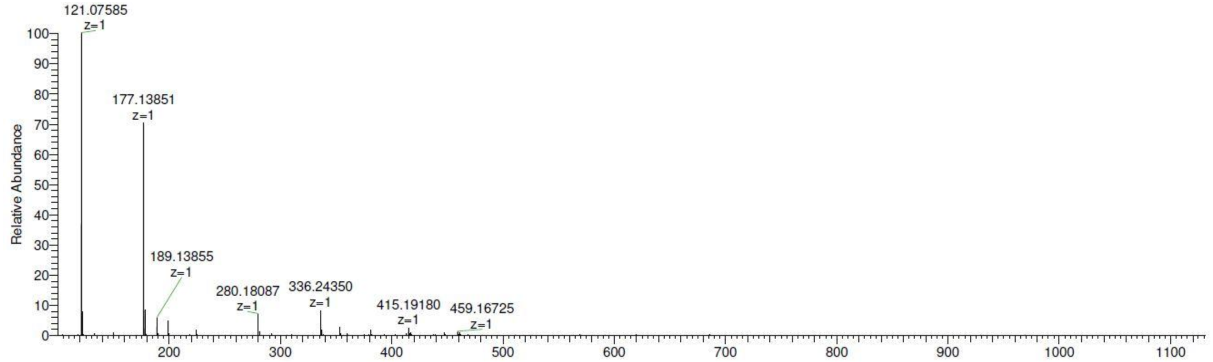

C9 High Resolution Mass Spectrum

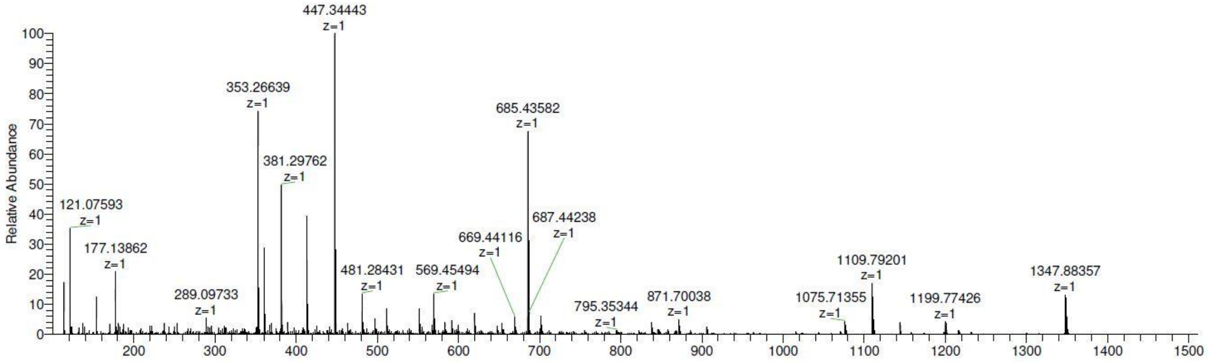

C10 High Resolution Mass Spectrum

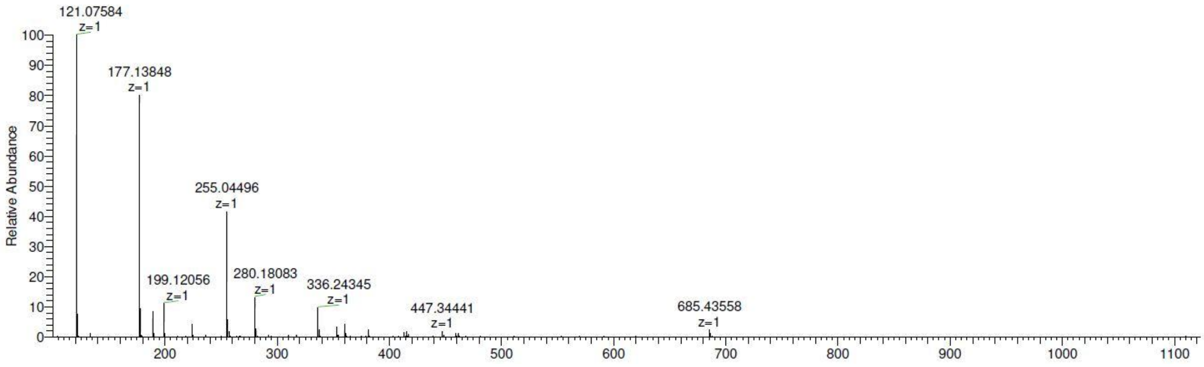

C11 High Resolution Mass Spectrum

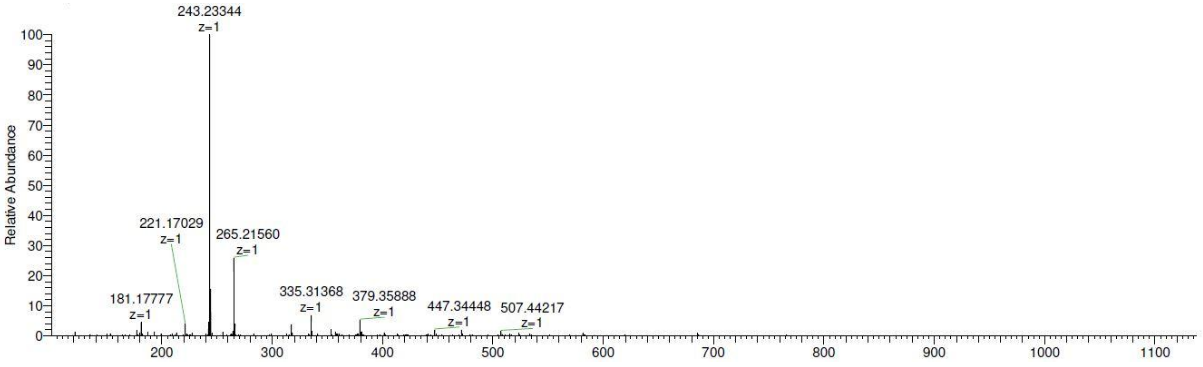

C12 High Resolution Mass Spectrum

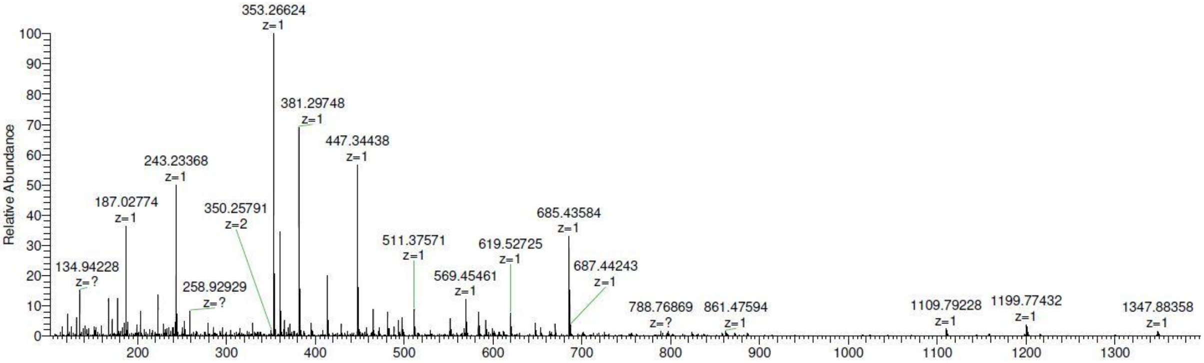

C13 High Resolution Mass Spectrum

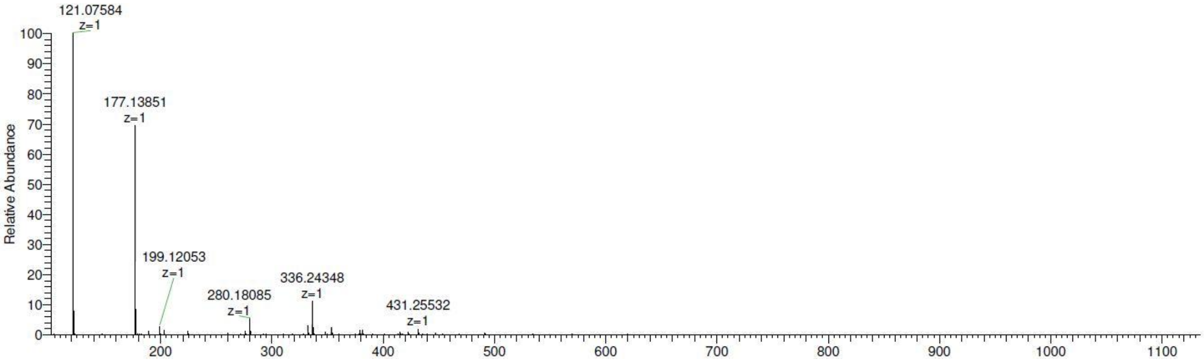

C14 High Resolution Mass Spectrum

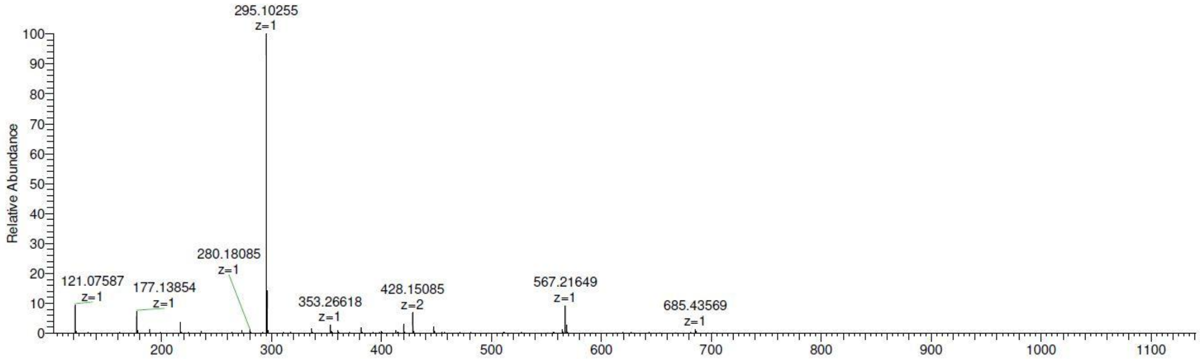

C15 High Resolution Mass Spectrum

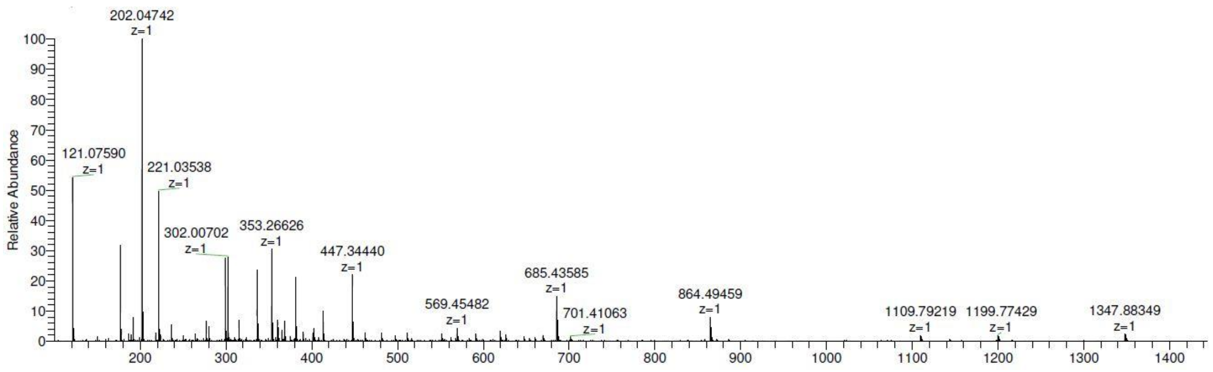

Table S27: Summary of relevant masses found in ESI-HRMS studies. Expected masses correspond to products suggested under 3.1.

Pair N°	SMs found?	Adduct found?	Formular of expected product?	Mass found?
**C1**	[**S38**+H]$^+$ /[**S4**+H]$^+$	no	-	-
**C2**	[**S4**+H]$^+$	no	-	-
**C3**	[**S88**+H]$^+$	no	$C_{18}H_{21}N_3O$	[M+H]$^+$/ [M+Na]$^+$
**C4**	[**S88**+H]$^+$	[**S88**+**S10**+H]$^+$	-	-
**C5**	[**S10**+H]$^+$	no	-	-
**C6**	no	no	-	-
**C7**	[**S38**+H]$^+$/[**S15**+Na]$^+$	no	-	-
**C8**	[**S88**+H]$^+$	no	-	-
**C9**	[**S38**+H]$^+$/[**S17**+H]$^+$	no	-	-
**C10**	[**S38**+H]$^+$/[**S19**+Na]$^+$	no	-	-
**C11**	no	no	-	-
**C12**	[**S32**+H]$^+$	no	-	-
**C13**	[**S88**+H]$^+$	no	-	-
**C14**	[**S88**+H]$^+$	no	$C_{12}H_{15}F_3N_2S$	[M+Na]$^+$
**C15**	[**S88**+H]$^+$	no	$C_{13}H_{15}F_3N_2O$	[M+H]$^+$

3.5.3. Interpretation

The formation of the expected products in case of **C3**, **C14** and **C15** was successfully verified by ESI-HRMS.

3.6 Conclusion of Origin Studies

To determine and classify the origin of cooperativity, results from GC-MS, TLC, NMR and ESI-HRMS experiments were taken into account (Table S28). GC-MS and TLC results were mainly used to identify and clarify the formation of new molecules formed due to chemical reaction between the cooperative pair. If a new peak is observed in the gas chromatogram as well as a new spot on TLC, we propose that the reaction product causes the quenching of the excited state photocatalyst **PC1**. In three of four of these cases, a product could be proposed and confirmed via ESI-HRMS. In three cases, the origin of cooperativity is a nucleophile/electrophile interaction between the substrates **C3**, **C14**, **C15** (Table 29, entry **C3**, **C14**, **C15**). In one case, an acid/base interaction causes a chemical reaction, forming an unidentified product (Table 29, entry **C11**). To distinguish between acid/base and weak acceptor/donor interactions, NMR was mainly taken into account. In cases in which strong shifts of signals corresponding to protons close to basic or acidic moieties were observed, we classify the cooperative interaction as acid/base interaction (Table 29, entry **C2**, **C11**). In these cases, an electron poor heteroarene gets protonated and thereby activated for reduction. In cases in which no to moderate shift of signals in the NMR-spectrum were observed, we propose a weak acceptor/donor interaction (Table 29, entry **C1**, **C6**, **C7**, **C8**, **C9**, **C10**, **C13**). Since it can hardly be distinguished between all possible weak interactions, we suspect mainly H-bonding and π-π-interactions to be at the origin of the shifts in the NMR as well as the observed change in quenching behaviour of the cooperative pairs. In two cases (Table 29, entry **C3**, **C4**), additional studies on various batches of **S10** led to the conclusion that an impurity within the substrate causes the cooperative effect to occur. In this case, the origin of cooperativity can barely be clarified. For the cooperative pair **C1**, the cooperative effect occurs between **S38** and imidazole (hydrolysis product of **S11**) based on the conducted NMR-studies.

Table S28: Results of the conducted GC-MS, TLC, NMR and ESI-HRMS experiments. Cells marked in yellow represent inconclusive results (weakly visible spots, slightly shifted signals, or single new and broad spots).

Pair N°	GC-MS	TLC	NMR		ESI
	New peak?	*New spot?*	*Signals shifted?*	*New Signals?*	*Product mass found?*
**C1**	no	(yes)	(yes)	no	no
**C2**	no	no	yes	no	no
**C3**	yes	yes	(yes)	yes	yes
**C4**	no	no	no	no	no
**C5**	no	no	(yes)	no	no
**C6**	no	no	yes	no	no
**C7**	no	no	yes	no	no
**C8**	no	no	(yes)	(yes)	no
**C9**	no	no	(yes)	no	no
**C10**	no	no	no	no	no
**C11**	yes	yes	no	yes	no
**C12**	no	no	yes	(yes)	no
**C13**	no	yes	yes	(yes)	no
**C14**	yes	no	no	yes	yes
**C15**	yes	yes	no	yes	yes

Table S29: Proposed cooperative interaction of every pair found in the conducted screening approach. Interaction types and products were proposed based on the conducted analytical experiments (**3.2** to **3.5**).

Pair N°	Pair Molecules	Proposed Cooperative Interaction	Proposed Product
**C1**	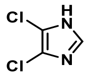 S38 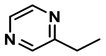 S4	**weak donor/acceptor** (H-bonding or π-π-interaction)	
**C2**	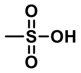 S95 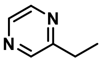 S4	**acid/base**	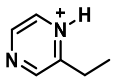
**C3**	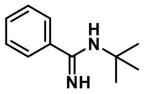 S88 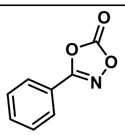 S5	**electrophile/nucleophile**	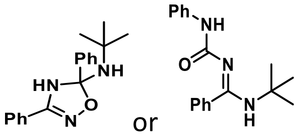
**C4**[a]	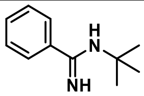 S88 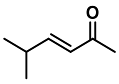 S10	unknown[a]	

**C5**[a]	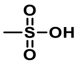 S95 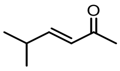 S10	unknown[a]	
**C6**[b]	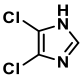 S38 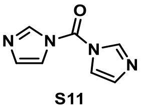 S11	**weak donor/acceptor** (H-bonding or π-π-interaction)[b]	
**C7**	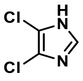 S38 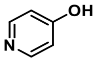 S15	**weak donor/acceptor** (H-bonding or π-π-interaction)	
**C8**	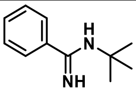 S88 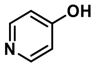 S15	**weak donor/acceptor** (H-bonding)	
**C9**	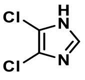 S38 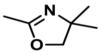 S17	**weak donor/acceptor** (H-bonding or π-π-interaction)	
**C10**	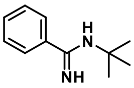 S88 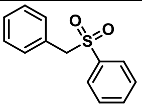 S19	**weak donor/acceptor** (H-bonding or π-π-interaction)	
**C11**	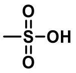 S95 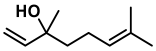 S22	**acid/base** followed by a chemical reaction of **S22**	unknown
**C12**	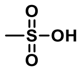 S95 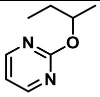 S32	**acid/base**	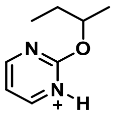
**C13**	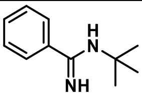 S88 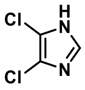 S38	**weak donor/acceptor** (H-bonding or π-π-interaction)	
**C14**	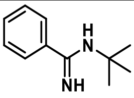 S88 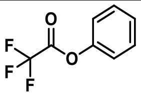 S48	**electrophile/nucleophile**	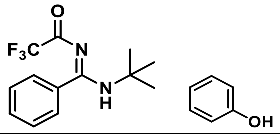
**C15**	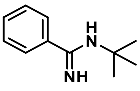 S88 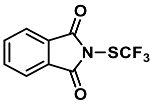 S75	**electrophile/nucleophile**	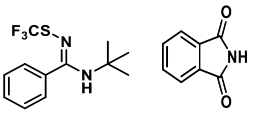

a) Additional quenching and NMR-studies with different batches of **S10** suggest that an impurity is responsible for the cooperative effect with **S88** and **S95**. Due to low reproducibility and the dependence on a specific batch of **S10**, this hit pair was not further investigated. b) Cooperative effect occurs between **S38** and imidazole (hydrolysis product of **S11**) based on the conducted NMR studies.

4. Investigation of the Change in Activity and Reactivity

4.1 Theoretical Procedure to Investigate the Fluorescence Quenching

For the investigation of the quenching mode, thereby the mode of activity, all individual substrates (Table S30) as well as the dual substrate combinations (Table S31) were tested for quenching of the fluorescence of $[Ir(ppy)_3]$ (**PC2**),[28] and $[Ru(bpz)_3][PF_6]_2$ (**PC3**)[30] as photocatalysts. Stock solutions of the photocatalysts (0.1 mM) and the substrates (250 mM) were prepared in MeCN. To ensure a good reproducibility and low errors during the quenching study, the fluorescence of photocatalysts was probed multiple times until reproducible intensities could be achieved. In doing so, 100 µL of the catalyst stock solution were pipetted into a cuvette and diluted with 900 µL of MeCN. To investigate the quenching mode, 100 µL of the respective catalyst and 100 µL of the substrates stock solution(s) were pipetted into the cuvette and diluted with 800 µL (for single compounds) or 700 µL (for dual substrate combinations) of MeCN, giving a 1:2500 ratio of photocatalyst:substrate. Since some individual compounds might also (partially) quench the luminescence of the chosen photocatalysts, the quenching (q) of those compounds was determined individually to carry out a background subtraction:

$$\Delta q(\%) = q(\%)_{pair} - (q(\%)_{substrate\ a} + q(\%)_{substrate\ b})$$

To determine the quenching mode of a cooperative pair, a threshold of $\Delta q(\%) > 20\%$ adjusted quenching was set. This was chosen since an error of up to 10% is expected in the conducted experiments on pairs and individual substrates (2.2.2). In additions systematical mistakes (e.g. catalyst deactivation, non-linearity at high quenching percentages, emerging side products) could lead to inconclusive results.

4.2 Results of Multi-Catalyst Quenching Studies

Table S30: Quenching of the luminescence of the photocatalysts **PC2** and **PC3** by the individual substrates. Values of moderate to strong quenching of individual substrates are marked (red), as they obstruct the evaluation of the subsequent dual experiments.

N°	Molecule	q_{PC2}	q_{PC3}	N°	Molecule	q_{PC2}	q_{C3}
**S4**	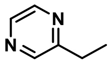	3%	1%	**S22**	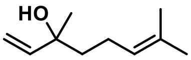	0%	4%
**S5**	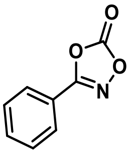	14%	10%	**S32**	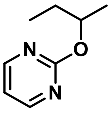	0%	2%
**S10**	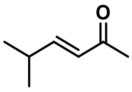	15%	2%	**S38**	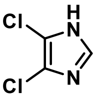	0%	15%
**S11**	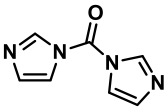	11%	41%	**S48**	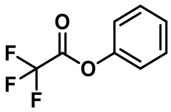	0%	19%
**S15**	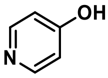	11%	12%	**S75**	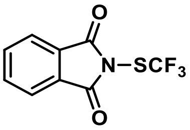	98%	2%
**S17**	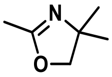	6%	3%	**S88**	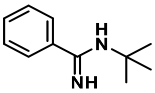	0%	66%
**S19**	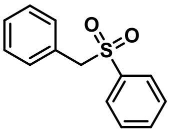	4%	16%	**S95**	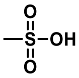	7%	96%

Table S31: Corrected quenching of the luminescence of the photocatalysts **PC2** and **PC3** by the cooperative pairs.

N°	Cooperative Pair	Δq_{PC2}	Δq_{PC3}	N°	Cooperative Pair	Δq_{PC2}	Δq_{PC3}
**C1**	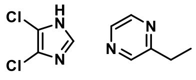 S38 S4	2%	32%	**C9**	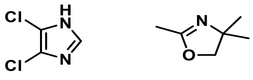 S38 S17	6%	34%
**C2**	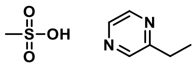 S95 S4	87%	-9%	**C10**	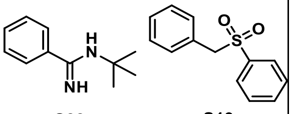 S88 S19	11%	-21%
**C3**	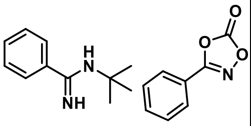 S88 S5	-7%	13%	**C11**	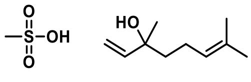 S95 S22	28%	-6%
**C4**[a]	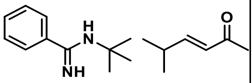 S88 S10	-7%	-6%	**C12**	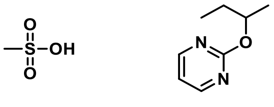 S95 S32	87%	-10%
**C5**[a]	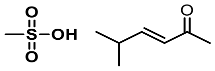 S95 S10	24%	-3%	**C13**	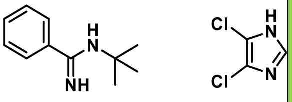 S88 S38	33%	12%
**C6**[b]	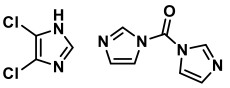 S38 S11	12%	-12%	**C14**	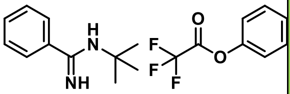 S88 S48	36%	-8%
**C7**	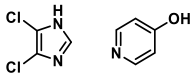 S38 S15	11%	5%	**C15**	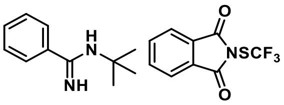 S88 S75	-1%	17%
**C8**	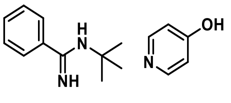 S88 S15	12%	-12%				

a) Additional quenching and NMR studies with different batches of **S10** suggest that an impurity is responsible for the cooperative effect with **S88** and **S95**. Due to low reproducibility and the dependence on a specific batch of **S10**, this hit pair was not further investigated. b) Cooperative effect occurs between **S38** and imidazole (hydrolysis product of **S11**) based on the conducted NMR studies.

4.3 Background for the usage of Cyclic Voltammetry Studies

Since the investigation of the photocatalysts quenching mode demonstrated that the observed cooperative interactions are predominated by a change in redox activity a more detailed study of these changes had to be conducted. Even though the fluorescence quenching of **PC1** was used to detect this change in redox or energy transfer activity (i.e. a change in the energy of the HOMO or LUMO)[31] it enables a broad variety of novel reactivities and reactions ranging from electrochemical over pure redox reactions to metal catalysed transformations. In addition, a change in the electrochemical properties goes hand in hand with changes in the electrophilicity/nucleophilicity of investigated substrate pairs. In order to quantify these changes of the redox properties for given cooperative pairs compared to the individual substrates cyclic voltammetry studies were carried out.

4.4 General Procedure for Cyclic Voltammetry Studies

Cyclic voltammetry experiments were conducted using a *CH Instruments CHI600E* potentiostat. The cell consists of the front-end of a platinum wire as the working electrode, a platinum crucible counter electrode and a silver/silver chloride reference electrode (Ag/AgCl, NaCl 3M). Voltammograms were measured for individual substrates **S4**, **S5**, **S11b**, **S15**, **S17**, **S19**, **S22**, **S32**, **S38**, **S48**, **S75**, **S88** and **S95**. Compound **S10** was excluded since the origin of cooperativity could not be determined but was traced back to an impurity in a specific batch of this compound. Substrate **S11** was replaced by imidazole (**S11b**) which is a major impurity in Carbonyldiimidazole (**S11**) and gets released via hydrolysis. Based on prior NMR studies (3.4) it can be assumed that **S11b** is responsible for the cooperative interaction. Voltammograms were measured for cooperative pairs **C1-C3**, **C6**, **C7-C15**. Pair **C4** and **C5** were excluded due to prior studies on impurities in **S10**. The examined substances (0.01 mmol) or substance pairs (0.01 mmol, each) were investigated in a solution of tetrabutylammonium hexafluorophosphate in acetonitrile (2.0 mL, 0.1 M) using a scan speed of 0.1 V/s. For each individual substrate and cooperative pair two measurements were carried out to detect potentials of reducible and oxidizable species matching the potential of **PC1**. Thereby, the first measurement was conducted to measure any substrate oxidation in the range of 0.00 V to 2.00 V, while a second measurement was carried out to measure any substrate reduction in a range of 0.00 V to –2.00 V, thereby covering the potentials of the photocatalyst (Table S31 to 34).[18] For every substrate and pair three full cycles between the mentioned potentials were measured. Datapoints from the second and third cycles were used to calculate redox potentials (4.5). The second cycle was used to approximate the transferred charge (4.6) and to determine reversibility of the redox

processes (4.6). In any case a solution of tetrabutylammonium hexafluorophosphate in acetonitrile (2.0 mL, 0.1 M) was measured as blank which was subtracted from every measurement. Voltammograms (Table S31 to S34) are given for the second scan.

4.5 Investigation on Redox Potentials and Transferred Charges

4.5.1 General Procedure for the Determination of Redox Potentials and Charges

As indicated by quenching experiments using **PC1** to **PC3** most cooperative pairs show a change in redox activities. Two major reasons for this could be either a shift in the redox potential towards accessible potentials or a change in the overall transferred charge. While the first could be enabled by a change in the (electronic) structure of a substrate (e.g. by the formation of H-, pi-, or covalent bonds) and consequently a shift in the HOMO or LUMO energy the second could be achieved by the shift of a chemical equilibrium towards a redox active species (e.g. an acid-base equilibrium or a tautomerization) which is enforced by the cooperative partner. In general, redox potentials ($E^{1/2}$) are determined as half-peak potentials, meaning that the potential at the half-height of the highest measured current was determined on the background-subtracted voltammogram. Thereby, a threshold of 25.0 µA was applied for the determination of the highest measured current. If the overall current is below this threshold, it can be assumed that the determined potential will be highly erogenous and is not usable for further comparisons. In cases where the highest current was detected above 1.95 V or below −1.95 V a further measurement was conducted cycling to higher voltages (2.50 V, oxidation potentials) or lower voltages (−2.50 V, reduction potentials). The half peak potential was determined as average from the second and third cycle (i.e. the third and fifth segment) if a clear maximum was recognizable in the third cycle. Since most examined redox processes are irreversible, a determination of the half peak potential for the back oxidation or reduction would lead to incomparable results. To compare the results with given potentials of the photocatalysts **PC1** to **PC3**, the potential was recalculated against the saturated calomel electrode (SCE) (Ag^+/Ag = −0.04 V against SCE).[32]

$$E^{SCE} = E^{measured(Ag+/Ag)} - 0.04\,V$$

In cases where multiple peaks are visible the redox potential was determined manually and individually for every peak, even though it has to be mentioned that an accurate determination is not possible in these cases while only qualitative information can be derived for the cooperative interaction. Differences in the half peak potentials as given in Table S37 and S38 were calculated as minimal difference of the potential determined for the pair compared to the

closest peak recognized for any corresponding substrate. For the determination of the transferred electric charge, all voltammograms were background subtracted while the third segment was integrated. As a scan-speed of 0.1 V/s was applied therefore a time frame of 20 s is used for integration. Q^{ox} refers to the charge as calculated from voltammograms measured in a positive scan mode between 0.00 V and 2.00 V while Q^{red} refers to charge as calculated from voltammograms measured in a negative scan mode between 0.00 V and –2.00 V. For general simplification and better comparability of the results, only the absolute values of the charges are taken into account. In any cases a single electron transfer process is to be assumed:

$$Q_{S0/S\pm} = |\int_0^{\pm 2} I(t)\, dt\,|$$

In between all measurements the electrodes were cleaned ensuring that the effective surface area stays similar, thereby giving comparable results. Q^{redox}_{Coop} corresponds to the transferred charge of the cooperative pair while Q^{redox}_{Sa} and Q^{redox}_{Sb} relate to the values for both individual substrates. Q^{redox}_{Add} is calculated as sum of Q^{redox}_{Sa} and Q^{redox}_{Sb} thereby giving the expected capacity in a purely additive case. By this means $Q^{redox}_{C\text{-}Add}$ gives the absolute capacity caused by cooperative effects while $Q^{red}_{C}/Q^{red}_{Add}$ relates to the relation ratio of overall observed and calculated additive transferred charge. Thereby a value $Q^{red}_{C}/Q^{red}_{Add} > 1$ corresponds to a positive cooperative effect with regard to the transferred charge while a value $Q^{red}_{C}/Q^{red}_{Add} < 1$ corresponds to a negative cooperative effect with regard to the transferred charge.

4.5.2 Results on the Oxidation of Substrates and Pairs

Individual Substrates

Table S31: Voltammograms of different non-quenching substrates (**S4**, **S5**, **S11b**, **S15**, **S17**, **S19**, **S22**, **S32**, **S38**, **S48**, **S75**, **S88** and **S95**) recorded between 0.00 V and 2.00 V (blue, y-scale between −10 µA and 210 µA); referenced to the silver/silver chloride electrode.

S4

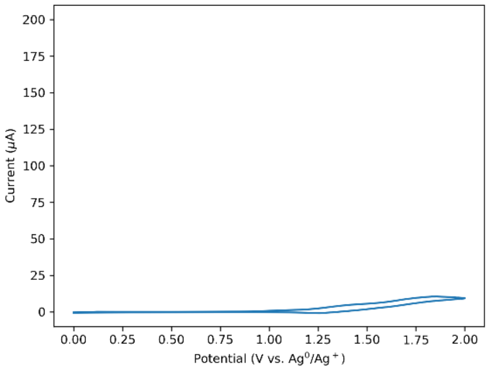

S5

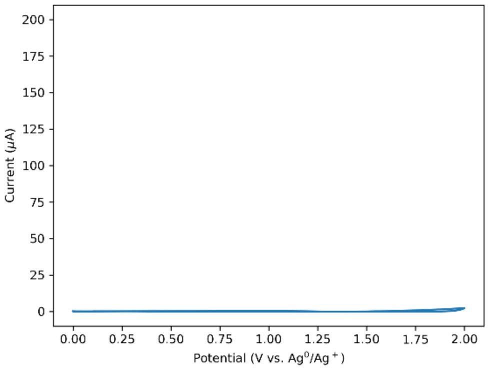

S11b

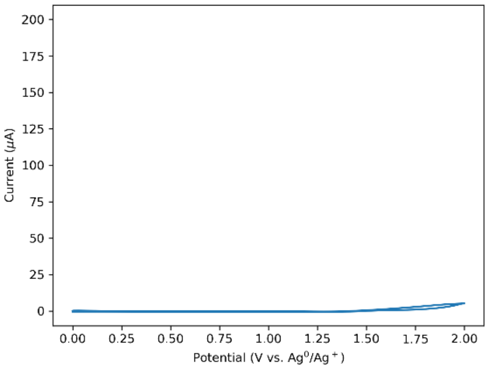

S15

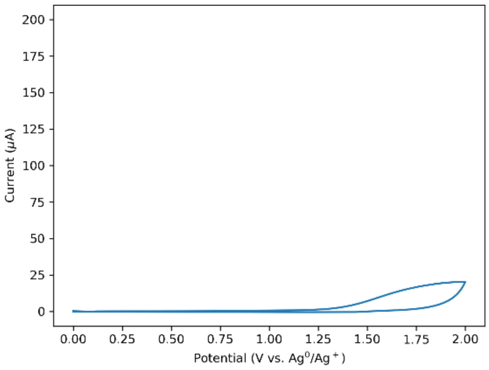

S17

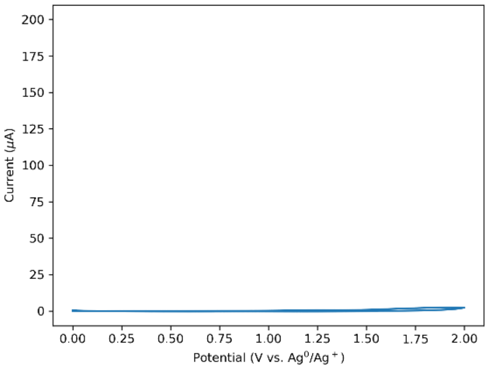

S19

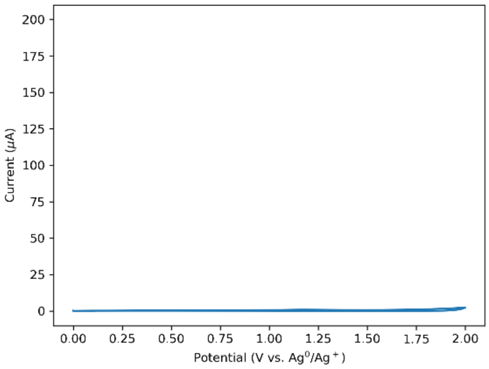

S22

S32

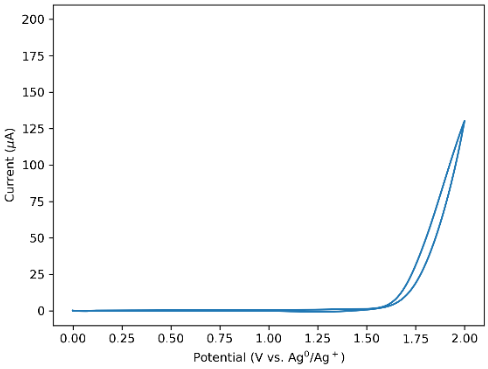

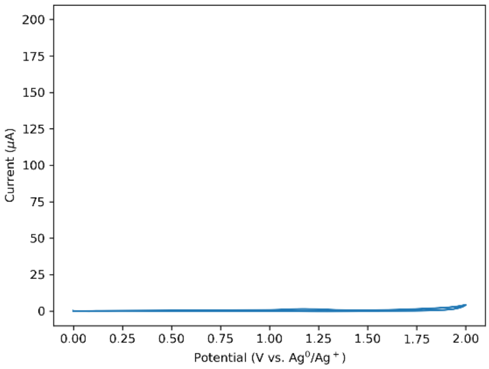

S38

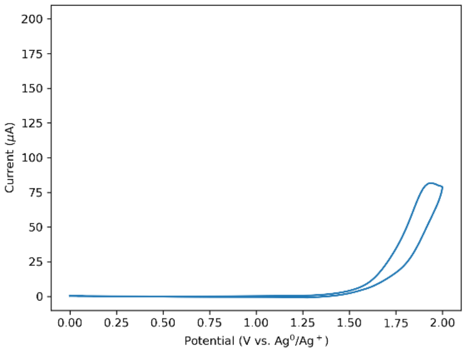

S48

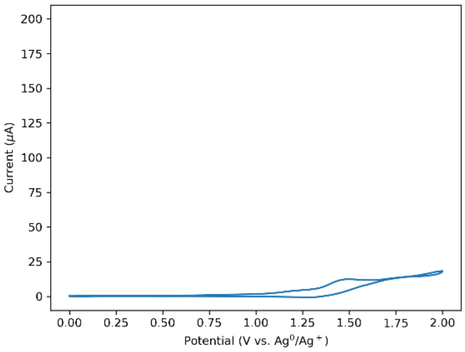

S75

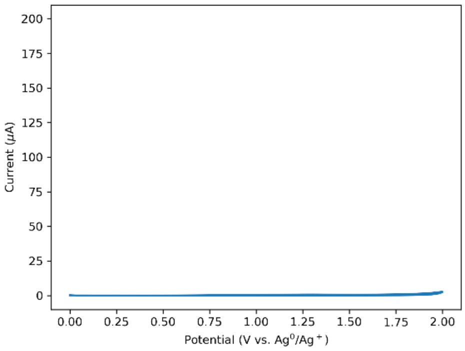

S88

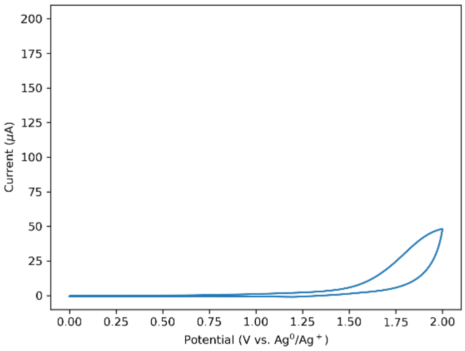

S95

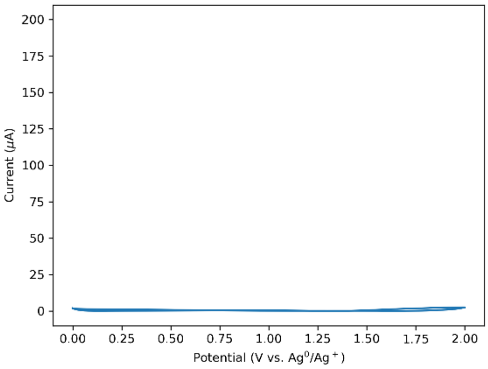

Cooperative Pairs

Table S32: Voltammograms of different cooperative pairs (**C1**, **C2**, **C3**, **C6**, **C7**, **C8**, **C9**, **C10**, **C11**, **C12**, **C13**, **C14** and **C15**.) recorded between 0.00 V and 2.00 V (blue, y-scale between −10.0 µA and 210 µA); referenced to the silver/silver chloride electrode.

C1

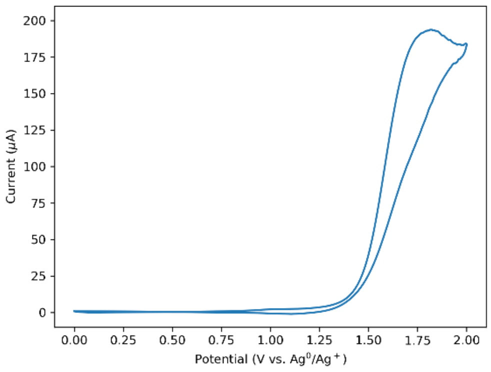

C2

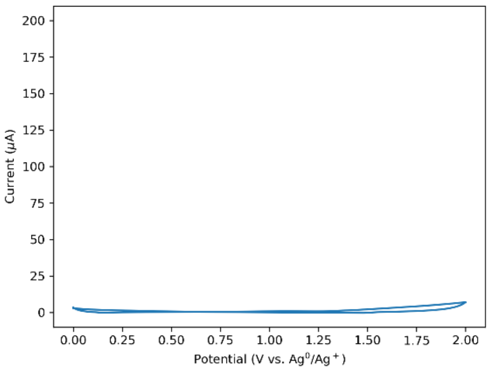

C3

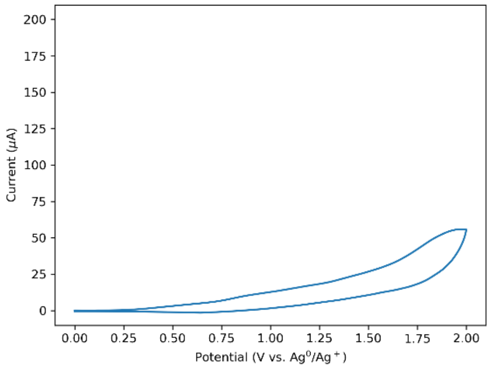

C6

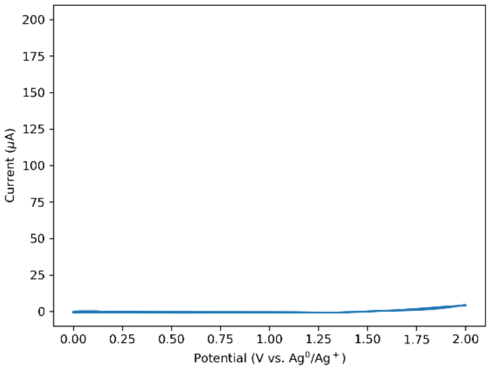

C7

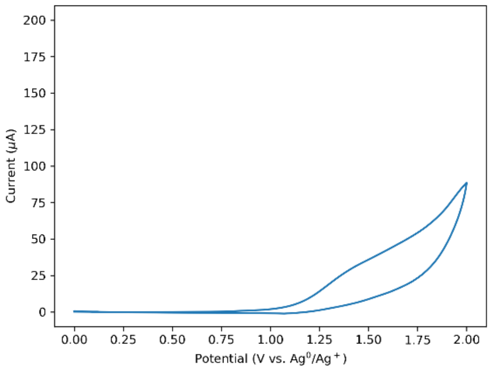

C8

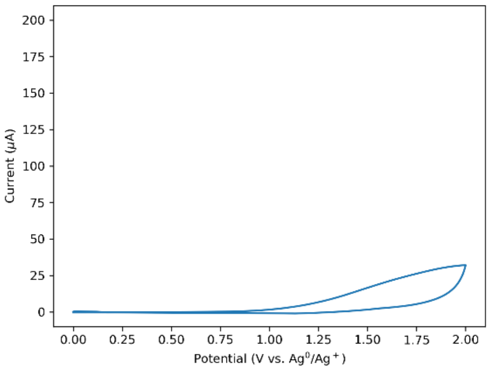

C9

C10

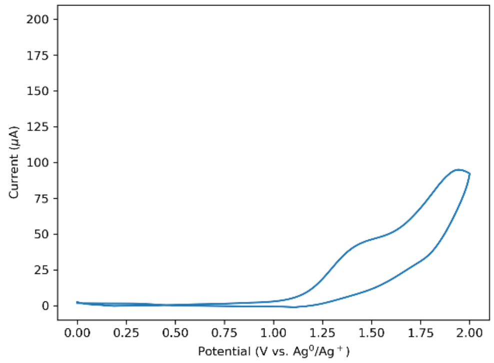

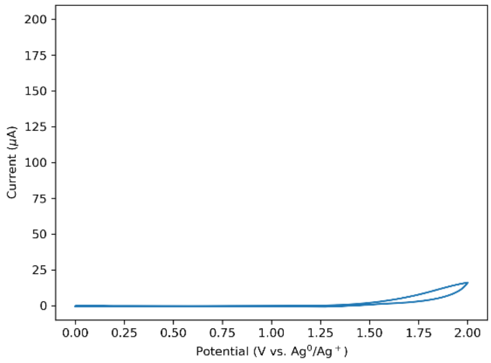

C11

C12

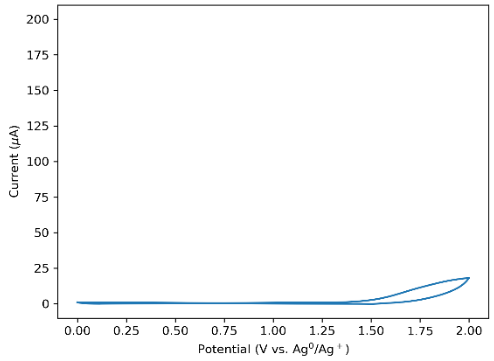

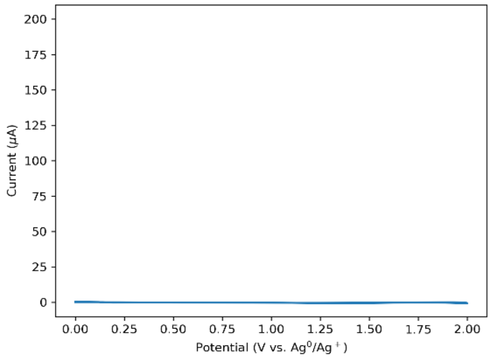

C13

C14

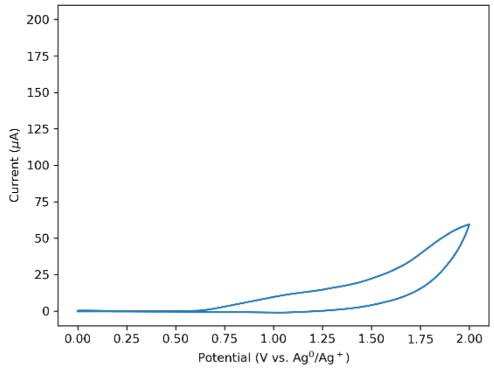

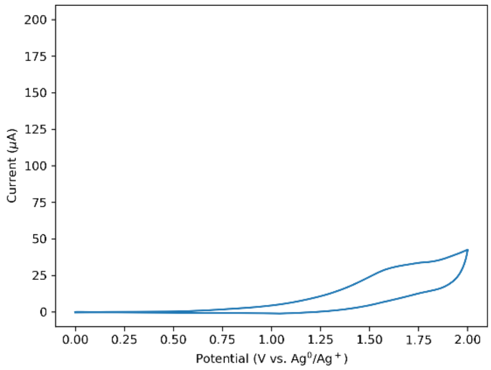

C15

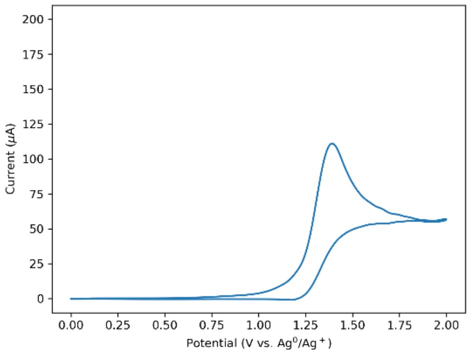

4.5.3 Results on the Reduction of Substrates and Pairs

Individual Substrates

Table S33: Voltammograms of different non-quenching substrates (**S4**, **S5**, **S11b**, **S15**, **S17**, **S19**, **S22**, **S32**, **S38**, **S48**, **S75**, **S88** and **S95**) recorded between 0.00 V and −2 .00 V (blue, y-scale between −10.0 µA and 210 µA); referenced to the silver/silver chloride electrode.

S4

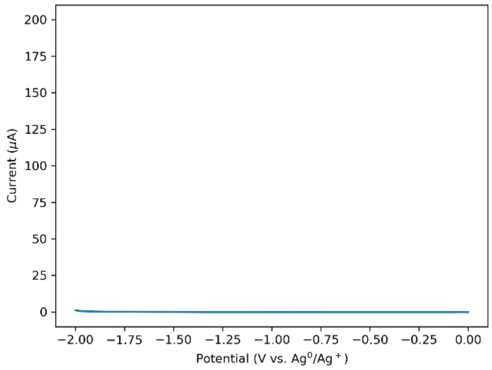

S5

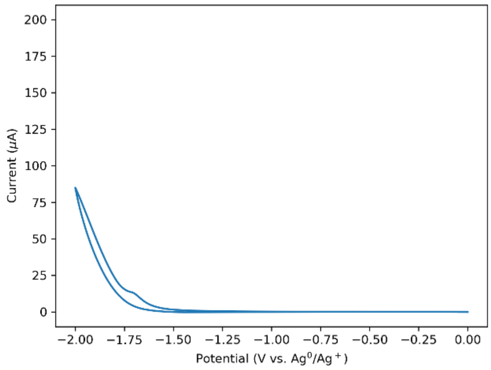

S11b

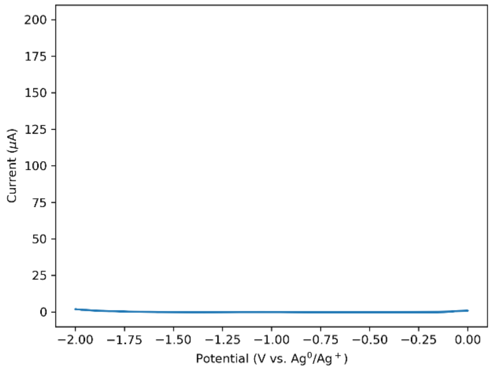

S15

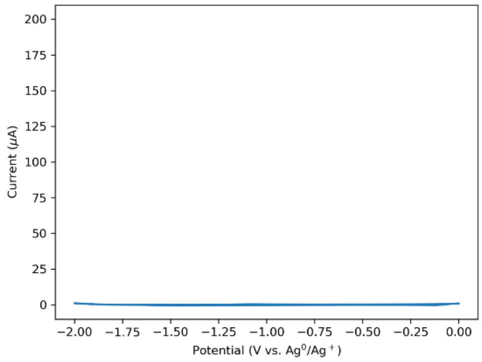

S17

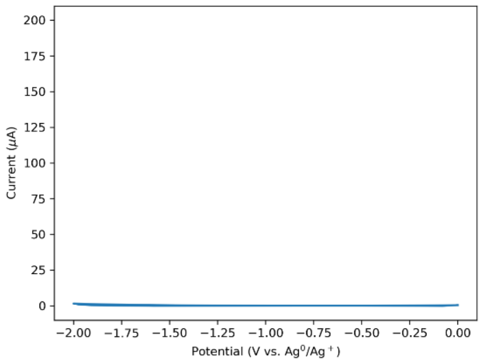

S19

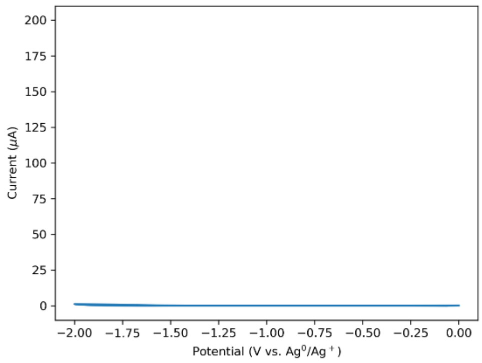

S22

S32

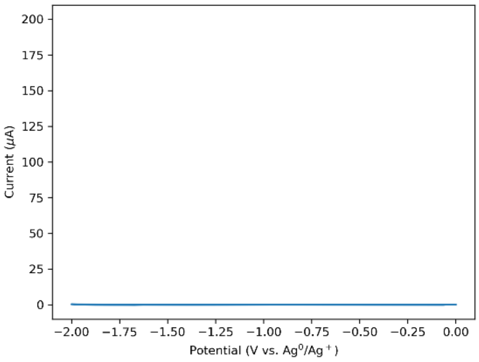

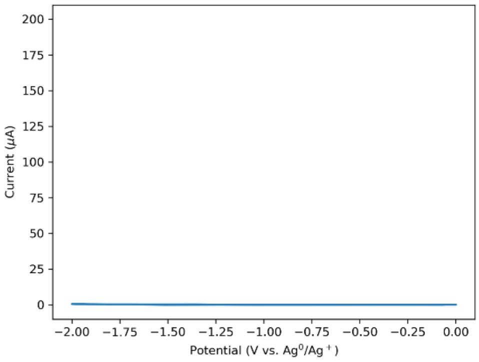

S38

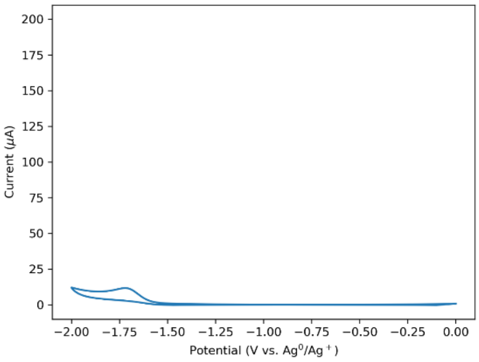

S48

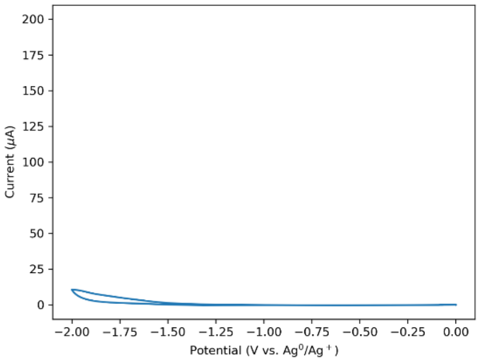

S75[a]

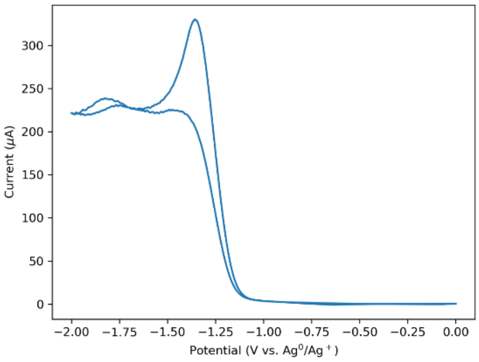

S88

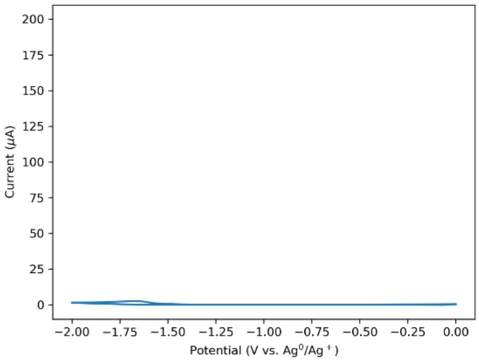

S95

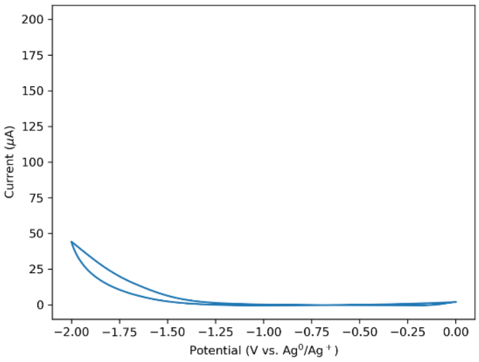

[a)]Scale between −10 μA and 375 μA.

Cooperative Pairs

Table S34: Voltammograms of different cooperative pairs (**C1**, **C2**, **C3**, **C6**, **C7**, **C8**, **C9**, **C10**, **C11**, **C12**, **C13**, **C14** and **C15**.) recorded between 0.00 V and −2.00 V (blue, y-scale between −10.0 µA and 210 µA); referenced to the silver/silver chloride electrode.

C1

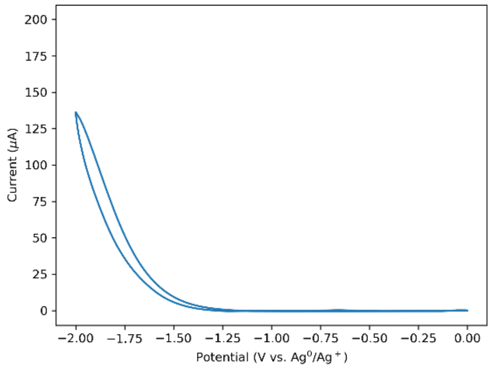

C2

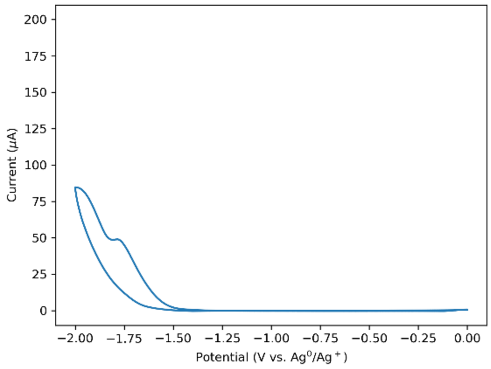

C3

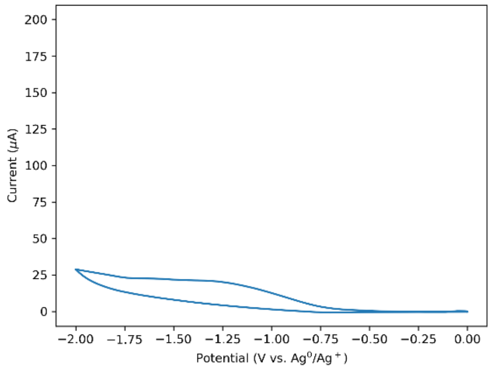

C6

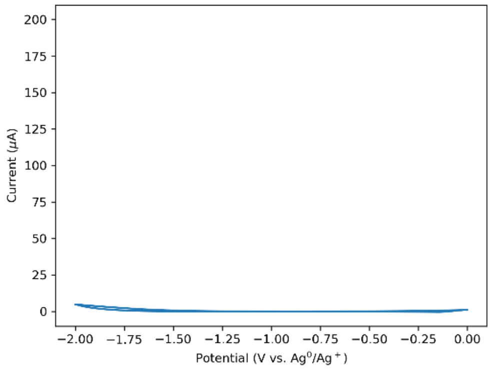

C7

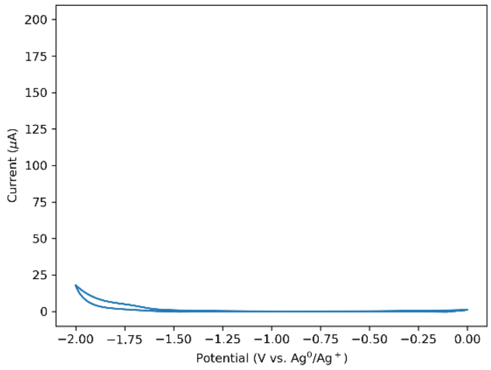

C8

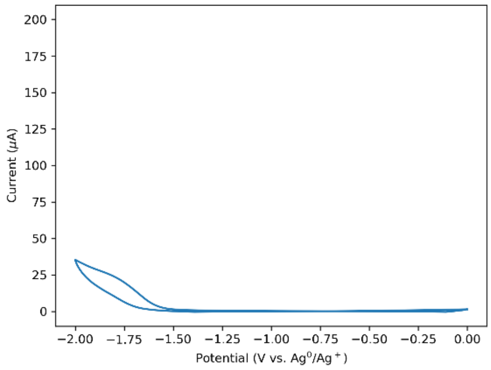

C9

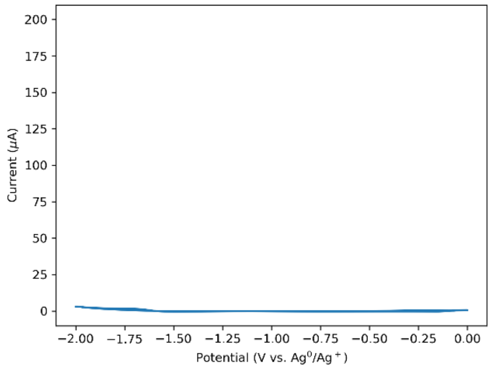

C10

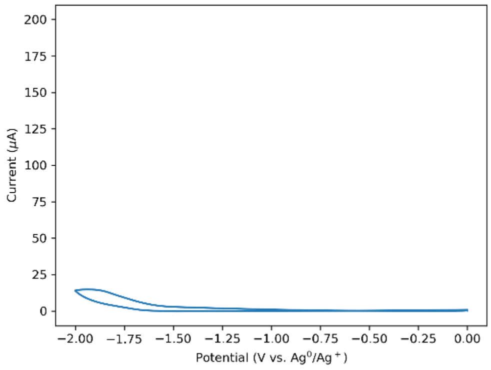

C11

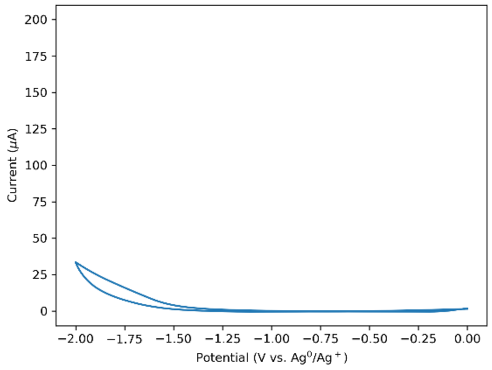

C12

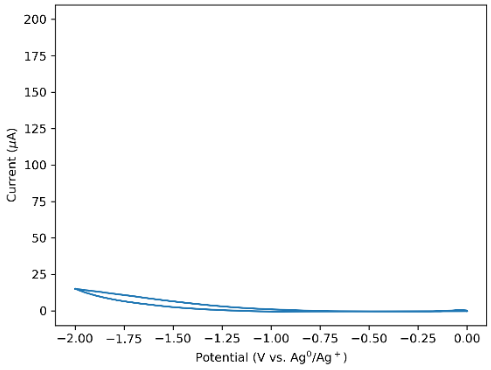

C13

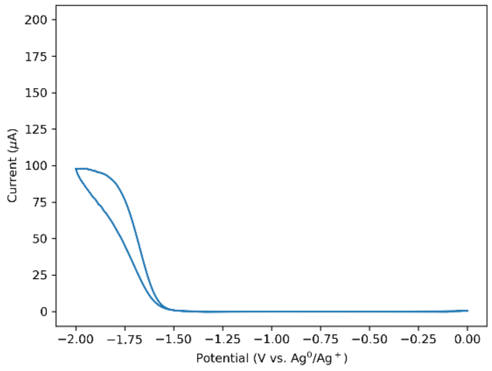

C14

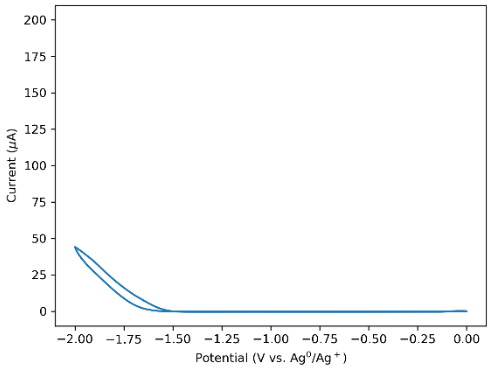

C15

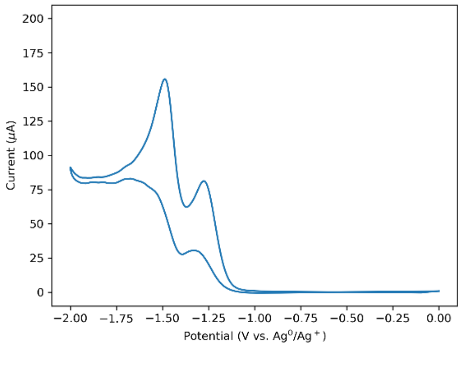

4.5.3 Comparison and Conclusion

Table S35: Comparison of voltammograms of the cooperative pairs and their corresponding individual compounds between 0.00 V and 2.00 V (blue, y-scale between −10.0 μA and 210 μA).

S4	*C1*	S38
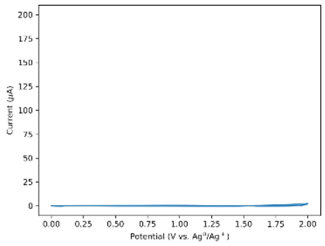	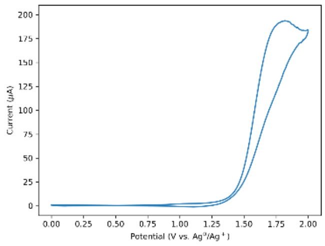	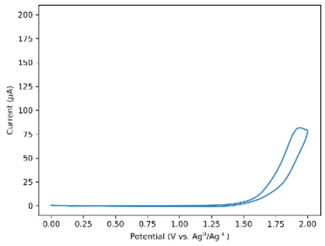
**S4**	***C2***	**S95**
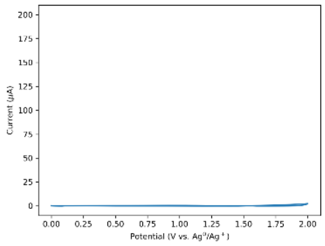	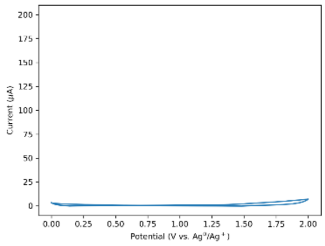	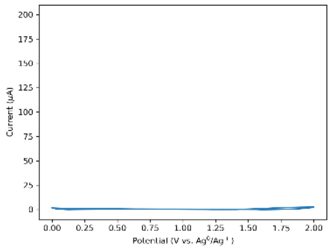
**S5**	***C3***	**S88**
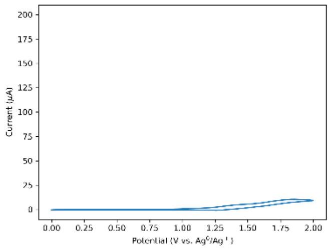	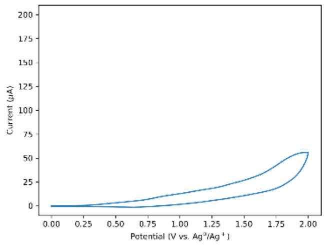	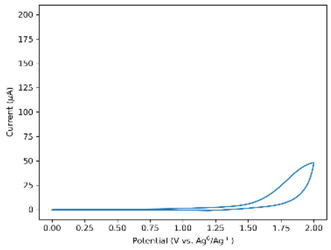
**S11b**	***C6***	**S38**
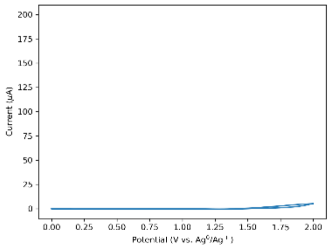	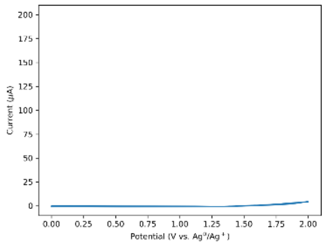	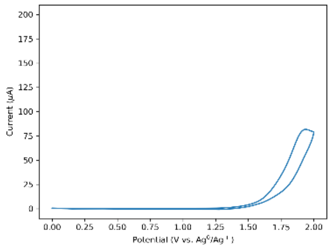

S15

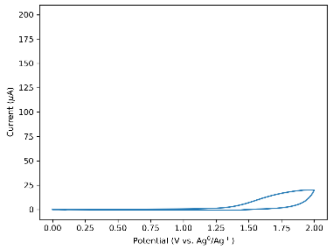

C7

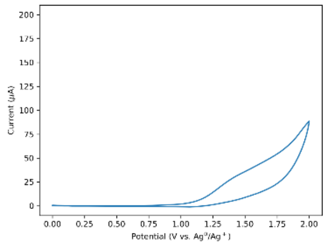

S38

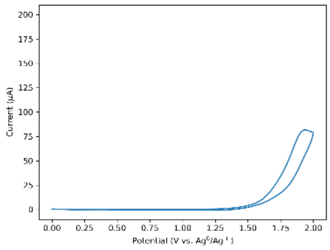

S15

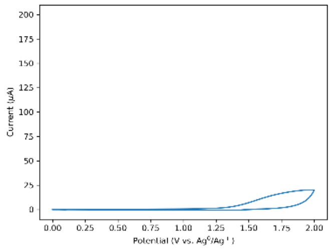

C8

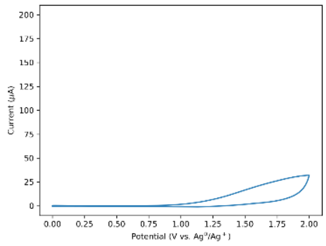

S88

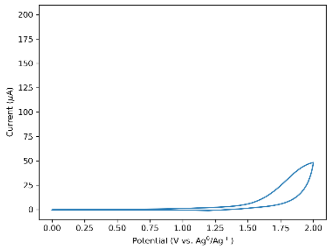

S17

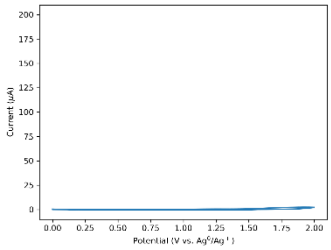

C9

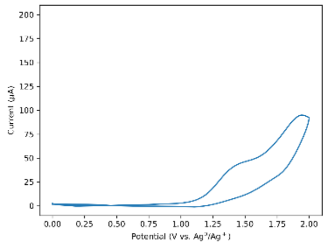

S38

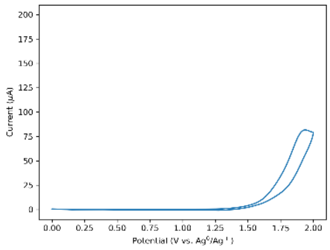

S19

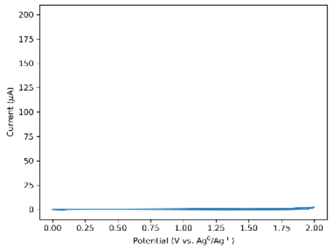

C10

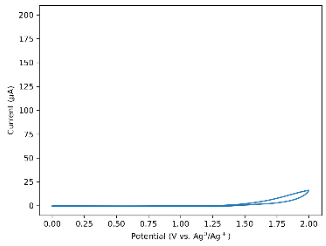

S88

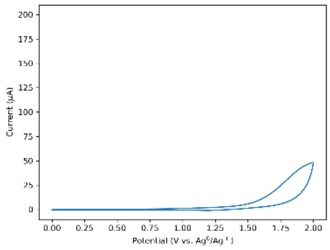

S22

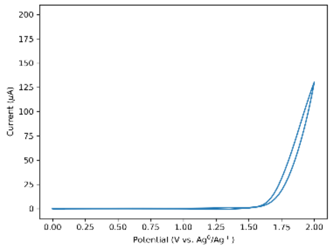

C11

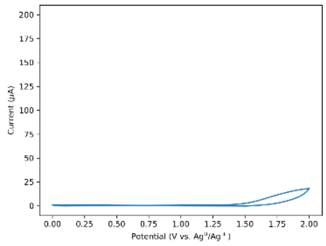

S95

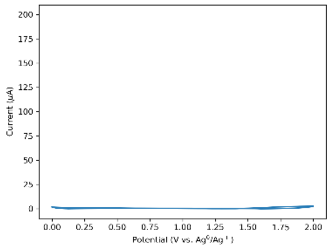

S32

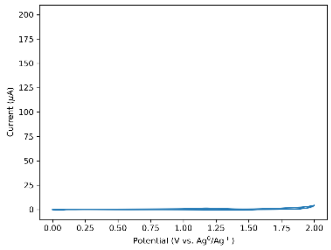

C12

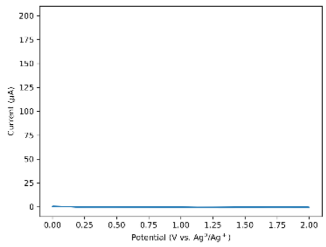

S95

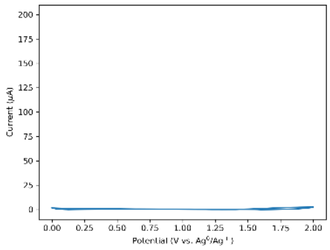

S38

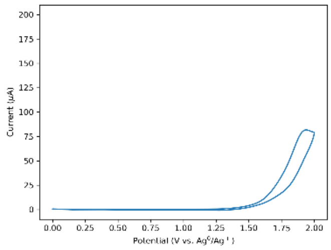

C13

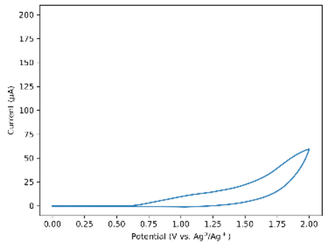

S88

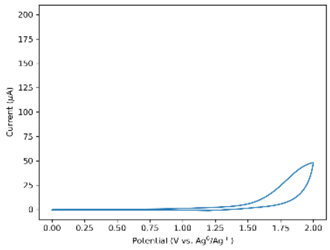

S48

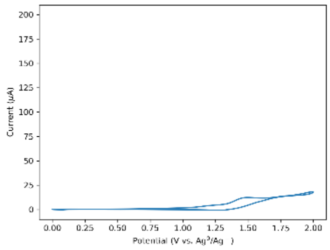

C14

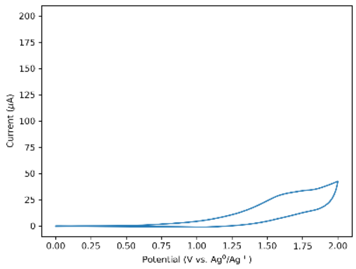

S88

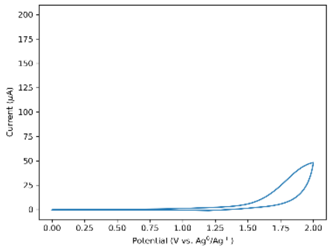

S75

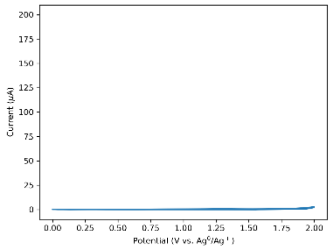

C15

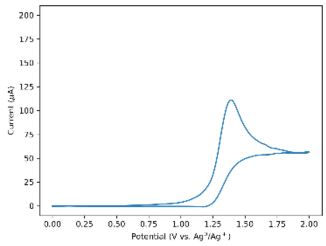

S88

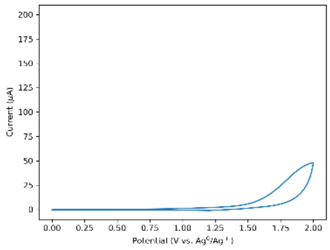

Table S35: Comparison of voltammograms of the cooperative pairs and their corresponding individual compounds between 0.00 V and −2.00 V (blue, y-scale between −10.0 μA and 210 μA).

**S4**	***C1***	**S38**
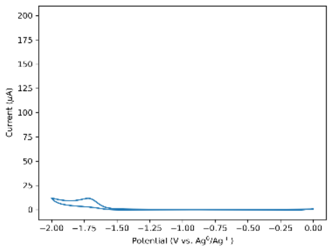	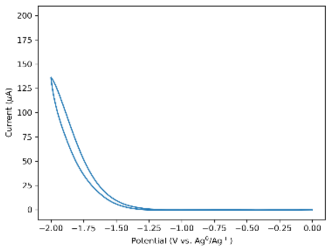	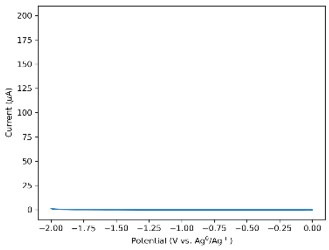
**S4**	***C2***	**S95**
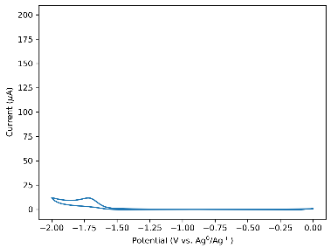	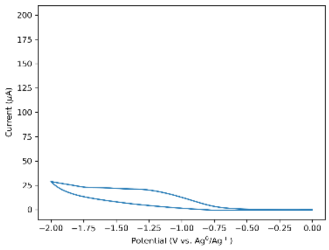	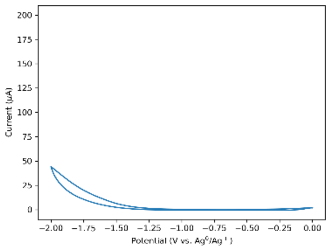
**S5**	***C3***	**S88**
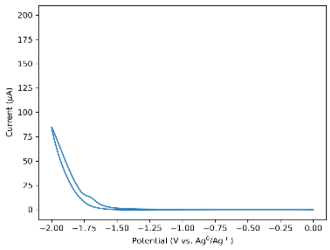	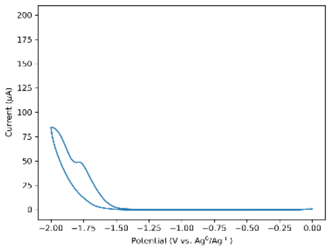	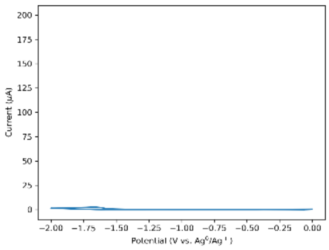
**S11b**	***C6***	**S38**
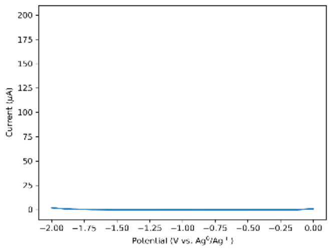	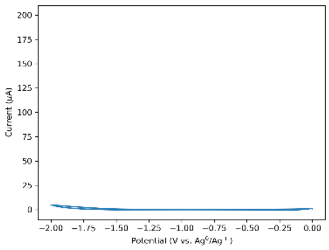	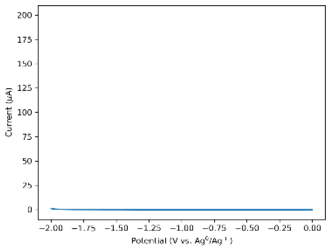

S15

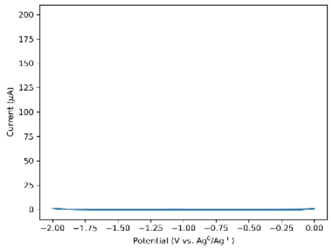

C7

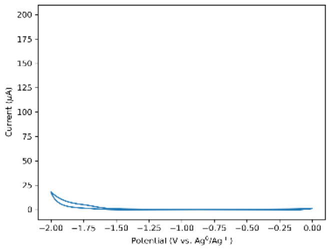

S38

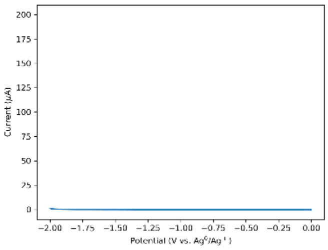

S15

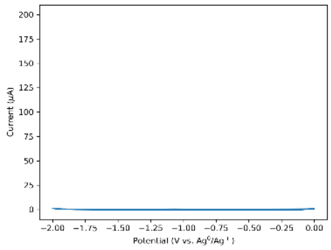

C8

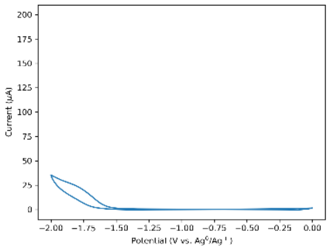

S88

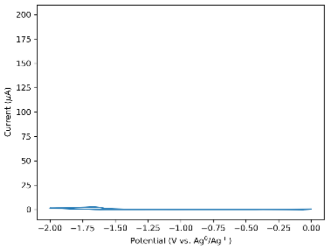

S17

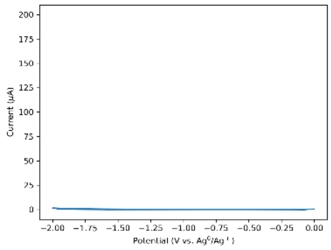

C9

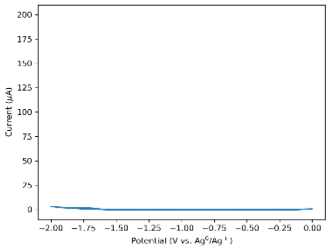

S38

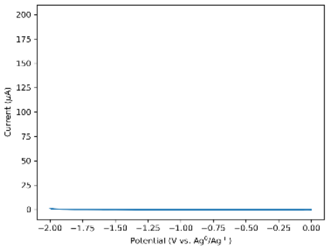

S19

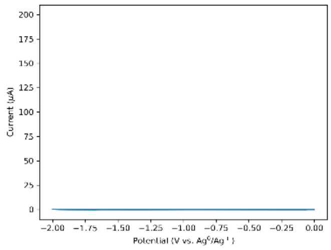

C10

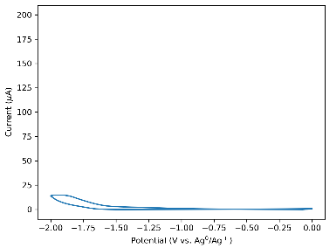

S88

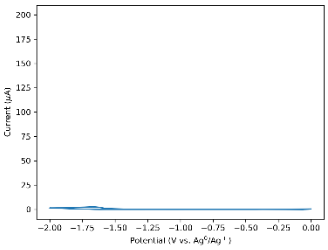

S22

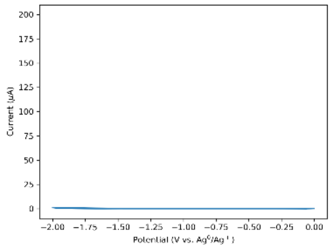

C11

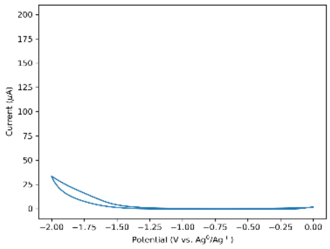

S95

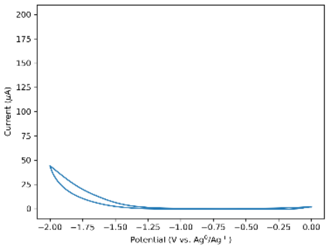

S32

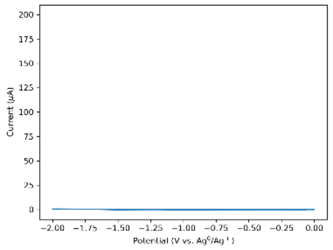

C12

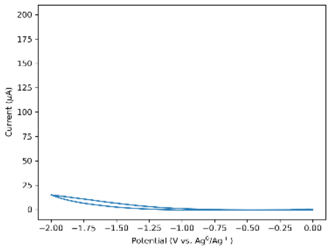

S95

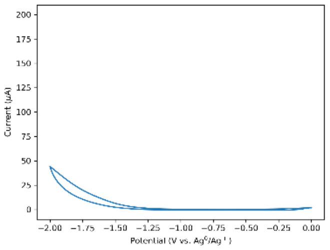

S38

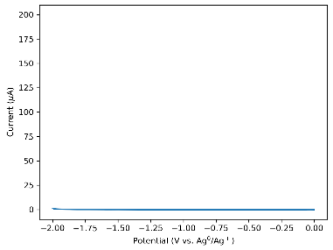

C13

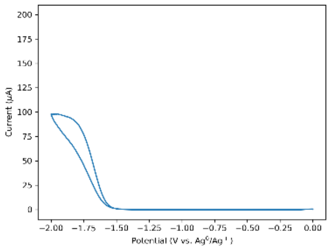

S88

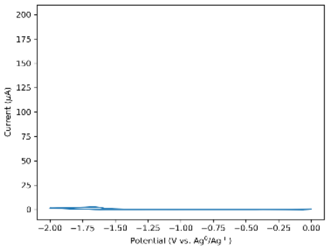

S48

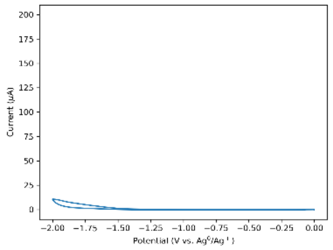

C14

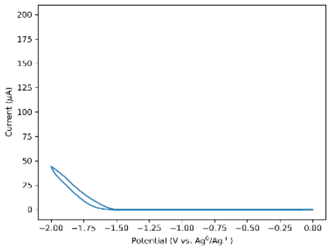

S88

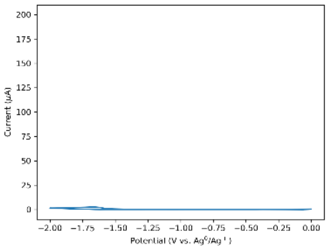

S75[a]

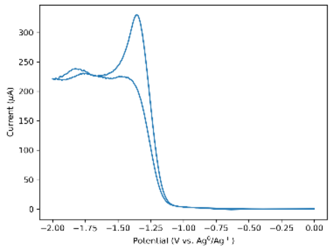

C15

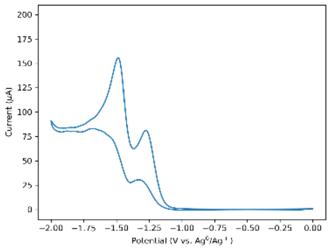

S88

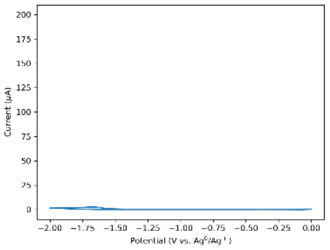

[a] Scale between −10 µA and 375 µA.

Table S36: Redox potentials $E^{1/2}$ of different non-quenching substrates (**S4**, **S5**, **S11b**, **S15**, **S17**, **S19**, **S22**, **S32**, **S38**, **S48**, **S75**, **S88** and **S95**). Potentials in the range of −2.50 V and 2.50 V are recalculated with reference to the SCE.

**Compound**	$E^{1/2}(S^0/S^+)$	$E^{1/2}(S^-/S^0)$
**S4**	-	−1.93 V[a]
**S5**	-	−2.01 V[a]
**S11b**	-	-
**S15**	1.53 V	-
**S17**	-	-
**S19**	-	-
**S22**	1.86 V	-
**S32**	-	-
**S38**	1.75 V[b]	-
**S48**	-	−2.34 V[a]
**S75**	-	−1.28 V
**S88**	1.74 V	-
**S95**	-	−2.27 V[a]

a)Potential determined from a voltammogram measured from 0.00 V to −2.50 V b)Potential determined from a voltammogram measured from 0.00 V to 2.50 V.

Table S37: Oxidation potentials $E^{1/2}$(S^0/S^+) of different cooperative pairs (**C1**, **C2**, **C3**, **C6**, **C7**, **C8**, **C9**, **C10**, **C11**, **C12**, **C13**, **C14** and **C15**.). Potentials in the range of 0.00 V and −2.50 V are recalculated with reference to the SCE. $\Delta E^{1/2}$ is calculated as difference of the pairs potential $E^{1/2}$(pair) and the $E^{1/2}$(single) most similar to the pair's potential. Potentials accessible by photocatalyst **PC1** (~1.21 V) are marked in green, potentials close to the oxidation potential of this catalyst are marked in yellow.

Pair	$E^{1/2}$(S^0/S^+)	Difference to single compounds ($\Delta E^{1/2}$)	Annotation
**C1**	1.56 V	−0.19 V	-
**C2**	-	-	-
**C3**	-	-	additional broad & weak peak at lower potential
**C6**	-	-	-
**C7**	1.19 V	-0.34	additional broad & weak peak at lower potential
**C8**	-	-	broad & weak peak below threshold
**C9**	1.40 V	-0.35	-
**C10**	-	-	-
**C11**	-	-	-
**C12**	-	-	-
**C13**	1.55	-0.31	additional broad & weak peak at lower potential
**C14**	1.49	-0.25	additional broad & weak peak at lower potential
**C15**	1.25	-0.49	-

[a)]Potential determined from a voltammogram measured from 0.00 V to −2.50 V [b)]Potential determined from a voltammogram measured from 0.00 V to 2.50 V.

Table S38: Reduction potentials $E^{1/2}(S^-/S^0)$ of different cooperative pairs (**C1**, **C2**, **C3**, **C6**, **C7**, **C8**, **C9**, **C10**, **C11**, **C12**, **C13**, **C14** and **C15**.). Potentials are given in the range of 0.00 V and 2.50 V and are recalculated with reference to the SCE. $\Delta E^{1/2}$ is calculated as difference of the pairs potential $E^{1/2}$(pair) and the $E^{1/2}$(single) most similar to the pair's potential. Potentials accessible by photocatalyst **PC1** (~0.89 V) are marked in green, potentials close to the reduction range of this catalyst are marked in yellow.

Pair	$E^{1/2}(S^-/S^0)$	Difference to single compounds ($\Delta E^{1/2}$)	Annotation
**C1**	−2.18 V[a]	-0.25	-
**C2**	−1.12 V	1.15 (new peak)	additional broad & weak peak at higher potential
**C3**	−1.49 V	0.52	additional peak at higher potential
**C6**	-	-	-
**C7**	-	-	weak peak below threshold
**C8**	−1.76 V	new peak	-
**C9**	-	-	-
**C10**	-	-	weak peak below threshold
**C11**	-	-	-
**C12**	-	-	weak peak below threshold
**C13**	−1.71 V	new peak	-
**C14**	−1.84 V	0.50	-
**C15**	−1.18 V (−1.28 V)	0.10	two distinguishable peaks

a)Potential determined from a voltammogram measured from 0.00 V to −2.50 V

Table S39: Redox potential of **PC1** to **PC3** against SCE.

	PC1	PC2	PC2
$E^{1/2}$ (PC^*/PC^+)	−0.89 V[18]	−1.73 V[28]	−0.26 V[30]
$E^{1/2}$ (PC^-/PC^*)	1.21 V[18]	0.31 V[28]	1.45 V[30]

Table S40: Absolute transferred charges Q^{ox} and Q^{red} for different non-quenching substrates (**S4**, **S5**, **S11b**, **S15**, **S17**, **S19**, **S22**, **S32**, **S38**, **S48**, **S75**, **S88** and **S95**).

Compound	Q^{ox}	Q^{red}
**S4**	18.3 µC	5.60 µAs
**S5**	119 µAs	274 µAs
**S11b**	33.2 µAs	14.4 µAs
**S15**	185 µAs	11.1 µAs
**S17**	27.6 µAs	14.2 µAs
**S19**	29.0 µAs	2.50 µAs
**S22**	460 µAs	10.6 µAs
**S32**	34.5 µAs	7.00 µAs
**S38**	417 µAs	87.0 µAs
**S48**	209 µAs	62.0 µAs
**S75**	24.5 µAs	3880 µAs
**S88**	291 µAs	21.4 µAs
**S95**	27.8 µAs	245 µAs

Table S41: Absolute transferred charges Q^{ox} for different cooperative pairs (**C1**, **C2**, **C3**, **C6**, **C7**, **C8**, **C9**, **C10**, **C11**, **C12**, **C13**, **C14** and **C15**.) and their corresponding different non-quenching substrates (**S4**, **S5**, **S11b**, **S15**, **S17**, **S19**, **S22**, **S32**, **S38**, **S48**, **S75**, **S88** and **S95**) as well as difference between the calculated additive charge and the charge for cooperative pairs (ΔQ^{ox}_{C-A}). Strong positive cooperative effects $Q^{ox}_{C}/Q^{ox}_{A} > 2.00$ (green) and strong negative cooperative effects $Q^{ox}_{C}/Q^{ox}_{A} <$ 0.50 (red) are marked accordingly.

Pair	Q^{ox}_{C}	S_a	Q^{ox}_{Sa}	S_b	Q^{ox}_{Sb}	Q^{ox}_{Add}	ΔQ^{ox}_{C-Add}	Q^{ox}_{C}/Q^{ox}_{Add}
**C1**	1660 µAs	**S4**	18.3 µAs	**S38**	417 µAs	435 µAs	1225 µAs	3.98
**C2**	65.3 µAs	**S4**	18.3 µAs	**S95**	27.8 µAs	46.1 µAs	19.2 µAs	1.41
**C3**	696 µAs	**S5**	119 µAs	**S88**	291 µAs	410 µAs	286 µAs	1.70
**C6**	28.8 µAs	**S11**	33.2 µAs	**S38**	417 µAs	450 µAs	−421 µAs	0.07
**C7**	740 µAs	**S15**	184 µAs	**S38**	417 µAs	601 µAs	139 µAs	1.23
**C8**	339 µAs	**S15**	184 µAs	**S88**	291 µAs	475 µAs	−136 µAs	0.71
**C9**	939 µAs	**S17**	27.6 µAs	**S38**	417 µAs	445 µAs	494 µAs	2.11
**C10**	91.4 µAs	**S19**	29.0 µAs	**S88**	291 µAs	446 µAs	−200 µAs	0.31
**C11**	128 µAs	**S22**	29.0 µAs	**S95**	27.8 µAs	56.8 µAs	71.2 µAs	0.23
**C12**	12.9 µAs	**S32**	34.5 µAs	**S95**	27.8 µAs	62.3 µAs	−49.4 µAs	0.21
**C13**	590 µAs	**S38**	416 µAs	**S88**	291 µAs	607 µAs	−17.0 µAs	0.88
**C14**	474 µAs	**S48**	209 µAs	**S88**	291 µAs	500 µAs	−26.0 µAs	0.95
**C15**	1130 µAs	**S75**	24.5 µAs	**S88**	291 µAs	336 µAs	794 µAs	3.34

Table S42: Absolute transferred charges Q^{red} for different cooperative pairs (**C1**, **C2**, **C3**, **C6**, **C7**, **C8**, **C9**, **C10**, **C11**, **C12**, **C13**, **C14** and **C15**.) and their corresponding different non-quenching substrates (**S4**, **S5**, **S11b**, **S15**, **S17**, **S19**, **S22**, **S32**, **S38**, **S48**, **S75**, **S88** and **S95**) as well as difference between the calculated additive charge and the charge for cooperative pairs (ΔQ^{red}_{C-A}). Strong positive cooperative effects Q^{red}_{C}/Q^{red}_{A} > 2.00 (green) and strong negative cooperative effects Q^{red}_{C}/Q^{red}_{A} < 0.50 (red) are marked accordingly.

Pair	Q^{red}_{Coop}	S_a	Q^{red}_{Sa}	S_b	Q^{red}_{Sb}	Q^{red}_{Add}	$\Delta Q^{red}_{C\text{-}Add}$	$Q^{red}_{C}/Q^{red}_{Add}$
**C1**	635 µAs	**S4**	5.60 µAs	**S38**	87.0 µAs	92.6 µAs	542 µAs	6.85
**C2**	482 µAs	**S4**	5.60 µAs	**S95**	245 µAs	251 µAs	231 µAs	1.92
**C3**	419 µAs	**S5**	274 µAs	**S88**	21.4 µAs	295 µAs	124 µAs	1.42
**C6**	31.0 µAs	**S11**	14.4 µAs	**S38**	21.4 µAs	35.8 µAs	−4.76 µAs	0.87
**C7**	65.3 µAs	**S15**	11.1 µAs	**S38**	87.0 µAs	98.1 µAs	−32.8 µAs	0.67
**C8**	192.9 µAs	**S15**	11.1 µAs	**S88**	21.4 µAs	32.5 µAs	160 µAs	5.93
**C9**	24.2 µAs	**S17**	14.2 µAs	**S38**	87.0 µAs	101 µAs	−77.0 µAs	0.24
**C10**	118 µAs	**S19**	2.50 µAs	**S88**	21.4 µAs	23.9 µAs	93.8 µAs	4.93
**C11**	187 µAs	**S22**	10.6 µAs	**S95**	245 µAs	256 µAs	−68.6 µAs	0.73
**C12**	148 µAs	**S32**	7.00 µAs	**S95**	245 µAs	252 µAs	−103 µAs	0.59
**C13**	613 µAs	**S38**	87.0 µAs	**S88**	21.4 µAs	108 µAs	504 µAs	5.65
**C14**	196 µAs	**S48**	62.0 µAs	**S88**	21.4 µAs	83.4 µAs	113 µAs	2.35
**C15**	1520 µAs	**S75**	3880 µAs	**S88**	21.4 µAs	3901 µAs	−2381 µAs	0.39

4.5.4 Interpretation

C1

The slight change of the half peak oxidation potential of this pair to $E^{1/2}$ = 1.56 V is a clear indication of a redox activation of this pair and a possible oxidative quenching. This is supported by the further decrease of the reduction potential from −1.93 V (**S4**) to −2.18 V. These potentials are clear electrochemical indications of an interaction between the two substrates **S4** and **S38**, with the HOMO of **S38** being raised (i.e., **S38** becoming more electron-rich). Since the observable oxidation peak is broad and intense meaning that the transferred electric charge increases significantly, the causative species might be responsible for the quenching of **PC1** even though its potential limit is not reached.

C2

The emergence of a new broad peak at a half peak potential of −1.12 V suggests a distinct new electron-deficient and thus reducible species. The width of the peak suggests that at lower voltages further electron transfer processes take place, superimposing the signal of the most easily reducible species. Thus, it can be assumed that the reduction potential of the quenching species (e.g. the protonated pyrazine **S4**) is considerably higher and thus within the range of the catalyst **PC1**.

C3

The combination of **S5** and **S88** results in a new reducible species with a half peak potential of −1.49 V. However, this species cannot be reduced by the employed photocatalyst and thus is not the cause of the observed quenching. In the range between 0.50 V and approximately 1.50 V, the broad signal of an easily oxidizable species becomes apparent. However, since this signal is below the set threshold of 25 µA and overlaps with the signals of **S5** and **S88**, a determination of the oxidation potential is not possible. The change of the electric charge integral in the positive mode between 0.00 and 2.00 V and in the negative mode between 0.00 and −2.00 V, as well as the clear visibility of the signals assignable to **S5** and **S88** suggests a new strongly bound and easily oxidizable species.

C6

The disappearance of any peaks within both measured voltammograms of the cooperative pair leads to the conclusion that the cooperative species is either involved in energy transfer processes or is involved in electron transfer processes but forms in small amounts only. Thereby

could fall below the limit of detection of the applied cyclic voltammetry experiment. However, the later cannot explain the disappearance of oxidation peak of substrate **S38** which indicates the formation of a new species. In addition, measurements between −2.7 V and 2.7 V were carried out whereby no exceptional current was measures. Measurements were repeated multiple times.

C7

The strong change of the oxidation potential of this pair to $E^{1/2}$ = 1.19 V is a clear indication of a redox activation of this pair and a oxidative quenching. This potential is a clear indication of an electrochemical interaction between the two substrates **S15** and **S38**, with the HOMO of **S38** or **S15** being raised (i.e., **S38** or **S15** becoming more electro-rich). Even though an additional reduction peak becomes visible above $E^{1/2}$ = −1.50 V it is not in the range of the photocatalysts redox potential. This could be caused by weak π−π interactions or H-bonding, which raises the electron density of one substrate (presumably **S38**) and lowers the electron density of the other. (3.1 to 3.5)

C8

As denoted by the changed potential and increased electric charge the combination of **S15** and **S88** results in a new reducible species with a half peak potential of −1.76 V. However, this species cannot be reduced by the employed photocatalyst and thus is presumably not causing reductive quenching. In addition, a broad and weak peak is visible below the applied threshold but in a range of 1.10 V and 1.60 V. This peak is overlayed by the peaks corresponding to **S15** and **S88** but in the range to oxidatively quench **PC1**.

C9

The appearance of a new oxidation peak and increased electric charge Q^{ox} indicate the formation of a new species. Even though the half peak potential is determined to be 1.40 V, two peaks clearly overlay in the recorded voltammogram. The half peak potential of the peak at lower potential can be estimated to be ~1.25 V meaning that a strong change occurs. This potential is an electrochemical interaction between the two substrates **S17** and **S38**, with the HOMO of probably **S38** being raised (i.e., **S38** becoming more electron-rich) into the range of **PC1**. Since the oxidation peak of **S38** is still visible, it is expected that only parts of this compound are involved in the formation of the oxidizable species. However, this species has clearly differentiable redox properties as evidenced by a $\Delta E^{1/2}_{C9\text{-}S38}$ = 0.50 V.

C10

Only slight changes in the cyclic voltammograms of **C10** can be detected. In general, a minor signal appears in the voltammograms at a potential <−1.50 V while the transferred electric charge between 0.00 V and 2.00 V gets slightly reduced. Based on these results no species exhibits redox-based cooperativity or forms in small amounts only and thereby falls below the detection limit of the applied cyclic voltammetry studies.

C11

Only slight changes in the cyclic voltammograms of **C11** can be detected in all measured potentials and the transferred electric charge. Based on these results no species exhibits redox-based cooperativity or forms in trace amounts only and thereby falls below the detection limit of the applied cyclic voltammetry studies.

C12

Although only slight changes in the cyclic voltammograms of **C12** can be detected in all measured potentials a significant change in measured electric charges can be recognized. In addition, a minor reduction peak appears in the area between −1.10 V and −1.60 V which overlaps with the reduction peak of **S32** ($E^{1/2}$ > 2.00 V). This signal might correspond to the protonated pyrimidine **S32**, which is thereby subjected to a lowering of the LUMO. Due to overlaps and the low intensity the half-peak potential cannot be determined but the reduction potential of this species could be in the range of the potentials of **PC1**.

C13

In the case of voltammograms for the mixture of **S38** and **S88**, numerous changes can be perceived compared to the voltammograms of both individual substrates. On the one hand, a new reduction peak with $E^{1/2} = -1.71$ V can be observed. However, this reduction cannot be achieved by **PC1**. Furthermore, the half peak potential of **S38** or **S88** shifts by $\Delta E^{1/2}_{C13\text{-}S38} = 0.35$ V or $\Delta E^{1/2}_{C13\text{-}S88} = -0.34$ V to 1.40 V, whereby this is probably caused by the superimposition of three peaks. The half peak potential of the lower peak can be estimated to be < 1.10 V, and therefore, oxidation could be achieved by the photocatalyst. The broadness of the peak, as well as the visibility of the oxidation peak corresponding to the starting material of **S38** and **S88** suggest a reversible equilibrium process being cause of the cooperativity.

C14

In the case of voltammograms of the mixture of **S48** and **S88**, numerous changes can be perceived compared to the voltammograms of both individual substrates. On the one hand, a new reduction peak with $E^{1/2} = -1.84$ V can be observed. However, this reduction cannot be achieved by **PC1**. Furthermore, a new broad peak occurs with $E^{1/2} = 1.74$ the half peak potential of **S88** shifts slightly by 0.25 V to 1.49 V, whereby superimposing with different peaks making the calculation of a distinct potential highly inaccurate, still giving hint for redox-based quenching.

C15

While the voltammogram between 0.00 V and −2.00 V shows a clear reduction of the peak belonging to **S75**, a second, clearly distinguishable signal indicates the emergence of a new species. This is also reinforced by the appearance of a peak $E^{1/2} = 1.25$ V whereby the causing species seems to be also responsible for the corresponding reductive quenching of **PC1**. The almost complete reduction of the signal from **S88** also suggests a clear, presumably covalent cooperative interaction (6 to 8).

4.6 Investigation on Redox Reversibility and Possible Reactivity

Besides quantifying the strength of all discovered cooperative effects and confirming that these cooperative interactions in most cases have an influence on the pair´s redox properties, the voltametric data can be used to determine characteristics of possible secondary reactions and thus reactivity. In cases where the oxidized or reduced species undergoes an irreversible subsequent reaction with a high rate constant k, a non-Nernstian response, more precisely no or a weakened back reduction or back oxidation, can be detected (Scheme S1).[33]

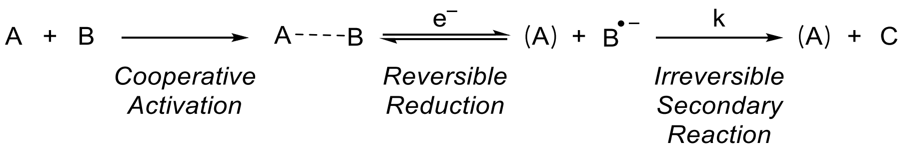

Scheme S1: Reversible electron transfer followed by an irreversible chemical reaction after cooperative redox activation exemplified for oxidative quenching.

For pairs (**C1**, **C2**, **C7**, **C9**, **C13**, **C14**, **C15**), for which a clear cooperative redox activation was verified by cyclic voltammetry by the appearance of a signal in the redox window of **PC1**, the corresponding peaks were analysed for back reduction or back oxidation. In none of the cases

this back reaction could be detected at a low scan speed of 0.1 V/s (4.5.2 and 4.5.3). Even in cases where only indications of a cooperative redox effect were found (**C3**, **C8, C12**), no signal of a back reduction was found.

Overall, this missing back reductions and oxidations confirm that most cooperative pairs found by this screening do not only exhibit higher activities but also higher reactivity. Although this missing reversibility, i.e., indication of an irreversible follow-up reaction, does not correspond to the finding of synthetically useful transformations, it proves that cooperative hits also show a rise in reactivity. As demonstrated by cyclic voltammetry, this rise in reactivity corresponds to more accessible redox activities and thereby enables milder activation. The combination of irreversible secondary reactions and accessible activation allows for the design of novel reactions having unique reaction pathways compared to reactions with higher activation barriers. Since this reactivity could reach from direct decomposition over structural rearrangements to dual reactions further studies have to be carried out, investigating this reactivity (5.1, 5.2).

To further investigate the rate constant of secondary reactions cyclic voltammetry could be conducted with higher scan rates whereby slow irreversible reactions will exhibit a back oxidation or reduction peak (4.5.2). However, since multiple species have been found to be present in most mixtures a clear investigation on redox reversibility and rate constants of the following reaction poses significant challenges going beyond the scope of this work.

4.7 Conclusion of Studies Investigating the Change in Activity and Reactivity

During the cyclic voltammetry studies, the nature of the cooperative interaction could be elucidated with a considerable degree of certainty for seven pairs. Large shifts in redox potentials, by up to 0.5 V (**C3**, **C14**, **C15**), as well as the occurrence of completely new peaks (**C8**) in the measured redox range were detected giving a hint of strong changes in the electronic structure of the cooperative pairs compared to their individual substrates. It has to be mentioned that shifts in potential do not necessarily correspond to a weak alteration in one substrate but could also correspond to a fully new species. In addition, evidence of a positive redox-based cooperative effect was found among three additional pairs, although the intensities of the newly formed peaks were below the limit for a reliable qualification and quantification of the interaction. This allows the conclusion that convolutional luminescence-based screening, as described in this work, is well suited to detect strong and thus utilizable cooperative effects.

This method is able to selectively detect significant changes in the HOMO or LUMO energies (either through the formation of a new product or weak interactions, 3.1 to 3.6) of tested substrate combinations via a change in the redox properties. These more accessible redox potentials could now be used in different types of reactions, where a general change of the redox properties (i.e. of the HOMO or LUMO energies) allows the use in photocatalytic but especially in electrochemical or metal catalytic transformations. Although CV is particularly well suited to qualify and quantify the type and strengths of cooperative interactions, it has also been found that overlapping signals and the weak intensity of the relevant peaks (i.e. peaks attributed to cooperatively interacting substrates) altering CV unapplicable for a convolutional detection cooperativity. In three cases, the mode and strength of the cooperative interaction could not be elucidated by CV. In these cases, either a non-redox-based cooperative interaction (e.g. energy transfer processes) is assumed to be responsible for the quenching of the catalyst or the detection limit of the CV was not reached, since the corresponding active species only forms in low concentrations. These active species could be found by using the quenching of **PC1**s fluorescence, which of course can only be used to detect interactions that either fall into the catalysts redox range or have a lower triplet energy than the catalyst. However, by changing or modifying the photocatalyst both the triplet energy[34] and the redox potentials[35] can be tuned broadly, meaning that the type of cooperative interaction which a screening searches for can also be adjusted. Based on this assumption quenching studies were carried out with **PC2** as strongly reducible and **PC3** as strongly oxidizable catalyst. Even though the results of these studies (4.2) mainly overlap with results of CV experiments (Table S43) major differences can be spotted (**C13**, **C14**). One major issue of this method is that some individual compounds are already quenching the photocatalysts on a moderate to high level, making distinctions between individual compounds and pair challenging bringing the need for calculating the change in quenching (4.1). This finally leads to inconclusive results which get aggravated by catalyst deactivation like in the case of **S95** and **PC2**. Overall, multi catalyst studies are suited to get indications about the mode of quenching but approximations of needed thresholds, the quenching by side products and the need to find robust catalysts with fitting potentials make the application of such studies troublesome.

In ten of thirteen cases the mode of quenching could be determined by cyclic voltammetry. Thereby, only pairs having methane sulfonic acid **S95** involved (**C2**, **C12**) have been found to get reduced by **PC1** (oxidative quenching). In both cases the substrates **S4**, **S32** get protonated by the acid whereby the LUMO gets lowered and becomes more accessible. For pairs having

S38 as substrate reductive quenching is proposed due to the shift of peaks towards lower oxidation potentials. Thereby often only a shift of signals or the occurrence of comparably weak signals can be detected supporting conclusion that **S38** partially and in equilibrium forms non-covalent bonds with its cooperative partner whereby its electron density gets increased. For all pairs an irreversible follow up reaction can be proposed due to the missing of a back reduction/oxidation peak. This leads to the conclusion that found cooperative interactions give access to highly reactive species which undergo fast chemical reactions after oxidation or reduction (5.1).

Table S43: Proposed quenching mechanisms based on the conducted multi-catalyst luminescence quenching experiments and cyclic voltammetry studies (4.2 to 4.5).

Pair	Multi catalyst quenching	Cyclic Voltammetry	Annotation
**C1**	reductive quenching	reductive quenching	Measured potential is not in the range of the photocatalyst **PC1**[a]
**C2**	oxidative quenching	oxidative quenching	Intensity of new peak low; **PC3** becomes inactivated by **S95**
**C3**		reductive quenching	Intensity of new peak low; quenching studies inconclusive
**C6**[c]			Quenching and CV studies inconclusive
**C7**		reductive quenching	Intensity of new peak low; Quenching studies inconclusive
**C8**		reductive quenching	Quenching studies inconclusive
**C9**	reductive quenching	reductive quenching	Determination of the oxidation potential inaccurate due to overlapping signals
**C10**			Quenching and CV studies inconclusive
**C11**	oxidative quenching		
**C12**	oxidative quenching	oxidative quenching	**PC3** becomes inactivated by **S95**
**C13**	oxidative quenching	reductive quenching	Quenching studies inconclusive since also the quenching of **PC3** increases with the pair
**C14**		reductive quenching	Side products could cause the quenching of **PC2**
**C15**	reductive quenching	reductive quenching	

a) Since a broad peak is visible a oxidation by the catalyst seems to be likely. b) Additional quenching and NMR studies with different batches of **S10** suggest that an impurity is responsible for the cooperative effect with **S88** and **S95**. Due to low reproducibility and the dependence on a specific batch of **S10**, this hit pair was not further investigated. c) Cooperative effect is proposed to occurs between **S38** and imidazole **S11b** (hydrolysis product of **S11**) based on the conducted NMR studies.CV studies were conducted using imidazole **S11b** as cooperative partner.

5. Investigations on Chemical Reactivity

5.1 Decomposition Screen

5.1.1 Background

After proving that found cooperative interactions strongly influence the electronic nature of the involved compound, thereby altering their activity, e.g. against a photocatalyst or redox potential this change in activity does not necessarily mean a significant change in reactivity. Even though cyclic voltammetry studies have demonstrated that irreversible secondary reactions occur in case of **C1**, **C2**, **C7**, **C9**, **C13**, **C14**, **C15** this is only an indication of possible decomposive reactivity and does also not correspond to applicability in the development of chemical reactions. In case of four pairs (**C3**, **C11**, **C14**, **C15**), the cooperative interaction itself was proven to be caused by a chemical reaction, i.e., a nucleophile-electrophile reaction, while it has to be investigated if these newly formed compounds can be utilized in chemical transformations.

To investigate the potential for the utilization of cooperative hits in chemical reaction, a second reaction screen can be employed whereby different potential coupling partners or radical acceptors can be tested against the cooperative pairs (5.2).[25] A simpler strategy to investigate potential reactivity of cooperative pairs could aim for elucidating the reaction pathway after the compound's interaction with the photocatalyst i.e. after utilizing the cooperative effect thereby transferring energy to or an electron to/from the substrate(complex). In these cases, either a transformation (e.g. decomposition) could take place, or the compound could return to its origin state (e.g. by a electron transfer to the oxidized/reduced photocatalyst). Since, the first process is a chemical reaction itself a decomposition would proof reactivity for the given pair. In addition, decomposing behaviour could be sign for the release of a highly energetic or chemically utilizable species e.g. a reactive radical.

5.1.2 Practical Design

Based on these assumptions, a screen was designed testing cooperative pairs in combination with either stoichiometric amounts of **PC1** or substoichiometric amounts of the same in the presence of $[NH_4]_2[S_2O_8]$ **10a** as oxidant or *N*,*N*-diisopropylethylamine **10b** as reductant. If electron or energy transfer of the excited photocatalyst to (one substrate of) the cooperative pair follows a decomposition, at least one substrate will not be detectable anymore within the reaction mixture after irradiation. In case reactions including **10a** and **10b** as oxidant or

reductant respectively only substoichiometric amounts of **PC1** are needed as catalyst turnover is insured, even though only half of a reductive or an oxidative catalytic cycle can be employed (Figure S20).

To make sure discovered cooperative interaction is cause of decomposition and to exclude reactions based on weak or unlikely interaction of the photocatalyst with one of the individual substrates control reactions were carried out by testing individual pairs in combination with either stoichiometric amounts of **PC1** or substoichiometric amounts of the same in the presence of $[NH_4]_2[S_2O_8]$ **10a** as oxidant or *N*,*N*-diisopropylethylamine **10b** as reductant (Figure S21).

5.1.3 Experimental Details

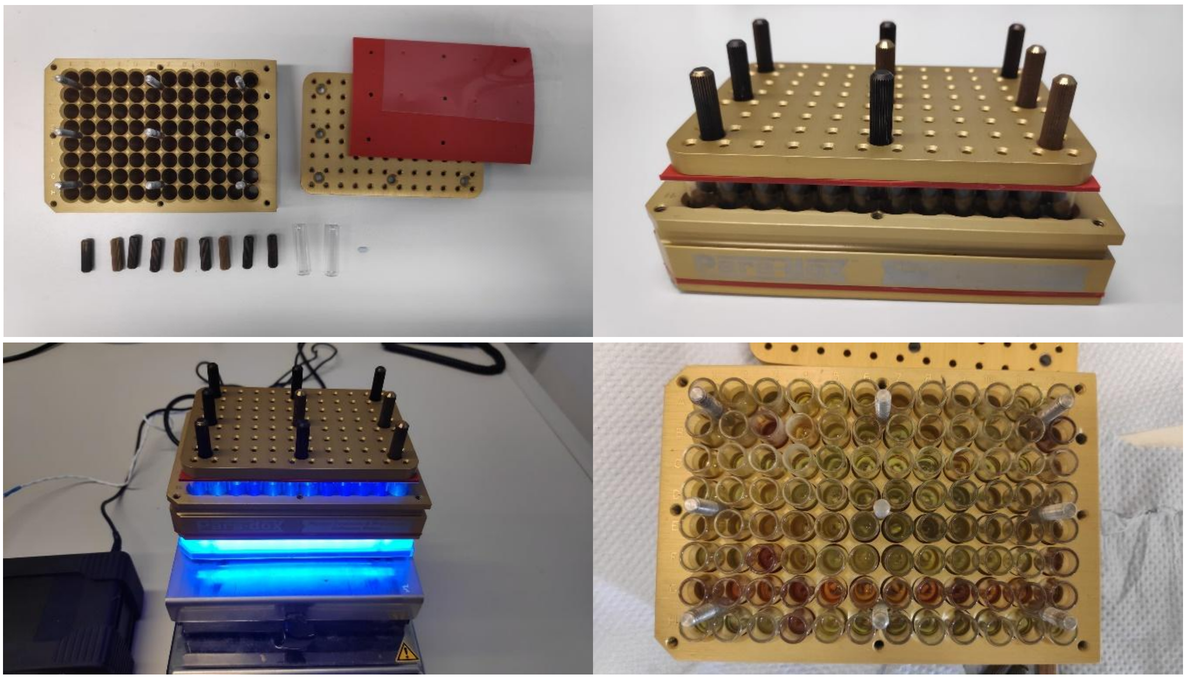

Figure S19: Photographs of the experimental setup used for the decomposition and radical acceptor screening. An 8 x 12 (96) well plate with 800 µL reaction vessels was used, with individual irradiation on every reaction vessel.

The decomposition screen was performed using the first five (three in case of the controlls) rows of a "Para-dox" 96-well plate, equipped with an appropriate irradiation system (Figure S19). Each reaction vessel was equipped with a magnetic stir bar and the whole plate was prepared under argon atmosphere. The whole setup was placed on a laboratory shaker and irradiated for 16 h. After the indicated reaction time, each reaction mixture was diluted with EtOAc to a volume of approximately 400 µL. An aliquot (100 µL) of each reaction mixture

was taken, filtered through a short plug of silica, eluted with EtOAc (1.0 mL) and analysed by means of GC-MS. All GC-MS data is available in the git repository at the path GC-MS-Plate-Analyser/input/GCMS5.asr or GC-MS-Plate-Analyser/input/GCMS6.asr.[26] All data with sample IDs of “PPP-PB-50-XY” correspond to the 96-well plate carrying reaction with the cooperative pairs in row A to E as depictured in Figure S20 while all data with sample IDs of “PPP-PB-53-XY” ” correspond to the 96-well plate carrying control reactions in row A to C as depictured in Figure S21.

Decomposition Experiments

Stock solutions of **PC1** (0.500 M for stoichiometric experiments; 0.0125 M in MeCN for substoichiometric experiments), the pairs (both substrates 0.250 M in MeCN), and the oxidation/reduction reagents (**10a/10b**) (0.250 M in MeCN) were prepared under an argon atmosphere. To each reaction vessel the catalyst stock solution (50 µL, 0.0250 mmol, 1.0 eq for stoichiometric experiments; 50 µL, 0.625 µmol, 2.5 mol% for substoichiometric experiments), the pairs stock solution (100 µL, 0.0250 mmol, 1.0 eq), and the oxidation or reduction reagents stock solution (100 µL, 0.0250 mmol, 1.0 eq) were added. In case of the experiments involving stoichiometric amounts of **PC1** MeCN (100 µL) was added into the corresponding reaction vessel. Cooperative pairs **C4** and **C5** were not included in the conducted screen due to the above-mentioned issues, while **C7** was left out randomly and for practical reasons to have the experiments fit on one 96-well plate.

Control Experiments

Stock solutions of **PC1** (0.500 M for stoichiometric experiments; 0.0125 M in MeCN for substoichiometric experiments), the substrate (0.250 M in MeCN), and the oxidation/reduction reagents (**10a/10b**) (0.250 M in MeCN) were prepared under an argon atmosphere. To each reaction vessel the catalyst stock solution (50 µL, 0.0250 mmol, 1.0 eq for stoichiometric experiments; 50 µL, 0.625 µmol, 2.5 mol% for substoichiometric experiments), the substrates stock solution (100 µL, 0.0250 mmol, 1.0 eq), and the oxidation or reduction reagents stock solution (100 µL, 0.025 mmol, 1.0 eq) were added. In case of the experiments involving stoichiometric amounts of **PC1** MeCN (100 µL) was added into the corresponding reaction vessel.

5.1.4 Results

To define whether decomposition occurred under corresponding reaction conditions, semiquantitative GC-EI-MS (Gas Chromatography Electron Ionization Mass Spectrometry) analysis was carried out, checking for a strong reduction in the substrate's peak area. In case of cooperative pairs where the cooperative interaction was based on the formation or break of at least one covalent bond (**C3**, **C11**, **C14**, **C15**) the decomposition of these newly formed products (cooperative product) was measured. A reference of ever substrate was measured having approximately the same concentration. Overall, only peaks of the substrates or cooperative products were analysed while peaks corresponding to possible products were ignored.

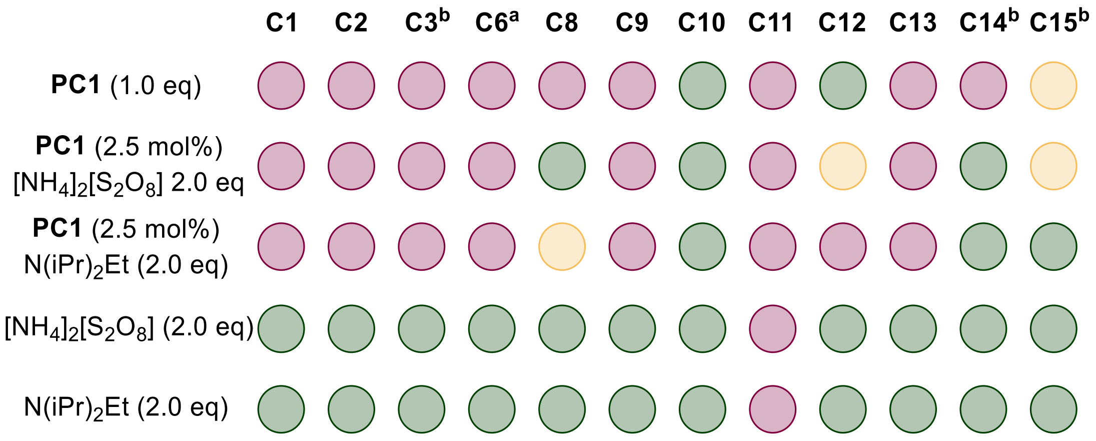

Red indicates full decomposition (<10% compound remaining) of at least one compound, Yellow indicates partial decomposition (<50% compound remaining) of at least one compound. Green indicates no detectable decomposition for both compounds. [a)]Imidazole **11b** used instead of **11** as it has been identified of being involved in the cooperative interaction, [b)] Decomposition of the cooperative product was tracked.

Figure S20: Results of the decomposition studies and oxidant/reductant controls as given by semiquantitative GC-MS analysis.

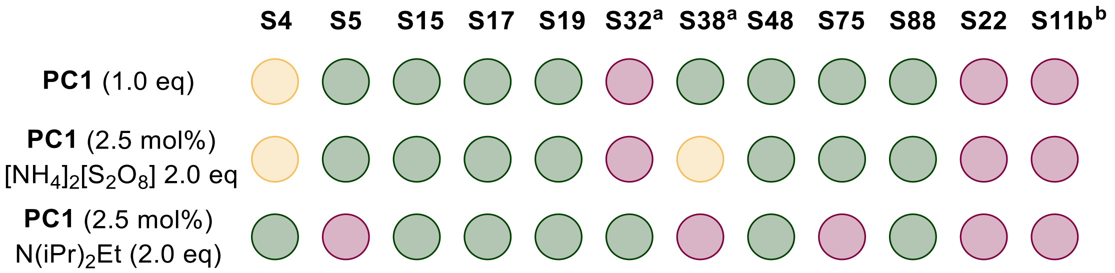

Red indicates full decomposition (<10% compound remaining) of at least one compound, Yellow indicates partial decomposition (<50% compound remaining) of at least one compound. Green indicates no detectable decomposition for both compounds. [a)] Reactions carried out on a different screening plate.[b)] Imidazole **11b** used instead of **11** as it has been identified of being involved in the cooperative interaction.

Figure S21: Results of the single compound controls for the decomposition study given by semiquantitative GC-MS analysis.

Experiments were classified into 3 classes: *full decomposition* (red) given if at least one substate or cooperative products was fully consumed (<10% peak area corresponding to the reference); *partial decomposition* (yellow) given if at least one substrate or cooperative products was consumed significantly (<50% peak area corresponding to the reference); *no decomposition* (green), given if no substrate or cooperative product was consumed significantly (>50% peak area corresponding to the reference). **S95** was left out since it cannot be detected with the utilized GC methods due to its low boiling point.

Table S44: Summary of all stoichiometric experiments within the decomposition screen.

	**Substrate A**	**Substrate B**	**Decomposition of A**	**Decomposition of B**	**Decomposition of C**	**Hit**
**C1**	**S4**	**S38**	🟡	🟢	🔴	yes
**C2**	**S4**	**S95**	🟡	not testet	🔴	yes
**C3**	**S5**	**S88**	🟢	🟢	🔴	yes
**C6**	**S11b**	**S38**	🔴	🟢	🔴	no
**C8**	**S15**	**S88**	🟢	🟢	🔴	yes
**C9**	**S17**	**S38**	🟢	🟢	🔴	yes
**C10**	**S19**	**S88**	🟢	🟢	🟢	no
**C11**	**S22**	**S95**	🔴	not testet	🔴	no
**C12**	**S32**	**S95**	🔴	not testet	🟢	no
**C13**	**S38**	**S88**	🟢	🟢	🔴	yes
**C14**	**S48**	**S88**	🟢	🟢	🔴	yes
**C15**	**S75**	**S88**	🟢	🟢	🟡	yes

5.1.5 Conclusion

In a first step, in categories classified, results of the decomposition studies, carried out with stoichiometric amounts of **PC1** and all cooperative pairs were compared to the results generated with the corresponding individual substrates. Thereby, a result is considered to be a hit if both individual compounds show no signs of or only partial decomposition while the cooperative pair exhibited full decomposition, as defined before. While following this comparison eight out

of twelve cooperative pairs (**C1**, **C2**, **C3**, **C8**, **C9**, **C13**, **C14**) were found as hits and thereby clear chemical reactivity. In two out of twelve cases (**C6**, **C11**) the decomposition could not clearly be assigned to the cooperative interaction since also one individual substrate decomposed. For two out of twelve reactions (**C10**, **C12**) involving cooperative pairs no decomposition was detected. Since reactions including the reducing **10b** and the oxidizing agent **10a** show high overlap with the stoichiometric experiments no deeper or statistical analysis of these results was carried out. For the sake of completeness results of those experiments are also given (Figure S20 and S21). Overall, ~67% of the analysed pairs exhibit cooperativity-based decomposition and thereby first signs of utilizable chemical reactivity. In a next step a second reaction screen could be employed whereby the pairs are tested against a variety of potential radical acceptors.

5.2 Reaction Screening

5.2.1 Background

Even though decomposition is a proof of reactivity itself also compounds which are stable under conditions could be able to undergo a chemical reaction with a suited reaction partner. Thereby reactivity of the cooperatively interacting species would be created after interaction with the catalyst enabling subsequential reactions. To test the found cooperative pairs for their reactivity and direct applicability in chemical reactions they can be tested against potential coupling partners or radical acceptors in a second screening approach. The goal of this approach is not the finding of high-yielding or out-of-the-box applicable reactions, but to initially find reactivity at all. If one is interested in developing reactions out of these initial reactivity, in prior generated insights into the nature of cooperative interaction (3.) as well as understanding about the change in electronic behaviour (4.) could be used to rationally design and optimize synthetically utilizable reactions. (6. to 7.). If reactions can be found that cannot be undergone by the individual substrates, but the cooperative pair the assumption can be confirmed that a cooperative screening approach is suitable for the discovery of new reactivities.

5.2.2 Practical Design

To carry out a second reaction screen twelve potential coupling partners or radical acceptors were chosen to cover a variety of different functionalities and potential reactivities. Beside from compounds carrying activated and unactivated double and triple bonds (**A1**, **A2**, **A5**, **A6**, **A7**), different electron rich and poor heterocycles (**A3**, **A4**, **A12**), acceptors with diverse functional

groups (**A8**, **A9**, **A10**, **A11**) have been chosen (Figure S22). Again, it has to be emphasized that the goal of this screen was to test cooperative pairs for reactivity in general, wherefore highly complex acceptors and functional groups have been excluded. 13 cooperative pairs (**C1**, **C2**, **C3**, **C6**, **C7**, **C8**, **C9**, **C10**, **C11**, **C12**, **C13**, **C14**, **C15**) have been tested against this acceptors on two 96-well plates (Figure S23). To differentiate between reactivity solely based on the coupling partners or on individual substrates reaction controls were carried out subjecting only the acceptors and the individual substrates (**S4**, **S5**, **S11b**, **S15**, **S17**, **S19**, **S22**, **S32**, **S38**, **S48**, **S75**, **S88**, **S95**) to the reaction conditions (Figure S23). Using this strategy and comparing the cooperative pair's reaction outcome to the outcome of its corresponding controls all background reactivity (i.e. potential false positives) can be subtracted. Including controls, 324 reactions were carried out (156 x cooperative pair and acceptor, 156 x substrate and acceptor, 12 x acceptor only) (5.2.3).

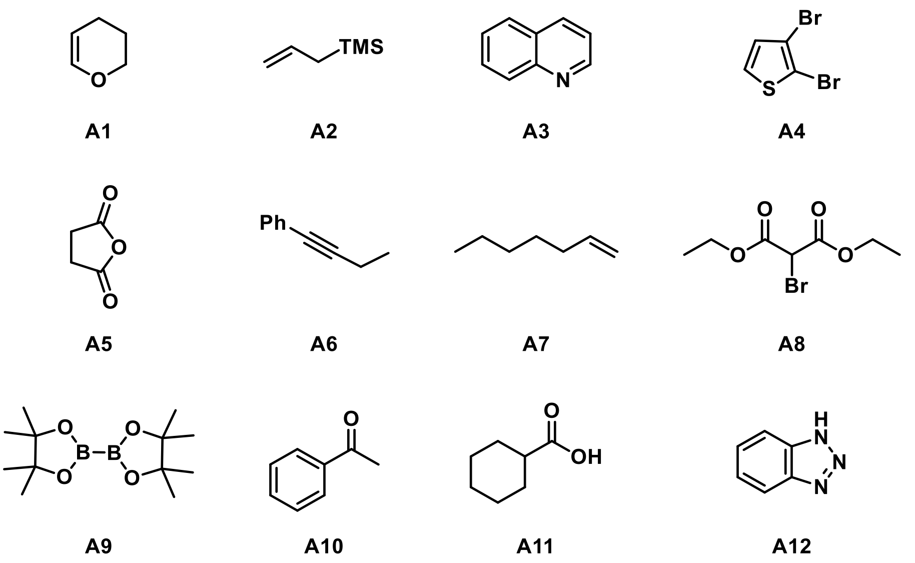

Figure S22: Potential coupling partners and radical acceptors used for the reaction-based screening.

Since acceptors were chosen comparably unpolar and light GC-MS has been selected as analysis method of choice. To make analysis of one plate possible over night while also taking into account compounds eluting at high boiling, a method was developed having a high heating rate and gas flow while holding higher temperatures for an over commonly long time. With a method time of 10 minutes a full 96-well plate can be analysed on two machines in approximately 8 to 9 hours.

Since it is expected that a large number of peaks will be visible in the chromatograms, which need to be traced back to different relevant or non-relevant reactions, an efficient way of peak

assessment needs to be found. For this purpose, chromatographic and spectral data was pre-processed using the MassHunter analysis software and exported to the .asr file format.[26] This contains all peak, spectral and chromatographic data as well as all meta information about the sample the chosen method. For further automatic analysis of the chromatographic and spectroscopic data, a custom evaluation software was developed using Python 3.9. This enables the systematic and reproducible analysis of the experimental results by comparing retention times and spectra of the main reactions with their corresponding control reactions and automatically classifying and labelling peaks accordingly (5.3.4). Peaks classified as unique, meaning that they neither occurred in one of the corresponding control reactions nor are related to the cooperative pair solely (i.e. are related to the unique reactivity of the cooperative pair with the acceptor) were taken into account for further analysis. Since it is expected that multiple small, therefore probably less import, peaks will be labelled as related to the pairs reactivity a threshold was applied for base peak intensity (BPI).

To make structural proposals from these filtered peaks, the EI mass spectra were analysed by means of manual and database-based assignment, while ESI-HRMS was applied to confirm sum formulas of proposed structures. With this approach, structures cannot be elucidated without uncertainty since no conclusion can be drawn about different (regio)isomers, but these proposals can be utilized to get insights into possible reaction mechanisms and further potential applications of the cooperative pairs. In addition, statistical information about the general reactivity of identified cooperative pairs can be gained by this approach.

5.2.3 Experimental Details

The reaction screen was performed using a "Para-dox" 96-well plate, equipped with an appropriate irradiation system (Figure S19). Each reaction vessel was equipped with a magnetic stir bar and all plates were prepared under argon atmosphere. The whole setup was placed on a laboratory shaker and was irradiated for 16 h. After the indicated reaction time, each reaction mixture was diluted with EtOAc to a volume of approximately 400 µL. An aliquot (100 µL) of each reaction mixture was taken, filtered through a short plug of silica, eluted with EtOAc (1.0 mL) and analysed by means of GC-MS. Overall, four screening plates were prepared carrying 324 reactions (Figure S23). On every plate, acceptor solutions were pipetted into column 1 to 12, corresponding to **A1** to **A12**. Cooperative Pair Plate 1 (Figure S23, upper left) contained the cooperative pairs **C1**, **C2**, **C3**, **C6**, **C7** and **C8** in row B to G while row A and H

were left without any compounds. Cooperative Pair Plate 2 (Figure S23, upper right) contained the cooperative pairs **C9** to **C15** and **C8** in row B to H while row A was left without any compounds. Control Plate 1 (Figure S23, upper left) contained the individual substrates **S4**, **S5**, **S11**, **S15**, **S17** and **S19** in row B to G while row A and H were left without any compounds. Control Plate 2 (Figure S23, upper right) contained the individual substrates **S22**, **S32**, **S38**, **S48**, **S75**, **S88**, **S95** in row B to H while row A contained only the radical acceptor solutions with **PC1**. Every vessel used either for reactions or controls contained photocatalyst **PC1**.

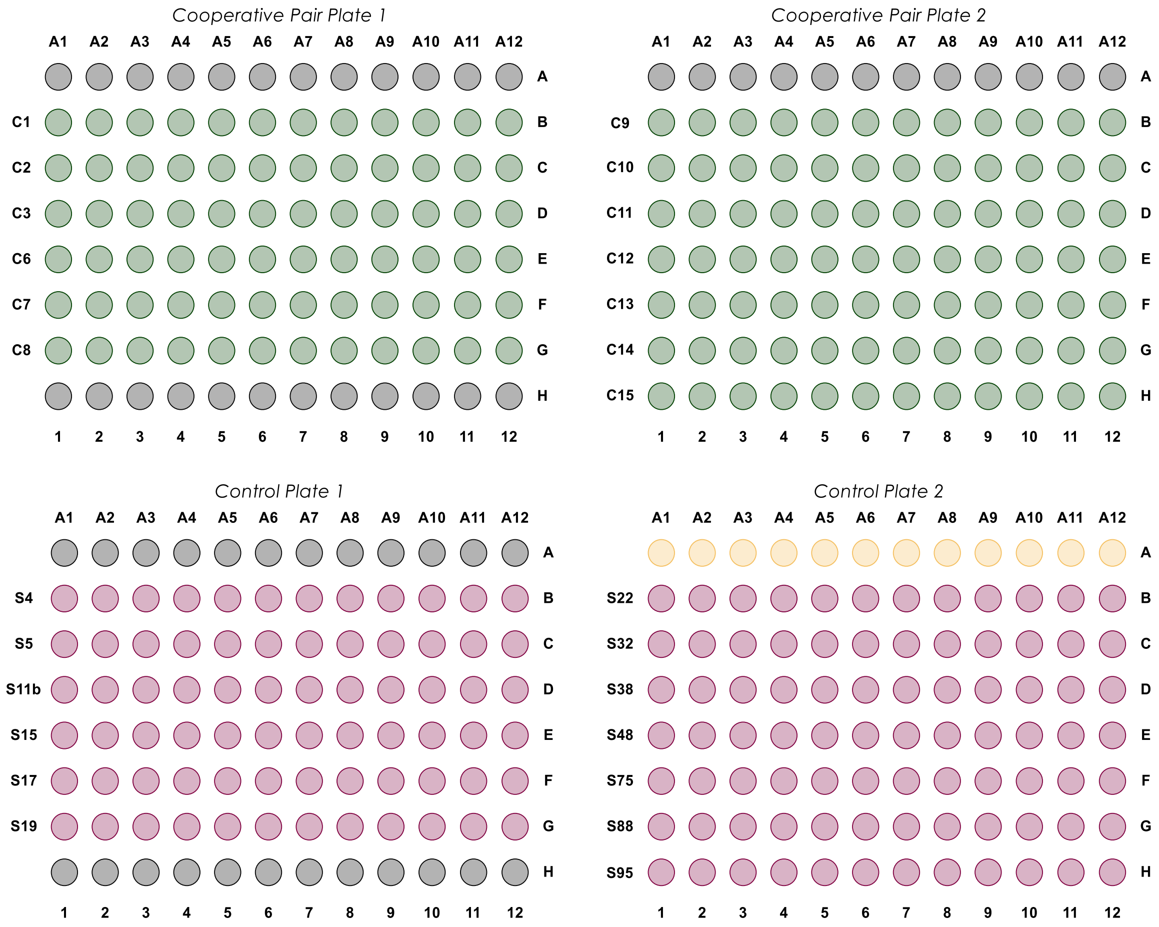

Figure S23: Plate design for the described reaction screen. Numbers and letters below and left of the plate relate to the plate positions. Colours relate to the reaction type: full reaction (green), substrate and acceptor control (red), acceptor only (yellow).

GC-MS spectra were recorded on an *Agilent* Technologies Intuvo 9000 GC-System with an *Agilent* 5977B GC/MSD inert Mass Selective Detector (EI) and a HP-5MS column (0.25 mm x 30 m, film: 0.25 µm). The used method starts with the injection temperature T_0 (50 °C); after holding this temperature for 0.5 min, the column is heated by 100 °C/min to temperature T_1 (300 °) and this temperature is held for an additional time t (7 min). A solvent delay of 1.9 min

was used before starting the ionization and detection. Helium was used as carrier gas while a flow rate of 16.2 mL/min was applied. The bus temperature was set on 300 °C while the guard chip was heated mapping the oven temperature starting with a temperature T_0^{Guard}(70 °C). All GC-MS data is made available in the projects git repository at the path GC-MS-Plate-Analyser/input/GCMS5.asr or GC-MS-Plate-Analyser/input/GCMS6.asr.[26] All data with sample IDs of "PPP-PB-54-P1/2-XY" correspond to Cooperative Pair Plate 1 or 2 reaction while all data with sample IDs of "PPP-PB-60-P1/2-XY" " Control Plate 1 or 2. If no plate name is given it can be assumed that samples belong to plate 1.

High resolution mass spectra (HRMS) were obtained by the MS service of the Organisch-Chemisches Institut, Universität Münster, using electrospray ionisation (ESI) on a *Thermo Fischer Scientific* Orbitrap LTQ XL. All ESI-HRMS data is made available in the git repository at the path Additional_Data/ESI-HRMS/. and is given in the .raw format.

Cooperative Pair Plates

Stock solutions of **PC1** (0.0125 M in MeCN), the pairs (both substrates 0.250 M in MeCN), and the acceptors (**A1** to **A12**) (0.500 M in MeCN) were prepared under an argon atmosphere. To each reaction vessel the catalyst stock solution (50 µL, 0.625 µmol, 2.5 mol%), the pairs stock solution (100 µL, 0.0250 mmol, 1.0 eq), and the additives stock solution (100 µL, 0.0500 mmol, 2.0 eq) were added following the pipetting scheme as described above.

Control Plates

Stock solutions of **PC1** (0.0125 M in MeCN), the individual substrates (0.250 M in MeCN), and the acceptors (**A1** to **A12**) (0.500 M in MeCN) were prepared under an argon atmosphere. To each reaction vessel the catalyst stock solution (50 µL, 0.625 µmol, 2.5 mol%), the individual substrates stock solution (100 µL, 0.0250 mmol, 1.0 eq), and the additives stock solution (100 µL, 0.0500 mmol, 2.0 eq) were added following the pipetting scheme as described above.

5.2.4 Semiautomated Analysis

To avoid manual comparison of the analytical data from the full reactions with data from corresponding control reactions, a semiautomated workflow was envisioned for unified and reproducible pre-processing, reading and labelling of spectra and chromatograms, leading to a final product proposal (Figure 24). The goal of this approach was to distinguish not cooperativity-related reactivity, e.g., decomposition of an acceptor or reactions of individual

substrates, from cooperativity-related reactivity. In a first step, GC-MS chromatograms and spectra were used since gas chromatography allows for simple separation of different compounds and thereby for identification of potential products. Other methods like UHPLC-ESI-MS were considered but due to polarity and boiling point of expected product motives an ionization/analysation by this method seems to be unlikely. In addition, fragmentation data of the EI-MS spectrum was used to identify products by the means of databases and human spectrum evaluation.

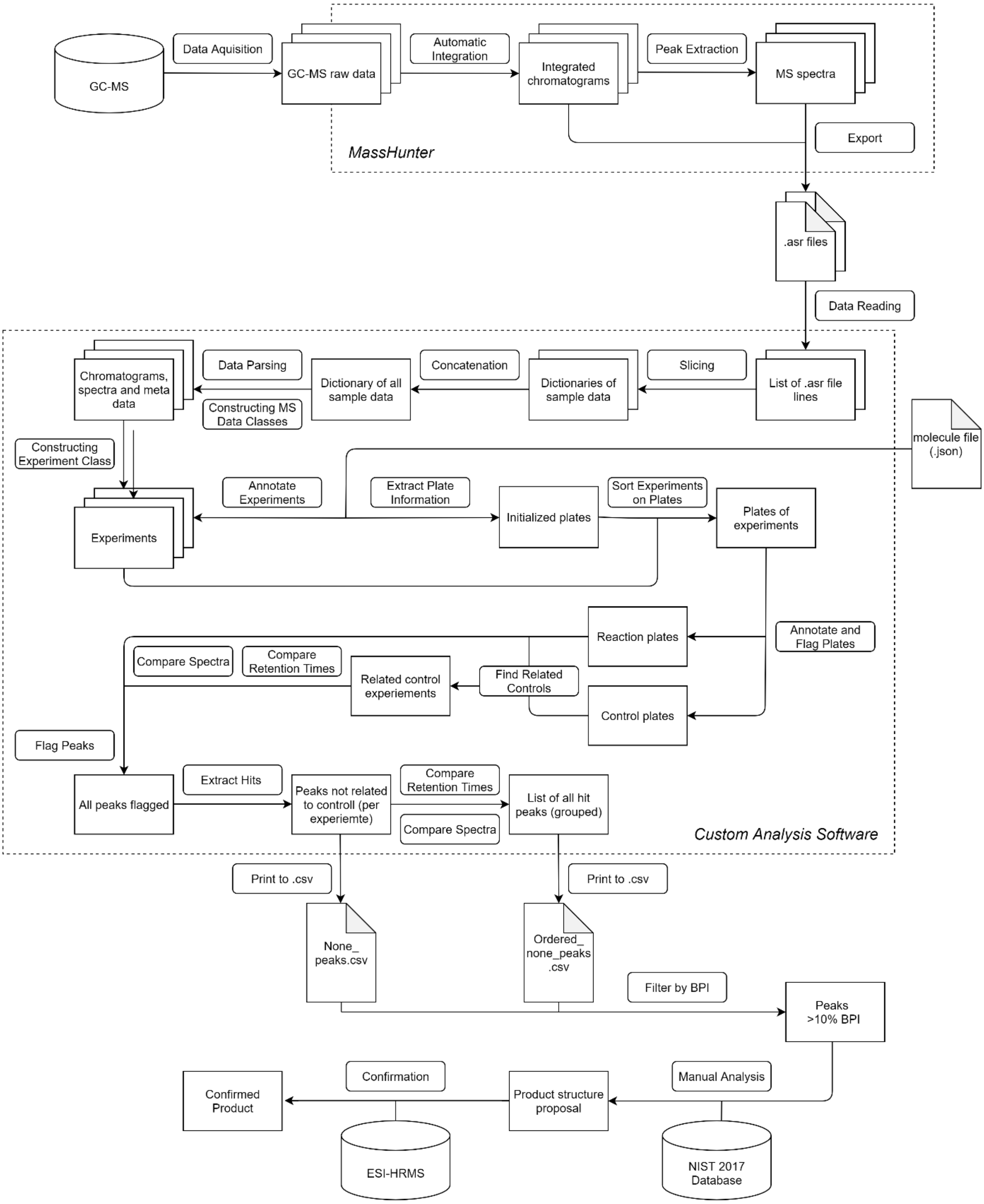

Figure S24: Workflow for the semiautomatic evaluation of the analytical data generated within the conducted reaction screenings.

As initial data reviews revealed the presence of highly complex mixtures, even in the case of some control reactions, a unified pre-processing approach was designed. Thereby, raw GC-MS file folders for all experiments were loaded into the Masshunter Software.[26] The procedure was conducted for data from both GC-MS instruments individualy to conserve this information for potential later analysis. All chromatograms were integrated automically using the "Integrate (MS)" (integration method: agile 2, absolute peak area >50.000 counts and 2.0 % BPI) and highlighted. For those peaks spectra were extracted using the "Extract (MS)" method whereby an automatic background subtraction was carried out. For this subtraction a background spectrum was defined at r_t = 9.0 min as it was found that no peak exists at this time in any spectrum. To speed up later spectrum comparison processes the maximal number of *m/z* peaks was set to 50 while peaks were filtered out if they only show <2% counts compared to the base peak. Finally, spectra were exported as .asr files while all data of one machine was combined in one export. For simplicity reasons no spectral devolution (i.e. the separation of overlapping peaks by following different mass traces) was carried out which we would strongly recommend in case on a full automatization of similar workflows.

In a subsequent, step this data should be read in, converted into a systematic format and labelled according to the plate design (Figure 23). Afterwards, chromatographic data of experiments and corresponding controls shall be connected and compared. Within this comparison peaks shall be labelled depending on their origin (e.g. peaks to simple acceptor decomposition should be flagged as "ACCEPTOR"). Finally, information shall be printed out in a human-readable format i.e. a .csv file.

Since .asr files cannot be parsed by commonly available tools, a parser was built reading all .asr files line-wise from the input folder before splicing them into a list of dictionaries containing different data segments as given by the file. Thereby, square brackets ("[" and "]") and curly braces ("{" and "}") were used to identify the beginning and ending of an informational segment. Each dictionary of information, corresponding to one measured sample, was then used to initialize these samples as instances of the "Experiment" class. This object contains metadata like name ("Chromatogram_name") and position on the plate but is mainly composed of corresponding chromatographic and spectrometric data as collected in an "MS_Data" object. Again, this object contains additional data from the .asr file like the GC-MS method name or time of data acquisition while its core elements are a list of all spectrometric and peak as well

as a instance of the chromatographic data ("Spectrum" class, "Peak" class, "Chromatogram" class). The Experiment object also includes molecular information read in from the mol.json file which needs to be defined in the input folder. This file contains a dictionary of all plates relevant for the screening and information about the included molecules. The plates sub dictionary contains one key for every row and column whereby the value defines a list of molecules which have been added into this column.

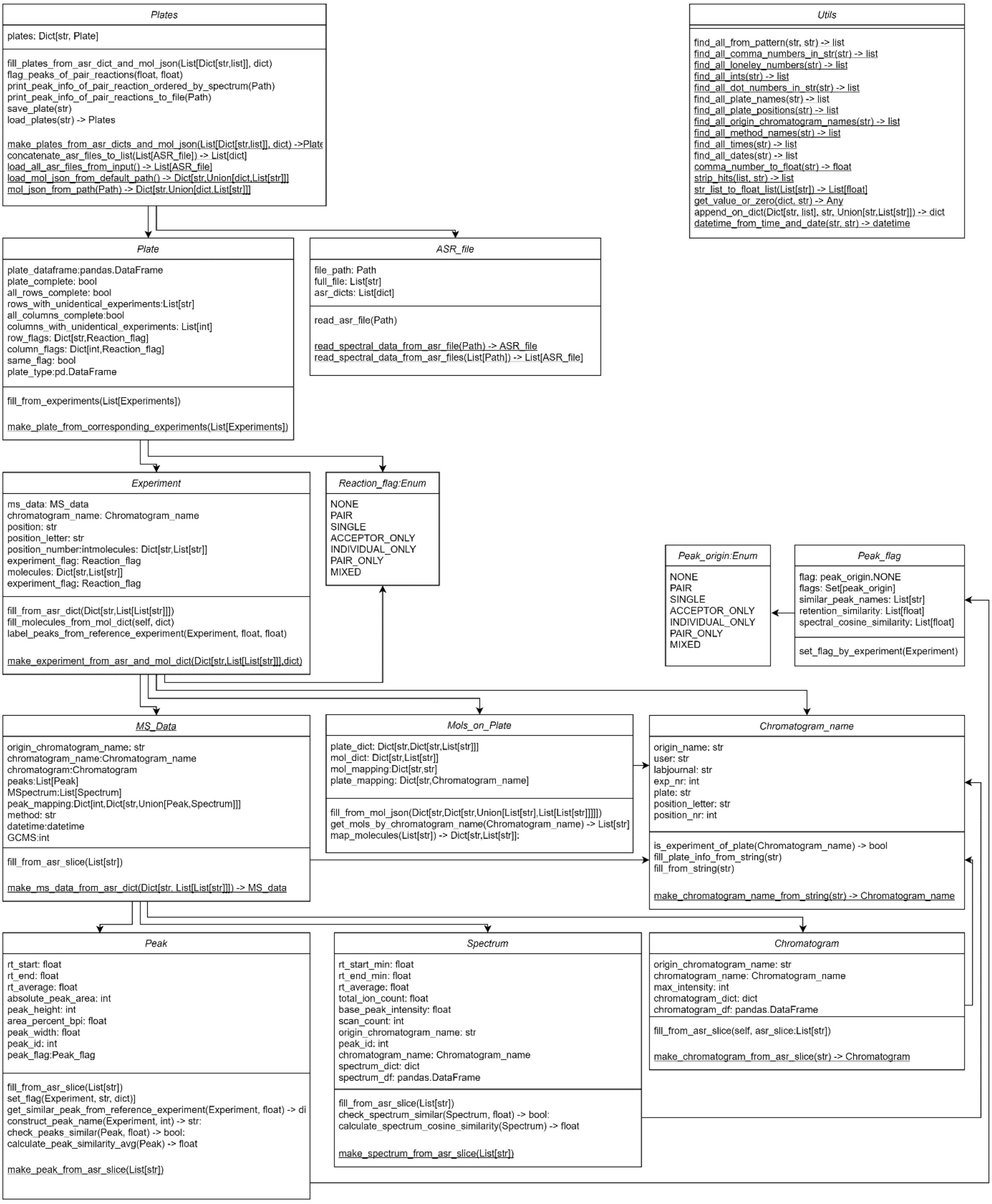

Figure S25: Simplified class diagram of the developed data analysis software.

Molecules were given as their corresponding canonical SMILES string. Keys of columns or rows which are left out in the screening hold a list only containing a single “NONE” string. Based on this dictionary, which gets read in once for the initialization of all plates, experiments first get their related molecular information and second get labelled with a reaction flag. This flag defines if a reaction is a control (“SINGLE”, “ACCEPTOR_ONLY”), a reaction of a cooperative pair with an acceptor (“PAIR”) or has not been classified yet (“NONE”). For details on the construction of Experiment objects please consider the simplified class diagram (Figure S25) and the code which is typed and named accordingly. Having all experiments initialized by the pseudo static function “make_experiment_from_asr_and_mol_dict()” they are getting collected in a list. Based on this list and the plates as defined in the mol.json file experiments having the corresponding experiment name and number can be filtered out,“Plate” objects can be initialized based on these by the function “make_plate_from_corresponding_experiments()”. While this initiation experiments will be ordered in a pandas.Dataframe object with the dimensions of the 96-well plate (8x12). Beside of other flag information which gets checked in the construction process and for simpler handling of experiment tags a second Dataframe will be constructed collecting all reaction_flags. Finally, these plates will be collected in a dictionary which forms the basis of a single plates object (Figure S24, S25).

Based on these Plates of flagged experiments, peaks, and spectra can know be compared and flagged accordingly. This is handled by the method plate.flag_peaks_of_pair_reactions(). Therefore, experiments with the reaction_flag “PAIR” are drawn from their corresponding plates while control reactions having the same acceptor molecule (acceptor controls, “ACCEPTOR_ONLY”) or substrate molecules (substrate controls, “SINGLE”) are searched from the other plates. In addition, not-control reactions having the same pair of substrates (cooperative pair) are picked and used for comparison. This is done to ensure that peaks related to the background reactivity of a cooperative pair (e.g. decomposition) can be traced and marked accordingly. In other words, if a peak with the same spectrum occurs independent of the acceptor molecule it is, most likely not caused by a reaction of the pair with the acceptor. All control experiments are collected and will be used for later comparison.

All peaks/spectra of the corresponding experiment which are to be assessed are scanned and compared with all peaks/spectra of the control experiments. A dual filter mechanism is used to compare the average retention time of the peak on the one hand and the mass spectra on the other hand. Since small changes and errors in column length and flow paths have a larger impact

on high retention times, a normalized metric (*ΔRT_{norm})* was used for the retention time comparison:

$$\Delta RT_{norm} = 1 - \frac{\left|RT^{a}_{average} - RT^{b}_{average}\right|}{\left(RT^{a}_{average} + RT^{b}_{average}\right)} * 2$$

For spectrum comparison a weighted *Cosine Similarity metric* was used which is commonly used in spectra library searches:

$$Cosine\ Similarity(Spec_a, Spec_b) = \frac{\sum_{i=1}^{mz_{max}} mz_i I_{ia}^{0.5} \cdot mz_i I_{ib}^{0.5}}{(\sum_{i=1}^{mz_a} mz_i I_{ia}^{0.5})^2 \cdot (\sum_{i=1}^{mz_b} mz_i I_{ib}^{0.5})^2}$$

Thereby $Spec_a$ and $Spec_b$ are the massspectra with *mz* as the rounded mass to charge ratio and intensity *I*. To determine if two peaks are similar a threshold of 0.975 is used for ΔRT_{norm} while a threshold of 0.900 was used for the *Cosine Similarity*. Thresholds were chosen comparably liberally to make sure that machine-specific differences between retention times and spectra are taken into account. Since computational costs for the calculation of the cosine similarity are, by multiple orders of magnitude higher than the determination of ΔRT_{norm} this metric is calculated first. In case the metric is above the threshold, the cosine similarity is also determined. In case that both thresholds are exceeded, the two peaks can be assigned to the same product or start material. In this case, the peak flag is set according to the reaction flag type of the comparison experiment. This class (Peak_flag) is designed to store similarity scores, distinct peak_ids of similar peaks and distinct flags, which can be used for possible future evaluations.

In total, all analysed reactions (excluding controls) feature 2150 peaks (6.6 peaks per reaction) whereas 421 peaks (~20%) from the actual reactions could not be assigned to substrates in corresponding controls. These peaks correspond to products which are likely to be caused of the unique reactivity of the cooperative pair with the additive. An issue in peak identification and flagging is that overlapping peaks could cause highly distorted spectra which than are not similar anymore to identical peaks in the control reactions. To write the obtained peak information into corresponding .csv files, two functions are available in the Plates class: "print_peak_info_of_pair_reactions_to_file()" and "print_peak_info_of_pair_reaction_ordered _by_spectrum()". The former constructs a list of all experiments, each with peaks ordered by unique reactivities ("NONE" tag). In addition, the peak ID and the retention time are given in this report to allow manual assignment. The latter compares all peaks with "NONE" tag according to the aforementioned threshold and groups highly similar peaks together. These are saved as a .csv file with corresponding experiment and peak ID, retention times, similarities

and base peak intensity. The appearance of identical products in reactions with difference cooperative pairs can be explained by the high similarity of some of them in terms of type of interaction and a potential quenching mode (e.g. **C1** and **C9**). From a total of 421 product-related peaks obtained from the 156 reactions performed, spectra of 299 unique peaks (supposably related to unique products) were identified. However, since the majority of these peaks have minimal intensities, i.e. only indicate the presence of a trace product, further filtering is necessary (Figure S26). This filtering was conducted founded on the relative base peak intensity (BPI, i.e. the intensity compared to the intensity of the largest peak in the spectrum) whereby a threshold of 10% was applied. It has to be mentioned that this does not correspond to a minimal yield of >10% since BPI is dependent of the ionization capability of the product and of the intensity of the largest peak. By this filtering 107 unique product peaks remain which need to be analysed. To avoid repetition of data parsing, object construction and peak labelling a save/load mechanism for Plates object was developed using python standard library pickle.

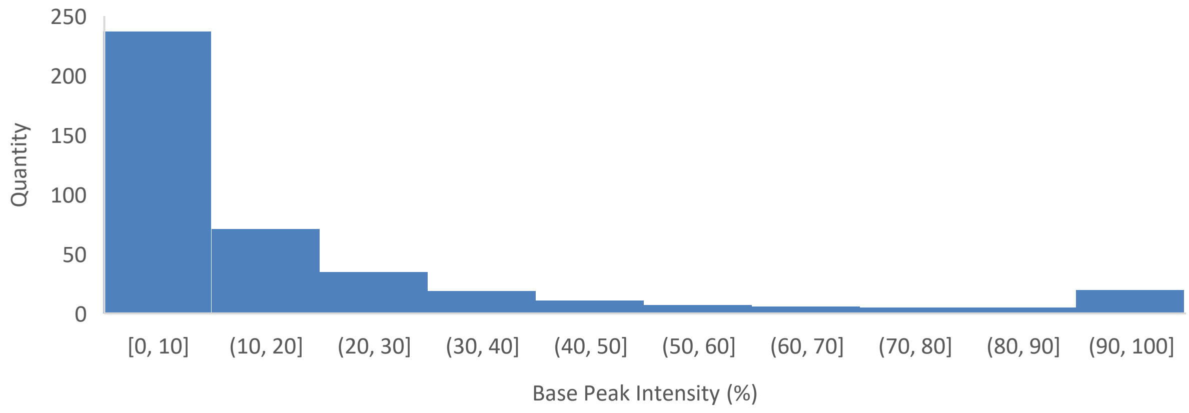

Figure S26: Quantity of calculated base peak intensities (%) of peaks which have been found to be related to reactions of the additive with the cooperative pair.

These peaks were selected and used for further manual analysis. The aim of this analysis is to postulate structural guesses for products which can be verified in a further step. Spectra were background corrected and evaluated manually again. On the one hand, cleaned spectra were compared with the NIST 2017 database and products with high similarity were suggested, while on the other hand, product proposals were designed based on the structures of the starting materials and reaction possible mechanisms while checking likely product fragmentations against the spectra. In general, it must be pointed out that due to the large amount of data and reaction hits, no isolations and confirmations of the structures could be carried out. Especially with regard to different isomers, only limited statements can be made on the basis of the GC-

MS spectra. In some cases, only sum formulas or product motives could be determined. To confirm postulated structures with regard to their elemental composition i.e. their sum formular ESI-high resolution mass spectra were measured and checked for this exact mass and isotope ratio.

All raw data regarding GC-MS and ESI-HRMS is available in the git repository at the path **GC-MS-Plate-Analyser/raw/**. . GC-MS data is separated by the machines used for measurement (5, 6) and is given in an Agilent file format. ESI-HRMS is available as Bruker .raw file formate which can be read by various open-source software like OpenChrom.[27]

5.2.5 Results

Overall, 50 of the 156 conducted reactions show no cooperativity-based, acceptor-related reactivity meaning that every peak detected by GC-MS was present in corresponding controls. Of the remaining 106 reactions 44 showed only traces (<10% BPI) of relevant products while it has to be mentioned that also spectrum comparison becomes unreliable for these peaks. The main fraction, namely 62 of the 156 conducted reactions (40%) showed clear peaks of cooperativity-based reactivity as described above (Figure S27). For 26 of these 62 reactions, product structures or structural motives have been proposed. Thereby, 22 product structures could be proposed, while in one case, only a core scaffold was proposed due to high product complexity. 20 (90%) products, more precise sum formulas of the products could be confirmed by means of ESI-HRMS. In two cases, the identification was not possible, most likely due to the missing polarity of the proposed structure. Some products occur in reactions with different cooperative pairs (5.2.6) as found by spectra and retention time comparison (5.2.4). Exact mass and thereby the sum formular was searched and confirmed only once for every product.

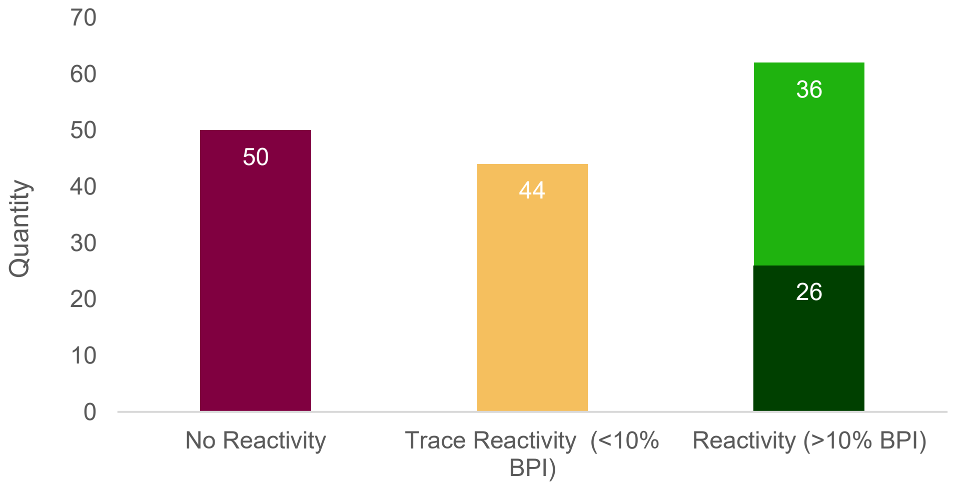

Figure S27: Quantity of reactions showing no (red), low (yellow) or significant (green) cooperativity-based reactivity with chosen acceptors. Reactions where at least one product structure was prosed are highlighted (light green).

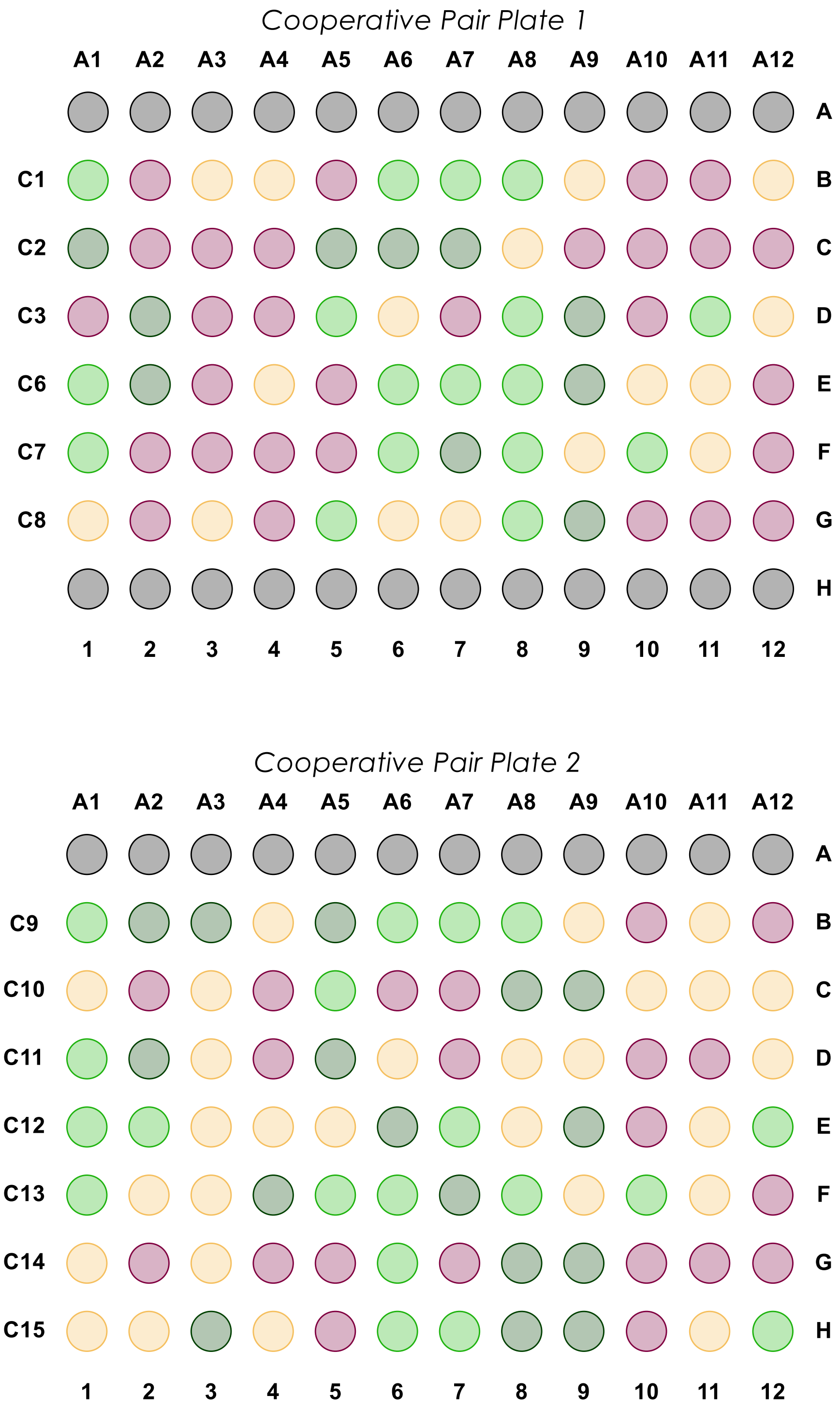

Figure S28: Classified reaction outcome for all conducted reactions. Classes are "no reactivity" (red), "low reactivity" (yellow) or "significant reactivity" (green) by the means of above mentioned criteria for reactivity. Reactions where at least one product structure was prosed are highlighted (light green).

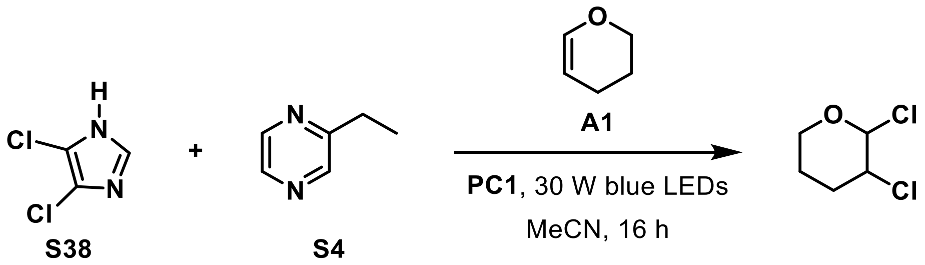

Scheme S2: Proposed reaction of **C1** with **A1**. Product as proposed based on GC-MS (RT = 2.737 min). **HRMS (ESI)** (*m/z*): $[M + H]^+$ calcd. for $C_5H_8OCl_2H^+$ = 155.0025; found: 155.0020.

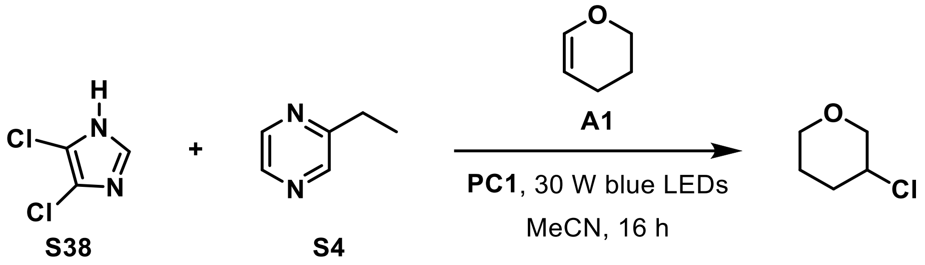

Scheme S3: Proposed reaction of **C1** with **A1**. Product as proposed based on GC-MS (RT = 2.796 min). **HRMS (ESI)** (*m/z*): $[M + Na]^+$ calcd. for $C_5H_9OClNa^+$ = 143.0234; found: 143.0232.

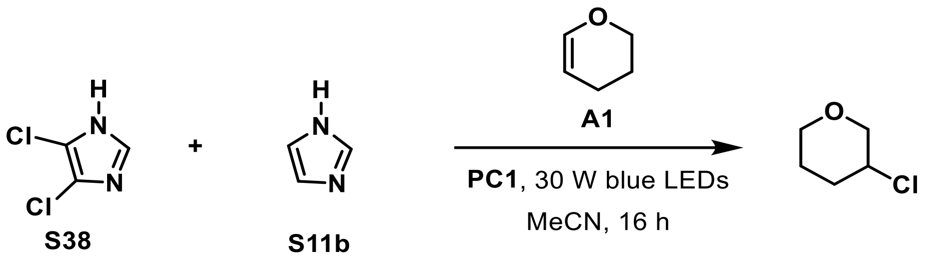

Scheme S4: Proposed reaction of **C6** with **A1**. Product as proposed based on GC-MS (RT = 2.796 min). **HRMS (ESI)** (*m/z*): $[M + Na]^+$ calcd. for $C_5H_9OClNa^+$ = 143.0234; found: 143.0232.

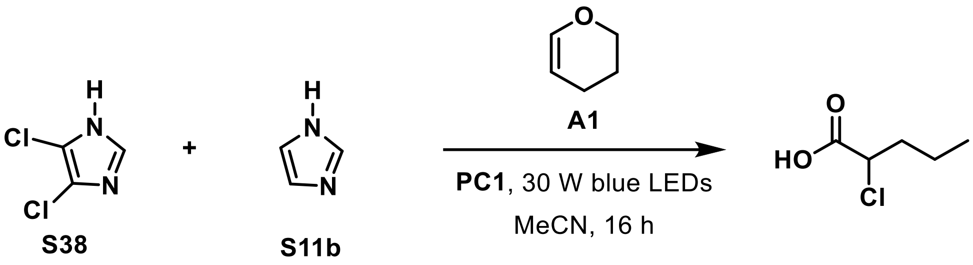

Scheme S5: Proposed reaction of **C6** with **A1**. Product as proposed based on GC-MS (RT = 2.761 min). **HRMS (ESI)** (*m/z*): $[M + Na]^+$ calcd. for $C_5H_9O_2ClNa^+$ = 159.0183; found: 3 159.0183.

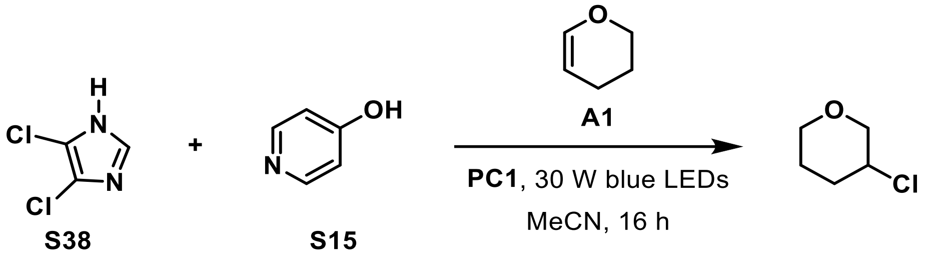

Scheme S6: Proposed reaction of **C7** with **A1**. Product as proposed based on GC-MS (RT = 2.831 min). **HRMS (ESI)** (*m/z*): $[M + Na]^+$ calcd. for $C_5H_9OClNa^+$ = 143.0234; found: 143.0232.

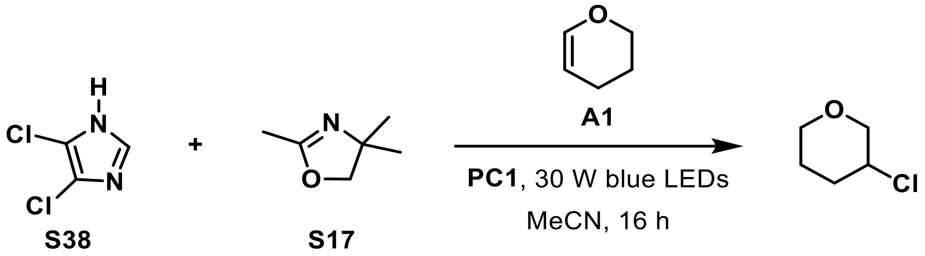

Scheme S7: Proposed reaction of **C9** with **A1**. Product as proposed based on GC-MS (RT = 2.796 min). **HRMS (ESI)** (*m/z*): $[M + Na]^+$ calcd. for $C_5H_9OClNa^+$ = 143.0234; found: 143.0232.

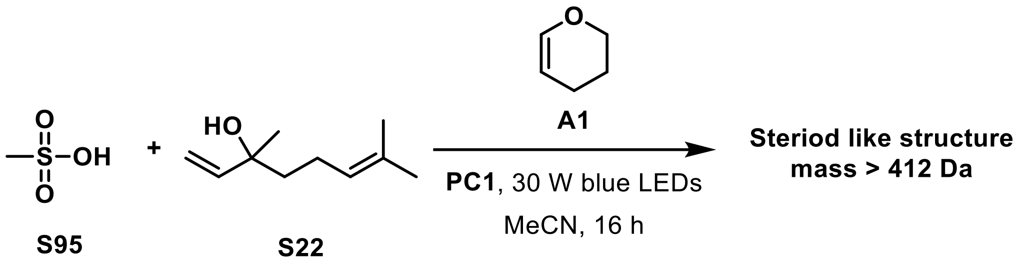

Scheme S8: Proposed reaction of **C11** with **A1**. Database searches suggest a steroid like structure product as major product (RT = 5.340 min). Conformation of the sum formular by accurate mass was not possible.

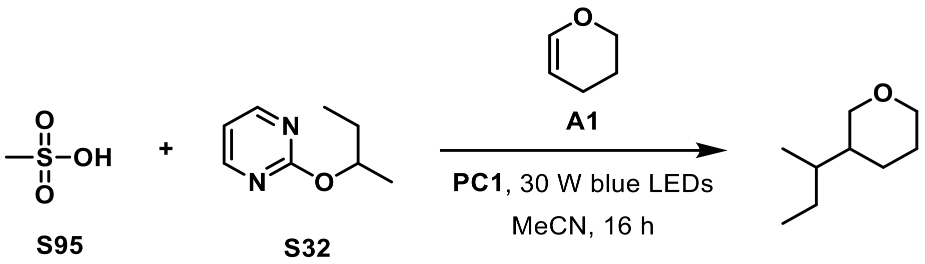

Scheme S9: Proposed reaction of **C12** with **A1**. Product as proposed based on GC-MS (RT = 2.972min). **HRMS (ESI)** (*m/z*): $[M + Na]^+$ calcd. for $C_9H_{18}ONa^+$ = 165.1250; found: 165.1260.

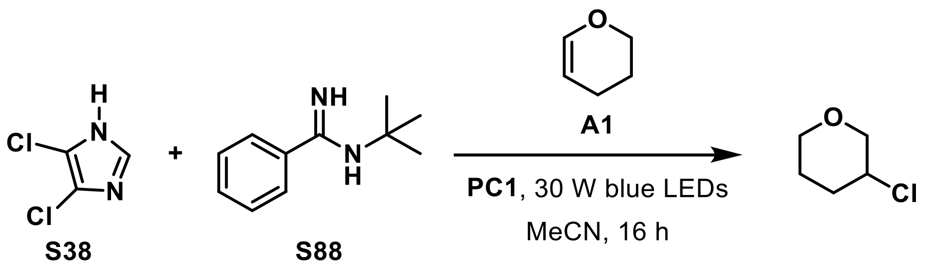

Scheme S10: Proposed reaction of **C13** with **A1**. Product as proposed based on GC-MS (RT = 2.831 min). **HRMS (ESI)** (*m/z*): $[M + Na]^+$ calcd. for $C_5H_9OClNa^+$ = 143.0234; found: 143.0232.

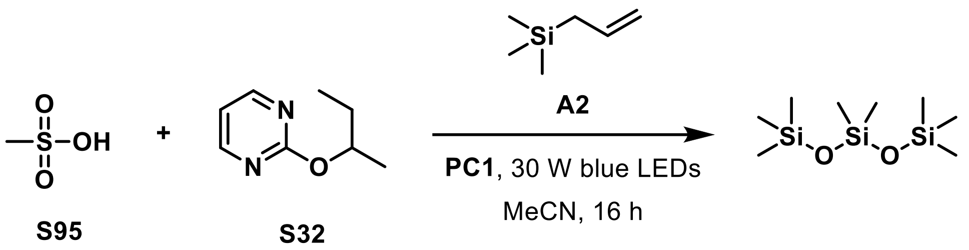

Scheme S11: Proposed reaction of **C12** with **A2**. Product as proposed based on GC-MS (RT = 2.321 min). **HRMS (ESI)** (*m/z*): $[M + Na]^+$ calcd. for $C_8H_{24}O_2Si_3Na^+$ = 259.0976; found: 259.0982.

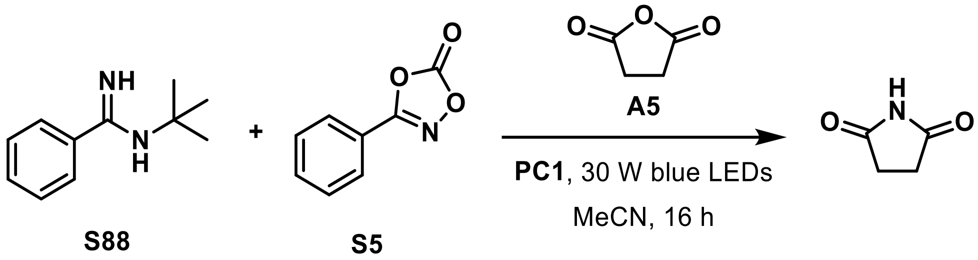

Scheme S12: Proposed reaction of **C3** with **A5**. Product as proposed based on GC-MS (RT = 2.802 min). **HRMS (ESI)** (*m/z*): $[M + Na]^+$ calcd. for $C_4H_5NO_2Na^+$ = 122.0212; found: 122.0211.

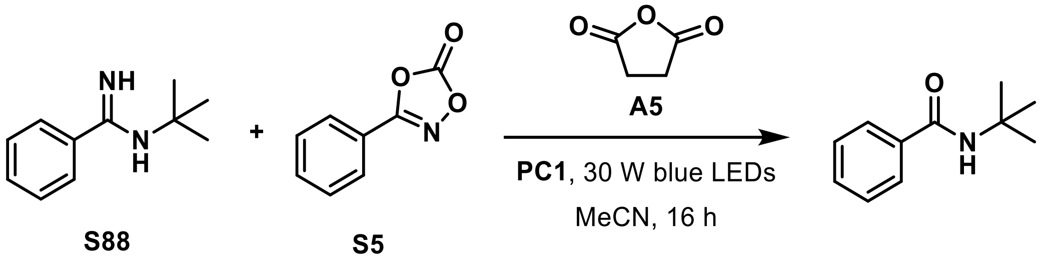

Scheme S13: Proposed reaction of **C3** with **A5**. Product as proposed based on GC-MS (RT = 3.294 min). **HRMS (ESI)** (*m/z*): $[M + H]^+$ calcd. for $C_{11}H_{15}NOH^+$ = 178.1226; found: 178.1226.

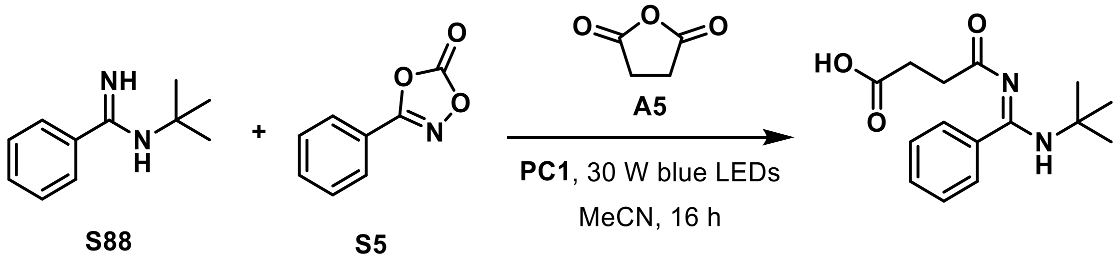

Scheme S14: Proposed reaction of **C3** with **A5**. Product as proposed based on GC-MS (RT = 3.986 min). **HRMS (ESI)** (*m/z*): $[M + Na]^+$ calcd. for $C_{15}H_{20}N_2O_2Na^+$ = 299.1366; found: 299.1366.

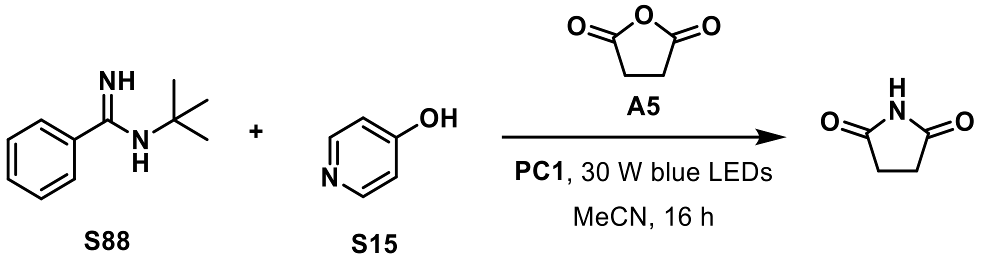

Scheme S15: Proposed reaction of **C8** with **A5**. Product as proposed based on GC-MS (RT = 2.825 min). **HRMS (ESI)** (*m/z*): $[M + Na]^+$ calcd. for $C_4H_5NO_2Na^+$ = 122.0212; found: 122.0211.

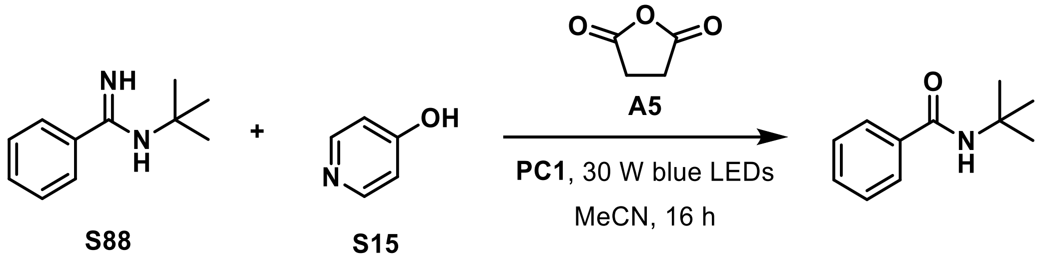

Scheme S16: Proposed reaction of **C8** with **A5**. Product as proposed based on GC-MS (RT = 3.306 min). **HRMS (ESI)** (*m/z*): $[M + H]^+$ calcd. for $C_{11}H_{15}NOH^+$ = 178.1226; found: 178.1226.

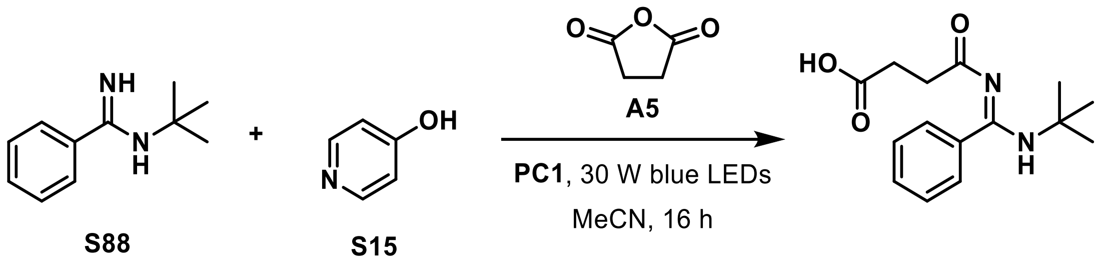

Scheme S17: Proposed reaction of **C8** with **A5**. Product as proposed based on GC-MS (RT = 3.997 min). **HRMS (ESI)** (*m/z*): $[M + Na]^+$ calcd. for $C_{15}H_{20}N_2O_2Na^+$ = 299.1366; found: 299.1366.

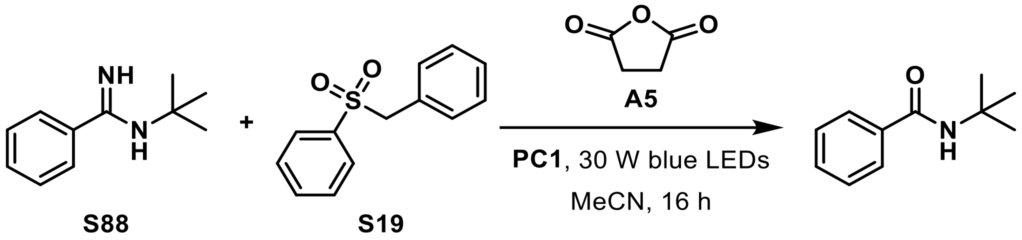

Scheme S18: Proposed reaction of **C10** with **A5**. Product as proposed based on GC-MS (RT = 3.294 min). **HRMS (ESI)** (*m/z*): $[M + H]^+$ calcd. for $C_{11}H_{15}NOH^+$ = 178.1226; found: 178.1226.

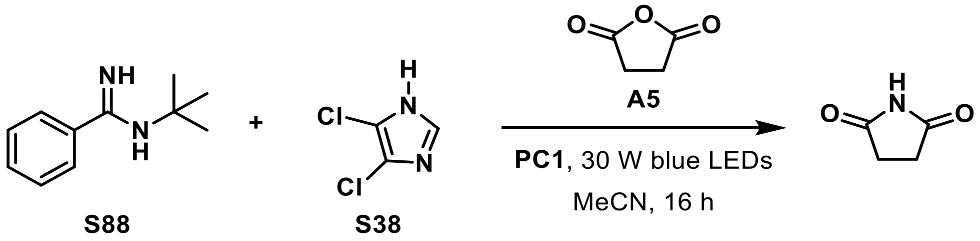

Scheme S19: Proposed reaction of **C13** with **A5**. Product as proposed based on GC-MS (RT = 2.825 min). **HRMS (ESI)** (*m/z*): $[M + Na]^+$ calcd. for $C_4H_5NO_2Na^+$ = 122.0212; found: 122.0211.

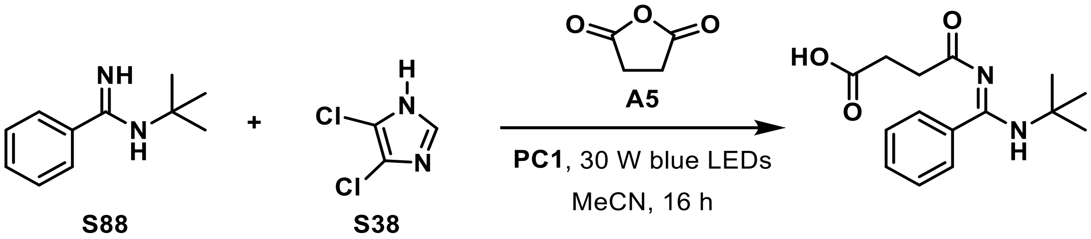

Scheme S20: Proposed reaction of **C13** with **A5**. Product as proposed based on GC-MS (RT = 3.986 min). **HRMS (ESI)** (*m/z*): $[M + Na]^+$ calcd. for $C_{15}H_{20}N_2O_2Na^+$ = 299.1366; found: 299.1366.

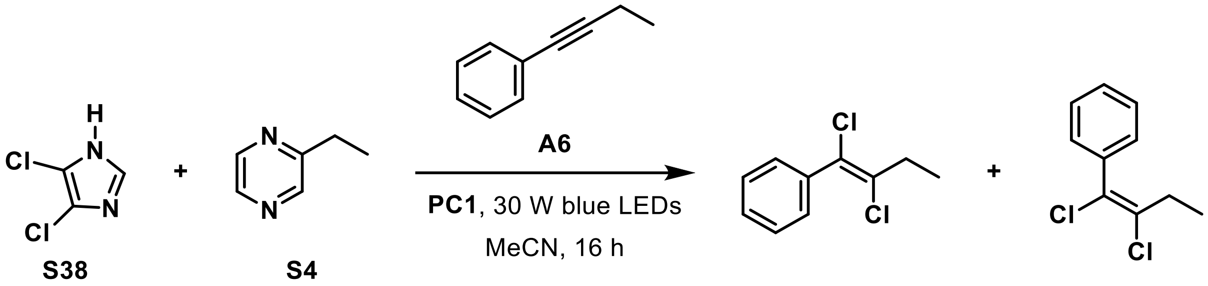

Scheme S21: Proposed reaction of **C1** with **A6**. Product as proposed based on GC-MS (RT = 3.171 min, 3.230 min). **HRMS (ESI)** (*m/z*): $[M + Na]^+$ calcd. for $C_{10}H_{10}Cl_2Na^+$ = 223.0052; found: 223.0049.

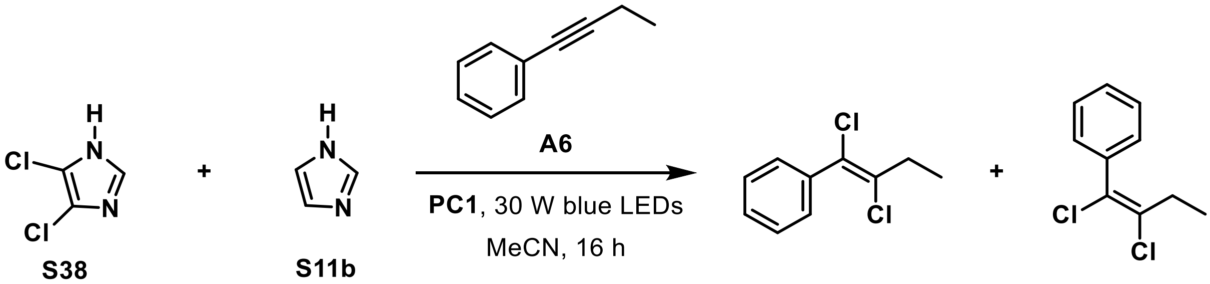

Scheme S22: Proposed reaction of **C6** with **A6**. Product as proposed based on GC-MS (RT = 3.171 min, 3.230 min). **HRMS (ESI)** (*m/z*): $[M + Na]^+$ calcd. for $C_{10}H_{10}Cl_2Na^+$ = 223.0052; found: 223.0049.

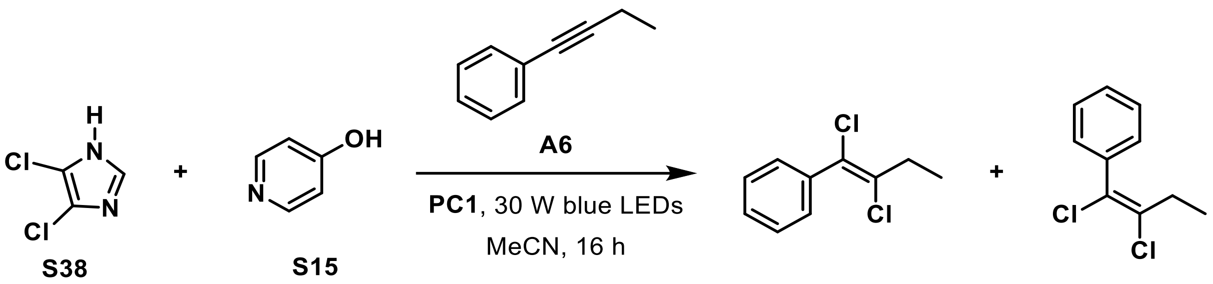

Scheme S23: Proposed reaction of **C7** with **A6**. Product as proposed based on GC-MS (RT = 3.200 min, 3.253 min). **HRMS (ESI)** (*m/z*): $[M + Na]^+$ calcd. for $C_{10}H_{10}Cl_2Na^+$ = 223.0052; found: 223.0049.

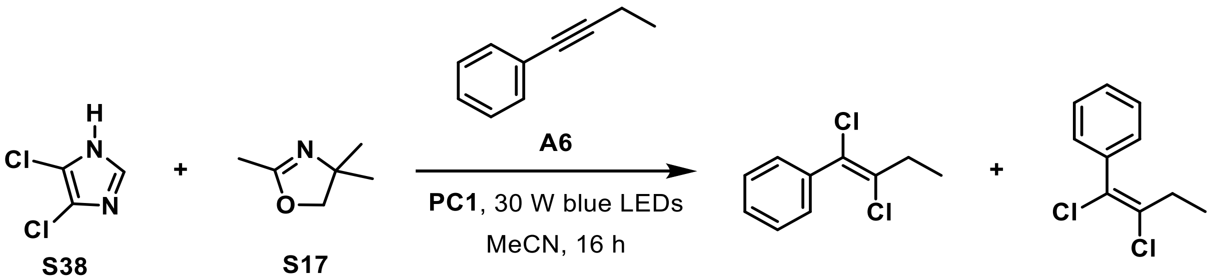

Scheme S24: Proposed reaction of **C9** with **A6**. Product as proposed based on GC-MS (RT = 3.171 min, 3.230 min). **HRMS (ESI)** (*m/z*): $[M + Na]^+$ calcd. for $C_{10}H_{10}Cl_2Na^+$ = 223.0052; found: 223.0049.

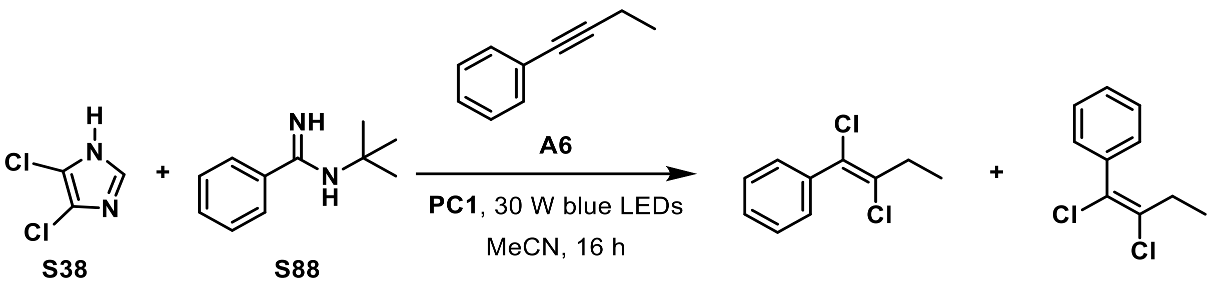

Scheme S25: Proposed reaction of **C13** with **A6**. Product as proposed based on GC-MS (RT = 3.200 min, 3.253 min). **HRMS (ESI)** (*m/z*): $[M + Na]^+$ calcd. for $C_{10}H_{10}Cl_2Na^+$ = 223.0052; found: 223.0049.

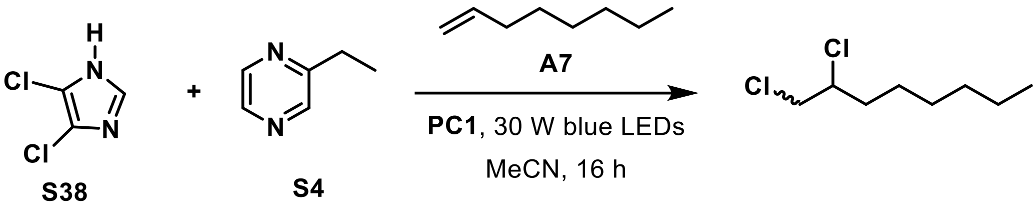

Scheme S26: Proposed reaction of **C1** with **A7**. Product as proposed based on GC-MS (RT = 2.937 min). **HRMS (ESI)** (*m/z*): $[M + H]^+$ calcd. for $C_8H_{14}Cl_2H^+$ = 183.0702; found: 183.0706.

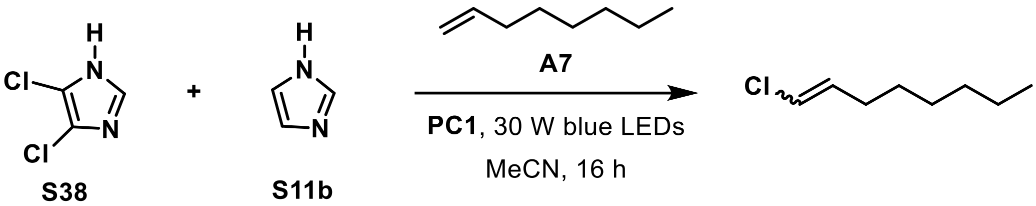

Scheme S27: Proposed reaction of **C6** with **A7**. Product as proposed based on GC-MS (RT = 2.696 min). $[M + H]^+$ and $[M + Na]^+$ not found due to missing polar groups.

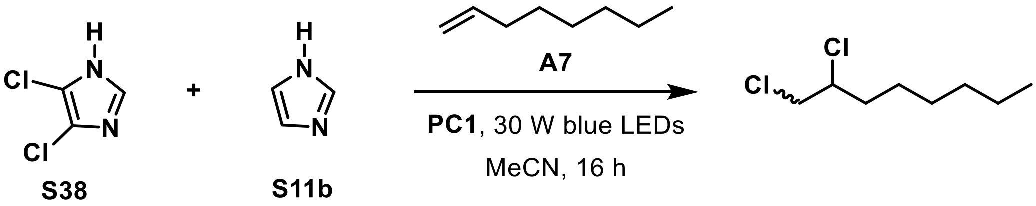

Scheme S28: Proposed reaction of **C6** with **A7**. Product as proposed based on GC-MS (RT = 2.943 min). **HRMS (ESI)** (*m/z*): $[M + H]^+$ calcd. for $C_8H_{14}Cl_2H^+$ = 183.0702; found: 183.0706.

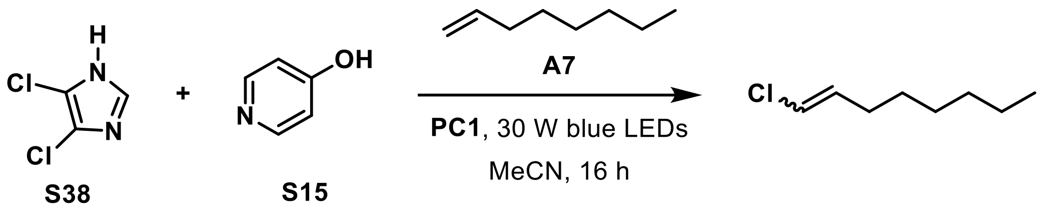

Scheme S29: Proposed reaction of **C9** with **A7**. Product as proposed based on GC-MS (RT = 2.726 min). $[M + H]^+$ and $[M + Na]^+$ not found due to missing polar groups.

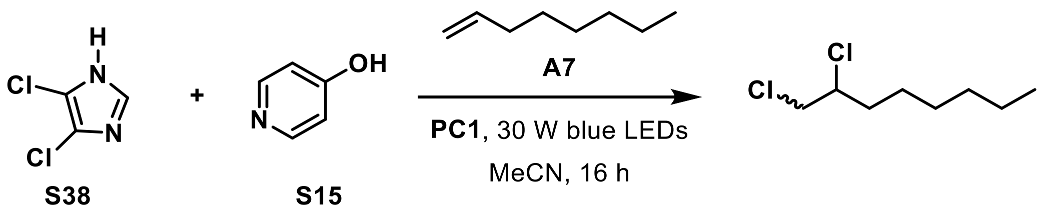

Scheme S30: Proposed reaction of **C7** with **A7**. Product as proposed based on GC-MS (RT = 2.966 min). **HRMS (ESI)** (*m/z*): $[M + H]^+$ calcd. for $C_8H_{14}Cl_2H^+$ = 183.0702; found: 183.0706.

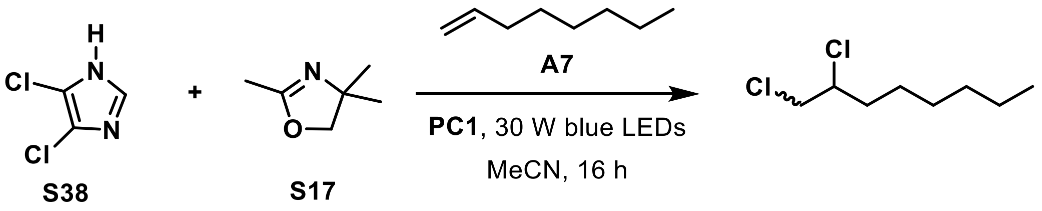

Scheme S31: Proposed reaction of **C9** with **A7**. Product as proposed based on GC-MS (RT = 2.942 min). **HRMS (ESI)** (*m/z*): $[M + H]^+$ calcd. for $C_8H_{14}Cl_2H^+$ = 183.0702; found: 183.0706.

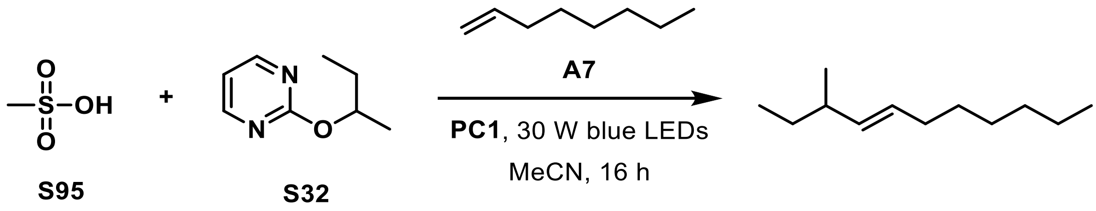

Scheme S32: Proposed reaction of **C12** with **A7**. Product as proposed based on GC-MS (RT = 3.312 min). $[M + H]^+$ and $[M + Na]^+$ not found due to missing polar groups.

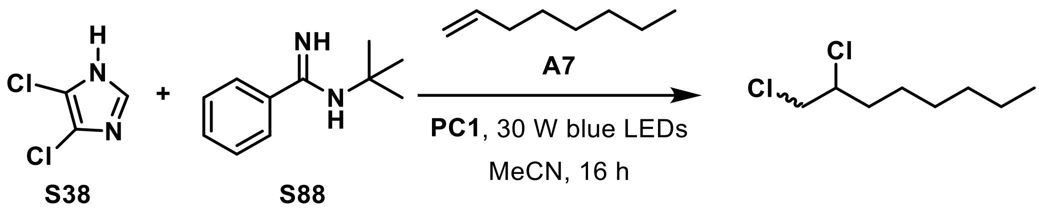

Scheme S33: Proposed reaction of **C13** with **A7**. Product as proposed based on GC-MS (RT = 2.966 min). **HRMS (ESI)** (*m/z*): $[M + H]^+$ calcd. for $C_8H_{14}Cl_2H^+$ = 183.0702; found: 183.0706.

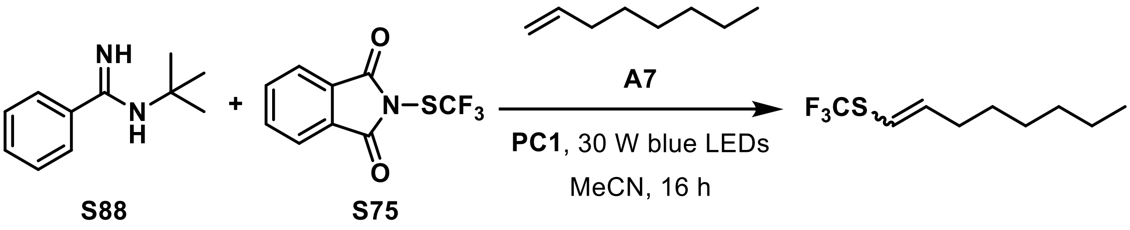

Scheme S34: Proposed reaction of **C15** with **A7**. Product as proposed based on GC-MS (RT = 2.719 min). **HRMS (ESI)** (*m/z*): $[M + Na]^+$ calcd. for $C_9H_{15}F_3SNa^+$ = 213.0919; found: 213.0921.

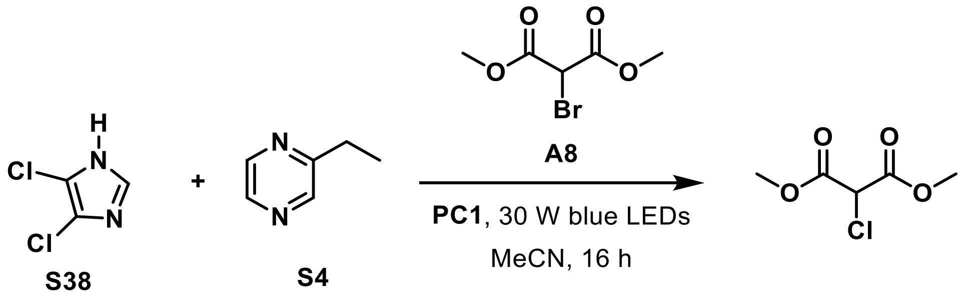

Scheme S35: Proposed reaction of **C1** with **A8**. Product as proposed based on GC-MS (RT = 2.907 min). **HRMS (ESI)** (*m/z*): $[M + H]^+$ calcd. for $C_5H_7O_4ClH^+$ = 167.0106; found: 167.0103.

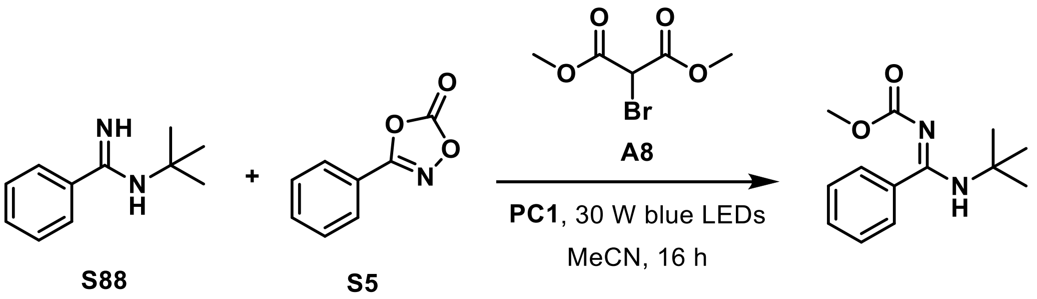

Scheme S36: Proposed reaction of **C3** with **A8**. Product as proposed based on GC-MS (RT = 4.085 min). **HRMS (ESI)** (*m/z*): $[M + Na]^+$ calcd. for $C_{13}H_{18}N_2O_2Na^+$ = 257.1260; found: 257.1265.

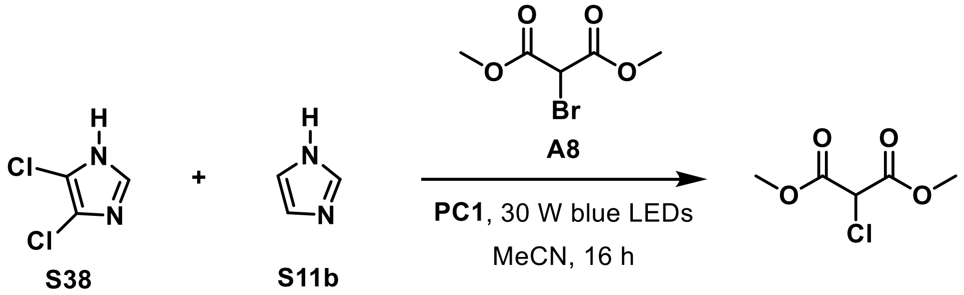

Scheme S37: Proposed reaction of **C6** with **A8**. Product as proposed based on GC-MS (RT = 2.907 min). **HRMS (ESI)** (*m/z*): $[M + H]^+$ calcd. for $C_5H_7O_4ClH^+$ = 167.0106; found: 167.0103.

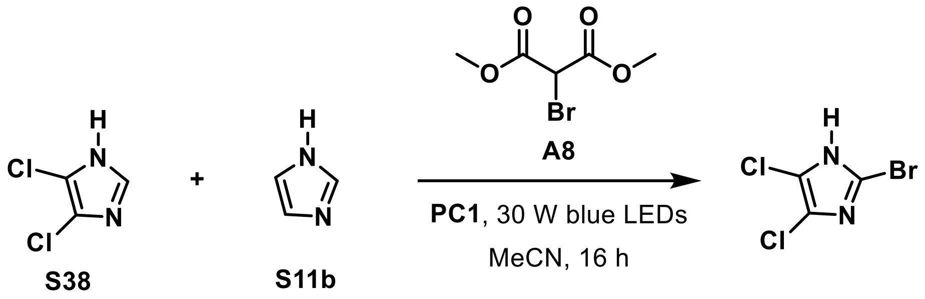

Scheme S38: Proposed reaction of **C6** with **A8**. Product as proposed based on GC-MS (RT = 3.341 min). **HRMS (ESI)** (*m/z*): $[M + Na]^+$ calcd. for $C_3HN_2ClBrNa^+$ = 214.8773; found: 214.8773.

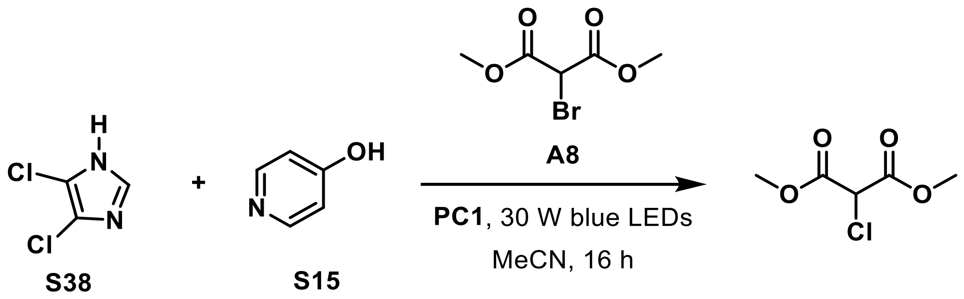

Scheme S39: Proposed reaction of **C7** with **A8**. Product as proposed based on GC-MS (RT = 2.931 min). **HRMS (ESI)** (*m/z*): $[M + H]^+$ calcd. for $C_5H_7O_4ClH^+$ = 167.0106; found: 167.0103.

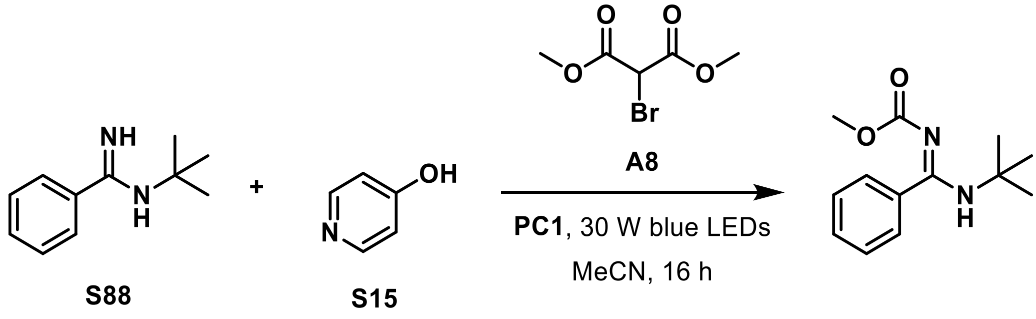

Scheme S40: Proposed reaction of **C8** with **A8**. Product as proposed based on GC-MS (RT = 4.091 min). **HRMS (ESI)** (*m/z*): $[M + Na]^+$ calcd. for $C_{13}H_{18}N_2O_2Na^+$ = 257.1260; found: 257.1265.

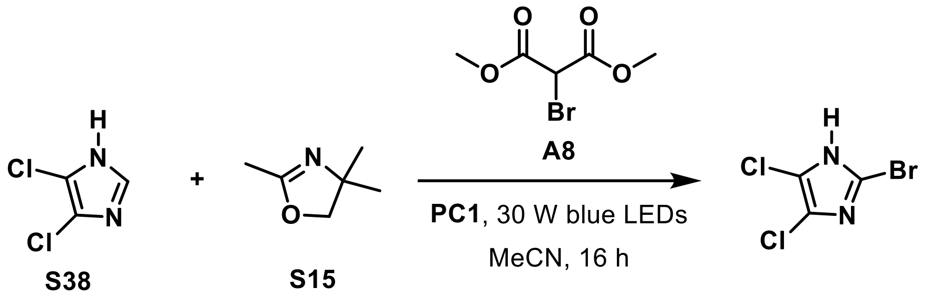

Scheme S41: Proposed reaction of **C9** with **A8**. Product as proposed based on GC-MS (RT = 3.335 min). **HRMS (ESI)** (*m/z*): $[M + Na]^+$ calcd. for $C_3HN_2ClBrNa^+$ = 214.8773; found: 214.8773.

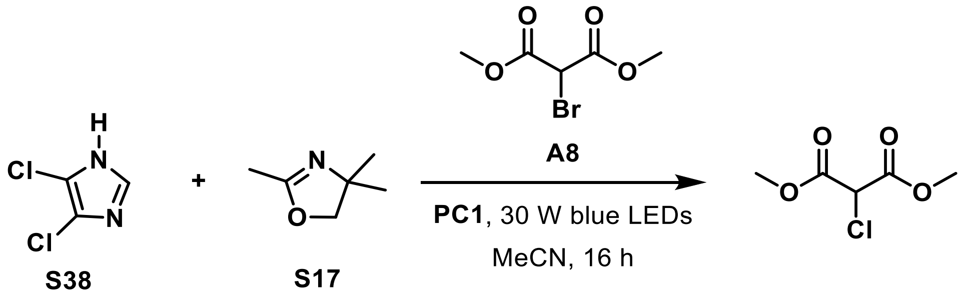

Scheme S42: Proposed reaction of **C9** with **A8**. Product as proposed based on GC-MS (RT = 2.907 min). **HRMS (ESI)** (*m/z*): $[M + H]^+$ calcd. for $C_5H_7O_4ClH^+$ = 167.0106; found: 167.0103.

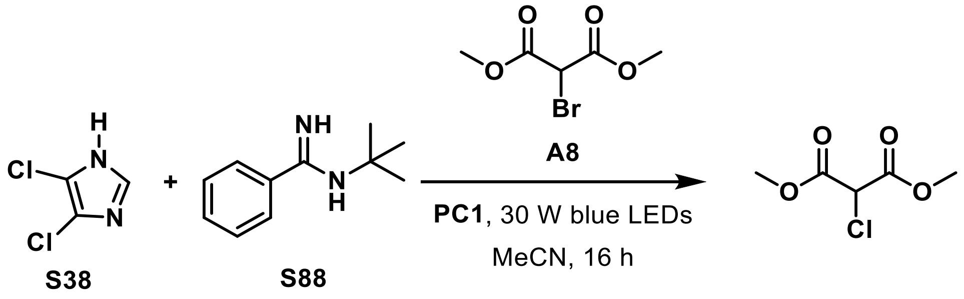

Scheme S43: Proposed reaction of **C13** with **A8**. Product as proposed based on GC-MS (RT = 2.931 min). **HRMS (ESI)** (*m/z*): $[M + H]^+$ calcd. for $C_5H_7O_4ClH^+$ = 167.0106; found: 167.0103.

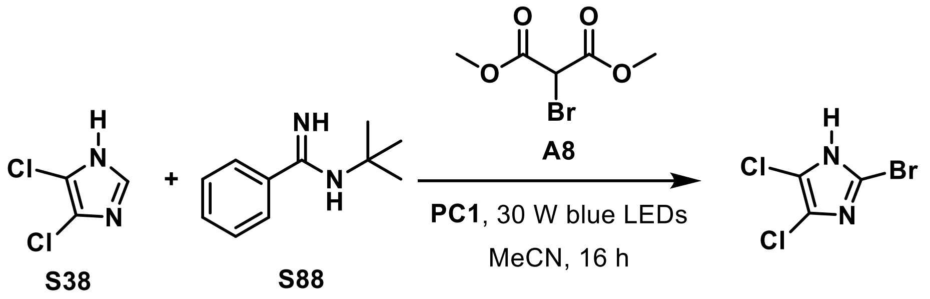

Scheme S44: Proposed reaction of **C13** with **A8**. Product as proposed based on GC-MS (RT = 3.341 min). **HRMS (ESI)** (*m/z*): $[M + Na]^+$ calcd. for $C_3HN_2ClBrNa^+$ = 214.8773; found: 214.8773.

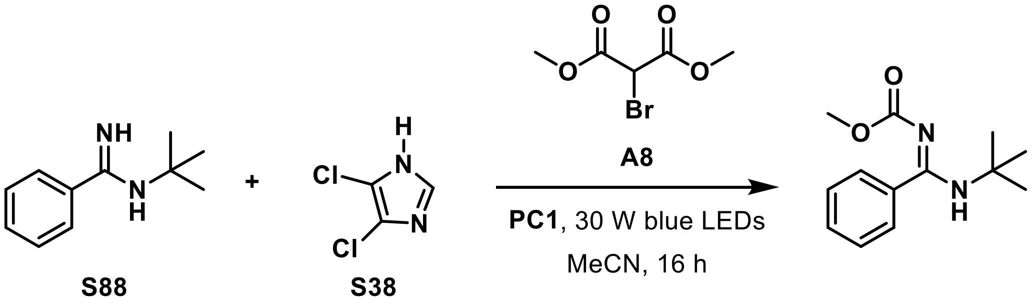

Scheme S45: Proposed reaction of **C13** with **A8**. Product as proposed based on GC-MS (RT = 4.091 min). **HRMS (ESI)** (*m/z*): $[M + Na]^+$ calcd. for $C_{13}H_{18}N_2O_2Na^+$ = 257.1260; found: 257.1265.

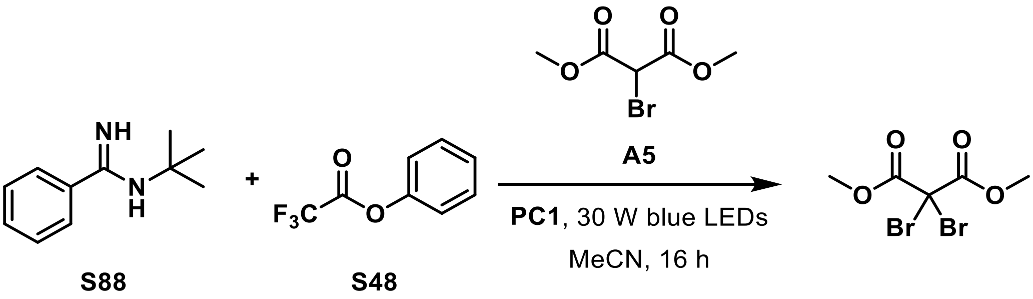

Scheme S46: Proposed reaction of **C14** with **A5**. Product as proposed based on GC-MS (RT = 3.288 min). **HRMS (ESI)** (*m/z*): $[M + Na]^+$ calcd. for $C_5H_6O_4Br_2Na^+$ = 288.8702; found: 288.8702.

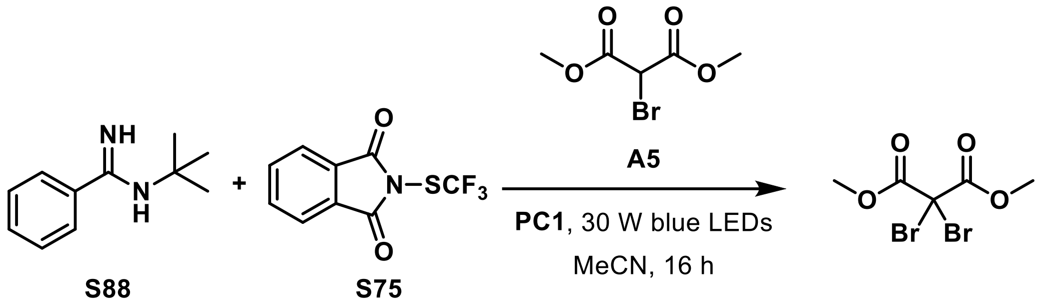

Scheme S47: Proposed reaction of **C15** with **A5**. Product as proposed based on GC-MS (RT = 3.288 min). **HRMS (ESI)** (*m/z*): $[M + Na]^+$ calcd. for $C_5H_6O_4Br_2Na^+$ = 288.8702; found: 288.8702.

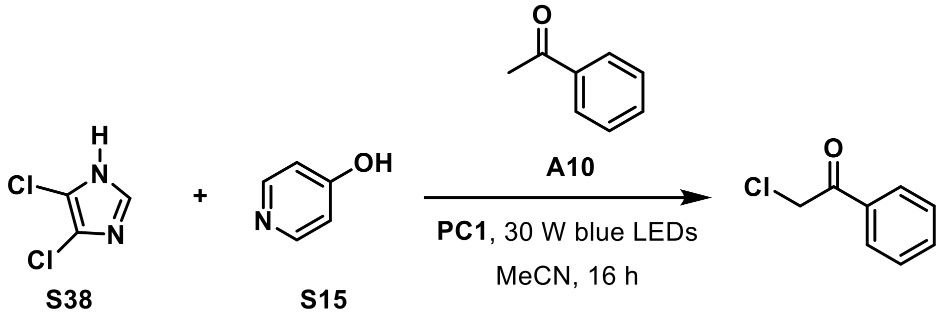

Scheme S48: Proposed reaction of **C7** with **A10**. Product as proposed based on GC-MS (RT = 3.206 min). **HRMS (ESI)** (*m/z*): $[M + Na]^+$ calcd. for $C_8H_7OClNa^+$ = 177.0078; found: 177.0075.

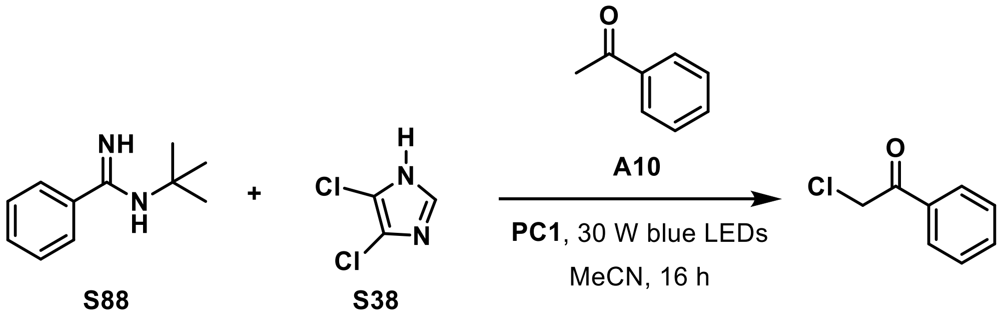

Scheme S49: Proposed reaction of **C13** with **A10**. Product as proposed based on GC-MS (RT = 3.206 min). **HRMS (ESI)** (*m/z*): $[M + Na]^+$ calcd. for $C_8H_7OClNa^+$ = 177.0078; found: 177.0075.

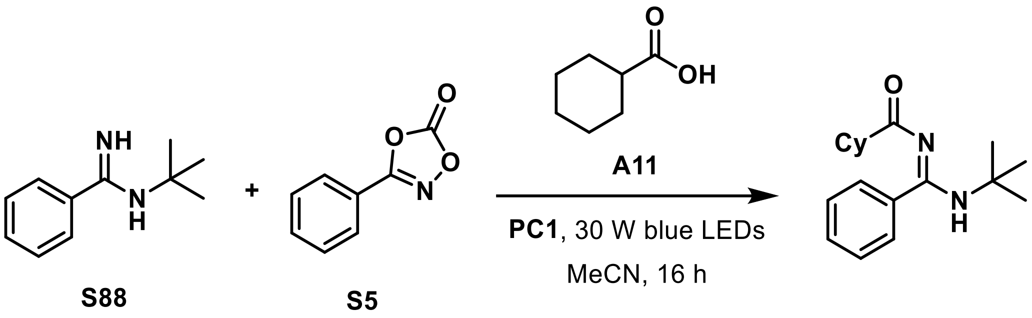

Scheme S50: Proposed reaction of **C3** with **A11**. Product as proposed based on GC-MS (RT = 4.285 min). **HRMS (ESI)** (*m/z*): [M + Na]$^+$ calcd. for $C_{18}H_{26}N_2ONa^+$ = 309.1937; found: 309.1927.

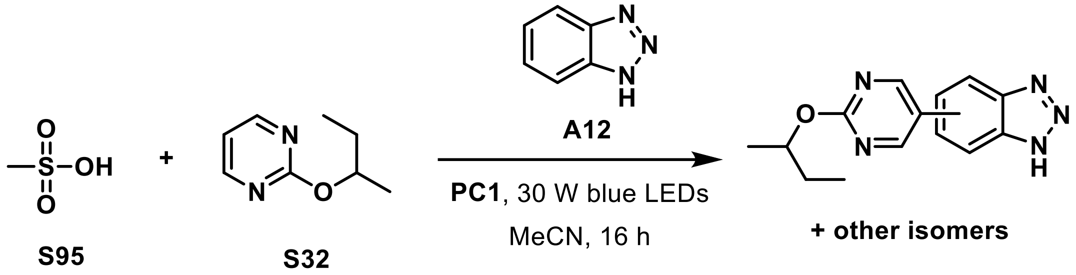

Scheme S51: Proposed reaction of **C12** with **A12**. Product as proposed based on GC-MS (RT = 4.332 min, 4.466 min). **HRMS (ESI)** (*m/z*): [M + Na]$^+$ calcd. for $C_{14}H_{15}N_5ONa^+$ = 292.1169; found: 292.1164.

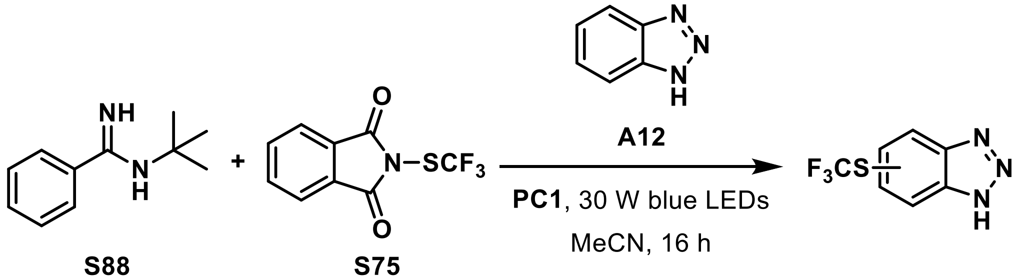

Scheme S52: Proposed reaction of **C15** with **A12**. Product as proposed based on GC-MS (RT = 3.370 min). **HRMS (ESI)** (*m/z*): [M + Na]$^+$ calcd. for $C_7H_4N_3F_3SNa^+$ = 241.9970; found: 241.9970.

5.2.6 Conclusion

In general, the performed reaction screen evidenced reactivity for each of the 13 tested pairs and enabled postulation of product structures for 11 pairs. Thereby, previously observed similarities in the cause and nature of the cooperative interaction are confirmed, especially with respect to pairs with **S38** as a substrate. Thus, identical product structures have been found repeatedly for pairs **C1**, **C6**, (**C7**), **C9**, and **C11**. The same applies to the pairs **C3**, **C8** and **C11** which all contain substrate **S88**. Interestingly, however, there are also clear quantitative and qualitative differences in reactivity, i.e. in the type and amount of product(s) formed within these pair groups. It is likely here, for example, that the cooperative interaction within the pair leads to activation of only one of the involved substrates (**S38**, **S88**) towards the photocatalyst, thus preparing this substrate for further reactions. In this way, it can be proposed that the strength and nature of the activation can be tuned (changed) by the choice of the cooperative partner, which also results in a change in reactivity.

In this context, the type and selectivity of the reactivities found varied considerably. On the one hand, different pairs for the efficient release of chlorine (**C1**, **C6**, **C7**, **C9**, **C11**) and SCF_3 radicals (**C15**) could be found and on the other hand substrates could be activated as a whole and coupled with acceptors (i.e. scheme S38, S51). In the case of some reactions, a product could be formed particularly efficiently (100% BPI) (i.e. Scheme S8, S35, S37, S39 S42, S52) while for other transformations both chemo- and regiounselective processes were found (i.e. Scheme S2 vs. S3, S27 vs. S28). It has to be pointed out that within the scope of this project, product suggestions cannot be made for all peaks and many reactivities are still unclear even though high efforts were involve in product identification. As an example, for the reaction of **C1** with **A2** (Scheme S11), a degradation product of the acceptor could be identified, but no major product of the activated substrate (**S32**) with the radical acceptor **A2** has been found.

It has to be mentioned once again that all proposed structural motifs were postulated on the basis of GC-MS and ESI-HRMS data, but in the future an isolation of the products has to be performed in order to clarify the structure with certainty. Due to the large number of reactions found, this is not possible within the scope of this comprehensive work. In addition, since only a minority of all products were elucidated other methods should be considered to identify more reactivities. In some cases (**C11**, **A1**) the selective formation of a complex reaction product could be detected (i.e. Scheme S8) but no concrete structure proposal could be made. In many other cases, despite intensive elucidation and good peak intensity/resolution, no rational product

proposal could be made to found product spectra. This speaks, as in the case of **C11** and **A1**, for an unexpected or particularly complex, and thus difficult to predict or design reactivity.

Altogether, reactivity has been detected and products identified for all pairs tested. It is particularly surprising that all acceptors were also involved in reactions, which need to be explained by the high reactivity of the pairs found. This reactivity and following hit rate is significantly higher than in comparable approaches.[25] Although reaction products could not be identified in all cases, by carrying out control reactions and automatic evaluation of the analytical data, it could be shown that the cooperative pairs are involved in unique reactions with tested acceptors, which the corresponding single substances do not possess. It is striking that even pairs with similar activation mechanisms show slightly different reactivities (i.e. selectivities and product quantities), which suggests a simultaneous tuneability of activity and reactivity. In general, the observations from the decomposition studies can be confirmed, with reactions also detected for non-decomposing pairs. This, the overall high reactivity (hit rate) and multiple acceptor possibilities give an interesting starting point for the development of further and synthetically useful reactions. This was done within the framework of this work using a proof-of-principle (**C15**, 6.0 to 8.0). Everything else goes far beyond the scope of this study and is left open for the interested reader. Further reaction developments would lead, from the point of view of the first author, to an absurdity of this work which deals with highly systematic approaches to the exploration of the high-dimensional chemical (reaction) space and not with rather less systematic rational designs.

6. SCF_3-Amidines

6.1 Preparation of Starting Materials

6.1.1 Synthesis of Amidines 1a to 1g.

General Procedure A

Fresh and water free $AlCl_3$ (1.0 eq) was placed in a pressure tube with the solid nitrile (1.0 eq). Liquid nitriles were added dropwise. After completion of the following heat release (in case of liquid nitriles), the water free amine (1.3 eq) was added quickly and in one portion. Some amines, e.g. *tert*-butylamine, were distilled in advance to avoid low conversion. The pressure tube was sealed immediately after the addition and the resulting gel was stirred at 140 °C for 1 h under air atmosphere. Before cooling down entirely to room temperature, CH_2Cl_2 was carefully added to the gel to avoid its solidification, followed by the addition of water (5 mL/mmol) for quenching the reaction. The mixture was acidified to pH < 1 with HCl (1.0 M), the aqueous layer was washed with CH_2Cl_2 (3 x 5 mL/mmol), basified to pH 13 to 14 with NaOH (1.0 M) and re-extracted with CH_2Cl_2 (3 x 10 mL/mmol). The organic layer was dried over Na_2SO_4 and the solvent was removed *in vacuo* to give the pure product. In some cases, purification by flash column chromatography was performed to obtain the product in higher purity.

***N*-(*tert*-butyl)benzimidamide (1a)**

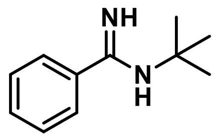

1a, 78%

Following the **general procedure A**, benzonitrile (1.89 ml, 20.0 mmol, 1.0 eq), $AlCl_3$ (2.46 g, 20.0 mmol, 1.0 eq) and *tert*-butylamine (2.70 ml, 26.0 mmol, 1.3 eq) were reacted and the crude product was purified by flash column chromatography (EtOAc:NEt_3, 49:1 v/v) to give the pure title compound **1a** as a pale yellow oil (2.76 g, 15.7 mmol, 78%).

TLC (EtOAc:NEt_3, 49:1 v/v): $\mathbf{R_f}$ = 0.20; ^{1}**H NMR** (300 MHz, $CDCl_3$) δ 7.53 – 7.45 (m, 2H), 7.40 – 7.32 (m, 3H), 1.47 (s, 9H) (N*H*-Protons not visible); ^{13}**C NMR** (75 MHz, $CDCl_3$) δ

164.4, 140.3, 129.7, 128.7, 125.9, 51.4, 28.9; **HRMS (ESI)** (*m/z*): $[M + H]^+$ calcd. for $C_{11}H_{16}N_2H$= 177.1386; found: 177.1401.

***N*-(*tert*-butyl)-4-methoxybenzimidamide (1b)**

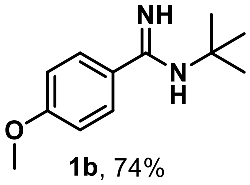

1b, 74%

Following the **general procedure A**, 4-methoxybenzonitrile (6.66 g, 50.0 mmol, 1.0 eq), $AlCl_3$ (6.67 g, 50.0 mmol, 1.0 eq) and *tert*-butylamine (6.83 mL, 65.0 mmol, 1.3 eq) were reacted and extraction gave the title compound **1b** as an off-white solid (7.60 g, 36.8 mmol, 74%).

TLC (EtOAc:NEt_3, 49:1 v/v): R_f = 0.19; ^{1}**H NMR** (300 MHz, $CDCl_3$): δ 7.57 – 7.38 (m, 2H), 6.98 – 6.77 (m, 2H), 3.82 (s, 3H), 1.48 (s, 9H) (N*H*-Protons not visible); ^{13}**C NMR** (75 MHz, $CDCl_3$): δ 160.8, 132.8, 127.4, 127.4, 113.9, 55.5, 51.4, 29.1; **HRMS (ESI)** (*m/z*): $[M + H]^+$ calcd. for $C_{12}H_{18}N_2OH$ = 207.1492; found: 207.1509.

***N*-(*tert*-butyl)-*N*-ethylbenzimidamide (1c)**

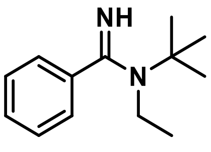

1d 28%

Following the **general procedure A**, benzonitrile (1.03 mL, 10.0 mmol, 1.0 eq), $AlCl_3$ (1.33 , 10.0 mmol, 1.0 eq) and *N*-ethyl-*tert*-butylamine (1.60 µL, 13.0 mmol, 1.3 eq) were reacted and extraction gave the title compound **1c** as a pale yellow oil (580 mg, 2.80 mmol, 28%).

TLC (EtOAc:NEt_3, 49:1 v/v): R_f = 0.24; ^{1}**H NMR** (300 MHz, $CDCl_3$): δ 7.24 – 7.28 (m, 5H), 3.10 (q, *J* = 7.0, 2H), 1.46 (s, 9H), 0.91 (t, *J* = 7.0, 3H) (N*H*-Protons not visible); ^{13}**C NMR** (75 MHz, $CDCl_3$): δ 170.6, 142.5, 128.5, 128.4, 126.7, 56.4, 42.1, 29.2, 16.9; **HRMS (ESI)** (*m/z*): $[M + H]^+$ calcd. for $C_{13}H_{20}N_2H$ = 205.1709; found: 205.1703.

***N*-(*tert*-butyl)isobutyrimidamide (1d)**

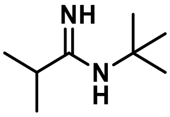

1c, 42%

Following the **general procedure A**, isobutyronitrile (449 µL, 5.00 mmol, 1.0 eq), $AlCl_3$ (617 mg, 5.00 mmol, 1.0 eq) and *tert*-butylamine (675 µL, 6.50 mmol, 1.3 eq) were reacted and extraction gave the title compound **1d** as a white solid (301 mg, 2.11 mmol, 42%).

TLC ($EtOAc:NEt_3$, 49:1 v/v): $\mathbf{R_f}$ = 0.19; ^{1}**H NMR** (300 MHz, $CDCl_3$): δ 2.18 (hept, J = 6.9 Hz, 1H), 1.40 (s, 9H), 1.13 (d, J = 6.9 Hz, 6H) (N*H*-Protons not visible); ^{13}**C NMR** (75 MHz, $CDCl_3$): δ = 155.6, 50.6, 37.0, 29.0, 20.9; **HRMS (ESI)** (*m/z*): $[M + H]^+$ calcd. for $C_8H_{18}N_2H$ = 143.1573; found: 143.1552.

***N,N*-diethylbenzimidamide (1e)**

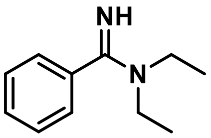

1e, 38%

Following the **general procedure A**, benzonitrile (946 µL, 10.0 mmol, 1.0 eq), $AlCl_3$ (1.33 g, 10.0 mmol, 1.0 eq) and diethylamine (1.34 mL, 13.0 mmol, 1.3 eq) were reacted and extraction gave the title compound **1e** as a pale yellow oil (668 mg, 3.79 mmol, 38%).

TLC ($EtOAc:NEt_3$, 49:1 v/v): $\mathbf{R_f}$ = 0.24; ^{1}**H NMR** (300 MHz, $CDCl_3$): 7.35 (m, 3H), 7.32 – 7.25 (m, 2H), 5.81 (s, 1H) 3.31 (q, J = 7.1 Hz, 4H), 1.12 (t, J = 7.1 Hz, 6H); ^{13}**C NMR** (75 MHz, $CDCl_3$): 168.8, 139.5, 128.5, 128.5, 126.4, 42.0, 13.3; **HRMS (ESI)** (*m/z*): $[M + H]^+$ calcd. for $C_{11}H_{16}N_2H$ 177.1386; found: 177.1402.

***N*-phenylbenzimidamide (1f)**

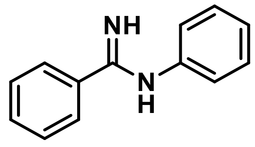

1f, 78%

Following the **general procedure A**, benzonitrile (473 µL, 5.00 mmol, 1.0 eq), $AlCl_3$ (617 mg, 5.0 mmol, 1.0 eq) and aniline (452 µL, 6.50 mmol, 1.3 eq) were reacted and extraction gave the title compound **1f** as a white solid (761 mg, 3.88 mmol, 78%).

TLC ($EtOAc:NEt_3$, 49:1 v/v): R_f = 0.23; **^{1}H NMR** (300 MHz, $CDCl_3$): δ 7.85 (d, *J* = 7.3 Hz, 2H), 7.52 – 7.41 (m, 3H), 7.35 (t, *J* = 7.8 Hz, 2H), 7.07 (tt, *J* = 7.4, 1.3 Hz, 1H), 7.02 – 6.96 (m, 2H) (N*H*-Protons not visible); **^{13}C NMR** (75 MHz, $CDCl_3$): δ 155.1, 149.5, 135.8, 130.7, 129.6, 128.7, 126.9, 123.2, 121.8; **HRMS (ESI)** (*m/z*): $[M + H]^+$ calcd. for $C_{13}H_{12}N_2H$ 197.1073; found: 197.1091.

N-ethyl-4-methoxy-*N*-methylbenzimidamide (1g)

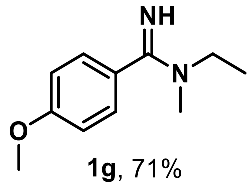

1g, 71%

Following the **general procedure A**, 4-methoxybenzonitrile (1.33 mg, 10.0 mmol, 1.0 eq), $AlCl_3$ (1.33 g, 10.0 mmol, 1.0 eq) and *N*-methylethanamine (1.72 mL, 20.0 mmol, 2.0 eq) were reacted and extraction gave the title compound **1g** as a pale yellow oil (1.37 g, 7.13 mmol, 71%).

TLC ($EtOAc:NEt_3$, 49:1 v/v): R_f = 0.22; **^{1}H NMR** (300 MHz, $CDCl_3$): δ 7.22 (m, 2H), 6.82 (m, 2H), 5.87 (s, 1H), 3.76 (s, 3H), 3.22 (q, *J* = 7.1 Hz, 2H), 2.88 (s, 3H), 1.02 (t, *J* = 7.1 Hz, 3H); **^{13}C NMR** (75 MHz, $CDCl_3$): δ 169.5, 159.9, 131.4, 128.0, 113.7, 55.3, 45.7, 35.0, 12.8; **HRMS (ESI)** (*m/z*): $[M + H]^+$ calcd. for $C_{11}H_{16}N_2OH$ 193.1335; found: 193.1357.

6.1.2 Graphical Guide for the Gram Scale Synthesis of *N*-(*tert*-butyl)-4-methoxy-benzimidamide

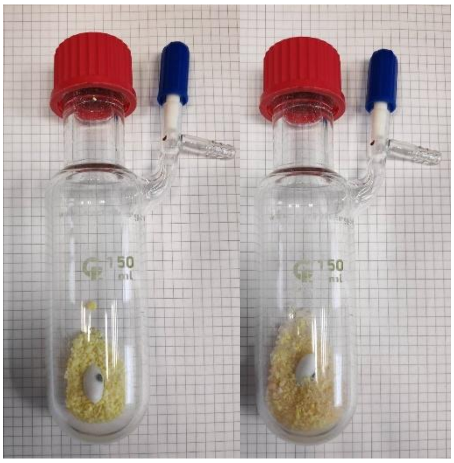

Step 1: Dry $AlCl_3$ (6.67 g, 50.0 mmol, 1.0 eq) was quickly weighed into an oven dried 150 ml screw cap pressure tube equipped with a high quality stirring bar (ellipsoidal). After the flask was cooled down to ambient temperature, 4-methoxy-benzonitrile (6.66 g, 50.0 mmol, 1.0 eq) was added.

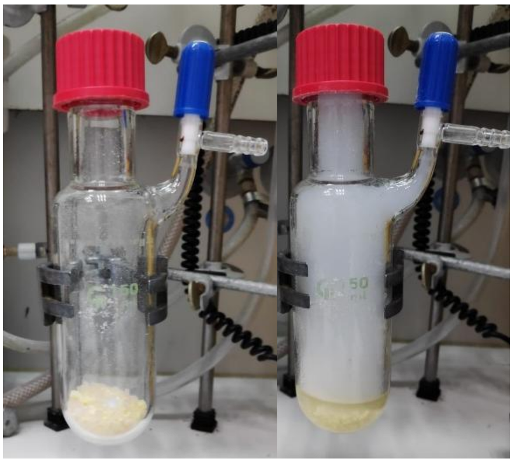

Step 2: Freshly distilled *tert*-butylamine (6.83 mL, 65.0 mmol, 1.3 eq) was quickly added to the reaction mixture using a syringe and the reaction vessel was sealed to trap the releasing fume inside.

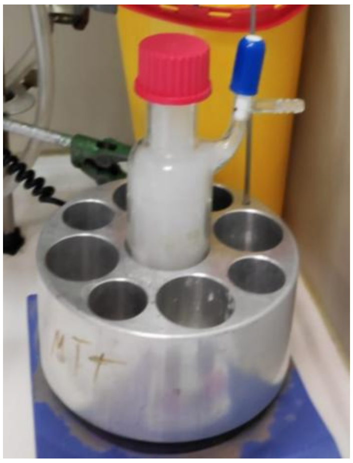

Step 3: The reaction vessel was placed in a preheated metal block or oil bath (140 °C) and was stirred on stirring rates around 1000 RPM for at least 1 hour. While stirring, the reaction mixture is becoming a viscous dark red to brown gel.

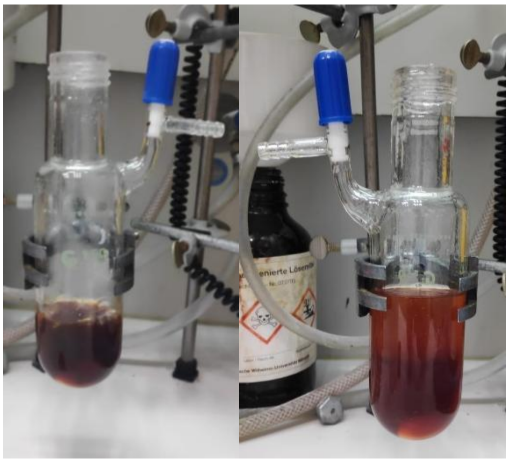

<u>Step 4</u>: The screw cap tube was taken of the heating source and CH_2Cl_2 was added carefully and portion-wise to the hot mixture to cool it down to approximately 40°C. This is done to avoid solidification of the reaction mixture which causes solvation issues. After cooling down to ambient temperature, water was added slowly and portion wise while gas releases from the mixture. In between the addition steps more CH_2Cl_2 was added to the reaction. After completion of the quenching process, additional portions of water (20 mL) and CH_2Cl_2 (20 mL) were added to solve remaining solids.

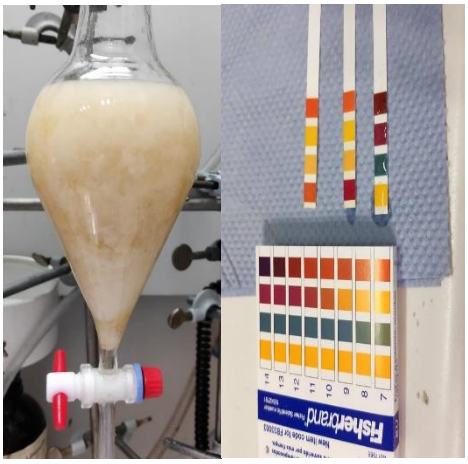

<u>Step 5</u>: The reaction mixture was added to a 1 or 2 liter separating funnel and water was added (250 mL). The mixture was acidified with HCl-solution (1.0 M) until pH < 1 was reached and washed with CH_2Cl_2 (3 x 250 ml). The remaining aqueous layer was basified using NaOH-solution (2.0 M) until pH 13 to 14 was reached. During this process, the amidine crashes out forming visible white clouds (compare picture). The layer was extracted with CH_2Cl_2 (3 x 500 ml) and the organic layer was dried over Na_2SO_4.

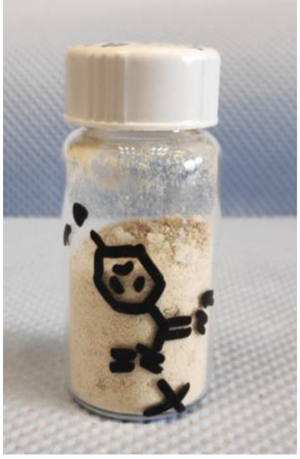

<u>Step 6</u>: The solvent was removed giving the pure product as an off-white solid (7.60 g, 36.8 mmol, 74%).

6.1.3 Synthesis of 2-((trifluoromethyl)thio)isoindoline-1,3-dione

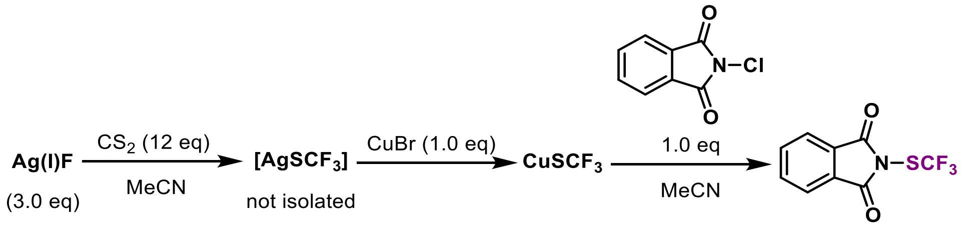

Following a modified literature procedure[19], acetonitrile (80 mL) was added to silver(I)fluoride (22.5 g, 180 mmol, 3.0 eq) and carbon disulfide (45 mL) in the dark. The mixture was stirred at 80 °C overnight and the remaining carbon disulfide was distilled off. Copper(I)bromide (8.60 g, 60.0 mmol, 1.0 eq) was added and the reaction mixture was stirred for an additional hour at 80 °C. After cooling down to room temperature, the suspension was filtered over celite and washed with acetonitrile (3 x 50 mL). The solvent was removed *in vacuo* to give $CuSCF_3$ (9.90 g, 60.0 mmol, quant.) as a brown solid which was used in the following step without further purification. To a solution of $CuSCF_3$ (9.90 g, 60.0 mmol, 1.0 eq) in acetonitrile (80.0 mL), *N*-chlorophthalimide (10.9 g, 60.0 mmol, 1.0 eq) was added and the solution was stirred at room temperature overnight. The resulting suspension was filtered over celite, washed with CH_2Cl_2 (3 x 50 mL) and the solvent was removed *in vacuo*. Purification by flash column chromatography (pentane:EtOAc, 4:1) gave the title compound **2** as a white powder (12.5 g, 50.6 mmol, 84%).

TLC (pentane:EtOAc, 4:1 v/v): $\mathbf{R_f}$ = 0.21; $\mathbf{^1H}$ **NMR** (300 MHz, $CDCl_3$) δ 7.94 (td, *J* = 5.3, 2.1 Hz, 2H), 7.81 (td, *J* = 5.3, 2.1 Hz, 2H); $\mathbf{^{13}C}$ **NMR** (75 MHz, $CDCl_3$) δ 165.9, 135.6, 131.6, 127.9 (q, *J* = 317.1 Hz), 124.9; $\mathbf{^{19}F}$ **NMR** (376 MHz, $CDCl_3$): δ –48.9[20].

6.2 Preparation of SCF_3-Amidines

6.2.1 Synthesis of SCF_3-Amidines 3a to 3g.

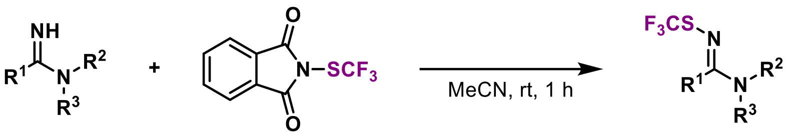

General Procedure B

Under an air atmosphere *N*-(trifluoromethylthio)phthalimide (1.0 eq) and the respective amidine (1.0 eq) were dissolved in acetonitrile (0.50 M) and the mixture was stirred at room temperature for 1 h. Liquid amidines were added after the acetonitrile. The solvent was removed *in vacuo* and purification by flash column chromatography (CH_2Cl_2/pentane, 1:1 v/v) gave the respective pure product.

***N*-(*tert*-butyl)-*N'*-((trifluoromethyl)thio)benzimidamide (3a)**

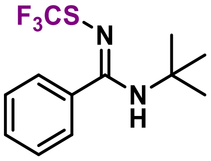

3a, 95%

Following **general procedure B**, *N*-(*tert*-butyl)benzimidamide (**1a**) (529 mg, 3.00 mmol, 1.0 eq) and *N*-(trifluoromethylthio)phthalimide (**2**) (742 mg, 3.00 mmol, 1.0 eq) were reacted to give the product **3a** as a yellow oil (808 mg, 2.85 mmol, 95%).

TLC (CH_2Cl_2): **R_f** = 0.66; **^{1}H NMR** (300 MHz, $CDCl_3$): δ 7.45 (dd, *J* = 5.0, 2.0 Hz, 3H), 7.37 – 7.30 (m, 2H), 4.73 – 4.51 (m, 1H), 1.46 (s, 9H); **^{13}C NMR** (75 MHz, $CDCl_3$): δ 160.2, 136.0 130.9 (q, *J* = 305.6 Hz), 130.3, 129.1, 126.8, 52.6, 28.9; **^{19}F NMR** (282 MHz, $CDCl_3$): δ -51.1; **HRMS (ESI)** (*m/z*): $[M + H]^+$ calcd. for $C_{12}H_{15}F_3N_2SH$ = 277.0981; found: 277.0981.

_N_-(_tert_-butyl)-4-methoxy-_N′_-((trifluoromethyl)thio)benzimidamide (3b)

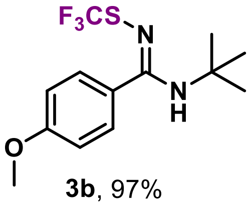

3b, 97%

Following **general procedure B**, *N*-(*tert*-butyl)-4-methoxybenzimidamide (**1b**) (5.16 g, 25.0 mmol, 1.0 eq) and *N*-(trifluoromethylthio)phthalimide (**2**) (6.18 g, 25.0 mmol, 1.0 eq) were reacted to give the product **3b** as an off-white solid (1.85 g, 24.2 mmol, 97%).

TLC (CH_2Cl_2): $\mathbf{R_f}$ = 0.67; **^{1}H NMR** (300 MHz, $CDCl_3$): δ 7.28 (dd, *J* = 9.2, 2.5 Hz, 2H), 7.00 – 6.86 (m, 2H), 4.57 (s, 1H), 3.84 (s, 3H), 1.44 (s, 9H); **^{13}C NMR** (75 MHz, $CDCl_3$): δ 160.8, 159.9, 131.0 (q, *J* = 305.8 Hz), 128.3, 128.3, 114.1, 55.4, 52.3, 28.8; **^{19}F NMR** (282 MHz, $CDCl_3$): δ −51.2; **HRMS (ESI)** (*m/z*): $[M + H]^+$ calcd. for $C_{13}H_{21}F_3N_2OSH$ = 307.1086; found: 307.1096.

_N_-(_tert_-butyl)-_N_-ethyl-_N′_-((trifluoromethyl)thio)benzimidamide (3c)

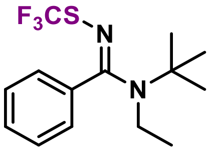

3d, 72%

Following **general procedure B**, *N*-(*tert*-butyl)-*N*-methylbenzimidamide (**1c**) (306 mg, 1.50 mmol, 1.0 eq) and *N*-(trifluoromethylthio)phthalimide (**2**) (371 mg, 1.00 mmol, 1.0 eq) were reacted to give the product **3c** as a yellow oil (333 mg, 1.09 mmol, 72%).

TLC (CH_2Cl_2): $\mathbf{R_f}$ = 0.68; **^{1}H NMR** (300 MHz, $CDCl_3$): δ 7.56 – 7.36 (m, 3H), 7.26 – 7.16 (m, 2H), 3.12 (q, *J* = 7.0 Hz, 2H), 1.53 (s, 9H), 0.95 (t, *J* = 7.0 Hz, 3H); **^{13}C NMR** (75 MHz, $CDCl_3$); δ 162.9, 136.8, 129.6, 129.2, 126.8, 57.4, 42.6, 29.2, 17.2 (S*C*F_3-carbon fully not visible); **^{19}F NMR** (282 MHz, $CDCl_3$): δ −50.3; **HRMS (ESI)** (*m/z*): $[M + H]^+$ calcd. for $C_{14}H_{19}F_3N_2SH$ = 305.1294; found: 305.1313.

(*N*-(tert-butyl)-*N′*-((trifluoromethyl)thio)isobutyrimidamide (3d)

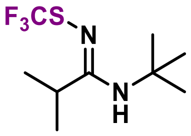

3c, 78%

Following **general procedure B**, *N*-(*tert*-butyl)isobutyrimidamide (**1d**) (156 mg, 1.00 mmol, 1.0 eq) and *N*-(trifluoromethylthio)phthalimide (**2**) (246 mg, 1.00 mmol, 1.0 eq) were reacted to give the product **3c** as a yellow oil (190 mg, 0.78 mmol, 78%).

TLC (CH_2Cl_2): **R_f** = 0.65; **^{1}H NMR** (300 MHz, $CDCl_3$): δ 4.33 (s, 1H), 2.74 (hept, *J* = 6.9 Hz, 1H), 1.34 (s, 9H), 1.10 (d, *J* = 6.9 Hz, 6H); **^{13}C NMR** (75 MHz, $CDCl_3$): δ 164.5, 129.2 (q, *J* = 305.5 Hz), 51.4, 32.9, 28.6, 19.3; **^{19}F NMR** (282 MHz, $CDCl_3$): δ −51.1; **HRMS (ESI)** (*m/z*): $[M + H]^+$ calcd. for $C_9H_{17}F_3N_2SH$ = 243.1137; found: 243.1136.

***N,N*-diethyl-*N′*-((trifluoromethyl)thio)benzimidamide (3e)**

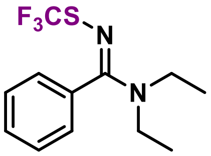

3e, 66%

Following **general procedure B**, *N,N*-diethylbenzimidamide (**1e**) (529 mg, 3.00 mmol, 1.0 eq) and *N*-(trifluoromethylthio)phthalimide (**2**) (742 mg, 3.00 mmol, 1.0 eq) were reacted to give the product **3e** as a yellow oil (546 mg, 1.98 mmol, 66%).

TLC (CH_2Cl_2): **R_f** = 0.67; **^{1}H NMR** (300 MHz, $CDCl_3$): δ 7.58 – 7.35 (m, 3H), 7.23 – 7.10 (m, 2H), 3.30 (s, 4H), 1.11 (s, 6H); **^{13}C NMR** (75 MHz, $CDCl_3$) δ 164.0, 134.7, 129.7, 130.6 (q, *J* = 306.3 Hz), 129.2, 126.8, 43.0, 13.6; **^{19}F NMR** (282 MHz, $CDCl_3$): δ −51.3; **HRMS (ESI)** (*m/z*): $[M + H]^+$ calcd. for $C_{13}H_{17}F_3N_2SH$ = 277.0981; found: 277.1002.

***N*-phenyl-*N′*-((trifluoromethyl)thio)benzimidamide (3f)**

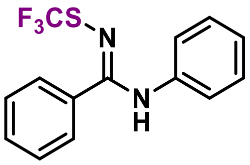

3f, 95%

Following **general procedure B**, *N*-phenylbenzimidamide (**1f**) (196 mg, 1.00 mmol, 1.0 eq) and *N*-(trifluoromethylthio)phthalimide (**2**) (246 mg, 1.00 mmol, 1.0 eq) were reacted to give the product **3f** as a white solid (282 mg, 0.95 mmol, 95%).

TLC (CH_2Cl_2): **R_f** = 0.63; **^{1}H NMR** (300 MHz, $CDCl_3$): δ 7.66 – 7.40 (m, 10H), 7.39 – 7.31 (m, 4H), 7.32 – 7.23 (m, 2H), 7.11 (q, J = 7.6 Hz, 2H), 6.86 (d, J = 7.8 Hz, 2H), 6.66 (s, 1H), 6.51 (s, 1H) (Two sets of signals could be explained by the appearance of rotamers); **^{13}C NMR** (75 MHz, $CDCl_3$): δ 156.6, 155.0, 138.5, 134.8, 134.6, 132.0, 131.1, 130.8, 129.5, 129.3, 129.1, 128.7, 128.6, 126.8, 124.6, 123.5, 122.4, 119.1 (S*C*F_3-carbon not visible) (Two sets of signals could be explained by the appearance of rotamers); **^{19}F NMR** (282 MHz, $CDCl_3$) δ = −50.2, −50.9 (Two sets of signals could be explained by the appearance of rotamers); **HRMS (ESI)** (*m/z*): $[M + H]^+$ calcd. for $C_{14}H_{11}F_3N_2SH$ = 297.0668; found: 297.0679.

N-ethyl-4-methoxy-*N*-methyl-*N'*-((trifluoromethyl)thio)benzimidamide (3g)

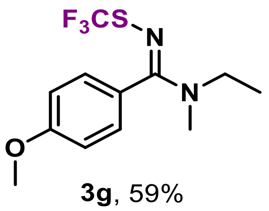

3g, 59%

Following **general procedure B**, *N*-ethyl-4-methoxy-*N*-methylbenzimidamide (**1g**) (961 mg, 5.00 mmol, 1.0 eq) and *N*-(trifluoromethylthio)phthalimide (**2**) (1.24 g, 5.00 mmol, 1.0 eq) were reacted to give the product **3g** as a white solid (865mg, 2.93 mmol, 59%).

TLC (CH_2Cl_2): **R_f** = 0.66; **^{1}H NMR** (400 MHz, $CDCl_3$): δ 7.14 (m, 2H), 6.98 (m, 2H), 3.84 (s, 3H), 3.20 (s, 2H), 2.93 (s, 3H), 1.05 (t, J = 7.1 Hz, 3H); **^{13}C NMR** (75 MHz, $CDCl_3$): δ 164.5, 160.6, 131.0 (q, J = 305.8 Hz), 128.5, 126.6, 114.6, 55.4, 46.1, 35.5, 13.0; **^{19}F NMR** (282 MHz, $CDCl_3$): δ −51.4; **HRMS (ESI)** (*m/z*): $[M + H]^+$ calcd. for $C_{12}H_{17}F_3N_2OSH$ = 293.0930; found: 293.0944.

6.2.2 Graphical Guide for the Large Scale Synthesis of of *N*-(*tert*-butyl)-4-methoxy-*N'*-((trifluoromethyl)thio)benzimidamide

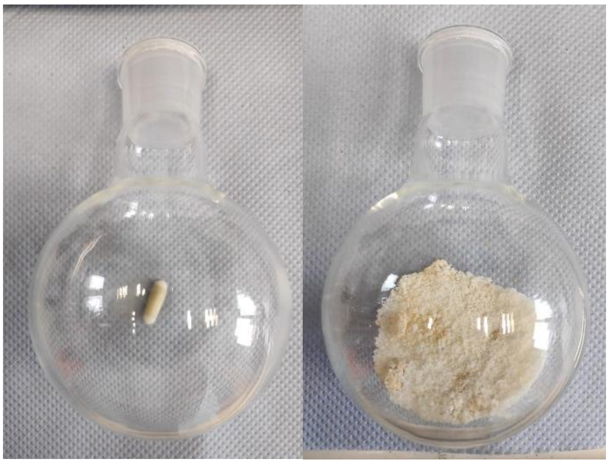

Step 1: A common round bottom flask was equipped with a stirring bar and *N*-(*tert*-butyl)-4-methoxybenz-imidamide (5.16 g, 25.0 mmol, 1.0 eq) as well as *N*-(trifluoromethylthio)phthalimide (6.18 g, 25.0 mmol, 1.0 eq) were added.

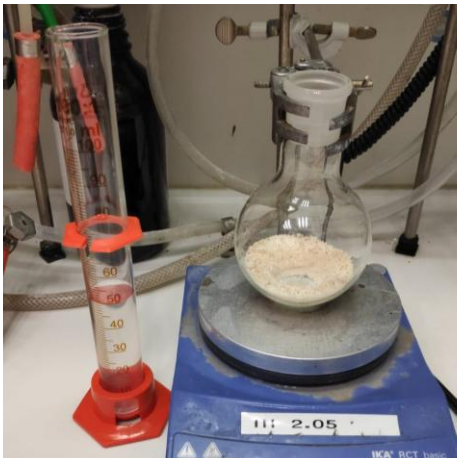

Step 2: Under moderate stirring (200 – 500 RPM), acetonitrile (50 mL), was added to the solid.

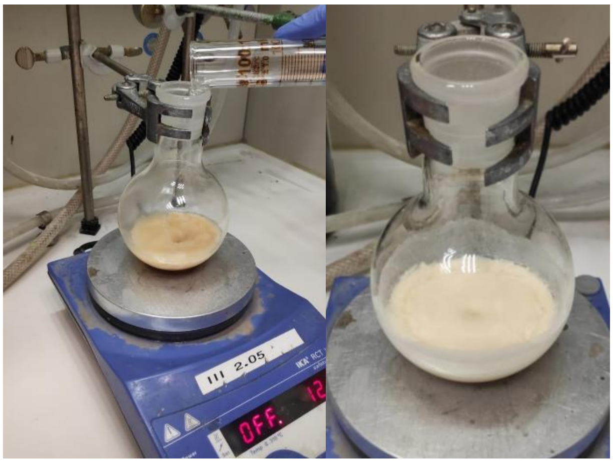

Step 3: The solids first dissolve upon addition of acetonitrile and after a few seconds phthalimide crashes out. The reaction mixture was stirred rigorously (1200 RPM) for 1 h.

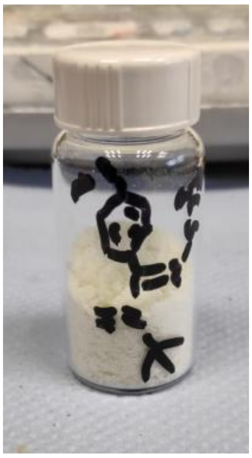

<u>Step 4</u>: The solvent was removed *in vacuo*, and the remaining dry mixture was purified via column chromatography (200 g silica gel; elution with pentane:EtOAc 4:1) to give the product as a pale yellow to white solid (7.42 g, 24.2 mmol, 97%).

6.3 Mechanistic Studies on SCF_3-Amidines

6.3.1 Computational Studies on Oxidation Potentials and Triplet Energies

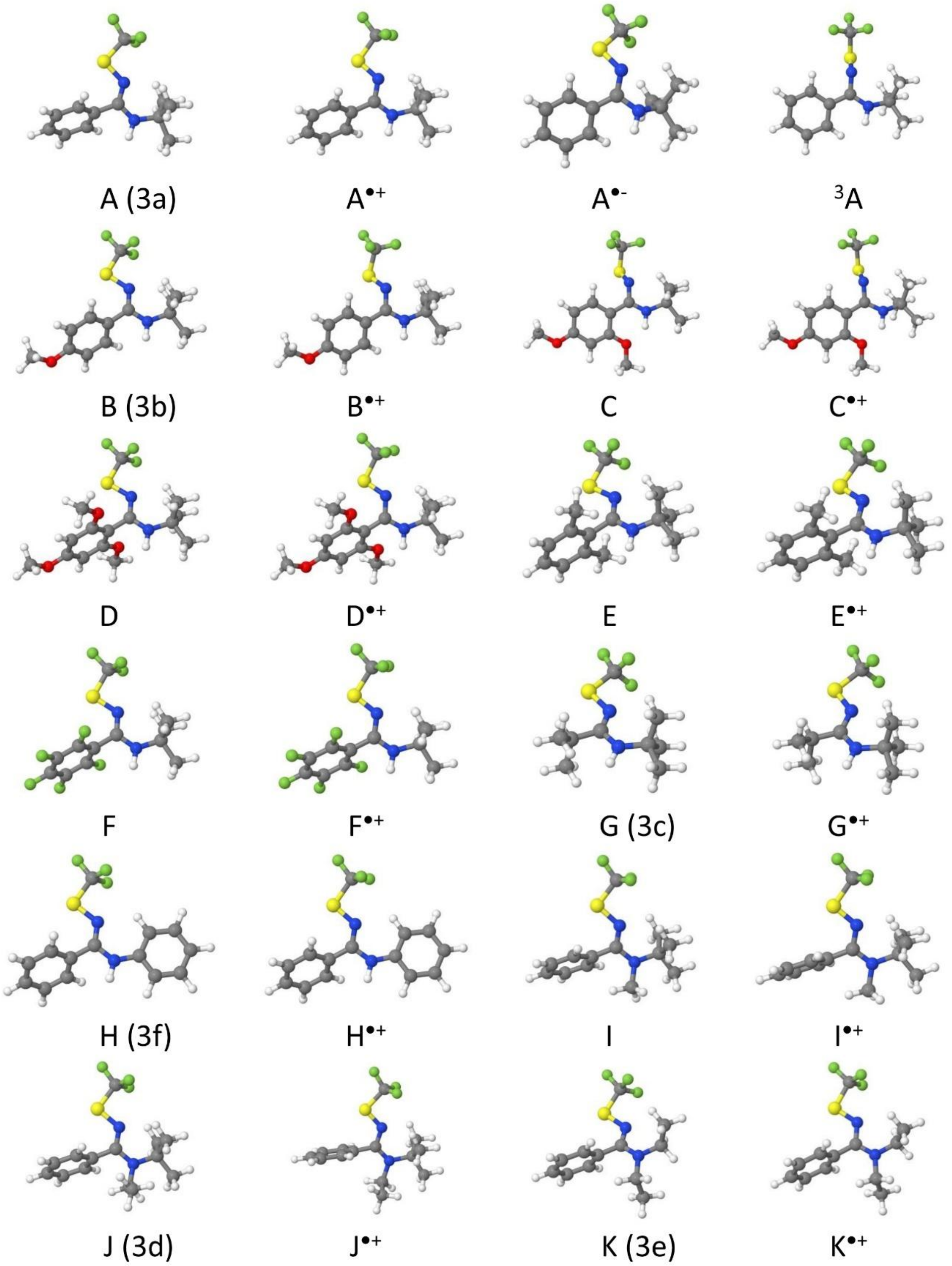

Figure S26: Optimized geometries of different SCF_3-amidines((CAM-B3LYP/TZVPP)/ CPCM (MeCN)).

Table S45: Determination of the Gibbs Free Energy (in Hartree) at 298 K (CAM-B3LYP/TZVPP)/CPCM(MeCN) for SCF_3-amidines as well as corresponding radical cation-, radical anion- and triplet structures.

	Electronic Energy	Thermal Corrections	ZPVE	Enthalpy Corrections	Entropy Corrections	Gibbs Free Energy
**A (3a)**	-1273.539061	0.018310	0.258764	0.000944	-0.063686	-1273.32473
**$A^{•+}$**	-1273.332321	0.017435	0.259353	0.000944	-0.062620	-1273.11721
**$A^{•-}$**	-1273.605475	0.018376	0.255518	0.000944	-0.064633	-1273.39527
**^{3}A**	-1273.450987	0.018634	0.256086	0.000944	-0.065376	-1273.2407
**B (3b)**	-1388.068960	0.020894	0.291434	0.000944	-0.069101	-1387.82479
**$B^{•+}$**	-1387.863942	0.020119	0.291941	0.000944	-0.068512	-1387.61945
**C**	-1502.595653	0.023606	0.324060	0.000944	-0.074912	-1502.32196
**$C^{•+}$**	-1502.393252	0.022700	0.324787	0.000944	-0.073990	-1502.11881
**D**	-1617.124878	0.025511	0.356424	0.000944	-0.078200	-1616.8202
**$D^{•+}$**	-1616.920571	0.025429	0.357229	0.000944	-0.078815	-1616.61578
**E**	-1352.152256	0.021864	0.313549	0.000944	-0.071046	-1351.88695
**$E^{•+}$**	-1351.942393	0.021108	0.313976	0.000944	-0.069931	-1351.6763
**F**	-1769.821418	0.023113	0.217763	0.000944	-0.074461	-1769.65406
**$F^{•+}$**	-1769.602733	0.021172	0.218502	0.000944	-0.070238	-1769.43235
**G (3c)**	-1160.448939	0.017295	0.261250	0.000944	-0.061122	-1160.23057
**$G^{•+}$**	-1160.243323	0.016334	0.261963	0.000944	-0.059725	-1160.02381
**H (3f)**	-1347.325855	0.016362	0.228650	0.000944	-0.060619	-1347.14052
**$H^{•+}$**	-1347.113436	0.016659	0.227023	0.000944	-0.062538	-1346.93135
**I**	-1312.818736	0.019425	0.287289	0.000944	-0.065718	-1312.5768
**$I^{•+}$**	-1312.615931	0.018868	0.287211	0.000944	-0.065766	-1312.37467
**J (3c)**	-1352.122246	0.020690	0.315971	0.000944	-0.068350	-1351.85299
**$J^{•+}$**	-1351.919405	0.019989	0.316148	0.000944	-0.067601	-1351.64993
**K (3e)**	-1273.525528	0.018451	0.259795	0.000944	-0.064851	-1273.31119
**$K^{•+}$**	-1273.324343	0.017708	0.259932	0.000944	-0.064417	-1273.11018

To determine the quenching mode of *N*-(*tert*-butyl)-*N'*-((trifluoromethyl)thio)benzimidamide (**A**), the energy of different conformers was evaluated and the most stabilized conformer was used for further calculations on redox potentials and the triplet energy. While the reduction potential (-2.5 V) is not in range of the photocatalyst [Ir(dF(CF_3)ppy)$_2$(dtbpy)][PF_6] (**PC1**), an

oxidation (1.2 V) seems to be feasible (Table S46). In addition, a triplet energy transfer would theoretically be possible based on the calculations, but was excluded experimentally (6. Redox-Neutral Radical Cyclization as Model Reaction). To find SCF_3-amidines with lower oxidation potentials, different candidates were evaluated *in silico* and synthesised if an oxidation by $[Ir(dF(CF_3)ppy)_2(dtbpy)][PF_6]$ (**PC1**) was likely to be possible (Table S47).

Table S46: Calculated redox potential and triplet energy of *N*-(*tert*-butyl)-*N'*-((trifluoromethyl)thio)benzimidamide (**A**) in comparison to redox potentials of **PC1**.

	N-(*t*Bu)-*N'*-((SCF_3) benzimidamide (**A**)		$[Ir(dF(CF_3)ppy)_2(dtbpy)][PF_6]$ (**PC1**)
E^{calc} ($\mathbf{A^0/A^{\bullet+}}$)	1.2 V	$E^{1/2}$ (**PC1** */**PC1** $^+$)	−0.9 V[18]
E^{calc} ($\mathbf{A^{\bullet-}/A^0}$)	−2.5 V	$E^{1/2}$ (**PC1** $^-$/**PC1** *)	1.2 V[18]
ΔE(^{1}**A**/^{3}**A**)	52.7 kcal/mol	ΔE(^{1} **PC1**/^{3} **PC1**)	61.8 kcal/mol[18]

Table S47: Calculated redox potentials of selected SCF_3-Amidines (CAM-B3LYP/TZVPP)/CPCM(MeCN).

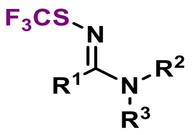

$X^0/X^{\bullet+}$	R^1	R^2	R^3	E^{calc} ($X^0/X^{\bullet+}$)
**A (3a)**	Ph	*t*Bu	H	1.22 V
**B (3b)**	4-MeOPh	*t*Bu	H	1.17 V
**C**	2,4-$(MeO)_2$Ph	*t*Bu	H	1.11 V
**D**	2,4,6-$(MeO)_3$Ph	*t*Bu	H	1.14 V
**E**	2,6-$(Me)_2$Ph	*t*Bu	H	1.31 V
**F**	C_6F_5	*t*Bu	H	1.61 V
**G (3d)**	*i*Pr	*t*Bu	H	1.20 V
**H (3f)**	Ph	Ph	H	1.27 V
**I**	Ph	*t*Bu	Me	1.08 V
**J (3c)**	Ph	*t*Bu	Et	1.10 V
**K (3e)**	Ph	Et	Et	1.05 V

6.3.2 Cyclic Voltammetry Studies on Oxidation Potentials and Reversibility

To confirm the calculated redox potentials and the irreversibility of the proposed decomposition process, cyclic voltammetry was carried out for three SCF_3-amidines (**3a**/**3b**/**3c**) (Figure S28 and Figure S29). While SCF_3-amidines **3a** and **3b** show a fully irreversible oxidation curve, even at high scan rates of 10 V/s amidine **3c** can partially be reduced back (Figure S28). Due to this reversibility, a lower reactivity in the respective (photo-) redox processes is expected since the decomposition and therefore the radical release is slowed down and therefore a back reduction by the reduced species of the photocatalyst becomes feasible. As a consequence of the irreversibility and the therefore missing corresponding reduction-peak, the half peak potential $E^{1/2}$, could not be calculated from the mean of both peaks[21]. To approximate $E^{1/2}$ the potential at half height of the oxidation curve was read out (Figure S29). To compare the results with the calculated potentials (Table S48), the potential was recalculated against the saturated calomel electrode (SCE) ($Fc^0/Fc^+ = 0.40$ V against SCE) (Table S48)[22].

$$E^{SCE} = E^{measured(Fc)} + 0.40\ V$$

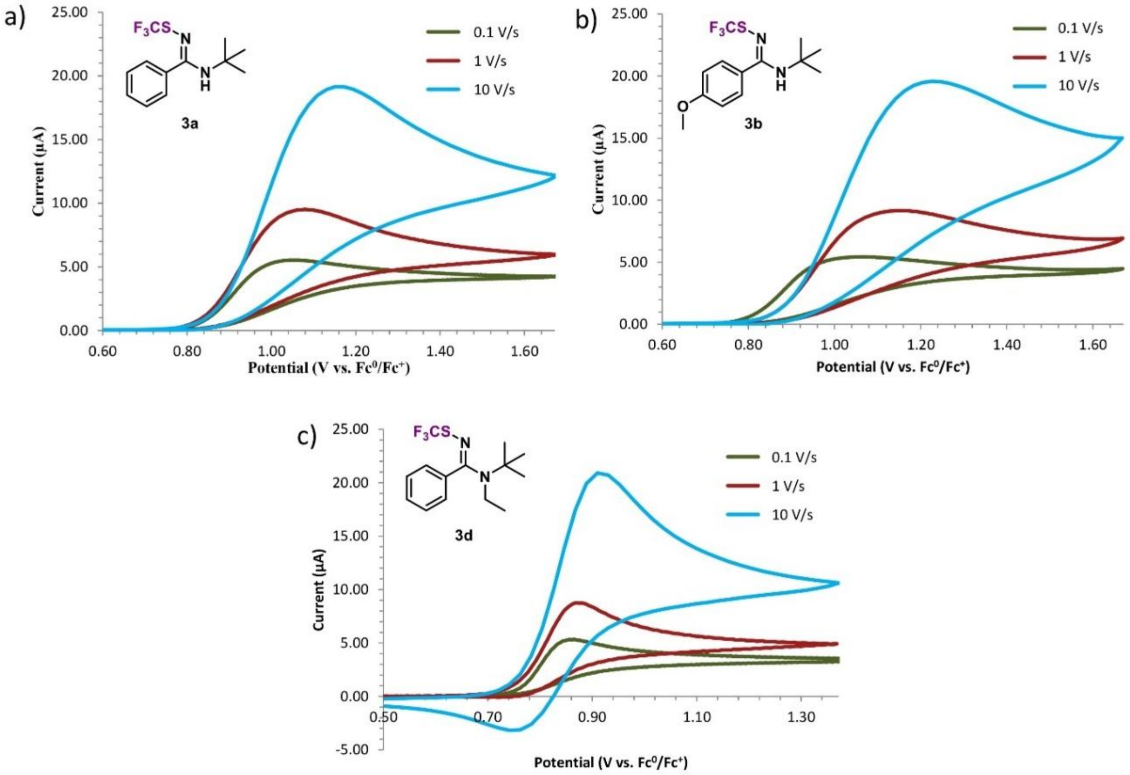

Figure S28: Voltammograms of three different SCF_3-Amidines (**3a**/**3b**/**3c**) in acetonitrile recorded at $\upsilon = 0.1$ V/s (green), $\upsilon = 1$V/s (red), $\upsilon = 10$ V/s (blue); referenced to the ferrocene/ferrocenium ion pair.

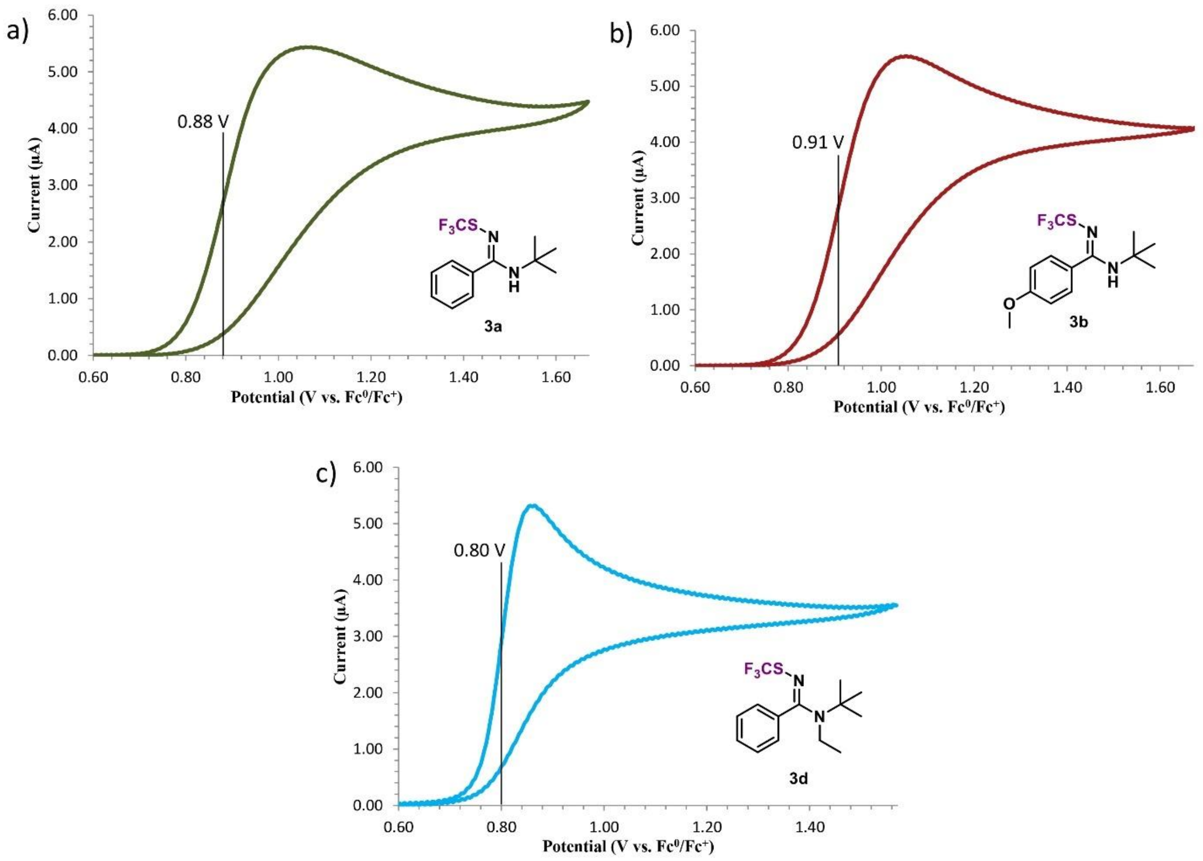

Figure S29: Voltammograms of three different SCF_3-amidines (**3a** (green); **3b** (red); **3c** (blue)) in acetonitrile recorded at $\upsilon = 0.1$ V/s; referenced to the ferrocene/ferrocenium ion pair. To approximate the oxidation potential, the potential at half height of the oxidation curve was read out (black line).

Table S48: Measured half peak potentials of the SCF_3-amidines **3a**, **3b**, **3c** against the ferrocene/ferrocenium ion pair and against the saturated calomel electrode.

	$E^{1/2}$ against Ferrocene	$E^{1/2}$ against SCE	E^{calc}
$\mathbf{A^0/A^{\bullet+}}$	0.88 V	1.3 V	1.2 V
$\mathbf{B^0/B^{\bullet+}}$	0.91 V	1.3 V	1.2 V
$\mathbf{J^0/J^{\bullet+}}$	0.80 V	1.2 V	1.1V

7. Redox-Neutral Radical Cyclization as Model Reaction

7.1 Preparation of Starting Materials

7.1.1 Synthesis of Catalysts

The photocatalysts [Ir(dFCF$_3$ppy)$_2$(dtbpy)][PF$_6$] (**PC1**)[23], [Ir(ppy)$_2$(dtbpy)][PF$_6$][24], *fac*-Ir(ppy)$_3$ (**PC2**)[28], [Ru(bpy)$_3$](PF$_6$)$_2$ (**PC3**)[29,30], 4CzIPN[36] were prepared following literature procedures.

7.1.2 Synthesis of Alkene 4a and Alkyne 4b.

5-Hexenal

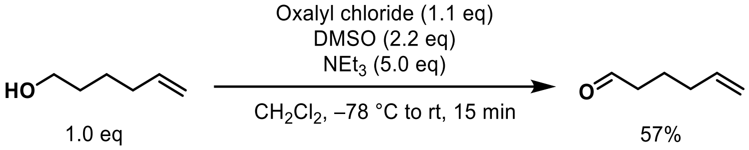

Following a modified literature procedure[37], oxalyl chloride (2.90 ml, 33.0 mmol, 1.1 eq) was added to CH_2Cl_2 (75 mL) at −78 °C followed by the addition of DMSO (4.70 mL, 66.0 mmol, 2.2 eq) in CH_2Cl_2 (15.0 mL). The reaction mixture was stirred at −78 °C for 5 min, hex-5-en-1-ol (3.6 mL, 30 mmol, 1.0 eq) in CH_2Cl_2 (30 mL) was added dropwise and the reaction mixture was stirred for additional 15 min. NEt_3 (21 mL, 150 mmol, 5.0 eq) was added and the resulting suspension was stirred at −78°C for 5 min. After warming up to room temperature, water (150 mL) was added the reaction mixture was extracted with CH_2Cl_2 (3 x 50 mL) and washed with brine (50 mL). The solvent was removed *in vacuo* and purification by flash column chromatography (pentane:EtOAc, 9:1 v/v) gave the title compound **82** as a colourless oil (1.70 g, 17.3 mmol, 57%). This compound was used directly in the following step.

Methyl octa-2,7-dienoate (4a)

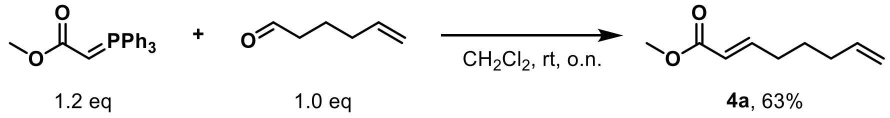

Following a modified literature procedure[37], methyl (triphenylphosphoranylidene)acetate (7.22 g, 21.6 mmol, 1.2 eq) was added to a solution of 5-hexenal (1.77 g 18.0 mmol, 1.0 eq) in CH_2Cl_2 (40 mL) and stirred at room temperature overnight. The solvent was removed *in vacuo* and purification by flash column chromatography (pentane:EtOAc, 99:1 v/v) gave the title compound **4a** as a colourless oil (95:5 d.r., 1.76 g, 11.4 mmol, 63%).

TLC (pentane:EtOAc, 19:1 v/v): **R_f** = 0.57; **^{1}H NMR** (300MHz, $CDCl_3$): δ 6.96 (dt, J = 15.6, 7.0 Hz, 1H), 5.94 – 5.67 (m, 2H), 5.11 – 4.91 (m, 2H), 3.72 (s, 3H), 2.26 – 2.16 (m, 2H), 2.12 – 2.02 (m, 2H), 1.66 – 1.51 (m, 2H) (major diasteriomer); **^{13}C NMR** (75 MHz, $CDCl_3$): δ 167.3, 149.4, 138.1, 121.2, 115.3, 51.6, 33.2, 31.7, 27.3 (major diasteriomer)[38].

Hex-5-ynal

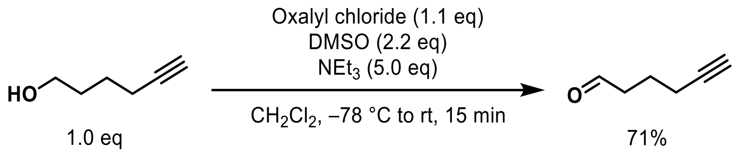

Following a modified literature procedure[39], oxalyl chloride (1.00 ml, 11.0 mmol, 1.1 eq) was added to CH_2Cl_2 (25 mL) at −78 °C followed by the addition of DMSO (1.60 mL, 22.0 mmol, 2.2 eq) in CH_2Cl_2 (5.0 mL). The reaction mixture was stirred at −78 °C for 5 min, hex-5-yn-1-ol (1.10 mL, 10.0 mmol, 1.0 eq) in CH_2Cl_2 (10 mL) was added dropwise and the reaction mixture was stirred for additional 15 min. NEt_3 (7.00 mL, 50.2 mmol, 5.0 eq) was added and the resulting suspension was stirred for 5 min. After heating up to room temperature, water (50 mL) was added, the reaction mixture was extracted with CH_2Cl_2 (3 x 20 mL) and washed with brine (20 mL). The solvent was removed *in vacuo* and purification by flash column chromatography (pentane:EtOAc, 9:1 v/v) gave the title compound **85** as a colourless oil (0.70 g, 7.1 mmol, 71%). This compound was used directly in the following step.

Methyl oct-2-en-7-ynoate (4b)

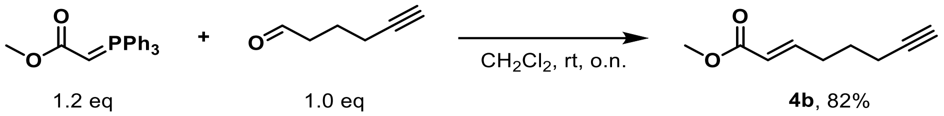

Following a modified literature procedure[39], methyl (triphenylphosphoranylidene)acetate (1.84 g, 5.50 mmol, 1.1 eq) was added to a solution of 5-hexenal (491 mg, 5.00 mmol, 1.0 eq) in CH_2Cl_2 (20 mL) and stirred at room temperature overnight. The solvent was removed *in vacuo* and purification by flash column chromatography (pentane:EtOAc, 9:1 v/v) gave the title compound **4b** as a colourless oil (619 mg, 4.10 mmol, 82%).

TLC (pentane:EtOAc, 4:1 v/v): **R_f** = 0.52; **^{1}H NMR** (300 MHz, $CDCl_3$): δ 6.93 (dt, *J* = 15.7, 7.0 Hz, 1H), 5.85 (dt, *J* = 15.6, 1.6 Hz, 1H), 3.71 (s, 3H), 2.38 – 2.29 (m, 2H), 2.21 (td, *J* = 7.0, 2.7 Hz, 2H) (major diastereomer), 1.96 (t, *J* = 2.6 Hz, 1H), 1.72 – 1.63 (m, 2H); **^{13}C NMR** (75 MHz, $CDCl_3$): δ 167.1, 148.3, 121.8, 83.6, 69.2, 51.6, 31.1, 26.8, 18.0 (major diastereomer)[39].

7.2 Optimization of the Model Reaction

7.2.1 General Optimization

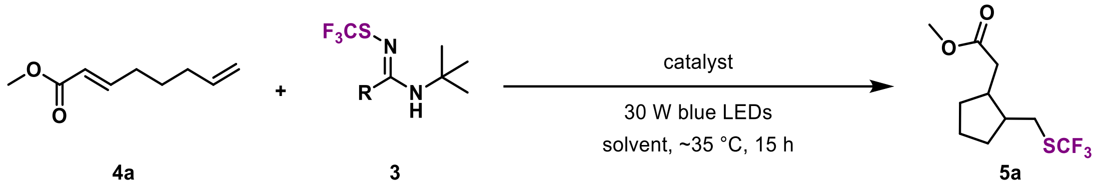

Unless otherwise noted, *N*-(*tert*-butyl)-*N*'-((trifluoromethyl)thio) benzimidamide (**3a**) (55.3 mg, 0.20 mmol, 2.0 eq) and [Ir(dFCF$_3$ppy)$_2$(dtbpy)][PF$_6$] (**PC1**) (2.8 mg, 0.0025 mmol, 2.5 mol%), were weighed into a Schlenk tube containing a magnetic stirring bar. The tube was evacuated and backfilled with argon three times. Toluene (1.0 mL) and methyl-octa-2,7-dienoate (**4a**) (17.0 µL, 0.10 mmol, 1.0 eq) were added and the resulting solution was degassed using three freeze-pump-thaw cycles. The reaction was stirred under irradiation with 450 nm LEDs for 16 h at room temperature. A solution of 1,3,5-trimethoxybenzene (100 µL, 0.10 mmol, 1.0 eq, 1 M in EtOAc) was added to the reaction mixture as an internal standard and an aliquot (30.0 µL) of the crude reaction mixture was filtered over a plug of silica (elution with EtOAc). The yield was determined via calibrated GC-FID.

Table S49: Optimization with respect to the solvent.

Entry	Solvent	Yield
**1**	MeCN	25%
**2**	DMF	28%
**3**	MeOH	29%
**4**	CH_2Cl_2	45%
**5**	**toluene**	**48%**
**6**	DMSO	36%
**7**	EtOAc	16%
**8**	acetone	20%
**9**	THF	6%
**10**	$PhCF_3$	19%
**11**	PhCl	36%
**12**	1,4-dioxan	38%
**13**	DCE	37%
**14**[a]	benzene	42%
**15**[a]	chloroform	18%

Standard conditions: **4a** (1.0 eq), **3a** (1.2 eq), [Ir(dFCF$_3$ppy)$_2$(dtbpy)][PF$_6$] (2.5 mol%), solvent (0.1 M), 16 h, ~35 °C; a) 2.0 eq of **3a** used.

Table S50: Optimization with respect to the catalyst.

Entry	Catalyst	Yield
**1**	[Ir(ppy)$_3$] (**PC2**)	Spuren
**2**	**[Ir(dF(CF$_3$)ppy)$_2$(dtbpy)][PF$_6$] (PC1)**	**48%**
**3**	[Ru(bpz)$_3$][PF$_6$]$_2$	n.r.
**4**[a]	[Ru(bpz)$_3$][PF$_6$]$_2$ (**PC3**)	n.r.
**5**	[Ir(dtbpy)(ppy)$_2$][PF$_6$]	n.r.
**6**[b]	$MesAcr^+BF4^-$	n.r.
**7**	[Ru(bpy)][PF$_6$]$_2$	n.r.
**8**	[Ir(dFppy)$_3$]	18%
**9**[b]	$TPP^+BF_4^-$	n.r.
**10**[b]	eosin Y	n.r.
**11**[c]	alloxazin	5%
**12**[c]	4-CzIPN	33%

Standard conditions: **4a** (1.0 eq), **3a** (1.2 eq), catalyst (2.5 mol%), toluene (0.1 M), 450 nm LED, 16 h, ~35 °C; a) CH_2Cl_2 as solvent; b) catalyst (10 mol%); c) catalyst (5.0 mol%).

Table S51: Optimization with respect to the reaction concentration.

Entry	Concentration (mol/L)	Yield
**1**	0.025	45%
**2**	0.050	37%
**3**	**0.10**	**49%**
**4**	0.20	45%
**5**	0.50	41%

Standard conditions: **4a** (1.0 eq), **3a** (1.2 eq), [Ir(dFCF$_3$ppy)$_2$(dtbpy)][PF$_6$] (**PC1**) (2.5 mol%), toluene, 450 nm LED, 16 h, ~35 °C.

Table S52: Optimization with respect to the stoichiometry.

Entry	Eq of 4a	Eq of 3a	Yield
**1**	1.0	1.2	48%
**2**	2.0	1.0	49%
**3**	1.0	1.5	54%
**4**	**1.0**	**2.0**	**62%**
**5**	1.0	3.0[a]	60%
**6**	1.0	5.0[a]	55%

Standard conditions: [Ir(dFCF$_3$ppy)$_2$(dtbpy)][PF$_6$] (**PC1**) (2.5 mol%), toluene (0.1 M), 450 nm LED, 16 h, ~35 °C; a) **3b** instead of **3a**.

Table S53: Optimization with respect to the catalyst loading.

Entry	Catalyst Loading (mol%)	Yield
**1**	1	36%
**2**	**2.5**	**62%**
**3**	5	43%

Conditions: **4a** (1.0 eq.), **3a** (2.0 eq.), [Ir(dFCF$_3$ppy)$_2$(dtbpy)][PF$_6$] (**PC1**), toluene (0.1 M), 450 nm LED, 16 h, ~35 °C.

Table S54: Optimization with respect to the SCF$_3$-reagent.

Entry	SCF$_3$-Reagent	Yield
**1**	3a	62%
**2**	**3b**	**65% (64%)**[a]
**3**	3c	8%
**4**	3d	60%
**5**	3e	23%
**6**	3f	trace

Standard conditions: **4a** (1.0 eq), **3** (2.0 eq), [Ir(dFCF$_3$ppy)$_2$(dtbpy)][PF$_6$] (**PC1**) (2.5 mol%), 450 nm LED, toluene (0.1 M), 16 h, ~35 °C; a) isolated yield.

Table S55: Optimization with respect to the additive.

Entry	Additive	Equivalents	Yield
**1**	Cs_2CO_3	0.5	19%
**2**	TFA	0.5	34%
**3**	benzoic acid	1.0	33%
**4**	pTsOH	1.0	22%
**5**	$(PhO)_2P(O)OH$	1.0	23%
**6**[a]	Cs_2CO_3	0.5	46%
**7**[a]	TFA	0.5	13%

Standard conditions: **4a** (1.0 eq), **3b** (2.0 eq), $[Ir(dFCF_3ppy)_2(dtbpy)][PF_6]$ (**PC1**) (2.5 mol%), toluene (0.1 M), 450 nm LED, 16 h, ~35 °C; a) CH_2Cl_2 as solvent.

Table S56: Control reactions.

Entry	Control	Yield
**1**	under air atmosphere	39%
**2**	no degassing	63%
**3**	no light	no reaction
**4**	no catalyst	no reaction
**5**	1.0 eq TEMPO	traces
**6**	2.5 eq TEMPO	no reaction
**7**[a]	365 nm LED	traces

Standard conditions: **4a** (1.0 eq), **3b** (2.0 eq), $[Ir(dFCF_3ppy)_2(dtbpy)][PF_6]$ (**PC1**) (2.5 mol%), toluene (0.1 M), 450 nm LED, 16 h, ~35 °C; a) no catalyst.

7.2.2 Determination of a Reaction Time Profile

To investigate the reaction time profile of the model reaction, experiments were set up (0.3 mmol scale) using the optimized reaction conditions. *N*-(*tert*-butyl)-4-methoxy-*N'*-((trifluoromethyl)thio) benzimidamide (**3a**) (184 mg, 0.60 mmol, 2.0 eq), 1,3,5-trimethoxybenzene (50.5 mg, 0.30 mmol, 1.0 eq) as internal standard and $[Ir(dFCF_3ppy)_2(dtbpy)][PF_6]$ (**PC1**) (8.4 mg, 0.0075 mmol, 2.5 mol%), were weighed into a Schlenk tube containing a magnetic stirring bar. The tube was evacuated and backfilled with argon three times. Toluene (3.0 mL) and methyl-octa-2,7-dienoate (**4a**) (51.0 µL, 0.30 mmol, 1.0 eq) were added and the resulting solution was degassed using three freeze-pump-thaw cycles. The reaction was stirred under irradiation with 450 nm LEDs. After addition of all reaction components the flask was kept attached to the Schlenk line. The irradiation was started and an aliquot (30.0 µL) of the reaction mixture was taken in a stream of argon at different times (0, 5, 10, 20, 30, 60, 120, 240, 480, 1380 min) using an argon purged air displacement pipette. The aliquot was filtered over a plug of silica (elution with EtOAc) and the yield at the corresponding time was determined via calibrated GC-FID (Figure S30).

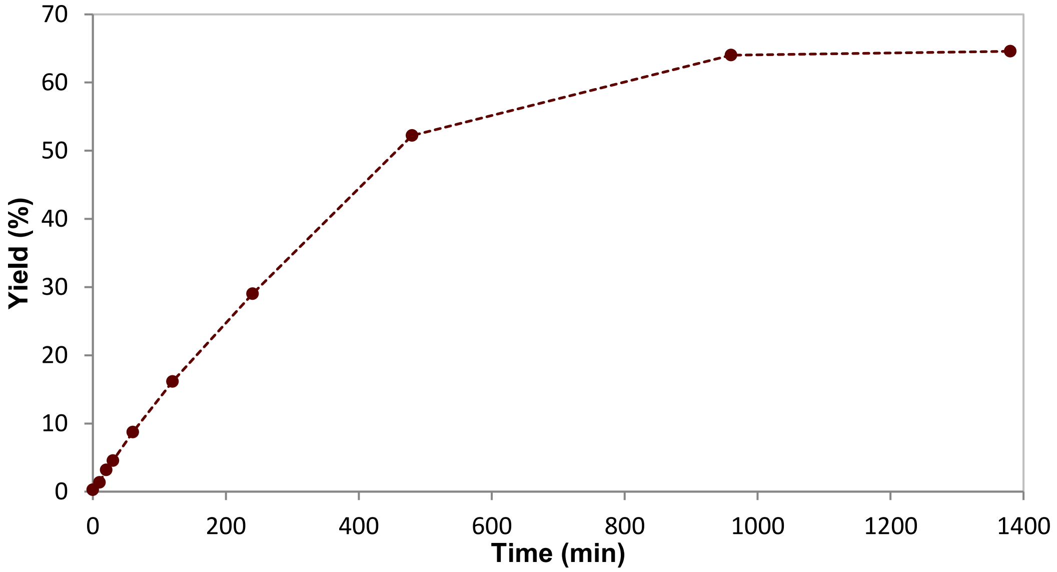

Figure S30: Reaction time profile of the model reaction.

In this study, no induction period could be detected, further verifying $[Ir^{III}(dF(CF_3)ppy)_2(dtbpy)]^{*+}$ as active species at the beginning of the catalytic cycle. Again this result underlines the proposed mechanism, wherein the SCF_3-amidine gets oxidized by the excited photocatalyst $[Ir^{III}(dF(CF_3)ppy)_2(dtbpy)]^{*+}$ followed by a deprotonation and direct decomposition of the oxidized SCF_3-amidine species ($\mathbf{A}^{\bullet+}$).

7.3 Scope of the Redox-neutral Radical Cyclization

General Procedure C

Unless otherwise noted, *N*-(*tert*-butyl)-4-methoxy-*N'*-((trifluoromethyl)thio) benzimidamide (**3a**) (184 mg, 0.60 mmol, 2.0 eq) and [Ir(dFCF$_3$ppy)$_2$(dtbpy)][PF$_6$] (**PC1**) (8.4 mg, 0.0075 mmol, 2.5 mol%), were weighed into a Schlenk tube containing a magnetic stirring bar. The tube was evacuated and backfilled with argon three times. Toluene (3.0 mL) and the corresponding alkene or alkyne (**4**) (0.30 mmol, 1.0 eq) were added and the resulting solution was degassed using three freeze-pump-thaw cycles. The reaction was stirred under irradiation with 450 nm LEDs for 24 h at room temperature. The solvent was removed *in vacuo* and the crude product was purified by flash column chromatography to obtain the pure products.

Methyl 2-(2-(((trifluoromethyl)thio)methyl)cyclopentyl)acetate (5a)

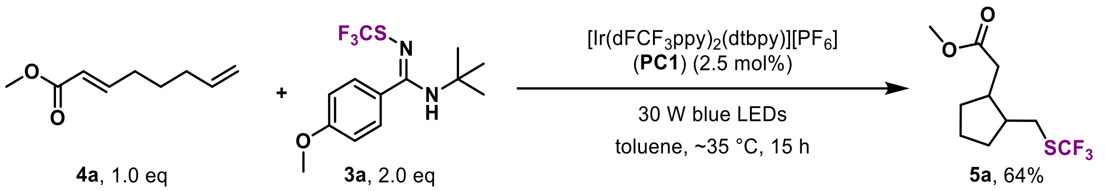

Following **general procedure C**, using methyl-octa-2,7-dienoate (**4a**) (51.0 µL, 0.30 mmol, 1.0 eq), the product **5a** (mixture of diastereomers, 70:30 d.r., 49.4 mg, 0.19 mmol, 64%) was obtained as a colourless oil after flash column chromatography (pentane:EtOAc, 49:1 to 19:1 v/v). 4-Methoxybenzonitrile (**6b**) (39.3 mg, 0.29 mmol, 98%) was isolated as side product.

TLC (pentane:EtOAc, 9:1 v/v): **R$_f$** = 0.57; **^{1}H NMR** (599 MHz, CDCl$_3$): δ 3.68 (s, 2H), 2.95 (dd, *J* = 12.2, 6.2 Hz, 1H), 2.69 (dd, *J* = 12.3, 9.7 Hz, 1H), 2.55 – 2.42 (m, 1H), 2.37 (dd, *J* = 15.0, 6.4 Hz, 1H), 2.31 – 2.14 (m, 2H), 1.85 (ddddd, *J* = 12.9, 10.7, 9.4, 7.2, 5.5 Hz, 2H), 1.78 – 1.66 (m, 1H), 1.66 – 1.58 (m, 2H), 1.52 – 1.42 (m, 1H), 1.38 (ddt, *J* = 13.1, 9.3, 6.8 Hz, 1H) (signals of the major diastereomer reported); **^{13}C NMR** (75 MHz, CDCl$_3$): δ 173.4, 131.3 (q, *J* = 305.7 Hz), 51.8, 41.7, 39.1, 34.6, 30.9, 30.8, 30.1, 22.3 (signals of the major diastereomer reported); **^{19}F NMR** (282 MHz, CDCl$_3$): δ = −41.4; **HRMS (ESI)** (*m/z*): [M + Na]$^+$ calcd. for $C_{10}H_{15}F_3O_2SNa$ = 279.0637; found: 279.0642.

Methyl 2-(2-(((trifluoromethyl)thio)methylene)cyclopentyl)acetate (5b)

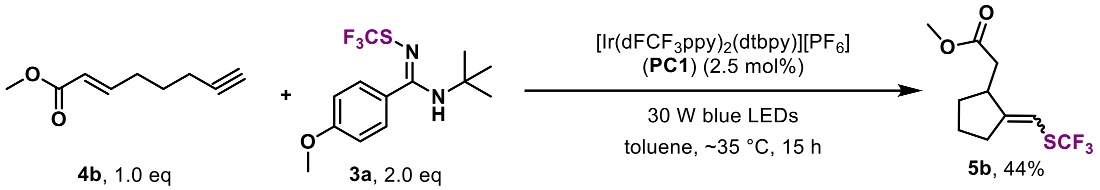

4b, 1.0 eq **3a**, 2.0 eq **5b**, 44%

Following **general procedure C**, using methyl oct-2-en-7-ynoate (**4b**) (50.6 µL, 0.30 mmol, 1.0 eq), the product **5b** (mixture of diastereomers, 80:20 d.r., 33.7 mg, 0.13 mmol, 44%) was obtained as a colourless oil after flash column chromatography (pentane:EtOAc, 49:1 v/v).

TLC (pentane:EtOAc, 9:1 v/v): $\mathbf{R_f}$ = 0.71; ^{1}**H NMR** (300 MHz, $CDCl_3$): δ 5.87 (q, *J* = 2.4 Hz, 1H), 3.69 (s, 3H), 2.95 (m, 1H), 2.58 (dd, *J* = 15.5, 5.9 Hz, 1H), , 2.35 (dd, *J* = 15.5, 8.5 Hz, 2H), 2.04 (td, *J* = 12.1, 6.8 Hz, 1H), 1.82 (m, 1H), 1.67 (m, 1H), 1.44 (m, 1H), 0.86 (m, 1H) (signals of the major diastereomer reported); ^{13}**C NMR** (75 MHz, $CDCl_3$-d) δ = 172.8, 159.8, 103.2, 51.8, 42.2, 38.4, 33.2, 31.7, 23.7 (S*C*F_3-carbon not visible) (Signals of the major diastereomer reported); ^{19}**F NMR** (282 MHz, $CDCl_3$): δ −42.7; **HRMS (ESI)** (*m/z*): $[M + Na]^+$ calcd. for $C_{10}H_{13}F_3O_2SNa$ = 277.0481; found: 277.0481.

7.4 Mechanistic Investigations on the Redox-neutral Radical Cyclization

7.4.1 Stern-Volmer Quenching Studies

To confirm the computationally supported mechanism of the model reaction, Stern-Volmer quenching experiments were carried out using the SCF_3-amidine **3a** and methyl-octa-2,7-dienoate **4** as substrates, and [Ir(dF(CF_3)ppy)$_2$(dtbpy)][PF_6] (**PC1**) as photocatalyst. Stock solutions of the photocatalyst [Ir(dF(CF_3)ppy)$_2$(dtbpy)][PF_6] (**PC1**) (0.1 mM) and the substrates (**3a**, **4**) (25 mM) were prepared in MeCN. To ensure good reproducibility and low errors during quenching, the fluorescence of the photocatalysts was probed multiple times until reproducible intensities could be achieved. In doing so, 100 µL of the catalyst stock solution was pipetted into a cuvette and diluted with 900 µL of MeCN. For Stern-Volmer quenching studies, 100 µL of the catalyst and 100 to 500 µL of the substrate stock solutions (**3a**, **4**) were pipetted into a cuvette and diluted with MeCN to a total volume of 1 mL. While the SCF_3-amidine **3a** shows significant quenching (Stern-Volmer constant of K_{sv} = 12.7 dm^3/mol), the substrate **4** shows no noticeable interaction (Figure S31).

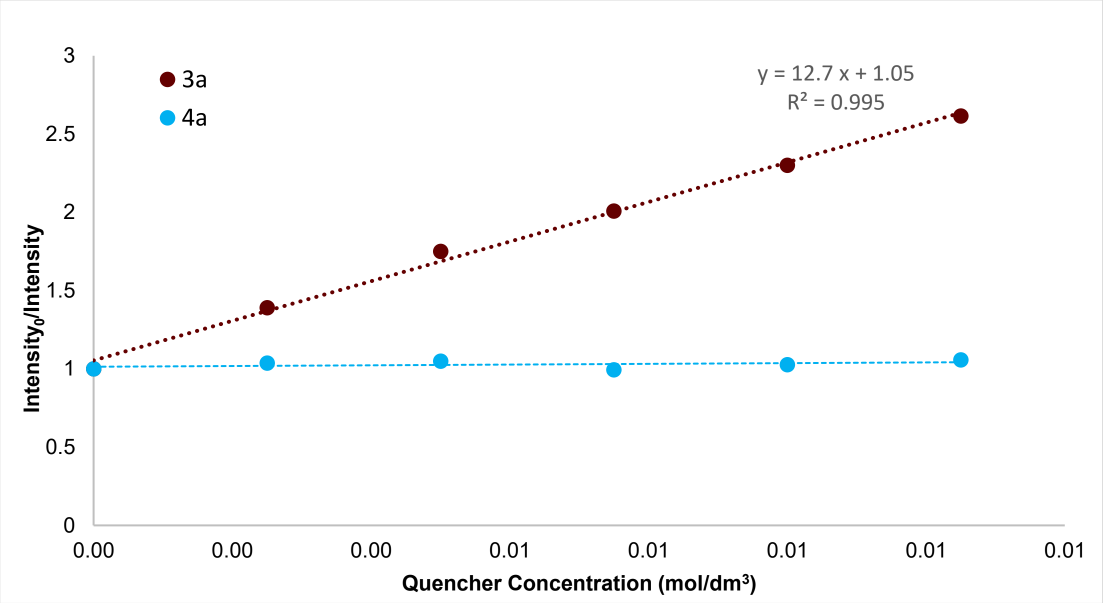

Figure S31: Stern-Volmer quenching studies using [Ir(dF(CF_3)ppy)$_2$(dtbpy)][PF_6] (**PC1**) (0.01 mM) as photocatalyst and SCF_3-amidine **3a** (red) or methyl-octa-2,7-dienoate **4a** (blue) (2.5 mM) as (potential) quencher.

7.4.2 Computational Investigation of the Reaction Pathways

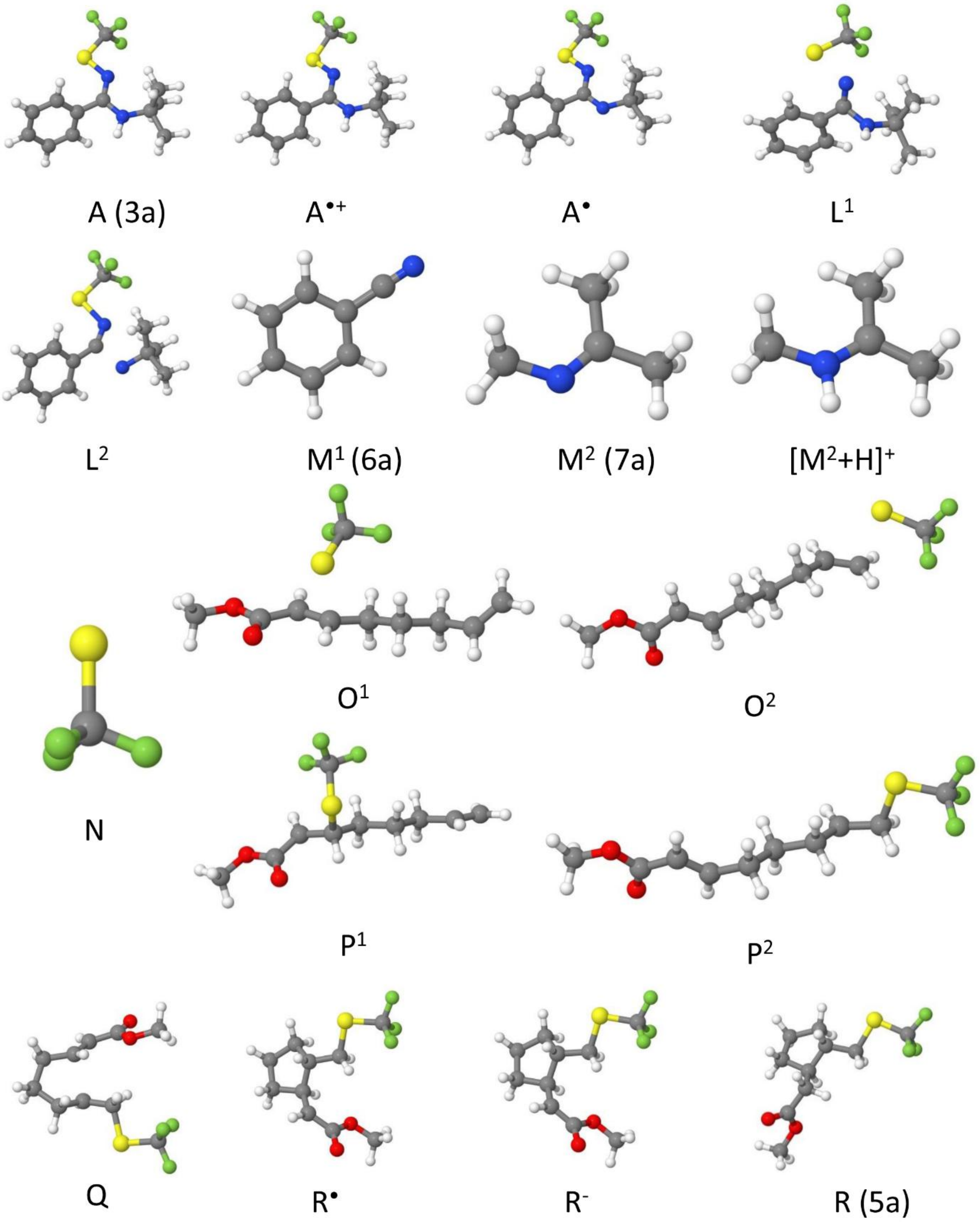

Figure S32: Optimized geometries of different intermediates of the model reaction ((CAM-B3LYP/TZVPP)/CPCM (MeCN)).

Table S57: Determination of the Gibbs Free Energy (in Hartree) at 298 K (CAM-B3LYP /TZVPP)/CPCM(MeCN) for all species involved in the presented mechanism.

	Electronic Energy	Thermal Corrections	ZPVE	Enthalpy Correctio ns	Entropy Correction s	Gibbs Free Energy
**A (3a)**	-1273.539061	0.018310	0.258764	0.000944	-0.063686	-1273.32473
**$A^{•+}$**	-1273.332322	0.017431	0.259358	0.000944	-0.062599	-1273.11719
**$A^{•}$**	-1272.876528	0.018020	0.245037	0.000944	-0.063811	-1272.67634
**L^1**	-1273.275836	0.016907	0.253948	0.000944	-0.061263	-1273.0653
**L^2**	-1272.836383	0.015992	0.241819	0.000944	-0.059701	-1272.63733
**M^1 (6a)**	-324.443057	0.006011	0.100308	0.000944	-0.037147	-324.372941
**M^2 (7a)**	-212.547721	0.006260	0.123783	0.000944	-0.036436	-212.45317
**$[M^2+H]^+$**	-213.014682	0.005736	0.137524	0.000944	-0.035401	-212.905879
**N**	-735.874728	0.004574	0.014579	0.000944	-0.034619	-735.88925
**O^1**	-1237.638027	0.016595	0.230154	0.000944	-0.061932	-1237.45227
**O^2**	-1237.644809	0.018394	0.229847	0.000944	-0.067581	-1237.46321
**P^1**	-1237.652368	0.017681	0.230805	0.000944	-0.064207	-1237.46715
**P^2**	-1237.658166	0.017877	0.230367	0.000944	-0.065808	-1237.47479
**Q**	-1237.641322	0.015653	0.231898	0.000944	-0.059857	-1237.45268
**$R^{•}$**	-1237.682047	0.016355	0.234465	0.000944	-0.061740	-1237.49202
**R^{-}**	-1237.819138	0.017220	0.233147	0.000944	-0.063637	-1237.63146
**R (5a)**	-1238.336828	0.015539	0.247719	0.000944	-0.059390	-1238.13202

To further understand the mechanistic pathway for releasing the trifluoromethylthiylradical **N** from the SCF_3-amidine radical cation (**$A^{•+}$**), various routes were computationally investigated from the thermodynamic and kinetic point of view. These studies showed that a decomposition to the corresponding diaziridine and tetrazine are thermodynamically unfavoured, as well as possible dimerization and rearrangement processes. Supported by the isolation of stoichiometric amounts of benzonitrile (**M^1**) from the reaction mixture, different concerted decomposition-rearrangement mechanisms from the SCF_3-amidine radical cation (**$A^{•+}$**) and its deprotonated counterpart (**$A^{•}$**) were investigated (Figure S33). According to these studies, the species **$A^{•+}$** firstly gets deprotonated by imine (**M^2**) (**7a**) or the starting material (**A**)(**3a**), followed by a concerted decomposition-rearrangement (**L^2**) leading to the release of trifluoromethylthiyl radical (**N**)**,** benzonitrile (**M^1**) (**6a**) and *N*-methylpropan-2-imine (**M^2**) (**6a**) (rate determining step). For the liberated •SCF_3 radical (**N**), addition to the model-substrate methyl-octa-2,7-dienoate (**4a**) can occur in various positions. The calculations demonstrate that

a reversible addition into the terminal position of the unactivated double bond is thermodynamically and kinetically most favoured, forming the alkyl radical **P²**. This radical undergoes a fast and irreversible intramolecular cyclization, forming **R•**. This more stabilized species can now close the catalytic cycle via reduction by the reduced form of the photocatalyst [Ir(dF(CF$_3$)ppy)$_2$(dtbpy)]. The thereby formed anion **R⁻** can undergo a proton abstraction from the prior protonated imine ([**M²**+H]$^+$) forming the product **R** (**5a**). A radical chain can be excluded in this reaction, since the energy gained from the reduction of the cyclised alkyl radical **R•** (117.5 kcal/mol) is lower than the energy (130.2 kcal/mol) needed to oxidize the SCF$_3$-amidin **A**.

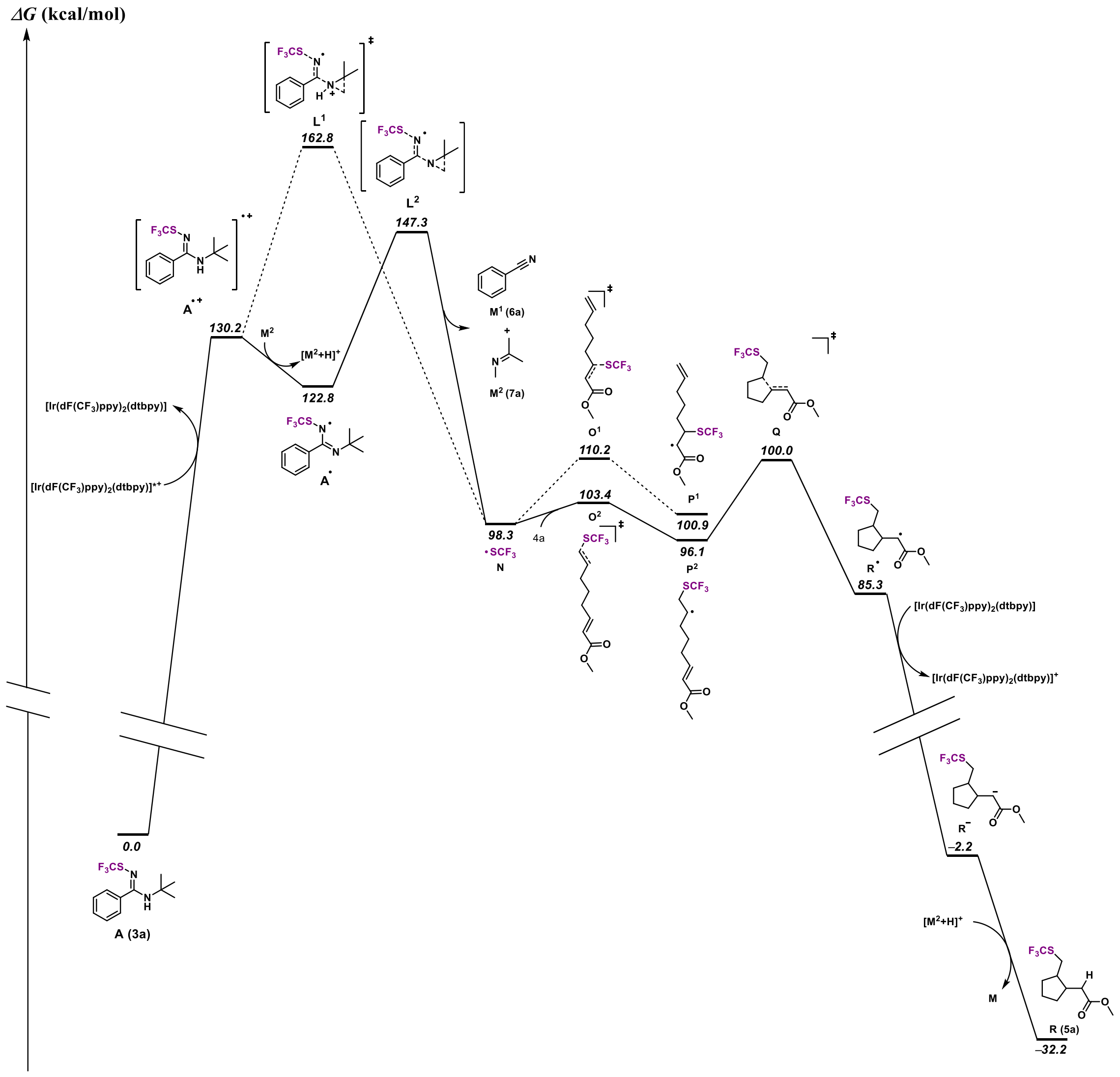

Figure S33: Proposed mechanism of the model reaction based on the computational studies (CAM-B3LYP/TZVPP)/CPCM (MeCN). For simplification just the most favoured, calculated pathways are shown.

8. Trifluoromethylthiolation of Heteroarenes

8.1 Preparation of Starting Materials

8.1.1 Synthesis of 4,5-Dichloro-imidazoles 8k to 8p and 8r to 8t.

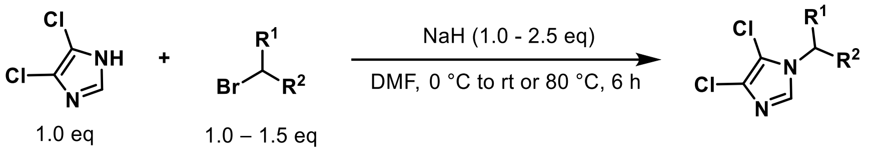

General Procedure D

4,5-Dichloro-1*H*-imidazole (1.0 eq) was dissolved in dimethylformamide (0.5 M) and after cooling down to 0 °C sodium hydride (1.2 eq, 60% dispersion in mineral oil) was added portion wise. The reaction mixture was stirred for 30 min at 0 °C followed by the addition of the alkyl or benzyl bromide (1.2 –1.5 eq). The reaction mixture was allowed to warm up to ambient temperature and stirred for another 6 h. Water (5.0 ml/mmol) was added to quench the reaction, the solution was extracted with EtOAc (3 x 5.0 mL/mmol), dried over Na_2SO_4 and the solvent was removed *in vacuo.* Purification by flash column chromatography gave the pure product.

General Procedure E

4,5-Dichloro-1*H*-imidazole (1.0 eq) was dissolved in dimethylformamide (0.5 M) and after cooling down to 0 °C sodium hydride (1.0 – 2.4 eq, 60% dispersion in mineral oil) was added portion wise. The reaction mixture was stirred for 30 min at 0 °C followed by the addition of the alkyl or benzyl bromide (1.0 – 1.2 eq). The reaction mixture was stirred overnight at 80 °C. Water (5.0 ml/mmol) was added to quench the reaction, the solution was extracted with EtOAc (3 x 5.0 mL/mmol), dried over Na_2SO_4 and the solvent was removed *in vacuo.* Purification by flash column chromatography gave the pure product.

3-(4,5-Dichloro-imidazol-1-yl)dihydrofuran-2(3*H*)-one (8e)

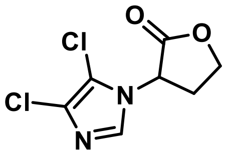

8o, quant.

Following the **general procedure D**, 4,5-dichloro-1*H*-imidazole (274 mg, 2.00 mmol, 1.0 eq), was reacted with sodium hydride (96.0 mg, 2.40 mmol, 1.2 eq, 60% dispersion in mineral oil) and 3-bromodihydrofuran-2(3*H*)-one (277 µL, 3.00 mmol, 1.5 eq) to give the product **8e** as a brown oil (446 mg, 2.00 mmol, quant.) after column flash chromatography (pentane:EtOAc, 4:1 to pure EtOAc v/v).

TLC (EtOAc): **R$_f$** = 0.34; **^{1}H NMR** (300 MHz, CDCl$_3$): δ 7.50 (s, 1H), 5.13 (dd, *J* = 11.7, 9.1 Hz, 1H), 4.61 (td, *J* = 9.2, 1.6 Hz, 1H), 4.43 (td, *J* = 10.2, 6.2 Hz, 1H), 3.01 – 2.81 (m, 1H), 2.62 (m, 1H); **^{13}C NMR** (75 MHz, CDCl$_3$): δ 170.7, 134.1, 127.3, 113.3, 65.5, 54.6, 30.0.; **HRMS (ESI)** (*m/z*): $[M + Na]^+$ calcd. for $C_7H_6Cl_2N_2O_2Na$ = 242.9699; found: 242.9702.

4,5-Dichloro-1-((perfluorophenyl)methyl)-imidazole (8m)

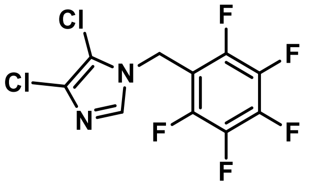

8k, quant.

Following the **general procedure D**, 4,5-dichloro-1*H*-imidazole (274 mg, 2.00 mmol, 1.0 eq), was reacted with sodium hydride (96.0 mg, 2.40 mmol, 1.2 eq, 60% dispersion in mineral oil) and 1-(bromomethyl)-2,3,4,5,6-pentafluorobenzene (453 µL, 3.00 mmol, 1.5 eq) to give the product **8m** as a yellow oil (641 mg, 2.00 mmol, quant.) after flash column chromatography (pentane:EtOAc, 9:1 to 4:1 v/v).

TLC (EtOAc): **R$_f$** = 0.60; **^{1}H NMR** (599 MHz, CDCl$_3$): δ 7.49 (s, 1H), 5.19 (s, 2H); **^{13}C NMR {^{19}F}** (151 MHz, CDCl$_3$): δ 145.5, 142.3, 137.9, 134.9, 126.9, 113.5, 108.3, 37.2; **^{19}F NMR** (282 MHz, CDCl$_3$): δ −141.1, −150.7, −159.9; **HRMS (ESI)** (*m/z*): $[M + Na]^+$ calcd. for $C_{10}H_3Cl_2F_5N_2H$ = 316.9666; found: 316.9667.

4,5-Dichloro-1-(4-((trifluoromethyl)thio)benzyl)-imidazole (8n)

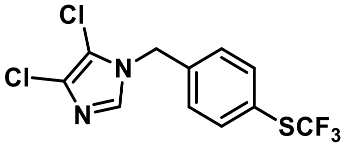

8l, 79%

Following the **general procedure D**, 4,5-dichloro-1*H*-imidazole (139 mg, 1.00 mmol, 1.0 eq), was reacted with sodium hydride (49.0 mg, 1.20 mmol, 1.2 eq, 60% dispersion in mineral oil) and (4-(bromomethyl)phenyl)(trifluoromethyl)sulfane (325 mg, 1.20 mmol, 1.2 eq) to give the product **8n** as a pale yellow solid (257 mg, 0.786 mmol, 79%) after flash column chromatography (pentane:EtOAc, 1:1 v/v).

TLC (EtOAc): **R$_f$** = 0.48; **^{1}H NMR** (300 MHz, $CDCl_3$): δ 7.60 (d, *J* = 8.2 Hz, 2H), 7.37 (s, 1H), 7.17 – 7.06 (m, 2H), 5.07 (s, 2H); **^{13}C NMR** (75 MHz, $CDCl_3$): δ 137.6, 137.1, 134.6, 131.5, 128.2, 127.0, 125.1, 49.2 (S*C*F^3-carbon not visible); **^{19}F NMR** (282 MHz, $CDCl_3$): δ −42.5; **HRMS (ESI)** (*m/z*): $[M + Na]^+$ calcd. for $C_{11}H_7Cl_2F_3N_2SNa$ = 348.9551; found: 348.9550.

***Tert*-butyl 4-((4,5-dichloro-imidazol-1-yl)methyl)benzoate (8o)**

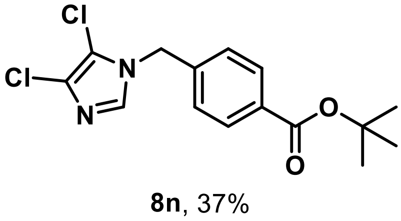

8n, 37%

Following the **general procedure D**, 4,5-dichloro-1*H*-imidazole (274 mg, 2.00 mmol, 1.0 eq), was reacted with sodium hydride (96.0 mg, 2.40 mmol, 1.2 eq, 60% dispersion in mineral oil) and *tert*-butyl 4-(bromomethyl)benzoate (813 µL, 3.00 mmol, 1.5 eq) to give the product **8o** as a pale yellow solid (242 mg, 0.738 mmol, 37%) after column flash chromatography (pentane:EtOAc, 7:3 v/v).

TLC (EtOAc): **R$_f$** = 0.44; **^{1}H NMR** (400 MHz, $CDCl_3$): δ 8.05 – 7.89 (m, 2H), 7.42 (s, 1H), 7.23 – 7.11 (m, 2H), 5.13 (s, 2H), 1.58 (s, 9H); **^{13}C NMR** (101 MHz, $CDCl_3$): δ 165.1, 138.8,

134.6, 132.5, 130.4, 127.1, 126.9, 113.8, 81.6, 49.6, 28.3; **HRMS (ESI)** (*m/z*): [M + Na]$^+$ calcd. for $C_{15}H_{16}Cl_2N_2O_2Na$ = 394.0481; found: 394.0454.

4,5-Dichloro-1-(3-methoxypropyl)-imidazole (8p)

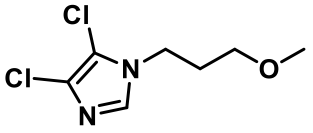

8m, quant.

Following the **general procedure D**, 4,5-dichloro-1*H*-imidazole (411 mg, 3.00 mmol, 1.0 eq), was reacted with sodium hydride (144.0 mg, 3.60 mmol, 1.2 eq, 60% dispersion in mineral oil) and 1-bromo-3-methoxypropane (507 µL, 4.50 mmol, 1.5 eq) to give the product **8p** as an amber oil (627 mg, 3.00 mmol, quant.) after flash column chromatography (pentane:EtOAc, 3:1 to 1:1 v/v).

TLC (EtOAc): $\mathbf{R_f}$ = 0.35; **^{1}H NMR** (400 MHz, $CDCl_3$): δ 7.34 (s, 1H), 4.02 (t, J = 6.8 Hz, 2H), 3.32 (s, 3H), 3.29 (t, J = 5.7 Hz, 2H), 1.97 (tt, J = 6.8, 5.7 Hz, 2H); **^{13}C NMR** (101 MHz, $CDCl_3$): δ 134.8, 126.2, 113.2, 68.1, 58.8, 43.2, 29.8; **HRMS (ESI)** (*m/z*): [M + Na]$^+$ calcd. for $C_7H_{10}Cl_2N_2ONa$ = 231.0062; found: 231.0074.

4-(4,5-Dichloro-imidazol-1-yl)butanenitrile (8q)

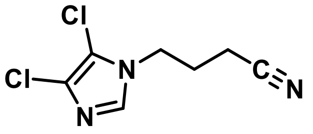

8r, 99%

Following the **general procedure D**, 4,5-dichloro-1*H*-imidazole (411 mg, 3.00 mmol, 1.0 eq), was reacted with sodium hydride (144 mg, 3.60 mmol, 1.2 eq, 60% dispersion in mineral oil) and 4-bromobutanenitrile (447 µL, 4.50 mmol, 1.5 eq) to give the product **8q** as a yellow oil (603 mg, 2.96 mmol, 99%) after flash column chromatography (pentane:EtOAc, 3:1 to 1:1 v/v).

TLC (EtOAc): $\mathbf{R_f}$ = 0.31; **^{1}H NMR** (400 MHz, $CDCl_3$): δ 7.41 (s, 1H), 4.09 (t, J = 6.9 Hz, 2H), 2.37 (t, J = 6.9 Hz, 2H), 2.12 (p, J = 6.9 Hz, 2H); **^{13}C NMR** (101 MHz, $CDCl_3$): δ 134.6, 127.0,

118.0, 113.1, 44.4, 25.8, 14.4; **HRMS (ESI)** (*m/z*): $[M + Na]^+$ calcd. for $C_7H_7Cl_2N_3Na$ = 225.9909; found: 225.9923.

2-(4,5-Dichloro-1*H*-imidazol-1-yl)ethan-1-ol (8r)

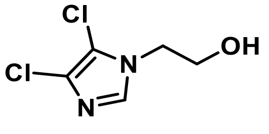

8p, 89%

Following the **general procedure E**, 4,5-dichloro-1*H*-imidazole (274 mg, 2.00 mmol, 1.0 eq), was reacted with sodium hydride (80.0 mg, 2.00 mmol, 1.0 eq, 60% dispersion in mineral oil) and 2-bromoethan-1-ol (168 mg, 2.00 mmol, 1.0 eq) to give the product **8r** as an amber solid (322 mg, 1.78 mmol, 89%) after flash column chromatography (EtOAc).

TLC (EtOAc): $\mathbf{R_f}$ = 0.27; **^{1}H NMR** (400 MHz, $CDCl_3$): δ 7.27 (s, 1H), 4.37 (s, 1H), 3.96 (dd, *J* = 5.6, 3.9 Hz, 2H), 3.84 (dd, *J* = 5.5, 3.9 Hz, 2H); **^{13}C NMR** (101 MHz, $CDCl_3$): δ 135.5, 125.7, 113.2, 60.0, 49.5; **HRMS (ESI)** (*m/z*): $[M + Na]^+$ calcd. for $C_5H_6Cl_2N_2ONa$ = 202.9794; found: 202.9781.

2-((4,5-Dichloro-imidazol-1-yl)methyl)pyridine (8s)

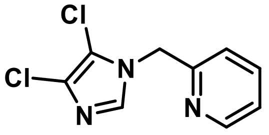

8s, 78%

Following the **general procedure E**, 4,5-dichloro-1*H*-imidazole (200 mg, 1.46 mmol, 1.0 eq), was reacted with sodium hydride (140 mg, 3.50 mmol, 2.4 eq, 60% dispersion in mineral oil) and 2-(bromomethyl)pyridine hydrobromide (813 mg, 1.75 mmol, 1.2 eq) to give the product **8s** as an orange solid (260 mg, 1.14 mmol, 78%) after flash column chromatography (pentane:EtOAc, 1:1 v/v).

TLC (EtOAc): $\mathbf{R_f}$ = 0.20; **^{1}H NMR** (400 MHz, $CDCl_3$): δ 8.59 (ddd, *J* = 4.8, 1.8, 1.0 Hz, 1H), 7.69 (td, *J* = 7.7, 1.8 Hz, 1H), 7.53 (s, 1H), 7.26 (dt, *J* = 4.8, 3.8 Hz, 1H), 7.03 (d, *J* = 7.8 Hz, 1H), 5.20 (s, 2H); **^{13}C NMR** (101 MHz, $CDCl_3$): δ 154.3, 150.1, 137.4, 135.1, 126.6, 123.5,

121.5, 113.7, 51.3; **HRMS (ESI)** (*m/z*): $[M + Na]^+$ calcd. for $C_9H_8Cl_2N_3Na$ = 249.9909; found: 249.9921.

Diethyl (3-(4,5-dichloro-imidazol-1-yl)propyl)phosphonate (8t)

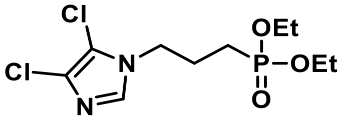

8t, 94%

Following the **general procedure D**, 4,5-dichloro-1*H*-imidazole (274 mg, 2.00 mmol, 1.0 eq), was reacted with sodium hydride (96.0 mg, 2.40 mmol, 1.2 eq, 60% dispersion in mineral oil) and diethyl (3-bromopropyl)phosphonate (577 mg, 3.00 mmol, 1.5 eq) to give the product **8t** as a yellow solid (565 mg, 1.89 mmol, 94%) after flash column chromatography (EtOAc, to EtOAc: MeOH, 1:1 v/v).

TLC (EtOAc): $\mathbf{R_f}$ = 0.18; ^{1}**H NMR** (400 MHz, $CDCl_3$): δ 7.41 (s, 1H), 4.25 – 3.83 (m, 6H), 2.24 – 1.87 (m, 2H), 1.66 (ddd, J = 18.8, 8.2, 7.0 Hz, 2H), 1.30 (t, J = 7.1 Hz, 6H); ^{13}**C NMR** (101 MHz, $CDCl_3$): δ 134.6, 126.5, 113.1, 62.0 (d, J = 6.6 Hz), 46.0 (d, J = 14.2 Hz), 23.4 (d, J = 4.8 Hz), 22.3 (d, J = 143.5 Hz) 16.6 (d, J = 5.9 Hz); **HRMS (ESI)** (*m/z*): $[M + Na]^+$ calcd. for $C_{10}H_{17}Cl_2N_2O_3PNa$ = 337.0246; found: 337.0251.

8.1.2 Synthesis of Pyrroles 8u to 8v.

2,3,4-Trichloro-1-methyl-5-phenyl-1H-pyrrole (8u)

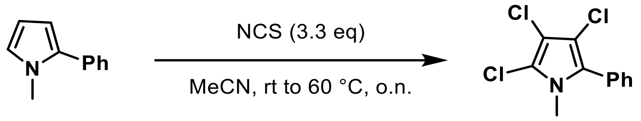

1.0 eq   **8u**, 49%

N-Chlorosuccinimide (467 mg, 3.3 mmol, 3.3 eq) was dissolved in acetonitrile (10 mL), 1-methyl-2-phenyl-1H-pyrrole[40] (157 mg, 1.0 mmol, 1.0 eq) was added and the reaction mixture was stirred at 60 °C overnight. The solvent was removed *in vacuo*, before flash column chromatography (pentane:EtOAc, 33:1 v/v) gave the product **8u** as a yellow oil (128 mg, 0.49 mmol, 49%).

TLC (pentane:EtOAc, 9:1 v/v): $\mathbf{R_f}$ = 0.42; **^{1}H NMR** (400 MHz, $CDCl_3$): δ 7.50 – 7.38 (m, 3H), 7.38 – 7.34 (m, 2H), 3.48 (s, 3H); **^{13}C NMR** (101 MHz, $CDCl_3$): δ 130.3, 129.3, 128.9, 128.6, 128.6, 114.25, 108.7, 108.2, 33.2; **HRMS (ESI)** (*m/z*): $[M + h]^+$ calcd. for $C_{1!}H_8Cl_3NH$ = 259.9795; found: 259.9795.

2,3,4-Tribromo-1-methyl-5-phenyl-1*H*-pyrrole (8v)

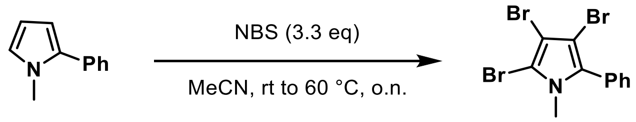

1.0 eq   **8v**, 79%

N-Bromosuccinimide (587 mg, 3.3 mmol, 3.3 eq) was dissolved in acetonitrile (10 mL), 1-methyl-2-phenyl-1*H*-pyrrole[40] (157 mg, 1.0 mmol, 1.0 eq) was added and the reaction mixture was stirred at 60 °C overnight. The solvent was removed *in vacuo* and purification by flash column chromatography (pentane:EtOAc, 33:1 v/v) gave the product **8v** as a colourless glass (310 mg, 0.79 mmol, 79%).

TLC (pentane:EtOAc, 9:1 v/v): $\mathbf{R_f}$ = 0.42; **^{1}H NMR** (400 MHz, $CDCl_3$): δ 7.45 (m, 3H), 7.34 (m, 2H), 3.52 (s, 3H); **^{13}C NMR** (101 MHz, $CDCl_3$): δ 137.6, 130.4, 129.4, 128.8, 128.7, 114.4, 108.9, 108.4, 33.3.; **HRMS (ESI)** (*m/z*): $[M + Na]^+$ calcd. for $C_{10}H_8Br_3NNa$ = 415.8079; found: 415.8070.

8.2. Reaction Optimization

8.2.1 General Reaction Optimization

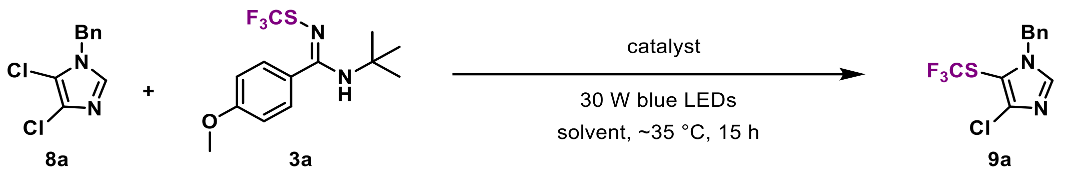

Unless otherwise noted, 1-benzyl-4,5-dichloro-1*H*-imidazole (**8a**) (22.7 mg, 0.10 mmol, 1.0 eq), *N*-(*tert*-butyl)-4-methoxy-*N'*-((trifluoromethyl)thio)benzimidamide (**3b**) (46.0 mg, 0.15 mmol, 1.5 eq) and [Ir(dFCF$_3$ppy)$_2$(dtbpy)][PF$_6$] (**PC1**) (2.2 mg, 0.0020 mmol, 2.0 mol%), were weighed into a Schlenk tube containing a magnetic stirring bar. The tube was evacuated and backfilled with argon three times. Dimethyl sulfoxide (1.0 mL) was added and the reaction was stirred under irradiation with 450 nm LEDs for 16 h at room temperature. 1,3,5-Trimethoxybenzene (1 M in EtOAc, 100 µL, 0.10 mmol, 1.0 eq) was added to the reaction mixture as an internal standard and an aliquot (30 µL) of the crude reaction mixture was filtered over a plug of silica (elution with EtOAc). The yield was determined via calibrated GC-FID.

Table S58: Optimization with respect to the solvent.

**Entry**	**Solvent**	**Yield**
**1**	DMF	6%
**2**	CH_2Cl_2	81%
**3**	THF	0%
**4**	MeCN	78%
**5**	MeOH	6%
**6**	toluene	60%
**7**	$PhCF_3$	63%
**8**	EtOAc	47%
**9**	**DMSO**	**97%**

Standard conditions: **8a** (1.0 eq), **3b** (1.5 eq), [Ir(dFCF$_3$ppy)$_2$(dtbpy)][PF$_6$] (**PC1**) (2.0 mol%), solvent (0.1 M), 16 h, ~35 °.

Table S59: Optimization with respect to the catalyst loading.

Entry	Catalyst Loading (mol%)	Yield
**1**	0.5	90%
**2**	1.0	94%
**3**	2.0	96%
**4**	**2.5**	**98%**

Conditions: **8a** (1.0 eq.), **3b** (1.2 eq.), [Ir(dFCF$_3$ppy)$_2$(dtbpy)][PF$_6$] (**PC1**), DMSO (0.1 M), 450 nm LED, 16 h, ~35 °C.

Table S60: Optimization with respect to the stoichiometry.

Entry	Eq of 8a	Eq of 3b	Yield
**1**	1.0	1.0	80%
**2**	1.0	1.2	93%
**3**	**1.0**	**2.0**	**98%**
**4**	1.5	1.0	83%

Standard conditions: [Ir(dFCF$_3$ppy)$_2$(dtbpy)][PF$_6$] (**PC1**) (1.0 mol%), DMSO (0.1 M), 450 nm LED, 16 h, ~35 °C.

Table S61: Optimization with respect to the SCF$_3$-reagent.

Entry	SCF$_3$-Reagent	Yield
**1**	**3a**	89%
**2**	**3b**	**92%**
**3**	**3c**	45%
**4**	**3d**	41%
**5**	**3e**	33%
**6**	**3f**	9%

Standard conditions: **8a** (1.0 eq), **3** (1.2 eq), [Ir(dFCF$_3$ppy)$_2$(dtbpy)][PF$_6$] (**PC1**) (1.0 mol%), 450 nm LED, DMSO (0.1 M), 16 h, ~35 °C.

Table S62: Optimization with respect to the reaction concentration.

Entry	Concentration (mol/L)	Yield
**1**	**0.05**	**96%**
**2**	0.1	94%
**3**	0.2	80%
**4**	0.5	51%

Standard conditions: **8a** (1.0 eq), **3b** (1.5 eq), [Ir(dFCF$_3$ppy)$_2$(dtbpy)][PF$_6$] (**PC1**) (1.0 mol%), DMSO, 450 nm LED, 16 h, ~35 °C.

Table S63: Optimization with respect to the catalyst.

Entry	Catalyst	Yield
**1**	**[Ir(dF(CF$_3$)ppy)$_2$(dtbpy)][PF$_6$] (PC1)**	**95%**
**2**	[Ir(ppy)$_3$] (**PC2**)	traces
**3**	[Ru(bpz)$_3$][PF$_6$]$_2$ (**PC3**)	traces
**4**[a]	MesAcr$^+$BF$_4^-$	traces
**5**[a]	TPP$^+$BF$_4^-$	5%
**6**[a]	4-CzIPN	37%

Standard conditions: **8a** (1.0 eq), **3b** (1.2 eq), catalyst (1.0 mol%), DMSO (0.1 M), 450 nm LED, 16 h, ~35 °C; a) catalyst (10 mol%).

Table S64: Control and competition experiments.

Entry	Control/Competition	Yield
**1**	under air atmosphere	76%
**2**	no light	n.r.
**3**	no catalyst	n.r.
**4**	methyl 3-mercaptopropanoate (1.5 eq)	37%
**5**	**1a** (1.5 eq) and **2** (1.5 eq) instead of **3b**	92%

Standard conditions: **8a** (1.0 eq), **3b** (1.5 eq), [Ir(dFCF$_3$ppy)$_2$(dtbpy)][PF$_6$] (**PC1**) (2.0 mol%), DMSO (0.1 M), 450 nm LED, 16 h, ~35 °C.

8.2.1 Reaction Profile

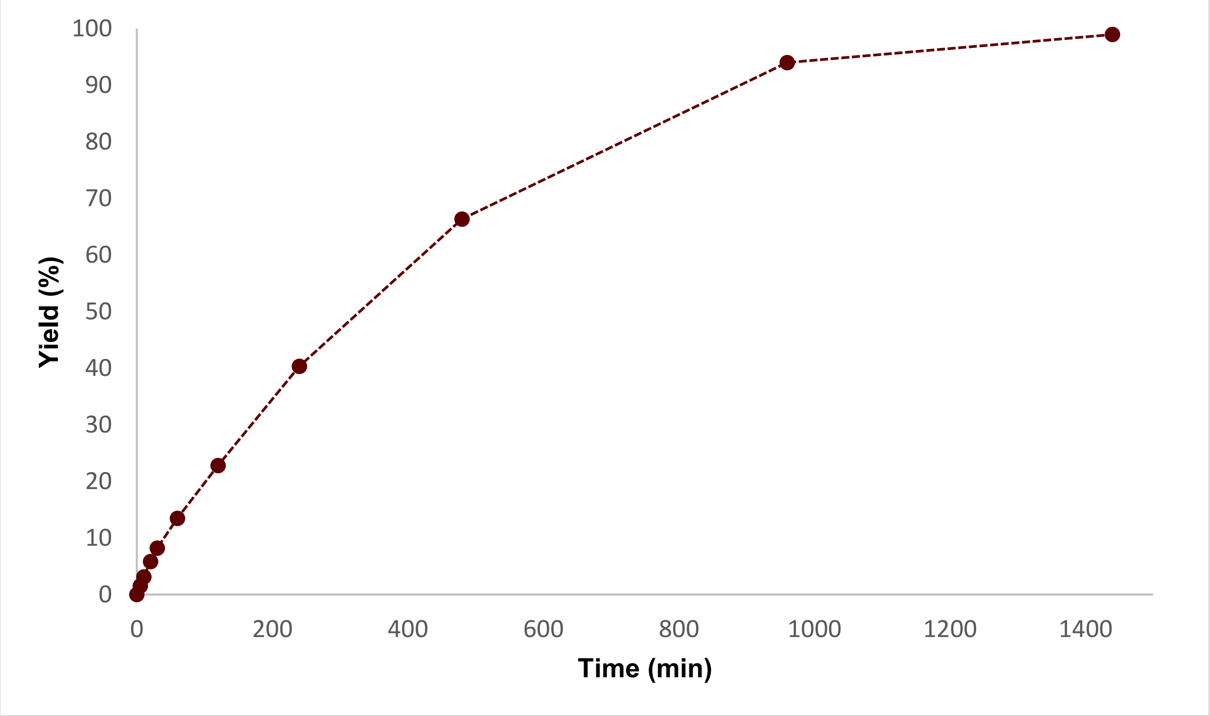

Figure S34: Reaction profile for the trifluoromethylthiolation of imidazole **8a** to give **9a**.

To obtain a reaction time profile, 1-benzyl-4,5-dichloro-1*H*-imidazole (**8a**) (68.1 mg, 0.30 mmol, 1.0 eq), *N*-(*tert*-butyl)-4-methoxy-*N'*-((trifluoromethyl)thio)benzimidamide (**3b**) (138 mg, 0.45 mmol, 1.5 eq), [Ir(dFCF$_3$ppy)$_2$(dtbpy)][PF$_6$] (**PC1**) (3.3 mg, 0.003 mmol, 1.0 mol%) and 1,3,5-trimethoxybenzene (50.5 mg, 0.30 mmol, 1.0 eq) as internal standard were weighed into a Schlenk tube containing a magnetic stirring bar. The tube was evacuated and backfilled with argon three times. Dimethyl sulfoxide (3.0 mL) was added and the reaction was stirred under irradiation with 450 nm LEDs while staying attached to the Schlenk line. At different times after starting the irradiation (0, 5, 10, 20, 30, 60, 120, 240, 480, 1380 min), an aliquot (30 µL) of the reaction mixture was taken under a stream of argon, using an argon purged air displacement pipette. The aliquot was filtered over a plug of silica (elution with EtOAc) and the yield at the corresponding time point was determined via calibrated GC-FID (Figure S34). Full conversion was observed after 24 h. To ensure high yields and high conversion for different substrates, the catalyst loading was set to 2 mol%, while 1.5 eq of the SCF$_3$-amidine was used and the reaction time was set to 24 h. Since only minor changes in yield could be achieved by increasing the loading of the SCF$_3$-amidine as well as by the reduction of the reaction concentration, the conditions were chosen as described in **general procedure F** in order to save resources.

8.3 Scope of the Trifluoromethylthiolation of Heteroarenes

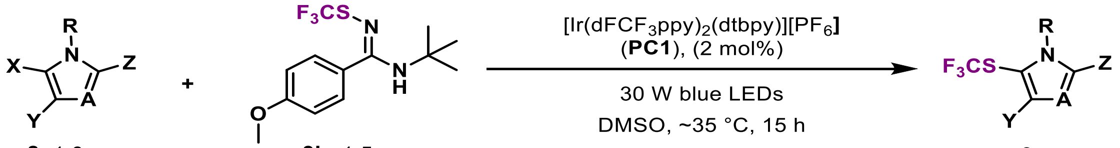

General Procedure F

Unless otherwise noted, the corresponding heteroarene (**8**) (0.30 mmol, 1.0 eq), *N*-(*tert*-butyl)-4-methoxy-*N'*-((trifluoromethyl)thio)benzimidamide (**3b**) (137 mg, 0.45 mmol, 1.5 eq) and $[Ir(dFCF_3ppy)_2(dtbpy)][PF_6]$ (**PC1**) (6.7 mg, 0.006 mmol, 2.0 mol%) were weighed into a Schlenk tube containing a magnetic stirring bar. The tube was evacuated and backfilled with argon three times. Dimethyl sulfoxide (3.0 mL) was added and the reaction was stirred under irradiation with 450 nm LEDs for 24 h at room temperature. The crude mixture was diluted with ethyl acetate (5.0 mL), followed by the addition of brine (5.0 mL) and water (5 mL). The layers were separated, the aqueous layer was extracted with ethyl acetate (2 x 10 mL) and the combined organic layers were dried over Na_2SO_4. The solvent was removed *in vacuo* and the crude product was purified by flash column chromatography to obtain the pure products.

1-Benzyl-4-chloro-5-((trifluoromethyl)thio)-1*H*-imidazole (9a)

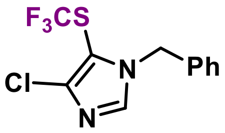

9a, 95%

Following **general procedure F**, using 1-benzyl-4,5-dichloro-1*H*-imidazole[41] (**8a**) (67.8 mg, 0.30 mmol, 1.0 eq), the pure product **9a** (83.6 mg, 0.29 mmol, 95%) was obtained as a white solid after flash column chromatography (CH_2Cl_2).

TLC (EtOAc): **R**$_f$ = 0.66; **^{1}H NMR** (599 MHz, $CDCl_3$): δ 7.57 (s, 1H), 7.45 – 7.33 (m, 3H), 7.20 – 7.15 (m, 2H), 5.20 (s, 2H); **^{13}C NMR** (151 MHz, $CDCl_3$): δ 142.6, 140.0, 134.3, 129.4, 129.1, 128.02 (q, J = 313.3 Hz), 127.8, 108.3, 50.4; **^{19}F NMR** (563 MHz, $CDCl_3$): δ −43.3; **HRMS (ESI)** (*m/z*): $[M + Na]^+$ calcd. for $C_{11}H_8ClF_3N_2SNa$ = 314.9941; found: 314.9948.

4-Bromo-1-methyl-5-((trifluoromethyl)thio)-1*H*-imidazole (9b)

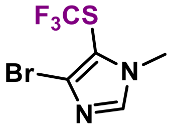

9c, 73%

Following **general procedure F**, using 4,5-dibromo-1-methyl-1*H*-imidazole (**8b**) (71.7 mg, 0.30 mmol, 1.0 eq), the pure product **9b** (57.1 mg, 0.22 mmol, 73%) was obtained as a colourless solid after flash column chromatography (pentane:EtOAc 1:1 v/v).

TLC (pentane:EtOAc, 1:1 v/v): $\mathbf{R_f}$ = 0.26; **^{1}H NMR** (400 MHz, $CDCl_3$): δ 7.65 (s, 1H), 3.74 (s, 3H); **^{13}C NMR** (101 MHz, $CDCl_3$): δ 141.4, 130.5, 127.9 (q, J = 313.1 Hz), 111.7, 33.3; **^{19}F NMR** (376 MHz, $CDCl_3$): δ −43.4; **HRMS (ESI)** (*m/z*): $[M + Na]^+$ calcd. for $C_{11}H_7F_3N_2SBrClNa$ = 284.9102; found: 284.9093.

2,4-Dibromo-1-methyl-5-((trifluoromethyl)thio)-1*H*-imidazole (9c)

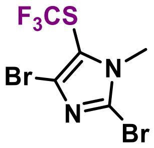

9d, 95%

Following a modified version of **general procedure F**, using 2,4,5-tribromo-1-methyl-1*H*-imidazole (**8c**) (95.6 mg, 0.30 mmol, 1.0 eq) and *N*-(*tert*-butyl)-4-methoxy-*N'*-((trifluoromethyl)thio)benzimidamide (**3b**) (110 mg, 0.60 mmol, 1.3 eq), the pure product **9c** (96.5 mg, 0.28 mmol, 95%) was obtained as a yellow solid after flash column chromatography (pentane:EtOAc 4:1 v/v).

TLC (pentane:EtOAc, 1:1 v/v): $\mathbf{R_f}$ = 0.54; **^{1}H NMR** (400 MHz, $CDCl_3$): δ 3.72 (s, 3H); **^{13}C NMR** (151 MHz, $CDCl_3$): δ 129.8, 127.6 (q, J = 313.6 Hz), 124.8, 114.4, 34.2; **^{19}F NMR** (376 MHz, $CDCl_3$): δ −43.4; **HRMS (ESI)** (*m/z*): $[M + H]^+$ calcd. for $C_5H_3F_3Br_2N_2SH$ = 362.8207; found: 362.8215.

Methyl (*S*)-2-((*tert*-butoxycarbonyl)amino)-3-(5-((trifluoromethyl)thio)-1*H*-imidazol-1-yl)propanoate (9d)

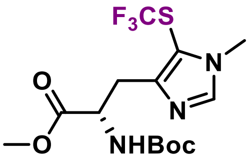

9q, 98%

Following **general procedure F**, using methyl (S)-2-((*tert*-butoxycarbonyl)amino)-3-(4,5-dichloro-1*H*-imidazol-1-yl)propanoate[41] (**8d**) (95.1 mg, 0.30 mmol, 1.0 eq), the pure product **9d** (112.3 mg, 0.29 mmol, 98%) was obtained as a pale yellow solid after flash column chromatography (pentane:EtOAc 1:1 v/v).

TLC (EtOAc): **R$_f$** = 0.31; **^{1}H NMR** (400 MHz, $CDCl_3$): δ 7.59 (s, 1H), 5.91 (d, *J* = 8.4 Hz, 1H), 4.56 (dt, *J* = 8.4, 5.2 Hz, 1H), 3.63 (s, 3H), 3.61 (s, 3H), 3.20 (dd, *J* = 15.0, 5.6 Hz, 1H), 3.07 (dd, *J* = 14.9, 5.0 Hz, 1H), 1.35 (s, 9H); **^{13}C NMR** (101 MHz, $CDCl_3$): δ 172.3, 155.4, 149.0, 141.4, 128.0 (q, *J* = 312.1 Hz), 109.5, 79.6, 52.7, 52.3, 32.2, 29.2, 28.3; **^{19}F NMR** (376 MHz, $CDCl_3$): δ −44.3; **HRMS (ESI)** (*m/z*): $[M + H]^+$ calcd. for $C_{14}H_{20}F_3O_4N_2SH$ = 403.0230; found: 403.0234.

3-(4-Chloro-5-((trifluoromethyl)thio)-1*H*-imidazol-1-yl)dihydrofuran-2(3H)-one (9e)

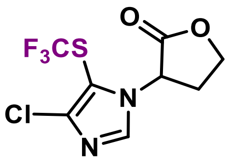

9o, 95%

Following **general procedure F**, using 3-(4,5-dichloro-1*H*-imidazol-1-yl)dihydrofuran-2(3H)-one (**8e**) (66.3 mg, 0.30 mmol, 1.0 eq), the pure product **9e** (81.4 mg, 0.28 mmol, 95%) was obtained as a pale yellow solid after flash column chromatography (pentane:EtOAc 4:1 v/v to pure EtOAc).

TLC (EtOAc): **R$_f$** = 0.34; **^{1}H NMR** (400 MHz, $CDCl_3$): δ 7.73 (s, 1H), 5.30 (dd, *J* = 12.1, 8.8 Hz, 1H), 4.62 (td, *J* = 9.2, 1.3 Hz, 1H), 4.46 (ddd, *J* = 11.1, 9.6, 5.9 Hz, 1H), 2.93 (dddd, *J* = 12.9, 8.8, 5.9, 1.3 Hz, 1H), 2.54 (tdd, *J* = 12.7, 11.2, 8.9 Hz, 1H); **^{13}C NMR** (101 MHz, $CDCl_3$):

δ 171.0, 142.9, 138.7, 127.8 (q, J = 313.7 Hz), 108.9, 65.4, 54.3, 31.5; **^{19}F NMR** (376 MHz, $CDCl_3$): δ −43.0; **HRMS (ESI)** (*m/z*): $[M + Na]^+$ calcd. for $C_8H_7F_6N_2ClS_2Na$ = 308.9663; found: 308.9685.

1-Methyl-5-((trifluoromethyl)thio)-1*H*-imidazole (9f)

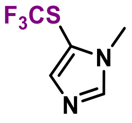

9h, 42%

Following **general procedure F**, using 5-bromo-1-methyl-1*H*-imidazole (**8f**) (48.3 mg, 0.30 mmol, 1.0 eq), the pure product **9f** (23.0 mg, 0.13 mmol, 42%) was obtained as a pale yellow oil after flash column chromatography (EtOAc:MeOH, 49:1 v/v).

TLC (EtOAc:MeOH, 19:1 v/v): **R_f** = 0.22; **^{1}H NMR** (400 MHz, $CDCl_3$): δ 7.72 (s, 1H), 7.48 (s, 1H), 3.72 (s, 3H); **^{13}C NMR** (151 MHz, $CDCl_3$): δ 142.7, 141.6, 128.0 (q, J = 314.7 Hz), 111.7, 31.9; **^{19}F NMR** (376 MHz, $CDCl_3$): δ −44.6; **HRMS (ESI)** (*m/z*): $[M + H]^+$ calcd. for $C_5H_5F_3N_2SH$ = 183.0198; found: 183.0196.

1,4-Dimethyl-2-((trifluoromethyl)thio)-1*H*-imidazole (9g)

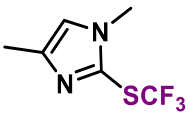

9i, 59%

Following a modified version of **general procedure F**, using 2-bromo-1,4-dimethyl-1*H*-imidazole (**8g**) (52.5 mg, 0.30 mmol, 1.0 eq) and *N*-(*tert*-butyl)-4-methoxy-*N'*-((trifluoromethyl)thio)benzimidamide (**3b**) (230 mg, 0.60 mmol, 2.5 eq), the pure product **9g** (34.8 mg, 0.18 mmol, 59%) was obtained as a sticky yellow oil after flash column chromatography (pentane:EtOAc, 4:1 v/v).

TLC (pentane:EtOAc, 1:1 v/v): **R_f** = 0.33; **^{1}H NMR** (400 MHz, $CDCl_3$): δ 6.90 (s, 1H), 3.74 (s, 3H), 2.23 (s, 3H); **^{13}C NMR** (151 MHz, $CDCl_3$): δ 141.1, 128.3 (q, J = 311.8 Hz), 128.1,

123.0, 34.2, 13.9; **^{19}F NMR** (376 MHz, $CDCl_3$): δ −42.2; **HRMS (ESI)** (*m/z*): $[M + H]^+$ calcd. for $C_6H_7F_3N_2SH$ = 197.0355; found: 197.0370.

1-Ethyl-2-methyl-5-((trifluoromethyl)thio)-1*H*-imidazole (9h)

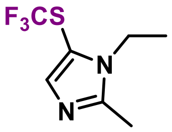

9j, 71%

Following **general procedure F**, using 5-chloro-1-ethyl-2-methyl-1*H*-imidazole (**8h**) (38.0 µL, 0.30 mmol, 1.0 eq), the pure product **9h** (44.7 mg, 0.21 mmol, 71%) was obtained as a colourless oil after flash column chromatography (EtOAc:MeOH, 49:1 v/v).

TLC (EtOAc:MeOH, 19:1 v/v): $\mathbf{R_f}$ = 0.26; **^{1}H NMR** (600 MHz, $CDCl_3$): δ 7.36 (s, 1H), 4.05 (q, *J* = 7.3 Hz, 2H), 2.45 (s, 3H), 1.29 (t, *J* = 7.3 Hz, 3H); **^{13}C NMR** (101 MHz, $CDCl_3$): δ 149.7, 140.4, 127.8 (q, *J* = 313.0 Hz), 109.7, 38.8, 15.6, 14.4; **^{19}F NMR** (376 MHz, $CDCl_3$): δ −45.2; **HRMS (ESI)** (*m/z*): $[M + H]^+$ calcd. for $C_7H_9F_3N_2SH$ = 211.0511; found: 211.0510.

1-Benzyl-2-bromo-4-chloro-5-((trifluoromethyl)thio)-1*H*-imidazole (9i)

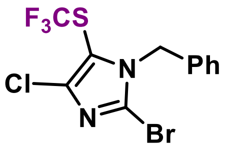

9e, 87%

Following **general procedure F**, using 1-benzyl-2-bromo-4,5-dichloro-1*H*-imidazole[41] (**8i**) (91.8 mg, 0.30 mmol, 1.0 eq), the pure product **9i** (96.8 mg, 0.26 mmol, 87%) was obtained as a white solid after flash column chromatography (pentane:EtOAc 9:1 to 7:3 v/v).

TLC (pentane:EtOAc, 1:1 v/v): $\mathbf{R_f}$ = 0.86; **^{1}H NMR** (300 MHz, $CDCl_3$): δ 7.33 – 7.21 (m, 3H), 7.01 (dd, *J* = 7.4, 2.2 Hz, 2H), 5.24 (s, 2H); **^{13}C NMR** (75 MHz, $CDCl_3$): δ 142.5, 134.2, 129.7, 128.5, 127.6 (q, *J* = 313.8 Hz), 126.7, 124.1, 111.0, 50.3; **^{19}F NMR** (282 MHz, $CDCl_3$): δ −43.2; **HRMS (ESI)** (*m/z*): $[M + Na]^+$ calcd. for $C_{11}H_7F_3N_2SBrClNa$ = 394.9025; found: 394.9027.

4-Chloro-1-methyl-5-((trifluoromethyl)thio)-1*H*-imidazole (9j)

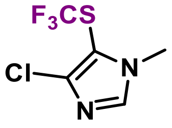

9b, 96%

Following **general procedure F**, using 4,5-dichloro-1-methyl-1*H*-imidazole (**8j**) (45.3 mg, 0.30 mmol, 1.0 eq), the pure product **9j** (62.4 mg, 0.29 mmol, 96%) was obtained as a pale yellow oil after flash column chromatography (pentane:EtOAc, 1:1 v/v).

TLC (pentane:EtOAc, 1:1 v/v): $\mathbf{R_f}$ = 0.26; **^{1}H NMR** (400 MHz, $CDCl_3$): δ 7.61 (s, 1H), 3.71 (s, 3H); **^{13}C NMR** (151 MHz, $CDCl_3$): δ 142.2, 140.3, 128.1 (q, J = 312.9 Hz), 108.5, 33.3; **^{19}F NMR** (376 MHz, $CDCl_3$): δ −43.6; **HRMS (ESI)** (*m/z*): $[M + Na]^+$ calcd. for $C_5H_4ClF_3N_2SNa$ = 238.9628; found: 238.9632.

2-Chloro-1-(4-chlorobenzyl)-5-((trifluoromethyl)thio)-1*H*-imidazole (9k)

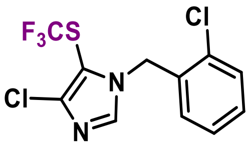

9f, quant.

Following **general procedure F**, using 4,5-dichloro-1-(2-chlorobenzyl)-1*H*-imidazole[41] (**8k**) (78.2 mg, 0.30 mmol, 1.0 eq), the pure product **9k** (98.9 mg, 0.30 mmol, quant.) was obtained as a yellow solid after flash column chromatography (pentane:EtOAc 4:1 v/v).

TLC (pentane:EtOAc, 1:1 v/v): $\mathbf{R_f}$ = 0.63; **^{1}H NMR** (400 MHz, $CDCl_3$): δ 7.49 (s, 1H), 7.38 (dd, J = 7.9, 1.5 Hz, 1H), 7.26 (td, J = 7.7, 1.8 Hz, 1H), 7.21 (td, J = 7.5, 1.9 Hz, 1H), 6.94 (dd, J = 7.6, 1.7 Hz, 1H), 5.24 (s, 2H); **^{13}C NMR** (101 MHz, $CDCl_3$): δ 142.4, 140.1, 133.6, 132.0, 130.4, 130.1, 129.6, 127.9 (q, J = 312.4 Hz), 127.6, 108.3, 47.9; **^{19}F NMR** (376 MHz, $CDCl_3$): δ −43.3; **HRMS (ESI)** (*m/z*): $[M + Na]^+$ calcd. for $C_{11}H_7F_3N_2Cl_2SNa$ = 348.9551; found: 348.9542.

1-(4-Bromobenzyl)-4-chloro-5-((trifluoromethyl)thio)-1*H*-imidazole (9l)

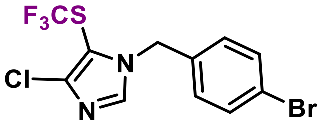

9g, 95%

Following **general procedure F**, using 4,5-dichloro-1-(4-bromobenzyl)-1*H*-imidazole[41] (**8l**) (91.8 mg, 0.30 mmol, 1.0 eq), the pure product **9l** (106.0 mg, 0.29 mmol, 95%) was obtained as a yellow solid after flash column chromatography (pentane:EtOAc 4:1 v/v).

TLC (EtOAc): **R_f** = 0.57; **^{1}H NMR** (400 MHz, $CDCl_3$): δ 7.58 (s, 1H), 7.51 (d, J = 8.4 Hz, 2H), 7.04 (d, J = 8.4 Hz, 2H), 5.16 (s, 2H).; **^{13}C NMR** (101 MHz, $CDCl_3$): δ 142.8, 140.0, 133.4, 132.6, 129.4, 127.93 (qd, *J* = 314.3), 123.1, 108.3, 49.7. **^{19}F NMR** (376 MHz, $CDCl_3$): δ −43.3; **HRMS (ESI)** (*m/z*): $[M + Na]^+$ calcd. for $C_{11}H_7F_3N_2ClBrSNa$ = 392.9046; found: 392.9046.

4-Chloro-1-((perfluorophenyl)methyl)-5-((trifluoromethyl)thio)-1*H*-imidazole (9m)

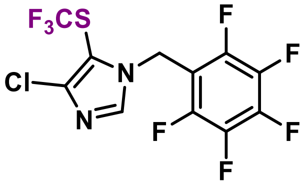

9k, 85%

Following **general procedure F**, using 4,5-dichloro-1-((perfluorophenyl)methyl)-1*H*-imidazole (**8m**) (95.1 mg, 0.30 mmol, 1.0 eq), the pure product **9m** (98.0 mg, 0.26 mmol, 85%) was obtained as a yellow oil after flash column chromatography (pentane:EtOAc 4:1 v/v).

TLC (EtOAc): **R_f** = 0.63; **^{1}H NMR** (500 MHz, $CDCl_3$): δ 7.70 (s, 1H), 5.31 (s, 2H); **^{13}C NMR {^{19}F}** (126 MHz, $CDCl_3$): δ 145.6, 143.0, 142.4, 140.3, 138.0, 129.2, 126.7, 108.3, 37.8; **^{19}F NMR** (470 MHz, $CDCl_3$): δ −43.4 (s), −141.0 (m), −150.3 (tt, *J* = 20.8, 2.8 Hz), −159.6 (m); **HRMS (ESI)** (*m/z*): $[M + Na]^+$ calcd. for $C_{11}H_3F_8N_2ClSNa$ = 404.9470; found: 404.9456.

4-Chloro-5-((trifluoromethyl)thio)-1-(4-((trifluoromethyl)thio)benzyl)-1*H*-imidazole (9n)

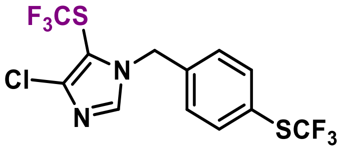

9l, 91%

Following **general procedure F**, using 4,5-dichloro-1-(4-((trifluoromethyl)thio)benzyl)-1*H*-imidazole (**8n**) (98.1 mg, 0.30 mmol, 1.0 eq), the pure product **9n** (107.7 mg, 0.27 mmol, 91%) was obtained as a pale yellow solid after flash column chromatography (pentane:EtOAc 4:1 to 2:1 v/v).

TLC (EtOAc): $\mathbf{R_f}$ = 0.63; ^{1}**H NMR** (400 MHz, $CDCl_3$): δ 7.73 – 7.59 (m, 3H), 7.19 (d, *J* = 8.0 Hz, 2H), 5.26 (s, 2H); ^{13}**C NMR** (101 MHz, $CDCl_3$): δ 142.9, 140.1, 137.6, 137.1, 129.5 (q, *J* = 308.1 Hz), 127.9 (q, *J* = 313.0 Hz), 128.4, 125.4, 108.5, 49.6; ^{19}**F NMR** (376 MHz, $CDCl_3$): δ −42.5, −43.3; **HRMS (ESI)** (*m/z*): $[M + Na]^+$ calcd. for $C_{12}H_7F_6N_2ClS_2Na$ = 414.9536; found: 414.9528.

***Tert*-butyl 4-((4-chloro-5-((trifluoromethyl)thio)-1*H*-imidazol-1-yl)methyl)benzoate (9o)**

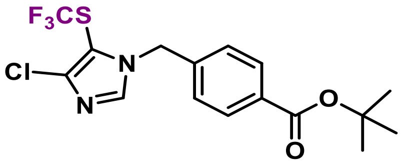

9n, 91%

Following **general procedure F**, using *tert*-butyl 4-((4,5-dichloro-1*H*-imidazol-1-yl)methyl)benzoate (**8o**) (98.2 mg, 0.30 mmol, 1.0 eq), the pure product **9o** (107.1 mg, 0.27 mmol, 91%) was obtained as a colourless solid after flash column chromatography (pentane:EtOAc 4:1 v/v).

TLC (EtOAc): $\mathbf{R_f}$ = 0.66; ^{1}**H NMR** (400 MHz, $CDCl_3$): δ 7.98 (d, *J* = 8.4 Hz, 2H), 7.60 (s, 1H), 7.17 (d, *J* = 8.0 Hz, 2H), 5.25 (s, 2H), 1.56 (s, 9H); ^{13}**C NMR** (101 MHz, $CDCl_3$): δ 164.9, 142.6, 140.0, 138.6, 132.5, 130.3, 127.8 (q, *J* = 313.3 Hz), 127.2, 108.3, 81.5, 49.8, 28.1; ^{19}**F NMR** (376 MHz, $CDCl_3$): δ −43.3; **HRMS (ESI)** (*m/z*): $[M + H]^+$ calcd. for $C_{16}H_{16}F_3N_2O_2ClSH$ = 393.0646; found: 393.0642.

4-Chloro-1-(3-methoxypropyl)-5-((trifluoromethyl)thio)-1*H*-imidazole (9p)

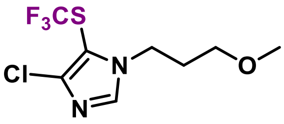

9m, 93%

Following **general procedure F**, using 4,5-dichloro-1-(3-methoxypropyl)-1*H*-imidazole (**8p**) (62.7 mg, 0.30 mmol, 1.0 eq), the pure product **9p** (76.5 mg, 0.28 mmol, 93%) was obtained as a colourless oil after flash column chromatography (pentane:EtOAc 1:1 v/v).

TLC (pentane:EtOAc, 1:1 v/v): **R$_f$** = 0.25; **^{1}H NMR** (400 MHz, $CDCl_3$): δ 7.63 (s, 1H), 4.16 (t, *J* = 7.0 Hz, 2H), 3.31 (s, 3H), 3.30 (t, *J* = 5.7 Hz, 2H), 1.99 (tt, *J* = 6.9, 5.6 Hz, 2H); **^{13}C NMR** (101 MHz, $CDCl_3$): δ 142.2, 140.2, 128.0 (q, *J* = 313.2 Hz), 107.60, 68.3, 58.8, 43.5, 30.3; **^{19}F NMR** (376 MHz, $CDCl_3$): δ −43.4; **HRMS (ESI)** (*m/z*): $[M + Na]^+$ calcd. for $C_8H_{10}F_3N_2OClSNa$ = 297.0047; found: 297.0051.

4-(4-Chloro-5-((trifluoromethyl)thio)-1*H*-imidazol-1-yl)butanenitrile (9q)

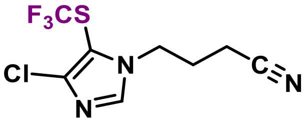

9r, quant.

Following **general procedure F**, using 4-(4,5-dichloro-1*H*-imidazol-1-yl)butanenitrile (**8q**) (61.2 mg, 0.30 mmol, 1.0 eq), the pure product **9q** (89.3 mg, 0.30 mmol, quant.) was obtained as a pale yellow oil after flash column chromatography (pentane:EtOAc 1:1 v/v to pure EtOAc).

TLC (pentane:EtOAc, 1:1 v/v): **R$_f$** = 0.15; **^{1}H NMR** (400 MHz, $CDCl_3$): δ 7.65 (s, 1H), 4.17 (t, *J* = 7.2 Hz, 2H), 2.34 (t, *J* = 6.9 Hz, 2H), 2.11 (p, *J* = 7.0 Hz, 2H); **^{13}C NMR** (101 MHz, $CDCl_3$): δ 142.8, 139.8, 127.7 (q, *J* = 312.4 Hz), 117.7, 107.7, 44.6, 26.1, 14.5; **^{19}F NMR** (376 MHz, $CDCl_3$): δ −43.2; **HRMS (ESI)** (*m/z*): $[M + Na]^+$ calcd. for $C_8H_7F_3N_3ClSNa$ = 291.9894; found: 291.9904.

2-(4-Chloro-5-((trifluoromethyl)thio)-1*H*-imidazol-1-yl)ethan-1-ol (9r)

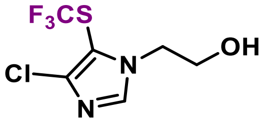

9p, quant.

Following **general procedure F**, using 2-(4,5-dichloro-1*H*-imidazol-1-yl)ethan-1-ol (**8r**) (54.3 mg, 0.30 mmol, 1.0 eq), the pure product **9r** (74.1 mg, 0.30 mmol, quant.) was obtained as a colourless solid after flash column chromatography (EtOAc:MeOH 99:1 v/v).

TLC (EtOAc): **R$_f$** = 0.29; **^{1}H NMR** (300 MHz, $CDCl_3$): δ 7.66 (s, 1H), 4.81 (s, 1H), 4.16 (m, 2H), 3.88 (m, 2H); **^{13}C NMR** (75 MHz, $CDCl_3$): δ 141.0, 140.7, 127.8 (q, *J* = 313.3 Hz), 107.9, 60.4, 49.6; **^{19}F NMR** (282 MHz, $CDCl_3$): δ −43.6; **HRMS (ESI)** (*m/z*): $[M + Na]^+$ calcd. for $C_6H_6F_3N_2OClSNa$ = 268.9734; found: 268.9737.

2-((4-Chloro-5-((trifluoromethyl)thio)-1*H*-imidazol-1-yl)methyl)pyridine (9s)

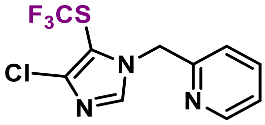

9s, 96%

Following a modified version of **general procedure F**, using 2-((4,5-dichloro-1*H*-imidazol-1-yl)methyl)pyridine (**8s**) (68.1 mg, 0.30 mmol, 1.0 eq) and NH_4Cl (8.1 mg, 0.3 mmol, 0.50 eq) as additive, the pure product **9s** (84.8 mg, 0.29 mmol, 96%) was obtained as a pale yellow oil after flash column chromatography (EtOAc:MeOH 199:1 v/v).

TLC (EtOAc:MeOH, 19:1 v/v): **R$_f$** = 0.41; **^{1}H NMR** (400 MHz, $CDCl_3$): δ 8.50 (m, 1H), 7.75 (s, 1H), 7.62 (td, *J* = 7.7, 1.8 Hz, 1H), 7.19 (dd, *J* = 7.6, 4.9 Hz, 1H), 7.04 (d, *J* = 7.8 Hz, 1H), 5.26 (s, 2H); **^{13}C NMR** (101 MHz, $CDCl_3$): δ 154.1, 150.1, 142.3, 140.6, 137.3, 127.9 (q, *J* = 313.4 Hz), 123.5, 121.8, 108.2, 51.4; **^{19}F NMR** (376 MHz, $CDCl_3$): δ −43.4; **HRMS (ESI)** (*m/z*): $[M + Na]^+$ calcd. for $C_{10}H_7F_3N_3ClSNa$ = 315.9894; found: 315.9888.

Diethyl (3-(4-chloro-5-((trifluoromethyl)thio)-1*H*-imidazol-1-yl)propyl)phosphonate (9t)

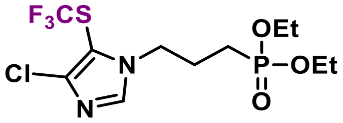

9t, 95%

Following **general procedure F**, using diethyl (3-(4,5-dichloro-1*H*-imidazol-1-yl)propyl)phosphonate (**8t**) (94.5 mg, 0.30 mmol, 1.0 eq), the pure product **9t** (108.2 mg, 0.28 mmol, 95%) was obtained as a yellow oil after flash column chromatography (EtOAc:MeOH 9:1 v/v).

TLC (EtOAc:MeOH, 9:1 v/v): **R_f** = 0.19; **^{1}H NMR** (400 MHz, $CDCl_3$): δ 7.69 (s, 1H), 4.14 (t, *J* = 7.2 Hz, 2H), 4.06 (dddd, *J* = 13.0, 8.1, 6.2, 4.4 Hz, 4H), 2.05 (m, 2H), 1.66 (ddd, *J* = 18.8, 8.1, 7.2 Hz, 2H), 1.28 (t, *J* = 7.2 Hz, 6H); **^{13}C NMR** (101 MHz, $CDCl_3$): δ 142.4, 140.0, 127.9 (q, *J* = 313.3 Hz), 107.6, 62.0 (d, *J* = 6.6 Hz), 46.2 (d, *J* = 14.4 Hz), 23.9 (d, *J* = 4.9 Hz), 22.5 (d, *J* = 143.6 Hz), 16.5 (d, *J* = 5.9 Hz); **^{19}F NMR** (376 MHz, $CDCl_3$): δ −43.4; **HRMS (ESI)** (*m/z*): $[M + Na]^+$ calcd. for $C_{11}H_{17}F_3O_3N_2ClPSNa$ = 403.0230; found: 403.0234.

3,4-Dichloro-1-methyl-2-phenyl-5-((trifluoromethyl)thio)-1*H*-pyrrole (9u)

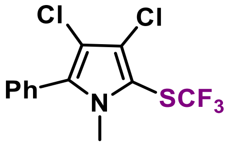

9u, 85%

Following **general procedure F**, using diethyl 2,3,4-trichloro-1-methyl-5-phenyl-1*H*-pyrrole (**8u**) (78.2 mg, 0.30 mmol, 1.0 eq), the pure product **9u** (66.3 mg, 0.26 mmol, 85%) was obtained as a pale yellow oil after flash column chromatography (pentane:CH_2Cl_2 49:1 v/v).

TLC (pentane:Et_2O, 9:1 v/v): **R_f** = 0.57; **^{1}H NMR** (300 MHz, CD_2Cl_2): δ 7.49 (dt, *J* = 5.9, 3.3 Hz, 3H), 7.39 (dt, *J* = 7.3, 3.3 Hz, 2H), 3.63 (s, 3H); **^{13}C NMR** (101 MHz, CD_2Cl_2): δ 136.7, 132.2, 130.8, 129.8, 129.6, 129.3, 128.6 (q, *J* = 313.4 Hz) 123.3, 110.8, 34.4; **^{19}F NMR** (282 MHz, $CDCl_3$): δ −43.9; **HRMS (APCI)** (*m/z*): $[M]^+$ calcd. for $C_{12}H_8F_3N_2SCl_2$ = 324.9701; found: 324.9700.

3,4-Dibromo-1-methyl-2-phenyl-5-((trifluoromethyl)thio)-1*H*-pyrrole (9v)

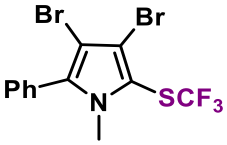

9v, 63%

Following a modified version of **general procedure F**, using 2-((4,5-dichloro-1*H*-imidazol-1-yl)methyl)pyridine (**8v**) (68.1 mg, 0.30 mmol, 1.0 eq) and *N*-(*tert*-butyl)-4-methoxy-*N*'-((trifluoromethyl)thio)benzimidamide (**3b**) (184 mg, 0.60 mmol, 2.0 eq), the pure product **9v** (78.5 mg, 0.19 mmol, 63%) was obtained after 48 h reaction time as a pale yellow oil after flash column chromatography (pentane:Et_2O 99:1 v/v).

TLC (pentane:Et_2O, 9:1 v/v): $\mathbf{R_f}$ = 0.38; **^{1}H NMR** (400 MHz, $CDCl_3$): δ 7.48 (m, 3H), 7.38 (m, 2H), 3.65 (s, 3H); **^{13}C NMR** (101 MHz, $CDCl_3$): δ 138.6, 130.5, 130.2, 129.4, 128.8, 128.3 (q, J = 312.6 Hz), 114.1, 111.0, 100.3, 34.7; **^{19}F NMR** (376 MHz, $CDCl_3$): δ −43.8; **HRMS (ESI)** (*m/z*): $[M + H]^+$ calcd. for $C_{12}H_8F_3Br_2NSH$ = 415.8749; found: 415.8748.

8.4 Mechanistic Studies on SCF_3-Amidines

8.4.1 Computational Investigation of the Reaction Pathway

Figure S35: Optimized geometries of different intermediates of the trifluoromethylthiolation of heteroarenes ((CAM-B3LYP/TZVPP)/CPCM (DMSO)).

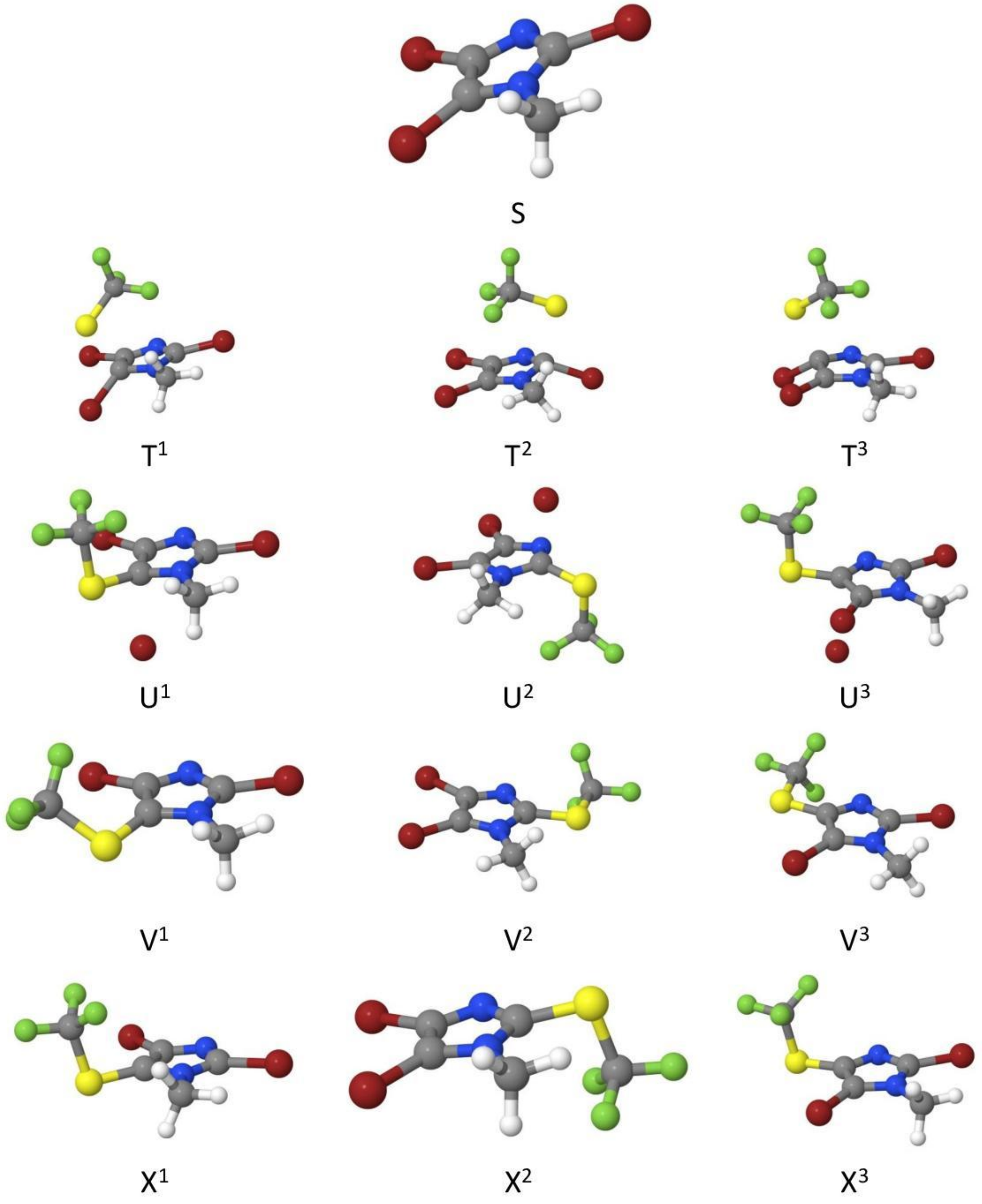

Table S65: Determination of the Gibbs Free Energy (in Hartree) at 298 K (CAM-B3LYP/TZVPP)/CPCM(DMSO) for the species involved in the presented mechanism.

	Electronic Energy	Thermal Corrections	ZPVE	Enthalpy Corrections	Entropy Corrections	Gibbs Free Energy
**S (8c)**	-7986.550408	0.008758	0.069788	0.000944	-0.046761	-7986.51768
**T^1**	-8722.431597	0.014904	0.084777	0.000944	-0.060236	-8722.39121
**T^3**	-8722.424533	0.014759	0.084988	0.000944	-0.059791	-8722.38363
**T^2**	-8722.427179	0.014853	0.084897	0.000944	-0.060104	-8722.38659
**U^1**	-8722.459306	0.014744	0.086036	0.000944	-0.060122	-8722.4177
**U^3**	-8722.455998	0.014914	0.085856	0.000944	-0.060603	-8722.41489
**U^2**	-8722.452230	0.014780	0.085875	0.000944	-0.060556	-8722.41119
**V^1**	-6147.963935	0.011683	0.084978	0.000944	-0.052877	-6147.91921
**V^3**	-6147.973000	0.011708	0.085200	0.000944	-0.053051	-6147.9282
**V^2**	-6147.971616	0.011720	0.085177	0.000944	-0.053008	-6147.92678
**X^1 (9c)**	-6148.202716	0.012467	0.085995	0.000944	-0.054166	-6148.15748
**X^3**	-6148.200872	0.012514	0.085884	0.000944	-0.054234	-6148.15576
**X^2**	-6148.199389	0.013269	0.086099	0.000944	-0.055937	-6148.15501
**Br^-**	-2574.140070	0.004249	0.000000	0.000944	-0.000000	-2574.13488
**Br•**	-2573.925801	0.004249	0.000000	0.000944	-0.000000	-2573.92061

To investigate the mechanism of the trifluoromethylthiolation of heteroarenes, various intermediates and transition states were calculated for 2,4,5-tribromo-1-methyl-imidazole (**S**) (**8c**) as model substrate using density functional theory ((CAM-B3LYP-D3/TZVPP)/CPCM (DMSO)). In a first step, the trifluoromethylthiyl radical (**N**) attacks the imidazole (**S**) (**8c**) either in the 2-, 4- or 5-position. For each of these possibilities, different transition states were evaluated, showing that endo-like transition states (**T^1**, **T^2**, **T^3**) are energetically favoured in all cases. Comparing the substitution in different positions, the attack at the electron-rich 5-position (**T^1**) is thermodynamically and kinetically favoured (Figure S36), while an attack in the 4-position has the highest energy barrier. These results are in accordance with the experimental evaluation of various mono- and polysubstituted imidazoles, whereby imidazoles bearing a halogen in the 5-position show the best, 2-halogeno-imidazoles show some, and 4-halogeno-imidazoles show no reactivity under the standard reaction conditions.

After the radical substitution, different product forming mechanisms are possible, in which either a bromide anion gets substituted, leaving a radical cation (**V**) or a bromine radical will get released, leaving the final trifluoromethylthiolated imidazole (**W**). Both, the radical cation (**V**) or the bromine radical, are able to oxidize the SCF_3-amidine **B** or the, in prior, reduced catalyst [Ir(dF(CF_3)ppy)$_2$(dtbpy)] to propagate a radical chain or to close the photocatalytic cycle. In case of the substitution in the 5-position, a direct reduction of the π-complex **U** is likely. This species (**U^1**) seems to be a slightly stabilized intermediate in which the spin density is mostly, but not fully localized on the dissociating bromine atom (Figure S36). For a substitution in 5-position of the imidazole, this species could get reduced without any further steps, forming the product (**X^1**) (**9c**) and a bromide anion. For substitution in other positions, the release of a bromine radical seems to be the thermodynamically favoured process. This radical would act as oxidant to propagate a radical chain or to close the catalytic cycle.

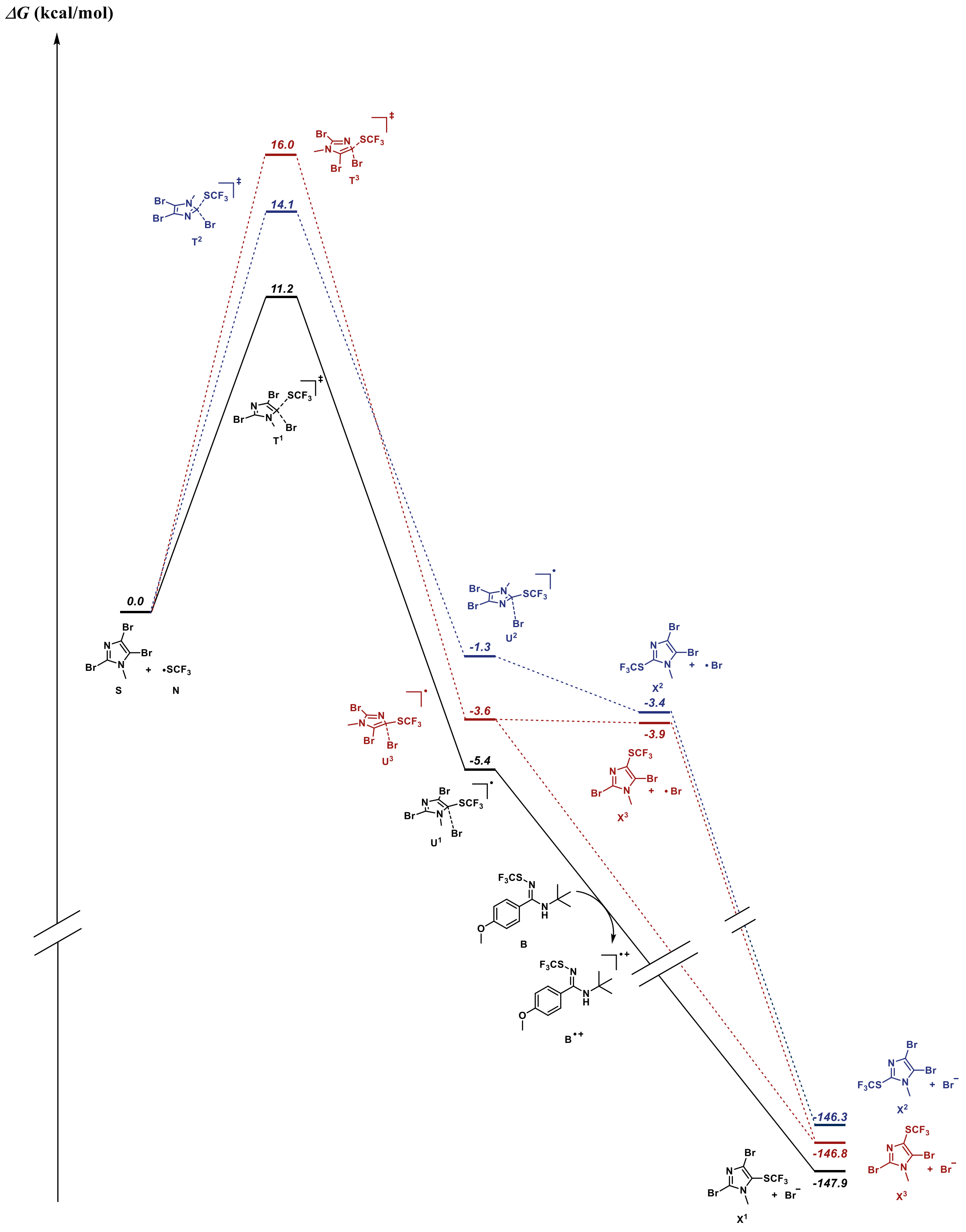

Figure S36: Proposed mechanism for the reaction of 2,4,5-tribromo-1-methyl-imidazole (**S**) (**8d**) and •SCF_3 (**N**) based on computational studies (CAM-B3LYP-D3/TZVPP)/CPCM (DMSO). Evaluation of pathways leading to different products.

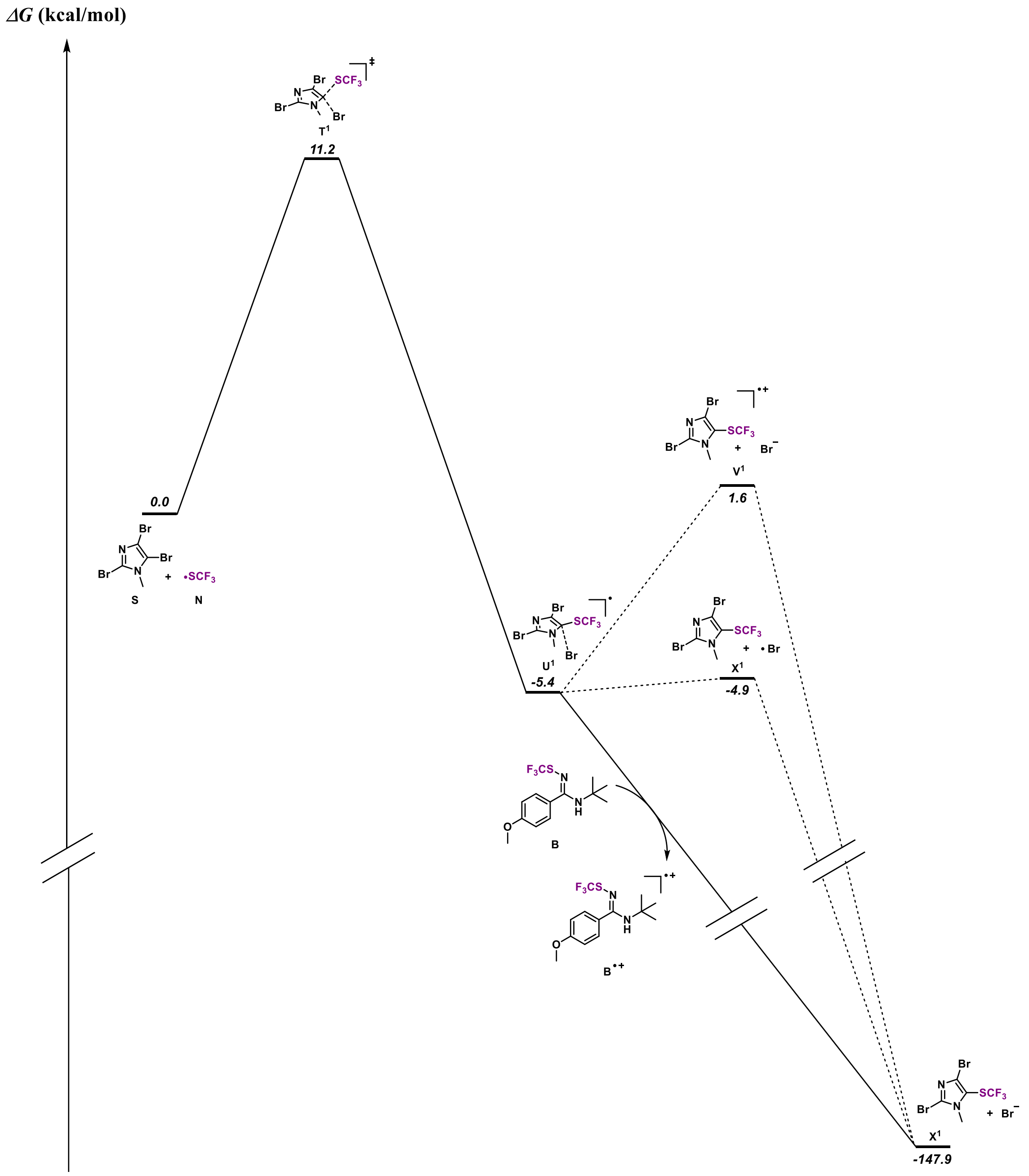

Figure S37: Proposed mechanism for the reaction of 2,4,5-tribromo-1-methyl-imidazole (**S**) (**8d**) and •SCF_3 (**N**) based on computational studies (CAM-B3LYP-D3/TZVPP)/CPCM (DMSO). Evaluation of different pathways for the substitution in 5-position.

9. Geometries Optimized Structures

A

C	-3.707863	0.217770	0.785008
C	-2.470234	0.836531	0.940995
C	-3.960275	-0.568752	-0.337608
C	-2.968372	-0.742912	-1.301297
C	-1.726431	-0.133784	-1.143066
C	-1.471630	0.666208	-0.023862
H	-4.477984	0.347722	1.548237
H	-2.275239	1.441556	1.828882
H	-0.950511	-0.269999	-1.899019
H	-4.932943	-1.050050	-0.460706
H	-3.162409	-1.356847	-2.183342
C	-0.141122	1.334393	0.134606
N	0.036759	2.589922	0.375597
N	0.924884	0.507268	-0.008081
S	-1.263408	3.686932	0.374288
C	2.351192	0.873755	0.013732
H	0.714531	-0.483188	-0.014568
C	2.661561	1.863115	-1.115890
H	2.404568	1.423727	-2.091643
H	3.735508	2.102147	-1.117645
H	2.095149	2.794137	-0.989235
C	3.121669	-0.426866	-0.219989
C	2.743780	1.468345	1.372378
H	2.848111	-0.876998	-1.186609
H	2.910854	-1.156978	0.577051
H	4.202838	-0.230813	-0.225607
H	2.198286	2.401979	1.558423
H	3.823588	1.679227	1.391795
H	2.517283	0.758604	2.182679
C	-0.204077	5.142139	0.321893
F	0.569364	5.191113	-0.765134
F	0.611039	5.235243	1.372819
F	-0.994266	6.216725	0.314618

A$^{\bullet+}$

C	-3.741627	0.328646	0.734736
C	-2.500198	0.942918	0.855437
C	-3.974805	-0.581562	-0.294924
C	-2.963030	-0.888865	-1.203289
C	-1.714652	-0.289222	-1.084135
C	-1.482014	0.634215	-0.056849
H	-4.529573	0.558844	1.453895
H	-2.317873	1.636109	1.678514

H	-0.928469	-0.519494	-1.806068
H	-4.953380	-1.056588	-0.389664
H	-3.147297	-1.596653	-2.013378
C	-0.152004	1.274757	0.062548
N	0.038359	2.598382	0.254193
N	0.917561	0.522363	-0.024174
S	-1.147324	3.661564	0.011399
C	2.361965	0.908331	-0.017765
H	0.736288	-0.479444	-0.090215
C	2.647211	1.889150	-1.157855
H	2.331892	1.469041	-2.123928
H	3.729985	2.071823	-1.200259
H	2.141224	2.849517	-1.002636
C	3.124342	-0.396524	-0.243922
C	2.722617	1.504563	1.346106
H	2.864643	-0.843741	-1.215070
H	2.907699	-1.123687	0.553035
H	4.203274	-0.194197	-0.237770
H	2.186886	2.443521	1.530352
H	3.802358	1.708773	1.365132
H	2.491684	0.795226	2.153892
C	-0.144321	5.143209	0.465569
F	0.865354	5.293799	-0.371997
F	0.332783	5.028675	1.688689
F	-0.945363	6.187918	0.390103

$A^{•-}$

C	-3.889234	0.505457	0.219679
C	-2.714573	1.066215	0.678205
C	-3.890806	-0.488008	-0.779945
C	-2.647440	-0.911453	-1.286578
C	-1.460235	-0.369302	-0.834165
C	-1.427748	0.669918	0.164375
H	-4.837820	0.831852	0.657832
H	-2.765403	1.792455	1.492072
H	-0.524590	-0.715538	-1.279476
H	-4.824809	-0.923439	-1.140679
H	-2.614574	-1.683460	-2.061900
C	-0.208855	1.226429	0.655184
N	-0.055256	2.380585	1.340707
N	0.945581	0.433954	0.570032
S	-1.111995	3.666925	1.016284
C	2.238052	0.905066	0.034997
H	0.738184	-0.510083	0.268919
C	2.045808	1.687312	-1.270571
H	1.546925	1.061983	-2.027340
H	3.015736	2.011339	-1.679469

H	1.432383	2.580225	-1.095522
C	3.069979	-0.350486	-0.238184
C	2.969432	1.774436	1.063737
H	2.583723	-0.991384	-0.991365
H	3.197863	-0.939252	0.683324
H	4.066747	-0.080606	-0.616009
H	2.383321	2.672929	1.288224
H	3.961820	2.066411	0.684818
H	3.107807	1.213933	2.001494
C	0.113976	4.942402	0.691749
F	0.800755	4.774947	-0.451540
F	1.038203	5.059187	1.654531
F	-0.518754	6.120421	0.596281

^{3}A

C	-3.871299	0.726932	0.197607
C	-2.624271	1.280159	0.440971
C	-3.997924	-0.491286	-0.479095
C	-2.840849	-1.147091	-0.911736
C	-1.585133	-0.608116	-0.676077
C	-1.434874	0.627452	0.011721
H	-4.764338	1.251913	0.545984
H	-2.550133	2.224289	0.981574
H	-0.708934	-1.137303	-1.055474
H	-4.983204	-0.921523	-0.669051
H	-2.920909	-2.094482	-1.450684
C	-0.148438	1.199757	0.265102
N	-0.089984	2.458688	0.907047
N	1.005684	0.453297	0.195945
S	-0.193733	3.775473	-0.074743
C	2.359554	0.902219	-0.185834
H	0.834275	-0.523017	-0.012221
C	2.350321	1.574036	-1.564826
H	1.940140	0.890945	-2.323684
H	3.372679	1.849234	-1.863795
H	1.745390	2.491639	-1.566292
C	3.213368	-0.366434	-0.245999
C	2.942468	1.841950	0.875918
H	2.824296	-1.066268	-1.002180
H	3.222044	-0.876249	0.728977
H	4.248658	-0.116366	-0.515721
H	2.372432	2.774437	0.967458
H	3.978294	2.102818	0.612962
H	2.947356	1.349082	1.859272
C	-0.214755	5.019831	1.259080
F	0.896153	4.981443	1.983929
F	-1.241490	4.838534	2.078436

F	-0.318643	6.215375	0.695129

B

C	-3.457406	-0.280540	0.599050
C	-2.288511	0.452473	0.791841
C	-3.593110	-1.079033	-0.541103
C	-2.543988	-1.138660	-1.470435
C	-1.383727	-0.412951	-1.261849
C	-1.240790	0.402714	-0.129067
H	-4.249287	-0.224774	1.344556
H	-2.194962	1.059038	1.695085
H	-0.577088	-0.467504	-1.995559
O	-4.683199	-1.817628	-0.822383
H	-2.666226	-1.765095	-2.355765
C	0.007893	1.194612	0.083531
N	0.056617	2.447487	0.394615
N	1.158146	0.492665	-0.086841
S	-1.343690	3.412636	0.410540
C	2.537253	1.006033	-0.034662
H	1.055530	-0.514179	-0.118671
C	2.745252	2.078174	-1.110934
H	2.537681	1.664207	-2.109312
H	3.788450	2.427370	-1.092684
H	2.084643	2.937610	-0.942359
C	3.441595	-0.192528	-0.327621
C	2.862805	1.569055	1.354931
H	3.219985	-0.618908	-1.318015
H	3.306932	-0.980859	0.429532
H	4.496124	0.116082	-0.314175
H	2.221322	2.429449	1.582858
H	3.914481	1.890702	1.393311
H	2.710258	0.799472	2.127070
C	-0.431996	4.965176	0.430039
F	0.347660	5.131995	-0.641238
F	0.356228	5.097501	1.497344
F	-1.323423	5.957477	0.449013
C	-5.778890	-1.799216	0.070161
H	-6.542029	-2.459362	-0.358556
H	-5.493451	-2.175385	1.065678
H	-6.195423	-0.784488	0.174584

B$^{\bullet+}$

C	-3.504179	-0.039263	0.443215
C	-2.329287	0.690783	0.538909
C	-3.563013	-1.150301	-0.410237
C	-2.426720	-1.517506	-1.154439
C	-1.258323	-0.794802	-1.043575

C	-1.192606	0.329177	-0.197911
H	-4.364480	0.258538	1.039887
H	-2.294520	1.537050	1.226038
H	-0.397035	-1.085107	-1.648290
O	-4.647708	-1.909060	-0.577131
H	-2.496510	-2.375808	-1.823927
C	0.056362	1.088203	-0.059660
N	0.059317	2.408590	0.262812
N	1.209146	0.480334	-0.175890
S	-0.938245	3.464779	-0.435433
C	2.590850	1.048718	-0.138671
H	1.160816	-0.533011	-0.266710
C	2.729598	2.188172	-1.149665
H	2.411368	1.867141	-2.152126
H	3.786448	2.484368	-1.203104
H	2.150591	3.071845	-0.854358
C	3.509263	-0.108541	-0.528041
C	2.899651	1.522107	1.283972
H	3.292521	-0.458904	-1.548179
H	3.398579	-0.952783	0.169075
H	4.554388	0.225907	-0.494469
H	2.234860	2.341246	1.587160
H	3.935924	1.886779	1.321952
H	2.797334	0.694851	2.000940
C	-0.604710	4.841397	0.744510
F	0.681783	5.136017	0.765307
F	-0.983504	4.506923	1.963303
F	-1.296682	5.884235	0.329835
C	-5.837205	-1.601356	0.130644
H	-6.577852	-2.350383	-0.170889
H	-5.678836	-1.662038	1.218351
H	-6.205724	-0.597480	-0.130694

C

C	-3.444622	-0.346917	0.605835
C	-2.259648	0.362956	0.791718
C	-3.616039	-1.065055	-0.579195
C	-2.607776	-1.072608	-1.554249
C	-1.428342	-0.365330	-1.349482
C	-1.242057	0.371415	-0.157509
H	-4.209578	-0.332158	1.379699
H	-2.122756	0.921267	1.720614
O	-0.419501	-0.329644	-2.242409
O	-4.716263	-1.782437	-0.876154
H	-2.784196	-1.642473	-2.464532
C	0.006267	1.159859	0.081444
N	0.034945	2.431190	0.302755

N	1.155776	0.437061	0.106654
S	-1.390254	3.356562	0.199748
C	2.530238	0.966995	0.085570
H	1.054584	-0.522674	-0.199140
C	2.742527	1.874512	-1.133441
H	2.518618	1.325603	-2.060683
H	3.788306	2.214298	-1.174731
H	2.089512	2.755185	-1.082328
C	3.447128	-0.253367	-0.015199
C	2.833224	1.727497	1.381612
H	3.249604	-0.819045	-0.939430
H	3.298226	-0.926341	0.843052
H	4.499735	0.061889	-0.027842
H	2.178442	2.601957	1.480278
H	3.880592	2.065100	1.380444
H	2.682807	1.073618	2.254145
C	-0.518766	4.931336	0.204522
F	0.313403	5.074888	-0.829727
F	0.207200	5.127935	1.305961
F	-1.432955	5.900794	0.134524
C	-5.783122	-1.823898	0.049881
H	-6.560649	-2.454054	-0.397736
H	-5.466840	-2.265700	1.008309
H	-6.193946	-0.818538	0.235089
C	-0.543332	-1.038512	-3.459038
H	0.386134	-0.867440	-4.014281
H	-1.394492	-0.668989	-4.052505
H	-0.667739	-2.118619	-3.282915

$C^{•+}$

C	-3.592440	0.122332	0.218786
C	-2.389264	0.789526	0.331500
C	-3.662582	-1.011244	-0.606257
C	-2.519123	-1.464789	-1.278568
C	-1.310564	-0.799722	-1.144813
C	-1.225939	0.380136	-0.346077
H	-4.458202	0.478760	0.772465
H	-2.344850	1.652093	0.994662
O	-0.188416	-1.219811	-1.749789
O	-4.770053	-1.725166	-0.799449
H	-2.612933	-2.355269	-1.895976
C	0.009041	1.142619	-0.173929
N	-0.021312	2.374317	0.428832
N	1.177416	0.650531	-0.492738
S	-0.834339	3.619689	-0.202747
C	2.538863	1.245846	-0.359625
H	1.131206	-0.288865	-0.895040

C	2.635316	2.566060	-1.127595
H	2.287623	2.446506	-2.163927
H	3.686919	2.884220	-1.152477
H	2.060240	3.366025	-0.646003
C	3.479447	0.214321	-0.983139
C	2.870842	1.438898	1.122114
H	3.248426	0.063509	-2.048469
H	3.404813	-0.752060	-0.462135
H	4.516677	0.565491	-0.904816
H	2.198760	2.167146	1.594064
H	3.900715	1.812722	1.211744
H	2.800123	0.484983	1.664284
C	-0.601099	4.743982	1.234914
F	0.683506	4.933125	1.479563
F	-1.166798	4.241677	2.317378
F	-1.170270	5.893305	0.926190
C	-5.976373	-1.345116	-0.155420
H	-6.733835	-2.071638	-0.469587
H	-5.868320	-1.379886	0.939166
H	-6.287708	-0.335514	-0.463333
C	-0.209988	-2.361054	-2.599700
H	0.811732	-2.477226	-2.976325
H	-0.899832	-2.202936	-3.440833
H	-0.503035	-3.259404	-2.037567

D

C	-3.399707	-0.410498	0.651486
C	-2.205138	0.294947	0.841981
C	-3.635147	-1.007239	-0.588811
C	-2.703907	-0.910832	-1.630255
C	-1.522355	-0.205279	-1.422044
C	-1.259825	0.411181	-0.184864
H	-4.122982	-0.494013	1.456244
O	-1.883738	0.895964	2.003322
O	-0.570558	-0.052744	-2.362905
O	-4.751383	-1.706247	-0.871879
H	-2.936233	-1.391987	-2.577588
C	0.000136	1.191077	0.018015
N	0.059548	2.479279	0.027720
N	1.116869	0.444753	0.204178
S	-1.373587	3.361715	-0.227987
C	2.506468	0.926973	0.247359
H	0.990157	-0.552118	0.083604
C	2.859670	1.671233	-1.047113
H	2.699277	1.018132	-1.918250
H	3.916541	1.977300	-1.031376
H	2.235425	2.566545	-1.162512

C	3.382224	-0.319894	0.380193
C	2.718995	1.834937	1.464554
H	3.246282	-0.989748	-0.483402
H	3.132958	-0.877771	1.295904
H	4.442795	-0.036109	0.427786
H	2.081137	2.725176	1.399192
H	3.770935	2.153908	1.515976
H	2.475835	1.294781	2.392201
C	-0.538764	4.953252	-0.276728
F	0.357433	5.047870	-1.261792
F	0.108754	5.243786	0.853382
F	-1.465432	5.893825	-0.472822
C	-5.750537	-1.853188	0.116339
H	-6.551823	-2.448660	-0.336865
H	-5.365502	-2.381594	1.003064
H	-6.156912	-0.877194	0.426306
C	-2.804561	0.889244	3.075203
C	-0.757231	-0.632470	-3.638109
H	0.132869	-0.381925	-4.226890
H	-1.649729	-0.223788	-4.137826
H	-0.851259	-1.727831	-3.571093
H	-2.336478	1.454647	3.889454
H	-3.012963	-0.135470	3.421230
H	-3.751266	1.376582	2.793537

D$^{•+}$

C	-3.507197	-0.277657	0.569313
C	-2.313173	0.412363	0.771211
C	-3.640720	-1.073024	-0.573126
C	-2.595683	-1.199848	-1.499923
C	-1.407330	-0.519264	-1.287459
C	-1.255968	0.331548	-0.161317
H	-4.309007	-0.207435	1.297038
O	-2.075046	1.165784	1.853243
O	-0.351465	-0.600539	-2.110635
O	-4.747286	-1.767547	-0.856275
H	-2.749476	-1.839843	-2.365276
C	-0.017723	1.099950	0.039004
N	0.023167	2.432553	0.283973
N	1.140492	0.488454	-0.043833
S	-1.257480	3.357321	-0.031155
C	2.524975	1.046078	-0.024071
H	1.080403	-0.514285	-0.215307
C	2.688936	2.079540	-1.142331
H	2.409392	1.649078	-2.114930
H	3.743776	2.383931	-1.190526
H	2.079813	2.972920	-0.959673

C	3.439343	-0.151795	-0.280177
C	2.816248	1.650571	1.352283
H	3.233521	-0.604188	-1.262158
H	3.312619	-0.918891	0.498437
H	4.486557	0.177376	-0.269442
H	2.166134	2.508784	1.560320
H	3.861594	1.989573	1.374275
H	2.680739	0.898600	2.142999
C	-0.477695	4.931149	0.521877
F	0.546159	5.246030	-0.252769
F	-0.049669	4.833441	1.766055
F	-1.400260	5.871741	0.437901
C	-5.862555	-1.704615	0.013788
H	-6.637297	-2.338801	-0.431690
H	-5.609973	-2.088095	1.014461
H	-6.241309	-0.674705	0.102039
C	-3.083331	1.344548	2.833221
C	-0.439760	-1.379863	-3.291437
H	0.527093	-1.280704	-3.797089
H	-1.238942	-1.007670	-3.949939
H	-0.622666	-2.438604	-3.053835
H	-2.659037	2.014610	3.589140
H	-3.351060	0.386287	3.303244
H	-3.982564	1.805648	2.397668

E

C	-3.482423	-0.171809	0.895268
C	-2.232649	0.442019	1.007944
C	-3.961607	-0.586150	-0.343807
C	-3.199111	-0.390878	-1.491395
C	-1.944439	0.219223	-1.419301
C	-1.473160	0.628686	-0.160018
H	-4.084811	-0.325690	1.793678
C	-1.714344	0.904407	2.344090
C	-1.120427	0.443930	-2.659621
H	-4.940389	-1.065943	-0.416049
H	-3.579793	-0.716418	-2.462384
C	-0.132774	1.298444	-0.061587
N	0.043261	2.572732	-0.148773
N	0.915261	0.460884	0.129705
S	-1.321307	3.566146	-0.389709
C	2.335468	0.824571	0.271134
H	0.685229	-0.523094	0.192809
C	2.838170	1.523979	-0.997976
H	2.710938	0.869955	-1.874030
H	3.908034	1.760433	-0.897193
H	2.286076	2.456326	-1.171464

C	3.092079	-0.489593	0.468525
C	2.538289	1.725999	1.495392
H	2.954121	-1.156240	-0.396803
H	2.741741	-1.013015	1.371633
H	4.167383	-0.293860	0.581881
H	1.975296	2.661652	1.386184
H	3.605580	1.967526	1.610639
H	2.200235	1.215262	2.409915
C	-0.370026	5.093783	-0.378924
F	0.541745	5.149593	-1.350482
F	0.280688	5.296132	0.767473
F	-1.222672	6.104606	-0.554041
H	-0.732770	0.459709	2.565539
H	-2.406649	0.633730	3.152089
H	-1.582224	1.997311	2.357246
H	-1.616991	0.025892	-3.545104
H	-0.127822	-0.021329	-2.567259
H	-0.955924	1.518448	-2.833833

$E^{\bullet+}$

C	-3.521075	-0.139542	0.888825
C	-2.257141	0.438017	1.013662
C	-3.997309	-0.547254	-0.353355
C	-3.220156	-0.388571	-1.496376
C	-1.949349	0.183015	-1.421479
C	-1.490154	0.591565	-0.155973
H	-4.136999	-0.269474	1.781009
C	-1.727905	0.867151	2.356067
C	-1.096078	0.342910	-2.651472
H	-4.989866	-0.995897	-0.431778
H	-3.601369	-0.712281	-2.467026
C	-0.149853	1.236996	-0.053393
N	0.032448	2.570312	-0.134416
N	0.909565	0.488997	0.128967
S	-1.251063	3.515738	-0.398622
C	2.349123	0.880591	0.259109
H	0.718612	-0.512495	0.193823
C	2.805789	1.587251	-1.020292
H	2.633943	0.952646	-1.901855
H	3.884676	1.781176	-0.944601
H	2.289983	2.544835	-1.160194
C	3.107339	-0.433306	0.439337
C	2.525007	1.771167	1.492380
H	2.972304	-1.092017	-0.431448
H	2.773108	-0.963349	1.343597
H	4.179351	-0.221967	0.544324
H	1.999398	2.727048	1.379600

H	3.596455	1.976585	1.623497
H	2.160845	1.263650	2.397437
C	-0.301443	5.099241	-0.344253
F	0.612507	5.124582	-1.294190
F	0.293262	5.239777	0.824113
F	-1.168034	6.075016	-0.527882
H	-0.842788	0.278702	2.642843
H	-2.487339	0.731418	3.136151
H	-1.430794	1.927004	2.360294
H	-1.653779	0.057569	-3.552171
H	-0.197517	-0.290993	-2.598791
H	-0.756426	1.381547	-2.784080

F

C	-3.460716	-0.025998	0.780823
C	-2.224781	0.593403	0.911198
C	-3.827034	-0.569071	-0.445354
C	-2.963480	-0.488721	-1.531518
C	-1.731727	0.134663	-1.379374
C	-1.343480	0.687102	-0.162393
F	-4.284942	-0.109259	1.813777
F	-1.888198	1.100392	2.089865
F	-0.922366	0.205399	-2.428363
F	-5.000357	-1.160696	-0.579154
F	-3.318490	-1.003797	-2.698549
C	-0.017199	1.374595	-0.018960
N	0.114803	2.649139	0.080080
N	1.046204	0.541422	-0.014886
S	-1.245119	3.664734	0.014492
C	2.468095	0.922226	0.062883
H	0.838427	-0.449328	-0.041112
C	2.845191	1.814620	-1.125670
H	2.626781	1.301562	-2.074356
H	3.921252	2.040843	-1.094010
H	2.287624	2.759332	-1.100185
C	3.256320	-0.386268	-0.004507
C	2.759106	1.635776	1.388718
H	3.056982	-0.916851	-0.948246
H	4.334067	-0.180724	0.052728
H	2.183223	2.566849	1.465973
H	3.830127	1.878290	1.455388
H	2.498348	0.987683	2.239064
C	-0.290313	5.177937	0.226997
F	0.595867	5.373434	-0.748693
F	0.387557	5.207517	1.373342
F	-1.147448	6.197767	0.222224
H	2.988688	-1.049182	0.832847

F$^{•+}$

C	-3.582178	0.129055	0.692614
C	-2.346492	0.739361	0.843682
C	-3.848406	-0.595354	-0.465514
C	-2.886772	-0.709067	-1.464445
C	-1.654127	-0.095558	-1.296397
C	-1.367900	0.642948	-0.147019
F	-4.497030	0.222731	1.638824
F	-2.092626	1.419109	1.951265
F	-0.743144	-0.210765	-2.249053
F	-5.018273	-1.176658	-0.616575
F	-3.150198	-1.395025	-2.560496
C	-0.050156	1.312187	0.008585
N	0.100394	2.636360	0.154248
N	1.026208	0.566092	-0.019125
S	-1.144673	3.646310	0.019272
C	2.467019	0.970384	0.027367
H	0.858494	-0.439758	-0.075762
C	2.775045	1.902153	-1.147949
H	2.493409	1.434953	-2.102649
H	3.856774	2.093447	-1.165377
H	2.256000	2.863147	-1.051333
C	3.248200	-0.334894	-0.111005
C	2.764228	1.632860	1.375409
H	3.026285	-0.832582	-1.066800
H	4.324076	-0.120283	-0.079800
H	2.218036	2.576509	1.492106
H	3.841075	1.845135	1.427533
H	2.504822	0.961246	2.206267
C	-0.168454	5.179968	0.362308
F	0.744951	5.358695	-0.570227
F	0.425078	5.094297	1.533925
F	-1.022073	6.182846	0.357388
H	3.015419	-1.025058	0.713802

G

C	-0.193400	1.825656	0.093828
N	0.164344	3.044161	0.317264
N	0.755675	0.874367	-0.093606
S	-0.991345	4.270938	0.553360
C	2.217732	1.047635	-0.090390
H	0.420081	-0.064807	-0.263236
C	2.649906	2.011358	-1.202908
H	2.315333	1.639916	-2.183535
H	3.746983	2.094725	-1.222143
H	2.223318	3.008985	-1.039672

C	2.807445	-0.338349	-0.356882
C	2.698580	1.555674	1.274832
H	2.471149	-0.728016	-1.330341
H	2.506242	-1.050901	0.426686
H	3.905045	-0.287884	-0.369797
H	2.274646	2.544034	1.492252
H	3.796320	1.632032	1.281612
H	2.396740	0.858700	2.071490
C	0.240008	5.564949	0.768331
F	1.020783	5.729525	-0.300967
F	1.053474	5.349099	1.803456
F	-0.409028	6.710092	0.985307
C	-1.631056	1.326079	0.011231
C	-1.927006	0.277356	1.086347
C	-1.962100	0.815687	-1.393833
H	-2.279631	2.192449	0.209443
H	-1.379212	-0.083862	-1.644351
H	-1.754718	1.579965	-2.157163
H	-3.027504	0.549629	-1.456302
H	-1.355243	-0.648350	0.918988
H	-2.994647	0.013611	1.069008
H	-1.683225	0.652437	2.091112

$G^{\bullet+}$

C	-0.218640	1.775519	0.080862
N	0.158485	3.048262	0.306427
N	0.742288	0.901600	-0.091580
S	-0.920796	4.220591	0.541851
C	2.227696	1.083870	-0.083263
H	0.433378	-0.057681	-0.254063
C	2.635069	2.047015	-1.201632
H	2.270374	1.690516	-2.175794
H	3.732379	2.092284	-1.241484
H	2.251211	3.058445	-1.023294
C	2.801819	-0.306899	-0.349576
C	2.676354	1.587856	1.291143
H	2.471894	-0.691640	-1.326260
H	2.499650	-1.016985	0.434630
H	3.898226	-0.253115	-0.357226
H	2.290147	2.593128	1.497641
H	3.774534	1.625573	1.308599
H	2.343173	0.904325	2.085405
C	0.328064	5.561810	0.763348
F	1.074271	5.687083	-0.317182
F	1.111324	5.303231	1.792879
F	-0.340686	6.677724	0.977987
C	-1.653304	1.298079	0.007297

C	-1.939217	0.247567	1.084518
C	-1.982231	0.795188	-1.402533
H	-2.291377	2.170786	0.210850
H	-1.403680	-0.107341	-1.651956
H	-1.776564	1.561468	-2.163140
H	-3.048268	0.534992	-1.453526
H	-1.364150	-0.674883	0.911642
H	-3.005434	-0.015315	1.057803
H	-1.700298	0.623841	2.089049

H

C	-3.033257	0.147089	0.786329
C	-1.810640	0.787797	0.968364
C	-3.262345	-0.610141	-0.361108
C	-2.262842	-0.733252	-1.324740
C	-1.035414	-0.102099	-1.142252
C	-0.805123	0.667831	0.003078
H	-3.809726	0.236502	1.548825
H	-1.632855	1.369816	1.874972
H	-0.254734	-0.198255	-1.899900
H	-4.223366	-1.109025	-0.503675
H	-2.439350	-1.324150	-2.225864
C	0.507440	1.361717	0.189398
N	0.659204	2.618213	0.416062
N	1.588135	0.526596	0.090510
S	-0.677516	3.670656	0.419265
H	1.354683	-0.457050	0.020115
C	0.327175	5.168266	0.433074
F	1.128861	5.268914	-0.626928
F	1.105113	5.264282	1.510320
F	-0.506255	6.207747	0.426724
C	2.963000	0.795669	0.127726
C	3.516827	2.080367	0.211509
C	4.903447	2.233754	0.226369
C	5.753834	1.134339	0.163539
C	5.200649	-0.144641	0.082746
C	3.823090	-0.313726	0.065272
H	2.861980	2.944773	0.266111
H	5.848659	-1.022678	0.032837
H	3.395707	-1.317783	0.000981
H	5.320366	3.241789	0.290333
H	6.837233	1.269034	0.177845

$H^{\bullet +}$

C	-3.091174	0.207164	0.682213
C	-1.876301	0.854298	0.880012
C	-3.262752	-0.641589	-0.410388

C	-2.215222	-0.852541	-1.304971
C	-0.994155	-0.215405	-1.110618
C	-0.822913	0.645307	-0.019297
H	-3.906599	0.361912	1.391055
H	-1.743209	1.499350	1.750499
H	-0.178661	-0.372353	-1.820087
H	-4.219773	-1.144380	-0.564149
H	-2.349232	-1.514170	-2.162688
C	0.477877	1.332382	0.176499
N	0.649224	2.615723	0.399290
N	1.579194	0.551586	0.110937
S	-0.581791	3.689240	0.333307
H	1.370209	-0.447858	0.076412
C	0.454405	5.156710	0.702719
F	1.451659	5.271143	-0.160952
F	0.966548	5.094936	1.918617
F	-0.332902	6.214264	0.608769
C	2.933784	0.835837	0.089042
C	3.477118	2.129663	-0.055917
C	4.853330	2.281502	-0.058860
C	5.698137	1.174616	0.073445
C	5.159735	-0.109230	0.201708
C	3.788641	-0.283001	0.204279
H	2.826400	2.990338	-0.174279
H	5.816138	-0.975348	0.297832
H	3.354378	-1.280360	0.303887
H	5.279011	3.279427	-0.176125
H	6.780876	1.313186	0.065647

I

C	-3.732595	0.296461	0.761802
C	-2.447652	0.759525	1.036044
C	-4.036025	-0.218292	-0.497053
C	-3.049031	-0.278020	-1.480602
C	-1.761140	0.171564	-1.204831
C	-1.453950	0.698391	0.054088
H	-4.500162	0.336884	1.537551
H	-2.212200	1.158186	2.025199
H	-0.983172	0.116956	-1.969290
H	-5.044961	-0.576935	-0.712348
H	-3.283769	-0.679806	-2.468629
C	-0.082359	1.243795	0.317078
N	0.123355	2.467211	0.672021
N	0.974435	0.392548	0.107178
S	-1.144171	3.603225	0.677303
C	2.372782	0.897687	-0.045045
C	0.810041	-1.011173	0.472523

C	2.424822	2.011640	-1.099725
H	2.016049	1.649147	-2.055302
H	3.473586	2.297690	-1.266174
H	1.868031	2.901543	-0.792600
C	3.262231	-0.242358	-0.561886
C	2.923855	1.390749	1.300432
H	2.870967	-0.661666	-1.500499
H	3.377663	-1.056574	0.165342
H	4.263470	0.162221	-0.763961
H	2.353242	2.256355	1.658228
H	3.979812	1.681041	1.191580
H	2.871609	0.593463	2.058314
C	-0.053540	5.028219	0.833579
F	0.770336	5.179078	-0.207351
F	0.713924	4.984864	1.922257
F	-0.821520	6.115782	0.909351
H	1.431700	-1.265135	1.346418
H	1.078501	-1.687129	-0.350438
H	-0.230005	-1.209438	0.746699

I$^{\bullet+}$

C	-3.601757	0.158838	1.011579
C	-2.284184	0.586245	1.149648
C	-4.099408	-0.177678	-0.245589
C	-3.280595	-0.091663	-1.370893
C	-1.959847	0.325355	-1.242915
C	-1.461662	0.661103	0.020564
H	-4.241984	0.089744	1.892931
H	-1.892131	0.848362	2.134775
H	-1.316518	0.391851	-2.122986
H	-5.134556	-0.508944	-0.350284
H	-3.671863	-0.351589	-2.356208
C	-0.071505	1.186623	0.151141
N	0.143054	2.496480	0.352973
N	0.986723	0.393390	0.056953
S	-1.104205	3.526109	0.423067
C	2.425382	0.915102	0.049598
C	0.788201	-1.053636	-0.015901
C	2.594310	1.912549	-1.103716
H	2.306072	1.450081	-2.059110
H	3.656642	2.184595	-1.167252
H	2.012978	2.827469	-0.957540
C	3.399073	-0.242874	-0.190513
C	2.735232	1.533060	1.418228
H	3.221515	-0.751744	-1.146755
H	3.394010	-0.981744	0.620363
H	4.406522	0.190620	-0.229630

H	2.140543	2.431876	1.609900
H	3.799515	1.805229	1.440580
H	2.555247	0.803277	2.221242
C	-0.067802	5.025682	0.681712
F	0.744096	5.222595	-0.342096
F	0.658855	4.916176	1.779048
F	-0.893703	6.049065	0.795340
H	1.393472	-1.534215	0.760167
H	1.105610	-1.427227	-0.997567
H	-0.261173	-1.302460	0.146398

J

C	-3.618171	0.045091	0.824415
C	-2.302143	0.439833	1.057394
C	-4.076337	-0.124370	-0.480235
C	-3.214685	0.097723	-1.554416
C	-1.896880	0.481796	-1.324588
C	-1.432823	0.652718	-0.016028
H	-4.288926	-0.129008	1.668456
H	-1.947010	0.578152	2.081183
H	-1.218762	0.648258	-2.164714
H	-5.109043	-0.429879	-0.662011
H	-3.571471	-0.030417	-2.578640
C	-0.037097	1.152110	0.223921
N	0.176763	2.342888	0.673011
N	1.008562	0.322191	-0.094743
S	-1.116907	3.433589	0.863591
C	2.411500	0.849030	-0.168039
C	0.774212	-1.125109	-0.024291
C	2.466950	2.021629	-1.160074
H	2.097865	1.701498	-2.146265
H	3.510733	2.348159	-1.276285
H	1.873858	2.875920	-0.819166
C	3.352803	-0.233991	-0.709604
C	2.927179	1.288755	1.212317
H	3.042433	-0.587190	-1.703643
H	3.441146	-1.098479	-0.037845
H	4.354643	0.205528	-0.809762
H	2.317916	2.104763	1.616437
H	3.967656	1.635923	1.121929
H	2.913124	0.449038	1.922187
C	-0.050415	4.858216	1.137944
F	0.734315	5.136684	0.093525
F	0.754563	4.717581	2.191445
F	-0.835606	5.914339	1.354312
C	0.943917	-1.730565	1.364738
H	1.432010	-1.628625	-0.739651

H	-0.243702	-1.323562	-0.376225
H	1.973788	-1.631248	1.736399
H	0.697164	-2.802953	1.337737
H	0.272879	-1.246656	2.091163

J•+

C	-3.466565	-0.102813	1.042968
C	-2.136452	0.297073	1.142429
C	-4.089800	-0.155769	-0.201704
C	-3.386681	0.194734	-1.353513
C	-2.056789	0.593413	-1.266010
C	-1.429217	0.634105	-0.015746
H	-4.017722	-0.371521	1.945970
H	-1.651200	0.347771	2.119374
H	-1.504718	0.869159	-2.167217
H	-5.133332	-0.468539	-0.275281
H	-3.876230	0.159434	-2.328487
C	-0.029431	1.147012	0.087769
N	0.188149	2.441786	0.368377
N	1.022590	0.361076	-0.096590
S	-1.067587	3.439393	0.588672
C	2.462072	0.884344	-0.058592
C	0.784191	-1.085613	-0.286107
C	2.630108	1.976139	-1.124460
H	2.320903	1.601217	-2.110953
H	3.696277	2.234871	-1.180897
H	2.066115	2.883907	-0.892303
C	3.445807	-0.239384	-0.396698
C	2.771999	1.392882	1.355603
H	3.324894	-0.607194	-1.424132
H	3.395243	-1.083580	0.301497
H	4.453992	0.187384	-0.319067
H	2.152869	2.252733	1.630565
H	3.826453	1.699801	1.386699
H	2.629945	0.594719	2.098326
C	-0.041001	4.935761	0.901932
F	0.721762	5.213263	-0.140443
F	0.734563	4.765153	1.957082
F	-0.875327	5.934989	1.119816
C	0.817627	-1.861677	1.024375
H	1.534199	-1.459565	-0.986224
H	-0.189122	-1.206155	-0.768739
H	1.787298	-1.780751	1.532928
H	0.636766	-2.922729	0.802252
H	0.033352	-1.517471	1.712406

K

C	-2.606847	-0.373851	0.810855
C	-1.338067	0.198729	0.816232
C	-3.415050	-0.274379	-0.321413
C	-2.953552	0.403638	-1.447430
C	-1.686236	0.983317	-1.443602
C	-0.870191	0.877000	-0.313854
H	-2.967091	-0.901285	1.696701
H	-0.704346	0.119599	1.702531
H	-1.327591	1.519888	-2.325055
H	-4.409269	-0.726455	-0.324497
H	-3.583294	0.484583	-2.335940
C	0.464931	1.556620	-0.288477
N	0.593123	2.838305	-0.158709
N	1.586995	0.794808	-0.389063
S	-0.794660	3.793096	0.098343
C	2.887824	1.451710	-0.299488
C	1.567333	-0.609522	-0.785259
H	2.860957	2.377747	-0.892565
C	3.296266	1.771521	1.132523
H	3.625799	0.783823	-0.764720
H	4.298419	2.226322	1.149133
H	3.323865	0.860035	1.748985
H	2.589581	2.482706	1.582191
C	0.125798	5.220301	0.693355
F	0.969543	5.719277	-0.209620
F	0.846507	4.959590	1.785989
F	-0.758962	6.171449	1.000047
C	1.709131	-0.821203	-2.287297
H	0.640048	-1.072524	-0.428371
H	2.387839	-1.113337	-0.252265
H	2.637956	-0.371866	-2.670227
H	1.732527	-1.896554	-2.519562
H	0.862811	-0.371668	-2.828531

$K^{•+}$

C	-2.781067	-0.155663	0.836640
C	-1.519231	0.427932	0.895346
C	-3.431298	-0.293344	-0.388476
C	-2.822819	0.153034	-1.560082
C	-1.558617	0.733437	-1.513853
C	-0.905332	0.863930	-0.284368
H	-3.257937	-0.504057	1.754580
H	-1.007592	0.535926	1.854164
H	-1.084927	1.091808	-2.429939
H	-4.422146	-0.750236	-0.430046
H	-3.334762	0.050661	-2.518646
C	0.422001	1.535720	-0.210515

N	0.550972	2.830497	0.128851
N	1.544100	0.890710	-0.468635
S	-0.766786	3.698682	0.490235
C	2.850401	1.571343	-0.401303
C	1.573677	-0.542701	-0.791753
H	2.730543	2.578311	-0.814407
C	3.389953	1.626149	1.020225
H	3.525040	1.005537	-1.054957
H	4.394568	2.070860	0.999693
H	3.469738	0.622028	1.461140
H	2.747089	2.248639	1.657606
C	0.146587	5.260160	0.835147
F	0.819813	5.658846	-0.226281
F	0.991146	5.098510	1.836257
F	-0.756848	6.168015	1.157298
C	1.721035	-0.800711	-2.284243
H	0.661897	-1.005564	-0.400235
H	2.427292	-0.961342	-0.240781
H	2.629727	-0.334650	-2.690410
H	1.793486	-1.885140	-2.444703
H	0.852396	-0.425905	-2.842609

L[1]

C	-3.701539	0.366514	0.386128
C	-2.566981	0.941228	0.936535
C	-3.604117	-0.382875	-0.787579
C	-2.368318	-0.574817	-1.404120
C	-1.220902	-0.015559	-0.855031
C	-1.327847	0.770241	0.298470
H	-4.663493	0.484043	0.886666
H	-2.624288	1.500234	1.870977
H	-0.256623	-0.180484	-1.334294
H	-4.500549	-0.833087	-1.217661
H	-2.294351	-1.166186	-2.317374
C	-0.136846	1.346027	0.943435
N	0.052846	2.285173	1.745518
N	1.062349	0.512283	0.901784
S	-1.854113	3.969514	0.583950
C	2.250688	0.816685	0.117550
H	1.184983	-0.095732	1.723205
C	1.889526	1.504144	-1.194505
H	1.488178	0.789841	-1.924448
H	2.804057	1.934974	-1.620660
H	1.176274	2.327079	-1.059906
C	2.967651	-0.516715	-0.116677
C	3.120523	1.747849	1.005562
H	2.313463	-1.213759	-0.657439

H	3.284849	-0.975232	0.829633
H	3.859254	-0.327234	-0.728902
H	2.593944	2.682800	1.234351
H	4.022571	1.977435	0.421242
H	3.409344	1.248497	1.939157
C	-0.405033	4.946668	0.101169
F	0.091300	4.558305	-1.076389
F	0.578655	4.887422	0.986870
F	-0.780924	6.216445	-0.025448

L²

C	-3.701539	0.366514	0.386128
C	-2.566981	0.941228	0.936535
C	-3.604117	-0.382875	-0.787579
C	-2.368318	-0.574817	-1.404120
C	-1.220902	-0.015559	-0.855031
C	-1.327847	0.770241	0.298470
H	-4.663493	0.484043	0.886666
H	-2.624288	1.500234	1.870977
H	-0.256623	-0.180484	-1.334294
H	-4.500549	-0.833087	-1.217661
H	-2.294351	-1.166186	-2.317374
C	-0.136846	1.346027	0.943435
N	0.052846	2.285173	1.745518
N	1.062349	0.512283	0.901784
S	-1.854113	3.969514	0.583950
C	2.250688	0.816685	0.117550
H	1.184983	-0.095732	1.723205
C	1.889526	1.504144	-1.194505
H	1.488178	0.789841	-1.924448
H	2.804057	1.934974	-1.620660
H	1.176274	2.327079	-1.059906
C	2.967651	-0.516715	-0.116677
C	3.120523	1.747849	1.005562
H	2.313463	-1.213759	-0.657439
H	3.284849	-0.975232	0.829633
H	3.859254	-0.327234	-0.728902
H	2.593944	2.682800	1.234351
H	4.022571	1.977435	0.421242
H	3.409344	1.248497	1.939157
C	-0.405033	4.946668	0.101169
F	0.091300	4.558305	-1.076389
F	0.578655	4.887422	0.986870
F	-0.780924	6.216445	-0.025448

M¹

C	-7.716217	2.905922	0.001077
C	-6.341221	3.110567	0.001212
C	-5.479924	2.005085	0.000265
C	-8.230925	1.609916	-0.000051
C	-7.372183	0.511121	-0.000976
C	-5.995270	0.701805	-0.000724
C	-4.056934	2.209649	0.000021
N	-2.913288	2.374101	-0.000922
H	-5.929460	4.120966	0.001540
H	-8.390839	3.764050	0.001773
H	-9.311906	1.454782	-0.000106
H	-7.777908	-0.502250	-0.001673
H	-5.315414	-0.151526	-0.001437

M^2

C	-5.968934	2.854851	-0.022210
N	-4.562601	2.498942	-0.016752
H	-6.248080	3.443726	-0.912662
H	-6.258438	3.444077	0.864744
H	-6.580516	1.940994	-0.025959
C	-3.669355	3.404351	0.005062
C	-3.907310	4.892894	0.023962
H	-4.965382	5.166415	0.101818
H	-3.496660	5.341880	-0.894816
H	-3.361223	5.347166	0.865313
C	-2.222321	2.989815	0.003279
H	-2.142438	1.895804	-0.026266
H	-1.709350	3.369156	0.901680
H	-1.698872	3.417169	-0.867203

[M^2+H]$^+$

C	-0.416092	2.748888	0.115659
H	0.569123	2.726792	-0.373786
H	-1.073194	3.454898	-0.406933
H	-0.253765	3.086585	1.150002
C	-0.973538	1.373362	0.137833
N	-2.100542	1.143994	-0.441705
C	-0.216865	0.305940	0.833178
H	0.799077	0.264247	0.412696
H	-0.684754	-0.680840	0.771041
H	-0.108512	0.593514	1.890353
C	-2.810532	-0.127997	-0.534215
H	-2.139293	-0.915809	-0.894979
H	-3.634410	-0.001180	-1.243336
H	-3.216101	-0.406539	0.447492
H	-2.560181	1.941175	-0.883011

N

S	-1.170333	3.645044	0.041612
C	-0.171485	5.108178	0.378989
F	0.797855	5.280442	-0.516227
F	0.406702	5.066040	1.576760
F	-0.967849	6.175327	0.339906

O[1]

C	-1.586247	5.649265	1.469097
C	-2.094916	4.652808	0.470189
C	-1.392394	4.757042	-0.884405
H	-1.480845	5.786428	-1.271143
C	-1.966148	3.786857	-1.911169
H	-0.317892	4.562201	-0.753988
H	-3.034058	4.027708	-2.068410
H	-1.949555	2.751903	-1.536785
C	-1.342834	3.856198	-3.269225
C	-1.496126	2.888872	-4.231774
C	-1.071629	3.147470	-5.621470
O	-0.563539	4.176288	-6.008133
O	-1.314979	2.104463	-6.410116
C	-0.949913	2.237783	-7.782100
H	-1.231299	1.297343	-8.267270
H	0.131837	2.406065	-7.877239
H	-1.485339	3.079289	-8.243114
C	-1.030202	5.350958	2.641965
H	-3.177779	4.813134	0.320647
H	-1.977772	3.632007	0.869826
S	1.058041	3.217714	-3.011514
H	-1.685249	6.704304	1.181887
H	-0.908536	4.312338	2.967500
H	-0.678048	6.130639	3.322892
C	0.827991	1.810980	-1.918240
F	-0.107423	0.957423	-2.352296
F	0.468828	2.157487	-0.673552
F	1.973280	1.132176	-1.813475
H	-1.912131	1.907223	-3.999422
H	-1.008942	4.839955	-3.616024

O[2]

C	-2.410583	6.139679	1.104016
C	-2.281394	4.791208	0.483594
C	-1.699022	4.833453	-0.930845
H	-2.312004	5.499360	-1.560377
C	-1.630777	3.445716	-1.573473

H	-0.689224	5.274953	-0.899711
H	-2.636203	2.998444	-1.618058
H	-1.015218	2.791488	-0.930708
C	-1.019652	3.473663	-2.934981
C	-1.604812	3.040492	-4.055186
C	-0.907320	3.116872	-5.355412
O	0.206436	3.558713	-5.535349
O	-1.659984	2.631721	-6.345388
C	-1.093076	2.652367	-7.651810
H	-1.846195	2.223130	-8.321397
H	-0.172271	2.053198	-7.684279
H	-0.858545	3.681966	-7.956452
C	-2.014954	6.458261	2.361960
H	-3.292717	4.344855	0.433322
H	-1.678934	4.132722	1.129254
H	-0.009348	3.892164	-3.018430
H	-2.610966	2.614304	-4.056959
H	-2.906735	6.909630	0.500604
H	-1.435092	5.749783	2.959560
S	-4.174682	5.791767	3.694004
H	-2.089918	7.483284	2.729724
C	-3.251309	6.012810	5.212645
F	-4.047127	5.778655	6.260975
F	-2.202505	5.184253	5.321833
F	-2.759891	7.250883	5.355721

P[1]

C	-1.502289	5.589272	1.499960
C	-1.941433	4.607207	0.441273
C	-1.052029	4.619415	-0.798281
H	-1.042882	5.632895	-1.234816
C	-1.507167	3.619918	-1.852739
H	-0.014121	4.400906	-0.502904
H	-2.551487	3.836283	-2.132971
H	-1.513588	2.600771	-1.434955
C	-0.707063	3.642401	-3.152855
C	-1.227725	2.732567	-4.195993
C	-1.112970	3.090006	-5.600346
O	-0.639241	4.134158	-6.008094
O	-1.591194	2.139687	-6.408576
C	-1.530488	2.400722	-7.807094
H	-1.957297	1.522289	-8.302852
H	-0.490030	2.549098	-8.128894
H	-2.112567	3.298353	-8.059678
C	-2.087190	5.758390	2.684244
H	-2.983718	4.822373	0.148659
H	-1.958009	3.589318	0.867785

S	1.125911	3.349432	-2.956259
H	-0.627982	6.206208	1.255583
H	-2.962001	5.169754	2.981244
H	-1.714676	6.492008	3.404004
C	1.159507	1.786371	-2.055139
F	0.356248	0.856754	-2.579543
F	0.808691	1.908405	-0.769636
F	2.405739	1.320489	-2.083820
H	-1.658258	1.765718	-3.931306
H	-0.674151	4.663433	-3.562493

P[2]

C	-1.954560	6.169812	1.140055
C	-1.803993	4.827470	0.514639
C	-1.419586	4.874659	-0.961486
H	-2.169439	5.463564	-1.515375
C	-1.304095	3.482746	-1.587901
H	-0.461549	5.409261	-1.072943
H	-2.259752	2.943171	-1.494197
H	-0.551792	2.905045	-1.021511
C	-0.878154	3.528079	-3.017601
C	-1.558805	3.015107	-4.046559
C	-1.042547	3.119803	-5.426773
O	-0.005330	3.653181	-5.755247
O	-1.868045	2.545542	-6.304895
C	-1.476151	2.583755	-7.673651
H	-2.267137	2.074774	-8.234944
H	-0.517279	2.066090	-7.816976
H	-1.374875	3.622150	-8.019109
C	-2.042911	6.337108	2.605227
H	-2.753338	4.260646	0.621494
H	-1.061489	4.236068	1.081789
H	0.070251	4.033041	-3.237797
H	-2.513561	2.500859	-3.912307
H	-2.200654	7.027935	0.506425
H	-1.389519	5.630500	3.136828
S	-3.780027	5.969049	3.180454
H	-1.816371	7.363826	2.919478
C	-3.490148	6.056614	4.953574
F	-4.638898	5.843242	5.592365
F	-2.609273	5.145926	5.381854
F	-3.019035	7.246019	5.342101

Q

F	3.461368	0.356007	-2.603838
S	1.924858	2.441510	-2.183552
C	3.517988	1.690769	-2.554280

F	3.937719	2.126951	-3.739362
H	-0.830531	0.542174	-0.664825
C	1.039658	1.500808	2.921660
O	3.228194	1.355749	3.816826
F	4.454568	1.993664	-1.649237
C	-0.943614	1.603791	-0.389851
C	0.376163	2.141637	0.050930
H	-1.294251	2.122749	-1.301781
C	2.399013	0.970568	3.016148
C	0.097973	1.054405	2.038665
H	-2.849023	1.136131	0.530468
C	-1.975063	1.772371	0.729580
O	2.665135	0.003605	2.124734
C	-1.351339	1.454906	2.110534
H	4.184062	-1.001295	3.136973
H	-2.340739	2.810038	0.731104
C	3.973663	-0.552593	2.156018
H	4.729827	0.218420	1.950280
H	-1.897185	0.626258	2.590774
H	-1.447194	2.318296	2.785003
H	4.000575	-1.322075	1.376550
H	0.826481	2.327030	3.604355
H	0.329796	0.142199	1.481554
C	1.652549	1.654223	-0.528331
H	0.385158	3.165001	0.439584
H	1.640518	0.566562	-0.681662
H	2.509442	1.926271	0.100950

R•

F	3.385759	0.623563	-2.766599
S	1.715960	2.522585	-2.067881
C	3.331498	1.950085	-2.612381
F	3.612761	2.511182	-3.785682
H	-0.832059	0.639605	-0.904501
C	1.249878	1.695795	2.649258
O	3.271780	1.364367	3.818067
F	4.310065	2.269890	-1.759041
C	-0.945605	1.621621	-0.417719
C	0.377230	1.983712	0.270792
H	-1.214863	2.341848	-1.203857
C	2.477761	0.996076	2.970012
C	0.254351	1.262708	1.641400
H	-2.598506	0.629378	0.615973
C	-1.997305	1.544899	0.709418
O	2.671952	-0.111083	2.236205
C	-1.202742	1.561956	2.031822
H	3.883049	-1.195176	3.538720

H	-2.699172	2.389738	0.666159
C	3.860984	-0.847959	2.496034
H	4.750385	-0.229485	2.309611
H	-1.582375	0.845948	2.774317
H	-1.247307	2.561322	2.492802
H	3.847953	-1.704720	1.813523
H	1.097988	2.630648	3.195576
H	0.376929	0.184424	1.452778
C	1.632425	1.594602	-0.494429
H	0.402820	3.069689	0.466905
H	1.631004	0.519008	-0.721174
H	2.534614	1.833365	0.083874

R$^-$

F	3.442998	0.624047	-2.722788
S	1.750452	2.514577	-2.053570
C	3.379433	1.950117	-2.556749
F	3.697776	2.520481	-3.718354
H	-0.856370	0.588378	-0.876118
C	1.276160	1.706159	2.672919
O	3.349697	1.332955	3.780668
F	4.336246	2.262261	-1.674811
C	-0.969420	1.586249	-0.420557
C	0.350246	1.971608	0.256687
H	-1.256492	2.274206	-1.230499
C	2.456890	1.045374	2.951191
C	0.261488	1.297577	1.650790
H	-2.616641	0.622234	0.656853
C	-2.012322	1.539483	0.721281
O	2.650798	-0.117851	2.182933
C	-1.199647	1.591764	2.031794
H	3.910883	-1.173479	3.471916
H	-2.717218	2.381781	0.655040
C	3.836865	-0.825112	2.428171
H	4.735690	-0.219655	2.223238
H	-1.573785	0.898291	2.800408
H	-1.246995	2.603716	2.468264
H	3.834587	-1.697768	1.758985
H	1.101912	2.631138	3.233955
H	0.358434	0.212270	1.467610
C	1.610300	1.575962	-0.488600
H	0.371071	3.064173	0.425355
H	1.603686	0.502543	-0.725585
H	2.490487	1.796111	0.129556

R

F	1.257663	2.999882	-5.149056

S	0.279952	2.670463	-2.807287
C	1.587441	2.395224	-4.010238
F	2.768712	2.881792	-3.614747
H	-1.216252	0.373760	-0.929869
C	1.967171	1.807609	1.634192
O	2.709337	1.658852	3.925669
F	1.783926	1.099699	-4.273937
C	-1.273668	1.285525	-0.313892
C	0.136241	1.884303	-0.176368
H	-1.967104	1.970388	-0.823105
C	2.479770	1.117327	2.870266
C	0.704172	1.169001	1.060165
H	-2.062318	-0.108757	1.176063
C	-1.725822	0.936645	1.123350
O	2.666152	-0.189014	2.676743
C	-0.492174	1.164885	2.017567
H	2.459843	-0.875688	4.633419
H	-2.572442	1.557463	1.449026
C	3.154268	-0.942325	3.784578
H	4.139494	-0.571518	4.099492
H	-0.393080	0.409673	2.811453
H	-0.553149	2.148937	2.511045
H	3.232882	-1.979208	3.440598
H	2.779027	1.780289	0.889547
H	1.787764	2.859082	1.898681
H	0.937916	0.128706	0.775843
C	1.011502	1.741206	-1.412371
H	0.051156	2.957432	0.071420
H	1.098111	0.684802	-1.705536
H	2.017160	2.145914	-1.236182

S

C	-2.257850	1.439062	-0.205699
C	-2.374715	0.095915	0.053762
N	-1.147266	-0.477823	0.132062
N	-0.905757	1.693698	-0.289458
C	-0.299751	0.495296	-0.075803
Br	-3.548900	2.751085	-0.423093
Br	-3.954378	-0.875247	0.279728
Br	1.552909	0.279317	-0.082292
C	-0.298812	2.986607	-0.552633
H	-0.580770	3.699067	0.233113
H	-0.631504	3.364123	-1.527922
H	0.788944	2.868820	-0.561626

T[1]

C	-1.896384	1.031945	-0.791556
C	-2.114092	-0.060520	0.115660
N	-1.166967	-0.099396	1.050924
N	-0.826158	1.714929	-0.217339
C	-0.409892	0.961299	0.830517
Br	-3.326928	2.036556	-1.524990
Br	-3.466771	-1.310804	-0.022716
Br	1.088577	1.365108	1.834765
C	-0.194287	2.881517	-0.802769
H	-0.928458	3.691055	-0.896282
H	0.197778	2.618584	-1.794883
H	0.623933	3.206631	-0.153562
F	-0.084887	-1.795505	-1.311295
S	-1.163326	0.114371	-2.758714
C	0.247677	-0.799688	-2.143123
F	0.917147	-1.343325	-3.161749
F	1.118688	-0.028839	-1.466239

T^2

C	-2.281959	0.983421	-0.643256
C	-2.265912	-0.398717	-0.354287
N	-1.058411	-0.794800	-0.013967
N	-1.015756	1.418521	-0.457203
C	-0.253401	0.288067	-0.161826
Br	-3.678391	2.029389	-1.219976
Br	-3.732537	-1.536801	-0.448050
Br	1.210552	0.496664	1.027237
C	-0.478371	2.748814	-0.669069
F	0.175704	-0.275499	-4.514217
F	-1.282223	-0.966541	-3.088164
C	-0.325310	-0.051423	-3.297619
S	0.986204	-0.099529	-2.079745
F	-0.957557	1.135327	-3.346962
H	0.319806	2.691546	-1.420961
H	-0.076284	3.141336	0.273424
H	-1.278564	3.402904	-1.028189

T^3

C	-1.758264	1.382253	-1.102910
C	-1.645096	-0.050701	-1.156098
N	-0.675986	-0.420698	-0.227156
N	-0.749750	1.820008	-0.295899
C	-0.155455	0.680209	0.200619
Br	-2.895168	2.482150	-2.028579
Br	-3.255898	-1.103857	-1.169798
Br	1.273051	0.760362	1.383259

C	-0.352018	3.203281	-0.101907
H	-1.243707	3.816008	0.074622
H	0.178169	3.565775	-0.993579
H	0.306658	3.264501	0.769452
F	1.144948	0.244133	-4.381504
S	-0.937959	-0.587103	-3.136022
C	0.525720	0.448442	-3.218308
F	0.252077	1.762501	-3.141335
F	1.413149	0.202846	-2.245106

U[1]

C	-2.682452	2.229411	0.019088
C	-3.119224	0.881184	0.103333
N	-2.171205	0.049047	-0.316928
N	-1.426649	2.143590	-0.560445
C	-1.167298	0.827246	-0.698019
S	-3.623198	3.704339	0.097087
Br	-4.772198	0.312707	0.703985
Br	0.434532	0.168530	-1.339421
C	-0.539597	3.270963	-0.796465
H	-0.201071	3.672353	0.168391
H	-1.078397	4.042990	-1.355238
H	0.319355	2.929069	-1.381120
C	-4.254884	3.680918	-1.617202
F	-5.075136	4.711135	-1.754270
F	-4.915243	2.562609	-1.896930
F	-3.276464	3.792978	-2.515101
Br	-2.012064	2.396432	2.557745

U[2]

C	-2.171697	2.220221	-0.303530
C	-2.525660	0.915532	0.124161
N	-1.447184	0.202715	0.363633
N	-0.831010	2.263232	-0.297716
C	-0.396769	0.988066	0.053015
Br	-3.254033	3.610701	-0.813566
Br	-4.257309	0.278109	0.322258
S	1.232960	0.683803	0.652741
C	0.006673	3.375143	-0.715682
H	-0.433268	4.310702	-0.353437
H	0.078328	3.388531	-1.811582
H	1.001825	3.241865	-0.281319
C	0.960205	1.270940	2.360287
F	2.118046	1.196529	3.000112
F	0.063245	0.544276	3.015089
F	0.537041	2.535319	2.386103

Br	0.210788	0.132675	-2.302838

U[3]

C	-3.560537	1.748429	-1.543639
C	-4.498167	0.763047	-1.169627
N	-3.933786	-0.467284	-1.308980
N	-2.425172	1.084891	-1.872173
C	-2.713123	-0.250787	-1.704687
Br	-1.459790	-1.580830	-2.005558
C	-1.158226	1.688118	-2.258730
H	-0.673177	2.128741	-1.377657
H	-1.342422	2.463016	-3.011413
H	-0.515422	0.912798	-2.685426
S	-6.195971	1.023646	-0.798272
Br	-3.768618	3.565754	-1.726326
C	-6.835170	0.813991	-2.489869
F	-6.652006	-0.416104	-2.958615
F	-6.258212	1.650404	-3.350810
F	-8.137985	1.064995	-2.462851
Br	-3.688905	1.465968	1.402704

V[1]

C	-2.552035	2.337793	-0.499392
C	-3.040075	0.977929	-0.325123
N	-2.023206	0.137911	-0.278540
N	-1.203819	2.216576	-0.520587
C	-0.928236	0.877550	-0.390142
S	-3.262957	3.896522	-0.516769
Br	-4.772188	0.444584	-0.085516
Br	0.765016	0.202858	-0.377674
C	-0.247122	3.311648	-0.651934
H	-0.143710	3.827747	0.311144
H	-0.611353	4.003948	-1.420430
H	0.717612	2.898322	-0.959187
C	-4.817223	3.644735	-1.497195
F	-5.021056	4.789029	-2.117503
F	-5.850682	3.388065	-0.723892
F	-4.676297	2.676254	-2.379232

V[2]

C	-2.552035	2.337793	-0.499392
C	-3.040075	0.977929	-0.325123
N	-2.023206	0.137911	-0.278540
N	-1.203819	2.216576	-0.520587
C	-0.928236	0.877550	-0.390142
S	-3.262957	3.896522	-0.516769

Br	-4.772188	0.444584	-0.085516
Br	0.765016	0.202858	-0.377674
C	-0.247122	3.311648	-0.651934
H	-0.143710	3.827747	0.311144
H	-0.611353	4.003948	-1.420430
H	0.717612	2.898322	-0.959187
C	-4.817223	3.644735	-1.497195
F	-5.021056	4.789029	-2.117503
F	-5.850682	3.388065	-0.723892
F	-4.676297	2.676254	-2.379232

V^3

C	-3.424130	1.927179	-1.438090
C	-4.401232	0.948008	-1.026258
N	-3.891625	-0.280477	-1.163131
N	-2.344709	1.224335	-1.812052
C	-2.674968	-0.108426	-1.628738
Br	-1.501088	-1.453322	-2.018029
C	-1.070918	1.729282	-2.312768
H	-1.117900	2.821664	-2.346025
H	-0.900424	1.333817	-3.321150
H	-0.268542	1.411778	-1.636264
S	-5.957248	1.368586	-0.458987
Br	-3.587693	3.736535	-1.457010
C	-6.641319	-0.314178	-0.135318
F	-7.866318	-0.110889	0.315468
F	-5.941565	-0.957633	0.775460
F	-6.694100	-1.037341	-1.234240

X^1

C	-2.719478	2.220813	-0.083803
C	-3.073654	0.915869	0.189463
N	-2.042585	0.075742	-0.038047
N	-1.394310	2.154808	-0.493943
C	-1.059629	0.847692	-0.443121
S	-3.615941	3.701979	0.079617
Br	-4.736873	0.312835	0.785625
Br	0.628591	0.206473	-0.902668
C	-0.578824	3.289111	-0.894752
H	-0.491358	3.997478	-0.061460
H	-1.039391	3.789422	-1.754775
H	0.416303	2.928340	-1.171604
C	-4.344504	3.772868	-1.587819
F	-5.083403	4.872442	-1.661518
F	-5.115764	2.723380	-1.848817
F	-3.416502	3.822237	-2.544327

X^2

C	-2.400726	2.128630	-0.545705
C	-2.928061	0.862877	-0.702112
N	-1.945306	-0.058390	-0.678227
N	-1.048830	1.963700	-0.429116
C	-0.826409	0.615941	-0.508016
Br	-3.234605	3.781641	-0.484500
Br	-4.729463	0.418883	-0.921543
S	0.768958	-0.111327	-0.460762
C	-0.072476	3.023125	-0.238634
H	-0.191556	3.471437	0.756266
H	-0.209741	3.791813	-1.008677
H	0.928929	2.591448	-0.327141
C	0.960392	-0.211943	1.346604
F	2.147301	-0.747756	1.598464
F	0.024655	-0.962946	1.915075
F	0.905937	0.987178	1.926264

X^3

C	-3.544125	1.578434	-1.262622
C	-4.049960	0.746763	-0.287235
N	-3.164768	-0.270773	-0.052545
N	-2.334004	1.056237	-1.635561
C	-2.168892	-0.058848	-0.862277
Br	-0.657004	-1.145974	-0.973220
C	-1.438422	1.604977	-2.639926
H	-1.104946	2.605777	-2.337160
H	-1.955328	1.665254	-3.605868
H	-0.570376	0.945798	-2.733990
S	-5.556820	0.938216	0.587078
Br	-4.248432	3.114761	-2.020746
C	-6.646771	0.038938	-0.551224
F	-7.879050	0.076143	-0.052667
F	-6.293452	-1.235646	-0.700515
F	-6.671429	0.578863	-1.768653

10. X-ray Crystal Structure Analysis

X-ray crystal structure analysis of 3b: A colorless plate-like specimen of $C_{13}H_{17}F_3N_2OS$, approximate dimensions 0.030 mm x 0.080 mm x 0.120 mm, was used for the X-ray crystallographic analysis. The X-ray intensity data were measured. A total of 1626 frames were collected. The total exposure time was 21.09 hours. The frames were integrated with the Bruker SAINT software package using a wide-frame algorithm. The integration of the data using a triclinic unit cell yielded a total of 21506 reflections to a maximum θ angle of 66.75° (0.84 Å resolution), of which 5335 were independent (average redundancy 4.031, completeness = 99.6%, R_{int} = 11.61%, R_{sig} = 11.51%) and 3028 (56.76%) were greater than 2σ(F^2). The final cell constants of a = 10.5097(6) Å, b = 12.2340(6) Å, c = 13.4900(7) Å, α = 81.668(4)°, β = 73.008(4)°, γ = 65.741(3)°, volume = 1511.68(15) $Å^3$, are based upon the refinement of the XYZ-centroids of 1640 reflections above 20 σ(I) with 9.756° < 2θ < 128.1°. Data were corrected for absorption effects using the multi-scan method (SADABS). The ratio of minimum to maximum apparent transmission was 0.807. The calculated minimum and maximum transmission coefficients (based on crystal size) are 0.7790 and 0.9370. The structure was solved and refined using the Bruker SHELXTL Software Package, using the space group *P*-1, with Z = 4 for the formula unit, $C_{13}H_{17}F_3N_2OS$. The final anisotropic full-matrix least-squares refinement on F^2 with 377 variables converged at R1 = 5.35%, for the observed data and wR2 = 11.99% for all data. The goodness-of-fit was 0.877. The largest peak in the final difference electron density synthesis was 0.278 $e^-/Å^3$ and the largest hole was −0.363 $e^-/Å^3$ with an RMS deviation of 0.071 $e^-/Å^3$. On the basis of the final model, the calculated density was 1.346 g/cm^3 and F(000), 640 e^-. The hydrogens at N1A and N1B atoms were refined freely. CCDC Nr.: 2049022.

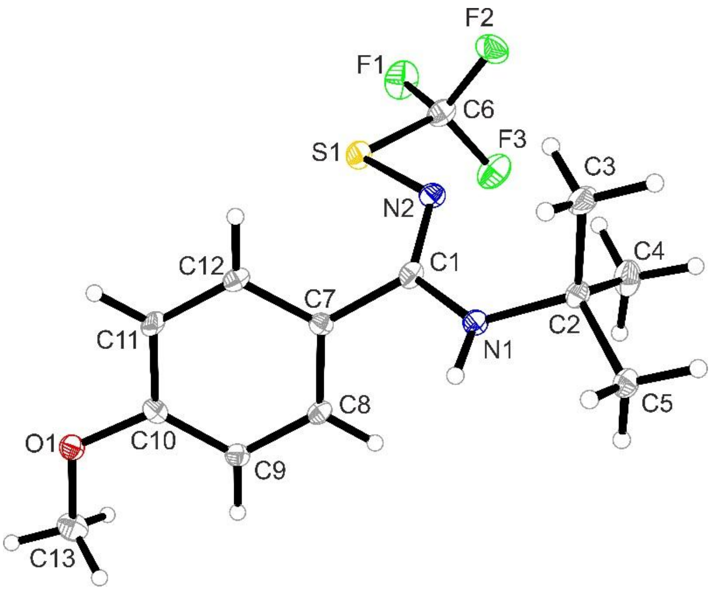

Figure S38: Crystal structure of compound **3b**. Only one independent molecule (molecule “A”) of two found in the asymmetric unit is shown. Thermal ellipsoids are set at 30% probability.

X-ray crystal structure analysis of 9c: A colorless plate-like specimen of $C_5H_3Br_2F_3N_2S$, approximate dimensions 0.035 mm x 0.094 mm x 0.116 mm, was used for the X-ray crystallographic analysis. The X-ray intensity data were measured. A total of 582 frames were collected. The total exposure time was 2.42 hours. The frames were integrated with the Bruker SAINT software package using a narrow-frame algorithm. The integration of the data using a monoclinic unit cell yielded a total of 19730 reflections to a maximum θ angle of 26.75° (0.79 Å resolution), of which 1969 were independent (average redundancy 10.020, completeness = 99.7%, R_{int} = 3.86%, R_{sig} = 1.76%) and 1740 (88.37%) were greater than 2σ(F^2). The final cell constants of a = 8.6428(2) Å, b = 6.4691(2) Å, c = 16.6210(4) Å, β = 91.3820(10)°, volume = 929.03(4) $Å^3$, are based upon the refinement of the XYZ-centroids of 8387 reflections above 20 σ(I) with 5.261° < 2θ < 53.48°. Data were corrected for absorption effects using the multi-scan method (SADABS). The ratio of minimum to maximum apparent transmission was 0.710. The calculated minimum and maximum transmission coefficients (based on crystal size) are 0.4230 and 0.7450. The structure was solved and refined using the Bruker SHELXTL Software Package, using the space group $P2_1/n$, with Z = 4 for the formula unit, $C_5H_3Br_2F_3N_2S$. The final anisotropic full-matrix least-squares refinement on F^2 with 119 variables converged at R1 = 1.66%, for the observed data and wR2 = 3.44% for all data. The goodness-of-fit was 1.069. The largest peak in the final difference electron density synthesis was 0.270 $e^-/Å^3$ and the largest

hole was −0.266 $e^-/Å^3$ with an RMS deviation of 0.067 $e^-/Å^3$. On the basis of the final model, the calculated density was 2.431 g/cm^3 and F(000), 640 e^-. CCDC Nr.: 2049023.

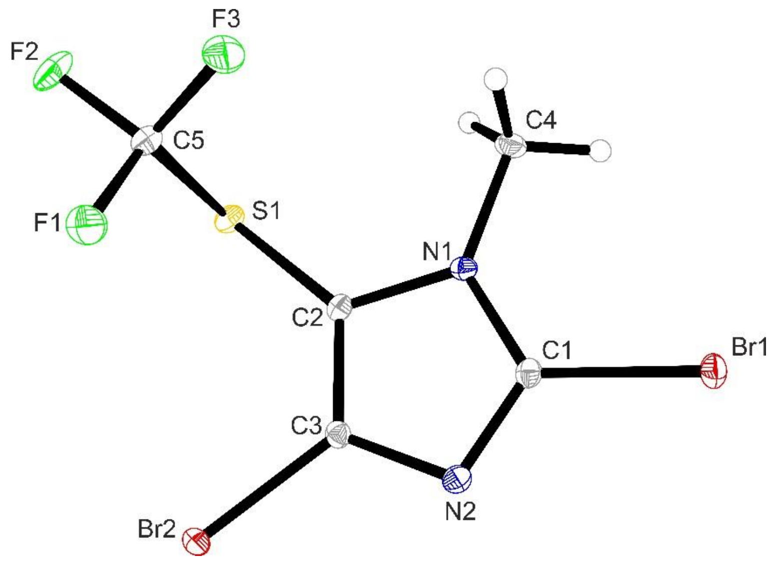

Figure S39: Crystal structure of compound **9c**. Thermal ellipsoids are shown at 30% probability.

11. NMR Spectra

N-(tert-butyl*)*benzimidamide (1a)

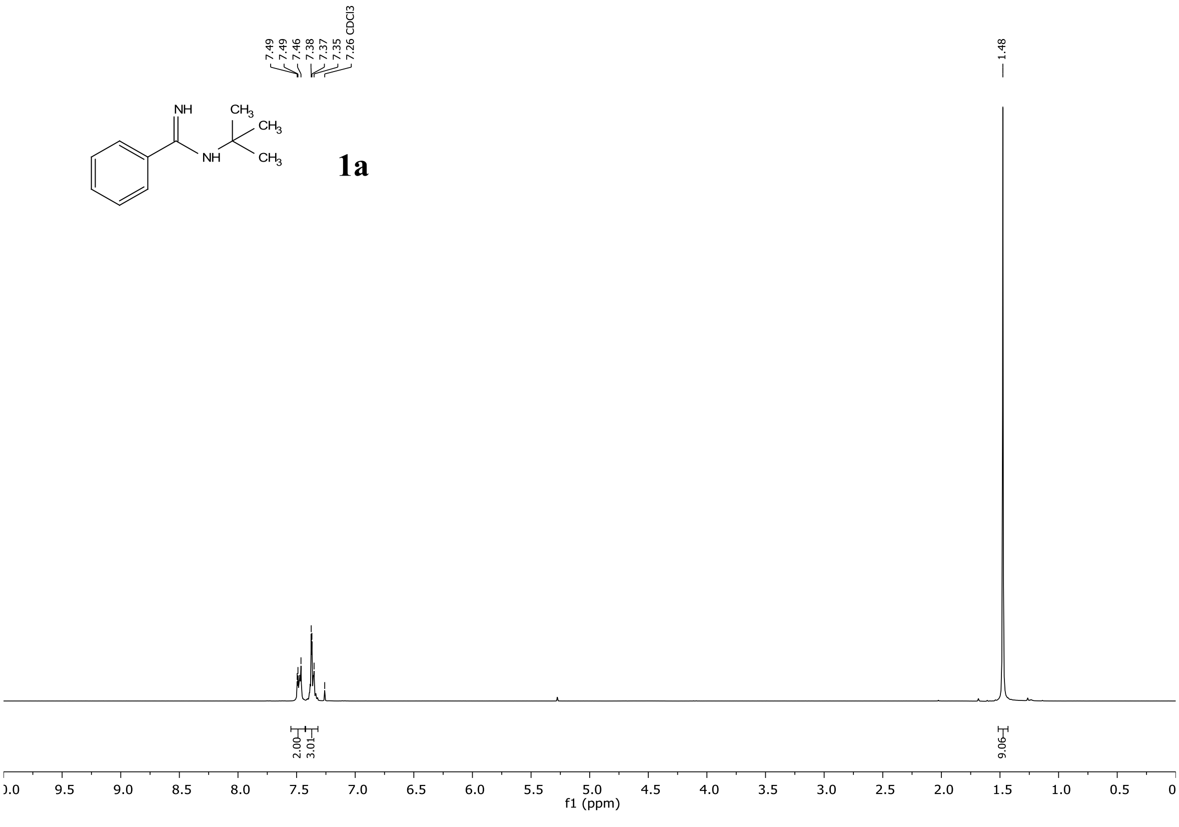

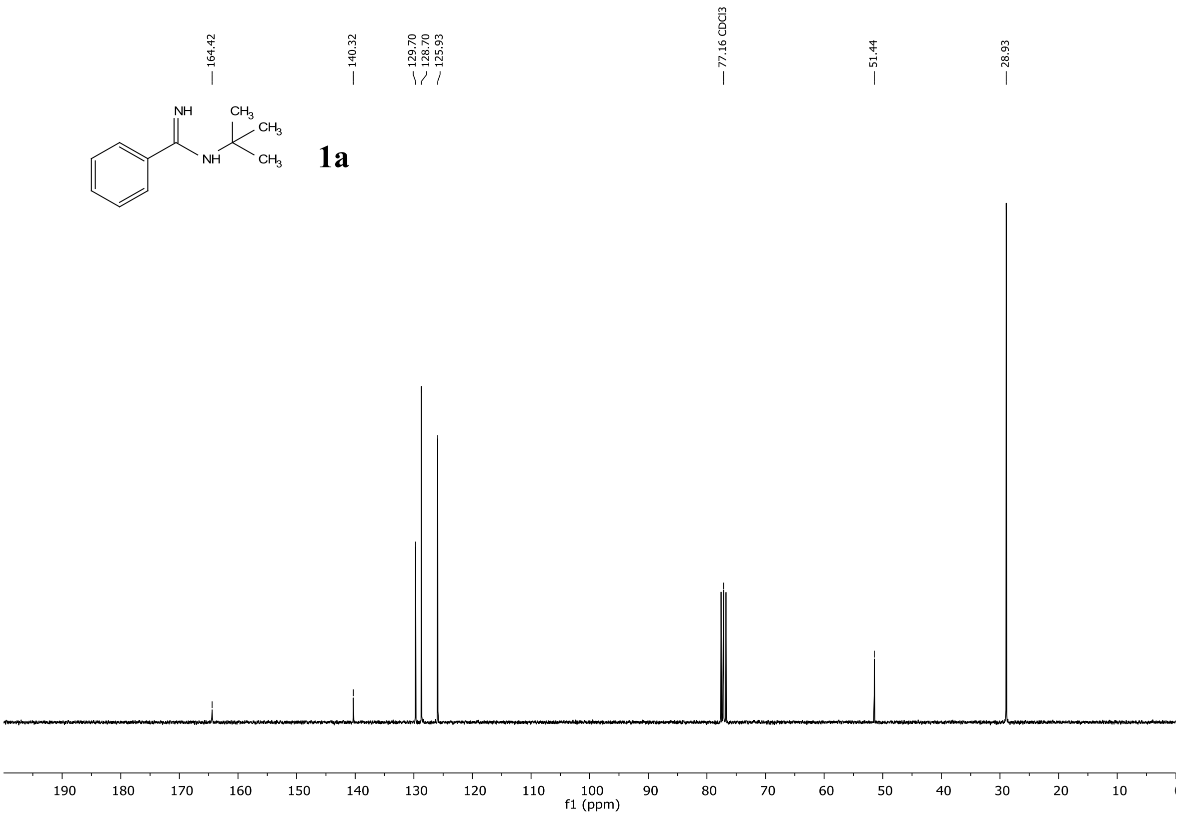

N-(*tert*-butyl)-4-methoxybenzimidamide (1b)

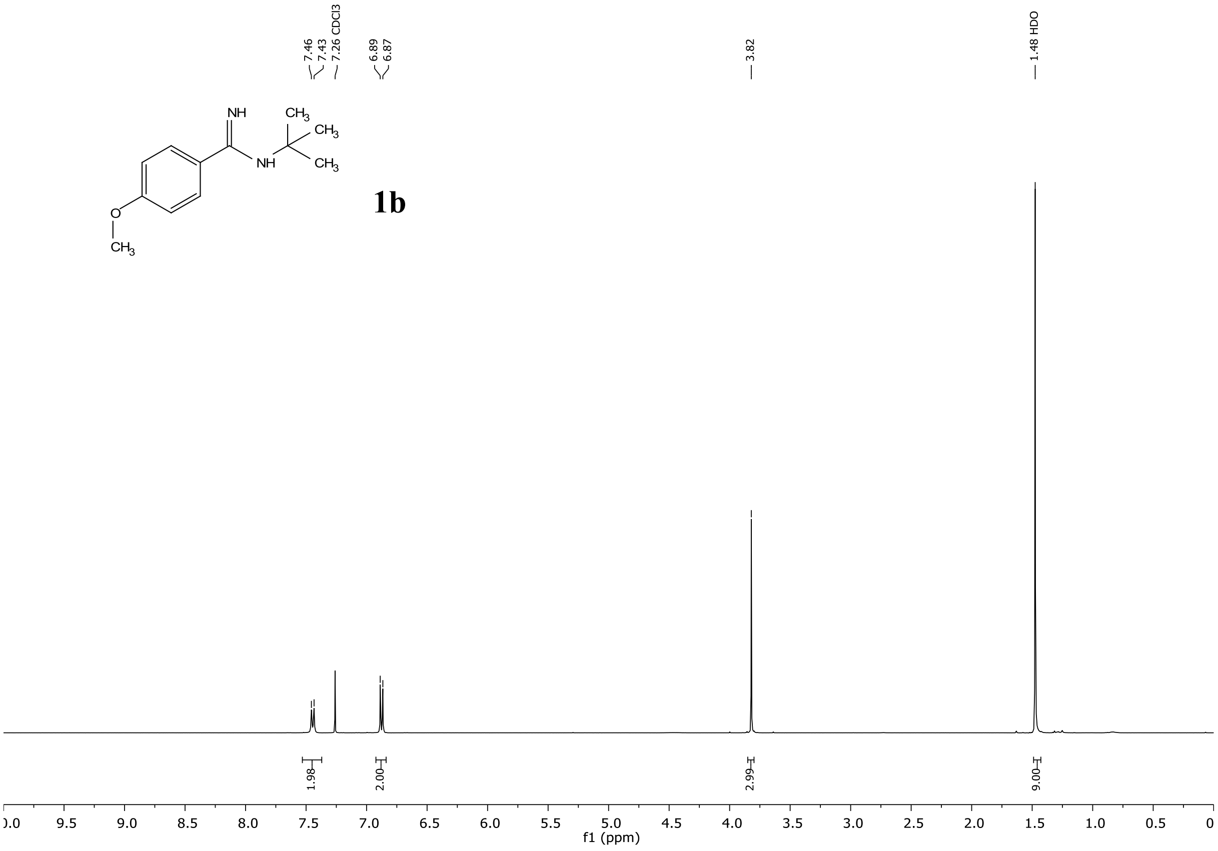

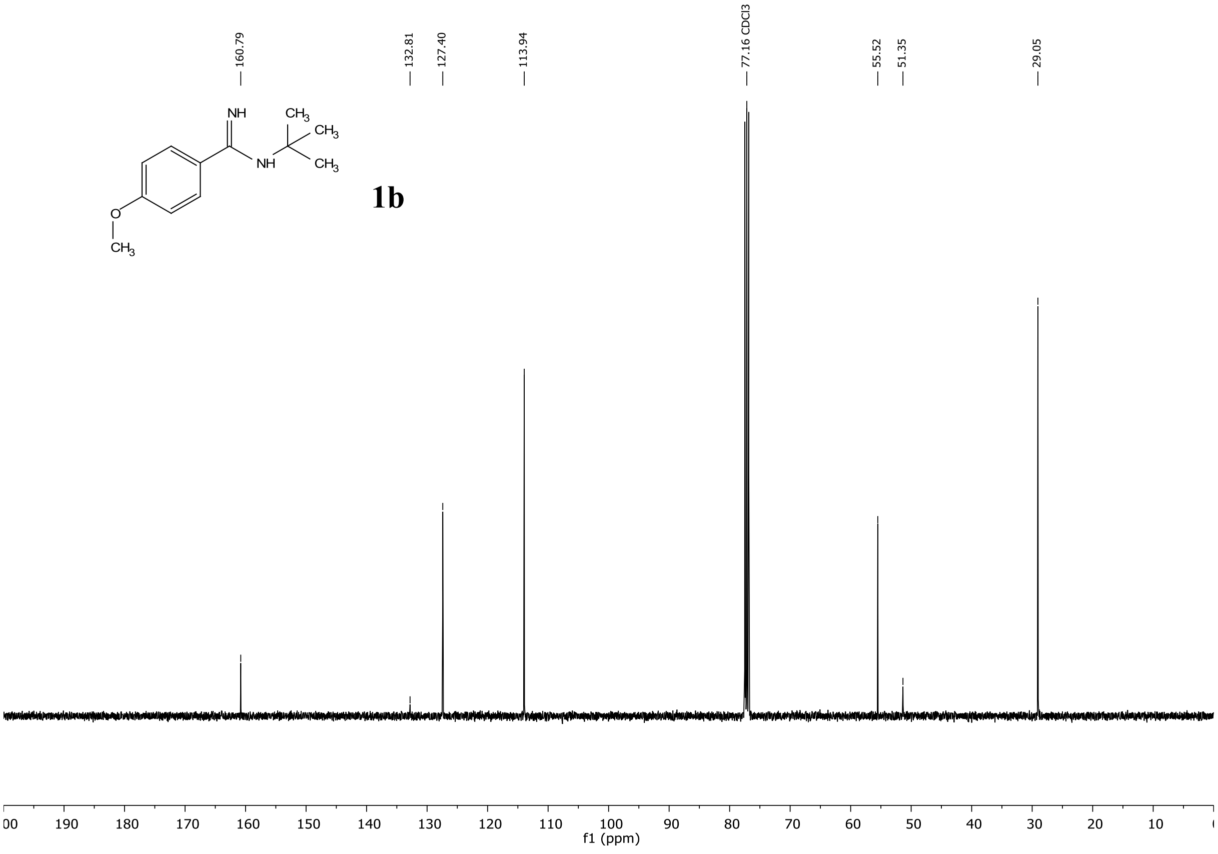

N-(*tert*-butyl)-*N*-ethylbenzimidamide (1c)

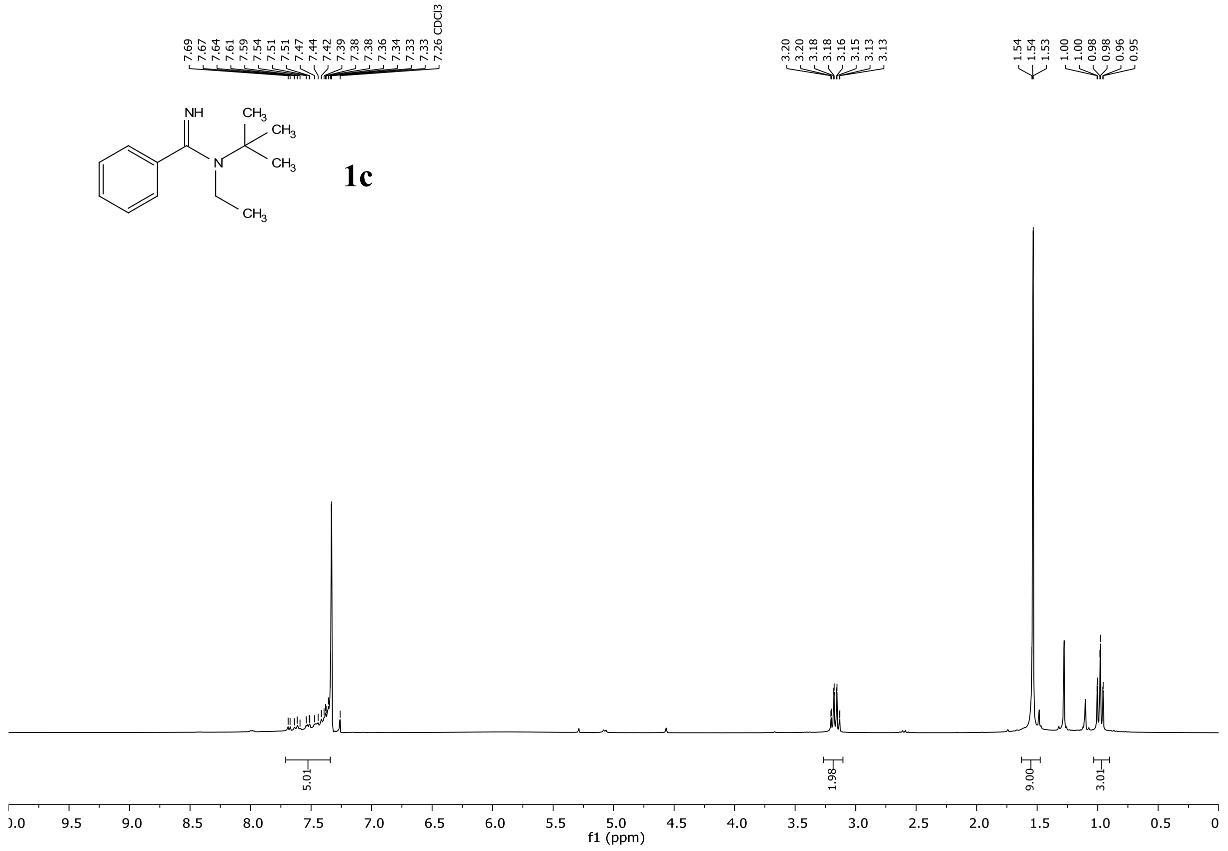

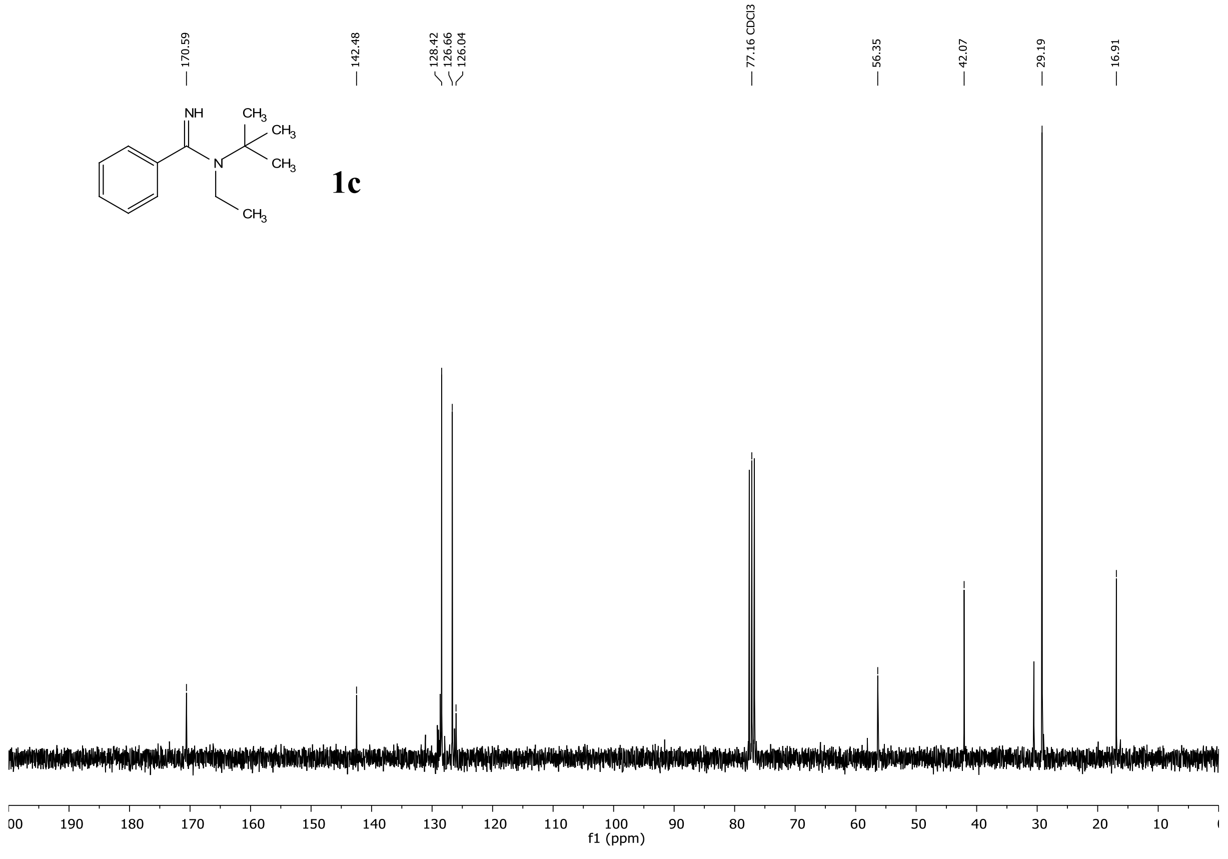

N-(*tert*-butyl)isobutyrimidamide (1d)

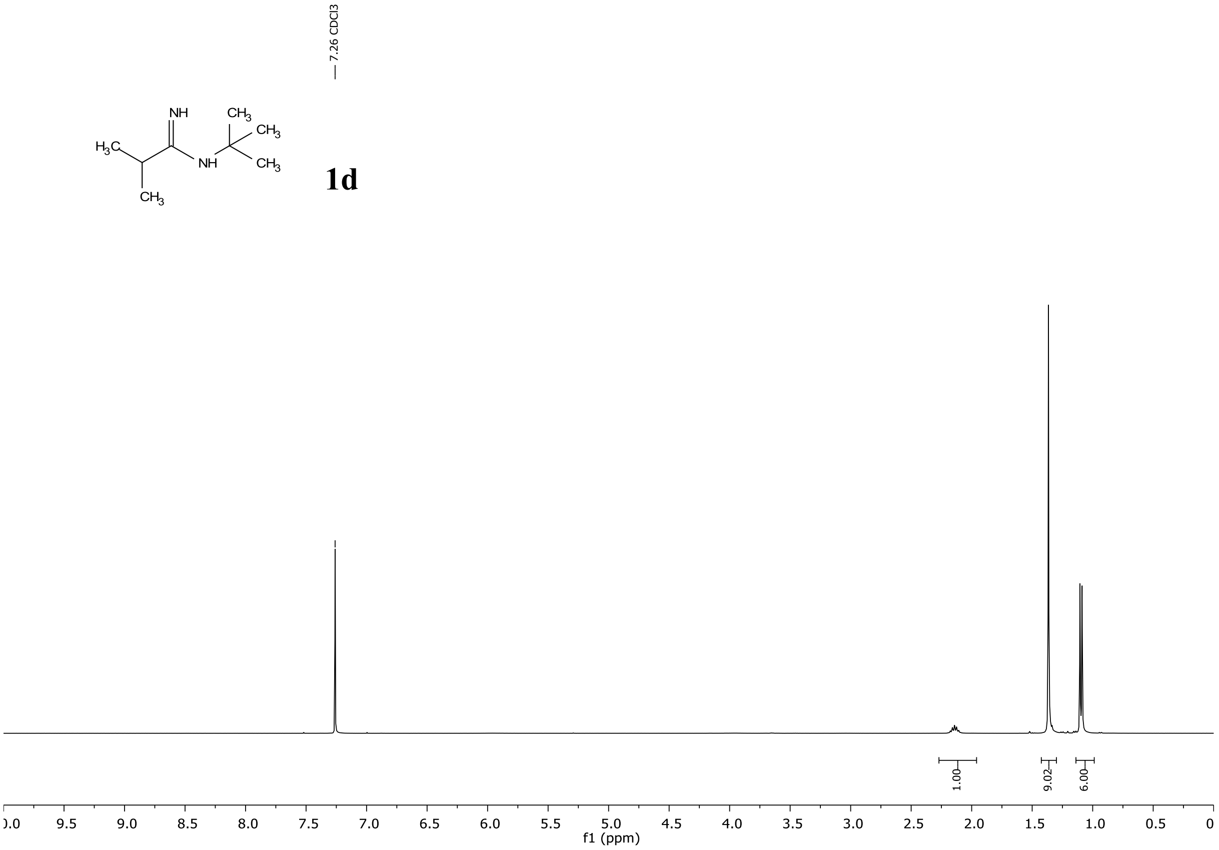

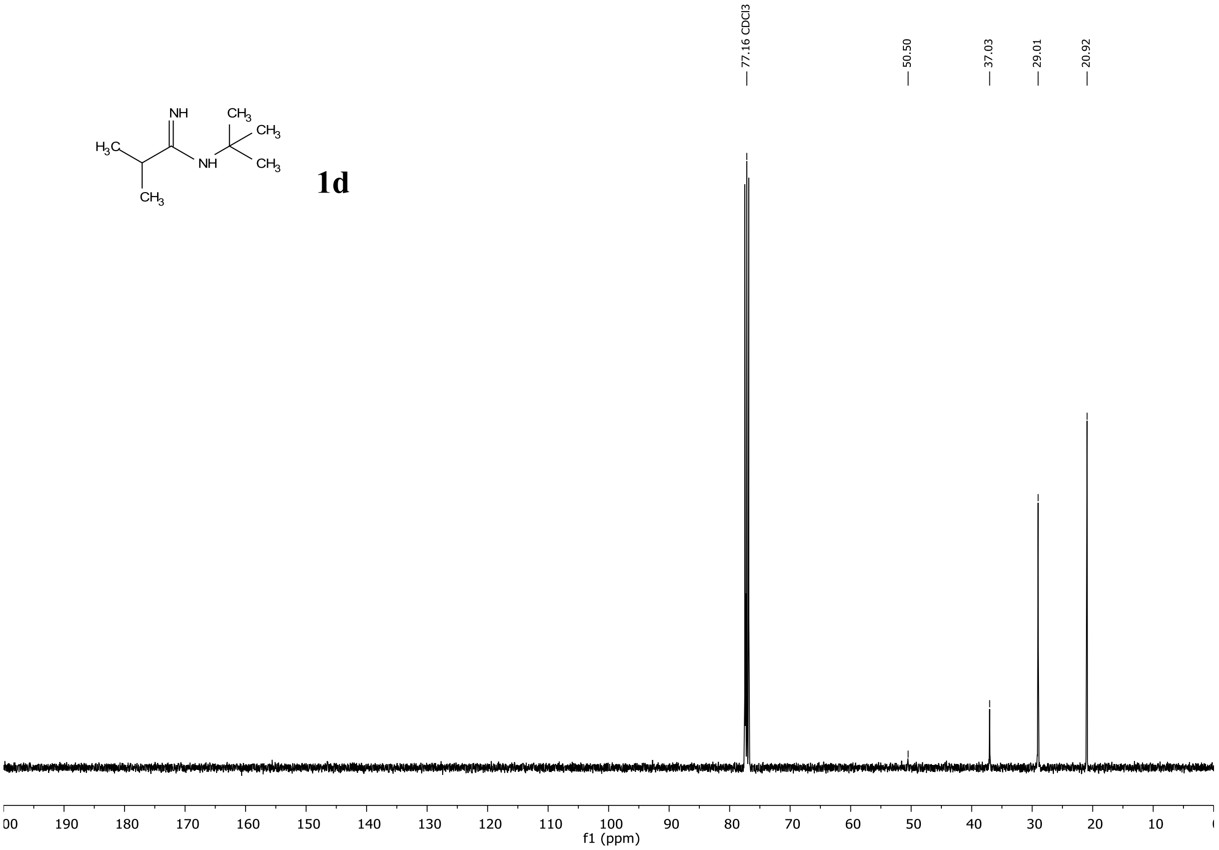

N,N-diethylbenzimidamide (1e)

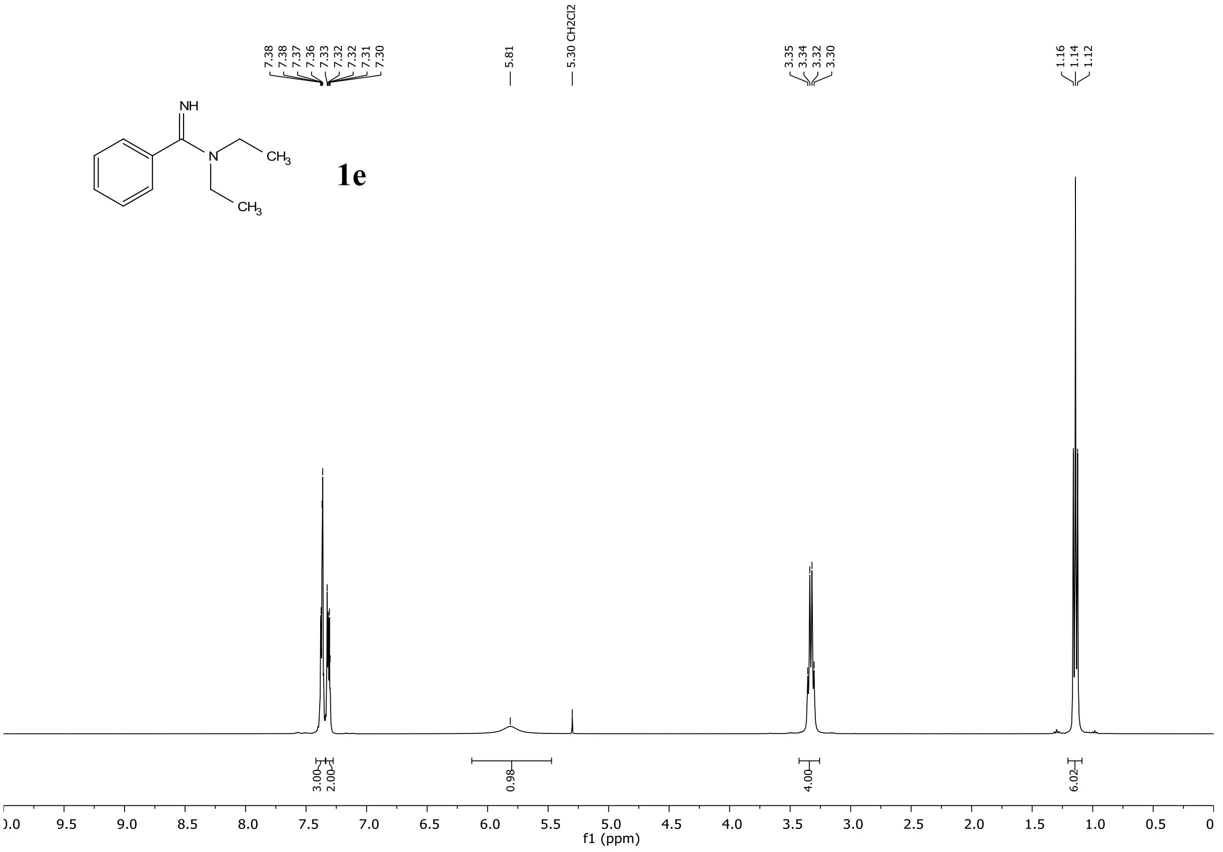

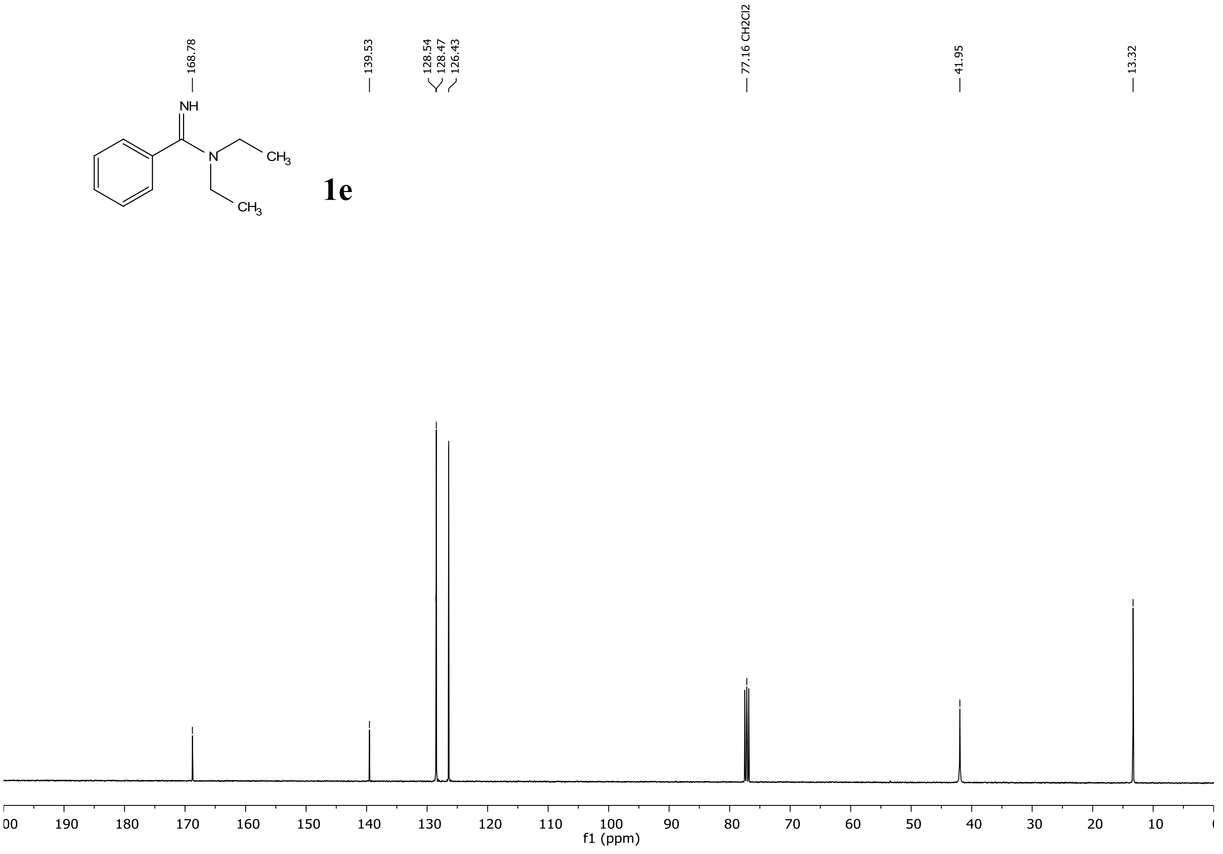

N-phenylbenzimidamide (1f)

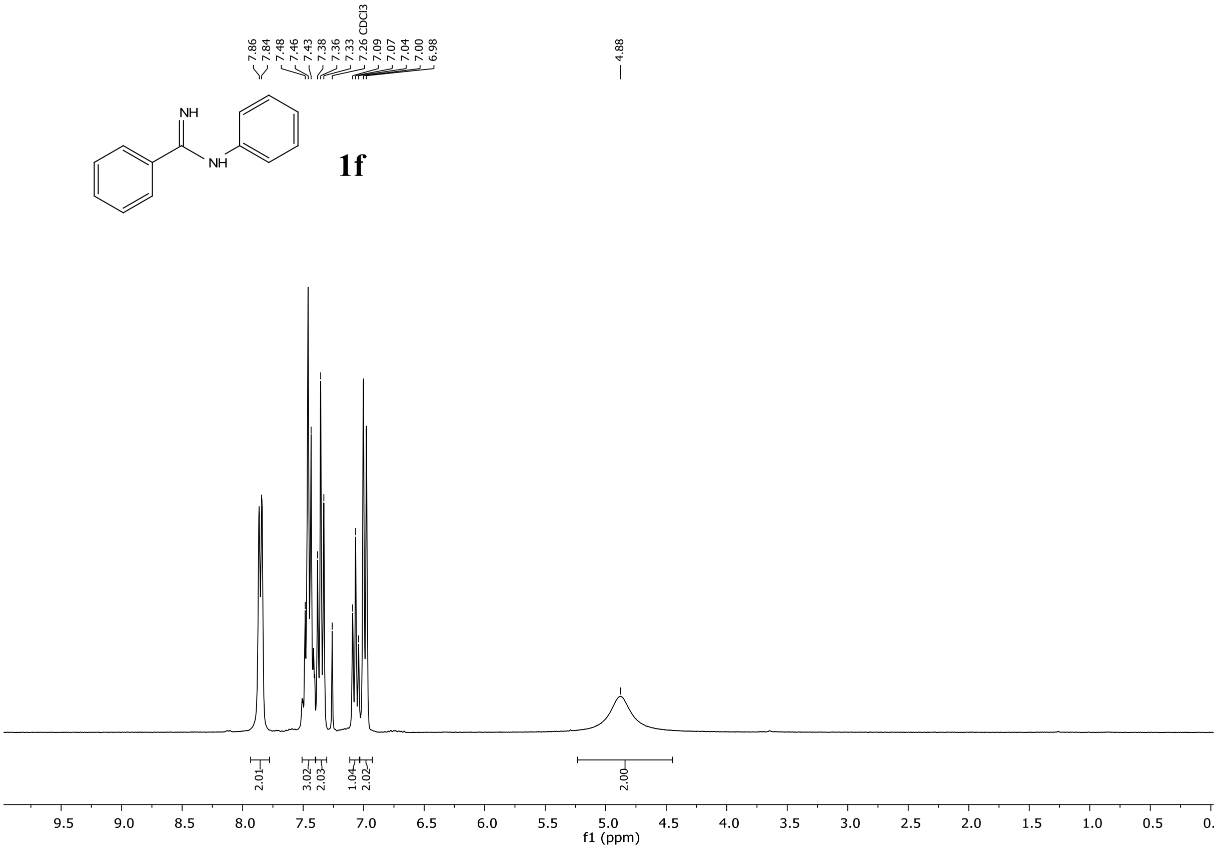

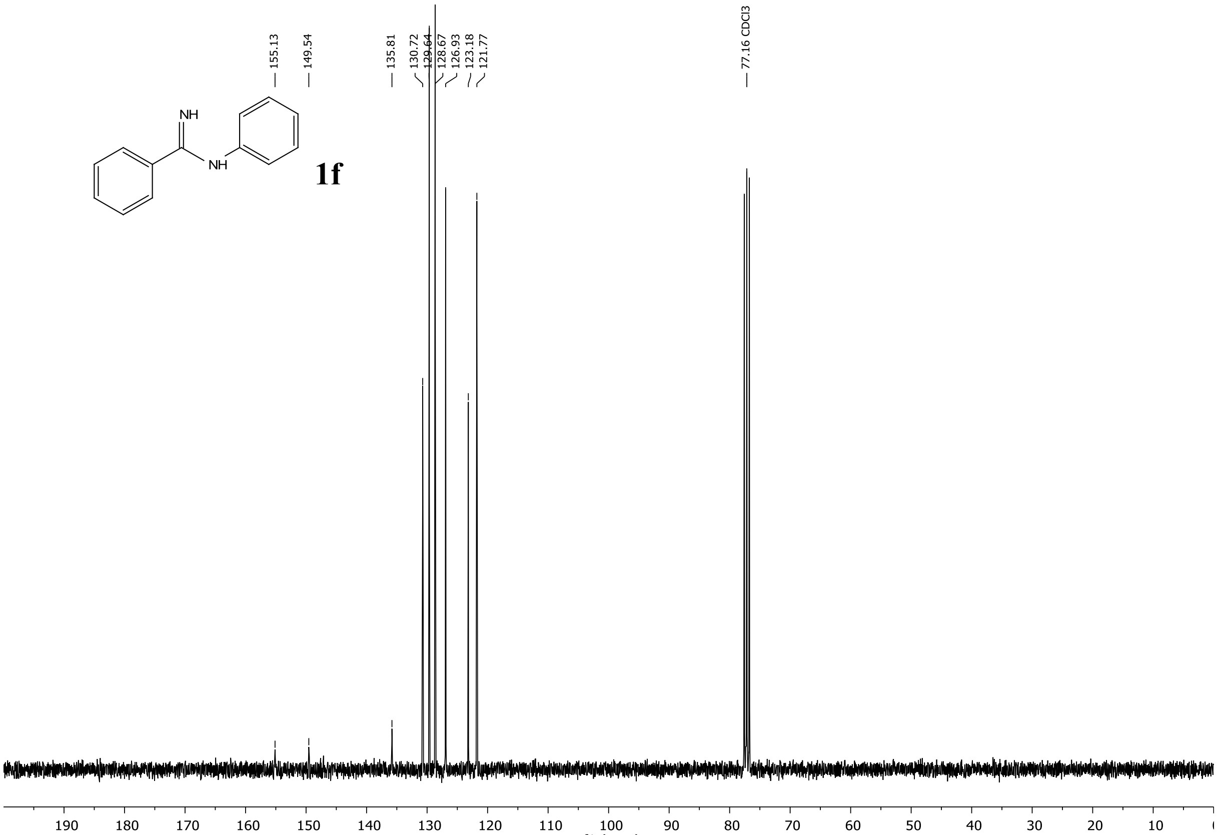

N-ethyl-4-methoxy-*N*-methylbenzimidamide (1g)

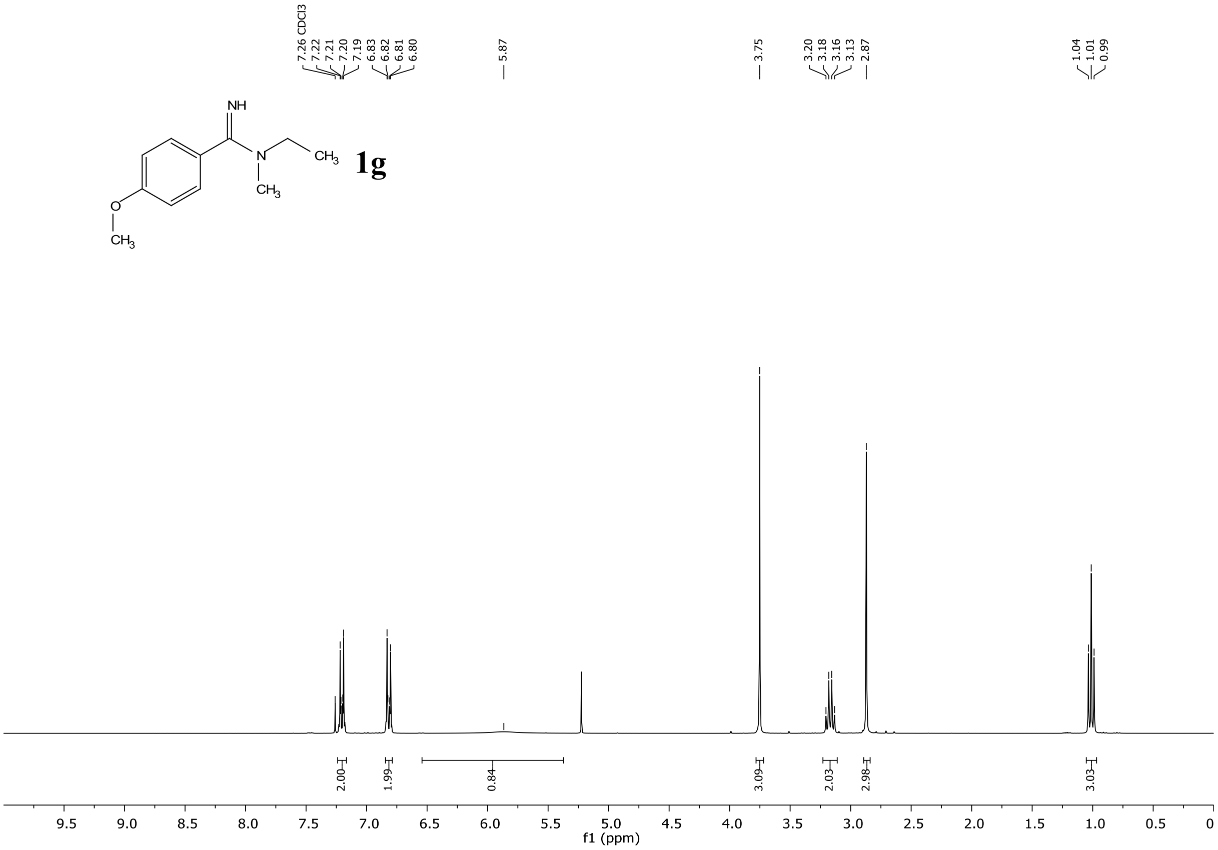

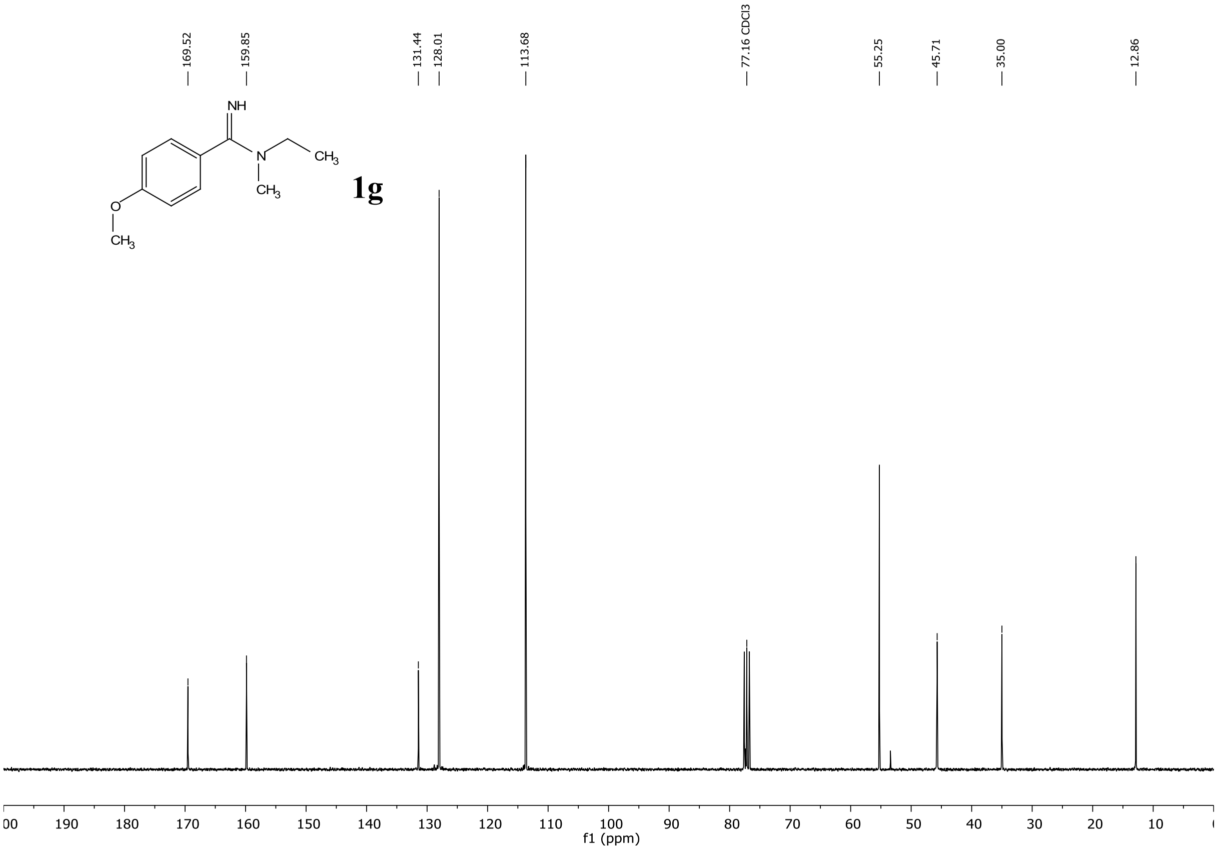

2-((Trifluoromethyl)thio)isoindoline-1,3-dione (2)

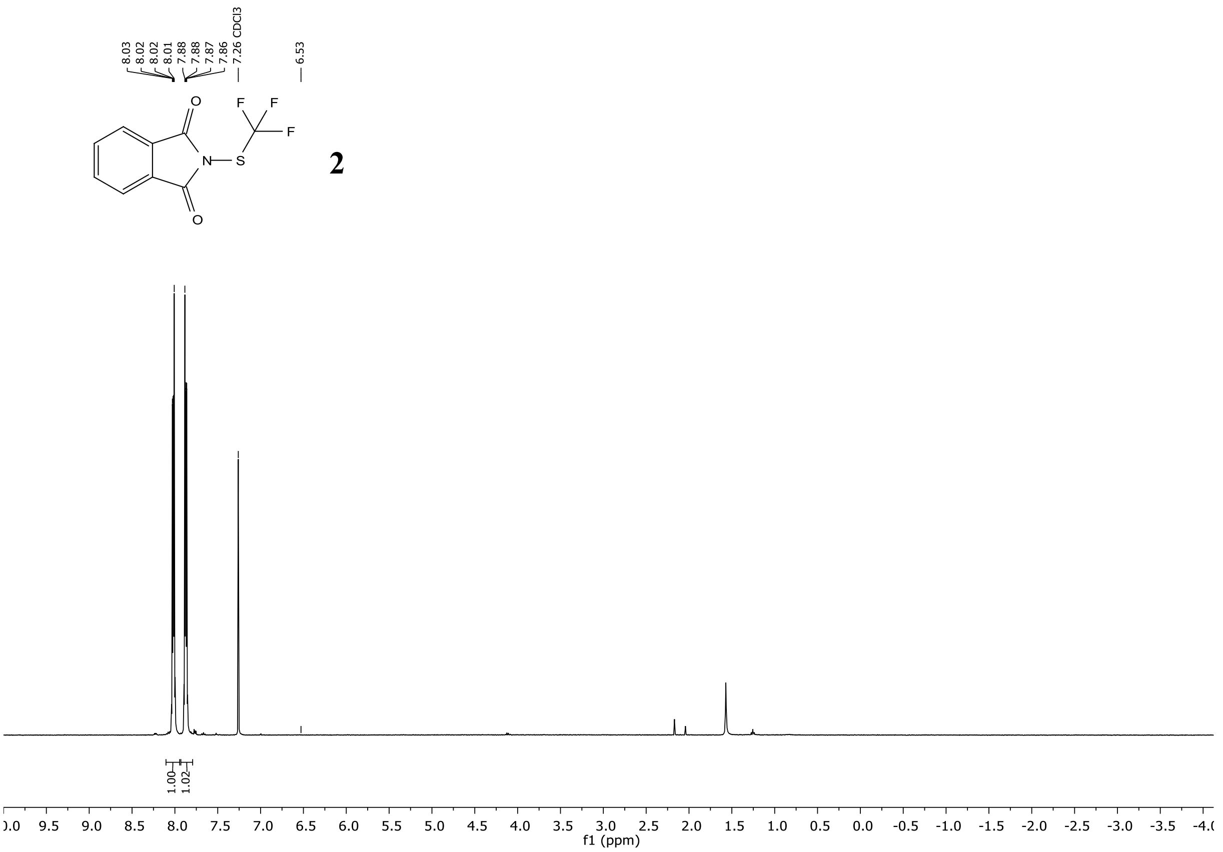

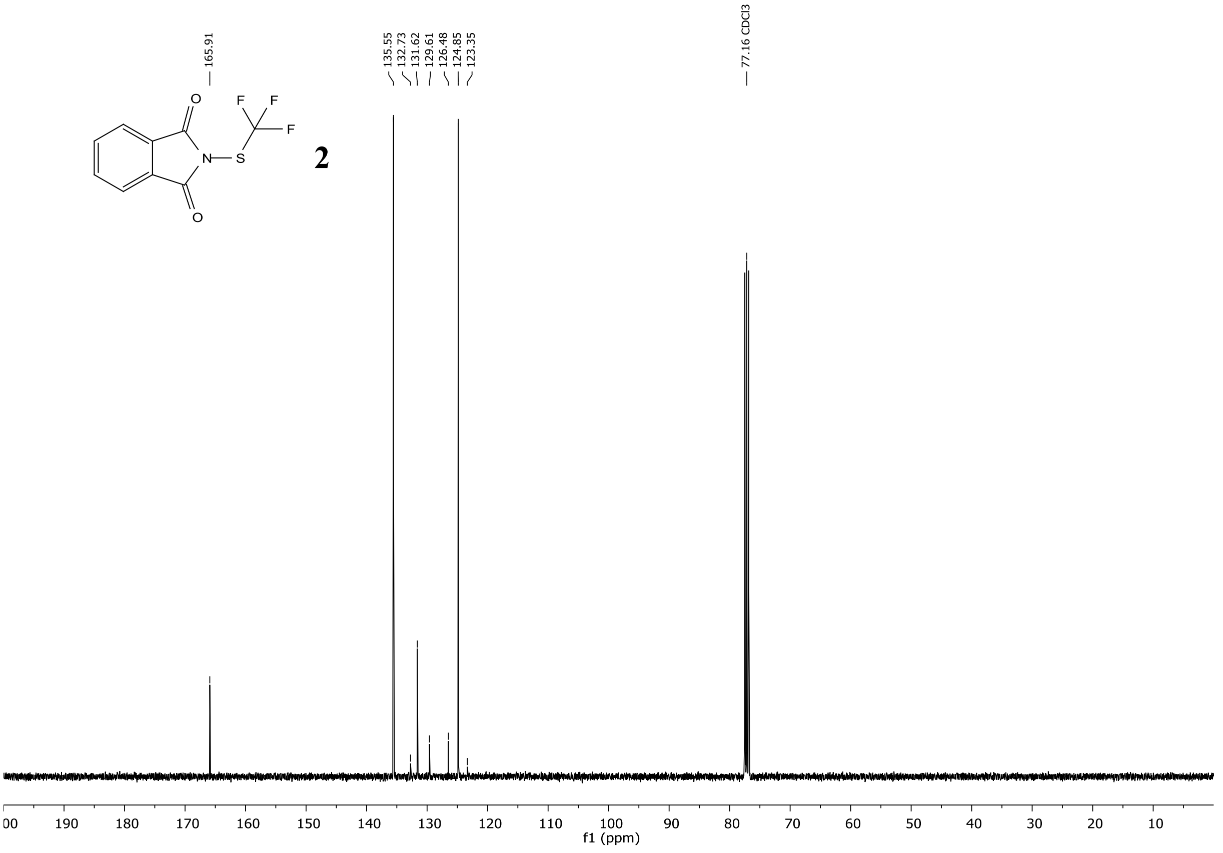

***N*-(*tert*-butyl)-*N′*-((trifluoromethyl)thio)benzimidamide (3a)**

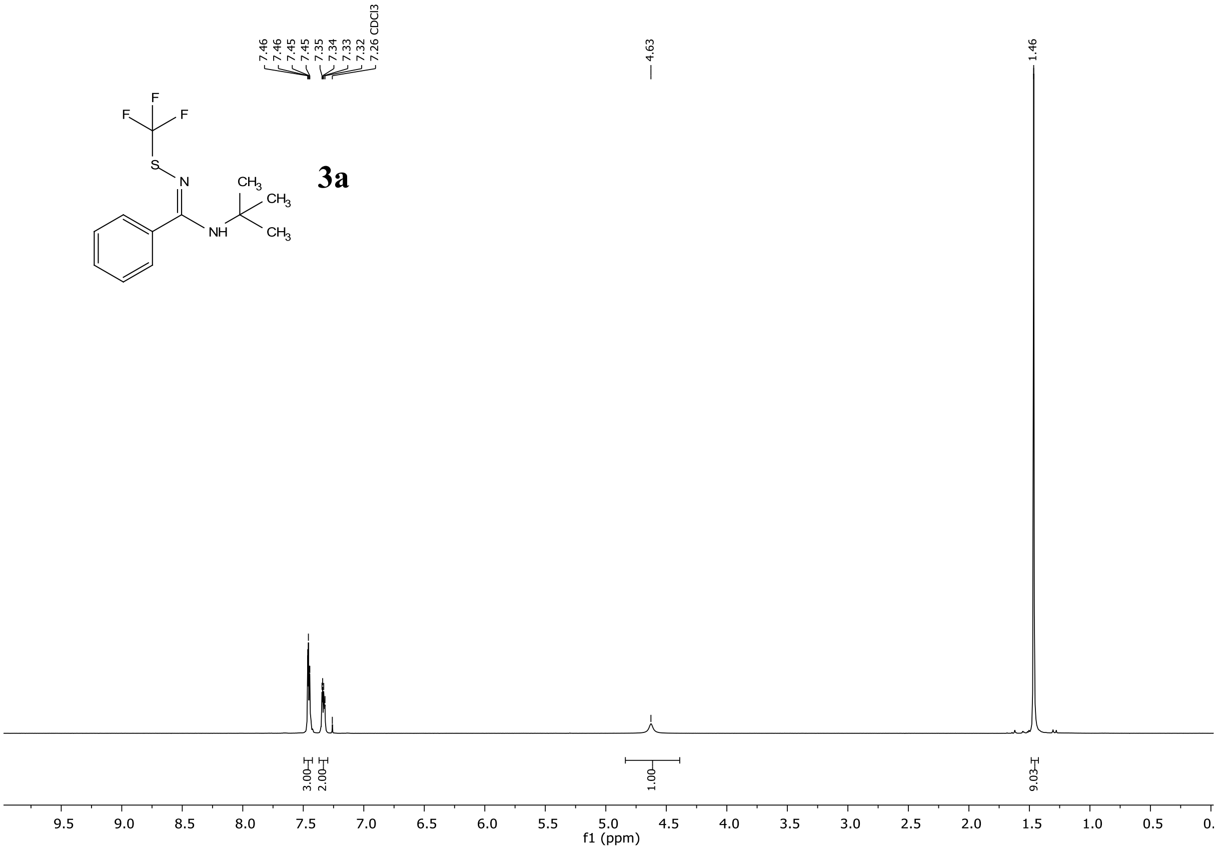

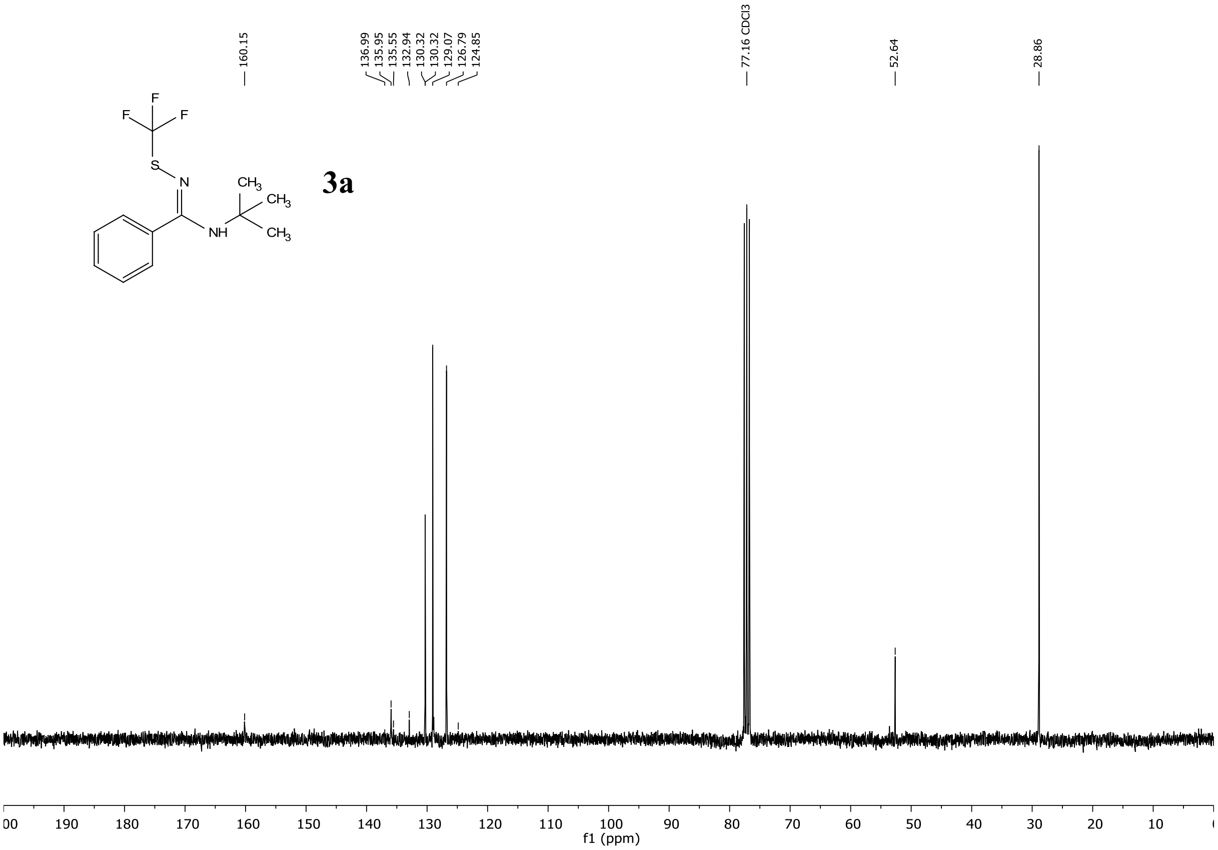

N-(*tert*-butyl)-4-methoxy-*N'*-((trifluoromethyl)thio)benzimidamide (3b)

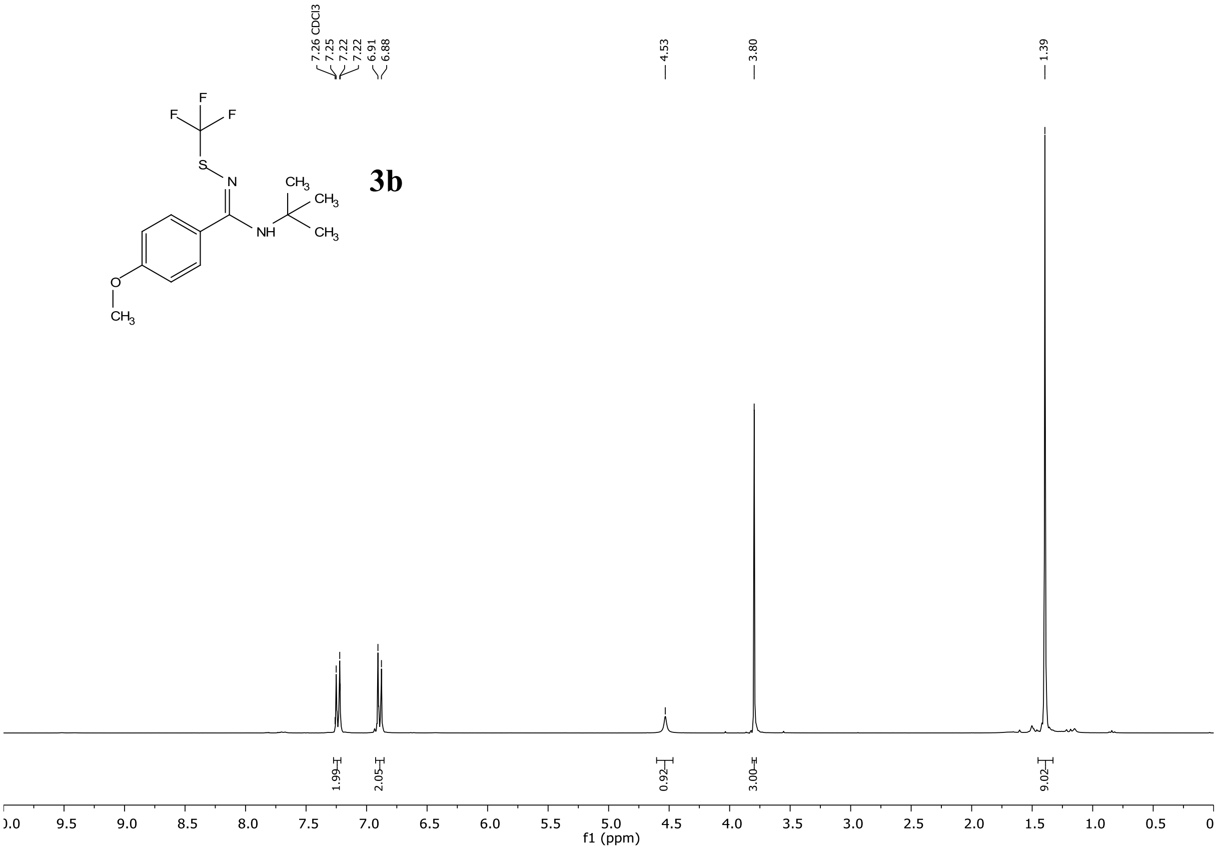

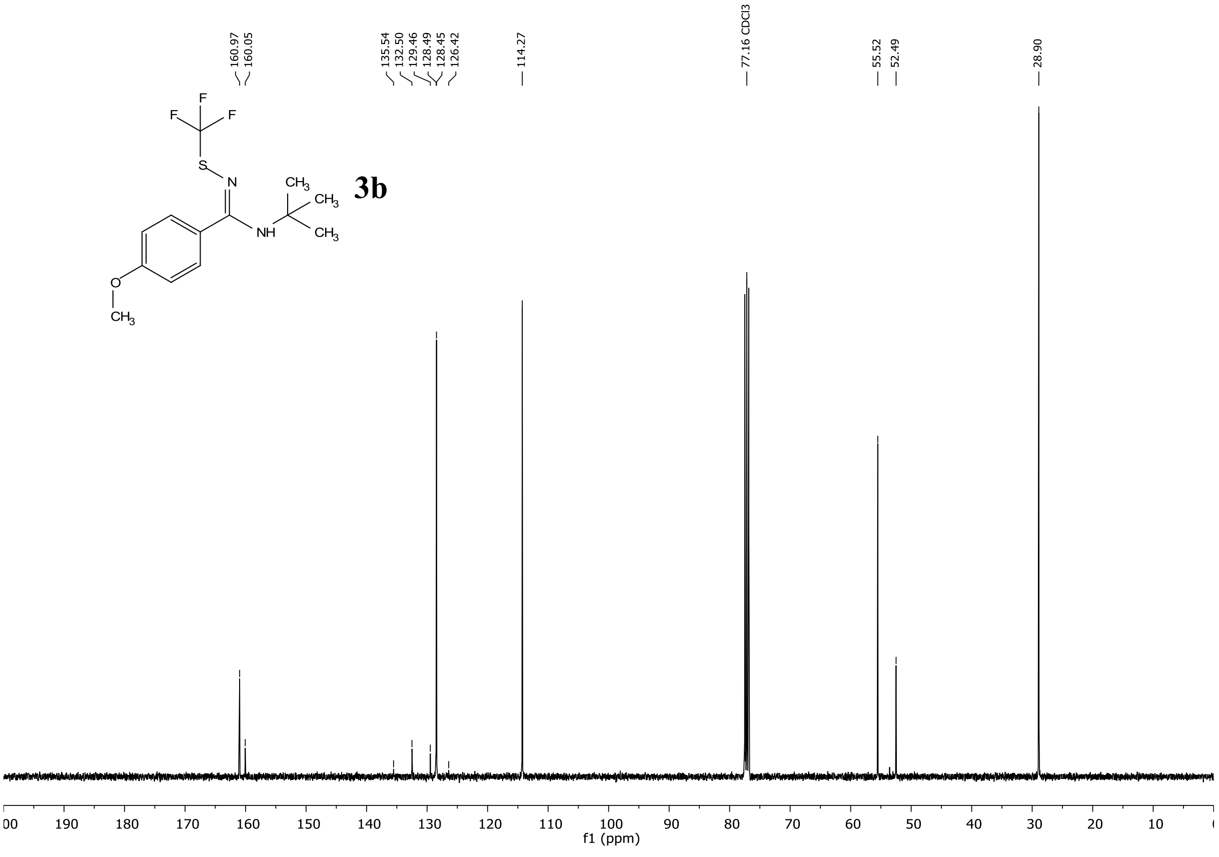

N-(tert-butyl)-*N*-ethyl-*N'*-((trifluoromethyl)thio)benzimidamide (3c)

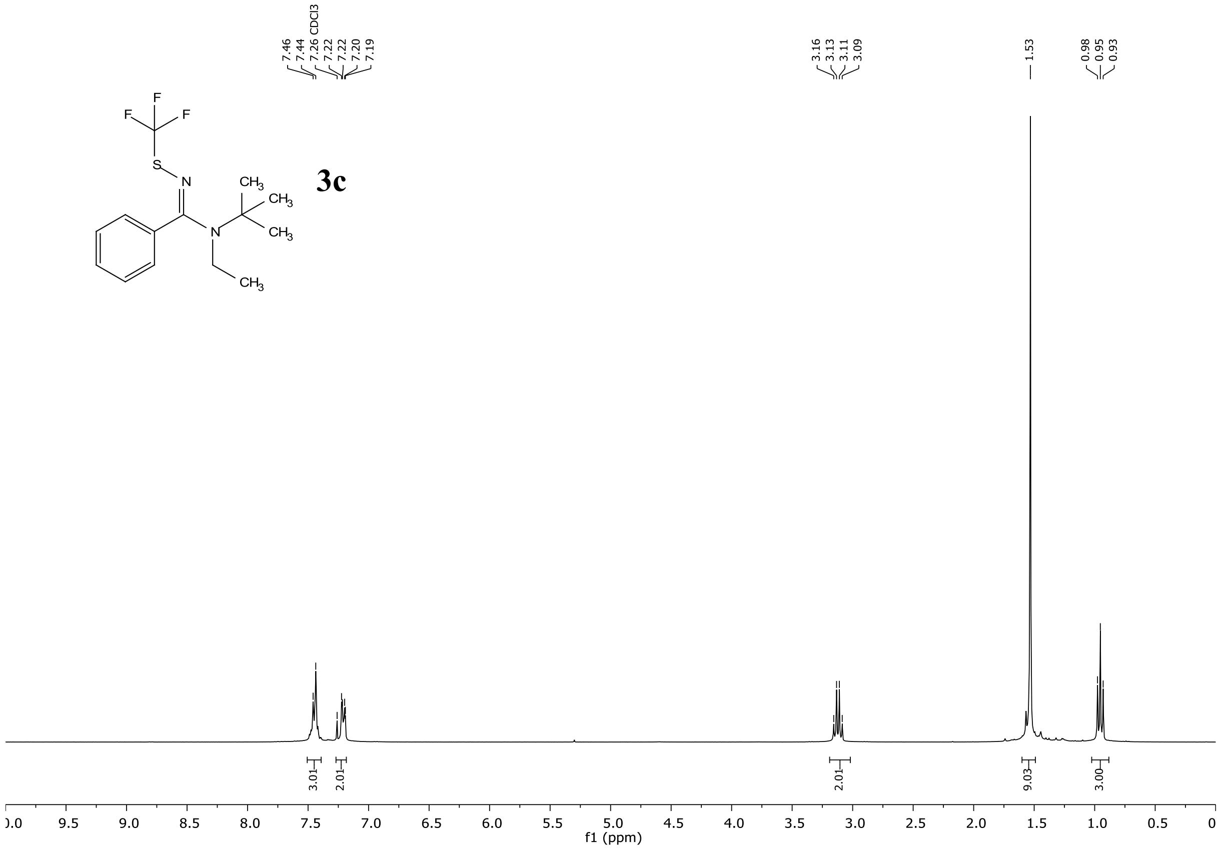

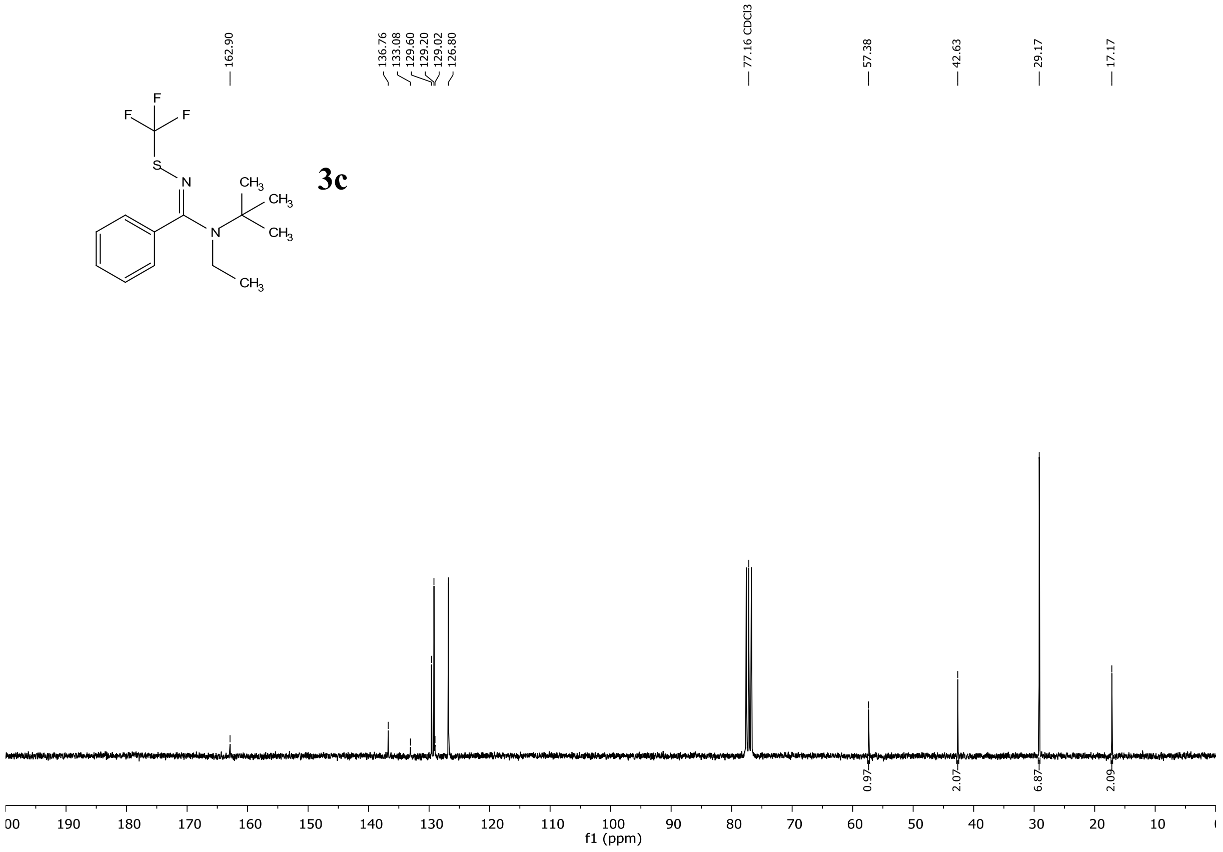

(*N*-(tert-butyl)-*N′*-((trifluoromethyl)thio)isobutyrimidamide (3d)

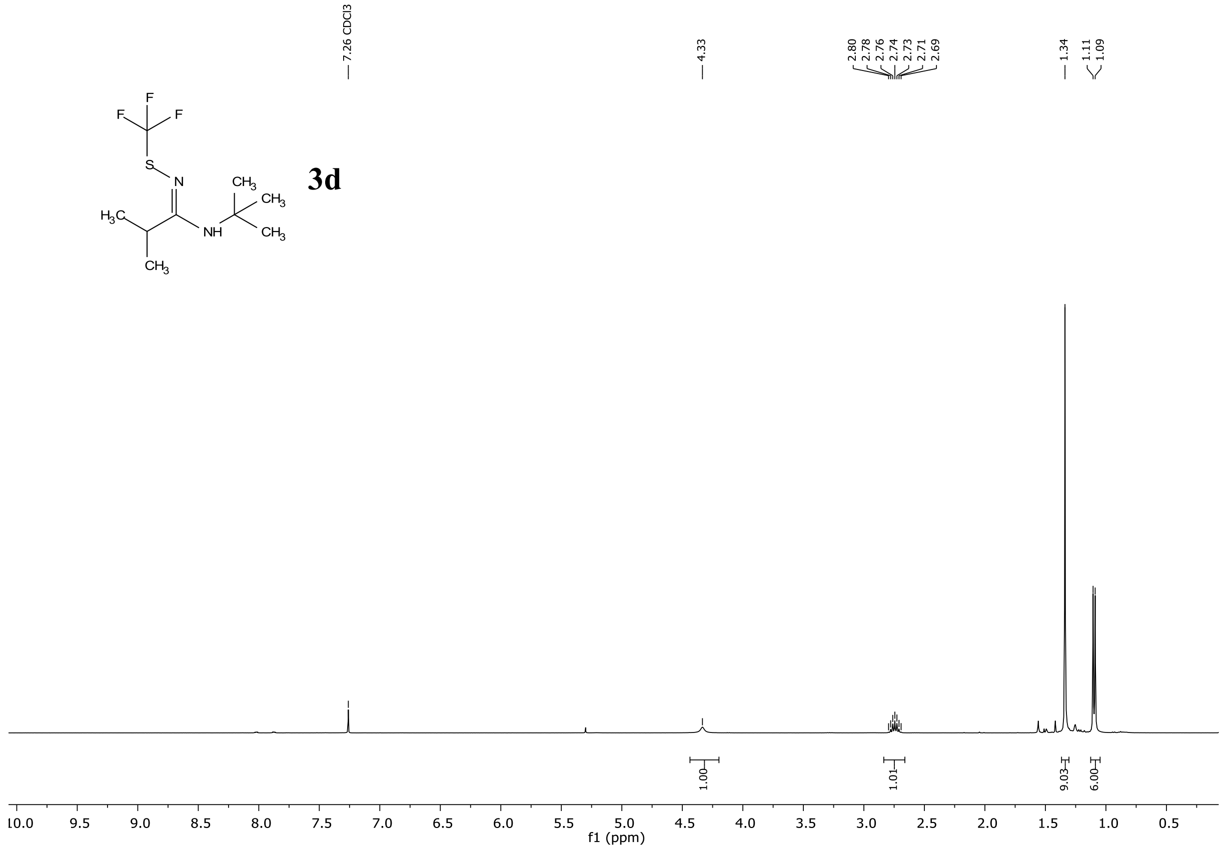

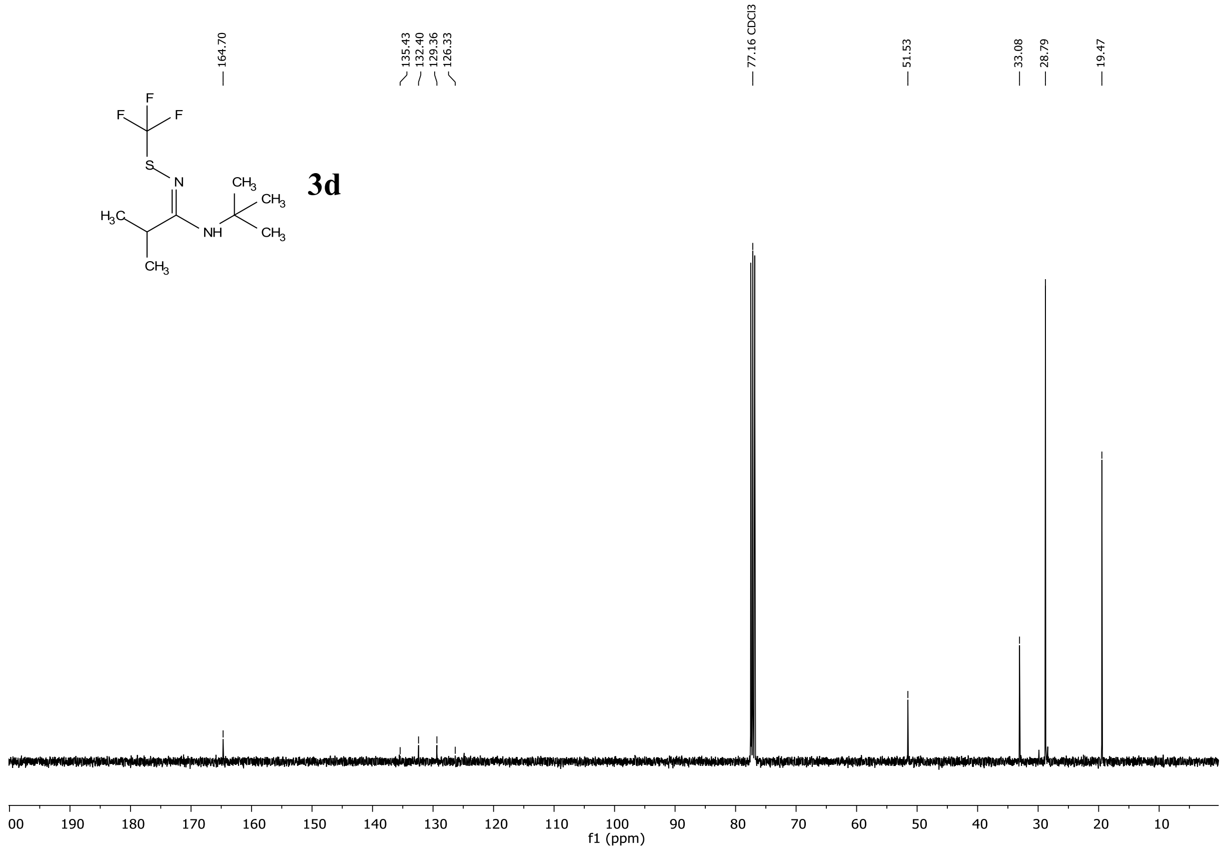

N,N-diethyl-*N'*-((trifluoromethyl)thio)benzimidamide (3e)

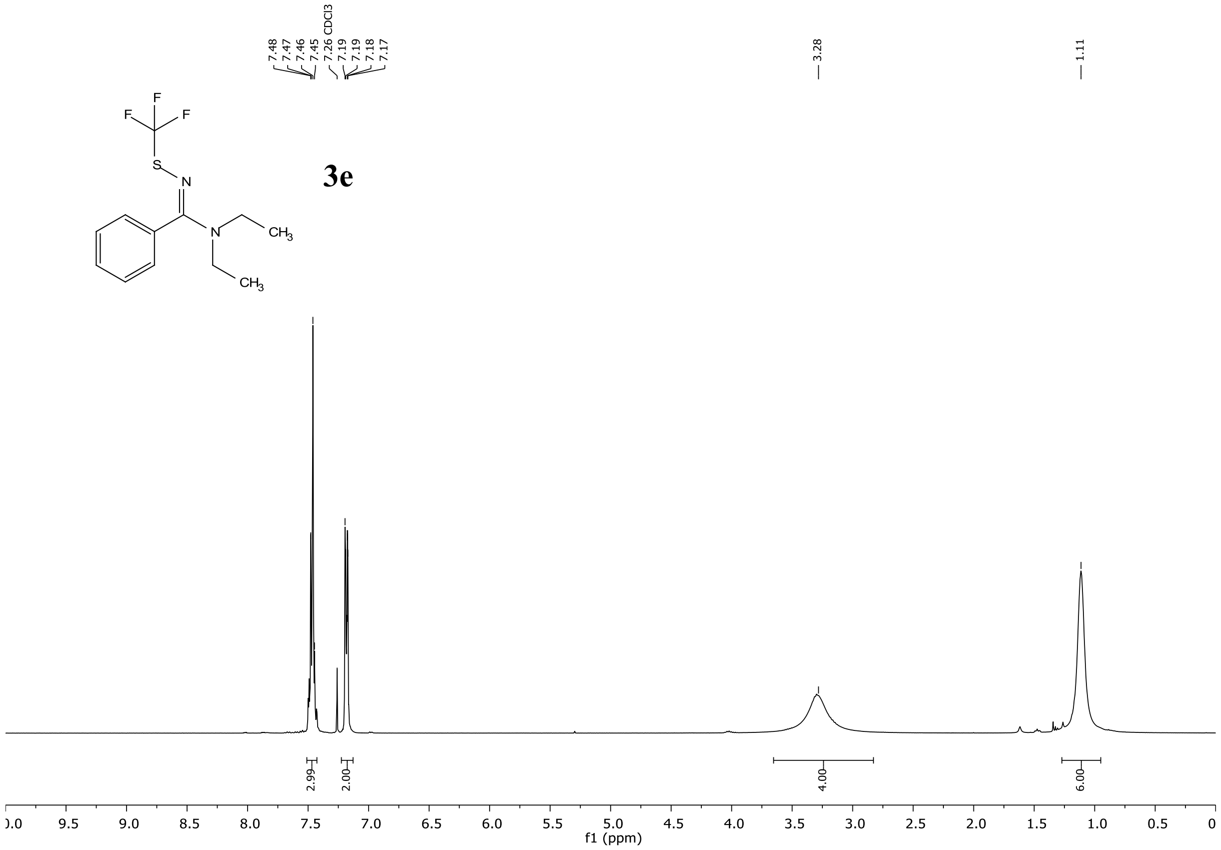

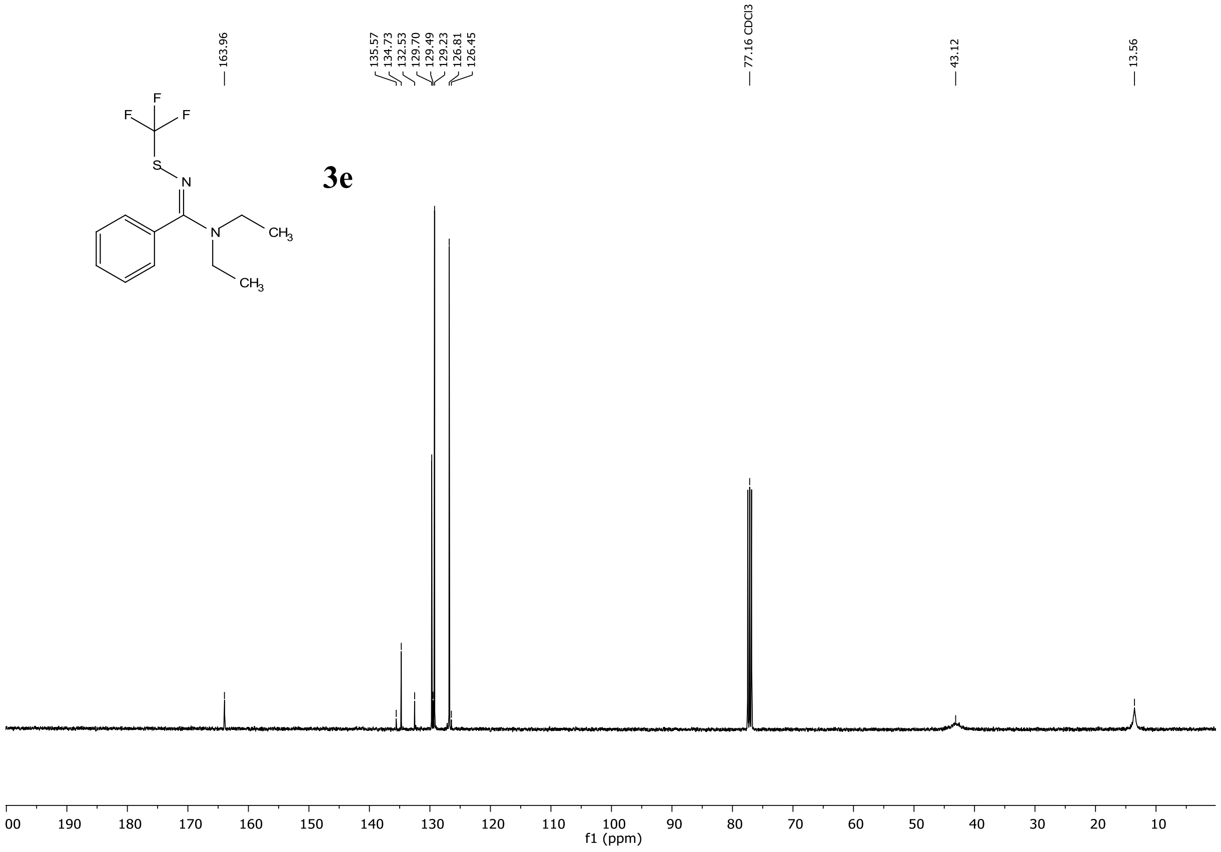

N-phenyl-*N′*-((trifluoromethyl)thio)benzimidamide (3f)

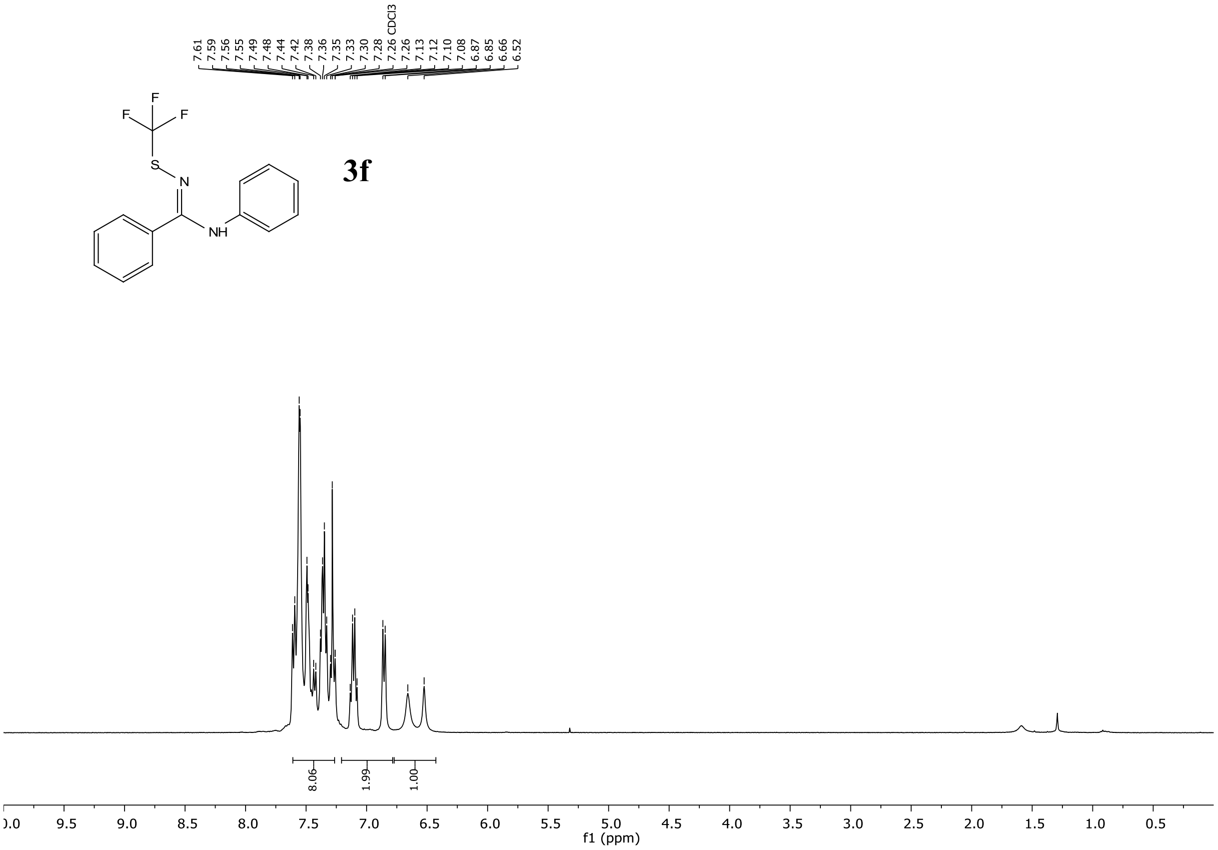

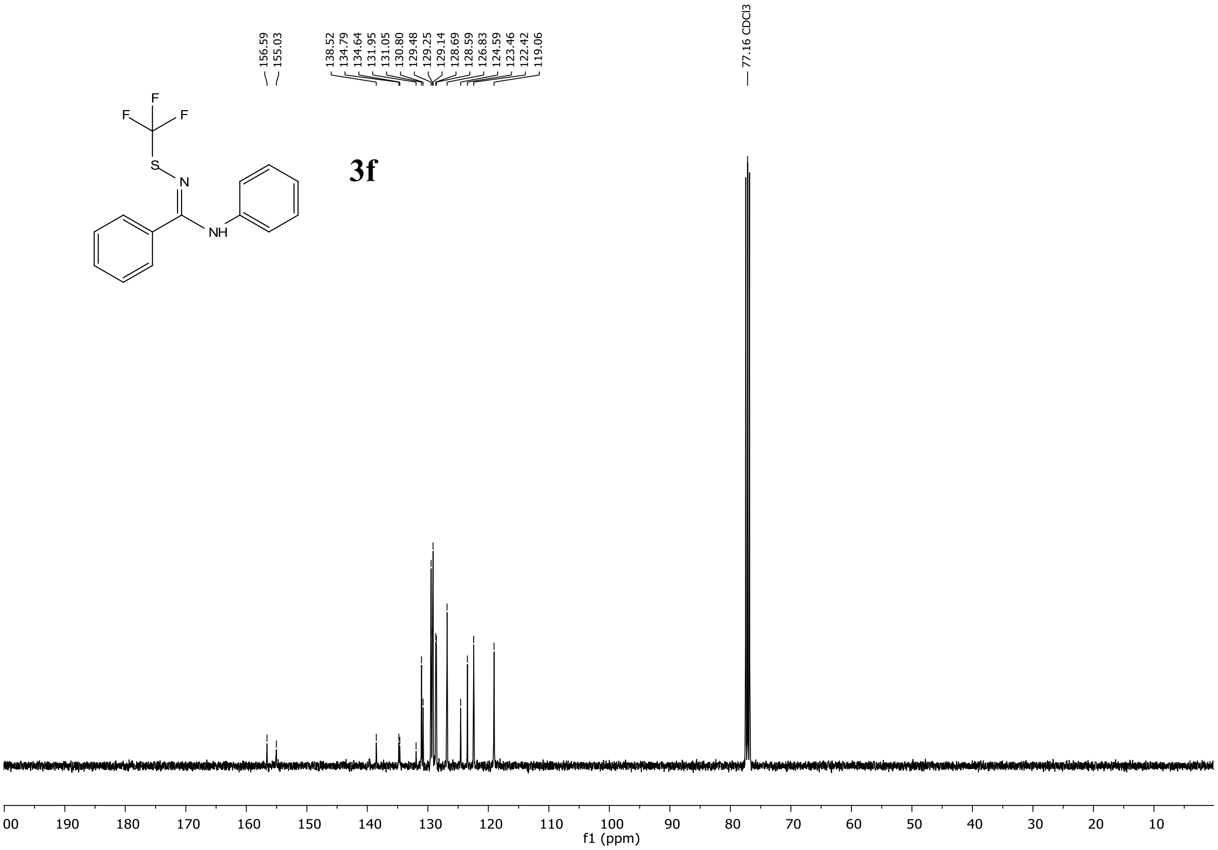

N-ethyl-4-methoxy-*N*-methyl-*N′*-((trifluoromethyl)thio)benzimidamide (3g)

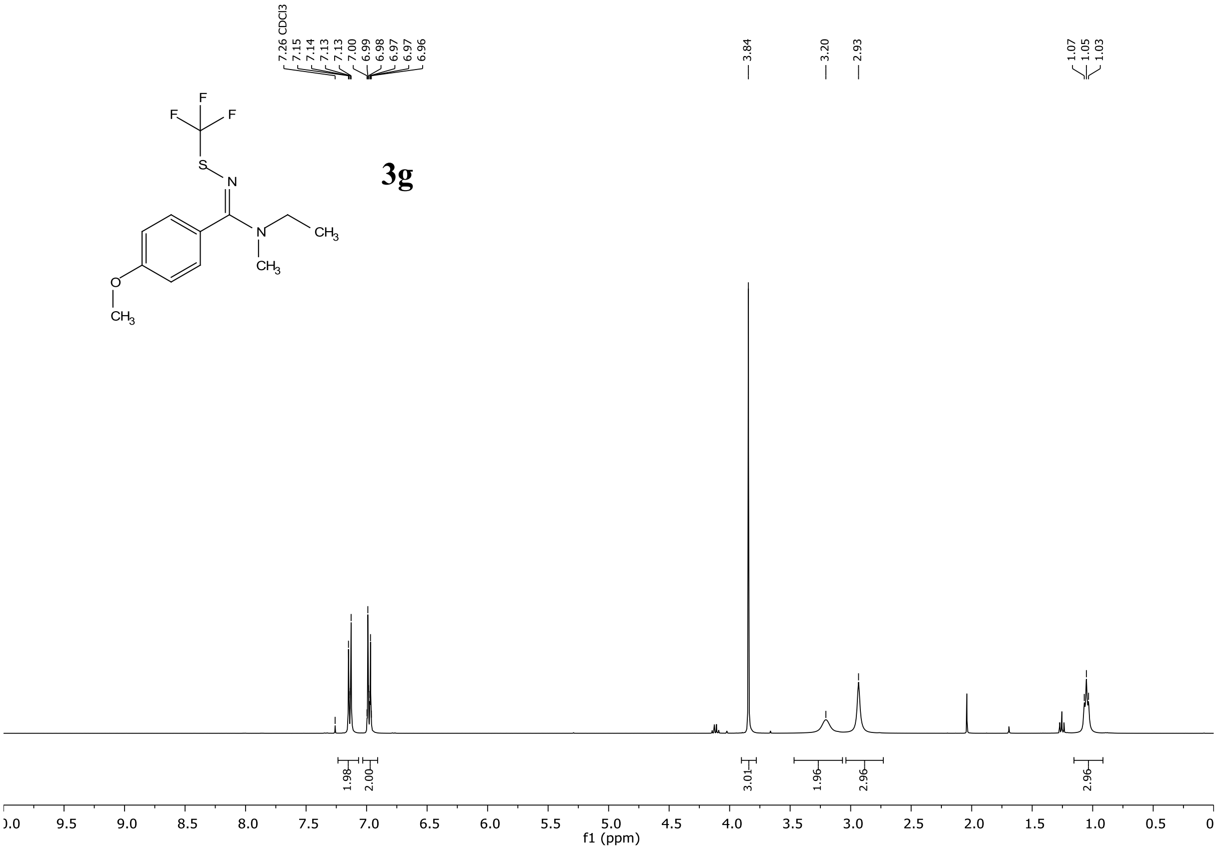

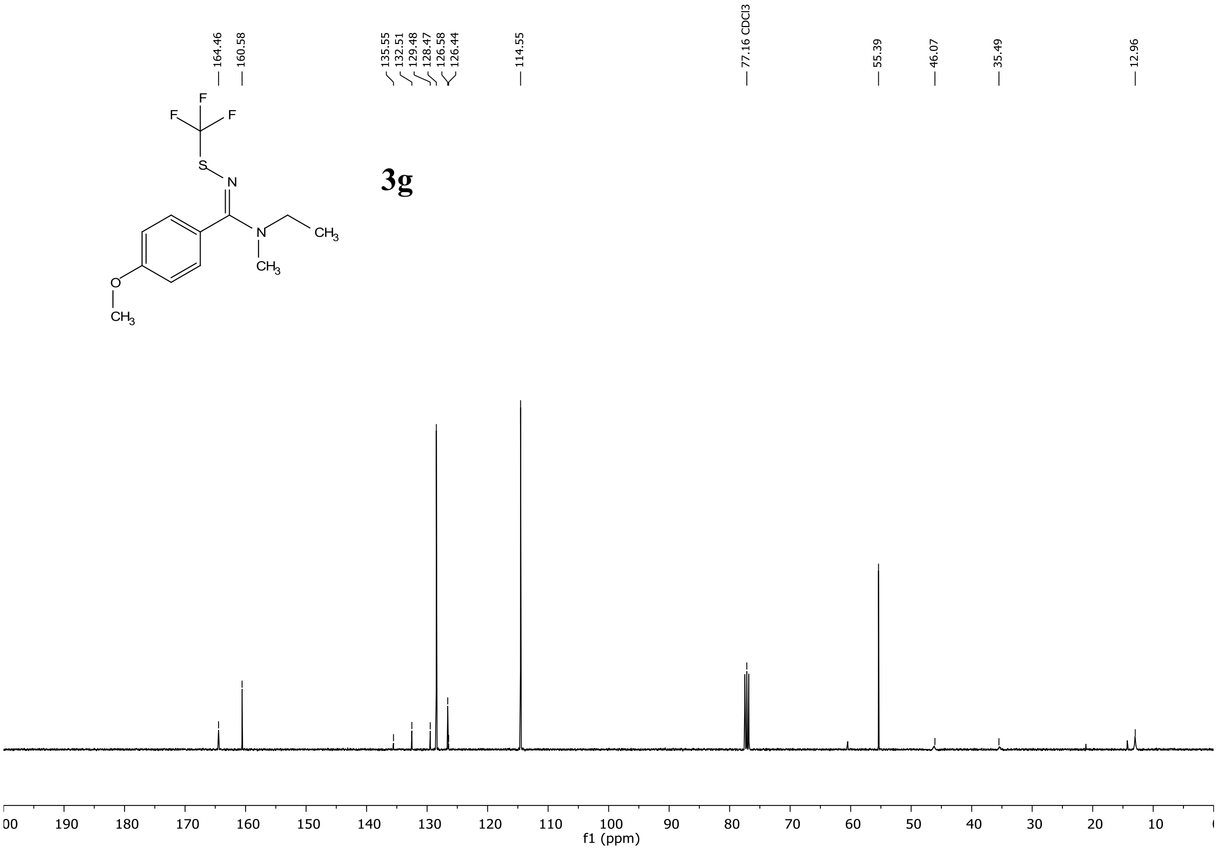

Methyl octa-2,7-dienoate (4a)

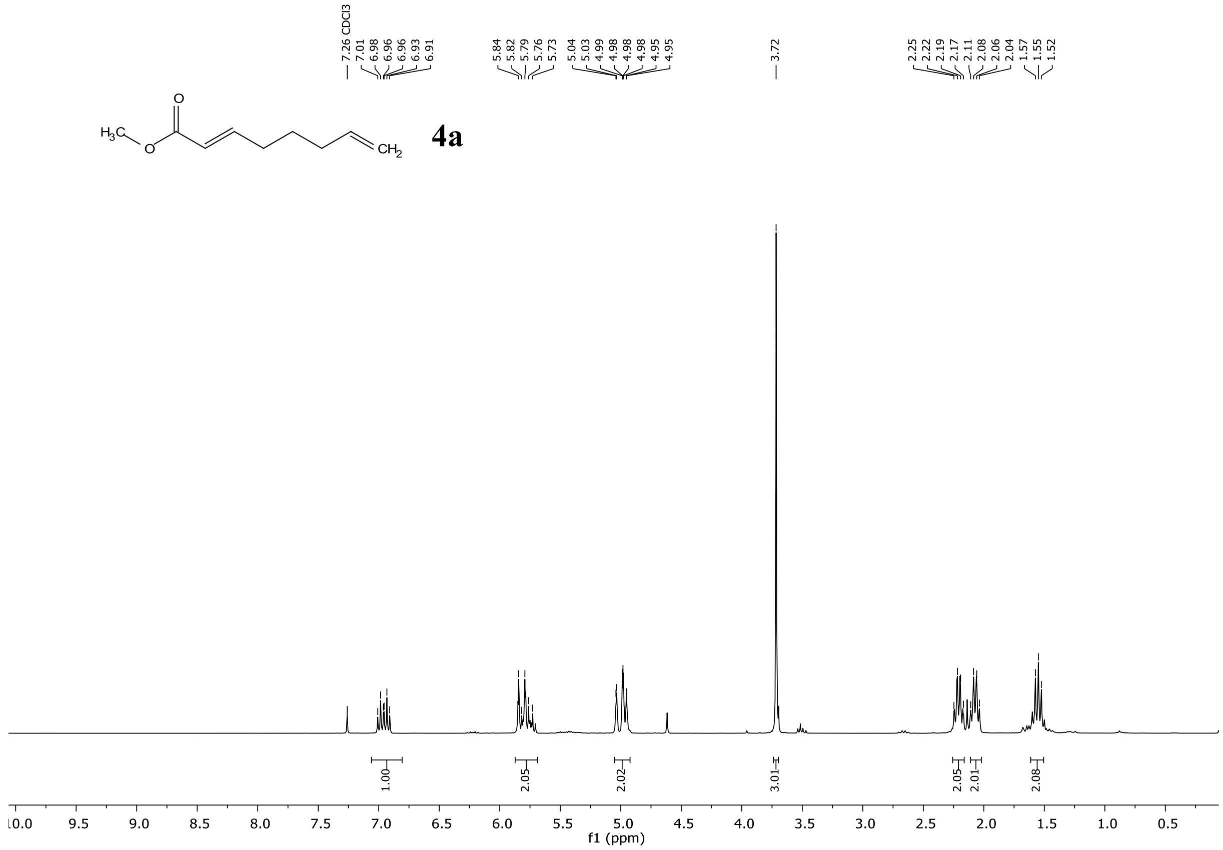

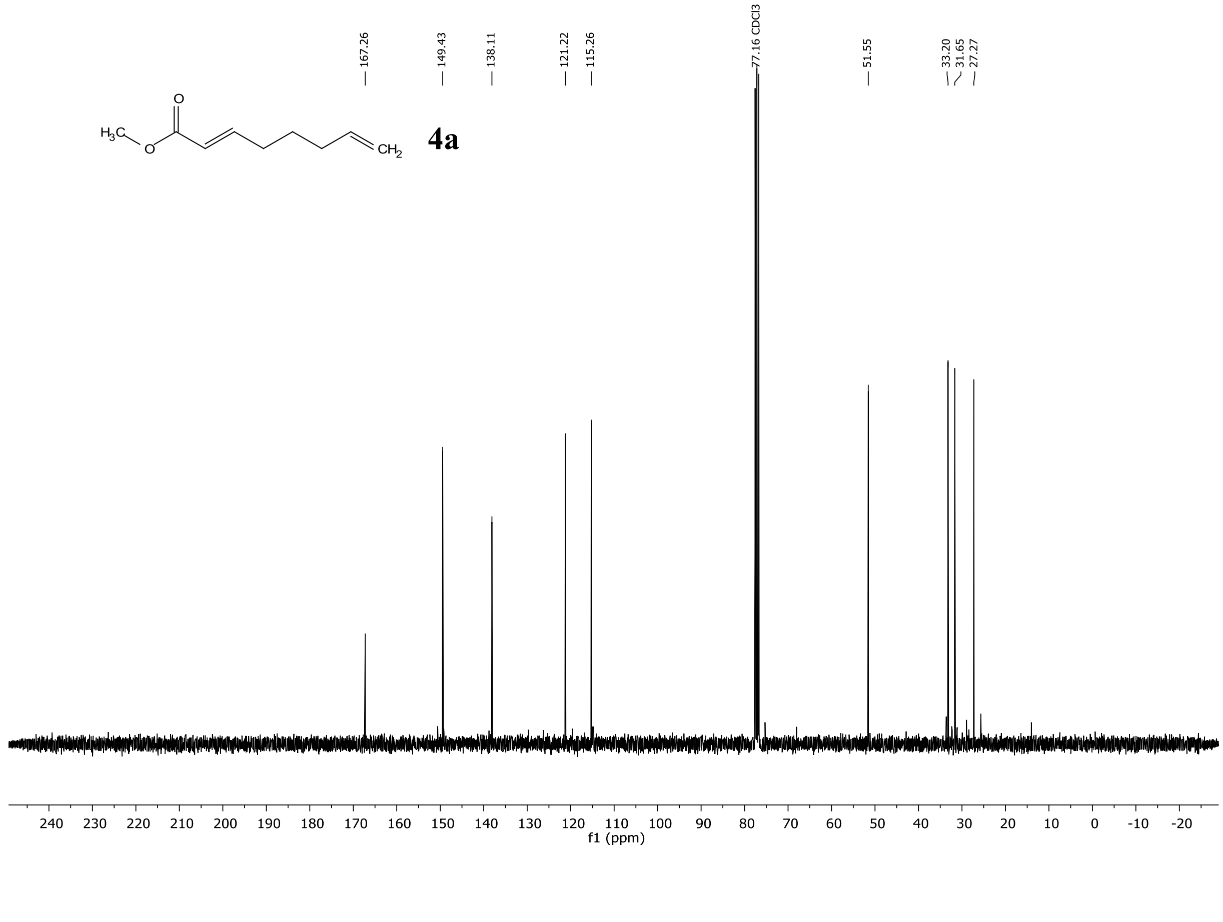

Methyl oct-2-en-7-ynoate (4b)

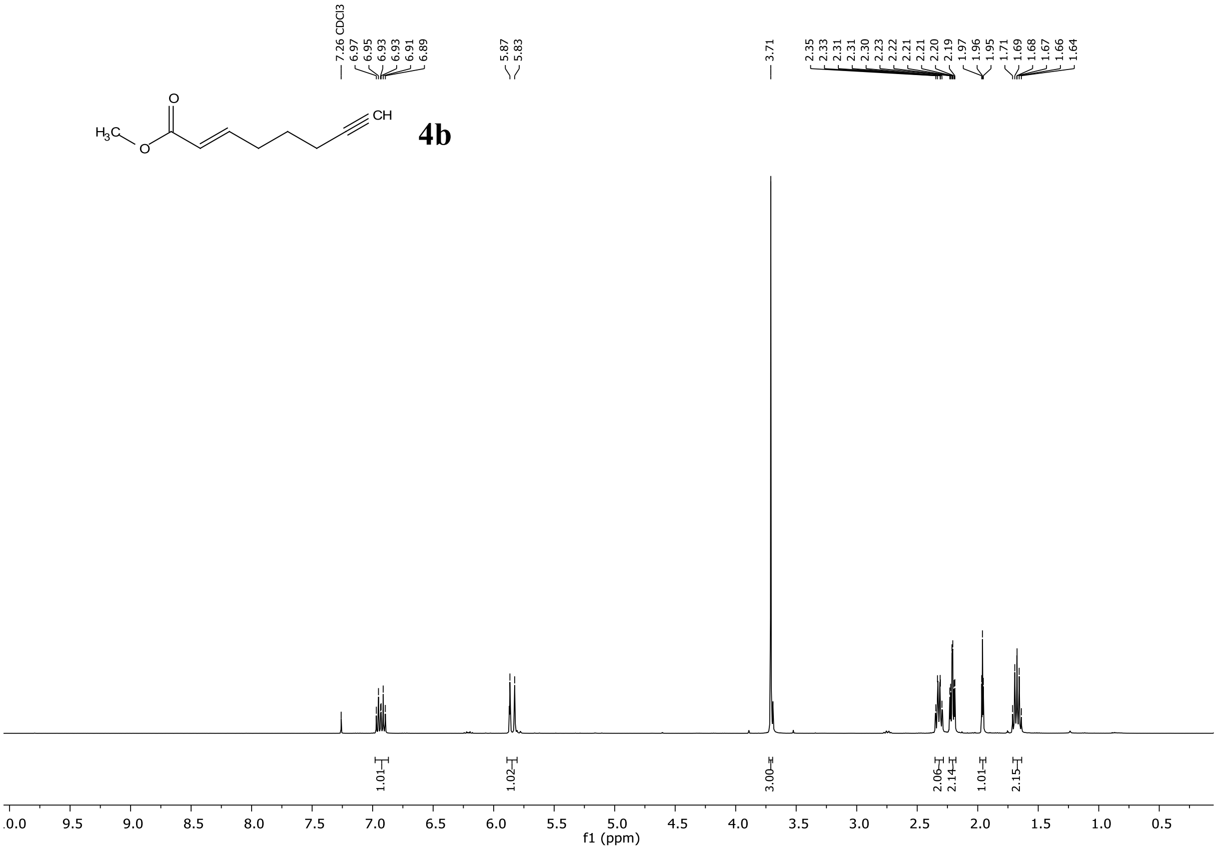

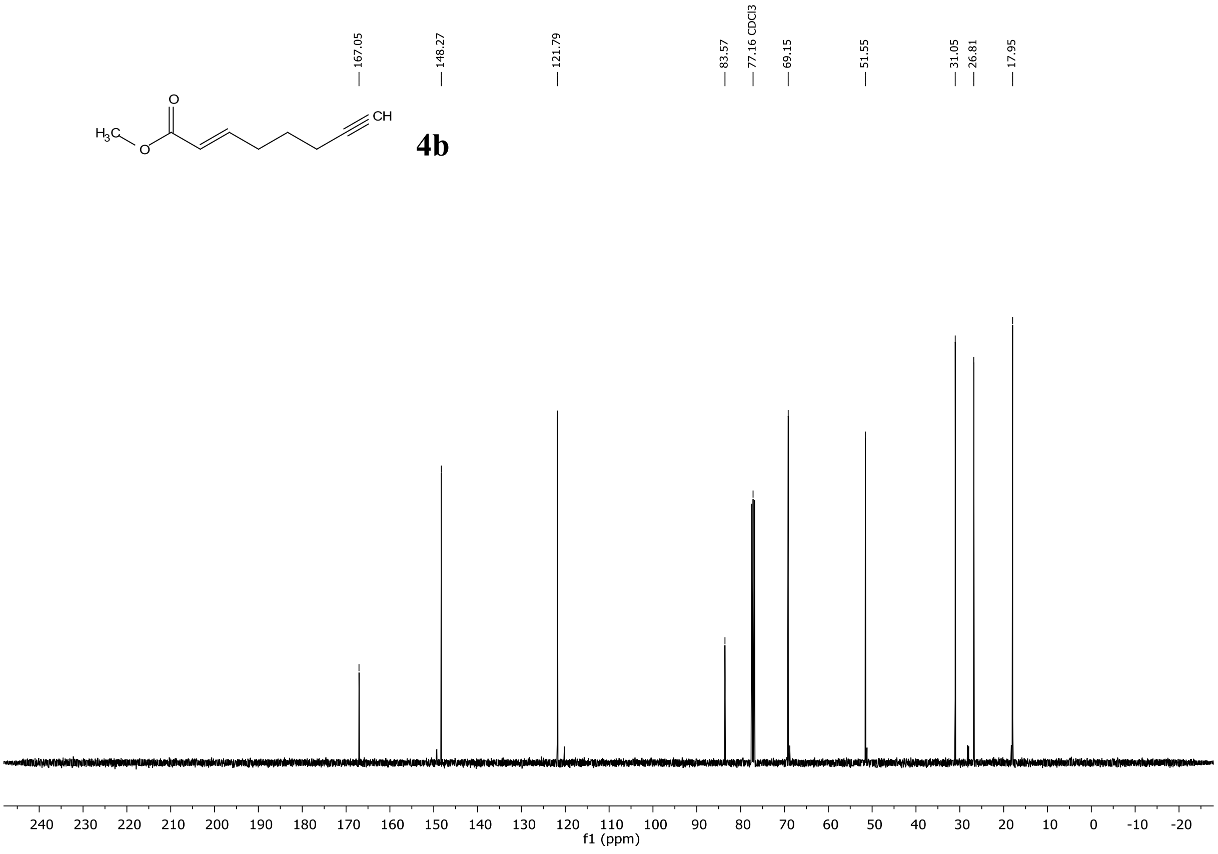

Methyl 2-(2-(((trifluoromethyl)thio)methyl)cyclopentyl)acetate (5a)

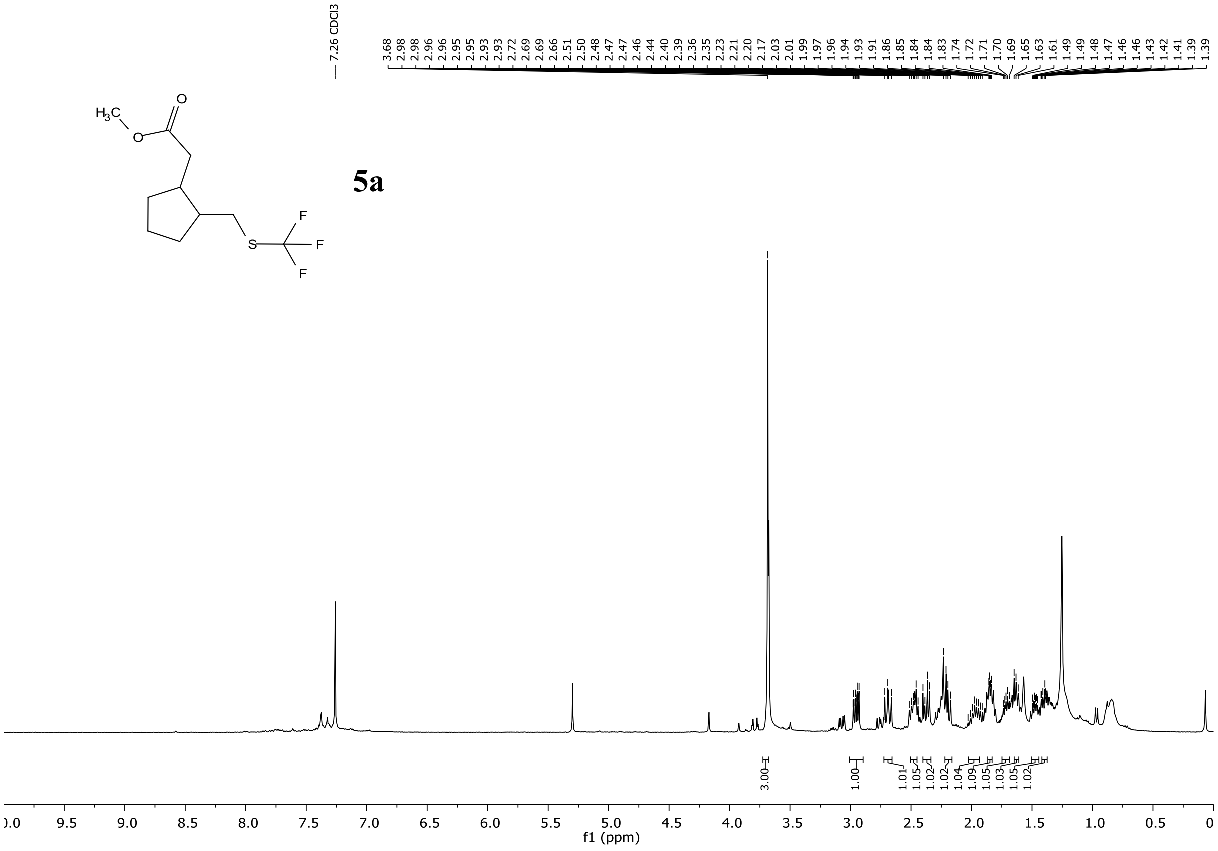

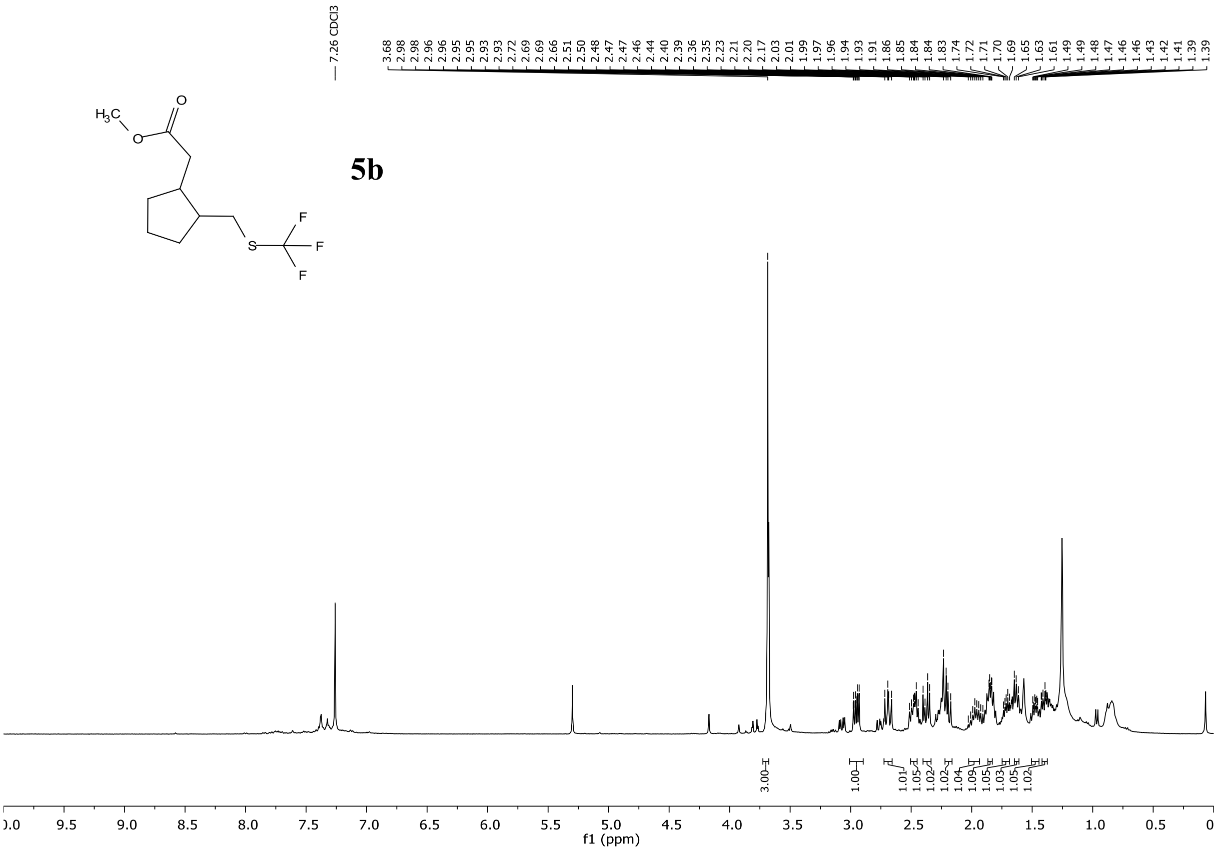

Methyl 2-(2-(((trifluoromethyl)thio)methylene)cyclopentyl)acetate (5b)

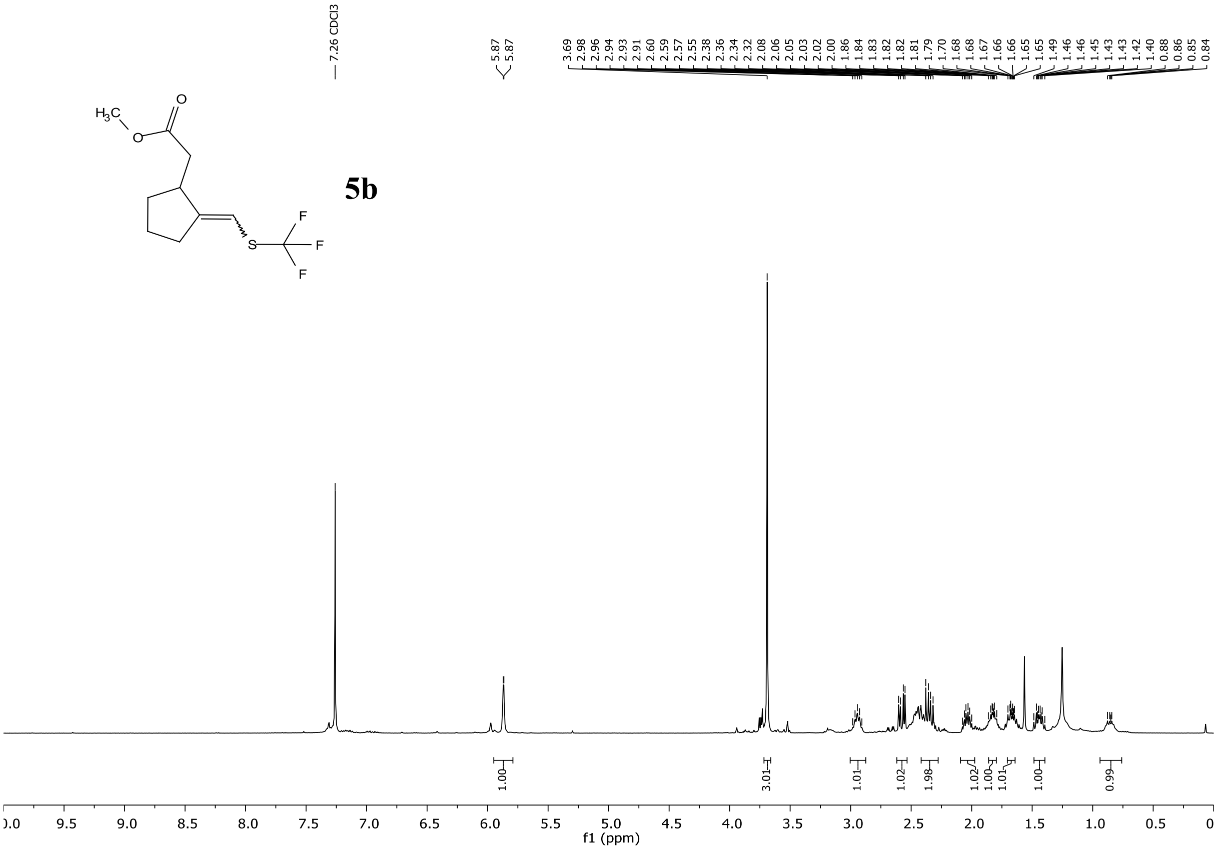

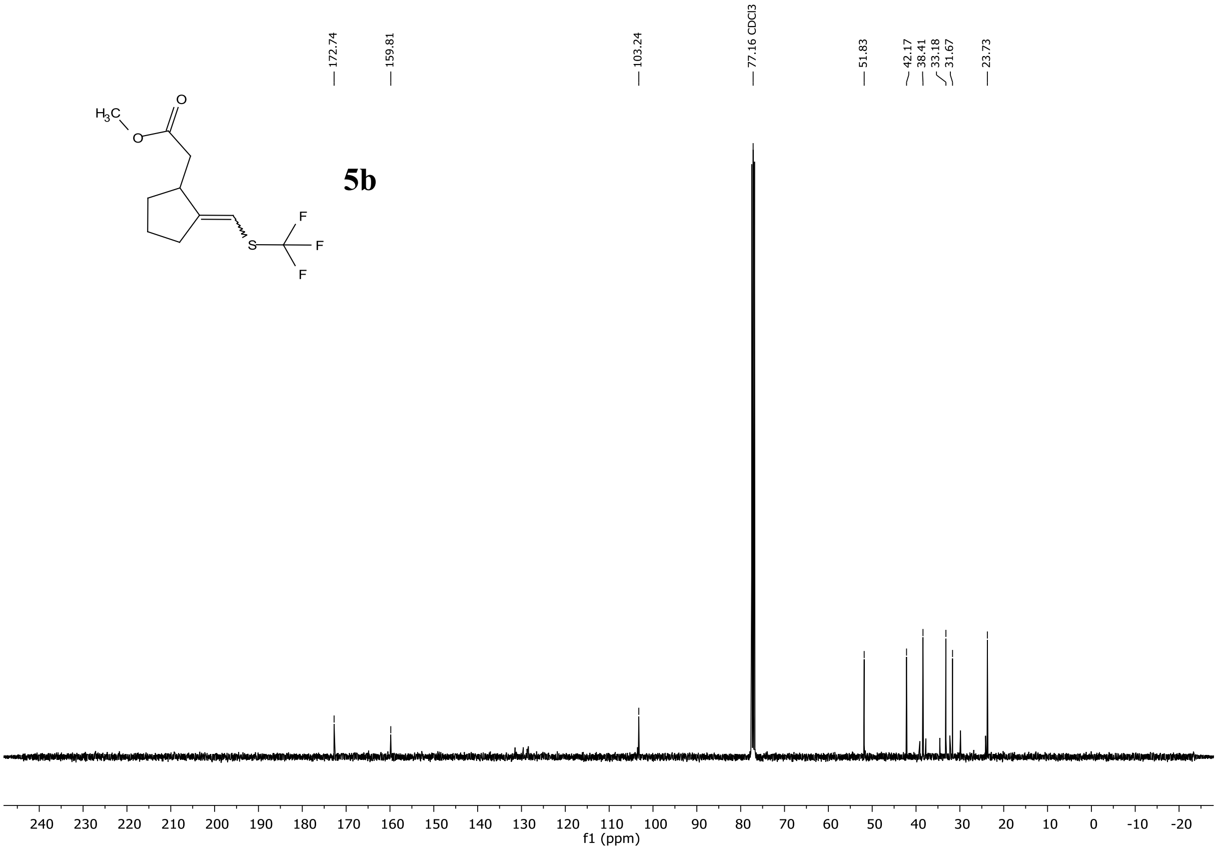

4-Methoxybenzonitril (6b)

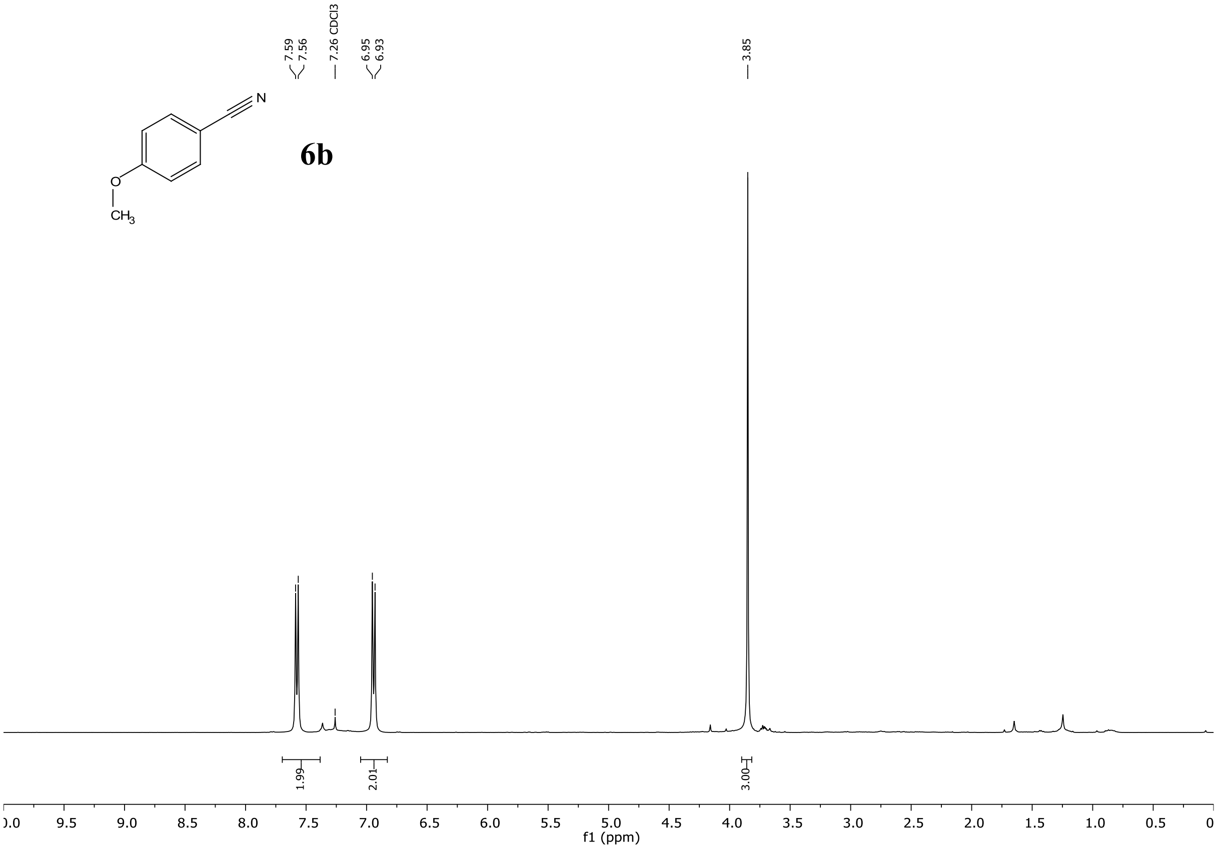

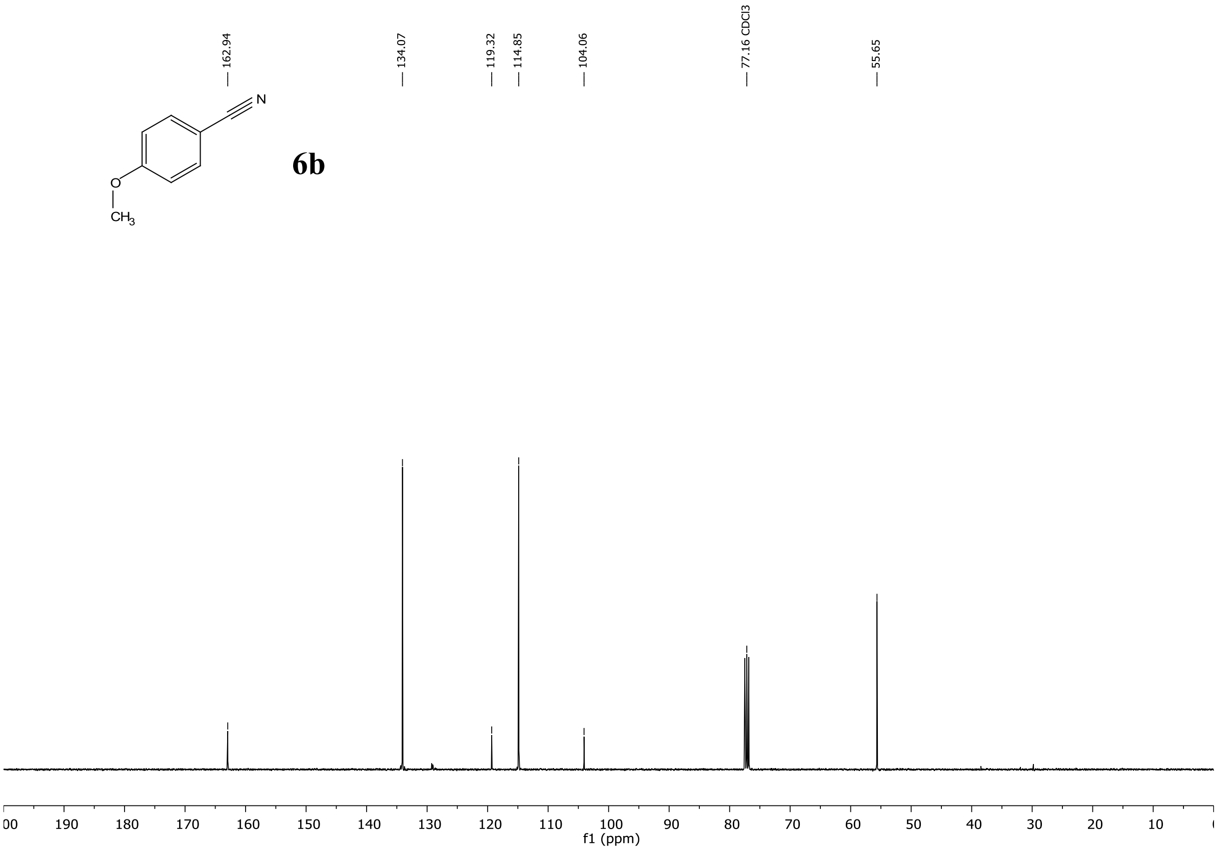

4,5-Dichloro-1-((perfluorophenyl)methyl)-imidazole (8m)

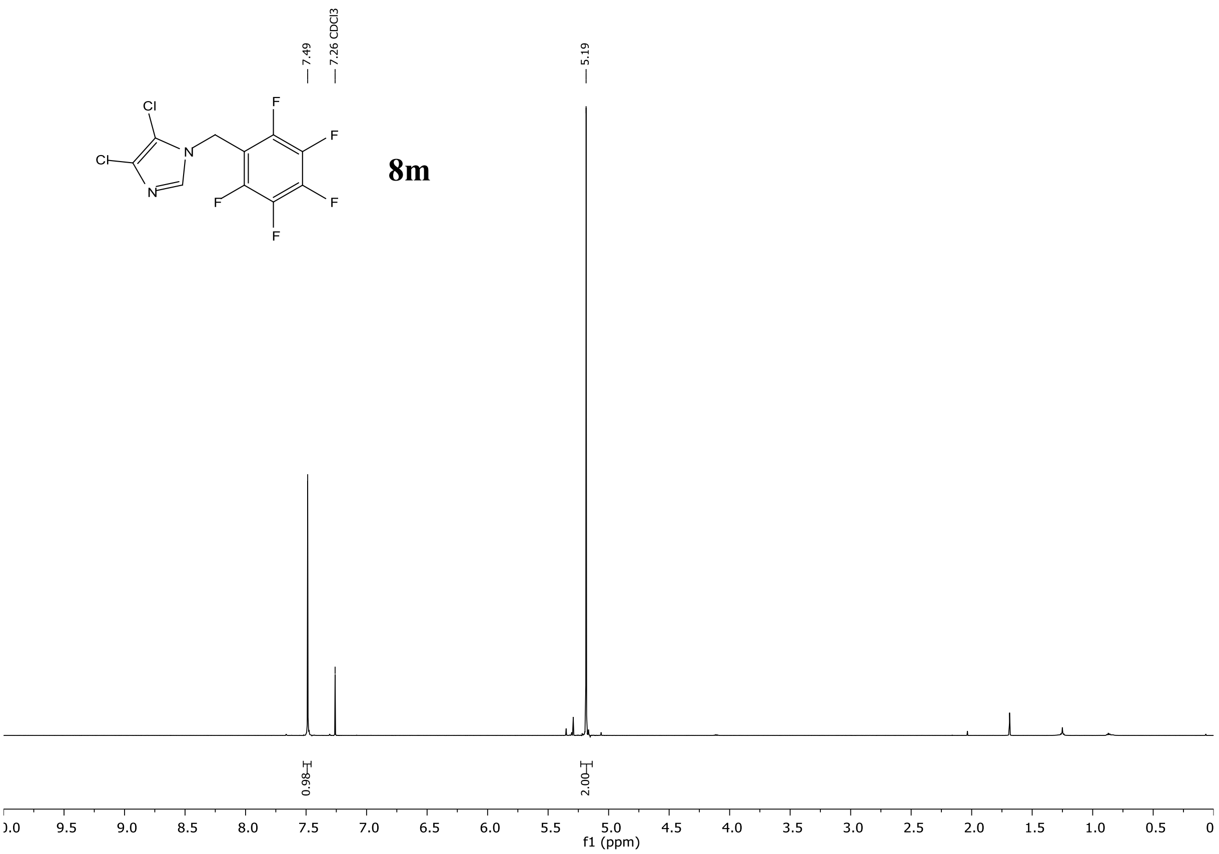

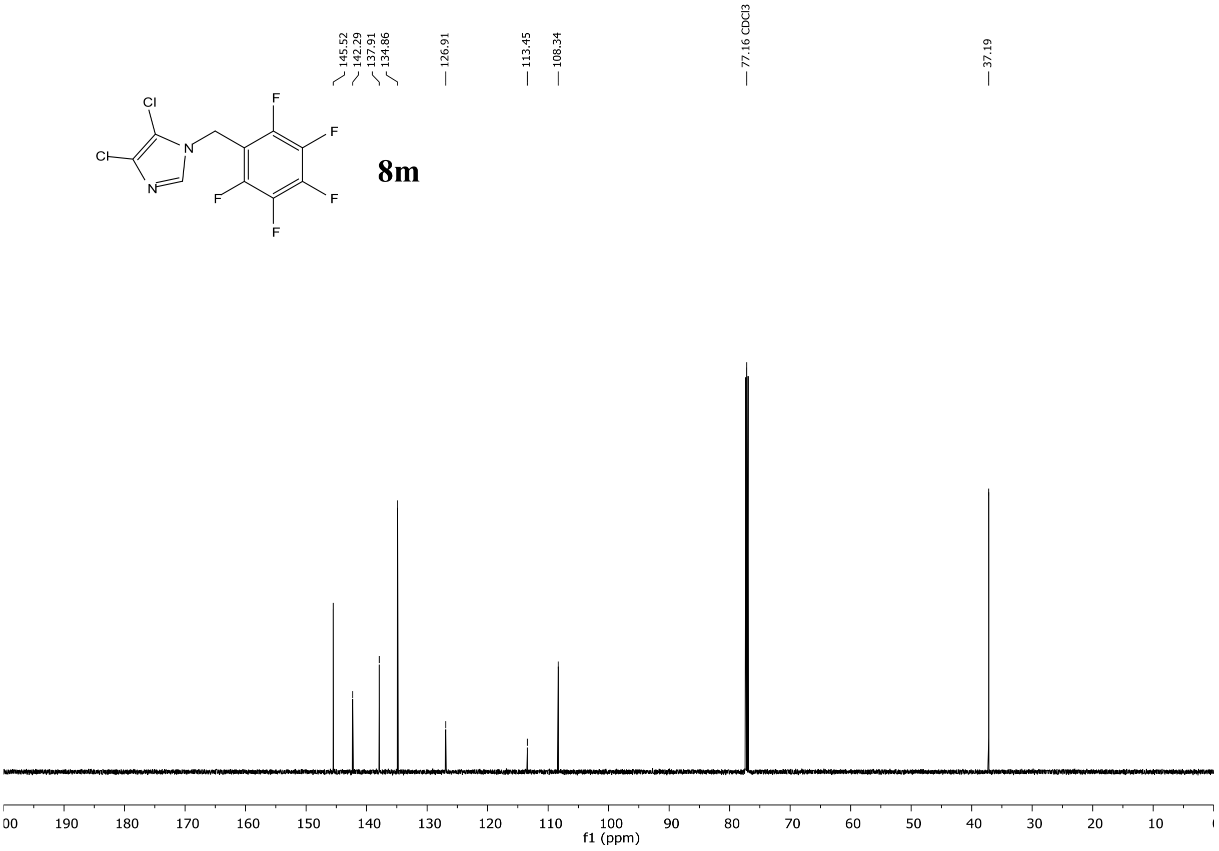

4,5-Dichloro-1-(4-((trifluoromethyl)thio)benzyl)-imidazole (8n)

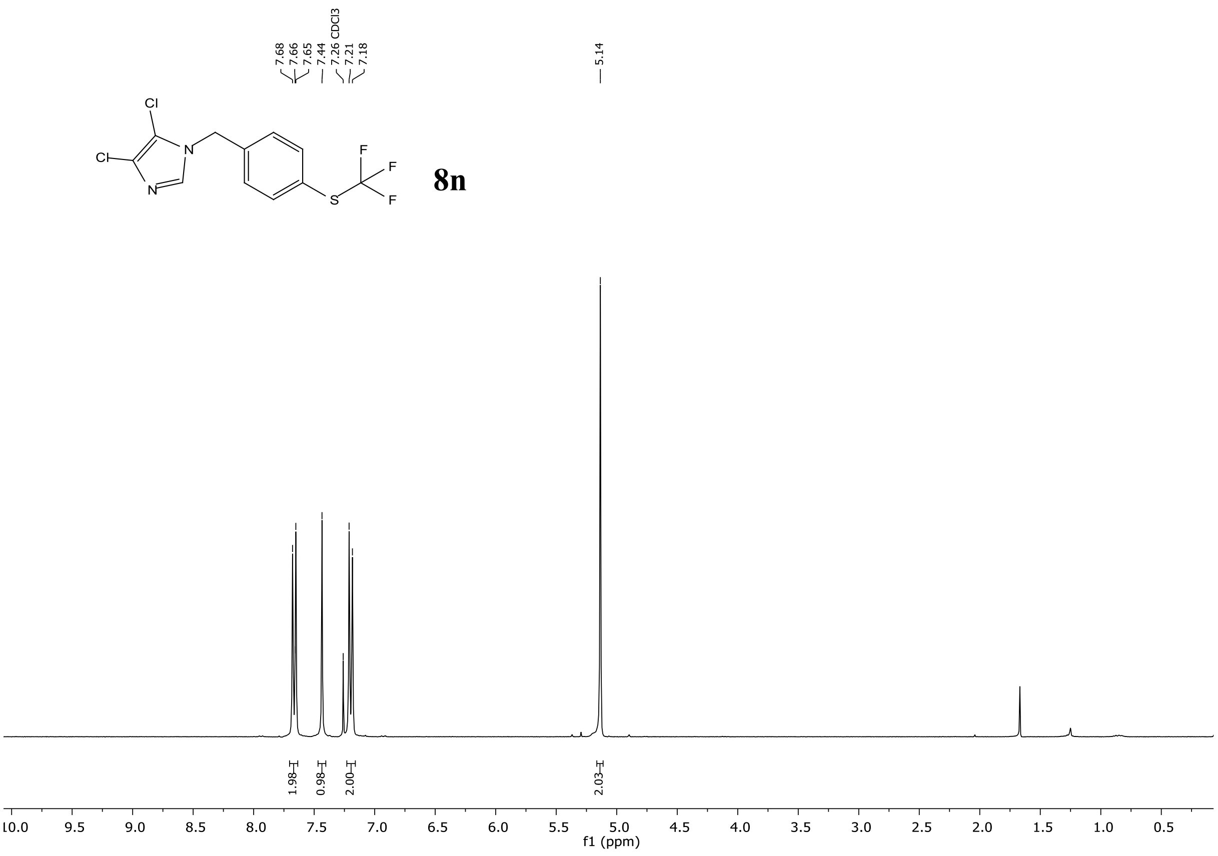

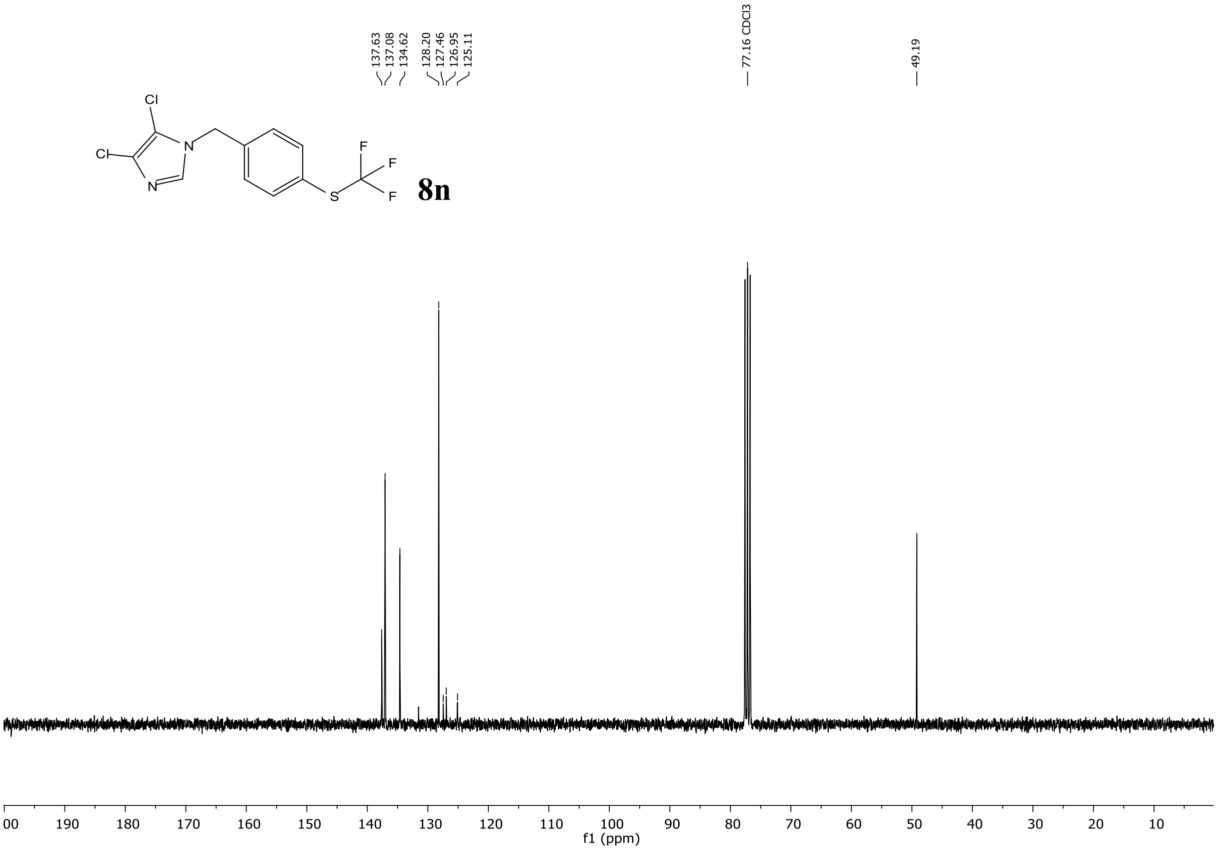

4,5-Dichloro-1-(3-methoxypropyl)-imidazole (8p)

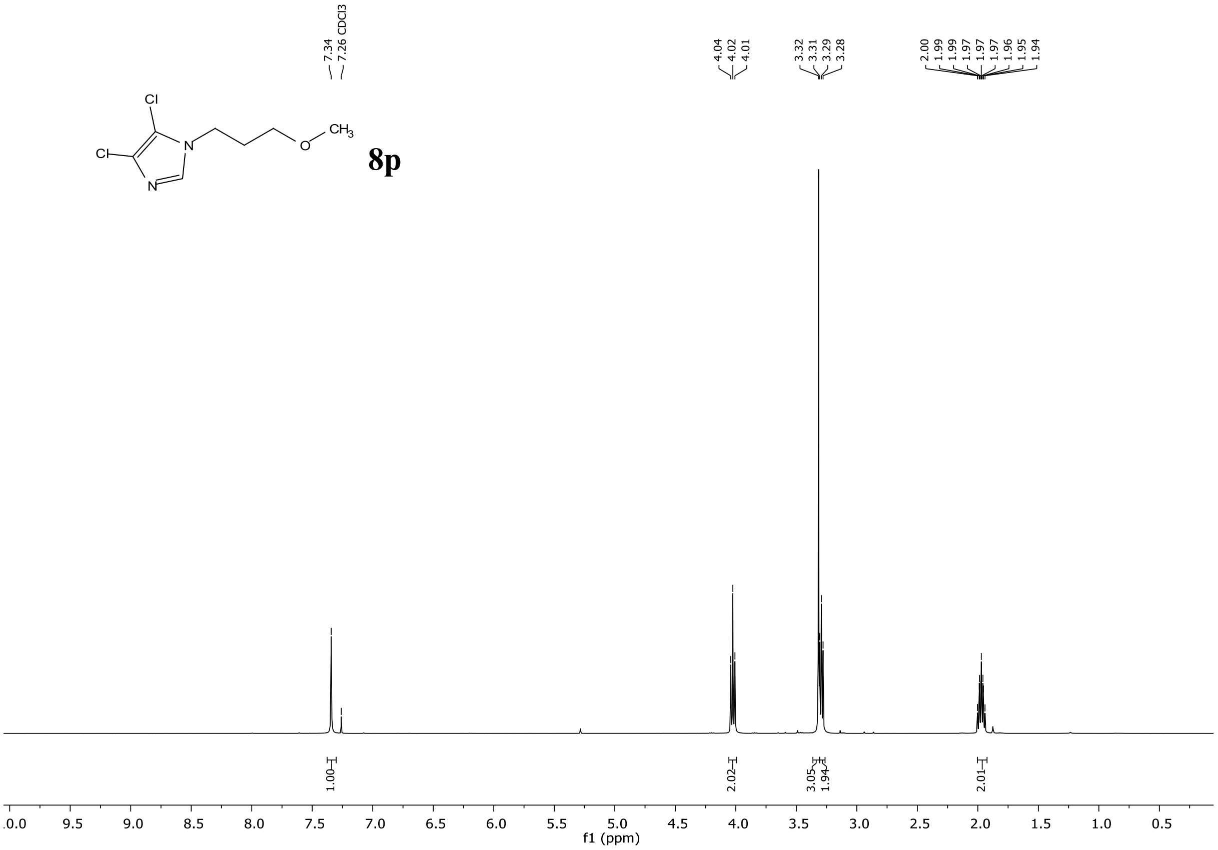

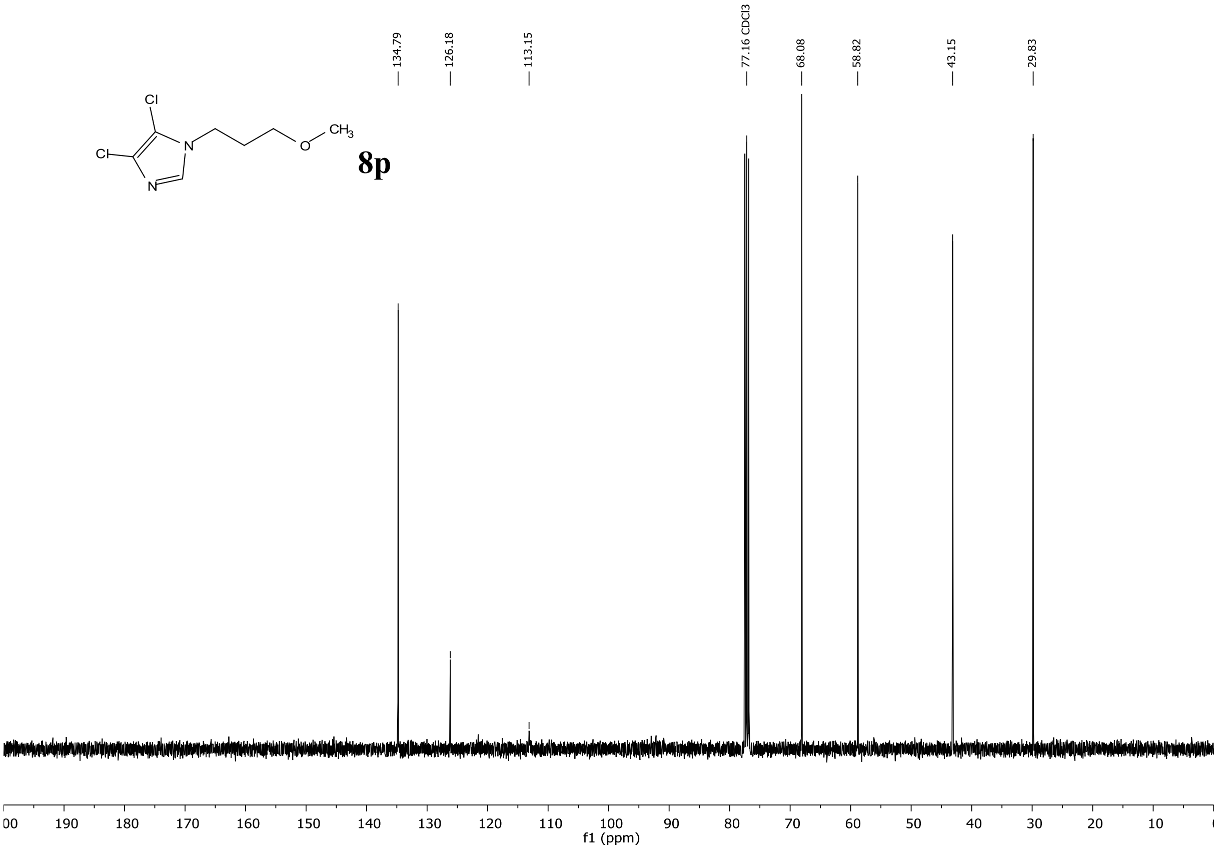

Tert-butyl 4-((4,5-dichloro-imidazol-1-yl)methyl)benzoate (8o)

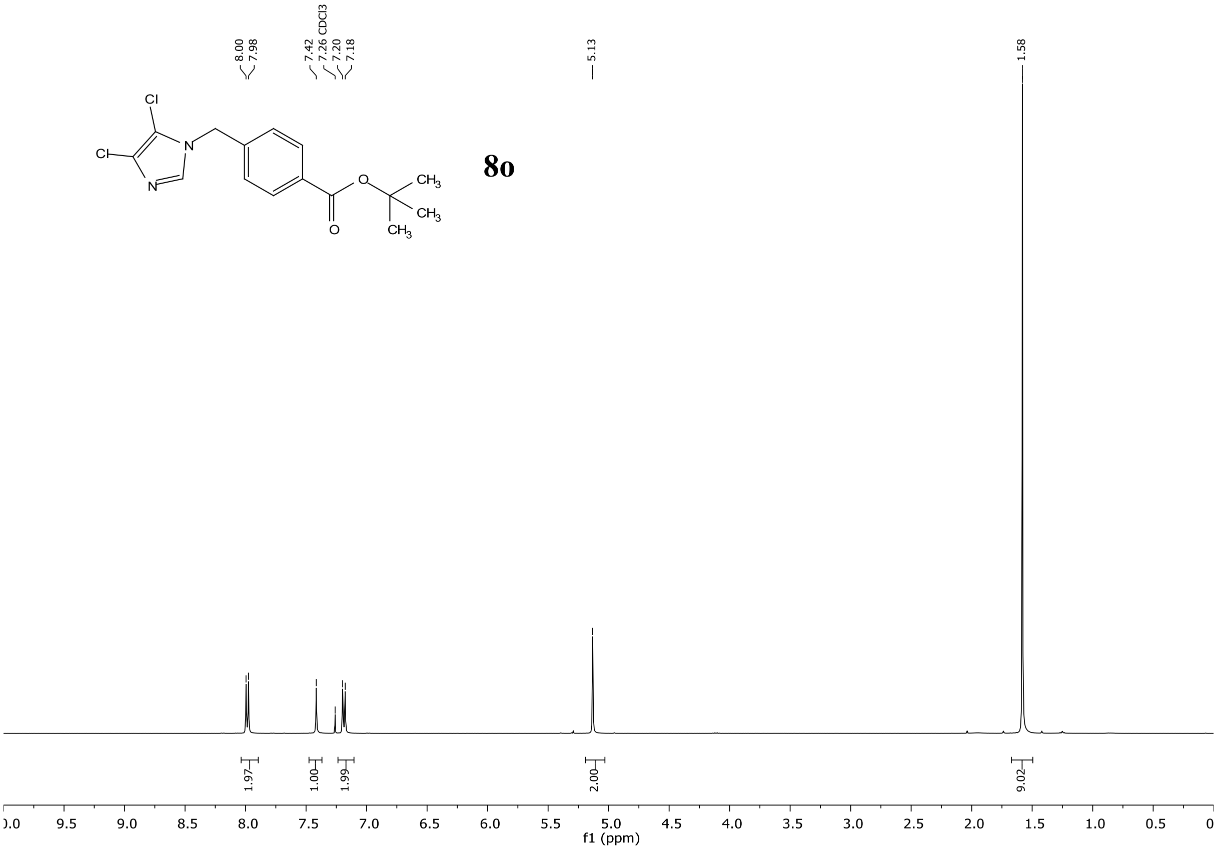

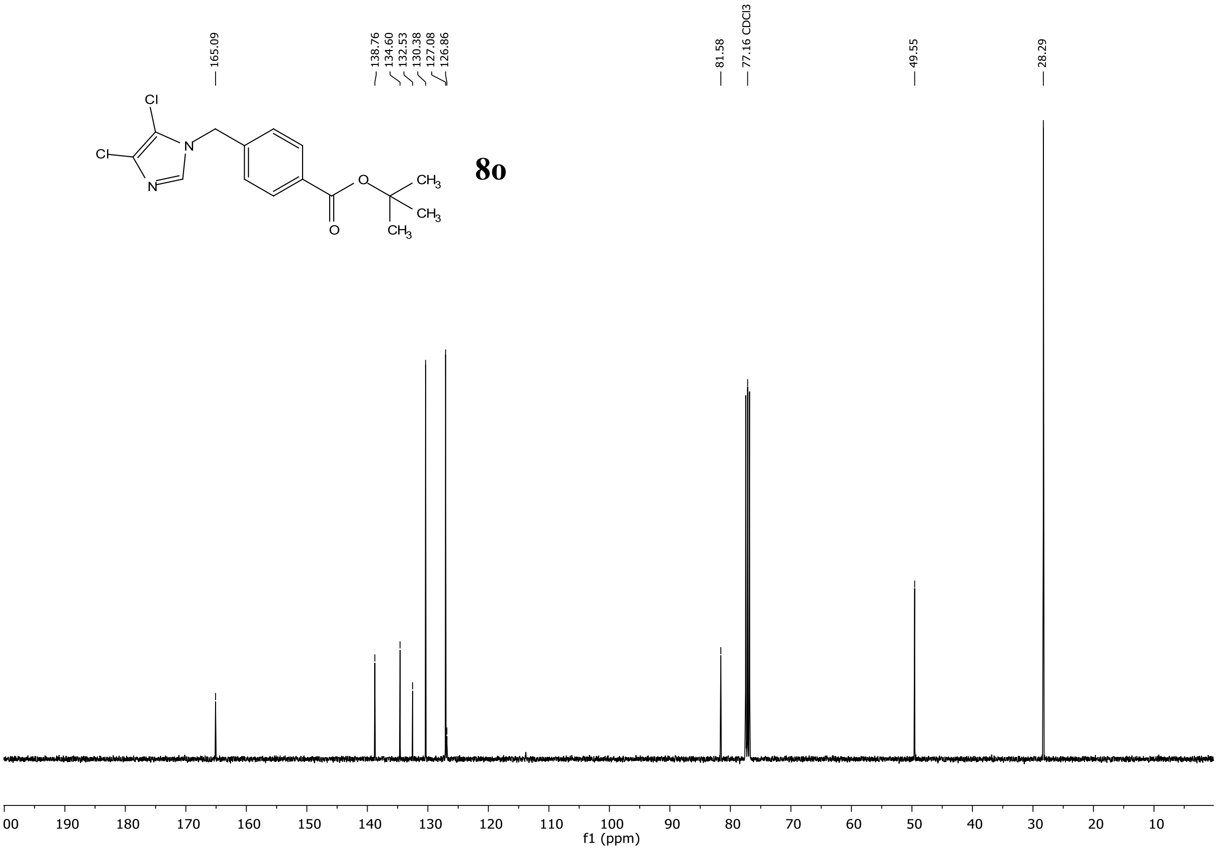

3-(4,5-Dichloro-imidazol-1-yl)dihydrofuran-2(3*H*)-one (8e)

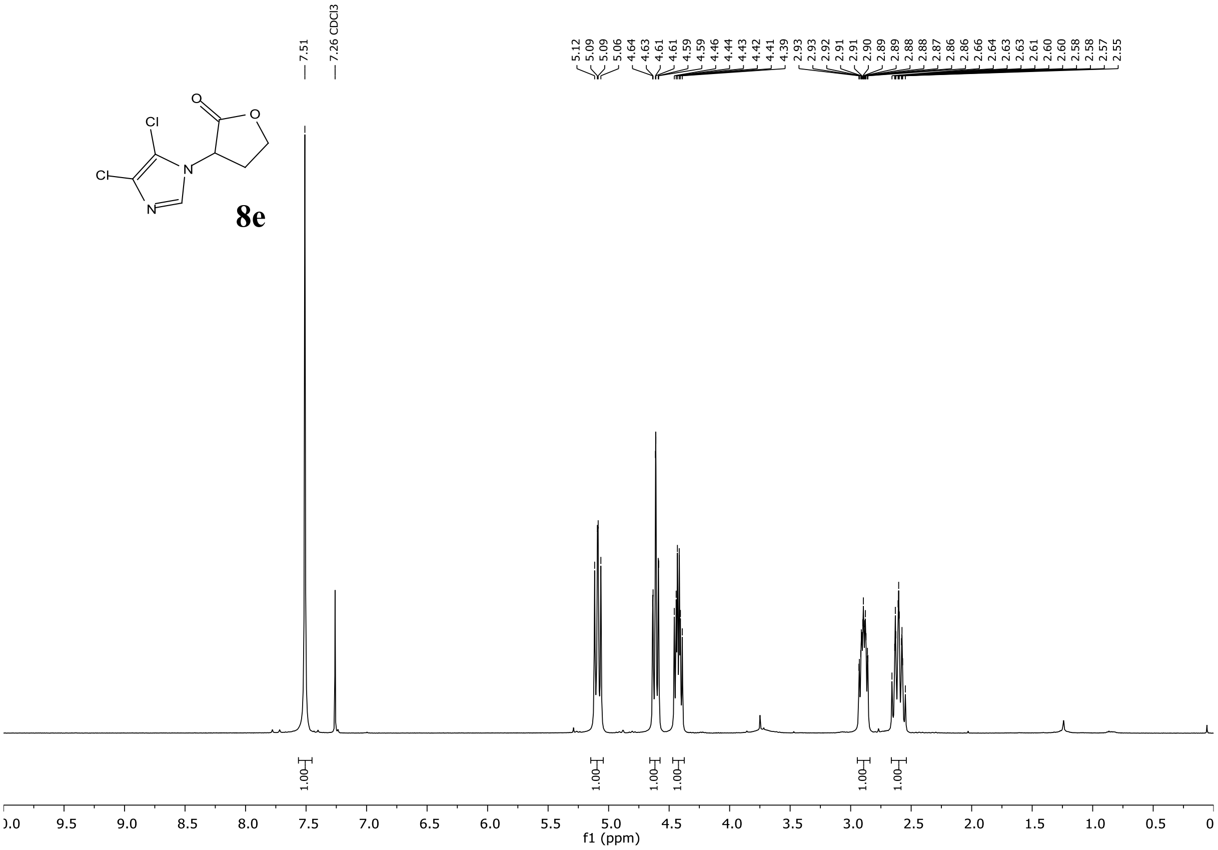

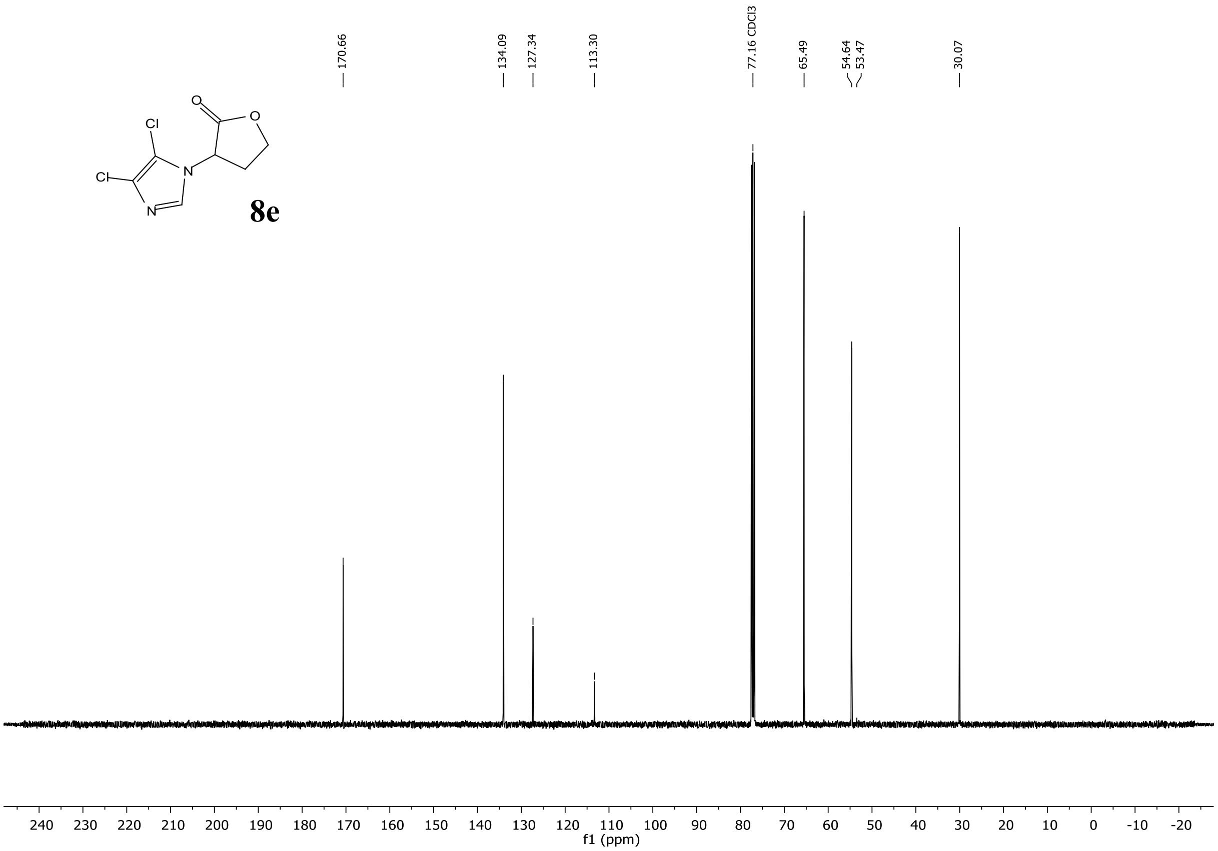

2-(4,5-Dichloro-1*H*-imidazol-1-yl)ethan-1-ol (8r)

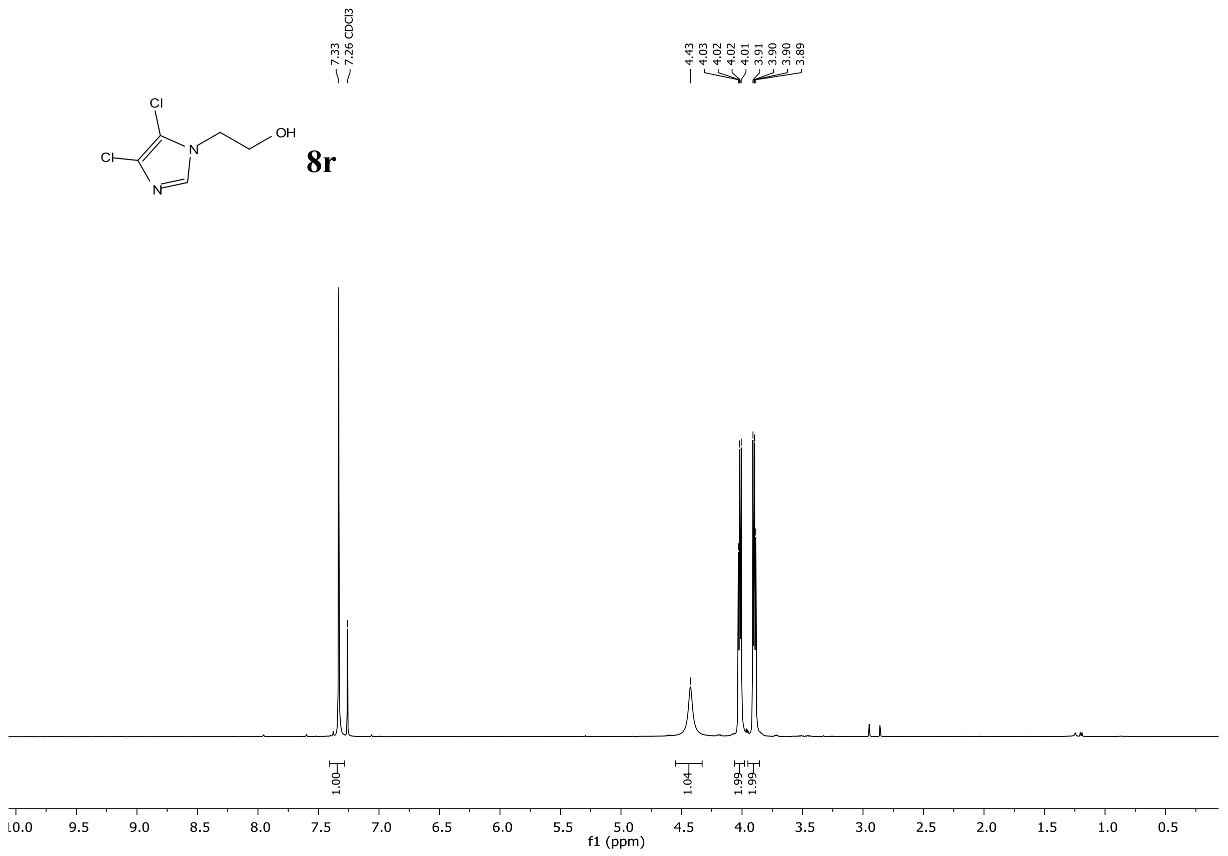

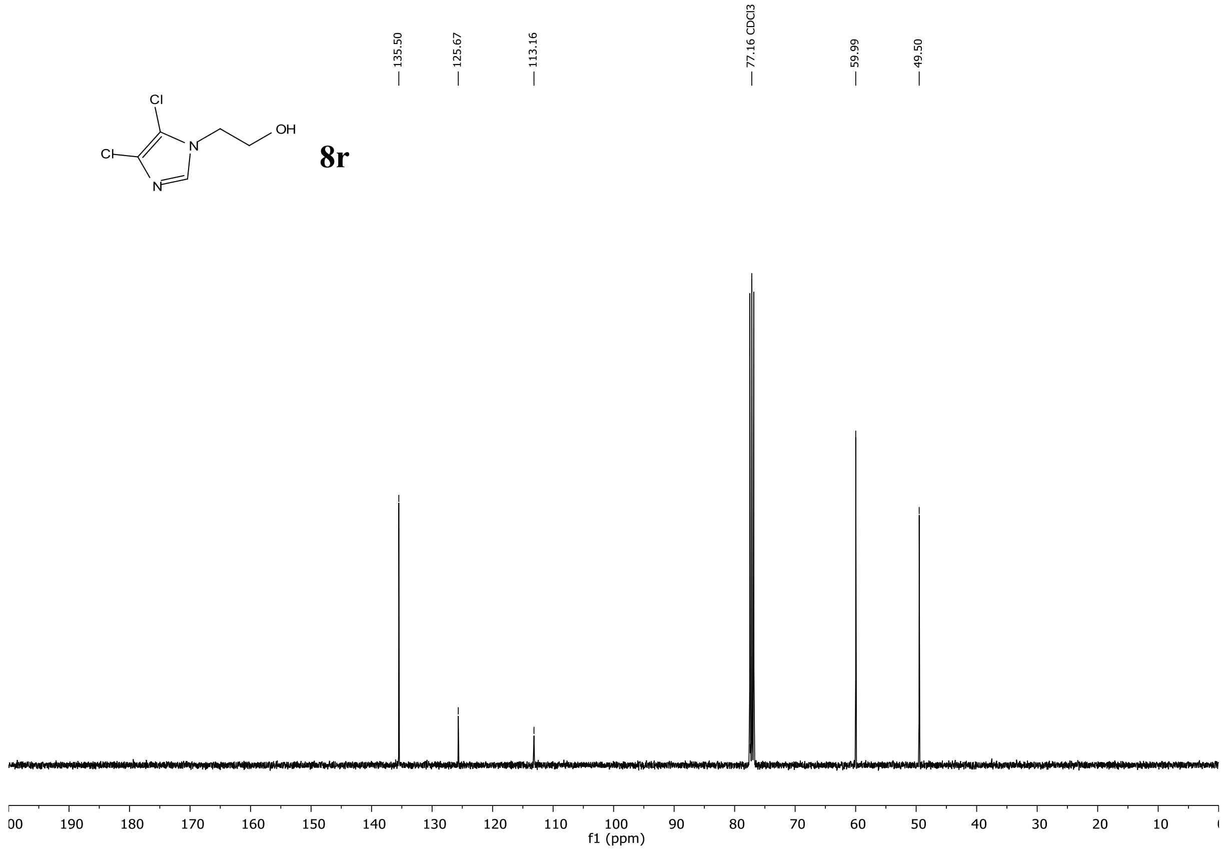

4-(4,5-Dichloro-imidazol-1-yl)butanenitrile (8q)

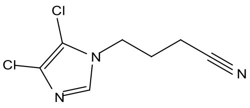

8q

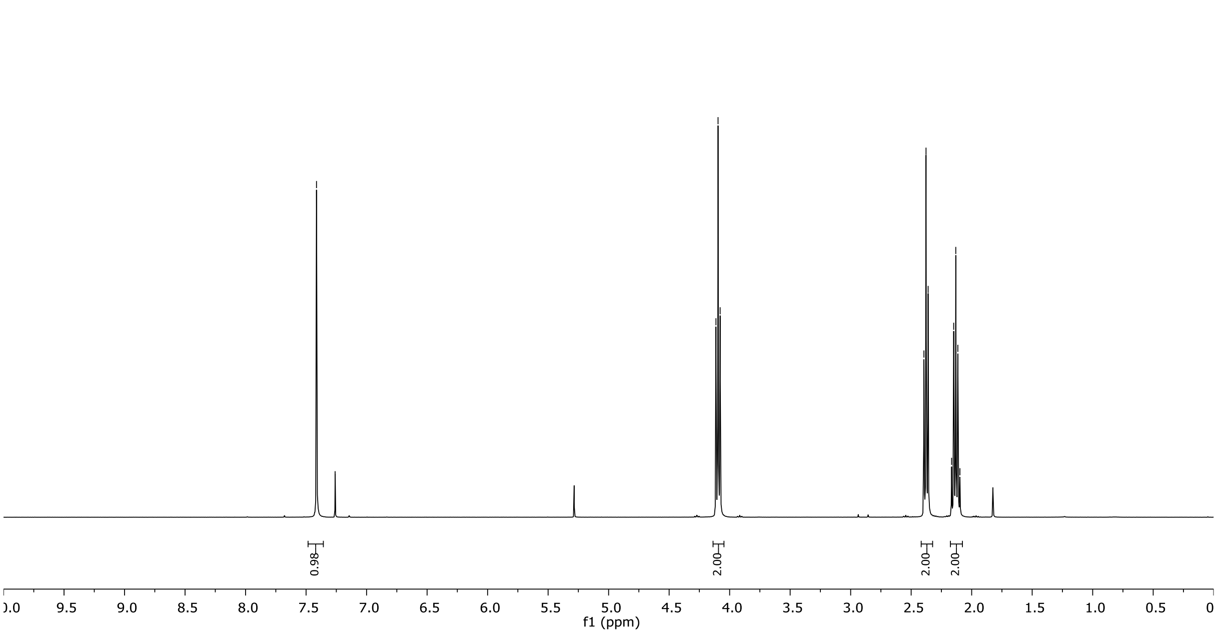

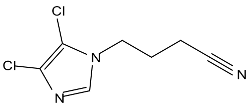

8q

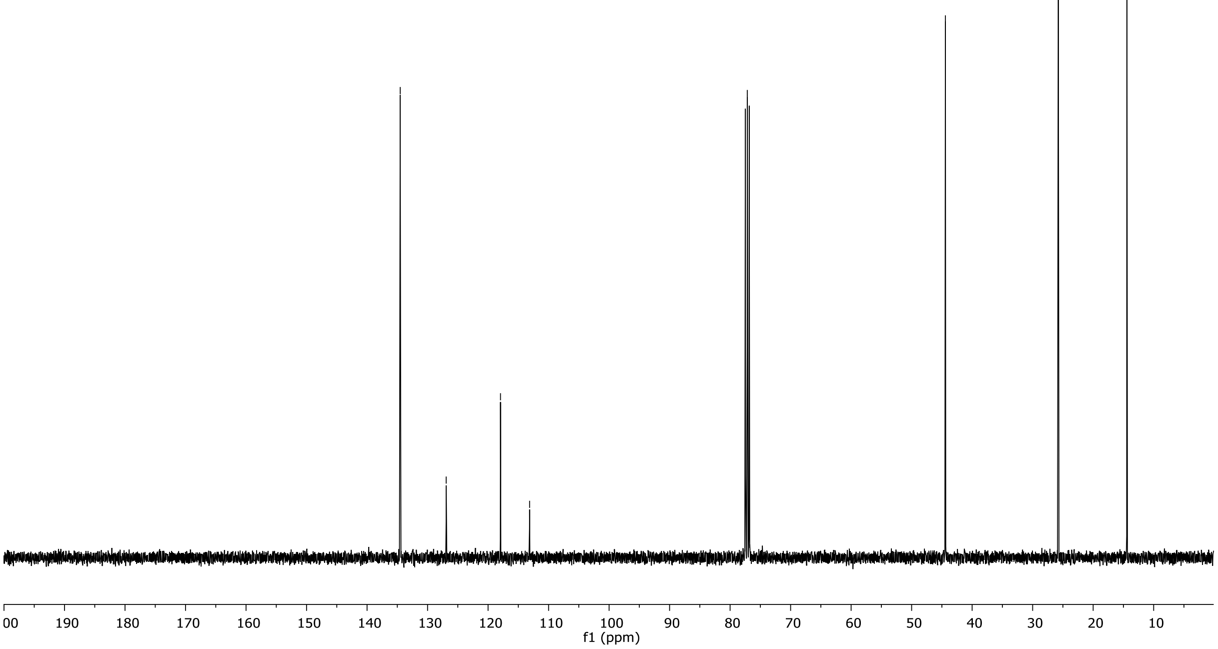

2-((4,5-Dichloro-imidazol-1-yl)methyl)pyridine (8s)

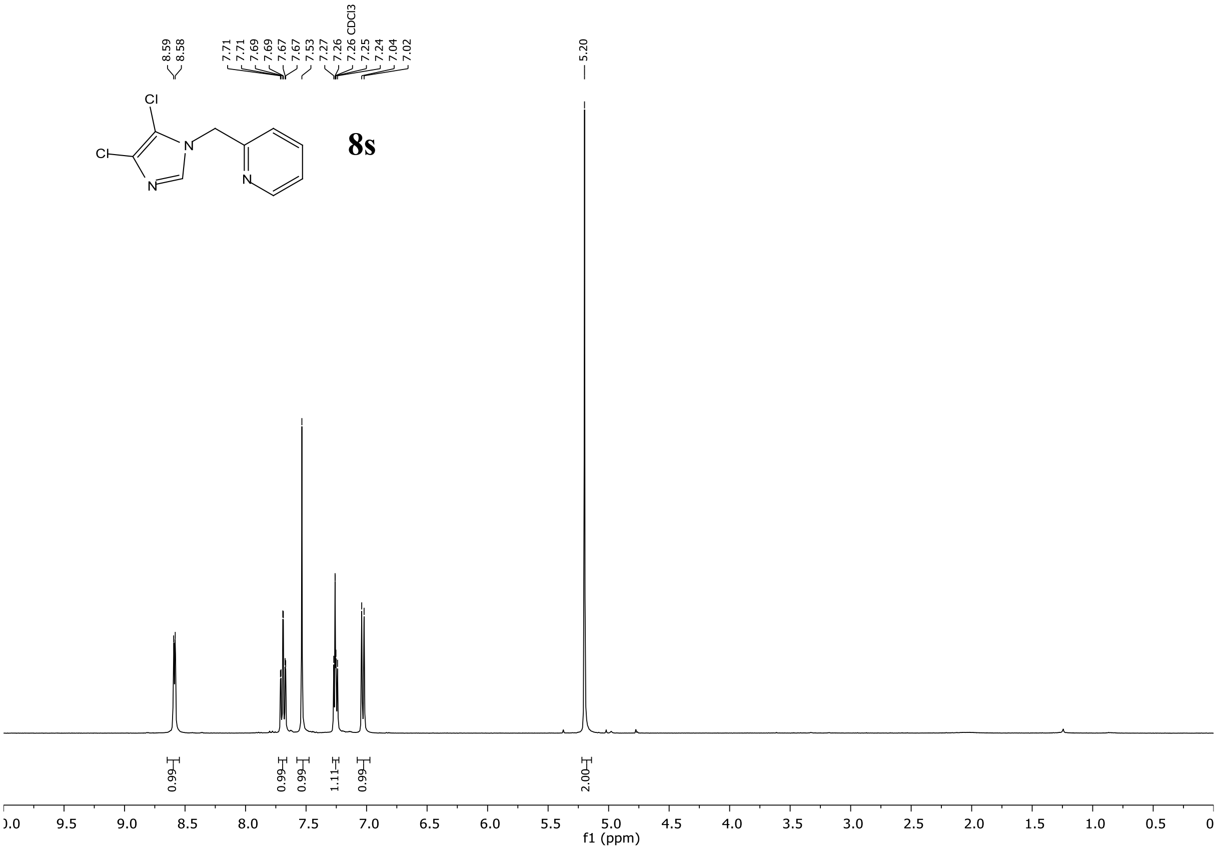

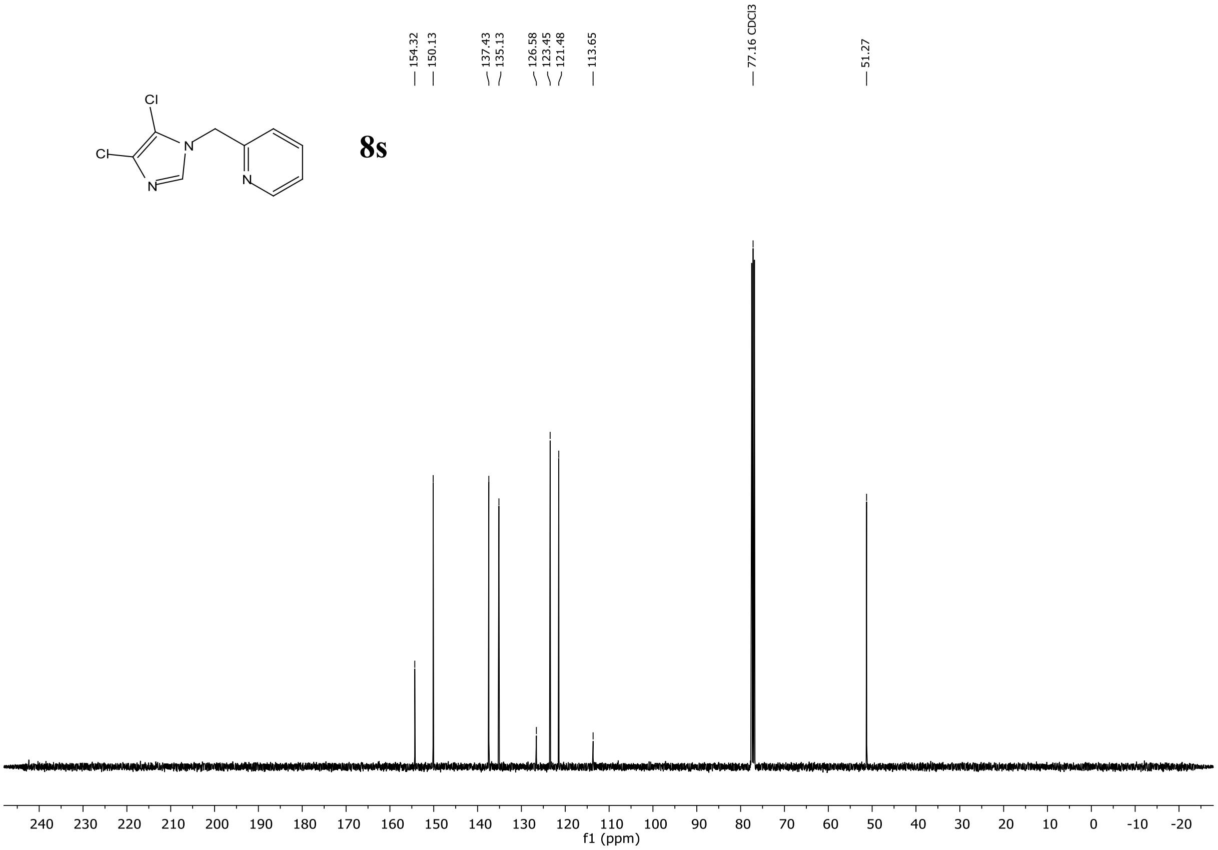

Diethyl (3-(4,5-dichloro-imidazol-1-yl)propyl)phosphonate (8t)

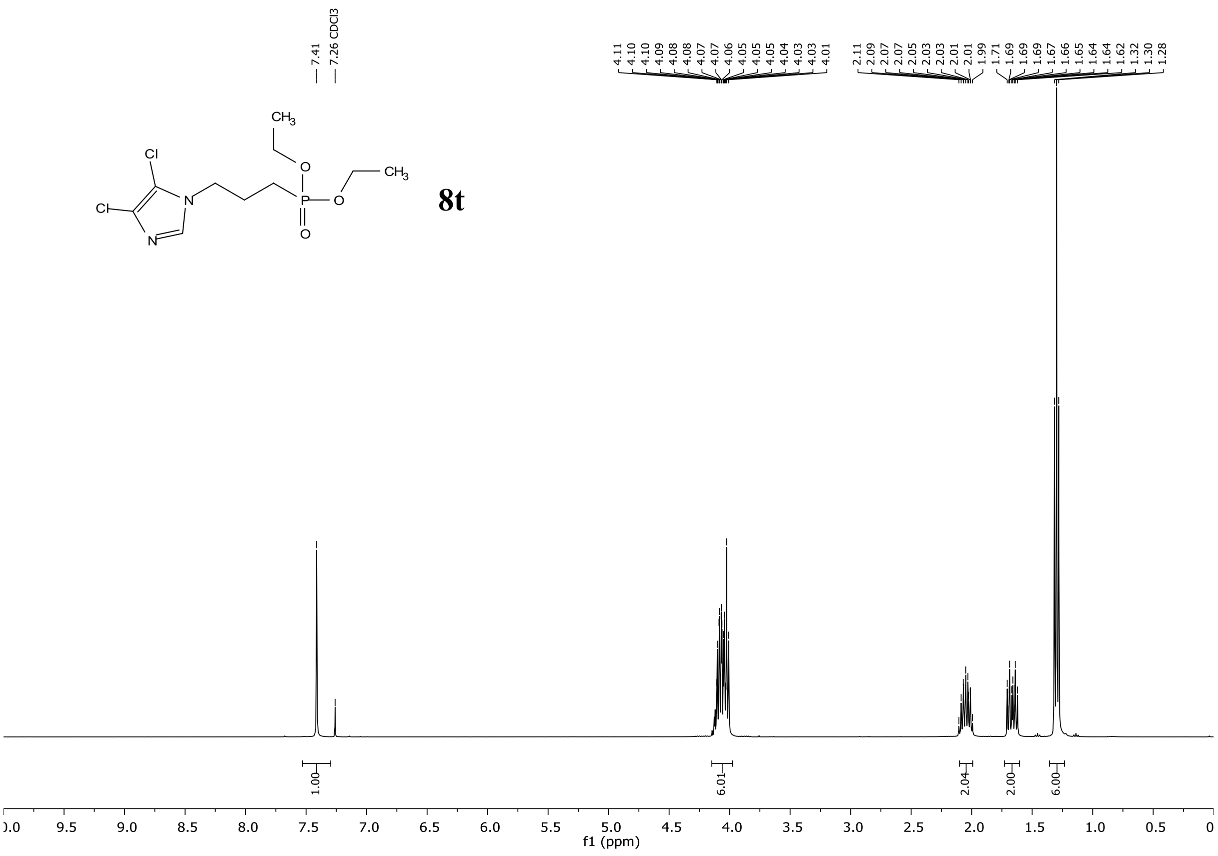

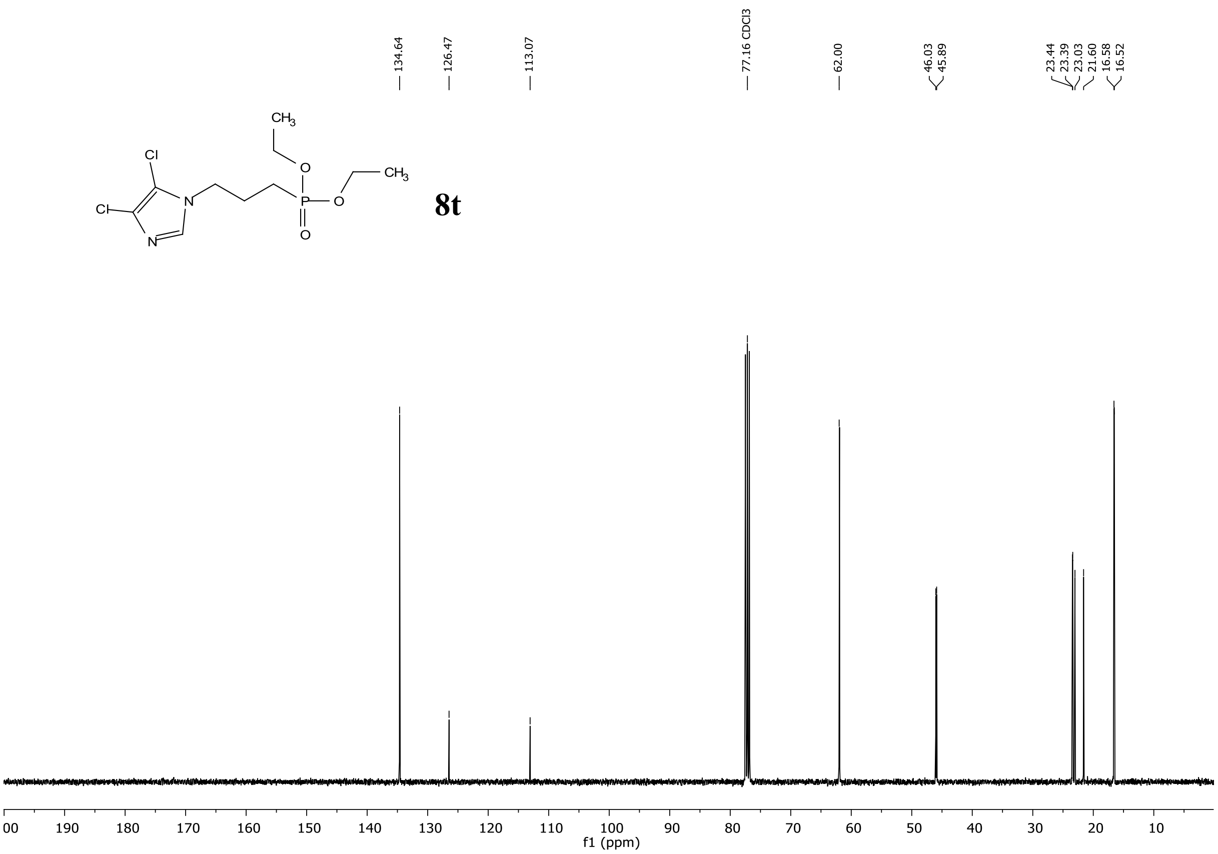

2,3,4-Trichloro-1-methyl-5-phenyl-1H-pyrrole (8u)

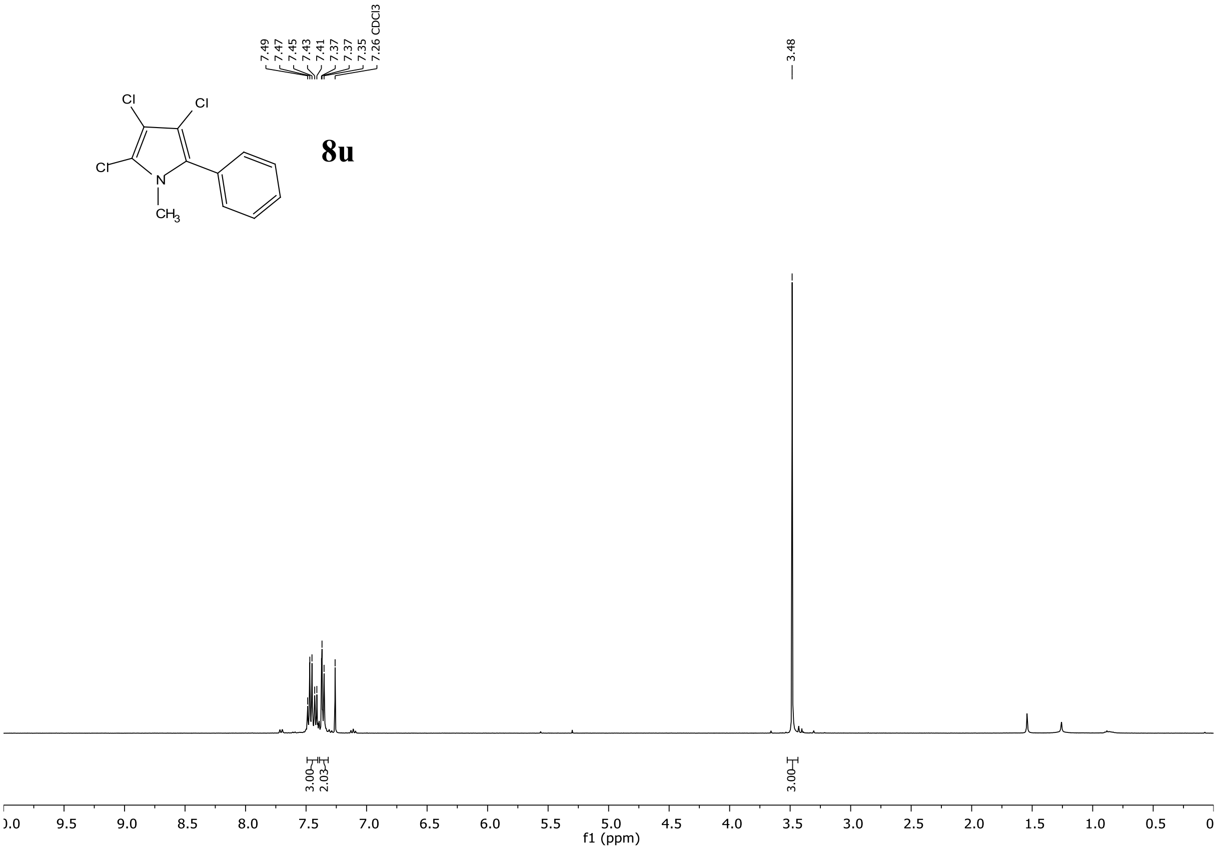

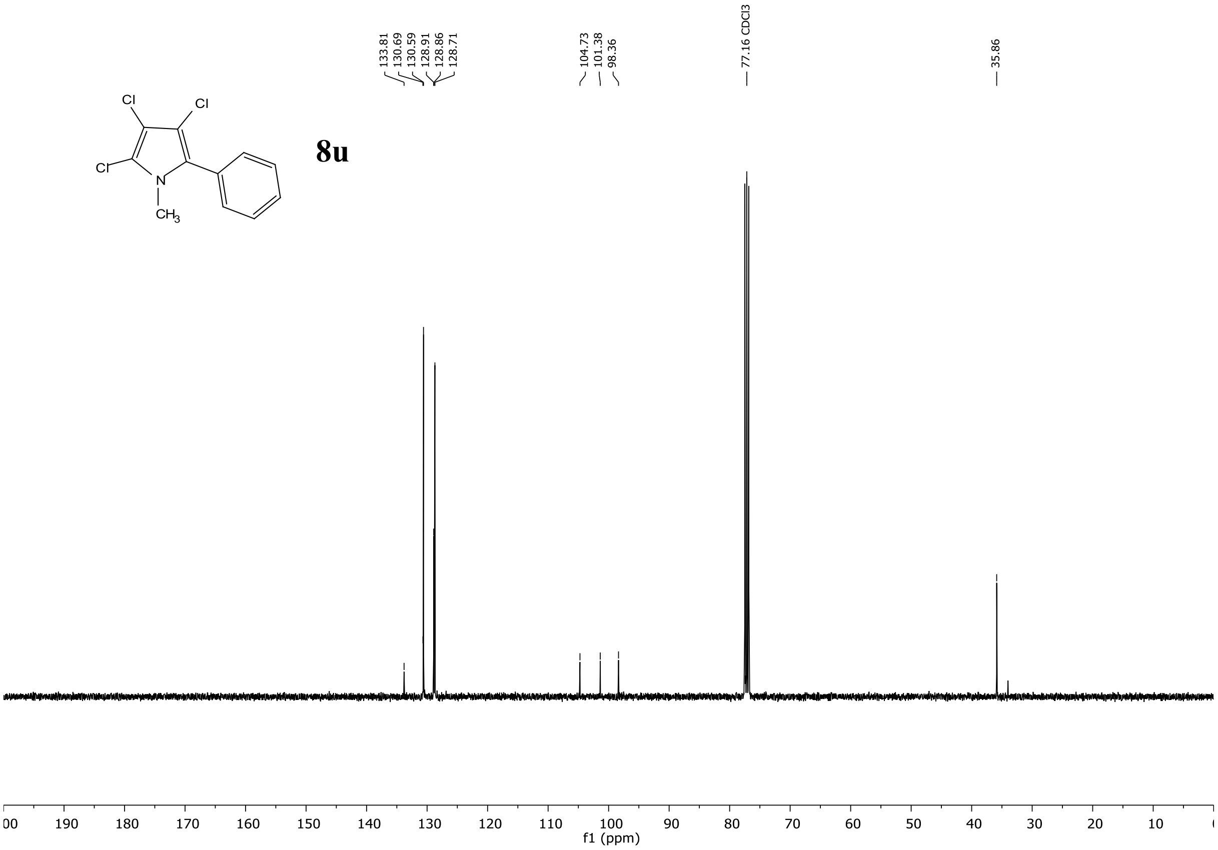

2,3,4-Tribromo-1-methyl-5-phenyl-1*H*-pyrrole (8v)

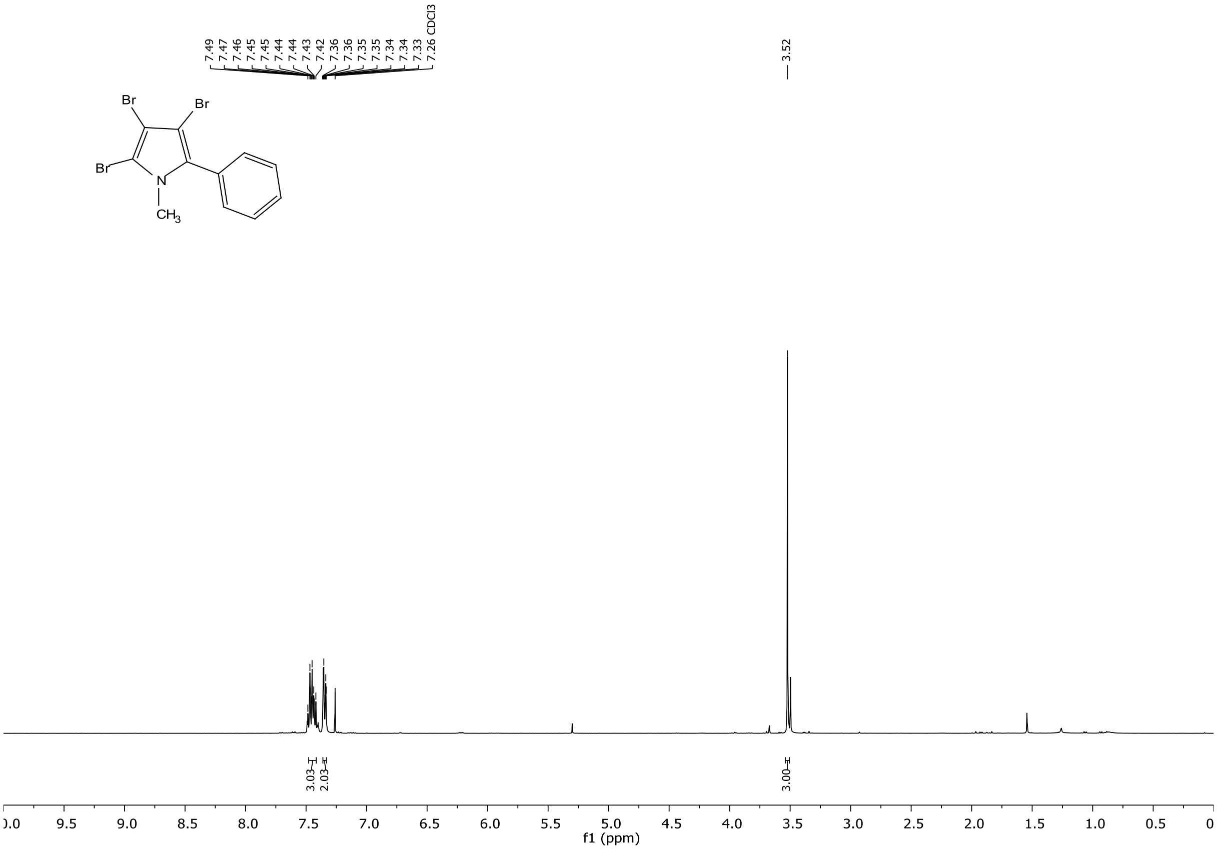

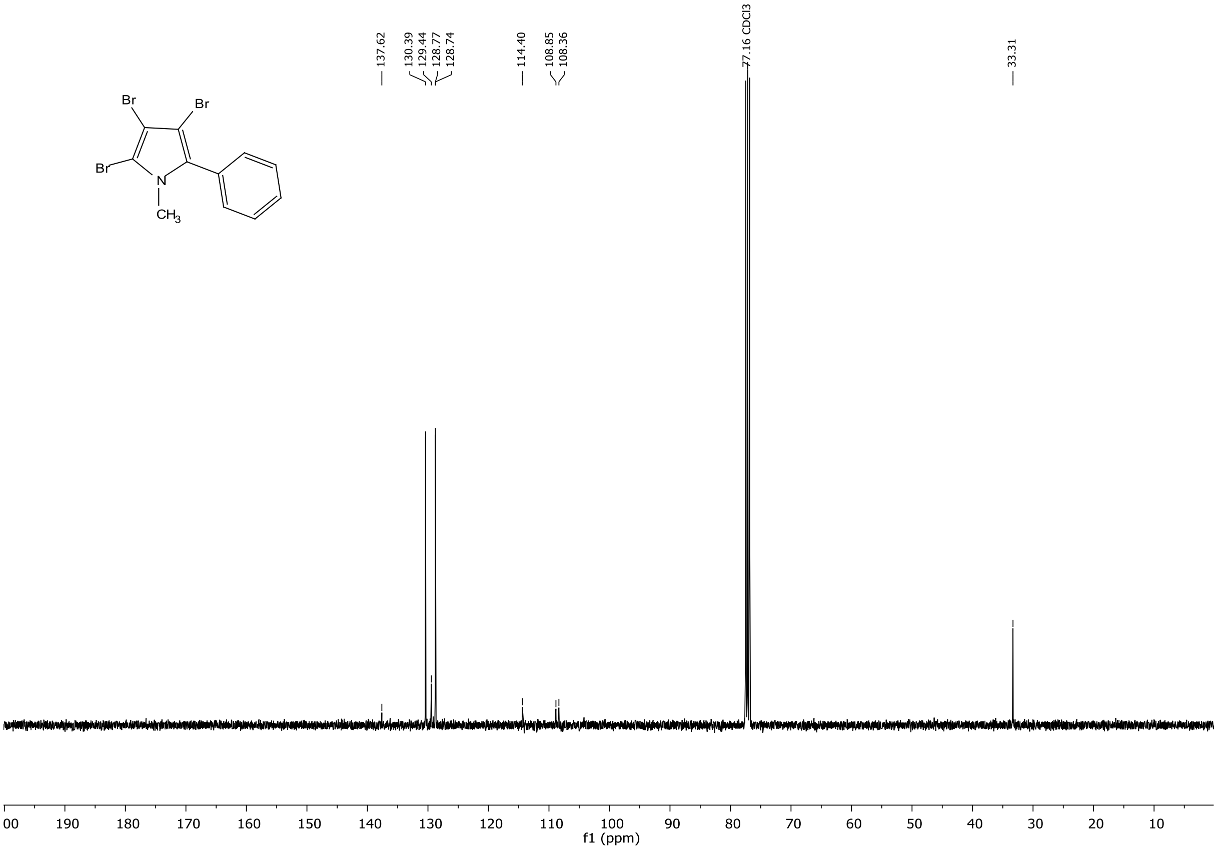

1-Benzyl-4-chloro-5-((trifluoromethyl)thio)-1*H*-imidazole (9a)

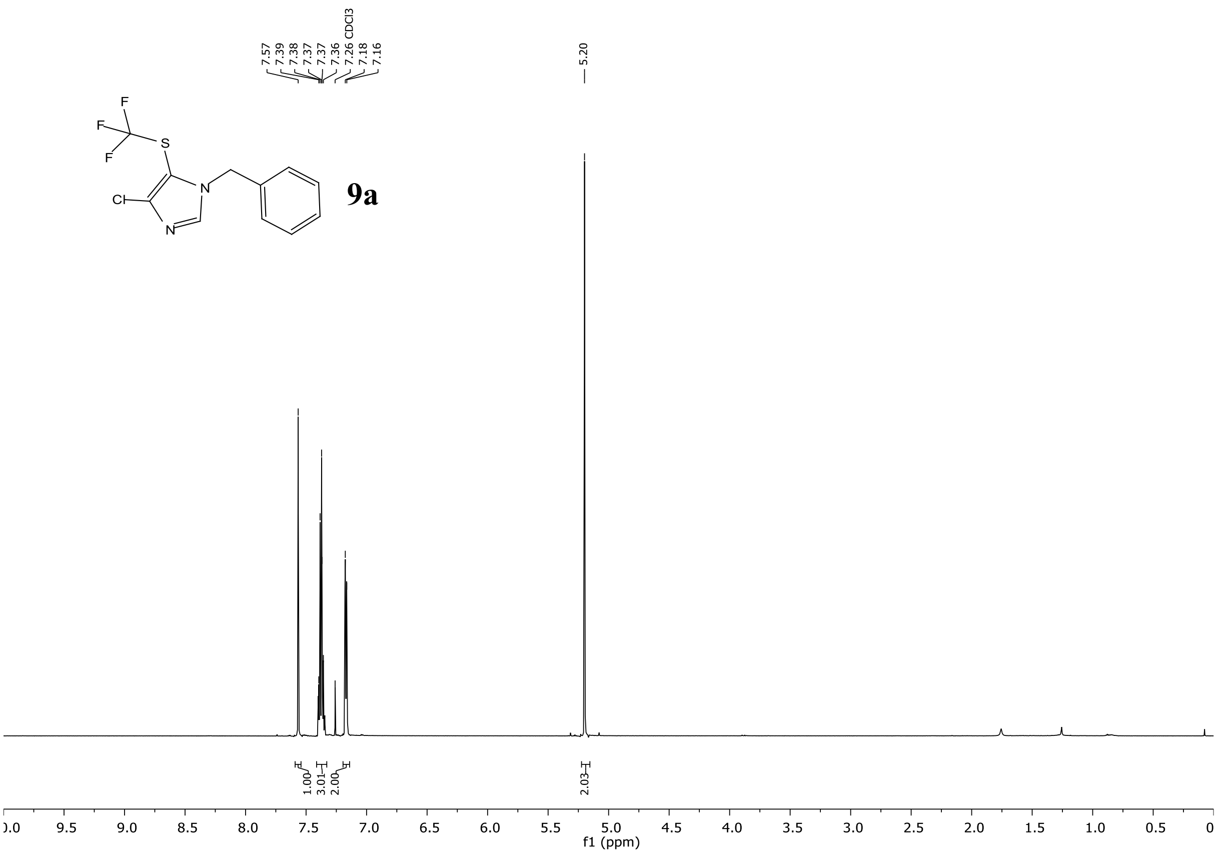

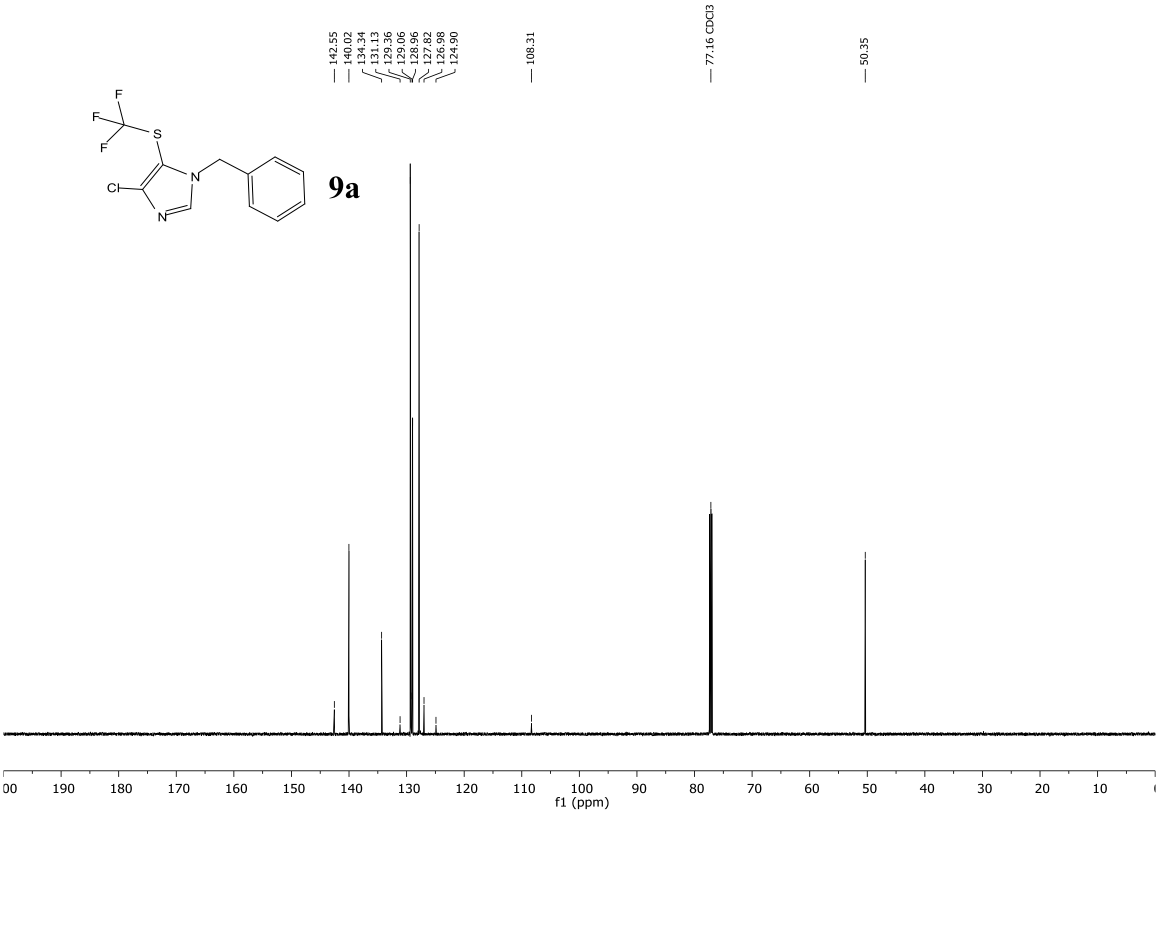

4-Chloro-1-methyl-5-((trifluoromethyl)thio)-1*H*-imidazole (9j)

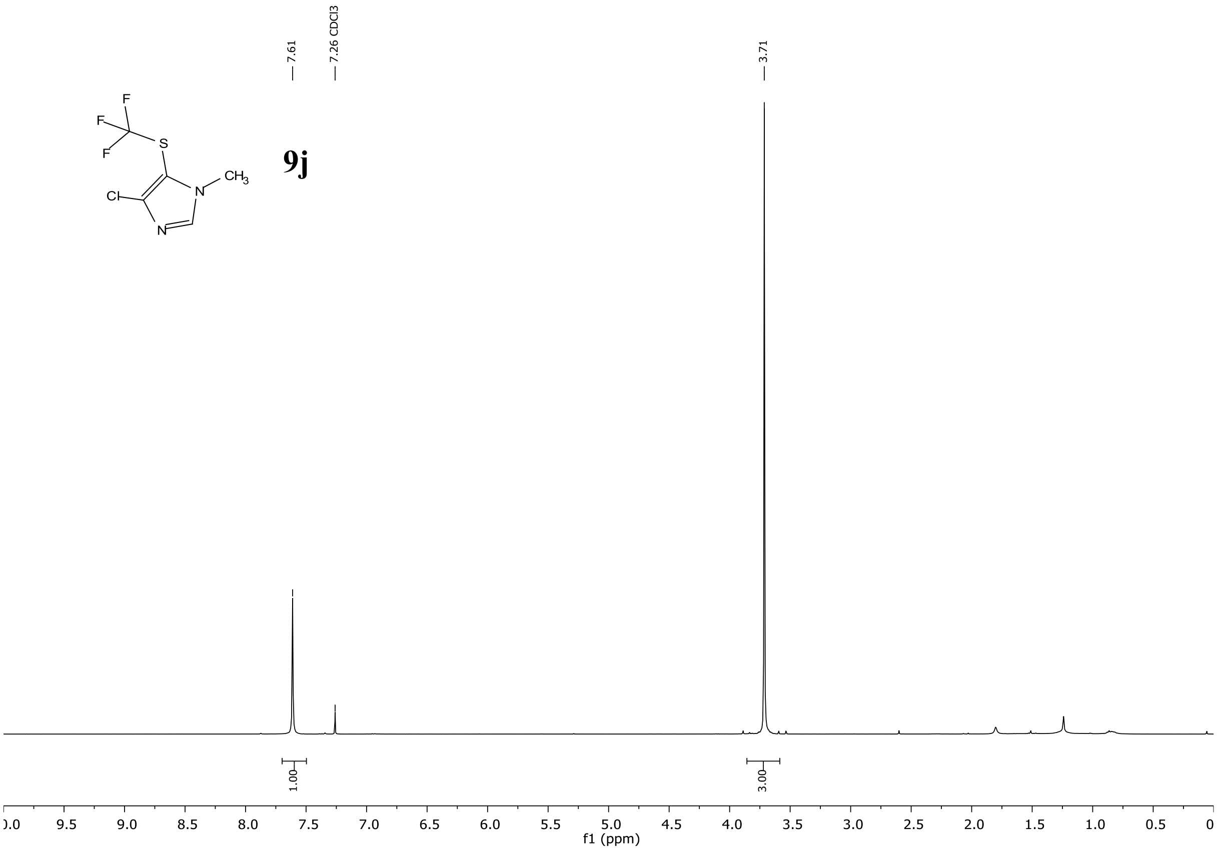

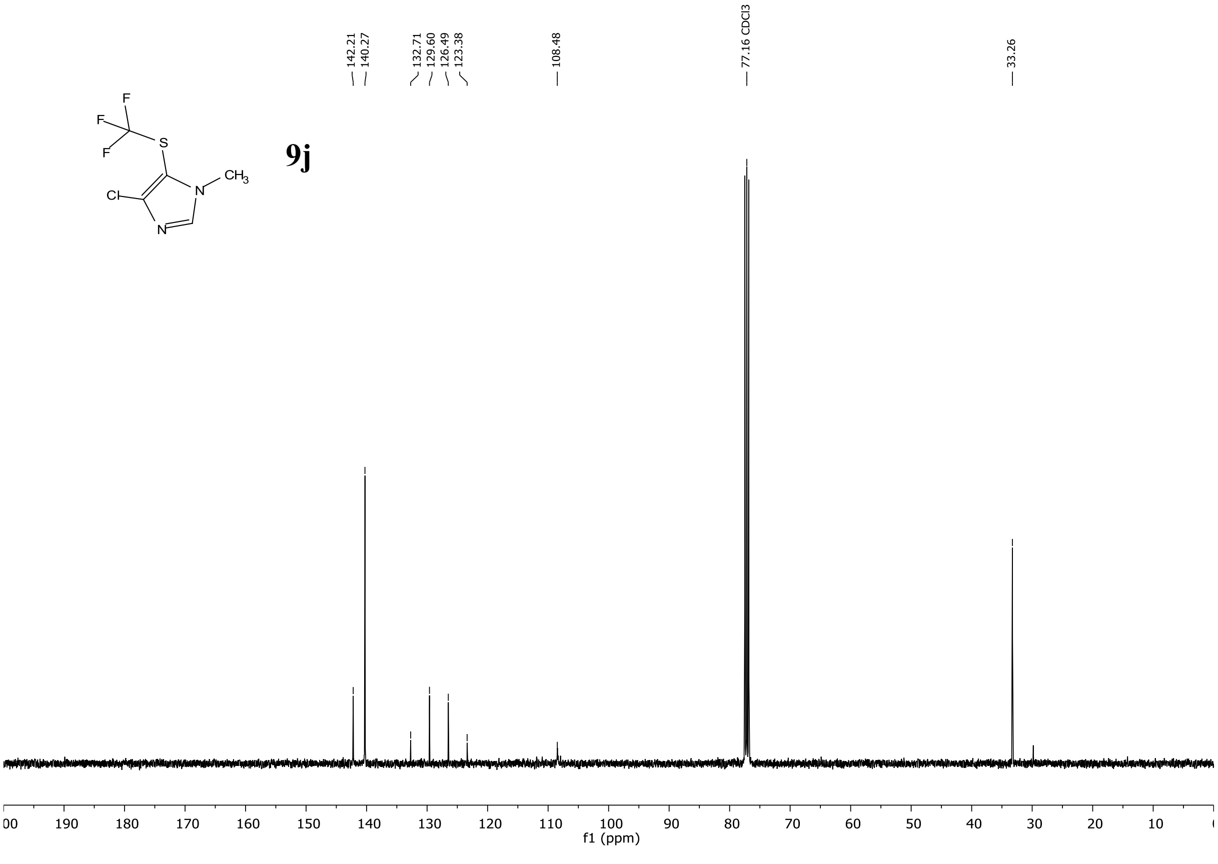

4-Bromo-1-methyl-5-((trifluoromethyl)thio)-1*H*-imidazole (9b)

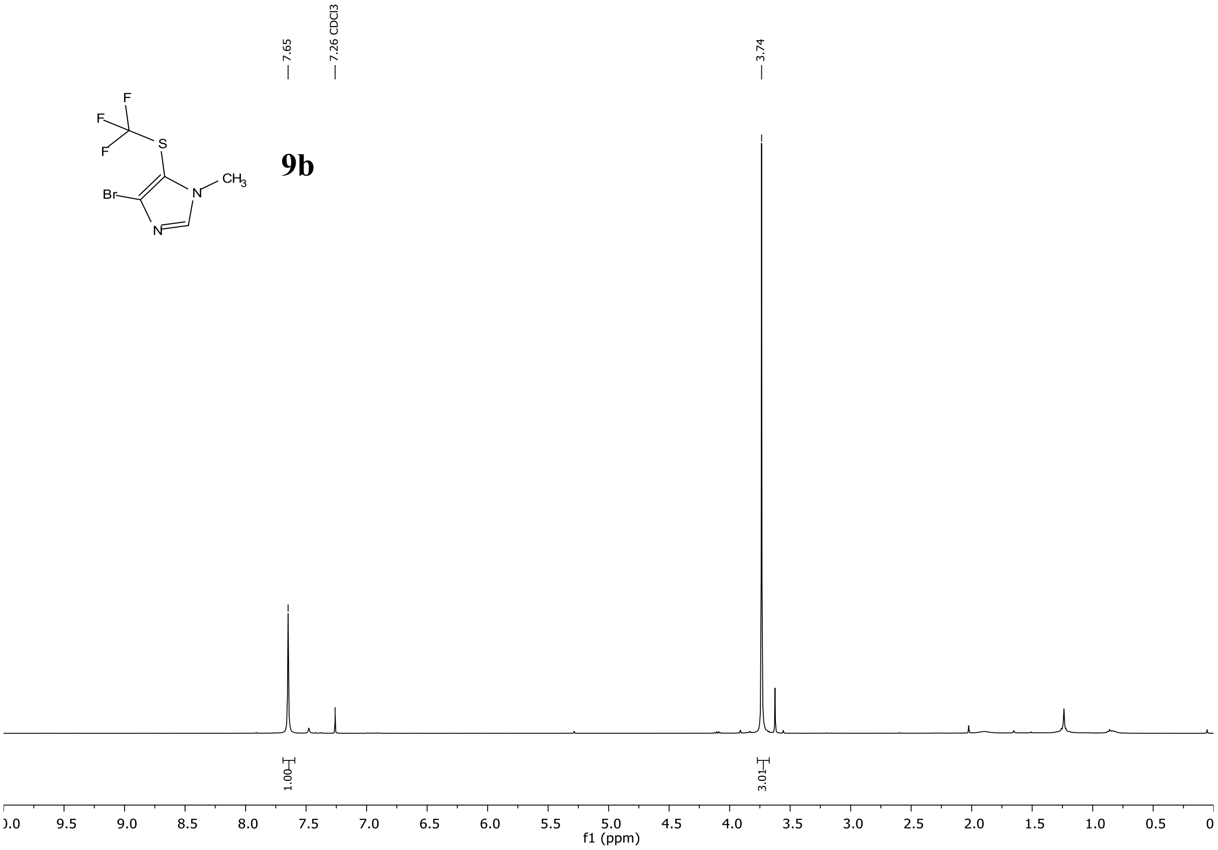

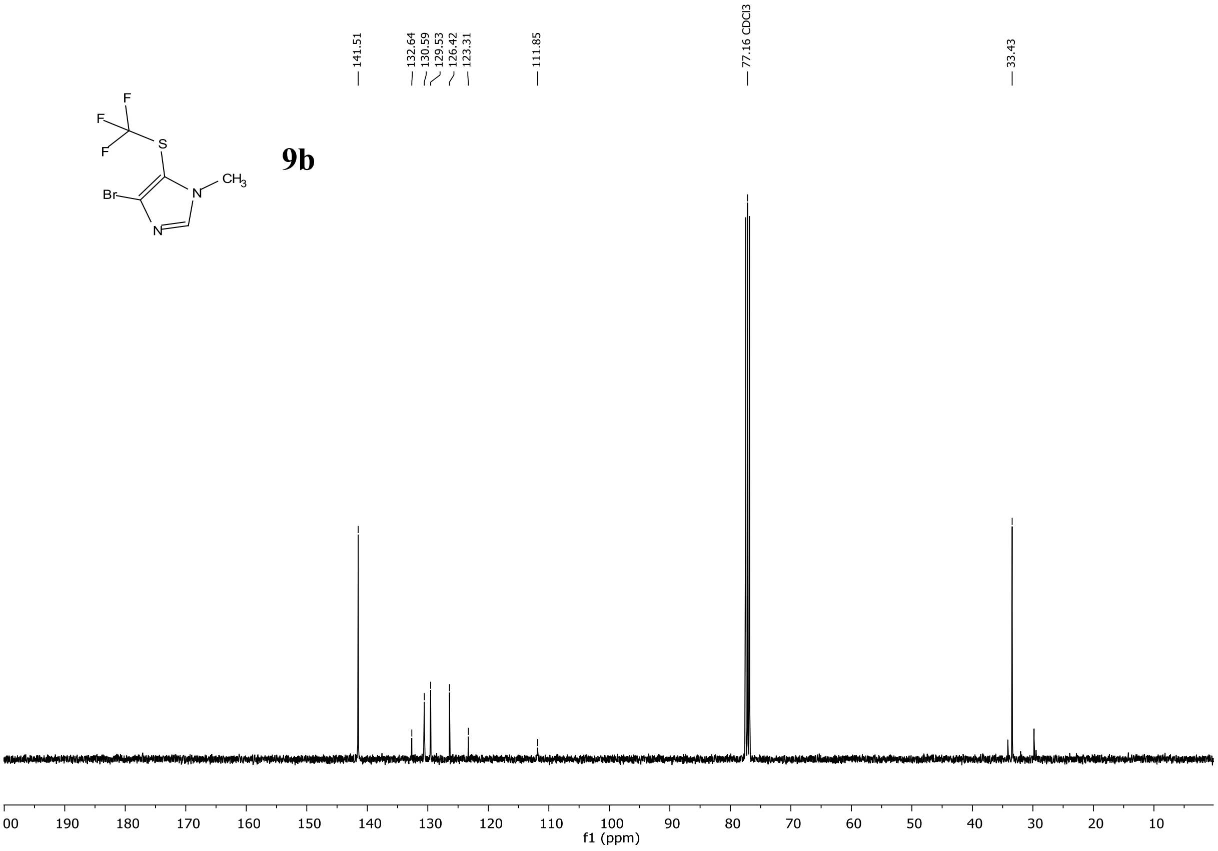

2,4-Dibromo-1-methyl-5-((trifluoromethyl)thio)-1*H*-imidazole (9c)

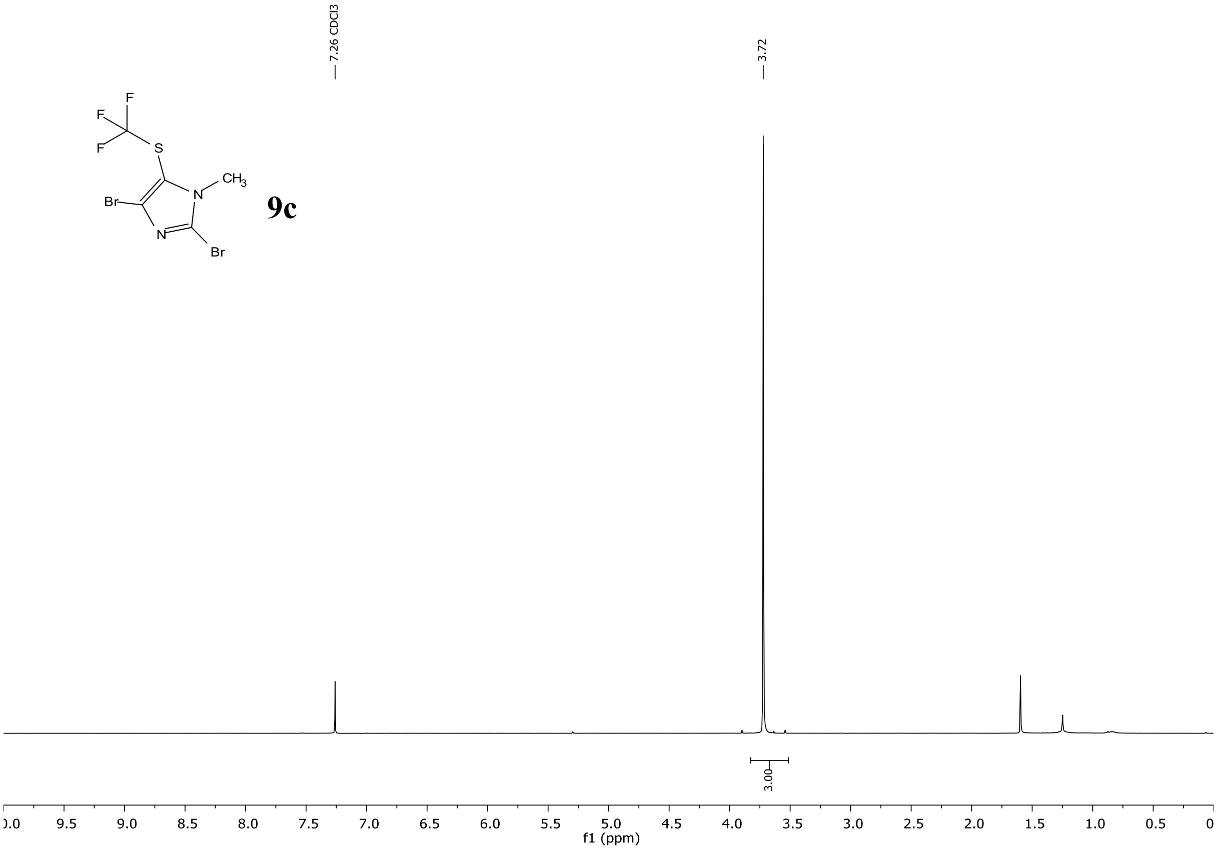

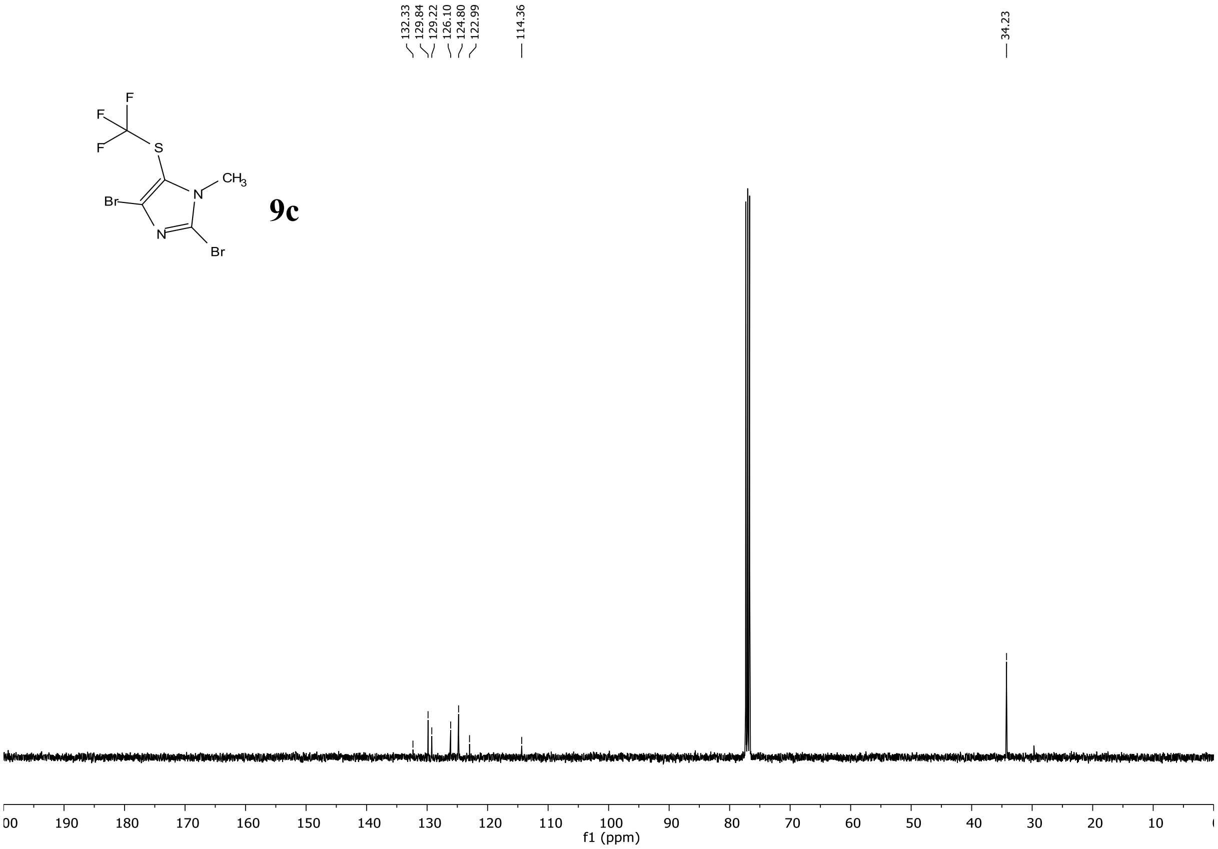

1-Benzyl-2-bromo-4-chloro-5-((trifluoromethyl)thio)-1*H*-imidazole (9i)

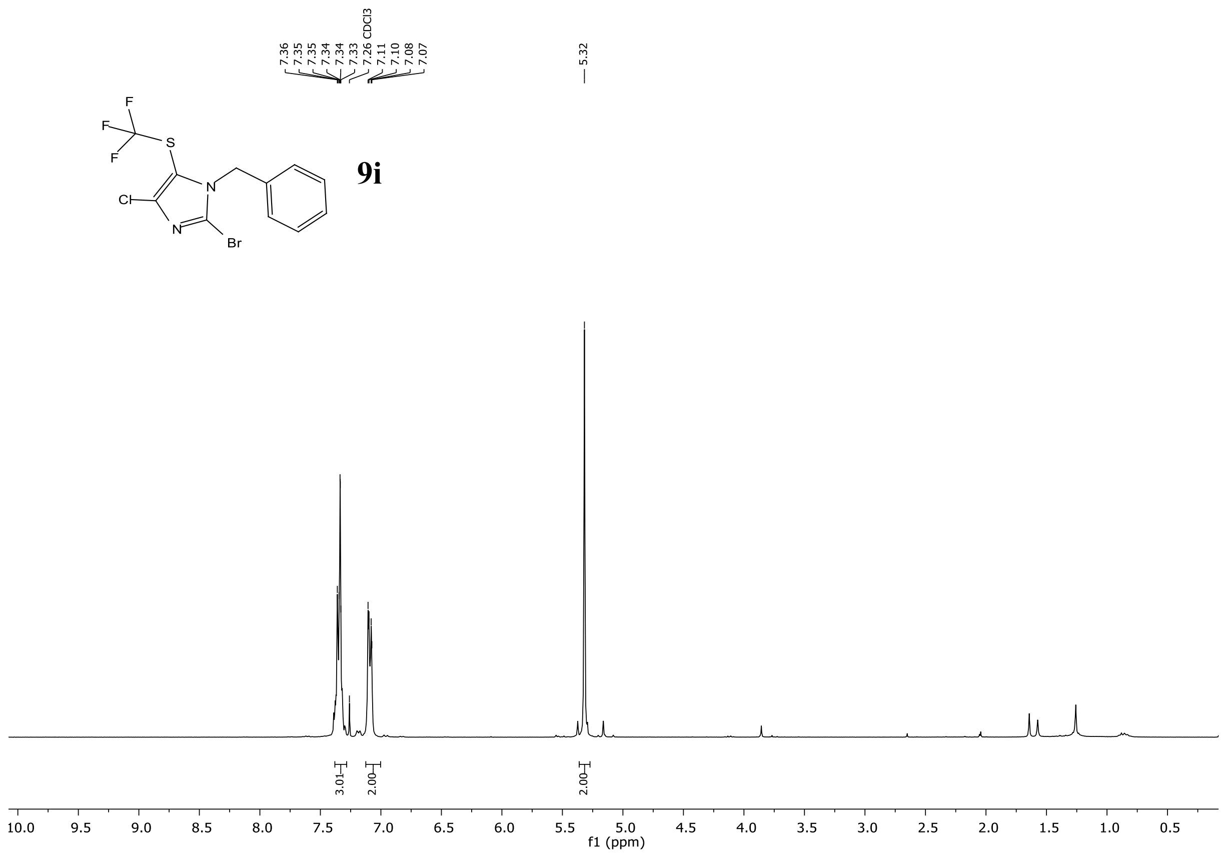

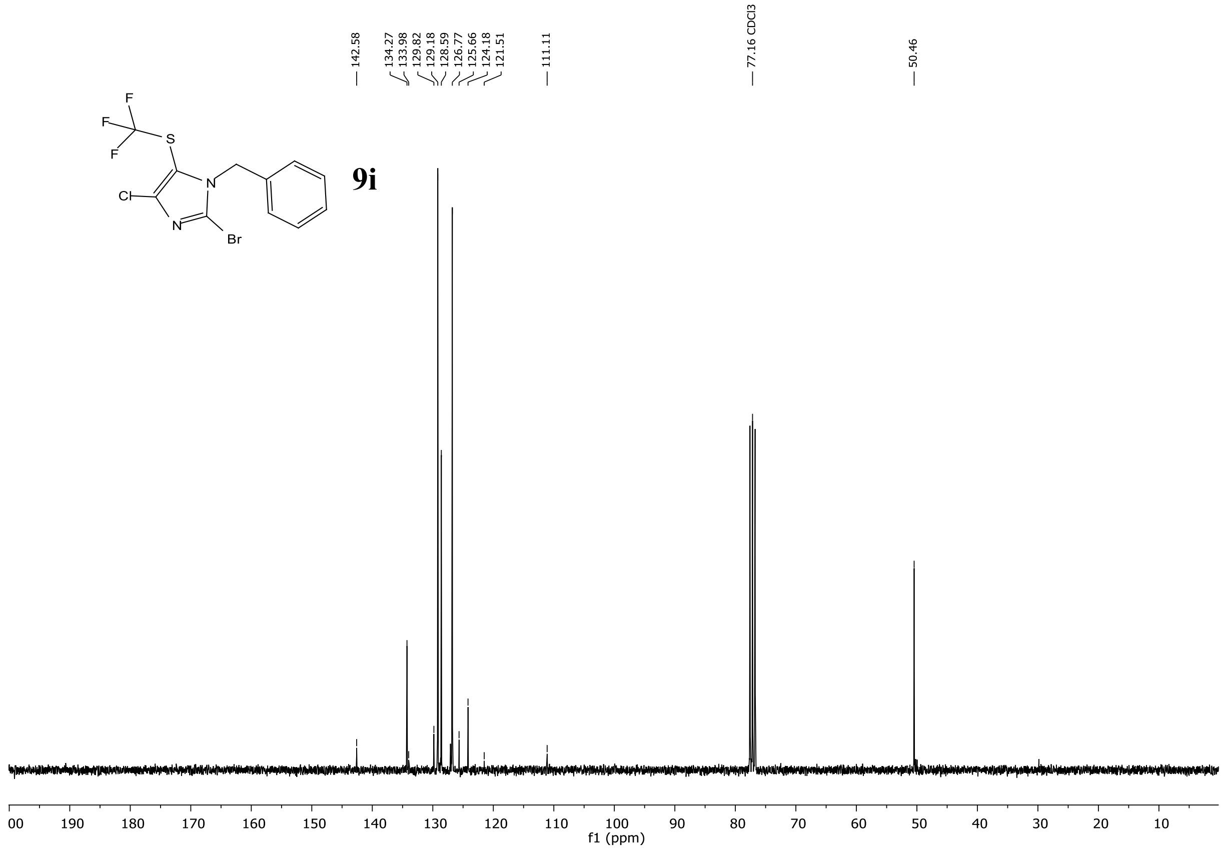

4-Chloro-1-(4-chlorobenzyl)-5-((trifluoromethyl)thio)-1*H*-imidazole (9k)

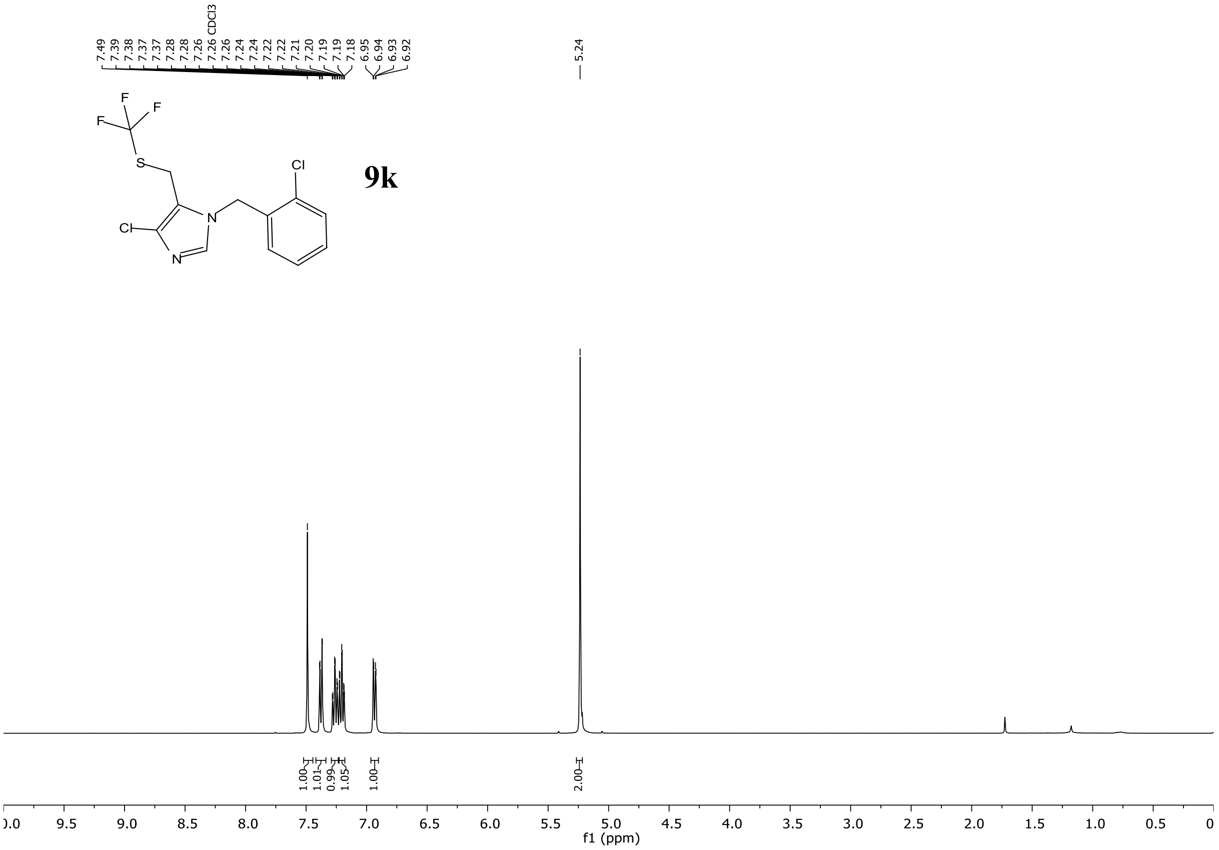

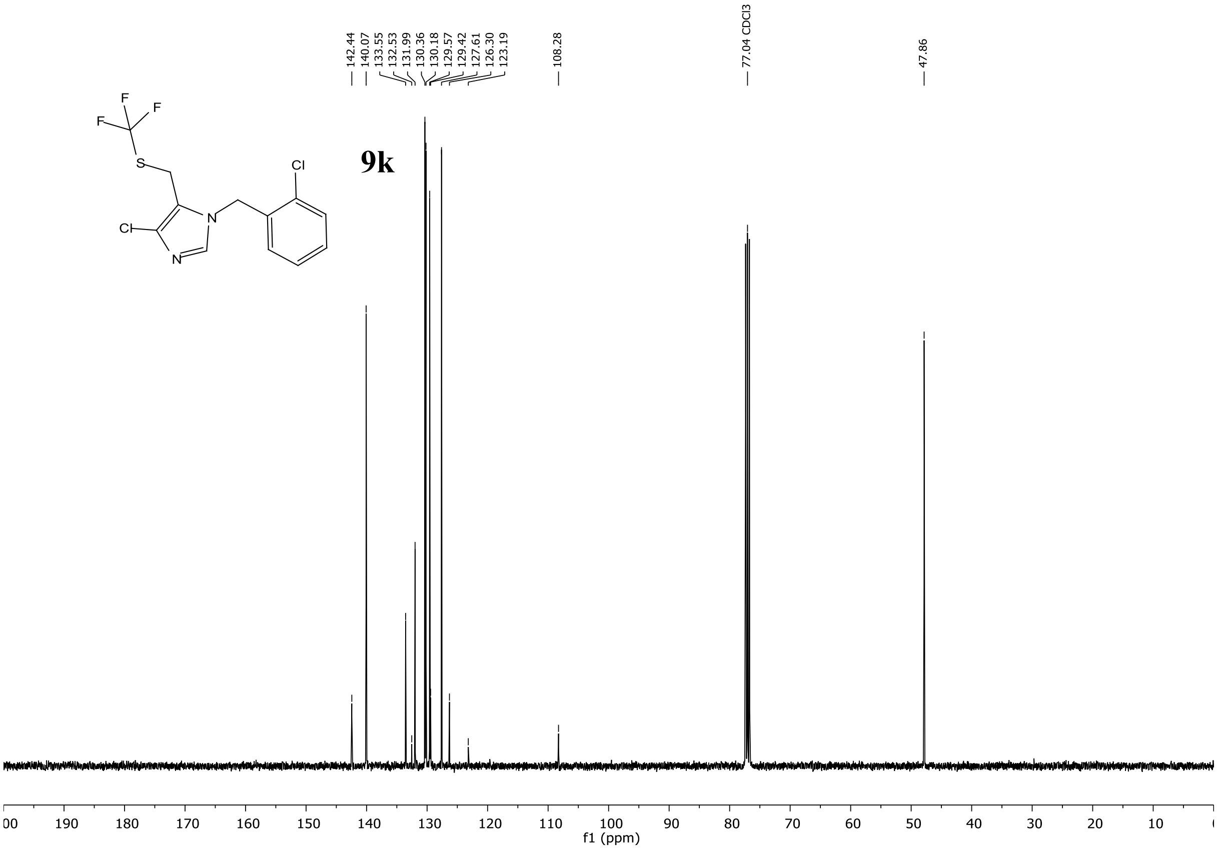

1-(4-Bromobenzyl)-4-chloro-5-((trifluoromethyl)thio)-1*H*-imidazole (9l)

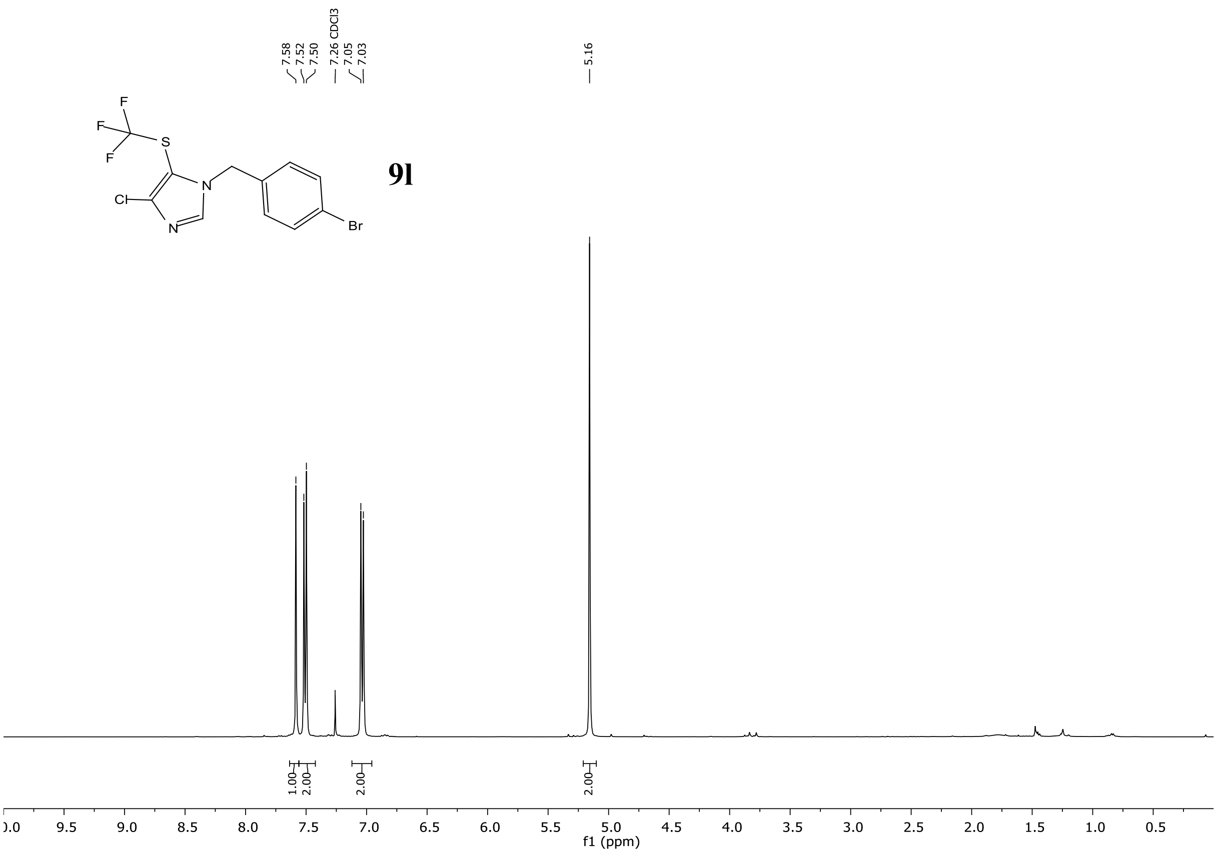

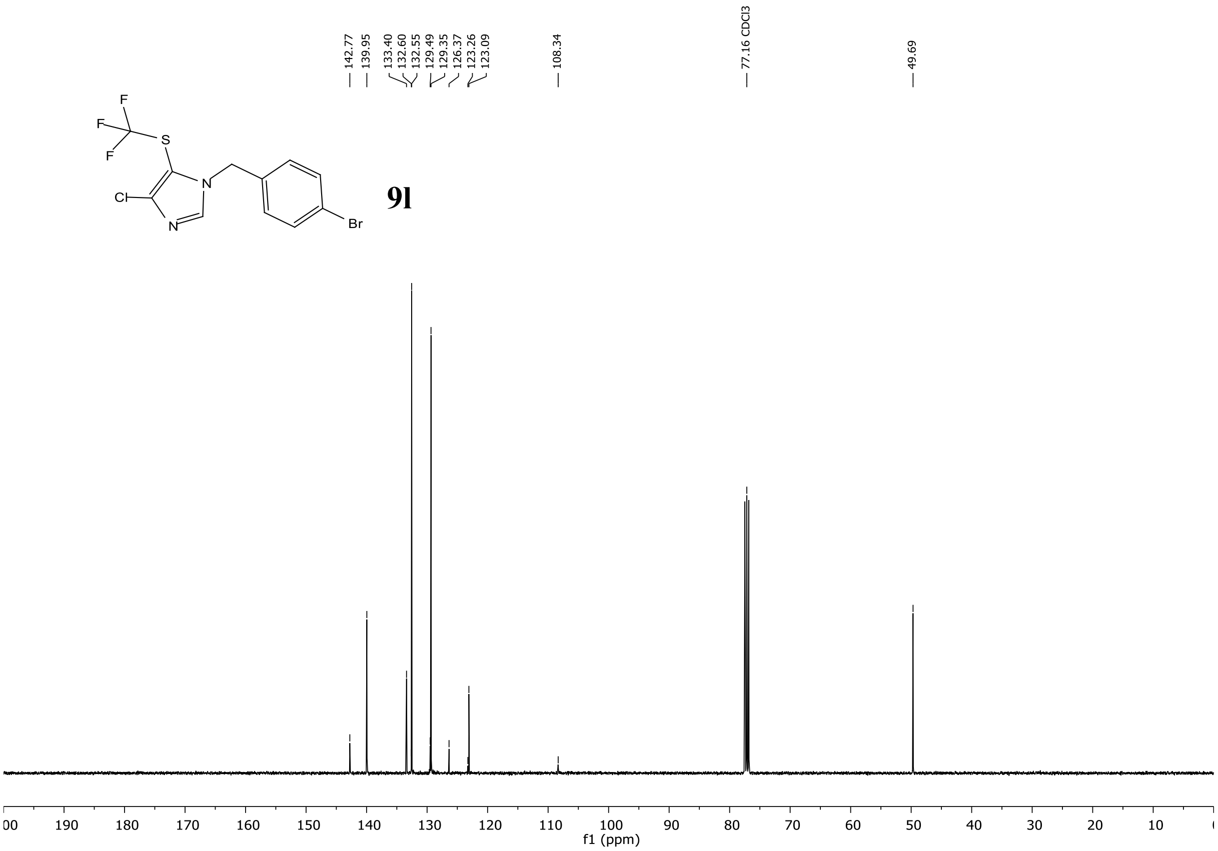

1-Methyl-5-((trifluoromethyl)thio)-1*H*-imidazole (9f)

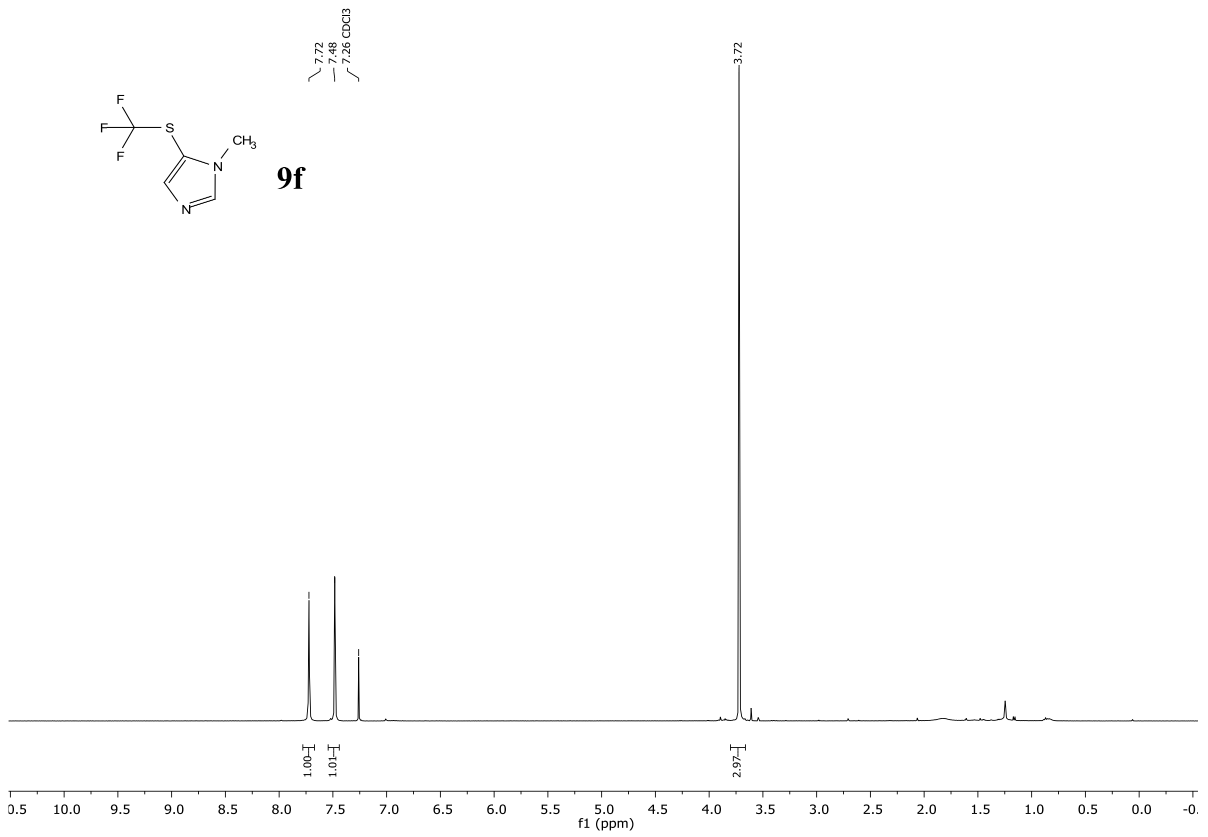

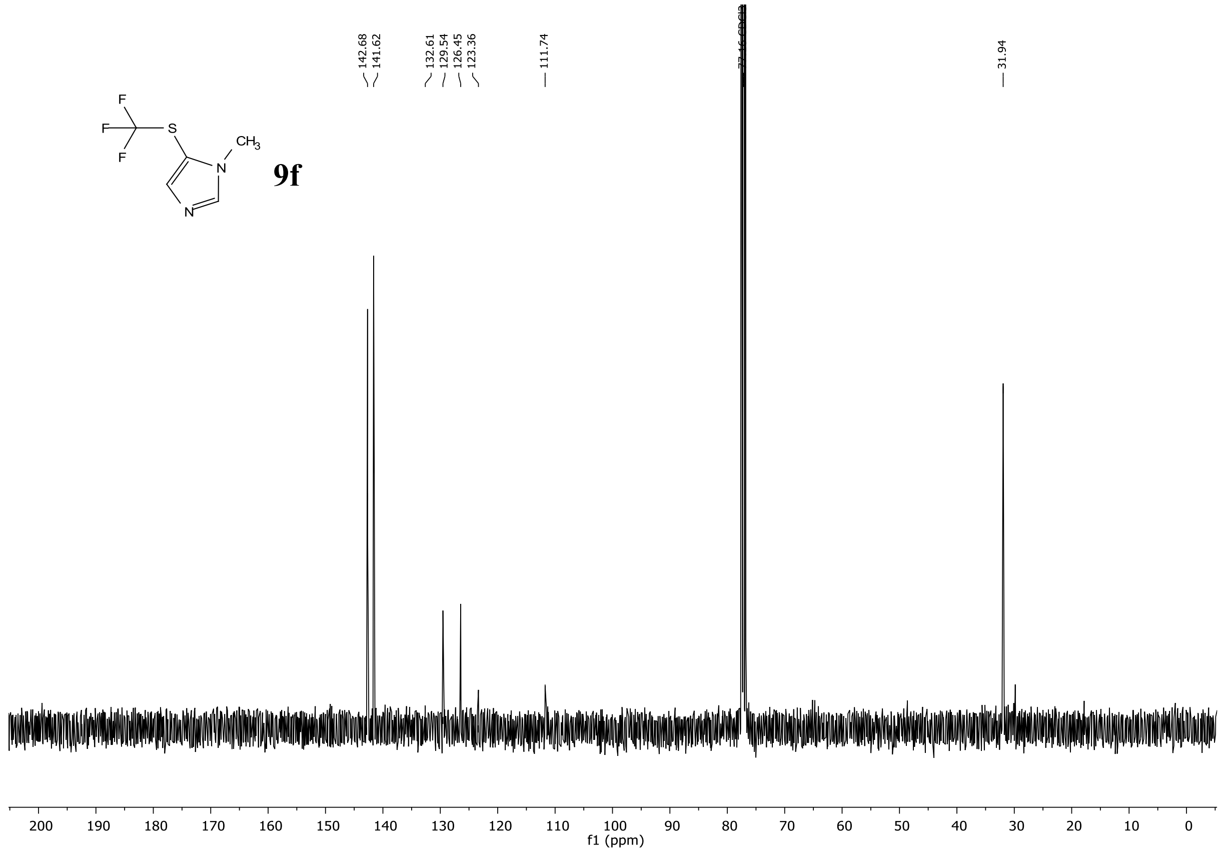

1,4-Dimethyl-2-((trifluoromethyl)thio)-1*H*-imidazole (9g)

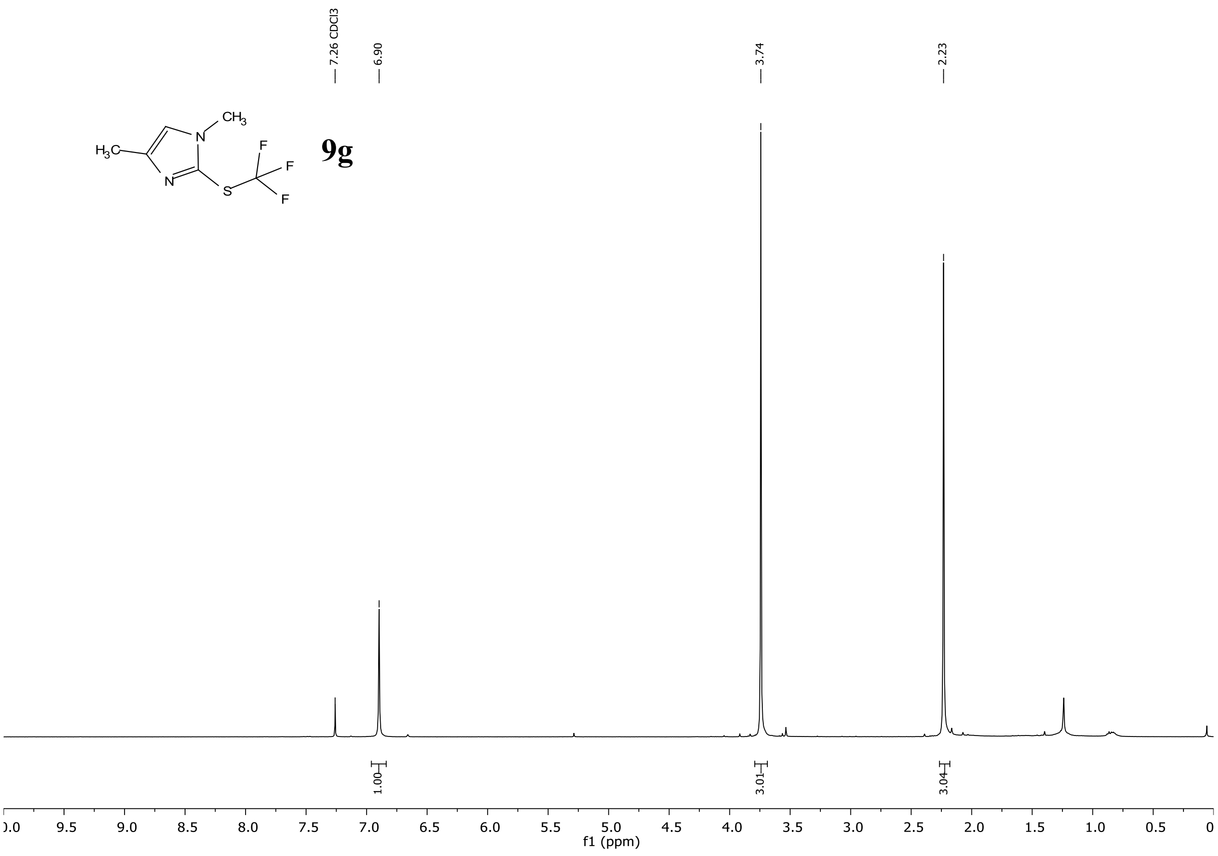

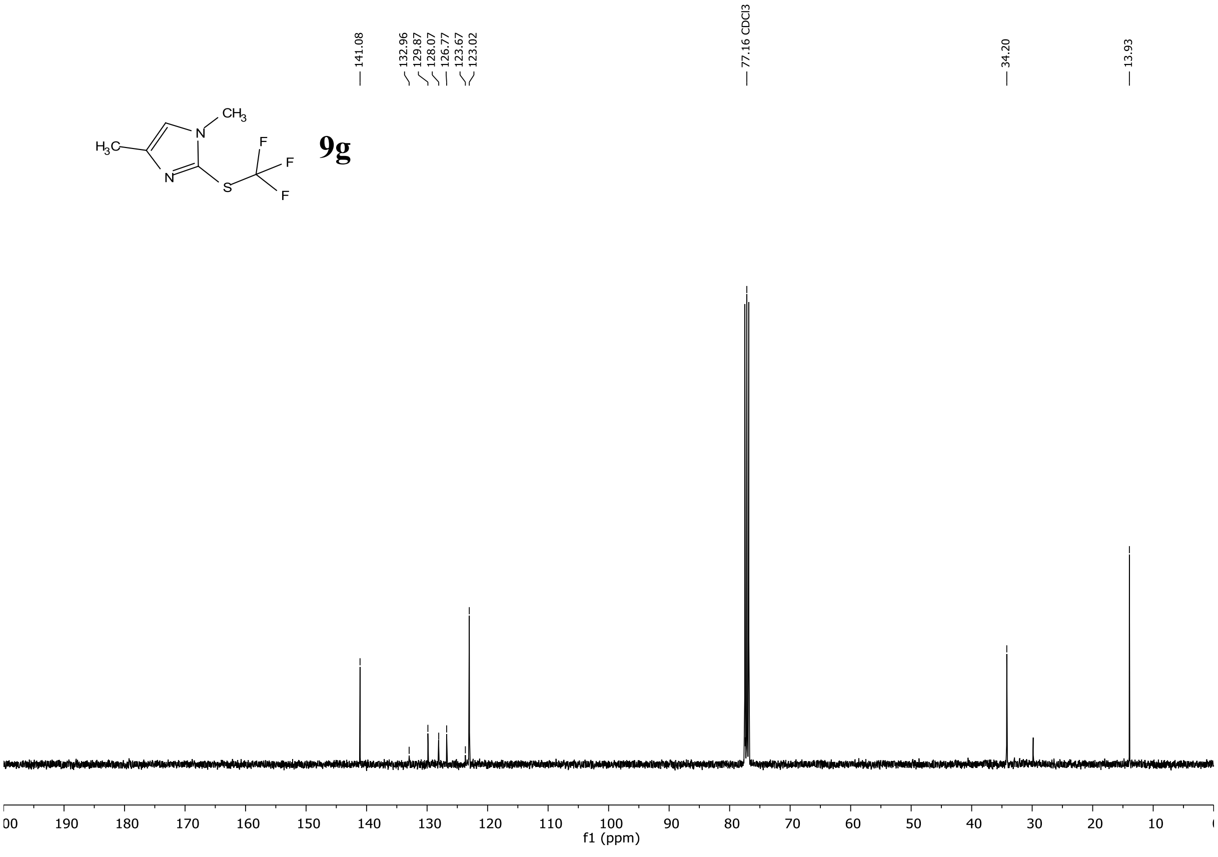

1-Ethyl-2-methyl-5-((trifluoromethyl)thio)-1*H*-imidazole (9h)

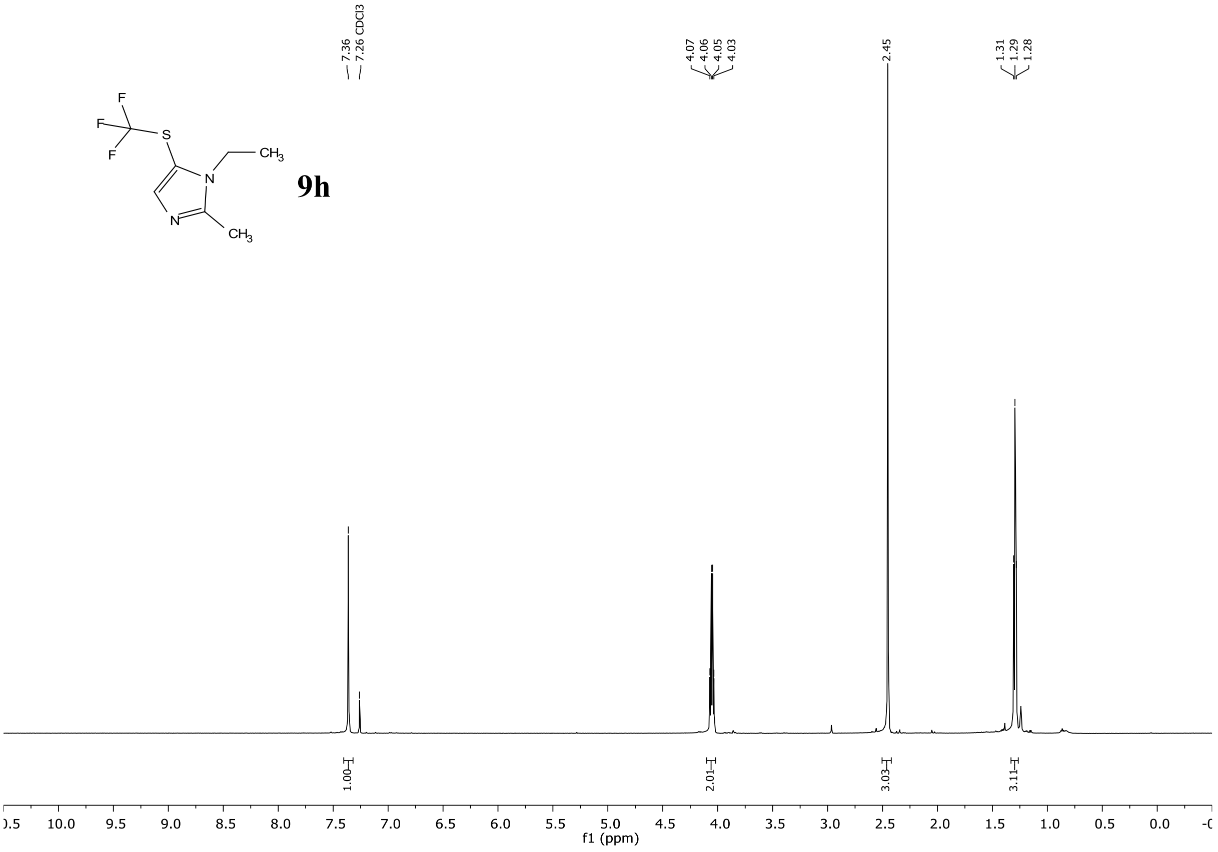

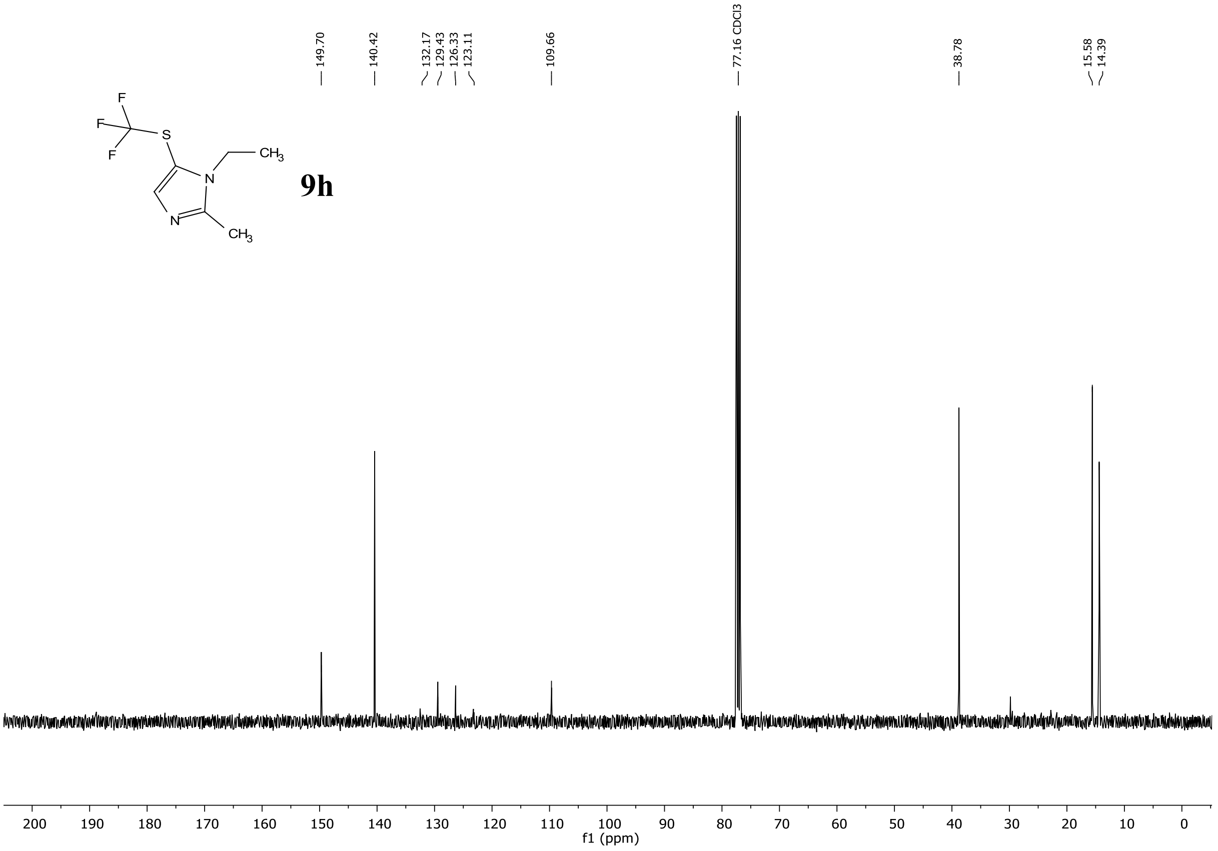

4-Chloro-1-((perfluorophenyl)methyl)-5-((trifluoromethyl)thio)-1*H*-imidazole (9m)

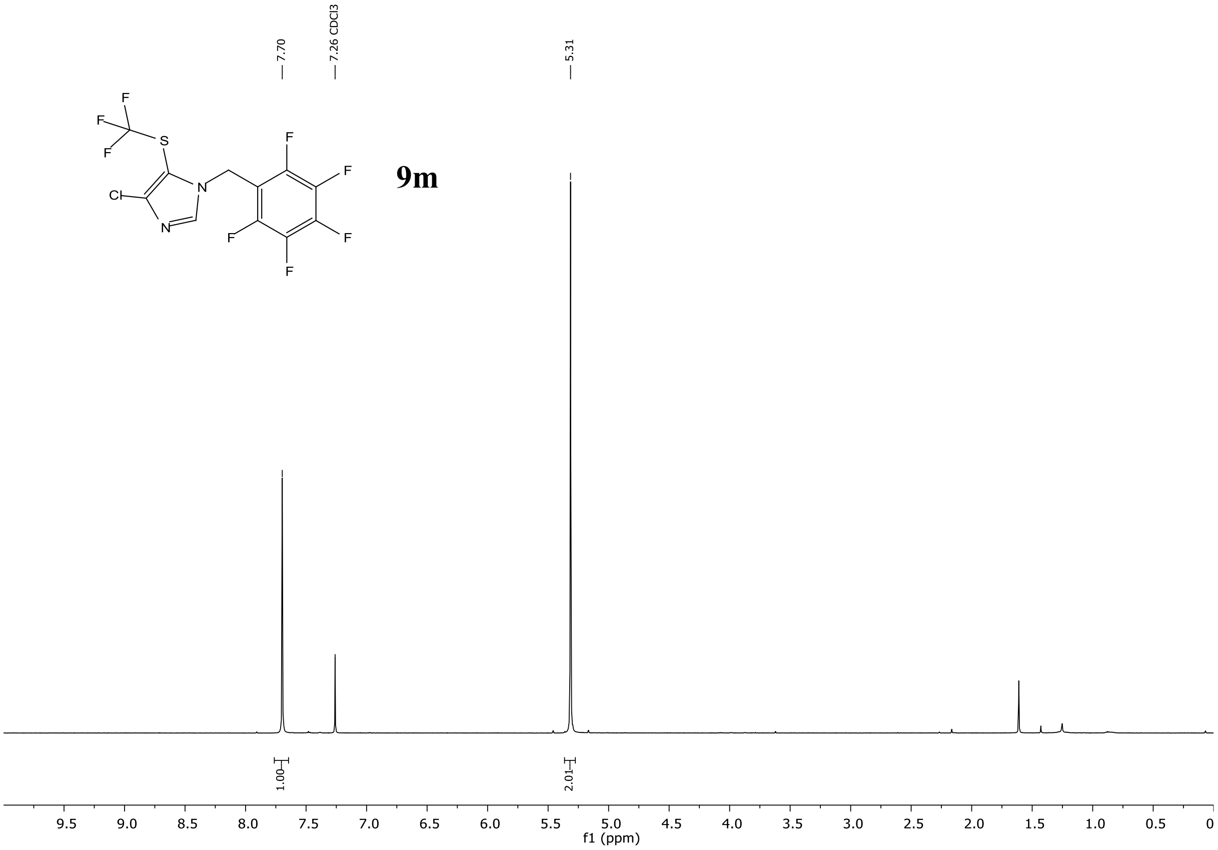

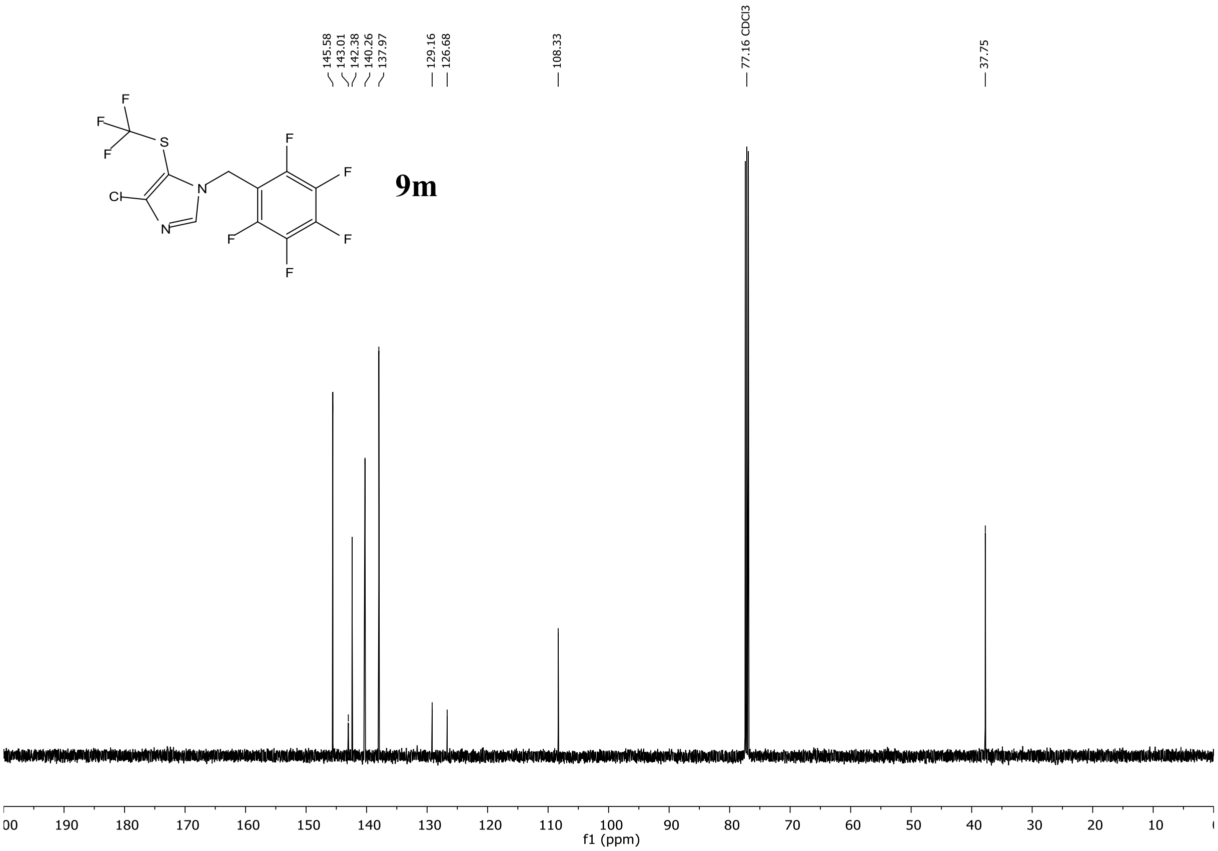

4-Chloro-5-((trifluoromethyl)thio)-1-(4-((trifluoromethyl)thio)benzyl)-1*H*-imidazole (9n)

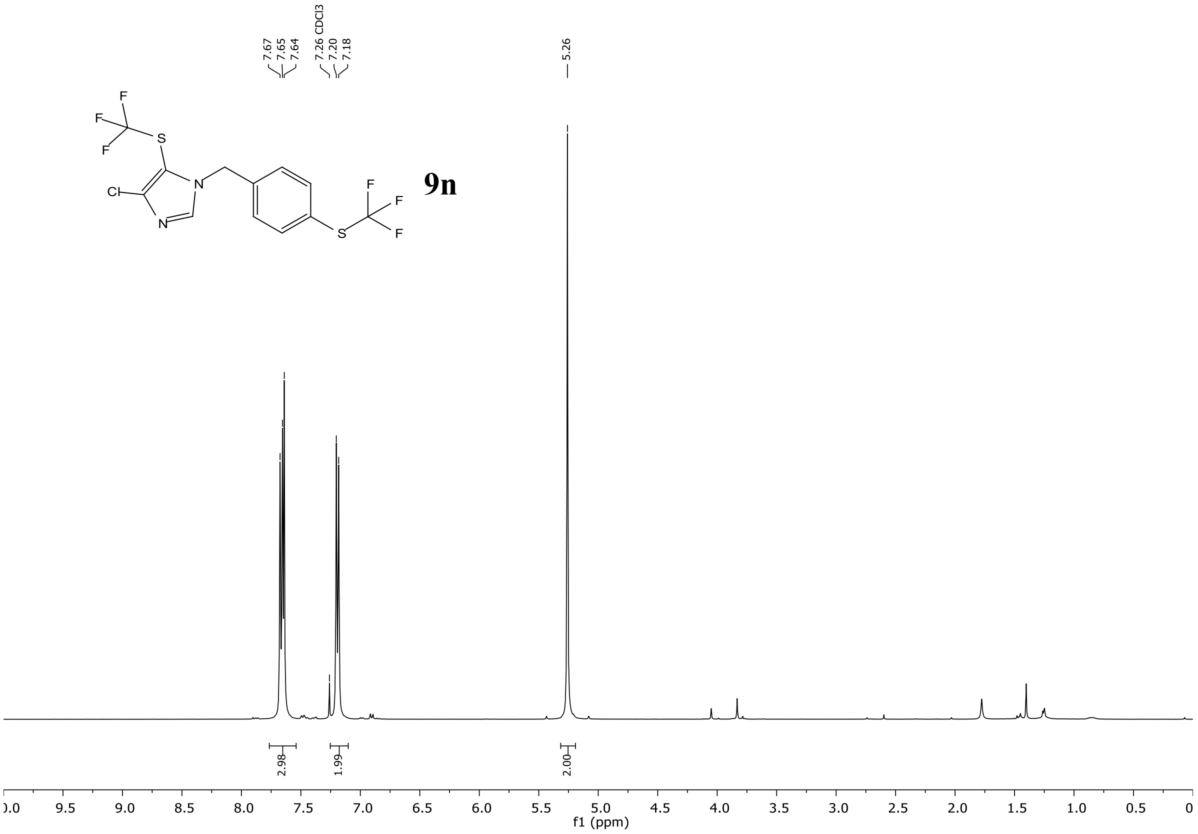

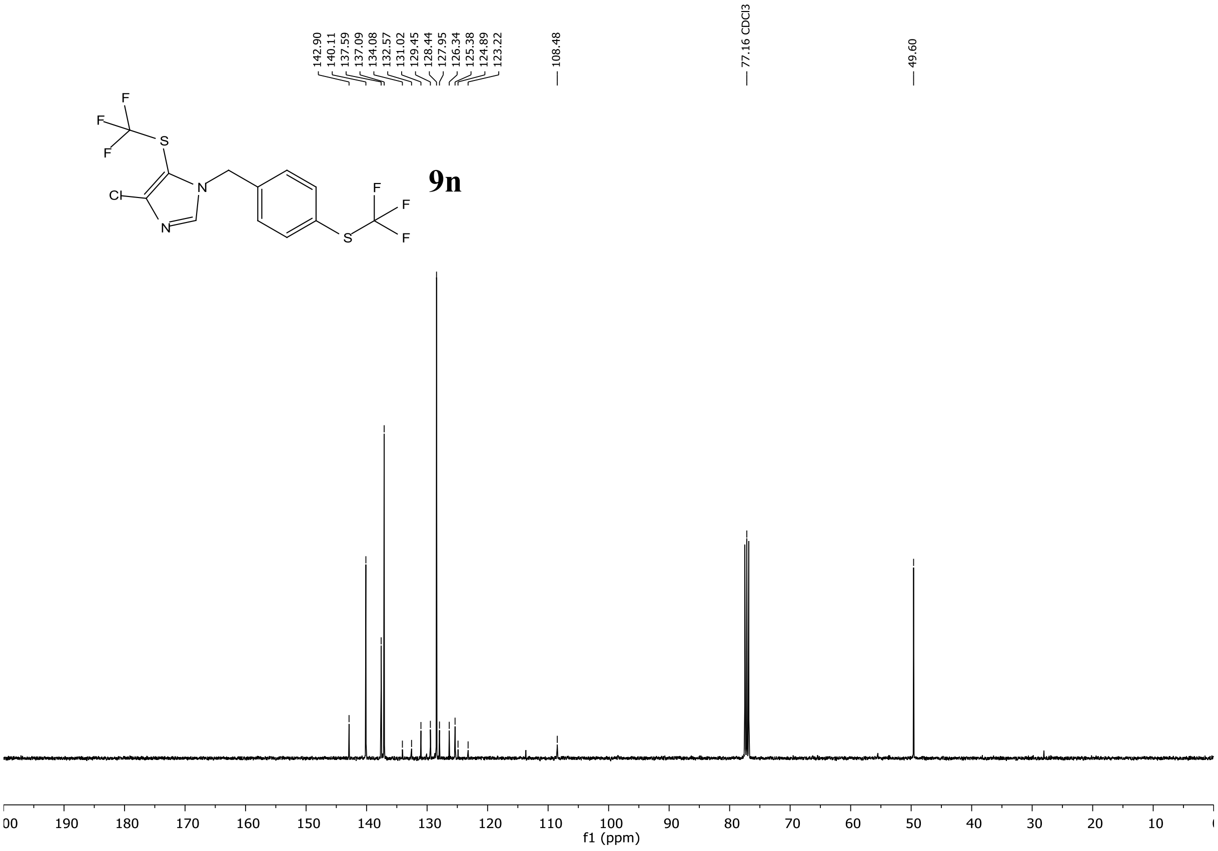

4-Chloro-1-(3-methoxypropyl)-5-((trifluoromethyl)thio)-1*H*-imidazole (9p)

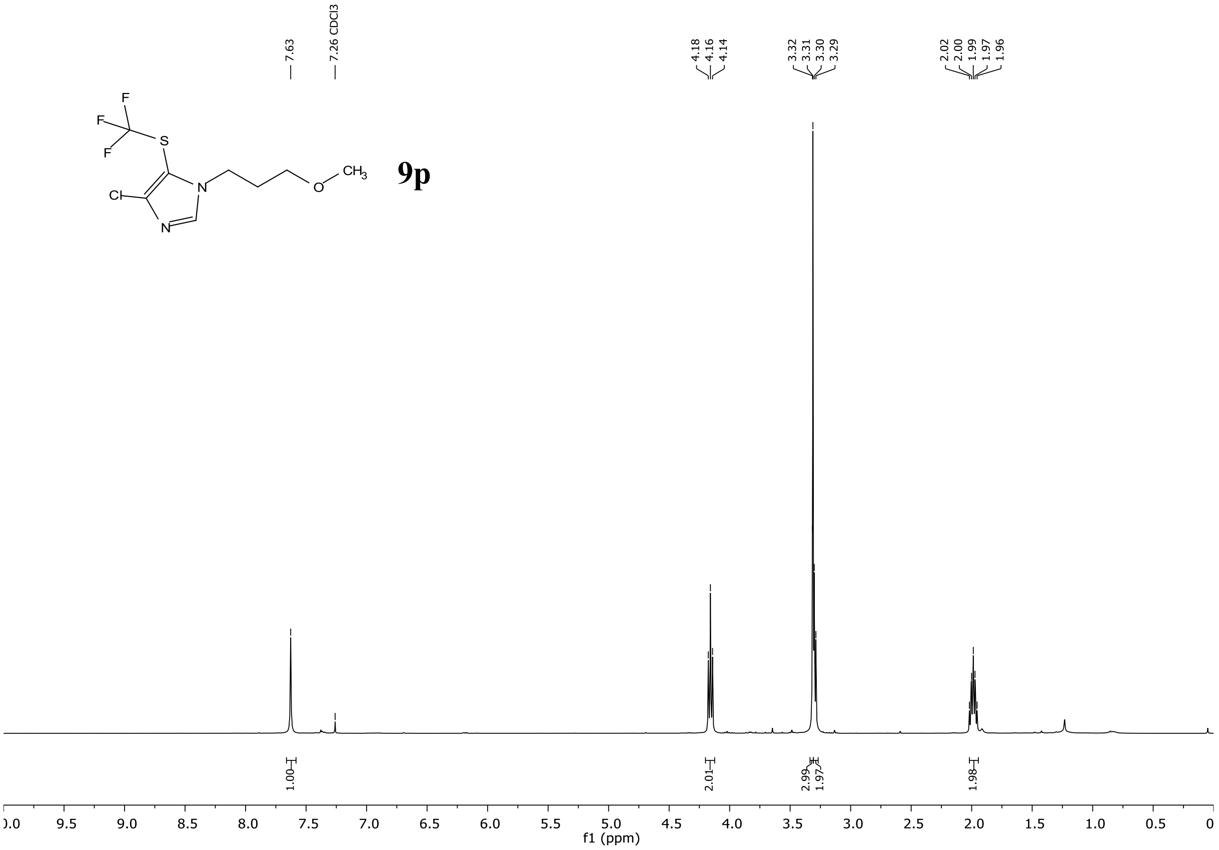

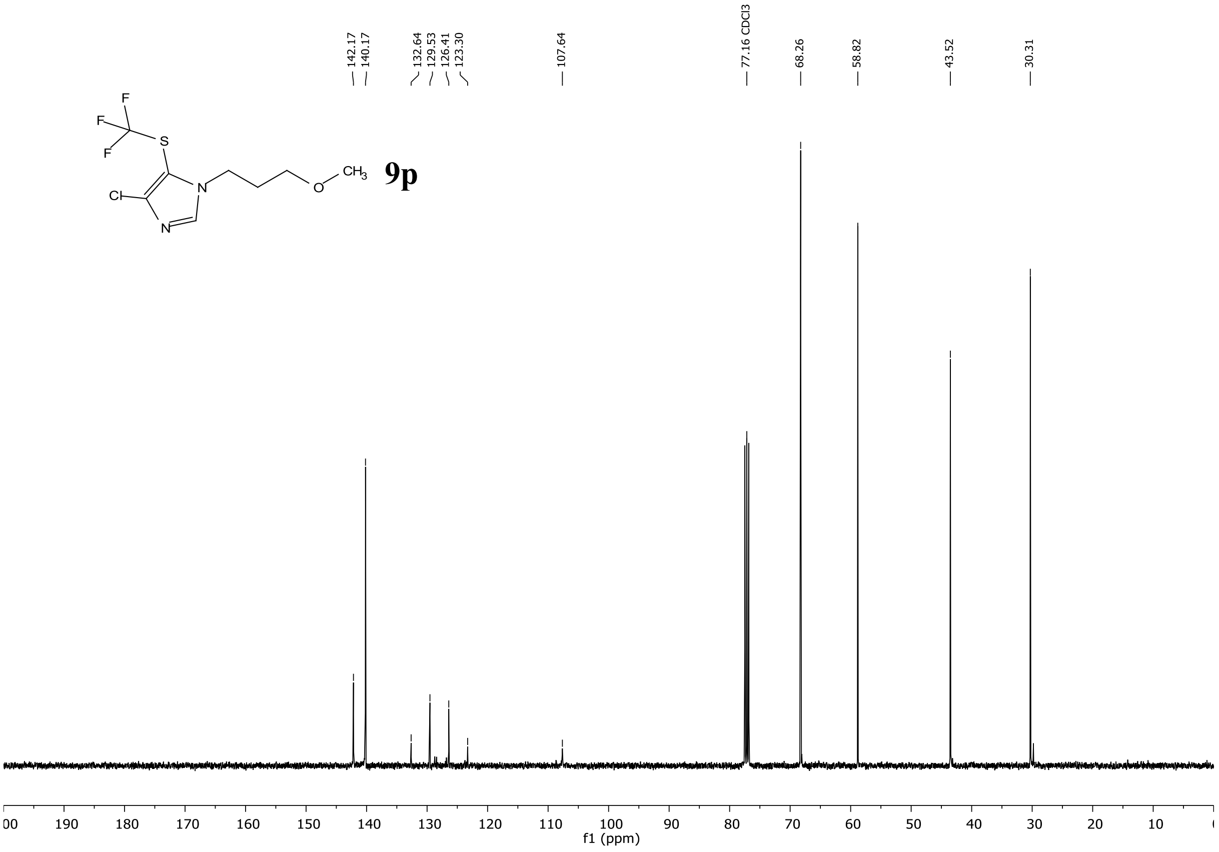

Tert-butyl 4-((4-chloro-5-((trifluoromethyl)thio)-1*H*-imidazol-1-yl)methyl)benzoate (9o)

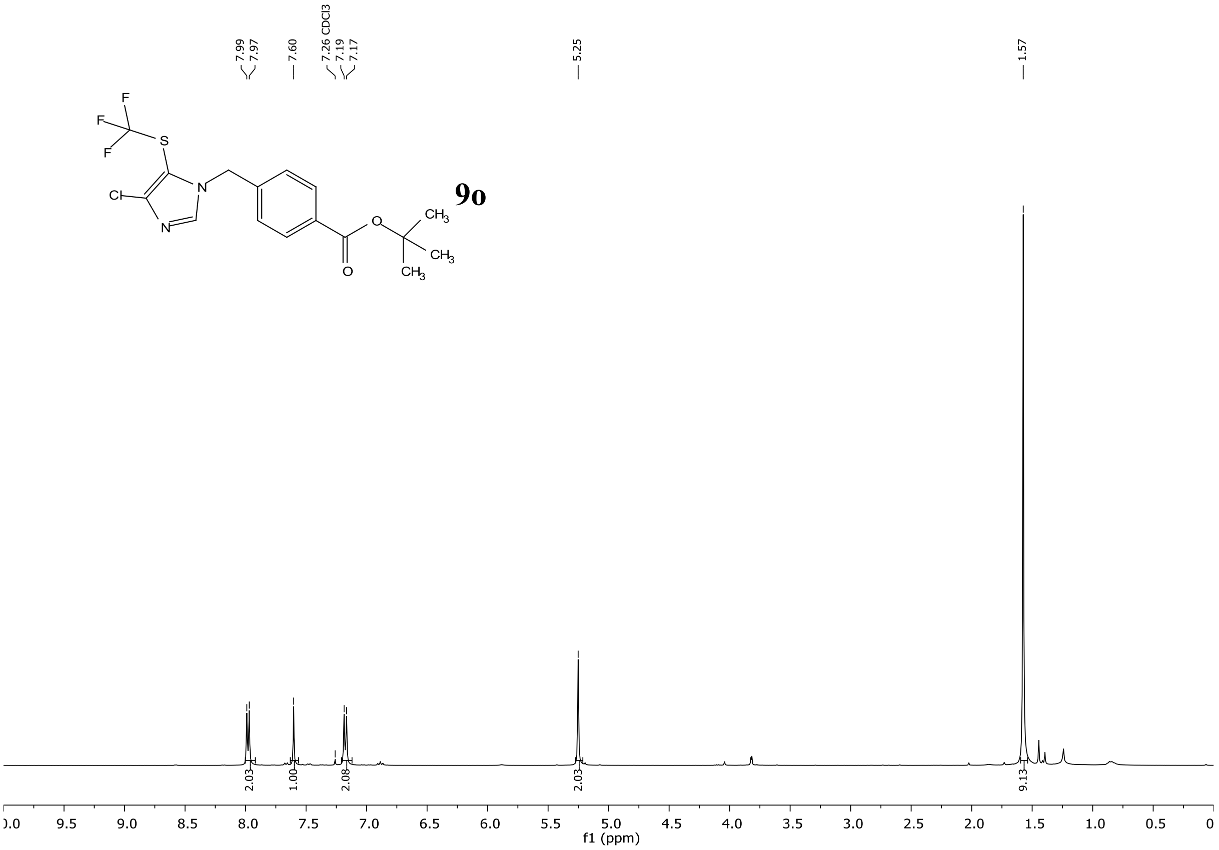

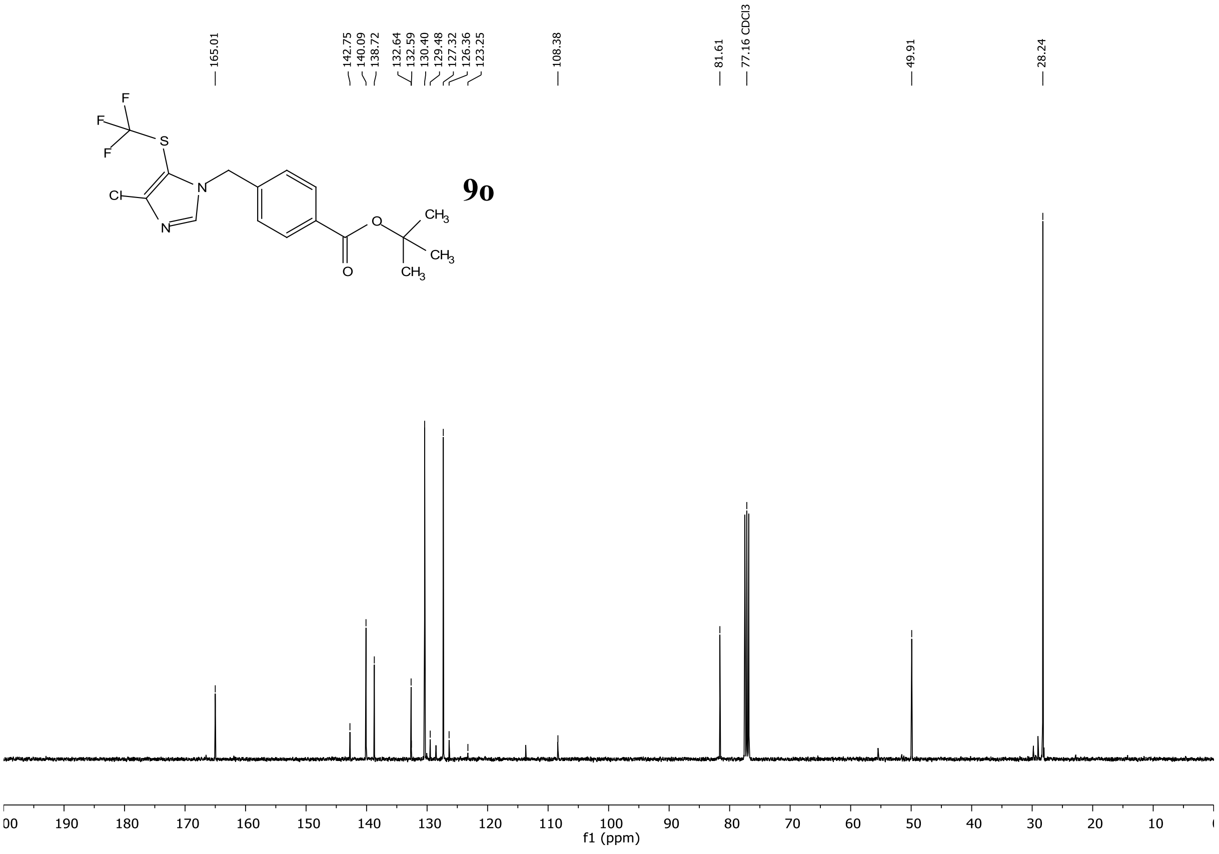

3-(4-Chloro-5-((trifluoromethyl)thio)-1*H*-imidazol-1-yl)dihydrofuran-2(3H)-one (9e)

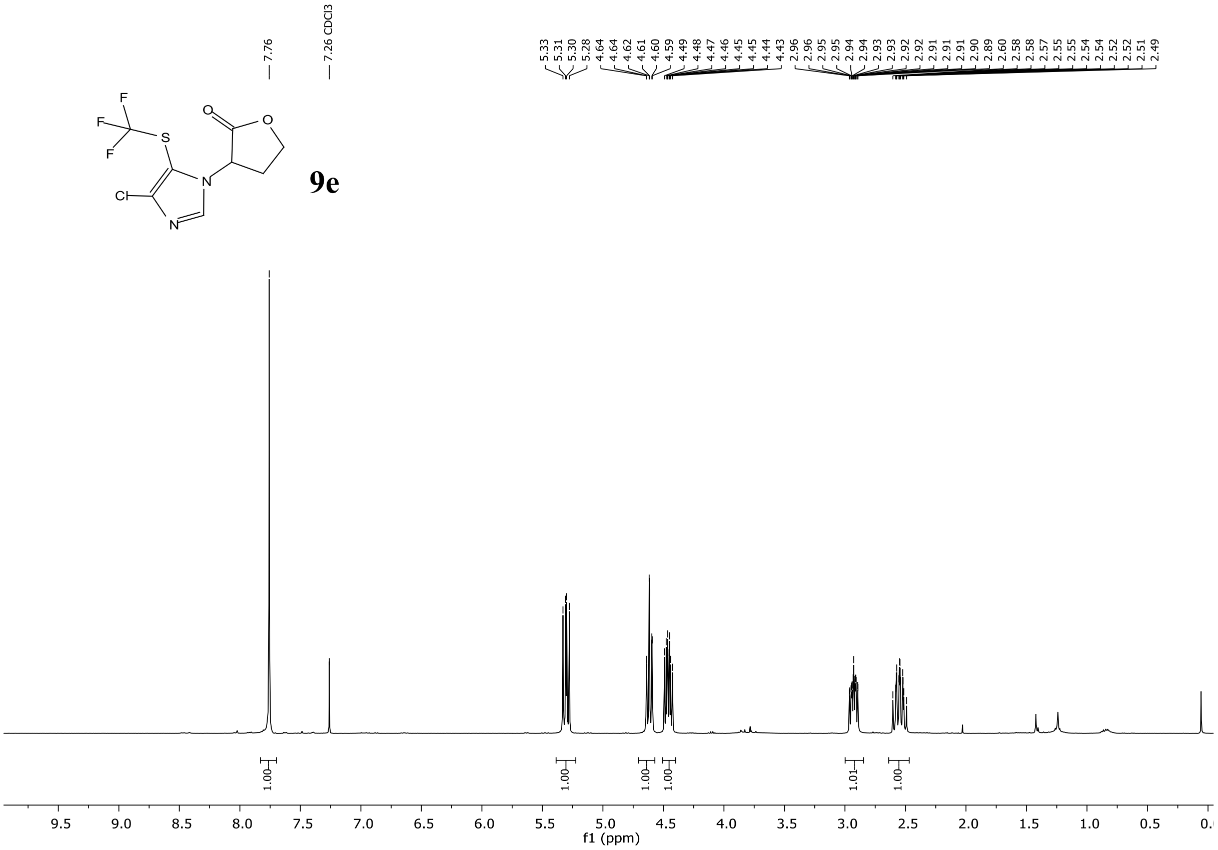

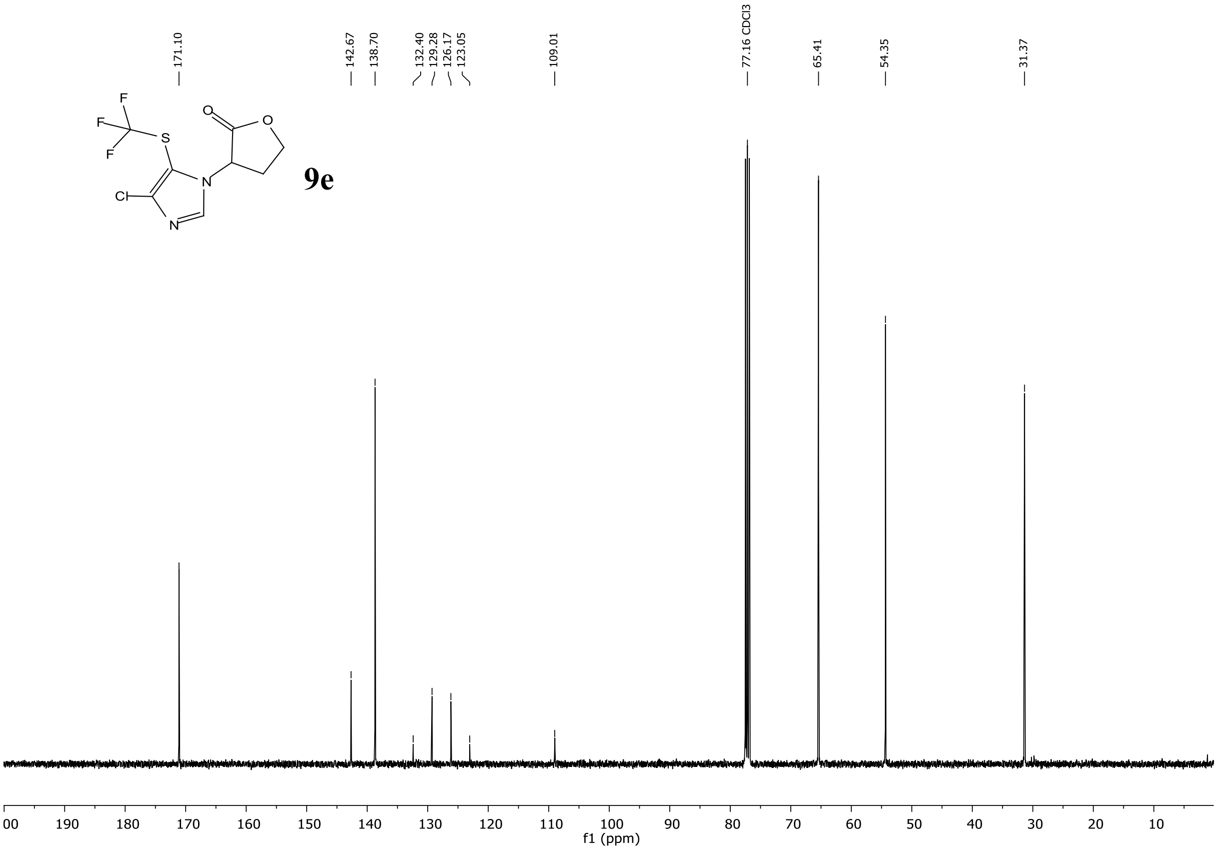

2-(4-Chloro-5-((trifluoromethyl)thio)-1*H*-imidazol-1-yl)ethan-1-ol (9r)

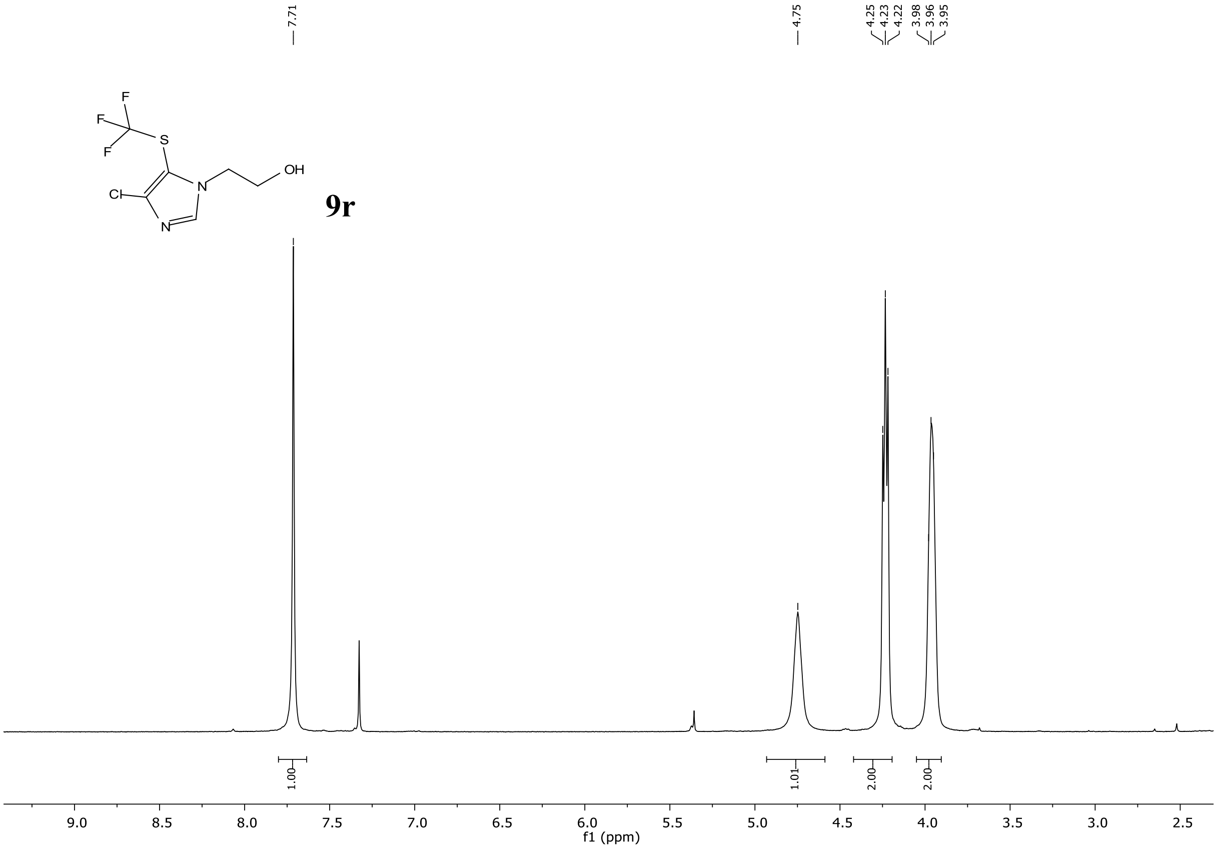

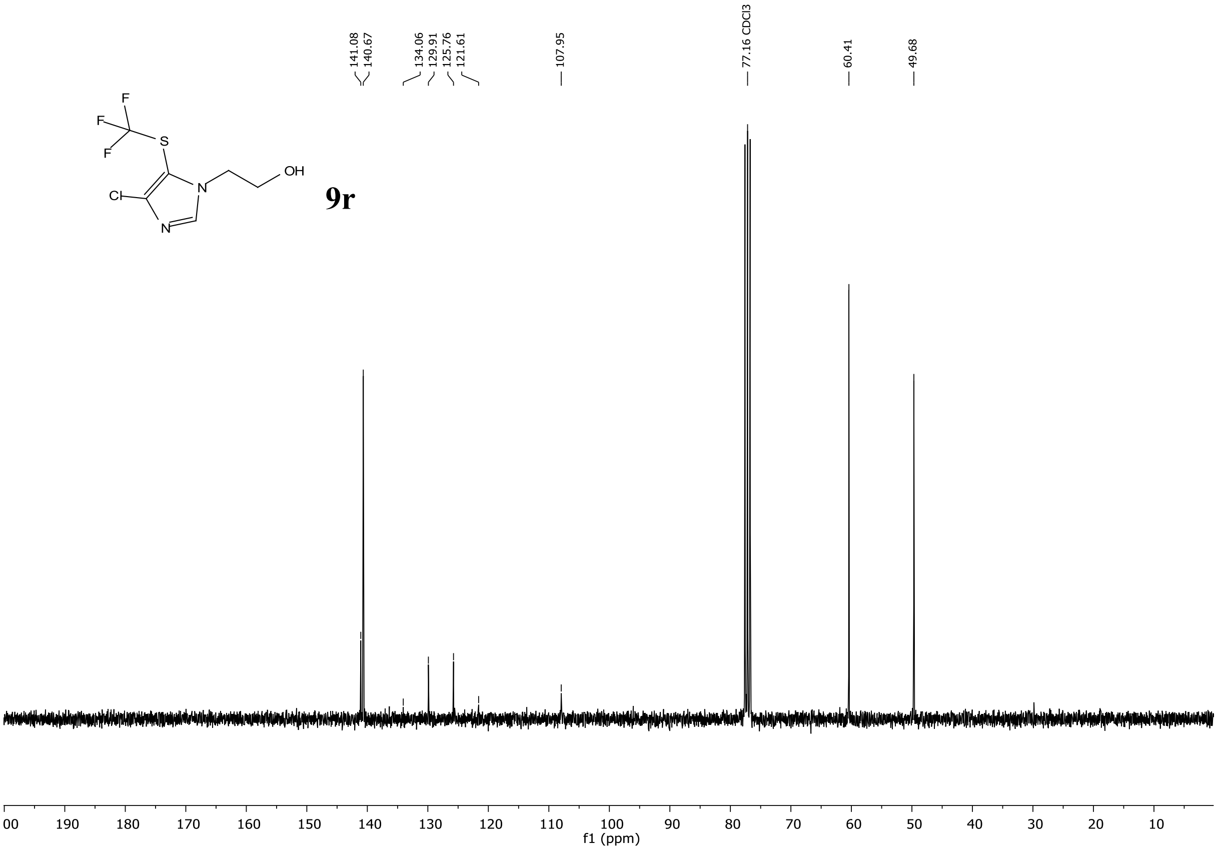

Methyl (*S*)-2-((tert-butoxycarbonyl)amino)-3-(5-((trifluoromethyl)thio)-1*H*-imidazol-1-yl)propanoate (9d)

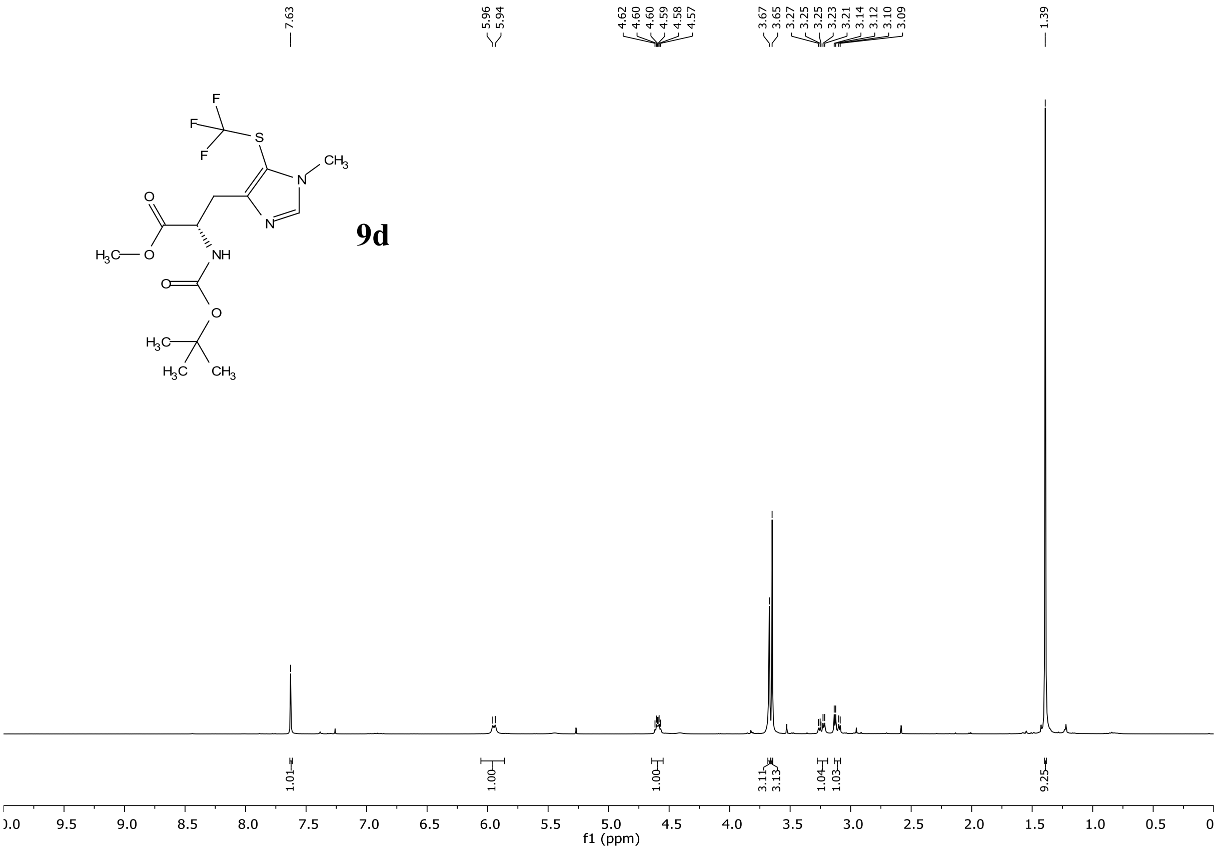

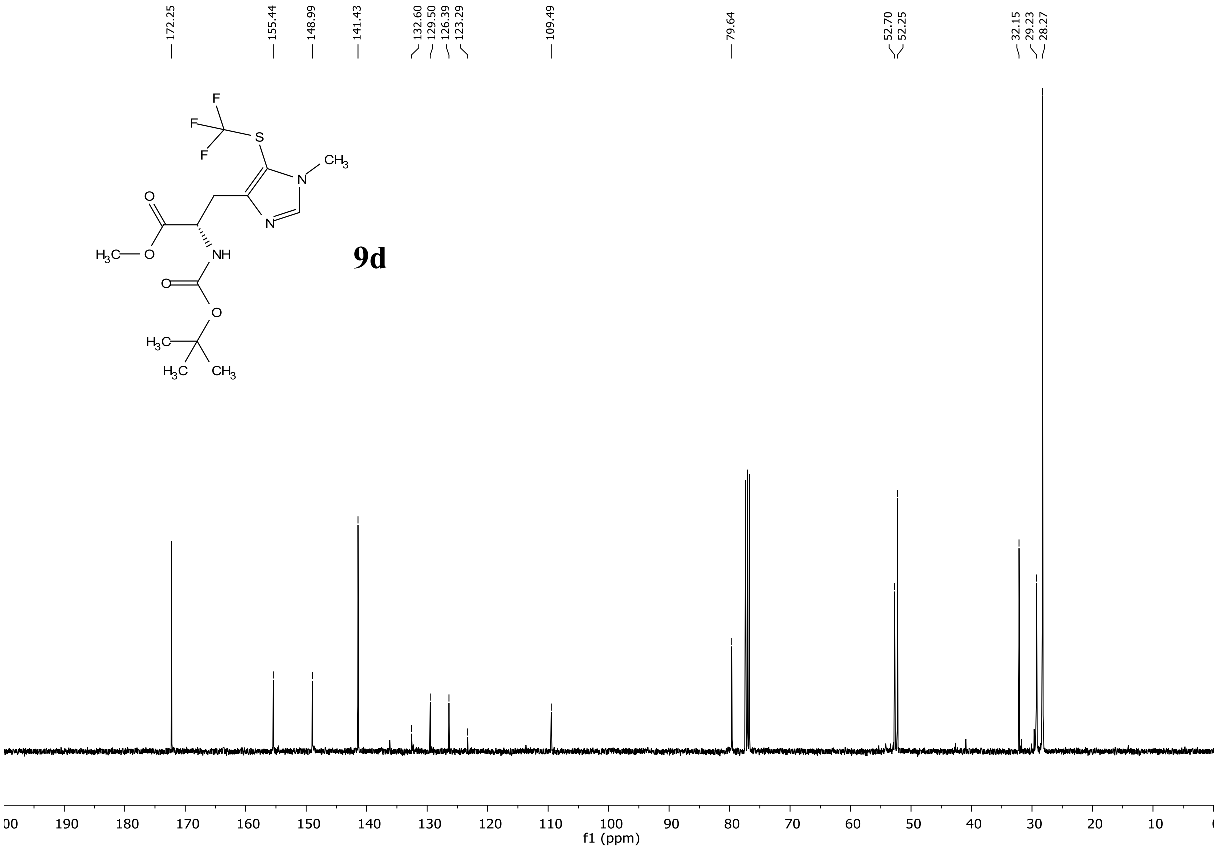

4-(4-Chloro-5-((trifluoromethyl)thio)-1*H*-imidazol-1-yl)butanenitrile (9q)

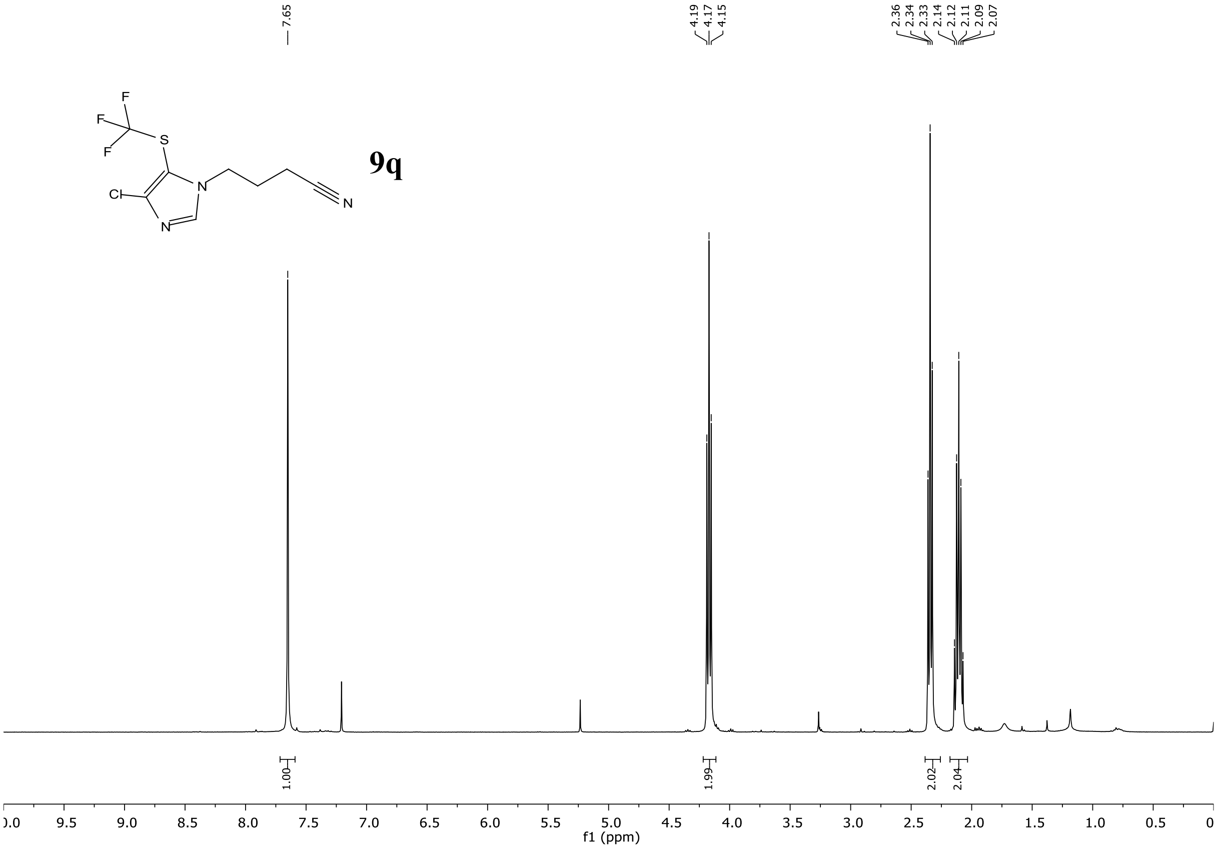

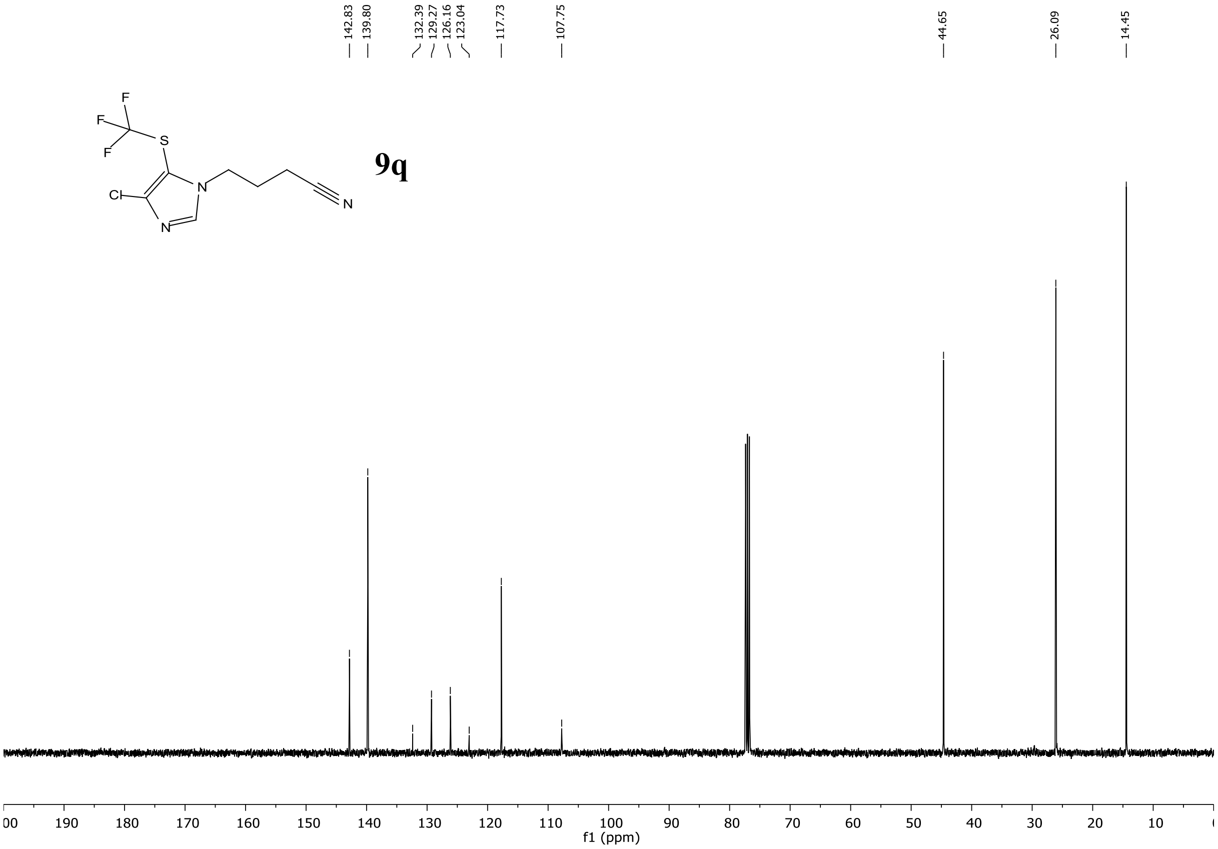

2-((4-Chloro-5-((trifluoromethyl)thio)-1*H*-imidazol-1-yl)methyl)pyridine (9s)

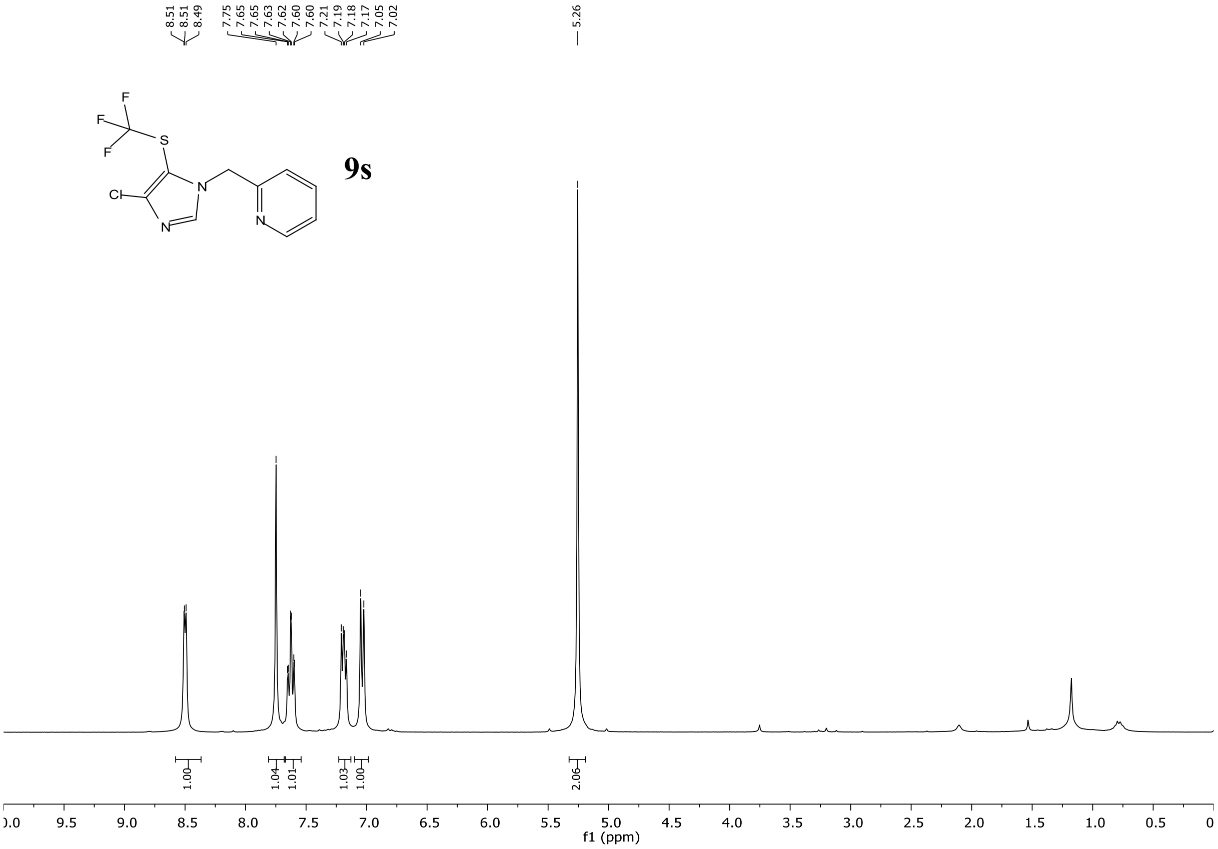

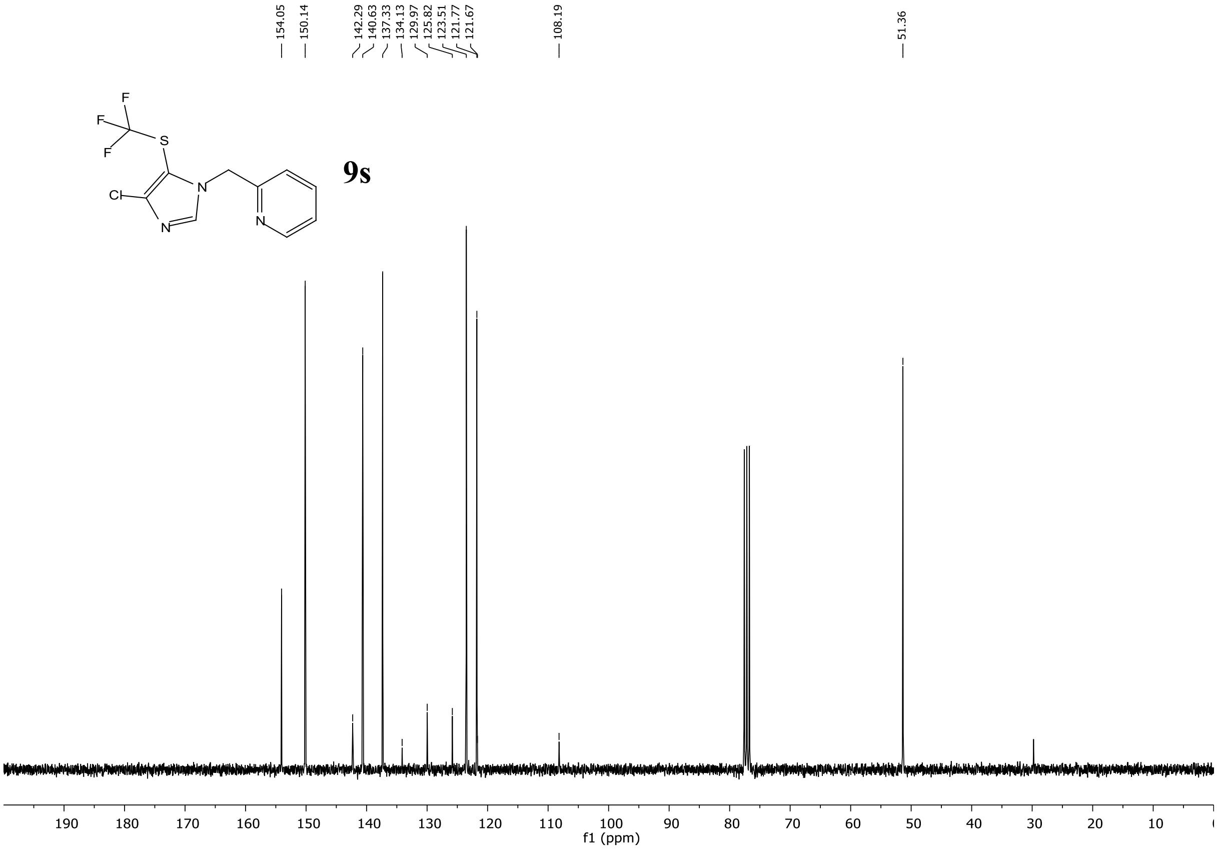

Diethyl (3-(4-chloro-5-((trifluoromethyl)thio)-1*H*-imidazol-1-yl)propyl)phosphonate (9t)

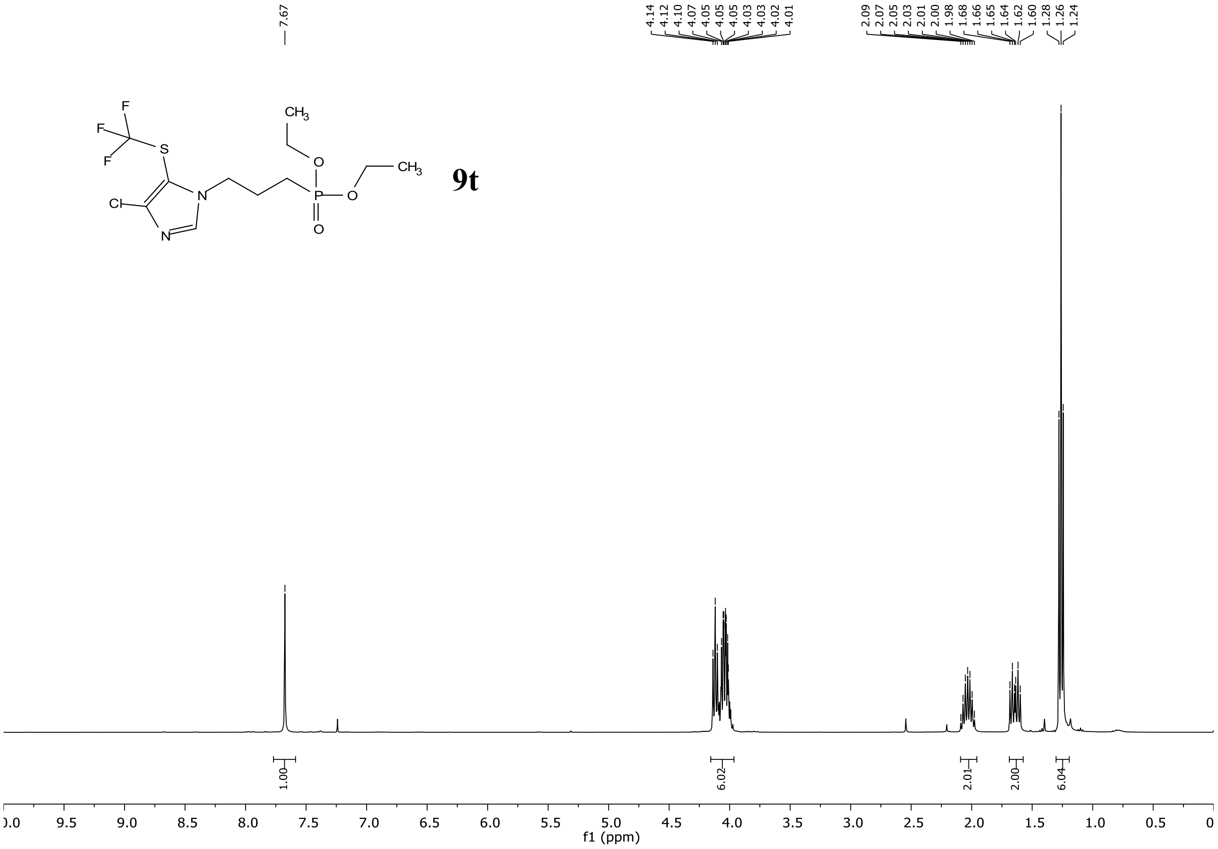

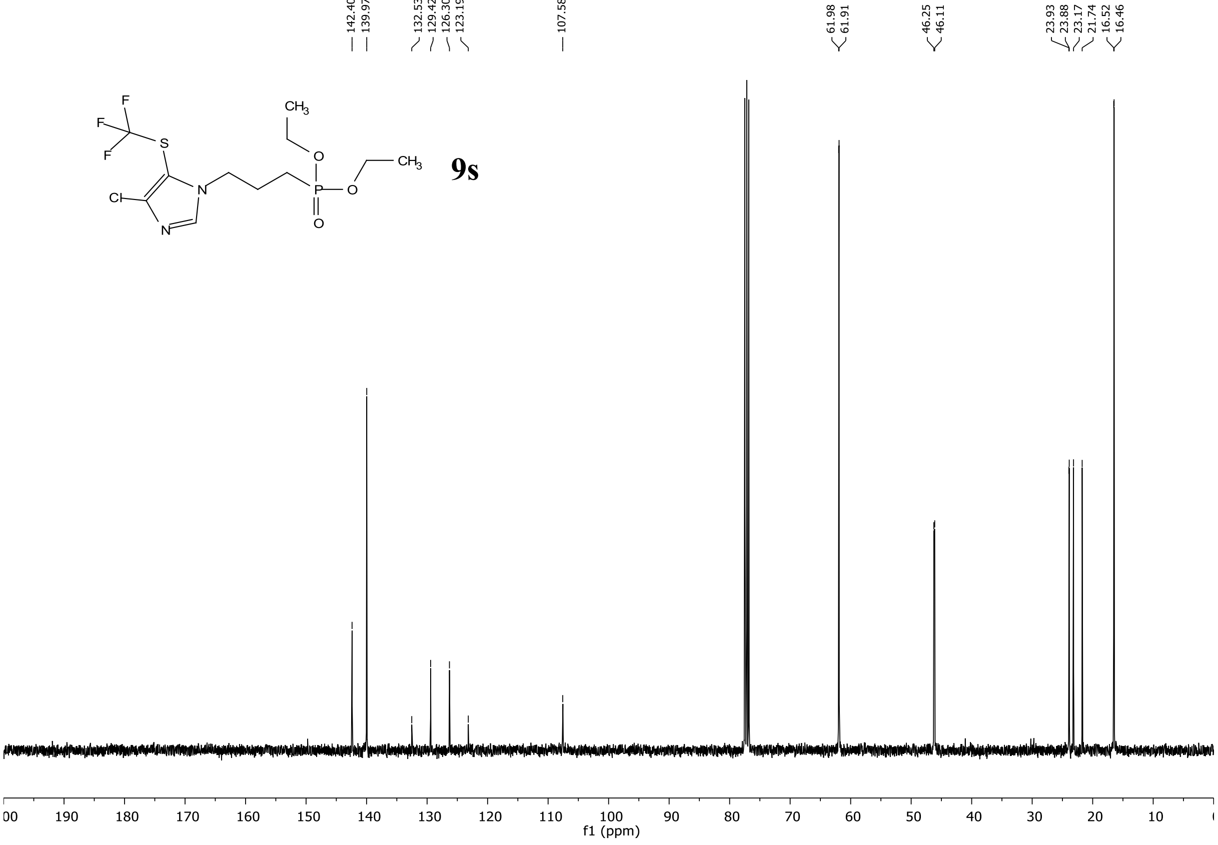

3,4-Dichloro-1-methyl-2-phenyl-5-((trifluoromethyl)thio)-1*H*-pyrrole (9u)

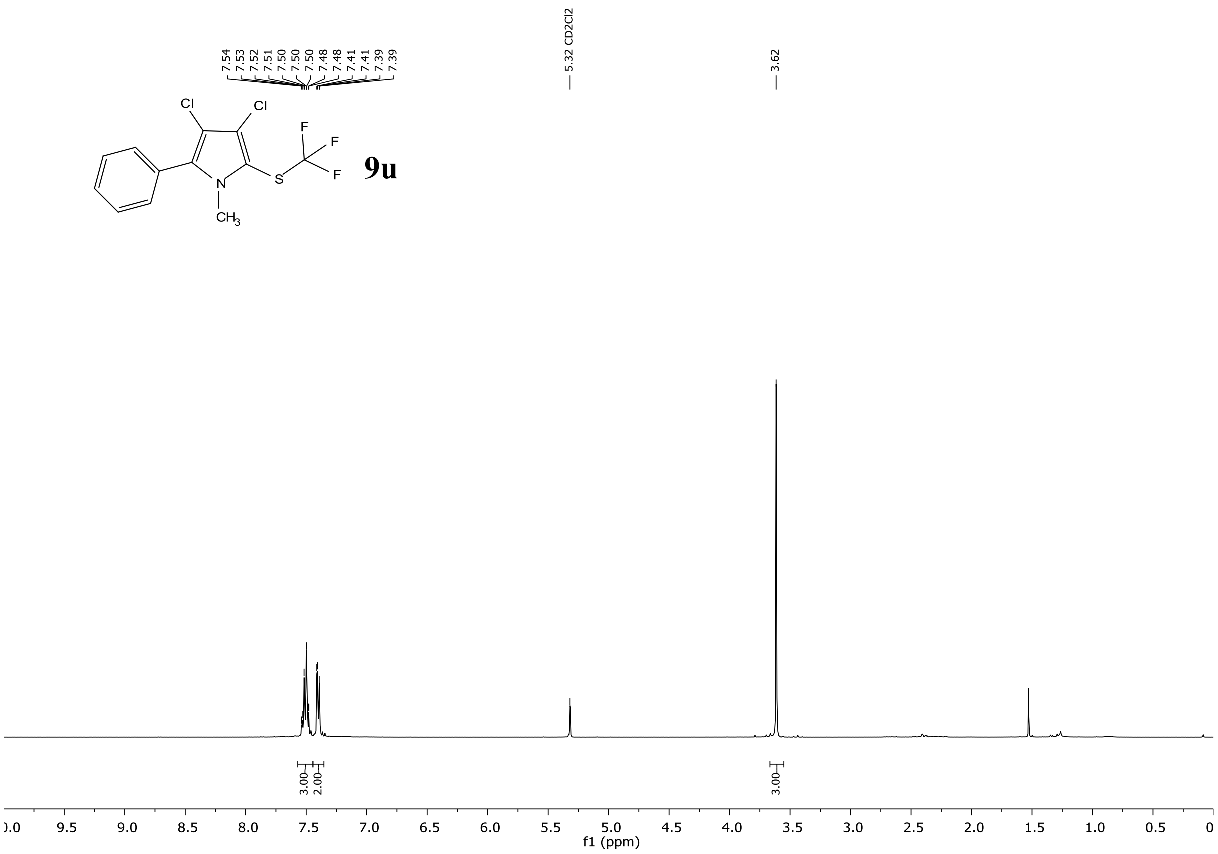

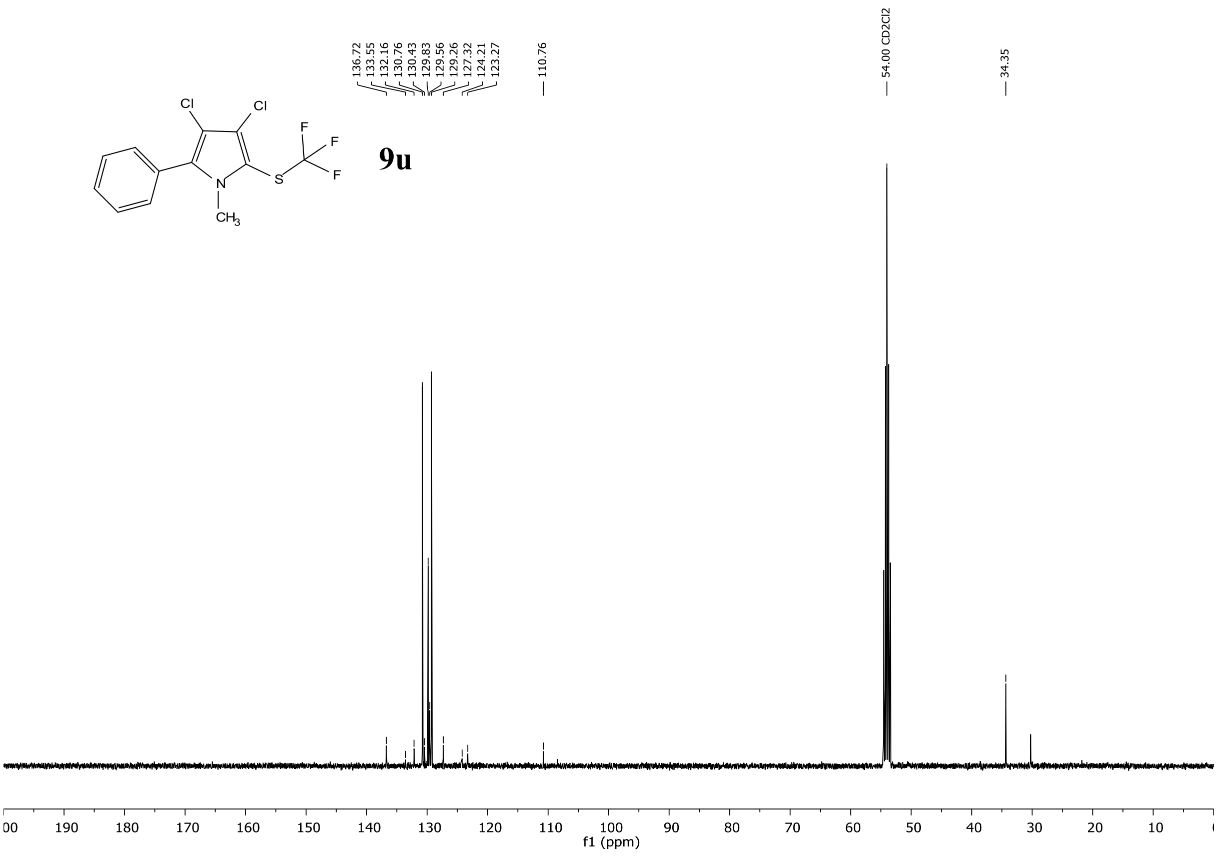

3,4-Dibromo-1-methyl-2-phenyl-5-((trifluoromethyl)thio)-1*H*-pyrrole (9v)

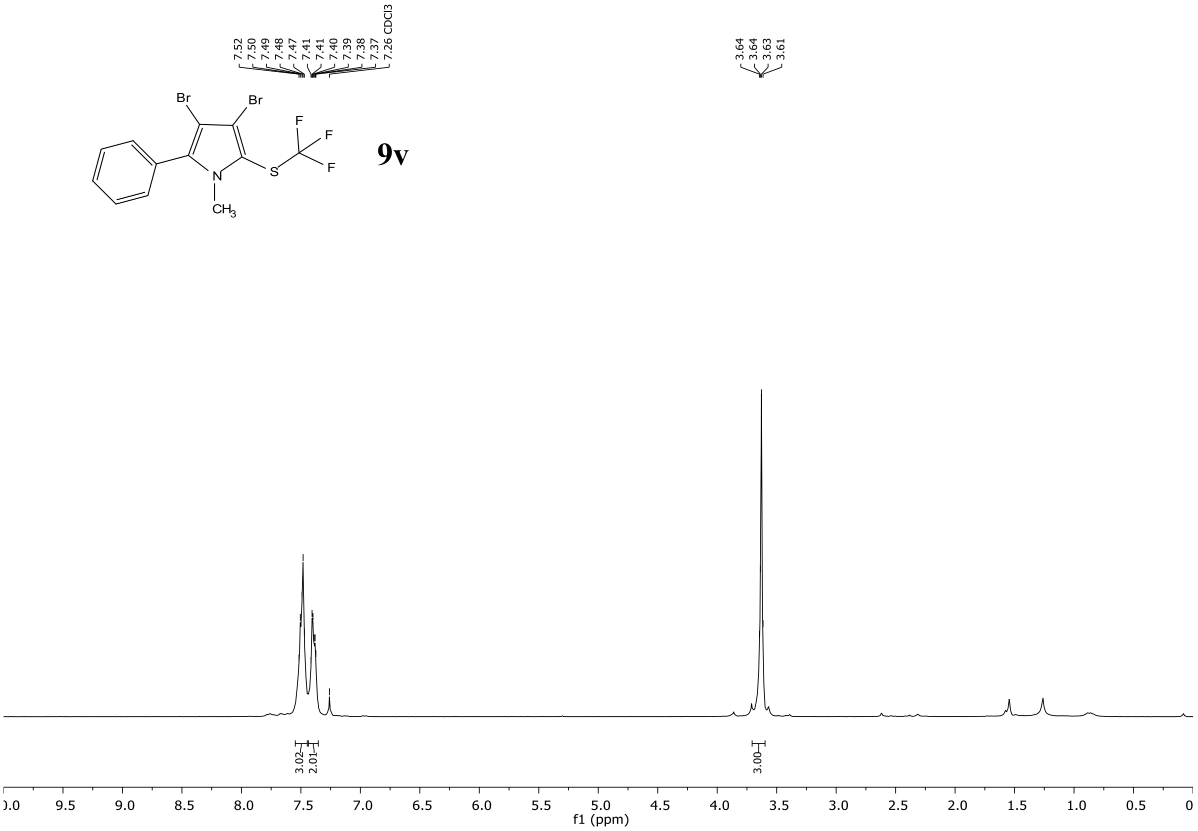

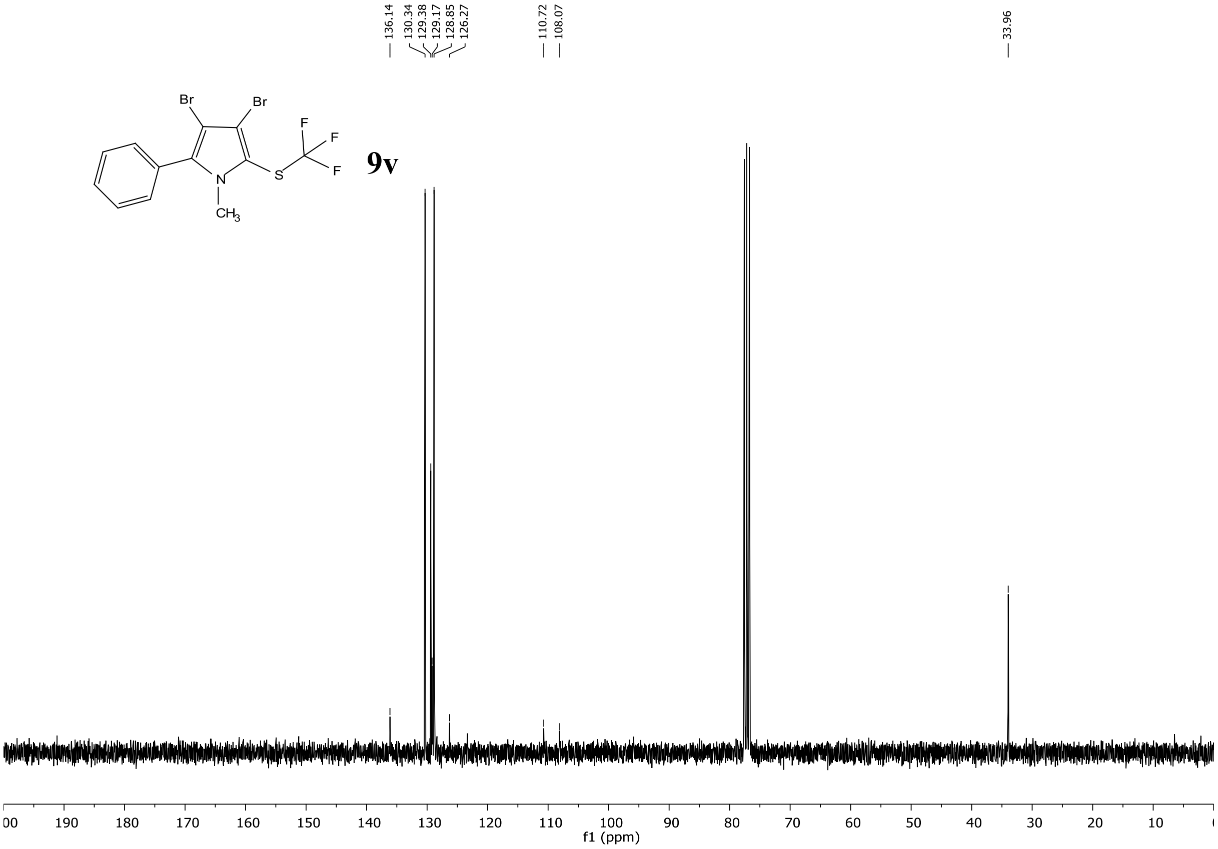

12. References

1. APEX3: Bruker AXS Inc., Madison, Wisconsin, USA, 2016.
2. SAINT: Bruker AXS Inc. Madison, Wisconsin, USA, 2015.
3. SADABS Bruker AXS Inc. Madison, Wisconsin, USA, 2015.
4. Sheldrick, G. M. SHELXT – Integrated Space-Group and Crystal-Structure Determination. *Acta Cryst.* **A71**, 3–8 (2015).
5. Sheldrick, G.M. Crystal Structure Refinement with SHELXL. *Acta Cryst.* C71, 3–8 (2015).
6. XP: Interactive Molecular Graphics, Version 5.1, Bruker AXS Inc., Madison, Wisconsin, USA, (1998).
7. Neese, F. The ORCA Program System. *Comput. Mol. Sci.* **2**, 73–78 (2012).
8. Guo, Y., Sivalingam, K., Valeev, E. F. & Neese, F. SparseMaps—A Systematic Infrastructure for Reduced-Scaling Electronic Structure Methods. III. Linear-Scaling Multireference Domain-Based Pair Natural Orbital N-Electron Valence Perturbation Theory. *J. Chem. Phys*. **144**, 094111 (2016).
9. Schäfer, A., Horn, H. & Ahlrichs, R. Fully Optimized Contracted Gaussian Basis Sets for Atoms Li to Kr. *J. Chem. Phys*. **97**, 2571–2577 (1992).
10. a) Barone, V. & Cossi, M. Quantum Calculation of Molecular Energies and Energy Gradients in Solution by a Conductor Solvent Model. *J. Phys. Chem. A* **102**, 1995–2001 (1998); b) Cossi, M., Rega, N., Scalmani, G. & Barone, V. Energies, Structures, and Electronic Properties of Molecules in Solution with the C-PCM Solvation Model. *J. Comput. Chem.* **24**, 669–681 (2003).
11. Grimme, S., Antony, J., Ehrlich, S. & Krieg, H. A Consistent and Accurate ab initio Parametrization of Density Functional Dispersion Correction (DFT-D) for the 94 Elements H-Pu. *J. Chem. Phys.* **132**, 154104 (2010).
12. Jmol: An Open-Source Java Viewer for Chemical Structures in 3D: www.jmol.org, 14.11.2025.
13. Roth, H. G., Romero, N. A. & Nicewicz, D. A. Experimental and Calculated Electrochemical Potentials of Common Organic Molecules for Applications to Single-Electron Redox Chemistry. *Synlett* **27**, 714–723 (2016).
14. Isse, A. A. & Gennaro, A. J. Absolute Potential of the Standard Hydrogen Electrode and the Problem of Interconversion of Potentials in Different Solvents. *J. Phys. Chem. B* **114**, 7894–7899 (2010).
15. Steiner, J. Combinatorische Aufgabe. *J. Reine Angew. Math.* **45**, 181–182 (1853).
16. MacDonald, J. A., Hout, M. C., Schmidt, J. An Algorithm to Minimize the Number of Blocks in Incomplete Block Designs. *Behav. Res. Methods* **52**, 1459–1468 (2020).
17. La Jolla Covering Repository Tables: https://ljcr.dmgordon.org/cover/table.html, 14.11.2025.
18. Lowry, M. S. et al. Single-Layer Electroluminescent Devices and Photoinduced Hydrogen Production from an Ionic Iridium(III) Complex. *Chem. Mater*. 17, 5712–5719 (2005).
19. Zhang, J. et al. Exploration of the Synthetic Potential of Electrophilic Trifluoromethylthiolating and Difluoromethylthiolating Reagents. *Angew. Chem. Int. Ed.* **57**, 12690–12695 (2018).
20. Xu, C. et al. Shen, Q. Palladium-Catalyzed Trifluoromethylthiolation of Aryl C–H Bonds. *Org. Lett*. **16**, 2046–2049 (2014).
21. Compton, R. G. & Banks, C. E. Understanding Voltammetry. (Imperial College Press, London, ed 2, 2011).
22. Housecroft, C. E. & Sharpe, A. G. Inorganic Chemistry. (Pearson, London, ed. 4, 2012).
23. Hanss, D., Freys, J. C., Bernardinelli, G. & Wenger, O. S. Cyclometalated Iridium(III) Complexes as Photosensitizers for Long-Range Electron Transfer: Occurrence of a Coulomb Barrier. *Eur. J. Org. Chem.* 4850–4859 (2009).
24. Slinker, J. D. et al. Efficient Yellow Electroluminescence from a Single Layer of a Cyclometalated Iridium Complex. *J. Am. Chem. Soc.* **126**, 2763–2767 (2004).
25. Strieth-Kalthoff, F., Henkel, C., Teders, M., Kahnt, A., Knolle, W., Gómez-Suárez, A., Dirian, K., Alex, W., Bergander, K., Daniliuc, C. G., Abel, B., Guldi, D. M. & Glorius, F. Discovery of Unforeseen Energy-Transfer-Based Transformations Using a Combined Screening Approach. *Chem* **5**, 2183–2194 (201).
26. MassHunter Software Version 10.0, Agilent Technologies Inc., Santa Clara, California, USA.
27. Wenig, P. & J. Odermatt, OpenChrom: a cross-platform open source software for the mass spectrometric analysis of chromatographic data. *BMC Bioinformatics*, **11**, 405 (2010).
28. Tamayo, A. B. et al. Synthesis and Characterization of Facial and Meridional Tris-cyclometalated Iridium(III) Complexes. J. Am. Chem. Soc. 125, 7377–7387 (2003).
29. Crutchley, R. J. & Lever, A. B. P. Ruthenium(II) tris(bipyrazyl) Dication - A New Photocatalyst. *J. Am. Chem. Soc.* 102, 7128−7129 (1980).
30. Bouzaid, J. et al. Supramolecular Selection in Molecular Alloys. *Cryst. Growth Des.* **12**, 3906–3916 (2012).
31. D. D. Méndez-Hernández, P. Tarakeshwar, D. Gust, E. A. Moore, A. L. Moore & V. Mujica. Simple and Accurate Correlation of Experimental Redox Potentials and DFT-calculated HOMO/LUMO energies of Polycyclic Aromatic Hydrocarbons. *J. Mol. Model.***19**, 2845–2848 (2013).

32. D. J. G. Ives, G. J. Janz & C. V. King, Reference Electrodes: Theory and Practice*, J. Electrochemical Society* **108**, 246Ca (1961).
33. N. Elgrish, K. J. Rountree, B. D. McCarthy, E. S. Rountree, T. T. Eisenhart & J. L. Dempsey. A Practical Beginner's Guide to Cyclic Voltammetry. *J. Chem. Educ*. **95**, 197–206 (2019).
34. F. Strieth-Kalthoff, M. J. James, M. Teders, L. Pitzer & F. Glorius. Energy Transfer Catalysis Mediated by Visible Light: Principles, Applications, Directions. *Chem. Soc. Rev.* **47**, 7190–7202.
35. C. K. Prier, D. A. Rankic & D. W. C. MacMillan. Visible Light Photoredox Catalysis with Transition Metal Complexes: Applications in Organic Synthesis. *Chem. Rev.* **113**, 5322–5363 (2013).
36. Luo, J. & Zhang, J. Donor–Acceptor Fluorophores for Visible-Light-Promoted Organic Synthesis: Photoredox/Ni Dual Catalytic C(sp3)–C(sp2) Cross-Coupling. *ACS. Catal.* **6**, 873–877 (2016).
37. Saoud, M., Romerosa, A. & Peruzzini, M. Water-Soluble Ruthenium Vinylidene and Allenylidene Complexes: Potential Catalysts for Ring-Opening Metathesis. *Organometallics* **19**, 4005–4007 (2000).
38. Gottschalk, P. & Neckers, D. C. Low Temperature Free-Radical Reactions Initiated with tert-Butyl p-Benzoylperbenzoate. Selective Acyl Radical Additions to Substituted Olefins. *J. Org. Chem.* **50**, 3498–3502 (1985).
39. Riedel, S., Maichle-Moessmer, C. & Maier, M. E. Intramolecular Diels–Alder Reactions of Tethered Enoate Substituted Furans Induced by Dialkylaluminum Chloride. *J. Org. Chem.* **82**, 12798–12805 (2017).
40. Yu, F., Mao, R., Yu, M., Gu, X. & Wang, Y. Generation of Aryl Radicals from Aryl Halides: Rongalite-Promoted Transition-Metal-Free Arylation. *J. Org. Chem.* **84**, 9946–9965 *(*2019).
41. Sandfort, F., Knecht, T., Pinkert, T., Daniliuc, C. G. & Glorius, F. Site-Selective Thiolation of (Multi)halogenated Heteroarenes. *J. Am. Chem. Soc.* **142**, 6913–6919 (2020).
